# Visible-light-promoted nickel-catalyzed cross-coupling of B-iodocarboranes with styrenes

**DOI:** 10.1039/d6sc04861g

**Published:** 2026-08-17

**Authors:** Xueyu Wang, Hangcheng Ni, Zhipeng Wang, Xinyao Li, Shimeng Li

**Affiliations:** a College of Sciences, Northeastern University Shenyang 110004 China lishimeng@neu.edu.cn; b Department of Chemistry, College of Sciences, Shanghai University Shanghai 200444 China; c Institute of Nuclear and New Energy Technology, Tsinghua University Beijing 100084 China; d College of Pharmacy, Jinhua University of Vocational Technology Jinhua 321007 China

## Abstract

A visible-light-promoted, nickel-catalyzed cross-coupling reaction of B-iodocarboranes with styrenes has been developed, enabling efficient and site-divergent alkenylation of carboranes under mild conditions. The reaction exhibits broad substrate scope, accommodating a wide range of styrene derivatives bearing various electronic and steric properties, as well as ethylene, to afford the corresponding alkenylated products in good to excellent yields. This work provides a practical and sustainable approach for carborane functionalization under ambient conditions and expands the synthetic utility of carboranyl radicals in transition metal-catalyzed Heck-type cross-coupling reactions.

## Abstract 2

A visible-light-promoted, nickel-catalyzed cross-coupling reaction of B-iodocarboranes with styrenes has been developed, enabling efficient and site-divergent alkenylation of carboranes under mild conditions.

## Graphical abstract

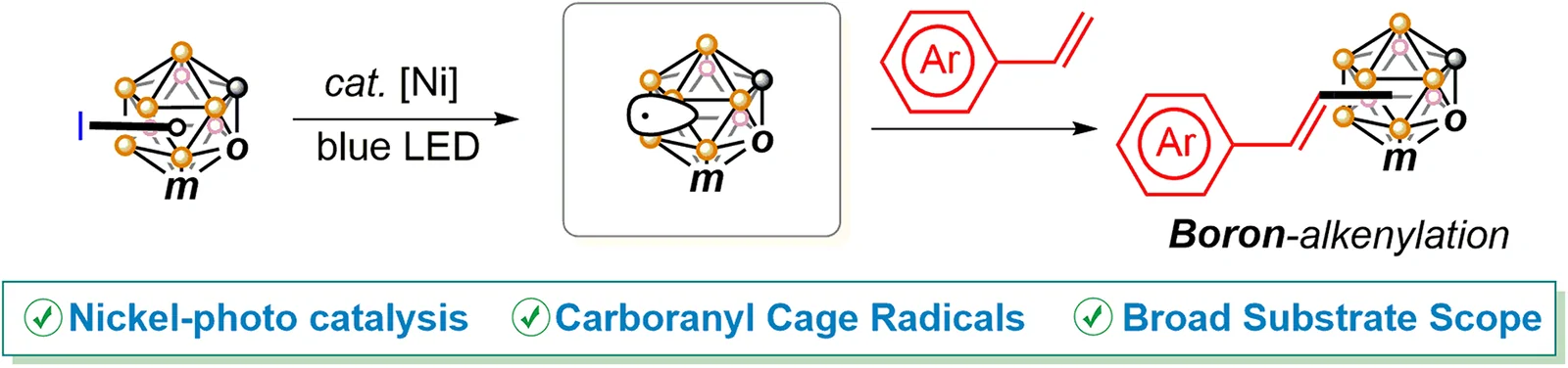

## Introduction

Boron-containing compounds continue to attract growing research attention in a variety of fields, such as materials science, medicinal chemistry, and synthetic chemistry, owing to their unique physicochemical properties and broad application prospects.^1–3^ Among these compounds, boron clusters, particularly polyhedral boranes, occupy a prominent position.^4^ Carboranes (C_2_B_10_H_12_), a quintessential class of boron clusters, are characterized by three-dimensional aromaticity, and their molecular architecture is thus often regarded as a three-dimensional analogue of the benzene ring.^5–8^ These compounds exhibit a combination of distinctive properties, including unique electronic effects, exceptional thermal and chemical stability, molecular rigidity, steric bulk, and high boron content. Such characteristics endow them with significant potential for advanced applications spanning materials science,^9–17^ catalysis,^18–23^ drug discovery,^24–26^ and organometallic/coordination chemistry.^27–35^

Owing to these attributes, there is growing interest in the functionalization of carboranes, particularly at specific vertices.^36–40^ Although conventional methods for vertex-specific functionalization—such as electrophilic/nucleophilic substitutions and metal-catalyzed B–H activation—are well established, methods involving boron-centered carboranyl radicals are relatively less explored and are now drawing growing research interest.^41^ The chemistry of boron-centered carboranyl radicals dates back to the 1980s, when Kabachnik first reported their reactivity.^42,43^ Recently, various approaches have been developed for the formation of boron-centered carboranyl radicals, especially from the B–H bond through the hydrogen atom transfer (HAT) process.^40,44–47^ However, selective B–H radical formation from carboranes with non-equivalent boron atoms remains challenging.

The alkenylation of cage boron atoms in carboranes has emerged as a valuable strategy for the functionalization of these boron-rich clusters. Over the past decades, several methodologies have been developed to address the challenges associated with site-selective B–H activation. Among the early achievements, Sneddon originally discovered the iridium-catalyzed alkenylation of the carborane B–H bond with alkynes in 1988 (Scheme 1a).^48^ The Xie group reported a carboxyl-directed, regioselective dialkenylation of *o*-carborane *via* direct B–H activation in 2015 (Scheme 1b).^49^

**Scheme 1:**
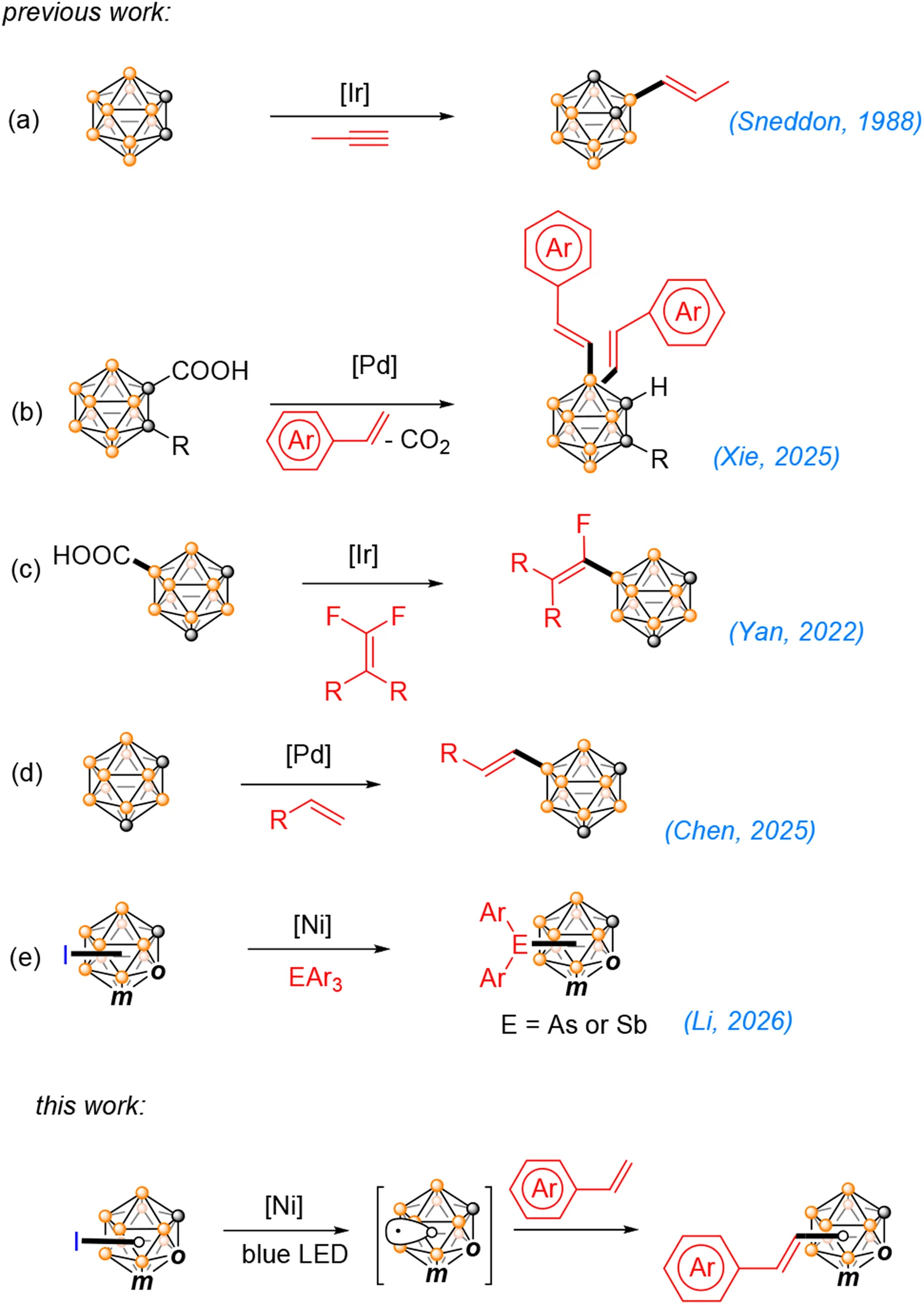
Alkenylation of cage boron in carboranes.

More recently, in 2022, the Yan group developed a decarboxylative strategy for the generation of carboranyl radicals from carboxylic acid-functionalized carboranes, allowing for the alkenylation of *m*-carborane under radical conditions (Scheme 1c).^50^ Concurrently, the Chen group demonstrated a palladium-catalyzed Heck-type reaction that achieved alkenylation at distal boron vertices of *m*-carborane (Scheme 1d).^51^ Despite these notable advances, existing methods are generally applicable to the alkenylation at the B(4) and B(5) vertices of *o*-carborane or at the B(9) position of *m*-carborane, whereas their efficiency for alkenylation at other boron vertices remains to be developed. Recently, our group reported a nickel-catalyzed system that enables efficient and site-diverse functionalization of both *o*- and *m*-carborane (Scheme 1e),^52^ and inspired by the progress in transition metal-catalyzed radical^53–55^ reactions of carboranyl radicals, we envisioned that this radical-based approach could be extended to the alkenylation of carboranes. Such an approach would not only circumvent the need for precious metals but also potentially enable site-divergent functionalization under milder conditions, describing a novel radical-mediated alkenylation protocol that addresses the limitations of previous methods. This approach not only obviates the need for precious metal catalysts but also holds the potential for site-divergent functionalization under milder conditions. Herein, we report the realization of this strategy through the development of a novel radical-mediated alkenylation protocol for carboranes, which effectively addresses the longstanding limitations of previous methods.

## Results and discussion

### Reaction condition optimization

To evaluate the feasibility of the proposed carboranyl boron radical-mediated strategy for carborane alkenylation, 9-iodo-*o*-carborane (1a) was selected as the model substrate for reaction condition optimization, with the results listed in Table 1.

**Table 1:** Optimization of the reaction conditions. Reactions were conducted on a 0.10 mmol scale in a closed flask at room temperature for 48 h under blue LED irradiation (410–510 nm) with an exhaust fan in a cooling water bath. 3 equiv. of 2a were utilized

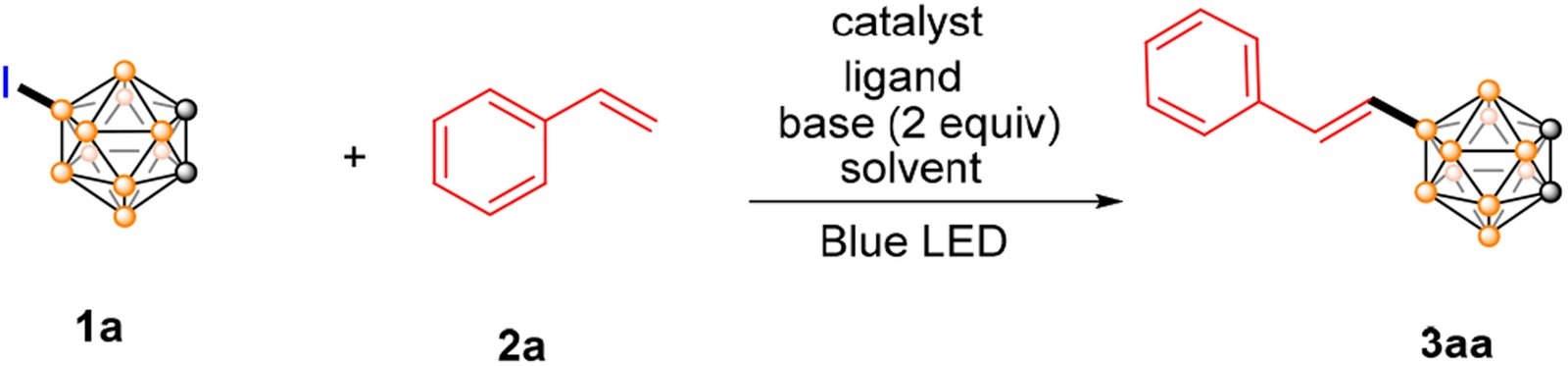				
Entry	Catalyst (mol%)	Ligand (mol%)	Solvent (1 mL)	Yield^a^ (%)
1	Ni(COD)_2_ (10)	PPh_3_ (40)	MeCN	51
2	Ni(COD)_2_ (10)	P(4-Me-C_6_H_4_)_3_ (40)	MeCN	21
3	Ni(COD)_2_ (10)	P(4-MeO-C_6_H_4_)_3_ (40)	MeCN	Trace
4	Ni(COD)_2_ (10)	P(4-CF_3_-C_6_H_4_)_3_ (40)	MeCN	Trace
5	Ni(COD)_2_ (10)	P(4-F-C_6_H_4_)_3_ (40)	MeCN	70
6	Ni(COD)_2_ (10)	P(3-F-C_6_H_4_)_3_ (40)	MeCN	62
7	Ni(COD)_2_ (10)	P(2-F-C_6_H_4_)_3_ (40)	MeCN	68
8	Ni(COD)_2_ (10)	PMePh_2_ (40)	MeCN	n.d.
9	Ni(COD)_2_ (10)	PCy_3_ (40)	MeCN	n.d.
10	Ni(COD)_2_ (10)	Phen (20)	MeCN	n.d.
11	Ni(COD)_2_ (10)	dtbpy (20)	MeCN	n.d.
12^b^	NiBr_2_ (10)	P(4-F-C_6_H_4_)_3_ (40)	MeCN	74
13^b^	NiI_2_ (10)	P(4-F-C_6_H_4_)_3_ (40)	MeCN	80
14^b^	Ni(PPh_3_)_2_I_2_ (10)	P(4-F-C_6_H_4_)_3_ (40)	MeCN	88
15^b^	Ni(PPh_3_)_2_I_2_ (10)	P(4-F-C_6_H_4_)_3_ (40)	DME	75
16^b^	Ni(PPh_3_)_2_I_2_ (10)	P(4-F-C_6_H_4_)_3_ (40)	THF	72
17^b^	Ni(PPh_3_)_2_I_2_ (10)	P(4-F-C_6_H_4_)_3_ (40)	Et_2_O	30
18^b^	Ni(PPh_3_)_2_I_2_ (10)	P(4-F-C_6_H_4_)_3_ (40)	Dioxane	69
19^b^	Ni(PPh_3_)_2_I_2_ (10)	P(4-F-C_6_H_4_)_3_ (60)	MeCN	86
20^b^	Ni(PPh_3_)_2_I_2_ (10)	P(4-F-C_6_H_4_)_3_ (50)	MeCN	88
21^b^	Ni(PPh_3_)_2_I_2_ (10)	P(4-F-C_6_H_4_)_3_ (30)	MeCN	74
22^b^	Ni(PPh_3_)_2_I_2_ (10)	P(4-F-C_6_H_4_)_3_ (20)	MeCN	62
23^a^^,^^c^	Ni(PPh_3_)_2_I_2_ (10)	P(4-F-C_6_H_4_)_3_ (40)	MeCN	48
24^b^^,^^d^	Ni(PPh_3_)_2_I_2_ (10)	P(4-F-C_6_H_4_)_3_ (40)	MeCN	70
25^b^^,^^e^	Ni(PPh_3_)_2_I_2_ (10)	P(4-F-C_6_H_4_)_3_ (40)	MeCN	90
26^b^	—	P(4-F-C_6_H_4_)_3_ (40)	MeCN	n.d.
27^b^^,^^f^	Ni(PPh_3_)_2_I_2_ (10)	P(4-F-C_6_H_4_)_3_ (40)	MeCN	n.d.

a GC-MS yield.

b Extra 2 equiv. of Zn.

c 12 h.

d 24 h.

e 72 h.

f Without blue LED.

With blue LED irradiation, the reaction of 1a with styrene (2a) in the presence of 10 mol% Ni(COD)_2_ and 40 mol% PPh_3_ in CH_3_CN afforded the desired product 3aa in 51% yield (entry 1, Table 1). Subsequent ligand screening indicated that tris(*p*-fluorophenyl)phosphine was the most effective, furnishing 3aa in 70% yield (entries 1–11, Table 1). Replacement of Ni(COD)_2_ with Ni(ii) salts in combination with Zn as a reductant led to improved yields, and using Ni(PPh_3_)_2_I_2_ as the nickel source gave the best result (88% yield, entries 12–14, Table 1). Solvent evaluation identified MeCN as the optimal medium (entries 14–18, Table 1). The ratio of Ni-to-ligand was tested subsequently, and a ratio of 1 : 4 resulted in the best reaction result (entries 14, 19–22, Table 1). Reducing the catalyst loading resulted in diminished yields of 3aa (entries 12–14, Table 1). Investigation of the reaction time indicated that 48 h was optimal (entries 14, 23–25, Table 1). Based on the yield of 3aa, the conditions described in entry 14 of Table 1 were chosen as the optimal reaction parameters.

### Substrate extension

The scope of styrene substrates was examined for their alkenylation potential with carborane, and the synthesized products are presented in Scheme 2. The character of styrene substituents was found to have a limited impact on reaction efficiency, as both electron-donating and electron-withdrawing groups afforded the corresponding products in good to excellent yields with 9-iodo-*o*-carborane (3ab, 3af and 3ai). However, the positions of substituents on the aromatic rings proved influential, with those nearer to the reactive double bond decreasing the yield (3ab, 3ac and 3ad), indicating steric hindrance as a significant parameter. Notably, styrenes bearing aniline, anisole, and even the simple ethylene unit were successfully coupled, delivering the desired alkenylated products (3ah, 3af and 3ag). Furthermore, we examined the reactivity of heterocyclic olefins under the standard conditions. 2-Vinylthiophene reacted smoothly with 1a to afford the corresponding vinylated product 3al in moderate yield. In contrast, 2-vinylpyridine failed to produce the expected product (3am), presumably because the pyridine moiety coordinates to the nickel center, leading to catalyst deactivation and thereby suppressing the generation of the carboranyl boron radical.^50^ To probe the generality of this protocol, we also tested ethylene, which gave the desired product 3am in good yield. This result represents a significant improvement over the previously reported palladium-catalyzed approaches that rely on vinyl Grignard reagents,^56^ as our method avoids the use of sensitive organometallic species and offers a more practical route to 9-vinylated carboranes. The reaction was subsequently extended to 4-iodo- and 3-iodo-*o*-carboranes under slightly modified conditions. The electron density at the boron vertex did not markedly affect the reaction efficiency, although a modest decrease in yield was observed when electron-deficient styrenes were employed (3ai, 3bi and 3ci). Moreover, 1,2-dimethyl-9-iodo-*o*-carborane participated readily in the reaction under the standard conditions, affording products (3da–3di) in yields comparable to those obtained from the parent 9-iodo-*o*-carborane. This result suggests that substituents on the cage carbon atoms exert minimal influence on the radical alkenylation process.

**Scheme 2:**
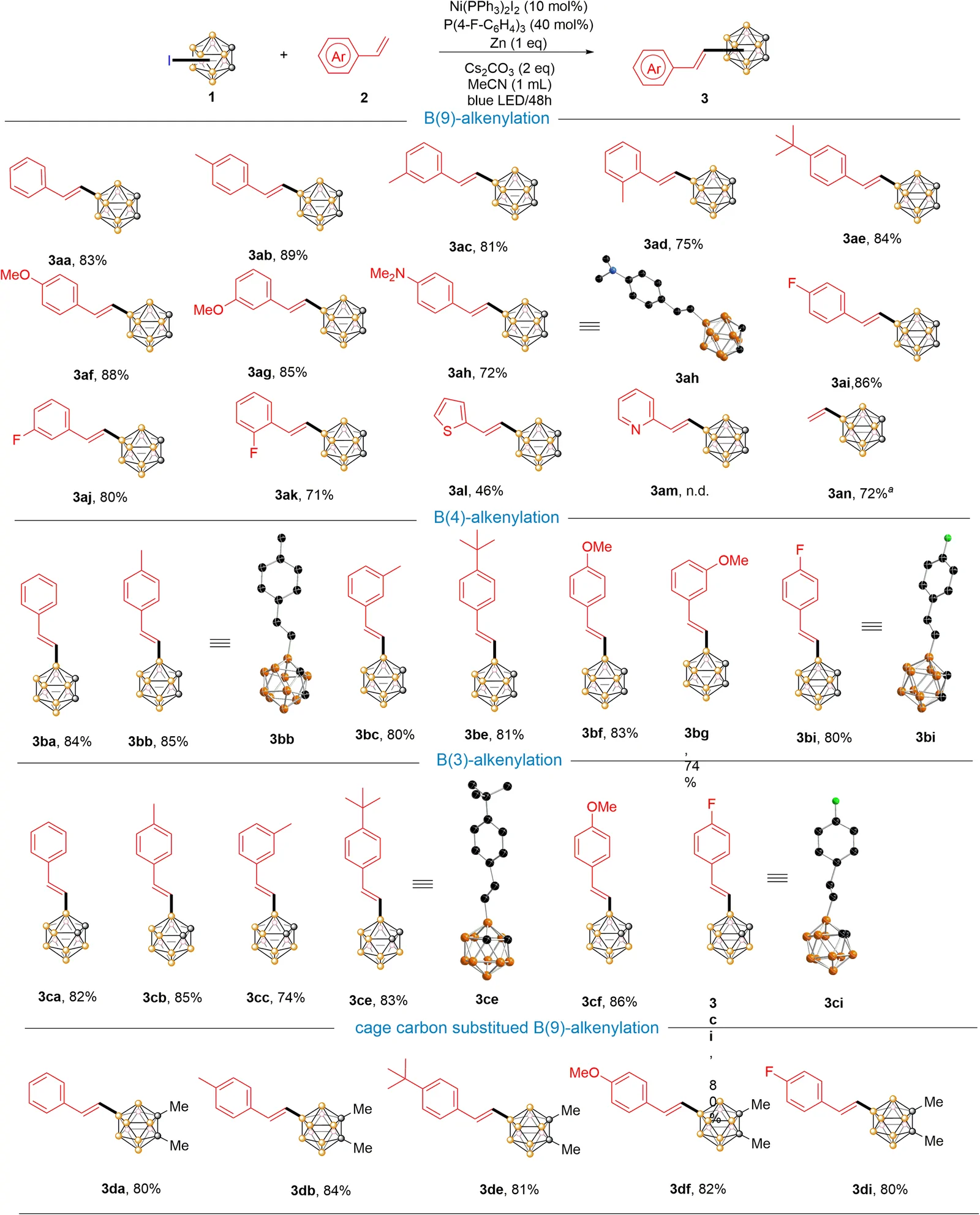
Synthesis of alkenyl-*o*-carboranes. Reactions were conducted on a 0.10 mmol scale in a closed flask at room temperature for 48 h under blue LED irradiation (450 nm) with an exhaust fan in a cooling water bath. 3 equiv. of 2 were utilized. Isolated yields were presented. ^*a*^2 atm of ethylene was utilized.

Furthermore, the synthetic utility of this protocol extended to the dialkenylation of carboranes. Employing 9,12-diiodo-*o*-carborane under the standard conditions afforded the corresponding bis(alkenylated) products in good yields (3ea, 3eb and 3ei), as depicted in Scheme 3.

**Scheme 3:**
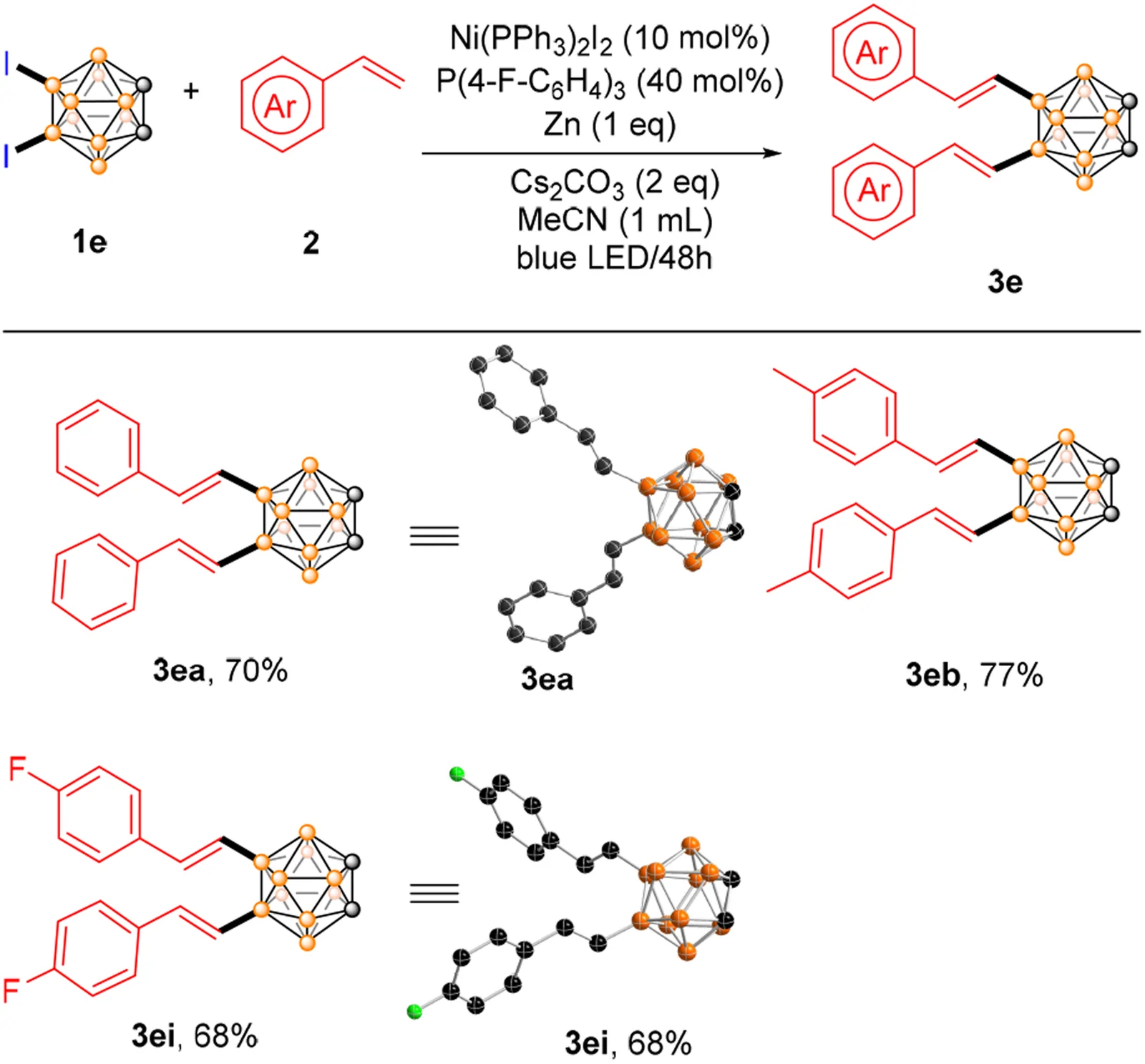
Synthesis of 9,12-dialkenyl-*o*-carboranes. Reactions were conducted on a 0.10 mmol scale in a closed flask at room temperature for 96 h under blue LED irradiation (450 nm, 30 W) with an exhaust fan in a cooling water bath. 5 equiv. of 2 were utilized. Isolated yields were presented.

To further investigate the versatility of this methodology, 9-iodo-*m*-carborane was also evaluated. Under the optimized conditions, the desired alkenylated-*m*-carboranes were obtained in good to excellent yields (Scheme 4).

**Scheme 4:**
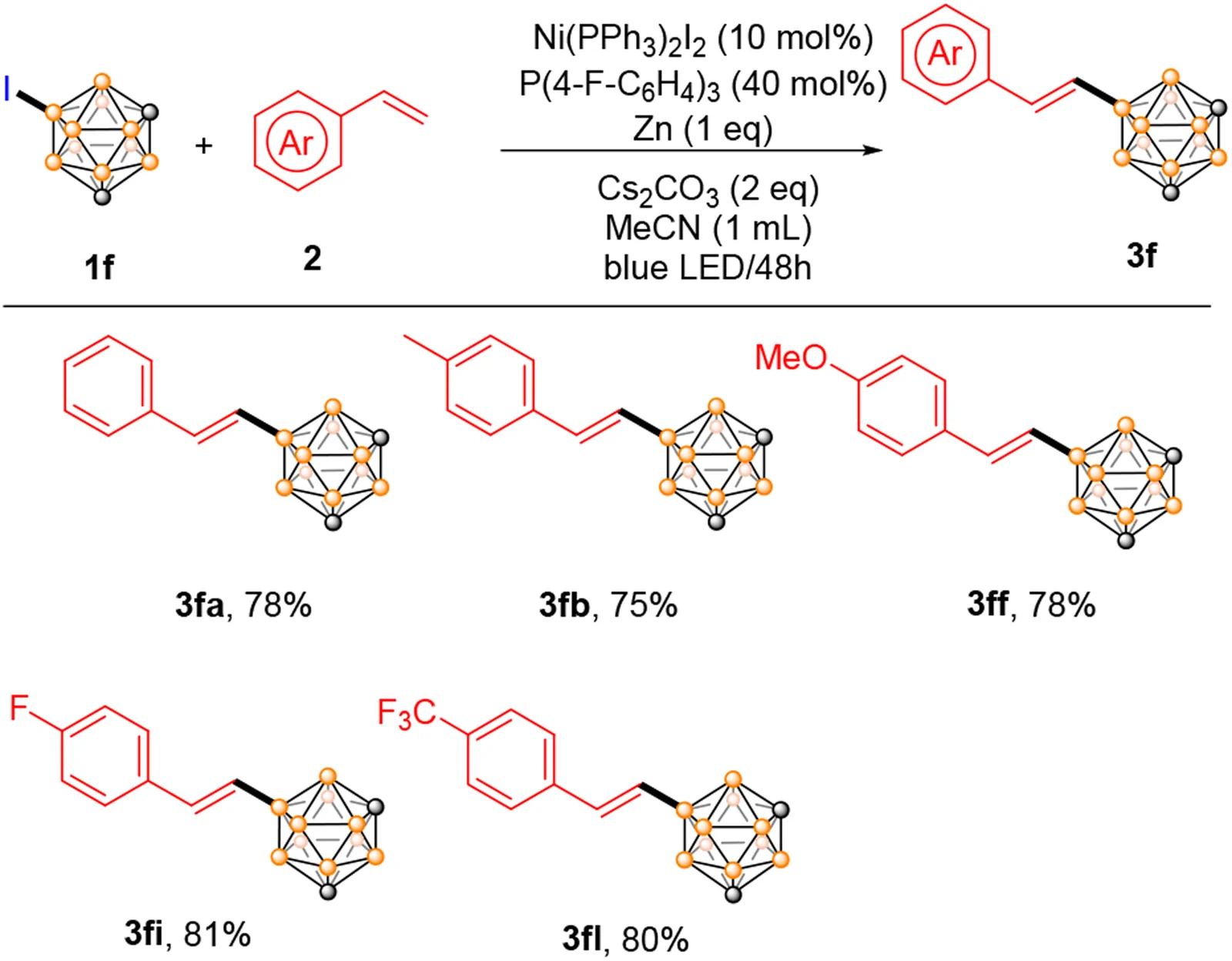
Synthesis of dialkenyl-*m*-carboranes. Reactions were conducted on a 0.10 mmol scale in a closed flask at room temperature for 96 h under blue LED irradiation (450 nm, 30 W) with an exhaust fan in a cooling water bath. 5 equiv. of 2 were utilized. Isolated yields were presented.

### Mechanistic investigation

To gain mechanistic insight into the alkenylation reaction, a series of control experiments were conducted. Based on previous reports demonstrating that 9-*o*-iodo-carborane can generate a carboranyl boron radical under visible-light-mediated nickel catalysis,^52,55^ we employed 9-*o*-iodo-carborane (1a) as a model substrate to further investigate the reaction mechanism of this alkenylation process. As illustrated in Scheme 5a, no product formation was detected in the absence of blue light irradiation, even under extended heating conditions, underscoring the essential role of light in initiating the reaction. Similarly, when the reaction was conducted without light but with additional heating, no formation of 3aa was observed (Scheme 5b and c). To probe radical involvement, 1,1-diphenylethylene (2n) was employed as a radical trapping agent. Under standard conditions, the reaction of 1a with 4a afforded the radical trapping product 5aa (Scheme 5d). In contrast, subjecting 2a to the same conditions in the presence of 2n did not yield any styrene-derived radical trapping product (Scheme 5d). Moreover, a radical clock reaction gave the cyclized product 5ab (Scheme 5e). These results are consistent with the generation of a carboranyl radical intermediate, which has been established in our previous work.

**Scheme 5:**
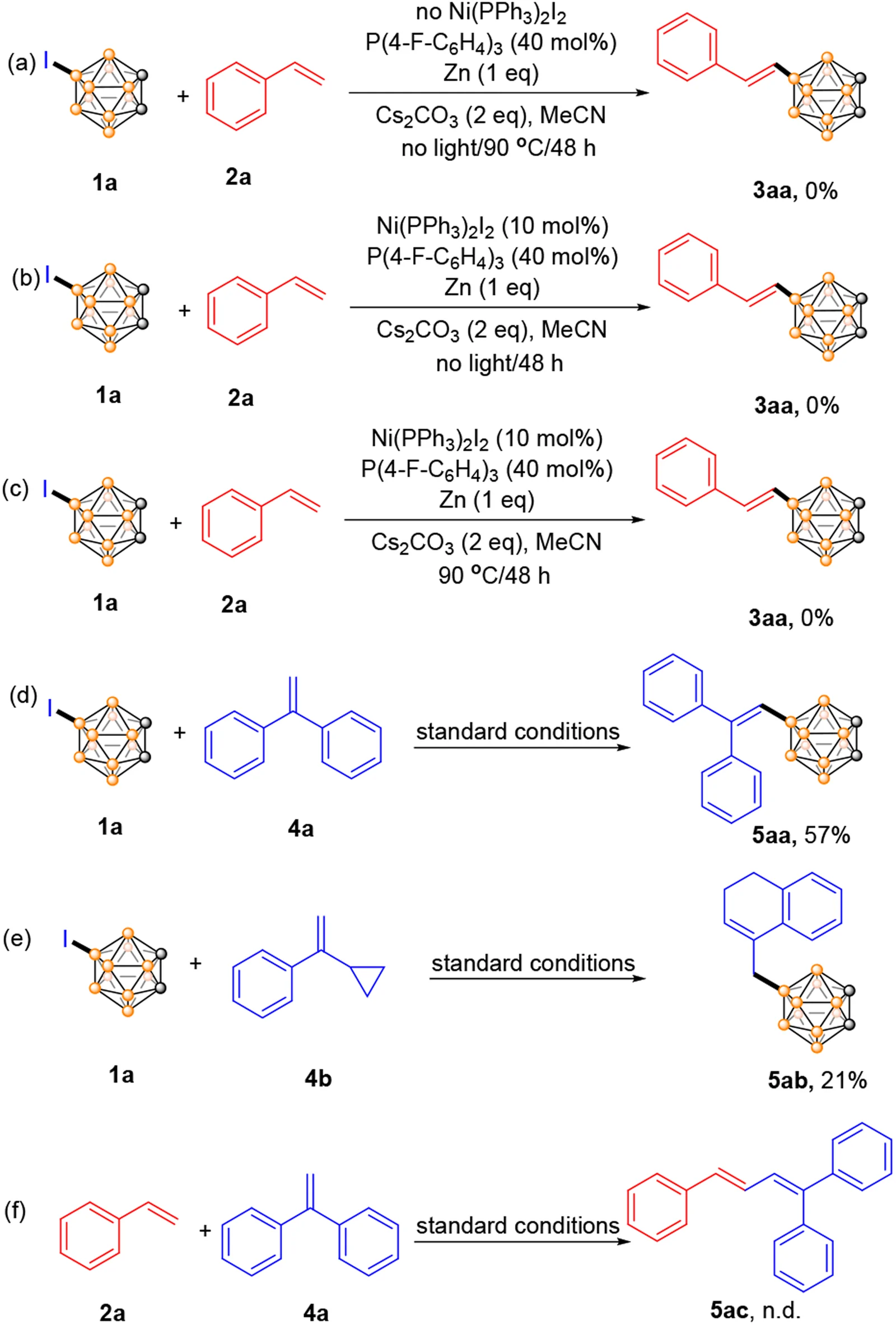
Control experiments.

To elucidate the mechanistic pathway of the visible-light-promoted, nickel-catalyzed B–I Heck reaction of iodo-carboranes, we performed density functional theory (DFT) calculations using 9-iodo-*o*-carborane (1a) and styrene (2a) as model substrates (Scheme 6). Initially, the *in situ* generated catalyst L_2_NiI exhibits a strong interaction with 1a, in which the distance between Ni and I atom of 1a is only 2.76 Å, forming the intermediate complex INT1. The photoexcited Ni(i) complex (INT1*) exhibits a strong absorbance in the visible region and the photoexcitation leads to B–I bond homolysis and an iodine-transfer process, resulting in the generation of a cage boron-centered *o*-carboranyl radical (INT2) and Ni(ii) species L_2_NiI_2_.^40^ The liberated carboranyl radical subsequently adds to styrene (2a) through transition state TS1 with a low energy barrier of 5.0 kcal mol^−1^, generating the benzyl radical intermediate INT3. This process from the boron radical to the carbon radical *via* the addition reaction is a significantly exothermic process. The following single-electron oxidation of the benzyl radical by the Ni(ii) species is facile and exothermic by 8.0 kcal mol^−1^, affording the benzylic carbocation intermediate INT4. Ultimately, in the presence of a base, an E2 elimination reaction affords the alkene product 3a in a significantly exothermic process.

**Scheme 6:**
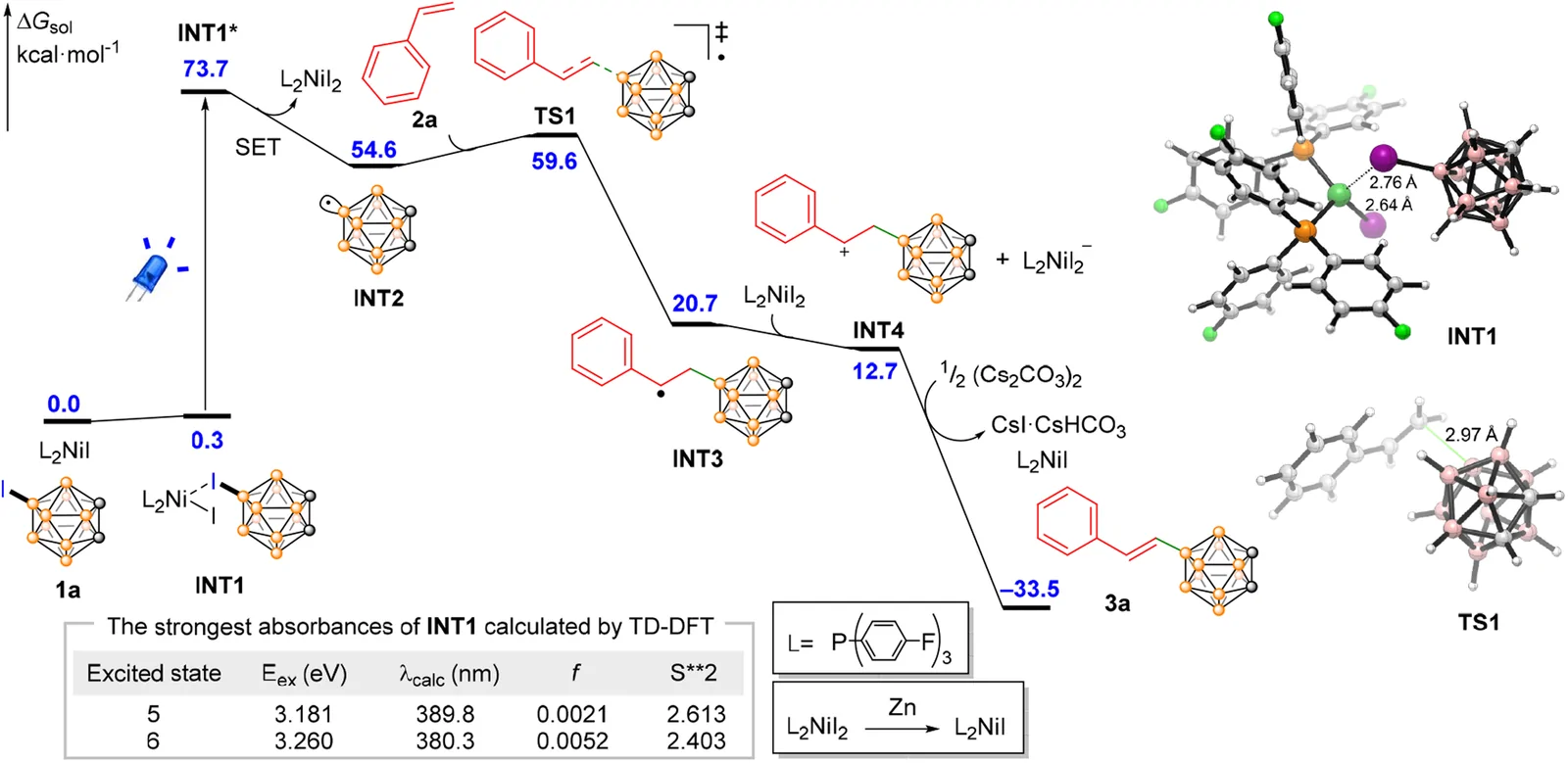
Calculated free-energy profiles of the photoinduced, Ni-catalyzed B–I Heck reaction of *ortho*-carborane. DFT calculations were performed at the (U)M06-D3/def2-TZVP/SMD(MeCN)//(U)B3LYP-D3(BJ)/def2-SVP/SMD(MeCN) level of theory. See the SI for details.

The reduction of the Ni(ii) intermediate back to the active Ni(i) species by zinc powder completes the catalytic cycle. These computational results support a coherent radical-based cross-coupling mechanism and reveal the key roles of visible light and nickel catalysis in enabling the challenging Heck reaction on the carborane cage.

Based on experimental findings and DFT calculations, a plausible reaction mechanism is proposed in Scheme 7. Initially, the Ni(ii) precatalyst is reduced by zinc to generate a Ni(i) species.^53–55,57^ With blue LED irradiation, A facilitates iodine atom abstraction from the iodo-*o*-carborane, producing a reactive carboranyl radical along with a Ni(ii) intermediate (B).^58–60^ The resulting carboranyl radical subsequently adds to the alkene double bond, forming a carbon-centered radical intermediate (C).^54,55,60–70^ Single electron transfer (SET) from this radical to the Ni(ii) species then regenerates the Ni(i) catalyst while affording a cationic intermediate (D).^71–74^ Finally, base-mediated deprotonation of D delivers the desired alkenylated carborane product 3a. The proposed radical pathway is consistent with both the control experiments and the computational results, with key transition states and intermediates further supported by DFT analysis.

**Scheme 7:**
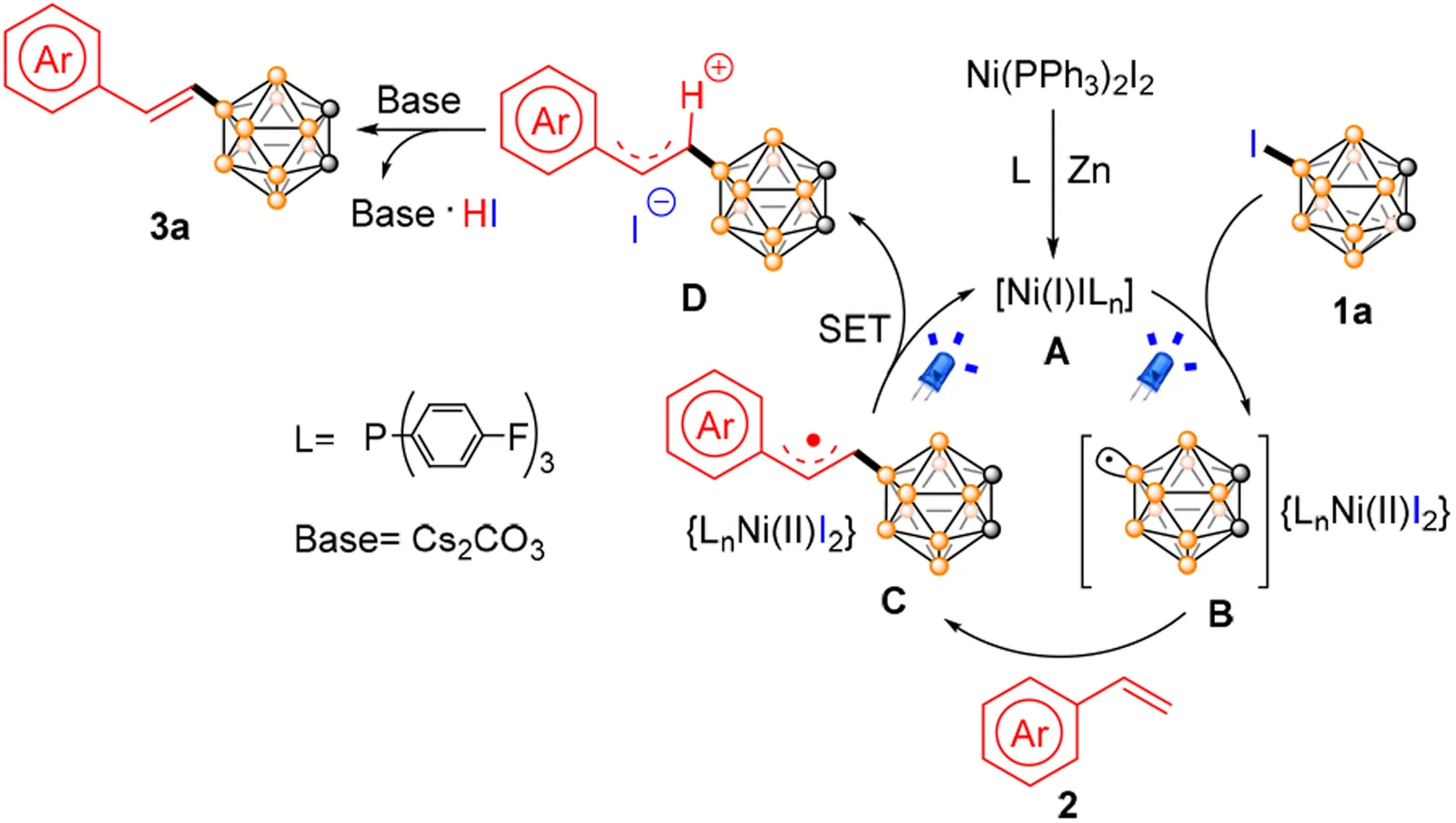
Plausible reaction mechanism.

## Conclusions

In summary, we have developed a visible-light-promoted, nickel-catalyzed cross-coupling reaction of *B*-iodocarboranes with styrenes, enabling efficient and site-divergent alkenylation of carboranes under mild conditions. The reaction exhibits excellent functional group tolerance and accommodates a range of styrene derivatives, including those bearing electron-donating and electron-withdrawing substituents, as well as sterically hindered and heteroaromatic alkenes. Importantly, the method is applicable to multiple boron positions (B3, B4 and B9) in *o*-carborane and extends to both *o*- and *m*-carborane isomers. Mechanistic studies, including control experiments and DFT calculations, support a carboranyl boron radical pathway. This work not only provides a practical and sustainable approach for carborane functionalization under ambient conditions but also expands the synthetic utility of carboranyl radicals in transition metal-catalyzed cross-coupling reactions.

## Experimental

### Catalysts and materials

All reactions were carried out in flame-dried glassware under an atmosphere of dry argon with the exclusion of air and moisture using standard Schlenk techniques or in a drybox. All organic solvents were dried and distilled by standard methods prior to use. Acetone (J.T. Baker, 99.8%), styrene (Sigma-Aldrich, ≥99%), ethylene (Sigma-Aldrich, ≥99.5%), 1,1-diphenylethylene (Sigma-Aldrich, 97%), nickel(ii) bromide (NiBr_2_, Sigma-Aldrich, 99%), nickel(ii) iodide (NiI_2_, Sigma-Aldrich, 99%), bis(triphenylphosphine)nickel(ii) iodide (Ni(PPh_4_)_2_I_2_, Sigma-Aldrich, 98%), triphenylphosphine (PPh_3_, Sigma-Aldrich, 99%), tris(4-fluorophenyl)phosphine (P(4-F-C_6_H_4_)_3_, Sigma-Aldrich, 97%), zinc powder (Zn, Sigma-Aldrich, ≥98%), cesium carbonate (Cs_2_CO_3_, Sigma-Aldrich, 99.9%), acetonitrile (MeCN, Sigma-Aldrich, 99.8%), dimethoxyethane (DME, Sigma-Aldrich, 99.5%), tetrahydrofuran (THF, Sigma-Aldrich, 99.9%), diethyl ether (Et_2_O, Sigma-Aldrich, ≥99.8%), dioxane (Sigma-Aldrich, 99.8%), and deuterated solvents (CDCl_3_, CD_2_Cl_2_ and CD_3_CN, Cambridge Isotope Laboratories) were used as received unless otherwise specified.

The following starting materials were prepared in the laboratory, and detailed synthetic procedures are provided in the SI: 9-iodo-*o*-carborane (1a), 4-iodo-*o*-carborane (1b), 3-iodo-*o*-carborane (1c), 1,2-dimethyl-9-iodo-*o*-carborane (1d), 9,12-diiodo-*o*-carborane (1e), 1,2-diethyl-9-iodo-*o*-carborane (1f), and 9-iodo-*m*-carborane (1g). Silica gel (230–400 mesh, SiliCycle) was used for column chromatography. High-resolution mass spectra (HRMS) were obtained on a Thermo Q Exactive™ Focus Hybrid Quadrupole-Orbitrap™ Mass Spectrometer. GC-MS analyses were performed on an Agilent GC-MS 6890N. NMR spectra were recorded on Bruker DPX 400/500 spectrometers.

### Synthesis

The alkenylated carborane products were synthesized *via* visible-light-promoted, nickel-catalyzed cross-coupling reactions. In a representative procedure for the synthesis of alkenyl-carboranes (3), iodo-carborane (1, 0.10 mmol), the corresponding alkene (2, 0.30 mmol), Ni(PPh_3_)_2_I_2_ (0.01 mmol), P(4-F-C_6_H_4_)_3_ (0.04 mmol), Zn (0.10 mmol), and Cs_2_CO_3_ (0.20 mmol) were mixed in MeCN (1 mL) under an argon atmosphere. The resulting mixture was stirred under irradiation with a 30 W blue LED (450 nm) at room temperature for 48 h. After completion, the reaction mixture was filtered, and the filtrate was concentrated *in vacuo*. The residue was purified by flash column chromatography on silica gel (230–400 mesh) using *n*-hexane and ethyl acetate as the eluent to afford the corresponding alkenylated product. Detailed synthetic procedures for all alkenylated carborane products are provided in the SI.

### Theoretical calculations

All of the DFT calculations were performed with the Gaussian 09 program package at the (U)B3LYP-D3(BJ) level of theory with the def2-SVP basis set using the SMD solvation model with acetonitrile solvent. Single-point solvent calculations were performed at the (U)M06-D3 level of theory with the def2-TZVP basis set using the SMD solvation model with acetonitrile solvent at the optimized geometries. The vertical excitation energies were calculated by time-dependent density functional theory (TD-DFT) calculations at the (U)M06-D3/def2-TZVP/SMD(MeCN) level of theory. All of the energies discussed in the paper are Gibbs free energies in acetonitrile solution (Δ*G*_sol_). Computational details and references are given in the SI.

## Author contributions

Conceptualization, S. L.; data curation, X. W.; methodology, H. N.; investigation, Z. W. and X. L.; writing – original draft, S. L.; writing – review & editing, S.L. and X. L.; funding acquisition, S. L.; resources, S. L. and Z. W.; supervision, S. L. and X. L.

## Conflicts of interest

There are no conflicts to declare.

## Supplementary Material

SC-017-D6SC04861G-s001

SC-017-D6SC04861G-s002

## Data Availability

CCDC 2533189–2533195 contain the supplementary crystallographic data for this paper.^75*a*–*g*^ The data supporting the findings of this study are available within the article and its supplementary information (SI) files. Supplementary information: additional figures, tables, and experimental details supporting the findings of this study. See DOI: https://doi.org/10.1039/d6sc04861g.
