# Supplementary material for: Visible-light-promoted nickel-catalyzed cross-coupling of B-iodocarboranes with styrenes
Source: Chem Sci. 2026 Aug 17;17(39):18896–904. doi: 10.1039/d6sc04861g (PMC13501473; doi:10.1039/d6sc04861g)
Supplement: Supplementary file 1 [file SC-017-D6SC04861G-s001.pdf]

Supplementary Information (SI) for Chemical Science.
This journal is © The Royal Society of Chemistry 2026

Supplementary Information

Visible-Light-Promoted Nickel-Catalyzed Cross-Coupling of Iodocarboranes with Styrenes

Xueyu Wang,†[a] Hangcheng Ni,†[d] Zhipeng Wang,*[c] Xinyao Li,*[b] and Shimeng Li*[a]

[a.] *College of Sciences, Northeastern University, Shenyang, 110004, China.*

[b.] *Department of Chemistry, College of Sciences, Shanghai University, Shanghai 200444, China.*

[c.] *Institute of Nuclear and New Energy Technology, Tsinghua University, Beijing 100084, China.*

[d.] *College of Pharmacy, Jinhua University of Vocational Technology, Jinhua, 321007, China.*

†These authors contributed equally to this work

Contents

1 General Experimental Procedures 3

2 Experimental Section 3

3 X-ray Structure Determination 30

4 DFT Calculations 34

4.1 Calculational details for the energy profiles 34

4.2 DFT-Computed Energies of All Stationary Points 35

5 Reference 43

6 NMR Spectra of New Compound 45

1 General Experimental Procedures

All reactions were carried out in flame-dried glassware under an atmosphere of dry argon with the exclusion of air and moisture using standard Schlenk techniques or in a drybox. All organic solvents were dried and distilled by standard methods prior to use. ^{1}H, ^{13}C, ^{11}B and ^{19}F spectra were recorded on Bruker DPX 400/500 spectrometer at 400/500, 100/125, 128/160 and 376/470 MHz, respectively. All chemical shifts were reported in δ units with reference to the residual solvent resonances of the deuterated solvents for proton and carbon chemical shifts, to external $BF_3{\cdot}OEt_2$ (0.00 ppm) for boron chemical shifts, and to external $CFCl_3$ (0.00) for fluorine chemical shifts. High Resolution Mass Spectra (HRMS) were obtained on a Thermo Q Exactive™ Focus Hybrid Quadrupole-Orbitrap™ Mass Spectrometer. GC-MS analyses were performed on Agilent GC-MS 6890N. Compounds **1** were prepared according to the literature methods.[1-3] All other chemicals were purchased from either Aldrich or Acros Chemical Co. and used as received unless otherwise specified.

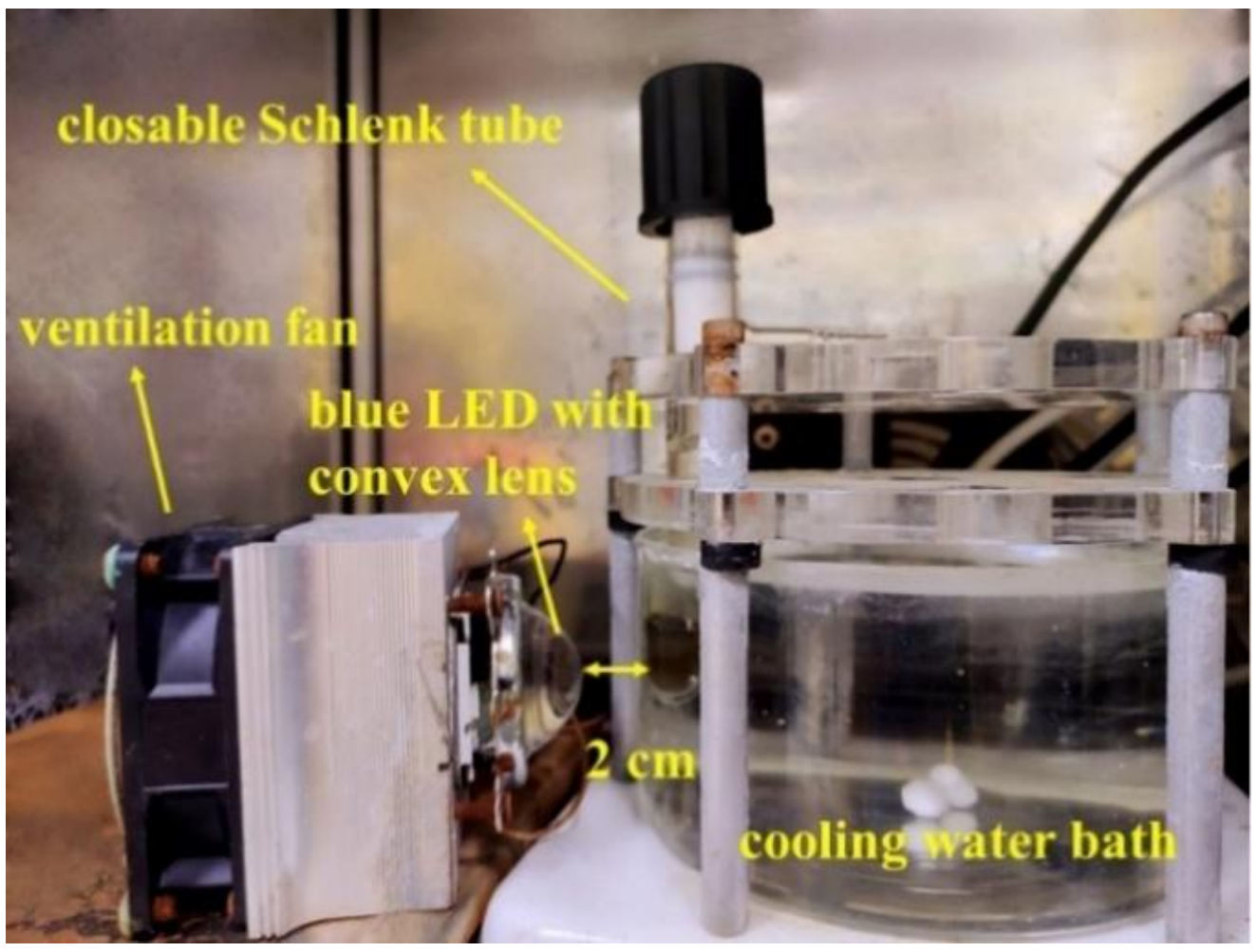

Fig. S1. General reaction setup.

2 Experimental Section

Preparation of 9-Iodo-*o*-Carborane (1a). According to the literature procedure,[1]

a mixture of *o*-carborane (1.440 g, 10.0 mmol), I_2 (2.540 g, 10.0 mmol) and a catalytic amount of $AlCl_3$ (0.130 g, 1.0 mmol) in CH_2Cl_2 (20 mL) was refluxed for 6 h. The clear yellow solution was poured into distilled water and the organic layer was separated. The aqueous solution was extracted with CH_2Cl_2 (15 mL × 3), and the combined organic portions were dried over Na_2SO_4. After filtration, the solution was concentrated under reduced pressure. Recrystallization of the residue from n-hexane gave **1a** as a white solid (2.030 g, 75%). ^1H NMR (500 MHz, $CDCl_3$): δ 3.87 (s, 1H), 3.76 (s, 1H) (cage *H*). $^{11}B\{^1H\}$ NMR (128 MHz, $CDCl_3$): δ -0.9 (1B), -7.1 (2B), -12.1, -12.9, -13.8 (6B), -16.5 (1B) (*B*-I). The data were the same as the reported ones.[1]

Preparation of 4-Iodo-*o*-Carborane (1b). According to the literature procedure,[2] a mixture of *o*-carborane monocarboxylic acid (1.880 g, 10.0 mmol), $[Cp^*IrCl_2]_2$ (0.200 g, 0.25 mmol), $AgNTf_2$ (0.390 g, 1.0 mmol), NaOAc (0.820 g,10.0 mmol) and NIS (N-iodosuccinimide) (2.250 g, 10.0 mmol) was dissolved in DCE (20 mL) under argon atmosphere. The resulting mixture was heated in a closed flask at 100 °C for 12 h. After hydrolysis with water (20 mL) and extraction with diethyl ether (15 mL × 3), the ether solutions were combined and concentrated to dryness in vacuo. The residue was subjected to flash column chromatography on silica gel (230-400 mesh) using n-hexane and ethyl acetate (500/1 in v/v) as eluent to give **1b** as a white solid (2.380 g, 88%). ^1H NMR (500 MHz, $CDCl_3$): δ 3.95 (s, 1H), 3.73 (s, 1H) (Cage *H*). $^{11}B\{^1H\}$ NMR (128 MHz, $CDCl_3$): δ -0.4 (1B), -1.4 (1B), -6.8 (1B), -8.3 (1B), -10.1 (1B), -12.5 (3B), -14.8 (1B), -27.0 (1B) (*B*-I). The data were the same as the reported ones.[2]

Preparation of 3-Iodo-*o*-Carborane (1c). According to the literature procedure,[3] lithium dicarbollide (1.550 g, 10.0 mmol) was suspended in anhydrous hexane (10 mL),

and the resultant solution was cooled to 0 °C under an argon atmosphere, which was slowly added via syringe a solution of boron triiodide (7.820 g, 20.0 mmol) in anhydrous hexane (10 mL). Then, the reaction mixture was allowed to warm to room temperature and stirred at room temperature for 18 h. The bright orange precipitate was removed by filtration, and washed with water (20 mL). The aqueous solution was extracted with diethyl ether (10 mL × 3). The ether solutions were combined and concentrated to dryness in vacuo. The residue was subjected to flash column chromatography on silica gel (230-400 mesh) using n-hexane as eluent to give the product **1c** as a white solid (1.120 g, 42%). ^{1}H NMR (500 MHz, CD_2Cl_2): *δ* 3.95 (s, 2H) (Cage *H*). ^{11}B{^{1}H} NMR (128 MHz, CD_2Cl_2): *δ* -0.9 (2B), -7.0 (1B), -10.8 (3B), -12.2 (3B), -29.2 (1B) (*B*-I). The data were the same as the reported ones.[1]

Preparation of 1,2-Dimethyl-9-Iodo-*o*-Carborane (1d). According to the literature procedure,[1] a mixture of 1,2-dimethyl-*o*-carborane (1.740 g, 10.0 mmol), I_2 (2.540 g, 10.0 mmol) and a catalytic amount of $AlCl_3$ (0.130 g, 1.0 mmol) in CH_2C1_2 (20 mL) was refluxed for 6 h. The clear yellow solution was poured into distilled water, and the organic layer was separated. The aqueous solution was extracted with CH_2Cl_2 (15 mL × 3), and the combined organic portions were dried over Na_2SO_4. After filtration and removal of the solvent in vacuo, the residue was recrystallized from hexane to give **1d** as a white solid (2.080 g, 70%). ^{1}H NMR (500 MHz, $CDCl_3$): *δ* 2.06 (s, 3H), 1.91 (s, 3H) (C*H₃*). ^{11}B{^{1}H} NMR (128 MHz, $CDCl_3$): *δ* -3.3 (1B), -7.4, -8.2 (8B), -17.7 (1B) (*B*-I). The data were the same as the reported ones.[1]

Preparation of 9,12-Diiodo-*o*-Carborane (1e). According to the literature procedure,[1] a mixture of *o*-carborane (1.440 g, 10.0 mmol), I_2 (2.540 g, 10.0 mmol) and

a catalytic amount of $AlCl_3$ (0.130 g, 1.0 mmol) in CH_2Cl_2 (20 mL) was refluxed for 6 h. To which was added a second equivalent of I_2 (2.540 g, 10.0 mmol) and $AlCl_3$ (0.130 g, 1.0 mmol), and the reaction mixture was heated at 45°C overnight. The resultant dark brown reaction mixture was diluted with H_2O (20 mL), and unreacted I_2 was quenched with $Na_2S_2O_3$ (1.580 g, 10.0 mmol). After stirring for 10 minutes, the purple organic layer was separated, and the aqueous layer was washed with CH_2Cl_2 (10 mL x 3). The combined organic solutions were dried over Na_2SO_4. This solution was passed through a silica plug and eluted with CH_2Cl_2 to give a light gray solution. After removal of the solvent in vacuo, the residue was recrystallized from hexane and ethyl acetate (10/1 in v/v) to give **1e** as a light-yellow solid (2.420 g, 61%). ^{1}H NMR (500 MHz, $CDCl_3$): δ 3.98 (s, 2H) (cage C-*H*). ^{11}B{^{1}H} NMR (128 MHz, $CDCl_3$): δ -5.1 (2B), -12.1 (4B), -13.9 (4B) (2*B*-I + 2 BH). The data were the same as the reported ones.

Preparation of 1,2-Diethyl-9-Iodo-*o*-Carborane (1f). According to the literature procedure,[1] a mixture of 1,2-diethyl-*o*-carborane (1.980 g, 10.0 mmol), I_2 (2.540 g, 10.0 mmol) and a catalytic amount of $AlCl_3$ (0.130 g, 1.0 mmol) in CH_2Cl_2 (20 mL) was refluxed for 6 h. The clear yellow solution was poured into distilled water, and the organic layer was separated. The aqueous solution was extracted with CH_2Cl_2 (15 mL × 3), and the combined organic portions were dried over Na_2SO_4. After filtration and removal of the solvent in vacuo, the residue was recrystallized from hexane to give **1f** as a white solid (2.217 g, 68%). ^{1}H NMR (400 MHz, $CDCl_3$): δ 2.24 (q, J = 7.6 Hz, 2H), 2.13 (q, J = 7.6 Hz, 2H) (CH_2), 1.20 (t, J = 7.6 Hz, 3H), 1.13 (t, J = 7.6 Hz, 3H) (CH_3). ^{11}B{^{1}H} NMR (128 MHz, $CDCl_3$): δ -3.3 (1B), -8.7 (2B), -10.1, -10.8 (6B), -17.6 (1B) (*B*-I). The data were the same as the reported ones.[1]

Preparation of 9-Iodo-*m*-Carborane (1g). According to the literature procedure,[1]

a mixture of *m*-carborane (1.440 g, 10.0 mmol), I_2 (2.540 g, 10.0 mmol) and a catalytic amount of $AlCl_3$ (0.130 g, 1.0 mmol) in CH_2Cl_2 (20 mL) was refluxed for 6 h. The clear yellow solution was poured into distilled water and the organic layer was separated. The aqueous solution was extracted with CH_2Cl_2 (15 mL × 3), and the combined organic portions were dried over Na_2SO_4. After filtration, the solution was concentrated under reduced pressure. Recrystallization of the residue from n-hexane gave **1g** as a white solid (2.106 g, 78%). ^1H NMR (400 MHz, $CDCl_3$): δ 3.05 (s, 2H) (cage *H*). $^{11}B\{^1H\}$ NMR (128 MHz, $CDCl_3$): δ -5.4 (2B), -8.3 (1B) -11.9 (2B), -13.0 (2B), -16.8 (1B), -18.9 (1B), -23.5 (1B) (*B*-I). The data were the same as the reported ones.[1]

Preparation of B(9)-Alkenyl-*o*-Carboranes (3a). A Representative Procedure. **1a** (0.10 mmol), alkene **2** (3 equiv, 0.3 mmol), $Ni(PPh_3)_2I_2$ (0.1 equiv, 8.4 mg, 0.01 mmol), $P(4\text{-}F\text{-}C_6H_4)_3$ (0.4 equiv, 12.8 mg, 0.04 mmol), Zn (1 equiv, 6.4 mg, 0.1 mmol) and Cs_2CO_3 (2 equiv, 65.2 mg, 0.2 mmol) were mixed in MeCN (1 mL). The resulting mixture was stirred with 30W blue LED for 48 h under argon. After reaction, the reaction mixture was filtered through a suction funnel and washed with diethyl ether. The filtrate was concentrated to dryness in vacuo, which was subjected to flash column chromatography on silica gel (230-400 mesh) using *n*-hexane and ethyl acetate (500/1 in V/V) as eluent to give the products **3a**.

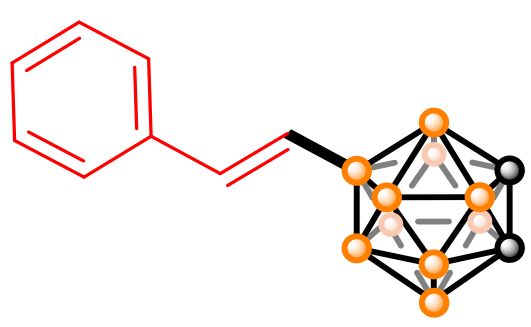

3aa: R_f (hexane/EA = 5:1): 0.65. 20.2 mg, 83% yield. White solid. Mp: 131-132 °C. ^1H NMR (500 MHz, CD_3CN): δ 7.37 (d, *J* = 7.0 Hz, 2H), 7.29 (t, *J* = 7.6 Hz, 2H), 7.21 (d, *J* = 7.3 Hz, 1H) (aryl C*H*), 6.58 (d, *J* = 18.0 Hz, 1H), 6.33 (d, *J* = 18.0 Hz, 1H) (alkenyl C*H*), 4.07 (s,

1H), 3.99 (s, 1H) (Cage C*H*). $^{13}C\{^1H\}$ NMR (125 MHz, CD_3CN): δ 139.7, 129.5, 128.2, 126.9 (aryl and alkenyl *C*), 55.7, 51.0 (cage *C*H). Bcage-*C* was not observed. $^{11}B\{^1H\}$ NMR (160 MHz, CD_3CN): δ 7.1 (1B) (*B*-C), -2.8 (1B), -8.9 (2B), -13.6 - -15.0 (6B). HRMS (APCI): *m/z* calcd for $C_{10}H_{18}{}^{10}B_2{}^{11}B_8$ $[M+H]^+$: 245.2344. Found: 245.2339.

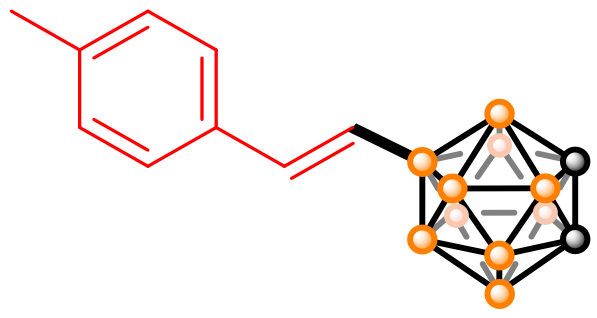

3ab: R_f (hexane/EA = 5:1): 0.68. 23.1 mg, 89% yield. White solid. Mp: 139-140 °C. ^1H NMR (500 MHz, CD_3CN): δ 7.26 (d, *J* = 8.2 Hz, 2H), 7.11 (d, *J* = 7.9 Hz, 2H) (aryl C*H*), 6.54 (d, *J* = 18.0 Hz, 1H), 6.26 (d, *J* = 18.0 Hz, 1H) (alkenyl C*H*), 4.06 (s, 1H), 3.98 (s, 1H) (Cage C*H*), 2.29 (s, 3H) (CH_3). $^{13}C\{^1H\}$ NMR (125 MHz, CD_3CN): δ 139.6, 138.1, 130.1, 126.8 (aryl and alkenyl *C*), 55.6, 51.0 (cage *C*H), 21.1 (CH_3). Bcage-*C* was not observed. $^{11}B\{^1H\}$ NMR (160 MHz, CD_3CN): δ 5.9 (1B) (*B*-C), -2.7 (1B), -9.0 (2B), -13.5 - -14.9 (6B). HRMS (APCI): *m/z* calcd for $C_{11}H_{20}{}^{10}B_2{}^{11}B_8$ $[M-H]^-$: 259.2501. Found: 259.2496.

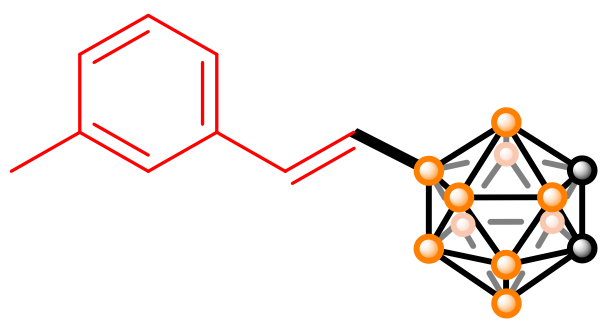

3ac: R_f (hexane/EA = 5:1): 0.68. 20.2 mg, 81% yield. White solid. Mp: 145-146 °C. ^1H NMR (500 MHz, CD_3CN): δ 7.20-7.15 (m, 3H), 7.03 (d, *J* = 6.1 Hz, 1H) (aryl C*H*), 6.55 (d, *J* = 18.0 Hz, 1H), 6.32 (d, *J* = 18.0 Hz, 1H) (alkenyl C*H*), 4.06 (s, 1H), 3.98 (s, 1H) (Cage C*H*), 2.30 (s, 3H) (CH_3). $^{13}C\{^1H\}$ NMR (125 MHz, CD_3CN): δ 139.8, 139.0, 129.4, 129.0, 127.6, 124.1 (aryl and alkenyl *C*), 55.6, 51.0 (cage *C*H), 21.4 (CH_3). Bcage-*C* was not observed. $^{11}B\{^1H\}$ NMR (160 MHz, CD_3CN): δ 5.8 (1B) (*B*-C), -2.7 (1B), -9.0 (2B), -13.5 - -14.9 (6B). HRMS (APCI): *m/z* calcd for $C_{11}H_{20}{}^{10}B_2{}^{11}B_8$ [M-H]-: 259.2501. Found: 259.2495.

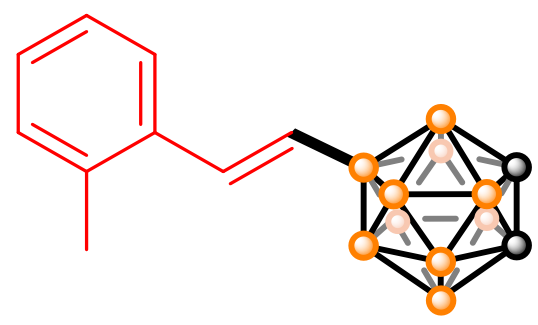

3ad: R_f (hexane/EA = 5:1): 0.66. 19.5 mg, 75% yield. White solid. Mp: 142−143 °C. ^{1}H NMR (500 MHz, CD_3CN): δ 7.45-7.36 (m, 1H), 7.10 (s, 3H) (aryl C*H*), 6.83 (d, *J* = 17.7 Hz, 1H), 6.21 (d, *J* = 17.8 Hz, 1H) (alkenyl C*H*), 4.03 (s, 1H), 3.95 (s, 1H) (Cage C*H*), 2.30 (s, 3H) (CH_3). ^{13}C{^{1}H} NMR (125 MHz, CD_3CN): δ 138.6, 137.5, 136.0, 131.1, 128.1, 127.0, 125.7 (aryl and alkenyl *C*), 55.6, 51.0 (cage *C*H), 19.7 (*C*H_3). Bcage-*C* was not observed. ^{11}B{^{1}H} NMR (160 MHz, CD_3CN): δ 5.9 (1B) (*B*-C), -2.6 (1B), -8.9 (2B), -13.4 – -14.8 (6B). HRMS (APCI): *m/z* calcd for $C_{11}H_{20}{}^{10}B_2{}^{11}B_8$ $[M-H]^-$: 259.2501. Found: 259.2495.

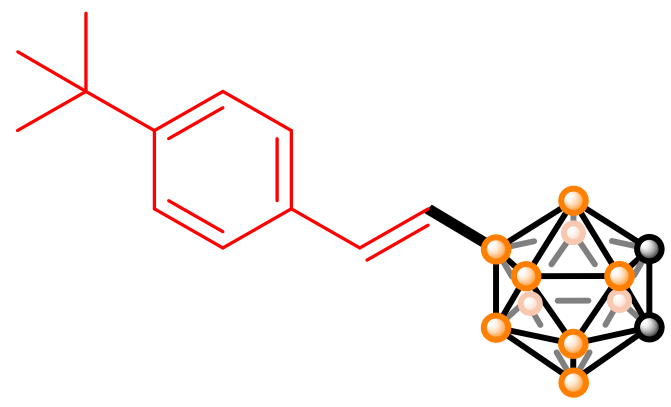

3ae: R_f (hexane/EA = 5:1): 0.70. 25.4 mg, 84% yield. White solid. Mp: 157−158 °C. ^{1}H NMR (500 MHz, CD_3CN): δ 7.35−7.32 (m, 2H), 7.31−7.28 (m, 2H), 6.55 (d, *J* = 18.0 Hz, 1H), 6.28 (d, *J* = 18.0 Hz, 1H) (alkenyl C*H*), 4.06 (s, 1H), 3.98 (s, 1H) (Cage C*H*), 1.28 (s, 9H) (C-CH_3). ^{13}C{^{1}H} NMR (125 MHz, CD_3CN): δ 151.3, 139.5, 136.9, 126.6, 126.3 (aryl and alkenyl *C*), 55.6, 51.0 (cage *C*H), 35.1 (*C*-CH_3), 31.5 (C-*C*H_3). Bcage-*C* was not observed. ^{11}B{^{1}H} NMR (160 MHz, CD_3CN): δ 3.2 (1B) (*B*-C), -5.4 (1B), -8.8 – -10.4 (6B). HRMS (APCI): *m/z* calcd for $C_{14}H_{26}{}^{10}B_2{}^{11}B_8$ $[M+H]^+$: 303.3117. Found: 303.3109.

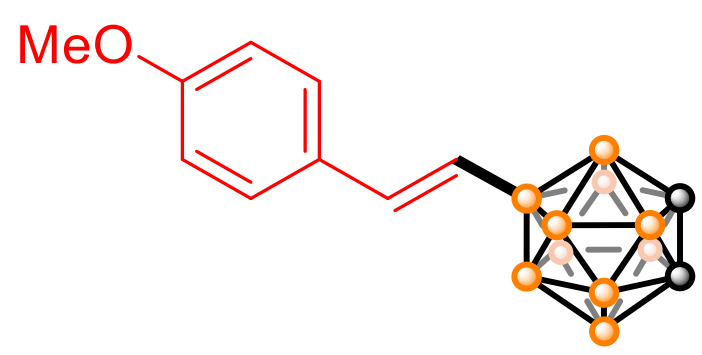

3af: R_f (hexane/EA = 5:1): 0.65. 24.3 mg, 88% yield. White solid. Mp: 148−150 °C. ^{1}H NMR (500 MHz, CD_3CN): δ 7.30 (d, *J* = 8.7 Hz, 2H), 6.84 (d, *J* = 8.6 Hz, 2H) (aryl C*H*), 6.52 (d, *J* = 17.9 Hz, 1H), 6.16 (d, *J* = 17.9 Hz, 1H) (alkenyl C*H*), 4.04 (s, 1H), 3.96 (s, 1H) (Cage C*H*), 3.76 (s, 3H) (OCH_3). ^{13}C{^{1}H} NMR (125 MHz, CD_3CN): δ 160.1, 139.1, 132.4, 128.1, 114.8 (aryl and alkenyl *C*), 55.8 (O*C*H_3), 55.6,

50.7 (cage *C*H). Bcage-*C* was not observed. $^{11}B\{^1H\}$ NMR (160 MHz, CD_3CN): *δ* 6.0 (1B) (*B*-C), -2.7 (1B), -9.0 (2B), -13.5 – -14.9 (6B). HRMS (APCI): *m/z* calcd for $C_{11}H_{20}{}^{10}B_2{}^{11}B_8O$ $[M+H]^+$: 277.2596. Found: 277.2590.

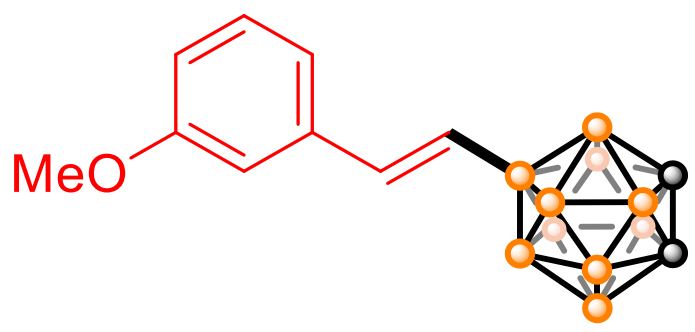

3ag: R_f (hexane/EA = 5:1): 0.66. 23.5 mg, 85%. White solid. Mp: 150−152 °C. ^1H NMR (500 MHz, CD_3CN): *δ* 7.20 (t, *J* = 7.9 Hz, 1H), 6.96−6.93 (m, 2H), 6.78−6.76 (m, 1H) (aryl C*H*), 6.55 (d, *J* = 18.0 Hz, 1H), 6.35 (d, *J* = 18.0 Hz, 1H) (alkenyl C*H*), 4.07 (s, 1H), 3.99 (s, 1H) (Cage C*H*), 3.77 (s, 3H) (OCH_3). $^{13}C\{^1H\}$ NMR (125 MHz, CD_3CN): *δ* 160.8, 141.1, 139.6, 130.4, 119.5, 114.0, 117.6 (aryl and alkenyl *C*), 55.7 (O*C*H_3), 55.6, 51.0 (cage *C*H). Bcage-*C* was not observed. $^{11}B\{^1H\}$ NMR (160 MHz, CD_3CN): *δ* 5.7 (1B) (*B*-C), -2.7 (1B), -9.0 (2B), -13.5 – -14.9 (6B). HRMS (APCI): *m/z* calcd for $C_{11}H_{20}{}^{10}B_2{}^{11}B_8O$ $[M+H]^+$: 277.2596. Found: 277.2592.

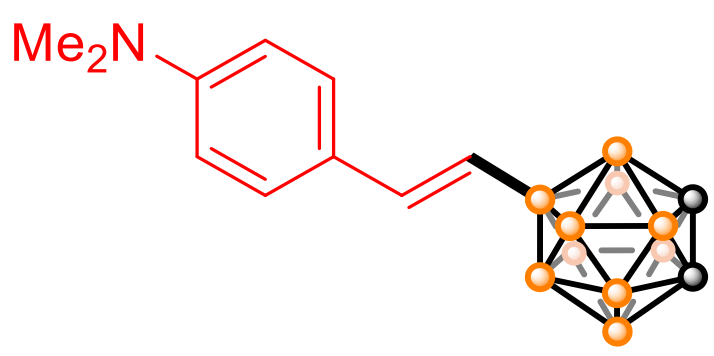

3ah: R_f (hexane/EA = 5:1): 0.42. 20.8 mg, 72% yield. White solid. Mp: 169−170 °C. ^1H NMR (500 MHz, CD_3CN): *δ* 7.22 (d, *J* = 8.8 Hz, 2H), 6.66 (d, *J* = 8.8 Hz, 2H) (aryl C*H*), 6.45 (d, *J* = 17.9 Hz, 1H), 6.04 (d, *J* = 18.0 Hz, 1H) (alkenyl C*H*), 4.04 (s, 1H), 3.96 (s, 1H) (Cage C*H*), 2.90 (s, 6H) (NCH_3). $^{13}C\{^1H\}$ NMR (125 MHz, CD_3CN): *δ* 151.1, 139.7, 127.8, 113.2 (aryl and alkenyl *C*), 55.5, 50.4 (cage *C*H), 40.6 (N*C*H_3). Bcage-*C* was not observed. $^{11}B\{^1H\}$ NMR (160 MHz, CD_3CN): *δ* 6.3 (1B) (*B*-C), -2.8 (1B), -9.0 (2B), -13.5 – -14.9 (6B). HRMS (APCI): *m/z* calcd for $C_{12}H_{23}O^{10}B_2{}^{11}B_8N$ $[M+H]^+$: 290.2906. Found: 290.2904.

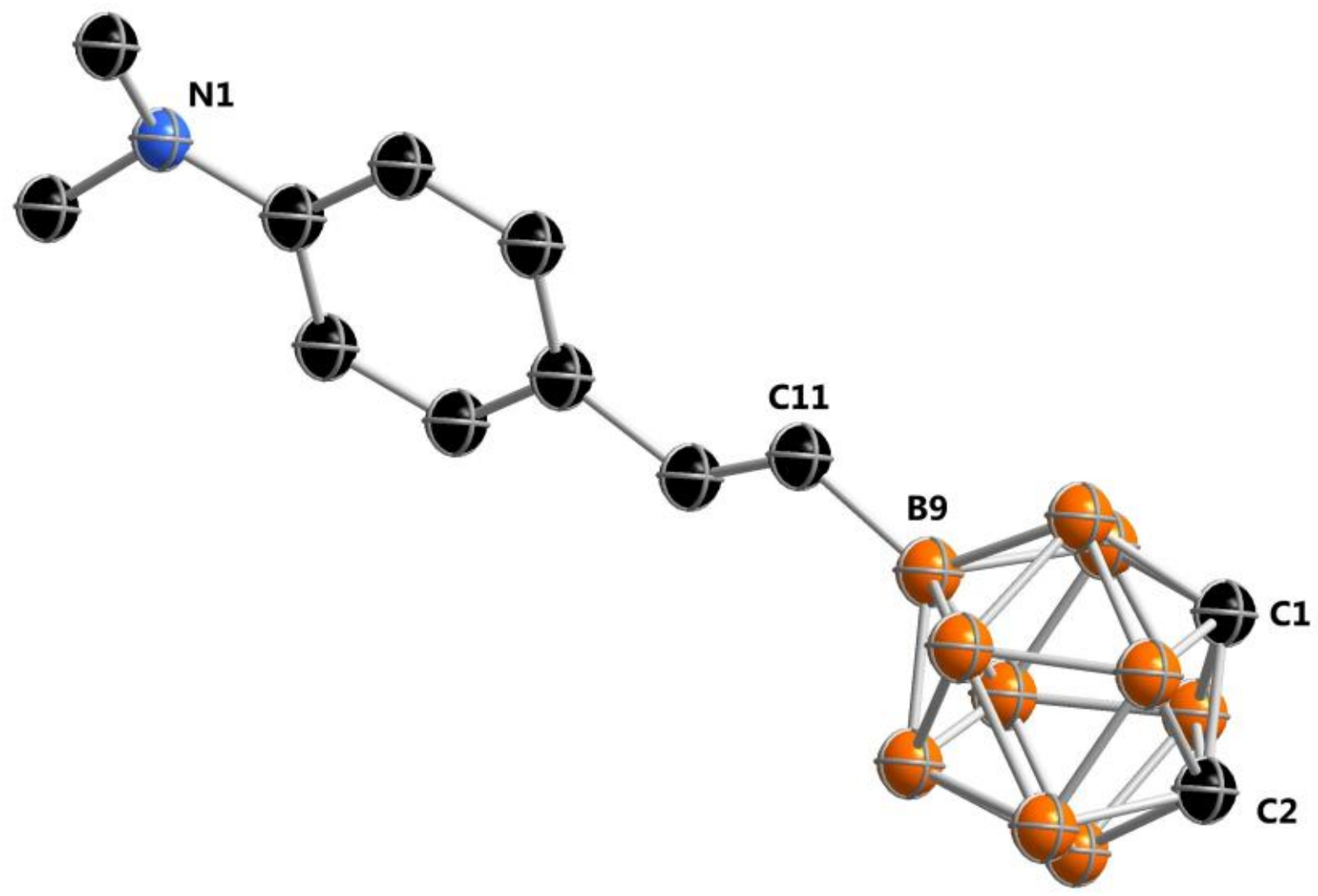

Fig. S2. Molecular Structure of **3ah**.

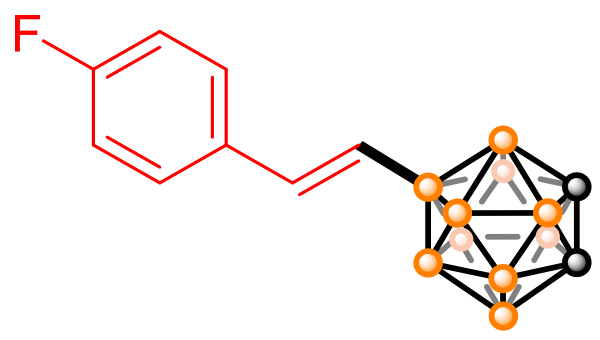

3ai: R_f (hexane/EA = 5:1): 0.60. 22.7 mg, 86% yield. White solid. Mp: 122−123 °C. ^{1}H NMR (500 MHz, CD_3CN): δ 7.38 (t, J = 6.9 Hz, 2H), 7.03 (t, J = 8.7 Hz, 2H). (aryl C*H*), 6.56 (d, J = 18.0 Hz, 1H), 6.26 (d, J = 18.0 Hz, 1H) (alkenyl C*H*), 4.06 (s, 1H), 3.99 (s, 1H) (Cage C*H*). ^{13}C{^{1}H} NMR (125 MHz, CD_3CN): δ 163.0 (d, $^1J_{CF}$ = 244.0 Hz), 138.4, 136.1, 128.6 (d, $^3J_{CF}$ = 8.1 Hz), 116.1 (d, $^2J_{CF}$ = 21.7 Hz) (aryl and alkenyl *C*), 55.7, 51.0 (cage *C*H). Bcage-*C* was not observed. ^{11}B{^{1}H} NMR (160 MHz, CD_3CN): δ 5.9 (1B) (*B*-C), -2.4 (1B), -8.9 (2B), -13.7 − -15.3 (6B). ^{19}F NMR (376 MHz, CD_3CN): δ -115.3 (s, 1F). HRMS (APCI): *m/z* calcd for $C_{10}H_{17}{}^{10}B_2{}^{11}B_8F$ [M-H]$^-$: 263.2250. Found: 263.2245.

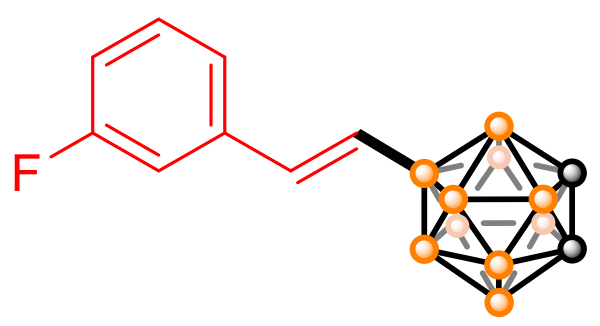

3aj: R_f (hexane/EA = 5:1): 0.60. 21.1 mg, 80% yield. White solid. Mp: 125−126 °C. ^{1}H NMR (500 MHz, CD_3CN): δ 7.32−7.29 (m, 1H), 7.19−7.12 (m, 2H), 6.96−6.92 (m, 2H) (aryl

C*H*), 6.56 (d, J = 18.0 Hz, 1H), 6.55 (s, 1H), 6.38 (d, J = 18.0 Hz, 2H) (alkenyl C*H*), 4.08 (s, 1H), 4.00 (s, 1H) (Cage C*H*). $^{13}C\{^1H\}$ NMR (125 MHz, CD_3CN): δ 164.0 (d, $^1J_{CF}$ = 242.7 Hz), 142.2, 138.4 (d, $^4J_{CF}$ = 2.7 Hz), 131.2 (d, $^3J_{CF}$ = 8.6 Hz), 123.1 (d, $^4J_{CF}$ = 2.7 Hz), 114.7 (d, $^2J_{CF}$ = 21.5 Hz), 113.0 (d, $^2J_{CF}$ = 21.7 Hz) (aryl and alkenyl *C*), 55.8, 51.3 (cage *C*H). $^{11}B\{^1H\}$ NMR (160 MHz, CD_3CN): δ 5.7 (1B) (*B*-C), -2.7 (1B), -9.0 (2B), -13.5 – -14.8 (6B). ^{19}F NMR (376 MHz, CD_3CN): δ -114.0 (s, 1F). HRMS (APCI): *m/z* calcd for $C_{10}H_{17}{}^{10}B_2{}^{11}B_8F$ $[M-H]^-$: 263.2250. Found: 263.2245.

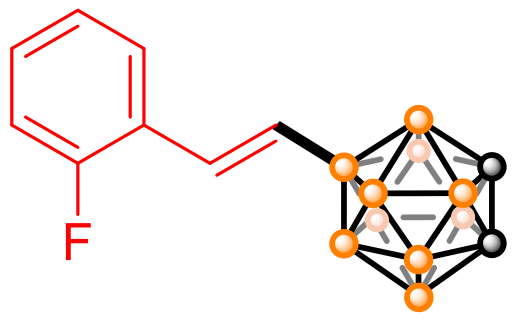

3ak: R_f (hexane/EA = 5:1): 0.61. 18.7 mg, 71% yield. White solid. Mp: 123−125 °C. ^1H NMR (500 MHz, CD_3CN): δ 7.53−7.49 (m, 1H), 7.23−7.20 (m, 1H), 7.12−7.09 (m, 1H), 7.07−7.02 (m, 1H) (aryl C*H*), 6.73 (d, J = 18.1 Hz, 1H), 6.41 (d, J = 18.2 Hz, 1H) (alkenyl C*H*), 4.09 (s, 1H), 4.01 (s, 1H) (Cage C*H*). $^{13}C\{^1H\}$ NMR (125 MHz, CD_3CN): δ 160.8 (d, $^1J_{CF}$ = 246.6 Hz), 115.0 (d, $^4J_{CF}$ = 4.5 Hz), 129.7 (d, $^3J_{CF}$ = 8.5 Hz), 127.6 (d, $^4J_{CF}$ = 3.8 Hz), 125.3 (d, $^4J_{CF}$ = 3.4 Hz), 116.4 (d, $^2J_{CF}$ = 22.4 Hz) (aryl and alkenyl *C*), 55.8, 51.3 (cage *C*H). Bcage-*C* was not observed. $^{11}B\{^1H\}$ NMR (160 MHz, CD_3CN): δ 5.5 (1B) (*B*-C), -2.7 (1B), -9.0 (2B), -13.4 – -14.8 (6B). ^{19}F NMR (376 MHz, CD_3CN): δ -113.4 (s, 1F). HRMS (APCI): *m/z* calcd for $C_{10}H_{17}{}^{10}B_2{}^{11}B_8F$ $[M+H]^+$: 265.2395. Found: 265.2394.

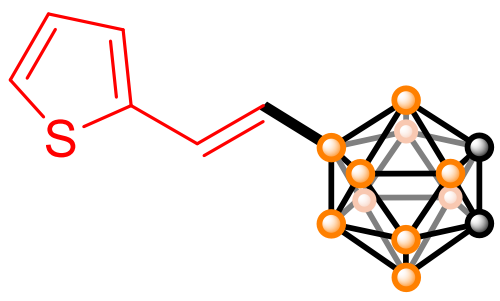

3al: R_f (hexane/EA = 5:1): 0.72. 11.6 mg, 46% yield. Colorless oil. ^1H NMR (400 MHz, $CDCl_3$): δ 7.09 (d, J = 4.8 Hz, 1H), 6.89 (dd, J = 13.7, 7.2 Hz, 3H), 6.69 (d, J = 17.4 Hz, 1H) (aryl and alkenyl C*H*), 3.56 (s, 1H), 3.46 (s, 1H) (Cage C*H*). $^{13}C\{^1H\}$ NMR (101 MHz, $CDCl_3$): δ 132.0, 127.3, 125.0, 123.9 (aryl and alkenyl *C*), 53.1, 48.7 (cage *C*H). Bcage-*C* was not observed. $^{11}B\{^1H\}$ NMR (128 MHz, $CDCl_3$): δ 4.9 (1B) (*B*-C), -3.1 (1B), -9.8 (2B),

-14.8, -15.3, -16.4 (6B). HRMS (APCI): *m/z* calcd for $C_8H_{17}{}^{10}B_2{}^{11}B_8S$ $[M-H]^-$: 252.1986. Found: 252.1992.

Preparation of B(9)- vinyl-*o*-Carboranes (3an. 1a (0.10 mmol), ethylene (2 atm), $Ni(PPh_3)_2I_2$ (0.1 equiv, 8.4 mg, 0.01 mmol), $P(4\text{-}F\text{-}C_6H_4)_3$ (0.4 equiv, 12.8 mg, 0.04 mmol), Zn (1 equiv, 6.4 mg, 0.1 mmol) and Cs_2CO_3 (2 equiv, 65.2 mg, 0.2 mmol) were mixed in MeCN (1 mL). The resulting mixture was stirred with 30W blue LED for 48 h under argon. After reaction, the reaction mixture was filtered through a suction funnel and washed with diethyl ether. The filtrate was concentrated to dryness in vacuo, which was subjected to flash column chromatography on silica gel (230-400 mesh) using *n*-hexane as eluent to give the products **3an**

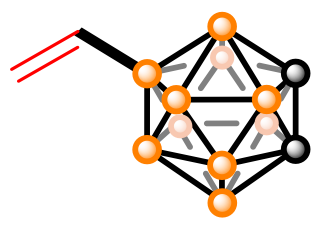

3an: R_f (hexane/EA = 5:1): 0.70. 13.5 mg, 80% yield. White solid. Mp: 111−112 °C. ^1H NMR (500 MHz, CD_3CN): δ 5.91 (d, J = 16.3 Hz, 1H), 5.29 (s, 2H) (alkenyl C*H*), 4.02 (s, 1H), 3.96 (s, 1H) (Cage C*H*). $^{13}C\{^1H\}$ NMR (125 MHz, CD_3CN): δ 140.1, 139.5, 138.9, 138.3 (d, B-*C*), 125.7 (alkenyl *C*), 55.8, 51.2 (cage *C*H). $^{11}B\{^1H\}$ NMR (160 MHz, CD_3CN): δ 5.1 (1B) (*B*-C), -3.18 (1B), -9.5 (2B), -14.0 – -15.3 (6B). HRMS (APCI): *m/z* calcd for $C_4H_{14}{}^{10}B_2{}^{11}B_8$ $[M-H]^-$: 169.2026. Found: 169.2025.

Preparation of B(4)-Alkenyl-*o*-Carboranes (3b). A Representative Procedure. 1b (0.10 mmol), alkene **2** (3 equiv, 0.3 mmol), $Ni(PPh_3)_2I_2$ (0.1 equiv, 8.4 mg, 0.01 mmol), $P(4\text{-}F\text{-}C_6H_4)_3$ (0.4 equiv, 12.8 mg, 0.04 mmol), Zn (1 equiv, 6.4 mg, 0.1 mmol) and Cs_2CO_3 (2 equiv, 65.2 mg, 0.2 mmol) were mixed in MeCN (1 mL). The resulting

mixture was stirred with 30W blue LED for 48 h under argon. After reaction, the reaction mixture was filtered through a suction funnel and washed with diethyl ether. The filtrate was concentrated to dryness in vacuo, which was subjected to flash column chromatography on silica gel (230-400 mesh) using *n*-hexane and ethyl acetate (500/1 in V/V) as eluent to give the products **3b**.

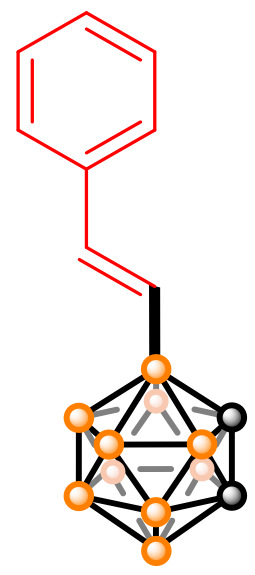

3ba: R_f (hexane/EA = 5:1): 0.69. 20.5 mg, 84% yield. White solid. Mp: 127−129 °C. ^{1}H NMR (500 MHz, CD_3CN): δ 7.45 (d, J = 7.6 Hz, 2H), 7.34 (t, J = 7.5 Hz, 2H), 7.27 (t, J = 7.3 Hz, 1H) (aryl C*H*), 6.91 (d, J = 18.1 Hz, 1H), 6.41 (d, J = 18.1 Hz, 1H) (alkenyl C*H*), 4.02 (s, 1H), 3.96 (s, 1H) (Cage C*H*). ^{13}C{^{1}H} NMR (125 MHz, CD_3CN): δ 143.5, 139.0, 129.6, 129.0, 127.1 (aryl and alkenyl *C*), 55.3, 57.2 (cage *C*H). ^{11}B{^{1}H} NMR (160 MHz, CD_3CN): δ -2.4 (1B), -3.3 (1B), -4.3 (1B) (*B*-C), -8.9 (1B), -9.9 (1B), -12.3 (1B), -13.8 − -14.4 (3B), -16.5 (1B). HRMS (APCI): *m/z* calcd for $C_{10}H_{18}{}^{10}B_2{}^{11}B_8$ $[M+H]^+$: 245.2344. Found: 245.2338.

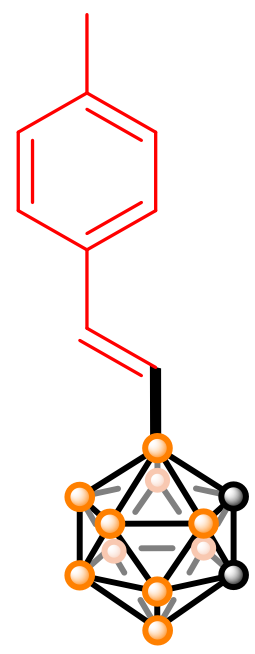

3bb: R_f (hexane/EA = 5:1): 0.71. 22.1 mg, 85%. White solid. Mp: 135−137 °C. ^{1}H NMR (500 MHz, CD_3CN): δ 7.33 (d, J = 7.1 Hz, 2H), 7.15 (d, J = 7.4 Hz, 2H) (aryl C*H*), 6.86 (d, J = 18.1 Hz, 1H), 6.34 (d, J = 18.2 Hz, 1H) (alkenyl C*H*), 4.15 (s, 2H) (Cage C*H*), 2.31 (s, 3H) (C*H₃*). ^{13}C{^{1}H} NMR (125 MHz, CD_3CN): δ 143.4, 138.9, 136.2, 130.2, 127.1 (aryl and alkenyl *C*), 57.3, 57.1 (cage *C*H), 21.2 (*C*H_3). ^{11}B{^{1}H} NMR (160 MHz, CD_3CN): δ -2.4 (1B), -3.3 (1B), -4.3 (1B) (*B*-C), -8.9 (1B), -9.9 (1B), -12.4 (1B), -13.8 − -14.3 (3B), -16.6 (1B). HRMS (APCI): *m/z* calcd for $C_{11}H_{20}{}^{10}B_2{}^{11}B_8$ $[M-H]^-$: 259.2501. Found:

259.2496.

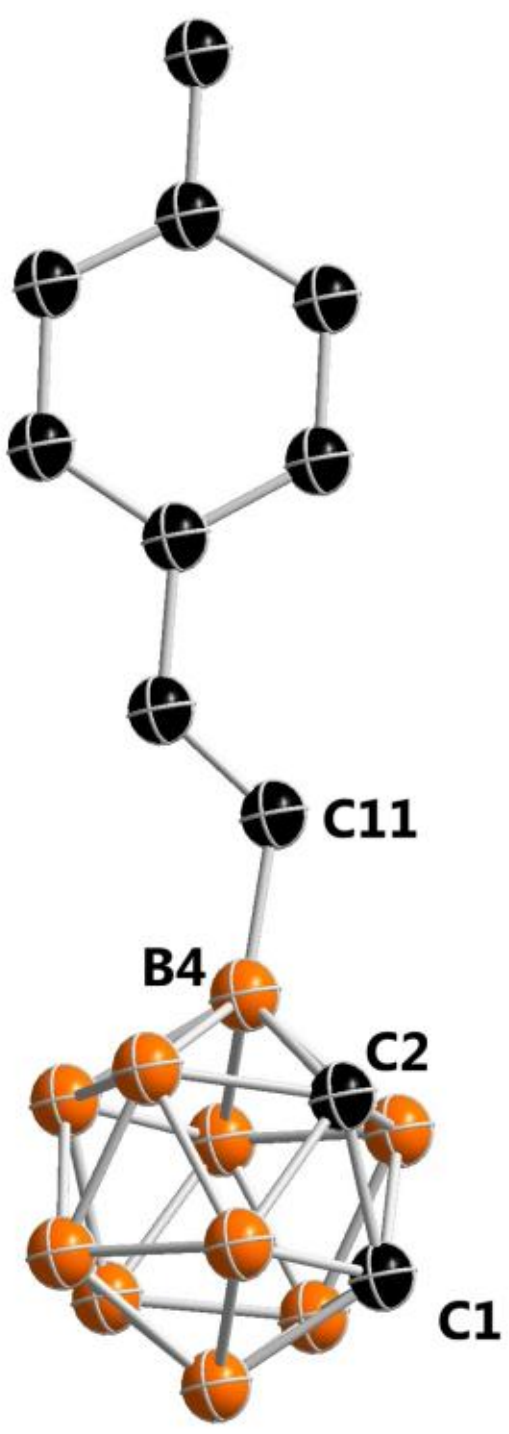

Fig. S3. Molecular Structure of **3bb**.

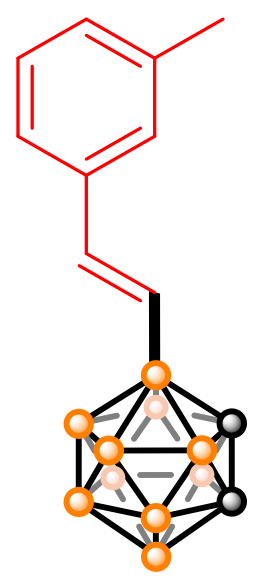

3bc: R_f (hexane/EA = 5:1): 0.71. 20.8 mg, 80%. White solid. Mp: 135−137 °C. ^{1}H NMR (500 MHz, CD_3CN): *δ* 7.46−7.44 (m, 1H), 7.36−7.32 (m, 2H) (aryl C*H*), 6.86 (d, *J* = 18.1 Hz, 1H), 6.34 (d, *J* = 18.2 Hz, 1H), 7.28 (d, *J* = 7.3 Hz, 1H). (alkenyl C*H*), 4.19, 4.16 (s, 2H) (Cage C*H*), 2.23 (s, 3H) (C*H₃*). ^{13}C{^{1}H} NMR (125 MHz, CD_3CN): *δ* 143.4, 138.9, 129.6, 128.9, 127.1 (aryl and alkenyl *C*), 57.3, 57.2 (cage *C*H), 35.6 (*C*H_3). ^{11}B{^{1}H} NMR (160 MHz, CD_3CN): *δ* -2.4 (1B), -3.3 (1B), -4.4 (1B) (*B*-C), -9.0 (1B), -9.9 (1B), -12.4 (1B), -13.7 – -14.3 (3B), -16.5 (1B). HRMS (APCI): *m/z* calcd for $C_{11}H_{20}{}^{10}B_2{}^{11}B_8$ [M-H]$^-$: 259.2501. Found: 259.2496.

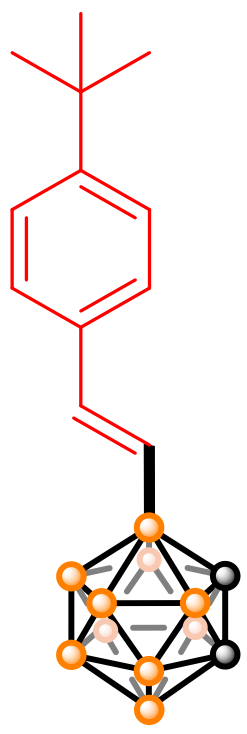

3be: R_f (hexane/EA = 5:1): 0.72. 24.5 mg, 81% yield. White solid. Mp: 152−153 °C. ^{1}H NMR (500 MHz, CD_3CN): δ 7.38 (m, 4H) (aryl C*H*), 6 6.87 (d, *J* = 18.1 Hz, 1H), 6.35 (d, *J* = 18.1 Hz, 1H) (alkenyl C*H*), 4.17 (s, 1H), 4.15 (s, 1H) (Cage C*H*), 1.30 (s, 9H) (C-CH_3). ^{13}C{^{1}H} NMR (125 MHz, CD_3CN): δ 152.1, 143.3, 136.3, 126.9, 126.5 (aryl and alkenyl *C*), 57.3, 57.2 (cage *C*H), 35.2 (*C*-CH_3), 31.4 (C-*C*H_3). ^{11}B{^{1}H} NMR (160 MHz, CD_3CN): δ -2.6 (1B), -3.5 (1B), -4.4 (1B) (*B*-C), -9.2 (1B), -10.1 (1B), -12.6 (1B), -14.0 − -14.6 (3B), -16.8 (1B). HRMS (APCI): *m/z* calcd for $C_{14}H_{26}{}^{10}B_2{}^{11}B_8$ $[M+H]^+$: 303.3117. Found: 303.3115.

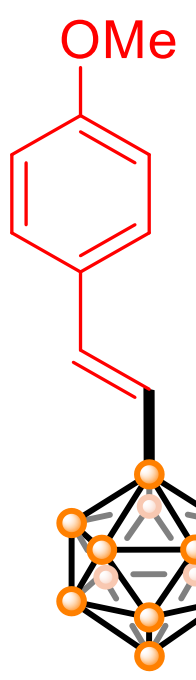

3bf: R_f (hexane/EA = 5:1): 0.70. 22.9 mg, 83% yield. White solid. Mp: 145−147 °C. ^{1}H NMR (500 MHz, CD_3CN): δ 7.38 (d, *J* = 8.8 Hz, 2H), 6.89 (d, *J* = 8.7 Hz, 2H) (aryl C*H*), 6.84 (d, *J* = 18.1 Hz, 1H), 6.23 (d, *J* = 18.0 Hz, 1H) (alkenyl C*H*), 4.15 (s, 2H) (Cage C*H*), 3.78 (s, 3H) (OCH_3). ^{13}C{^{1}H} NMR (125 MHz, CD_3CN): δ 160.6, 143.0, 131.8, 129.7, 128.4, 114.9 (aryl and alkenyl *C*), 57.3, 57.1 (cage *C*H), 55.9 (O*C*H_3). ^{11}B{^{1}H} NMR (160 MHz, CD_3CN): δ -2.4 (1B), -3.3 (1B), -4.1 (1B) (*B*-C), -9.0 (1B), -10.0 (1B), -12.4 (1B), -13.8 − -14.4 (3B), -16.7 (1B). HRMS (APCI): *m/z* calcd for $C_{11}H_{20}O^{10}B_2{}^{11}B_8$ $[M+H]^+$: 277.2596. Found: 277.2589.

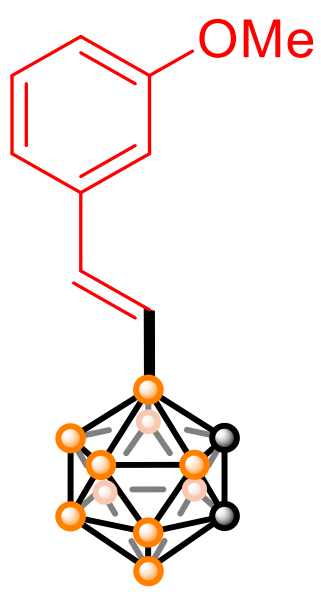

3bg: R_f (hexane/EA = 5:1): 0.71. 20.4 mg, 74% yield. White solid. Mp: 141−142 °C. ^{1}H NMR (500 MHz, CD_3CN): δ 7.27−7.23 (m, 1H), 7.04−7.01 (m, 2H), 6.91-6.84 (m, 2H), 6.43 (d, *J* = 20.2 Hz, 1H) (aryl and alkenyl C*H*), 4.18 (s, 1H), 4.16 (s, 1H) (Cage C*H*), 3.79 (s, 3H) (OCH_3). ^{13}C{^{1}H} NMR (125 MHz, CD_3CN): δ 161.0, 145.5, 131.2, 128.7, 115.0 (aryl and alkenyl *C*), 58.7 (cage *C*H), 55.9 (O*C*H_3). ^{11}B{^{1}H} NMR (160 MHz, CD_3CN):

δ -2.4 (1B), -3.3 (1B), -4.4 (1B) (*B*-C), -8.9 (1B), -9.9 (1B), -12.4 (1B), -13.8 – -14.3 (3B), -16.5 (1B). HRMS (APCI): *m/z* calcd for $C_{11}H_{20}O^{10}B_2{}^{11}B_8$ $[M+H]^+$: 277.2596. Found: 277.2588.

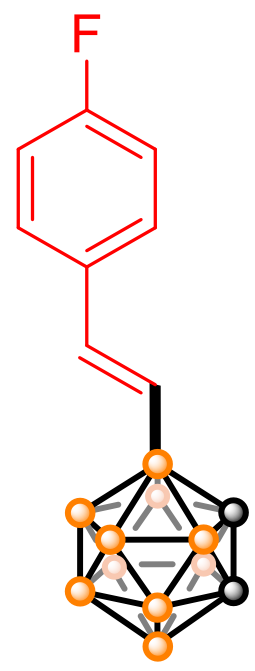

3bi: R_f (hexane/EA = 5:1): 0.73. 21.1 mg, 80% yield. White solid. Mp: 122−123 °C. ^1H NMR (500 MHz, CD_3CN): δ 7.48−7.45 (m, 2H), 7.10−7.06 (m, 2H) (aryl C*H*), 6.88 (d, J = 18.0 Hz, 1H), 6.34 (d, J = 18.1 Hz, 1H) (alkenyl C*H*), 4.18 (s, 1H), 4.17 (s, 1H) (Cage C*H*). $^{13}C\{^1H\}$ NMR (125 MHz, CD_3CN): δ 163.3 (d, $^1J_{CF}$ = 244.8 Hz), 142.1, 135.5, 128.9 (d, $^3J_{CF}$ = 8.2 Hz), 116.2 (d, $^2J_{CF}$ = 21.8 Hz) (aryl and alkenyl *C*), 57.3, 57.2 (cage *C*H). $^{11}B\{^1H\}$ NMR (160 MHz, CD_3CN): δ -2.4 (1B), -3.3 (1B), -4.4 (1B) (*B*-C), -9.0 (1B), -9.9 (1B), -12.4 (1B), -13.8 – -14.3 (3B), -16.5 (1B). ^{19}F NMR (376 MHz, CD_3CN): δ -115.7 (s, 1F). HRMS (APCI): *m/z* calcd for $C_{10}H_{17}F^{10}B_2{}^{11}B_8$ $[M-H]^-$: 263.2250. Found: 263.2247.

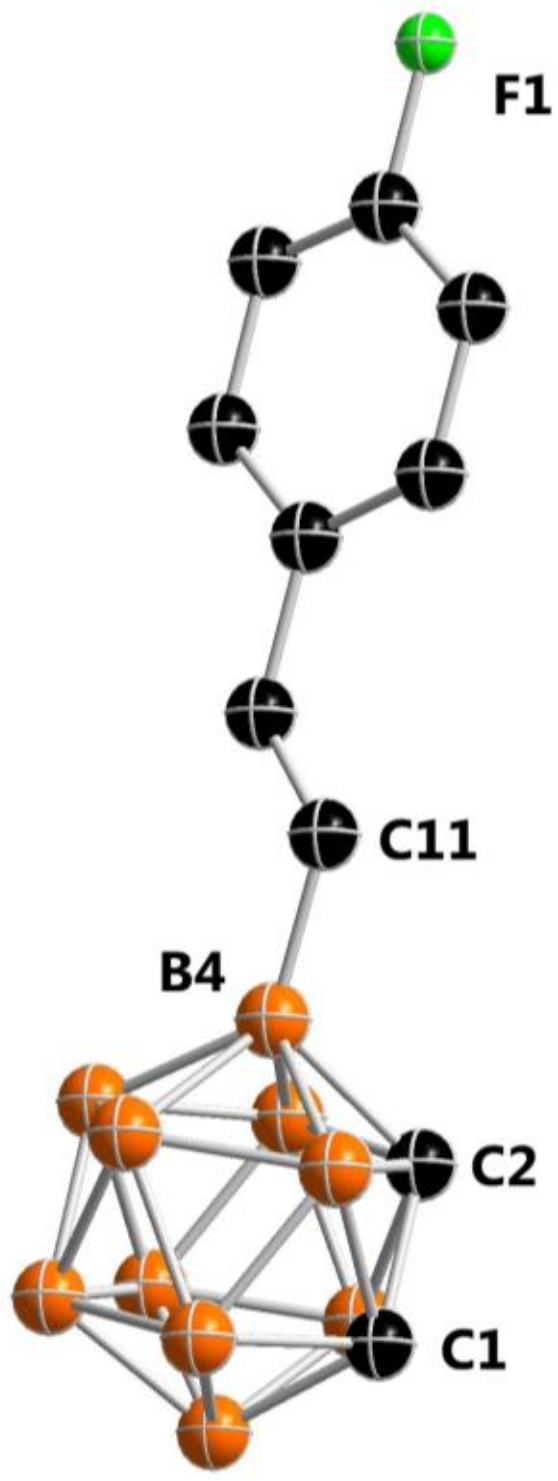

Fig. S4. Molecular Structure of **3bi**.

Preparation of B(3)-Alkenyl-*o*-Carboranes (3c). A Representative Procedure. **1c** (0.10 mmol), alkene **2** (3 equiv, 0.3 mmol), $Ni(PPh_3)_2I_2$ (0.1 equiv, 8.4 mg, 0.01 mmol), $P(4\text{-}F\text{-}C_6H_4)_3$ (0.4 equiv, 12.8 mg, 0.04 mmol), Zn (1 equiv, 6.4 mg, 0.1 mmol) and Cs_2CO_3 (2 equiv, 65.2 mg, 0.2 mmol) were mixed in MeCN (1 mL). The resulting mixture was stirred with 30W blue LED for 48 h under argon. After reaction, the reaction mixture was filtered through a suction funnel and washed with diethyl ether. The filtrate was concentrated to dryness in vacuo, which was subjected to flash column chromatography on silica gel (230-400 mesh) using *n*-hexane and ethyl acetate (500/1 in V/V) as eluent to give the products **3c**.

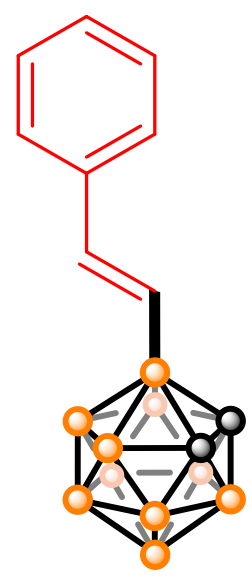

3ca: R_f (hexane/EA = 5:1): 0.73. 20.0 mg, 82% yield. White solid. Mp: 130−131 °C. ^1H NMR (500 MHz, CD_3CN): δ 7.49 (d, J = 7.4 Hz, 2H), 7.39−7.36 (m, 2H), 7.33−7.30 (m, 1H) (aryl C*H*), 7.13 (d, J = 18.2 Hz, 1H), 6.43 (d, J = 18.2 Hz, 1H) (alkenyl C*H*), 4.13 (s, 2H) (Cage C*H*). $^{13}C\{^1H\}$ NMR (125 MHz, CD_3CN): δ 146.0, 138.4, 129.7, 129.5, 127,3 (aryl and alkenyl *C*), 55.8 (cage *C*H). $^{11}B\{^1H\}$ NMR (160 MHz, CD_3CN): δ -3.2 (2B), -5.5 (1B) (*B*-C), -9.0 (1B), -12.6 – -13.4 (6B). HRMS (APCI): *m/z* calcd for $C_{10}H_{18}{}^{10}B_2{}^{11}B_8$ $[M\text{-}H]^-$: 245.2344. Found: 245.2338.

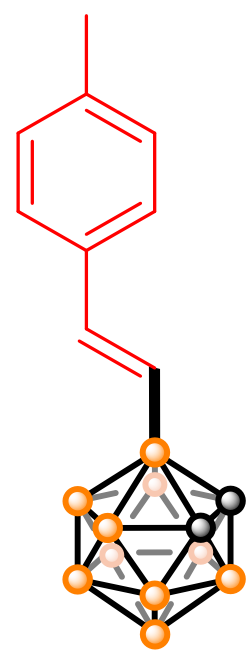

3cb: R_f (hexane/EA = 5:1): 0.75. 22.1 mg, 85% yield. White solid. Mp: 137−138 °C. ^1H NMR (500 MHz, CD_3CN): δ 7.38 (d, J = 8.1 Hz, 2H), 7.19 (d, J = 7.9 Hz, 2H) (aryl C*H*), 7.09 (d, J = 18.2 Hz, 1H), 6.36 (d, J = 18.1 Hz, 1H) (alkenyl C*H*), 4.11 (s, 2H) (Cage C*H*), 2.33 (s, 3H) (C*H₃*). $^{13}C\{^1H\}$ NMR (125 MHz, CD_3CN): δ 145.9, 139.7, 135.7, 130.3, 127.3 (aryl and alkenyl *C*), 58.4 (cage *C*H), 21.2 (*C*H_3). $^{11}B\{^1H\}$ NMR (160 MHz, CD_3CN): δ -3.7 (2B), -5.8 (1B) (*B*-C), -9.5 (1B), -13.1 – -13.9 (6B). HRMS (APCI): *m/z* calcd for

$C_{11}H_{20}{}^{10}B_2{}^{11}B_8$ [M-H]$^-$: 259.2501. Found: 259.2495.

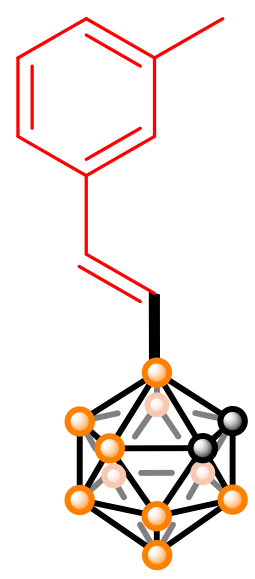

3cc: R_f (hexane/EA = 5:1): 0.73. 21.6 mg, 19.2% yield. White solid. Mp: 135−136 °C. ^{1}H NMR (500 MHz, CD_3CN): δ 7.34−7.28 (m, 3H), 7.17−7.10 (m, 2H) (aryl and alkenyl C*H*), 4.16 (s, 2H) (Cage C*H*), 2.36 (s, 3H) (C*H₃*). $^{13}C\{^1H\}$ NMR (125 MHz, CD_3CN): δ 146.0, 139.4, 130.2, 129.6, 127.9, 124.5 (aryl and alkenyl *C*), 58.7 (cage *C*H), 21.3 (*C*H₃). $^{11}B\{^1H\}$ NMR (160 MHz, CD_3CN): δ -3.3 (2B), -5.6 (1B) (*B*-C), -9.1 (1B), -12.6 – -13.5 (6B). HRMS (APCI): *m/z* calcd for $C_{11}H_{20}{}^{10}B_2{}^{11}B_8$ [M-H]$^-$: 259.2501. Found: 259.2495.

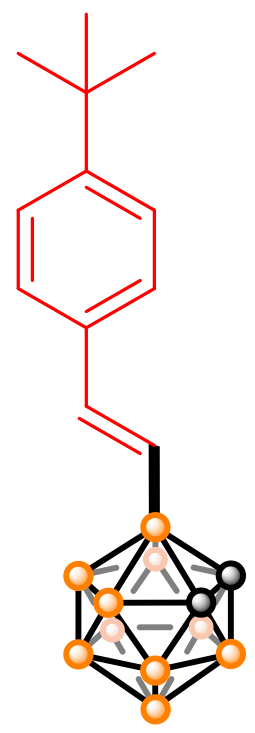

3ce: R_f (hexane/EA = 5:1): 0.76. 25.1 mg, 83% yield. White solid. Mp: 151−153 °C. ^{1}H NMR (500 MHz, CD_3CN): δ 7.42 (m, 4H) (aryl C*H*), 7.10 (d, *J* = 18.2 Hz, 1H), 6.37 (d, *J* = 18.2 Hz, 1H) (alkenyl C*H*), 4.11 (s, 2H) (Cage C*H*), 1.30 (s, 9H) (C-C*H₃*). $^{13}C\{^1H\}$ NMR (125 MHz, CD_3CN): δ 152.8, 145.8, 135.8, 127.1, 126.6, 135.7, 130.3, 127.3 (aryl and alkenyl *C*), 58.8 (cage *C*H), 35.3, (*C*-CH₃), 31.4 (C-*C*H₃). $^{11}B\{^1H\}$ NMR (160 MHz, CD_3CN): δ -3.2 (2B), -5.3 (1B) (*B*-C), -9.0 (1B), -12.6 – -13.5 (6B). HRMS (APCI): *m/z* calcd for $C_{14}H_{26}{}^{10}B_2{}^{11}B_8$ [M+H]$^+$: 303.3117. Found: 303.3115.

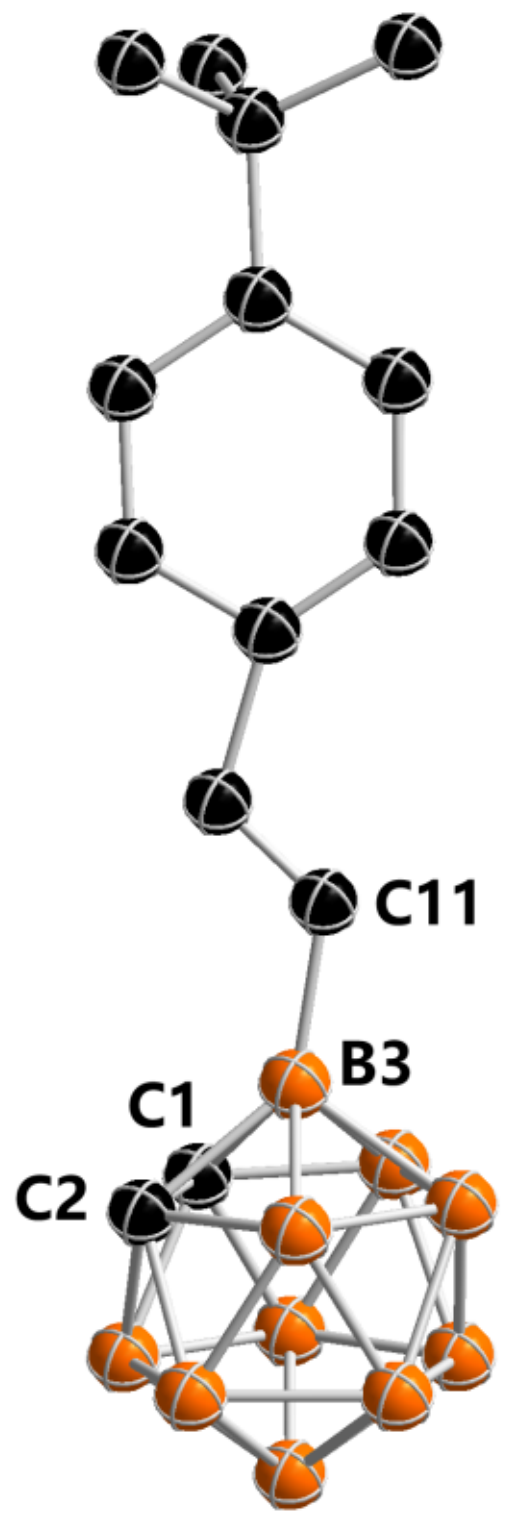

Fig. S5. Molecular Structure of **3ce**.

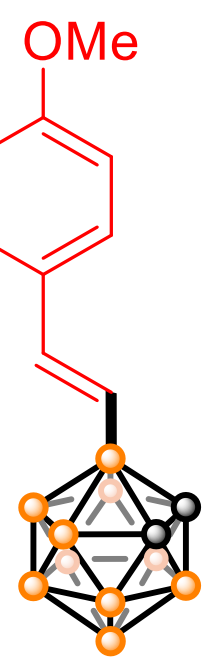

3cf: R_f (hexane/EA = 5:1): 0.71. 23.7 mg, 86% yield. White solid. Mp: 148–150 °C. ^{1}H NMR (500 MHz, CD_3CN): δ 7.43 (d, J = 8.8 Hz, 2H), 7.06 (d, J = 18.1 Hz, 1H), 6.92 (d, J = 8.8 Hz, 2H), 6.25 (d, J = 18.2 Hz, 1H).) (aryl and alkenyl C*H*), 4.10 (s, 2H) (Cage C*H*), 3.79 (s, 3H) (OC*H₃*). ^{13}C{^{1}H} NMR (125 MHz, CD_3CN): δ 161.0, 145.5, 131.2, 128.7, 115.0 (aryl and alkenyl *C*), 58.7 (cage *C*H), 55.9 (O*C*H₃). ^{11}B{^{1}H} NMR (160 MHz, CD_3CN): δ -3.0 (2B), -4.9 (1B) (*B*-C), -8.7 (1B), -12.3 – -13.1 (6B). HRMS (APCI): *m/z* calcd for $C_{11}H_{20}{}^{10}B_2{}^{11}B_8O$ [M+H]$^+$: 277.2596. Found: 277.2591.

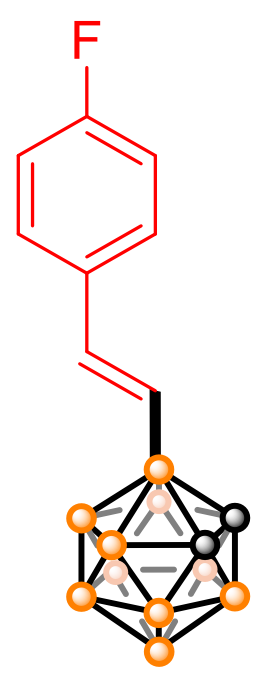

3ci: R_f (hexane/EA = 5:1): 0.70. 21.1 mg, 80% yield. White solid. Mp: 121−122 °C. ^{1}H NMR (500 MHz, CD_3CN): δ 7.53−7.50 (m, 2H), 7.13−7.10 (m, 3H) 6.36 (d, J = 18.2 Hz, 1H) (aryl and alkenyl C*H*), 4.13 (s, 2H). ^{13}C{^{1}H} NMR (125 MHz, CD_3CN): δ 163.71 (d, $^1J_{CF}$ = 246.0 Hz), 144.6, 135.0, 129.2 (d, $^3J_{CF}$ = 8.5 Hz), 116.4 (d, $^2J_{CF}$ = 21.9 Hz) (aryl and alkenyl *C*), 58.7 (cage *C*H). ^{11}B{^{1}H} NMR (160 MHz, CD_3CN): δ -2.9 (2B), -5.3 (1B) (*B*-C), -8.7 (1B), -12.3 − -13.1 (6B). ^{19}F NMR (376 MHz, CD_3CN): δ -115.3 (s, 1F). HRMS (APCI): *m/z* calcd for $C_{10}H_{17}{}^{10}B_2{}^{11}B_8F$ [M-H]$^-$: 263.2250. Found: 263.2245.

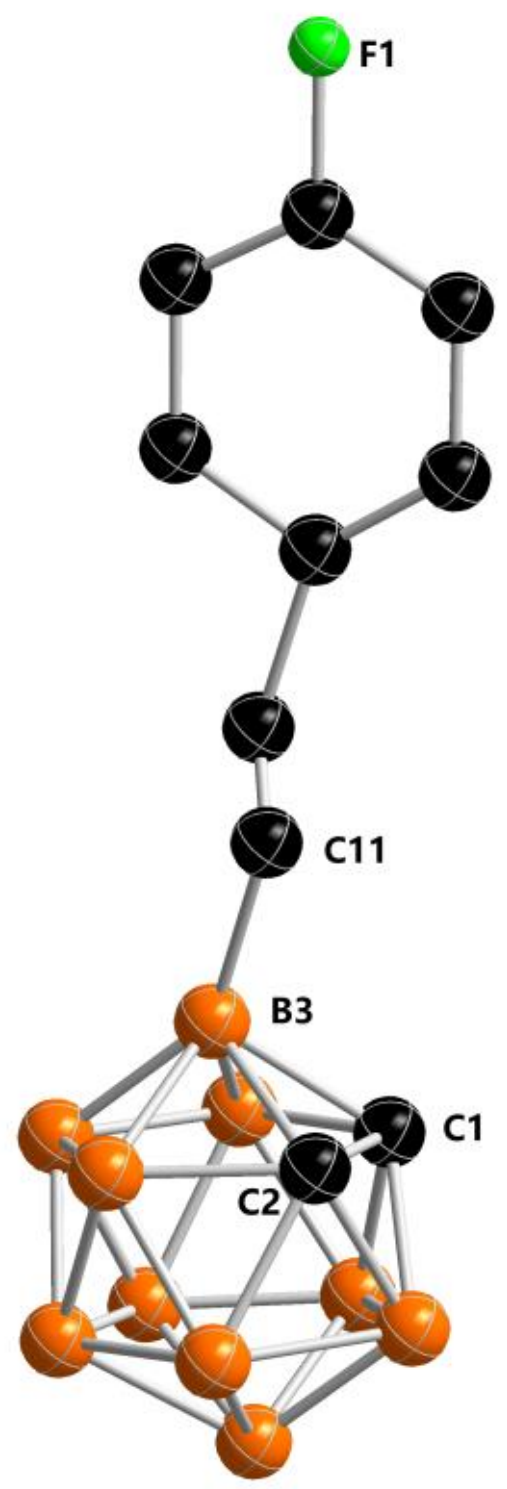

Fig. S6. Molecular Structure of **3ci**.

Preparation of 1,2-Dimethyl-B(9)-Alkenyl-*o*-Carboranes (3d). A Representative Procedure. **1d** (0.10 mmol), alkene **2** (3 equiv, 0.3 mmol), $Ni(PPh_3)_2I_2$ (0.1 equiv, 8.4 mg, 0.01 mmol), $P(4\text{-}F\text{-}C_6H_4)_3$ (0.4 equiv, 12.8 mg, 0.04 mmol), Zn (1

equiv, 6.4 mg, 0.1 mmol) and Cs_2CO_3 (2 equiv, 65.2 mg, 0.2 mmol) were mixed in MeCN (1 mL). The resulting mixture was stirred with 30W blue LED for 48 h under argon. After reaction, the reaction mixture was filtered through a suction funnel and washed with diethyl ether. The filtrate was concentrated to dryness in vacuo, which was subjected to flash column chromatography on silica gel (230-400 mesh) using *n*-hexane and ethyl acetate (500/1 in V/V) as eluent to give the products **3d**.

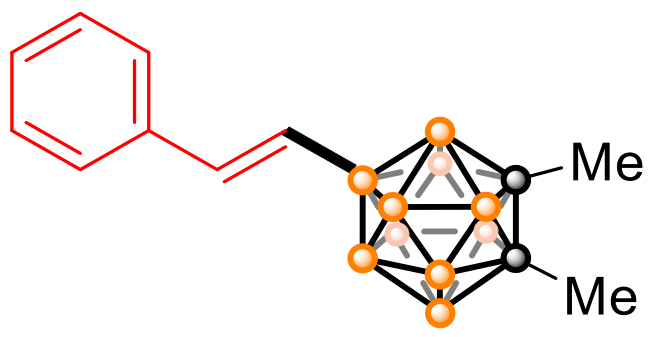

3da: R_f (hexane/EA = 5:1): 0.65. 22.0 mg, 80% yield. White solid. Mp: 145−147 °C. ^{1}H NMR (500 MHz, CD_3CN): δ 7.36 (d, J = 7.2 Hz, 2H), 7.28 (t, J = 7.6 Hz, 2H), 7.20 (t, J = 7.2 Hz, 1H) (aryl C*H*), 6.57 (d, J = 18.0 Hz, 1H), 6.33 (d, J = 17.9 Hz, 1H) (alkenyl C*H*), 2.06 (s, 6H) (CH_3). ^{13}C{^{1}H} NMR (125 MHz, CD_3CN): δ 139.8, 129.5, 128.2, 126.9 (aryl and alkenyl *C*), 74.2, 69.5 (cage *C*), 23.7, 22.8 (*C*H_3). Bcage-*C* was not observed. ^{11}B{^{1}H} NMR (160 MHz, CD_3CN): δ 3.1 (1B) (*B*-C), -5.4 (1B), -8.8 – -10.4 (8B). HRMS (APCI): *m/z* calcd for $C_{12}H_{22}{}^{10}B_2{}^{11}B_8$ $[M+H]^+$: 275.2803. Found: 275.2796.

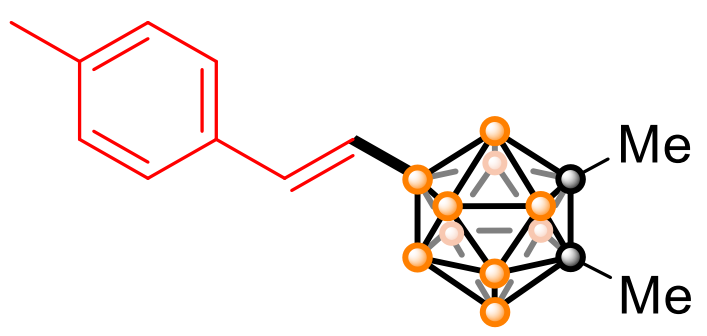

3db: R_f (hexane/EA = 5:1): 0.66. 24.2 mg, 84% yield. White solid. Mp: 152−153 °C. ^{1}H NMR (500 MHz, CD_3CN): δ 7.25 (d, J = 8.2 Hz, 2H), 7.10 (d, J = 8.0 Hz, 2H) (aryl C*H*), 6.53 (d, J = 18.0 Hz, 1H), 6.25 (d, J = 17.9 Hz, 1H) (alkenyl C*H*), 2.29 (s, 3H) (Ar CH_3), 2.06 (s, 6H) (cage CH_3). ^{13}C{^{1}H} NMR (125 MHz, CD_3CN): δ 139.7, 138.0, 137.0, 130.1, 126.9 (aryl and alkenyl *C*), 74.2, 69.4 (cage *C*), 23.7, 22.8 (cage *C*H_3), 21.2 (Ar *C*H_3). Bcage-*C* was not observed. ^{11}B{^{1}H} NMR (160 MHz, CD_3CN): δ 3.2 (1B) (*B*-C), -5.4 (1B), -8.8 – -10.4 (8B). HRMS (APCI): *m/z* calcd for $C_{13}H_{24}{}^{10}B_2{}^{11}B_8$ $[M+H]^+$: 289.2960. Found: 289.2953.

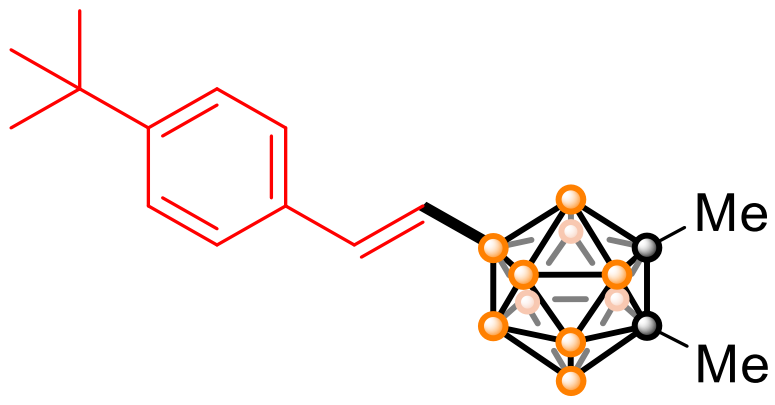

3de: R_f (hexane/EA = 5:1): 0.68. 26.7 mg, 81% yield. White solid. Mp: 168−170 °C. ^{1}H NMR (500 MHz, CD_3CN): δ 7.32 (d, J = 8.6 Hz, 2H), 7.28 (d, J = 8.6 Hz, 2H) (aryl C*H*), 6.54 (d, J = 18.0 Hz, 1H), 6.27 (d, J = 18.0 Hz, 1H) (alkenyl C*H*), 2.29 (s, 3H) (Ar CH_3), 2.05 (s, 6H) (cage CH_3), 1.28 (C-(CH_3)). $^{13}C\{^{1}H\}$ NMR (125 MHz, CD_3CN): δ 151.2, 139.6, 137.0, 126.7, 126.4 (aryl and alkenyl *C*), 74.2, 69.4 (cage *C*), 35.2 (*C*-CH_3), 31.6 (C-*C*H_3), 23.7, 22.8 (cage *C*H_3), 21.2 (Ar *C*H_3). Bcage-*C* was not observed. $^{11}B\{^{1}H\}$ NMR (160 MHz, CD_3CN): δ 3.2 (1B) (*B*-C), -5.4 (1B), -8.8 − -10.4 (8B). HRMS (APCI): *m/z* calcd for $C_{16}H_{30}{}^{10}B_2{}^{11}B_8$ $[M+H]^+$: 331.3426. Found: 331.3420.

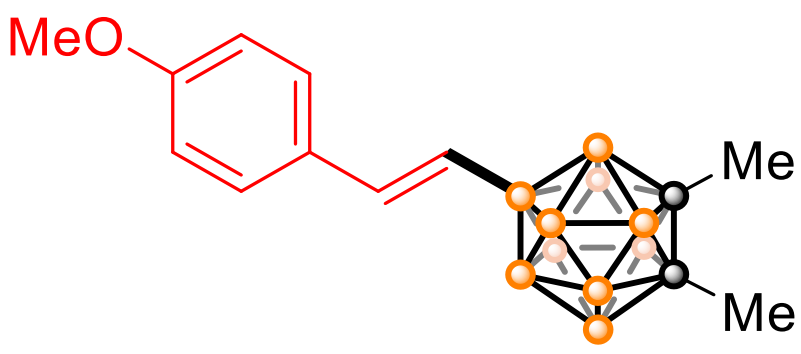

3df: R_f (hexane/EA = 5:1): 0.67. 24.9 mg 82% yield. White solid. Mp: 155−156 °C. ^{1}H NMR (500 MHz, CD_3CN): δ 7.30 (d, J = 8.3 Hz, 2H), 6.84 (d, J = 8.3 Hz, 2H) (aryl C*H*), 6.50 (d, J = 17.9 Hz, 1H), 6.14 (d, J = 18.0 Hz, 1H) (alkenyl C*H*), 3.76 (s, 3H) (OCH_3), 2.07 (s, 6H) (cage CH_3). $^{13}C\{^{1}H\}$ NMR (125 MHz, CD_3CN): δ 160.1, 139.2, 138.0, 132.5, 128.1, 114.8 (aryl and alkenyl *C*), 74.1, 69.2 (cage *C*), 55.8 (O*C*H_3), 23.7, 22.7 (cage *C*H_3). Bcage-*C* was not observed. $^{11}B\{^{1}H\}$ NMR (160 MHz, CD_3CN): δ 3.4 (1B) (*B*-C), -5.4 (1B), -8.7 − -10.3 (8B). HRMS (APCI): *m/z* calcd for $C_{13}H_{24}{}^{10}B_2{}^{11}B_8O$ $[M+H]^+$: 305.2910. Found: 305.2904.

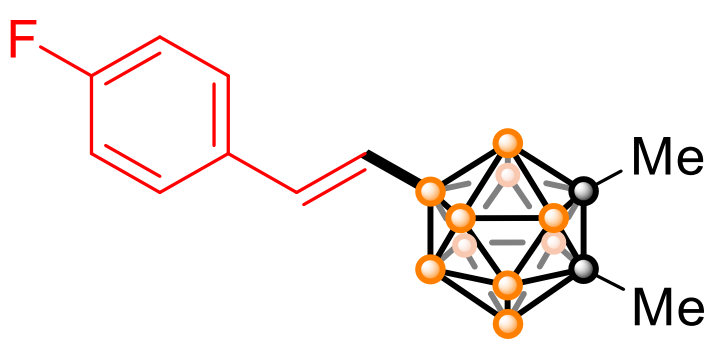

3di: R_f (hexane/EA = 5:1): 0.63. 23.4 mg, 80% yield. White solid. Mp: 139−140 °C. ^{1}H NMR (500 MHz, CD_3CN): δ 7.51−7.21 (m, 2H), 7.00 (t, J = 8.9 Hz, 2H) (aryl C*H*), 6.52 (d, J = 18.0 Hz, 1H), 6.22 (d, J = 18.0 Hz, 1H) (alkenyl C*H*), 2.05 (s, 6H) (CH_3). $^{13}C\{^{1}H\}$ NMR (125 MHz, CD_3CN): δ 163.0 (d, $^{1}J_{CF}$ = 244.1 Hz), 138.5,

136.2, 131.6 (d, $^3J_{CF}$ = 7.8 Hz), 116.1 (d, $^2J_{CF}$ = 21.7 Hz), 115.1 (d, $^2J_{CF}$ = 21.3 Hz) (aryl and alkenyl *C*), 74.2, 69.6 (cage *C*). Bcage-*C* was not observed. $^{11}B\{^1H\}$ NMR (160 MHz, CD_3CN): δ 3.0 (1B) (*B*-C), -5.4 (1B), -8.8 – -10.4 (8B). ^{19}F NMR (376 MHz, CD_3CN): δ 115.8 (s, 1F). HRMS (APCI): *m/z* calcd for $C_{12}H_{21}{}^{10}B_2{}^{11}B_8F$ $[M+H]^+$: 293.2709. Found: 293.2704.

Preparation of B(9,12)-Dilkenyled *o*-Carboranes (VI-3). A Representative Procedure. **1e** (0.10 mmol), alkene **2** (5 equiv, 0.5 mmol), $Ni(PPh_3)_2I_2$ (0.1 equiv, 8.4 mg, 0.01 mmol), $P(4\text{-}F\text{-}C_6H_4)_3$ (0.4 equiv, 12.8 mg, 0.04 mmol), Zn (1 equiv, 6.4 mg, 0.1 mmol) and Cs_2CO_3 (2 equiv, 65.2 mg, 0.2 mmol) were mixed in MeCN (0.8 mL). The resulting mixture was stirred with 30W blue LED for 48 h under argon. After reaction, the reaction mixture was filtered through a suction funnel and washed with diethyl ether. The filtrate was concentrated to dryness in vacuo, which was subjected to flash column chromatography on silica gel (230-400 mesh) using *n*-hexane and ethyl acetate (500/1 in V/V) as eluent to give the products **3e**.

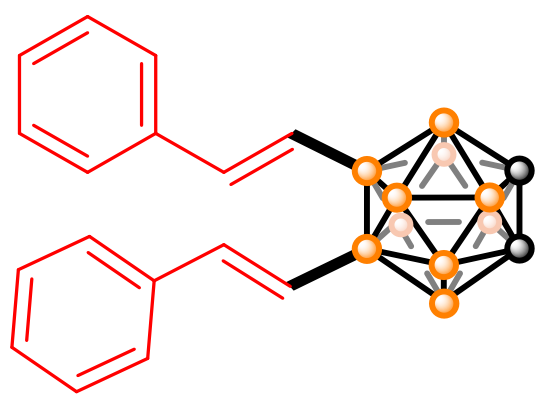

3ea: R_f (hexane/EA = 5:1): 0.55. 24.3 mg, 70% yield. White solid. Mp: 198−200 °C. ^1H NMR (500 MHz, CD_3CN): δ 7.36 (d, *J* = 7.3 Hz, 4H), 7.27 (t, *J* = 7.7 Hz, 4H), 7.19 (t, *J* = 7.3 Hz, 2H). (aryl C*H*), 6.63 (d, *J* = 18.0 Hz, 2H), 6.35 (d, *J* = 18.1 Hz, 2H (alkenyl C*H*), 4.03 (s, 2H) (Cage C*H*). $^{13}C\{^1H\}$ NMR (125 MHz, CD_3CN): δ 140.0, 139.6, 129.4, 128.2, 126.9 (aryl and alkenyl *C*), 50.6 (cage *C*H). Bcage-*C* was not observed. $^{11}B\{^1H\}$ NMR (160 MHz, CD_3CN): δ 5.6 (2B) (*B*-C), -8.9 (2B), -14.4 – -15.8 (6B). HRMS (APCI): *m/z* calcd for $C_{18}H_{24}{}^{10}B_2{}^{11}B_8$ $[M-H]^-$: 347.2806. Found: 347.2805.

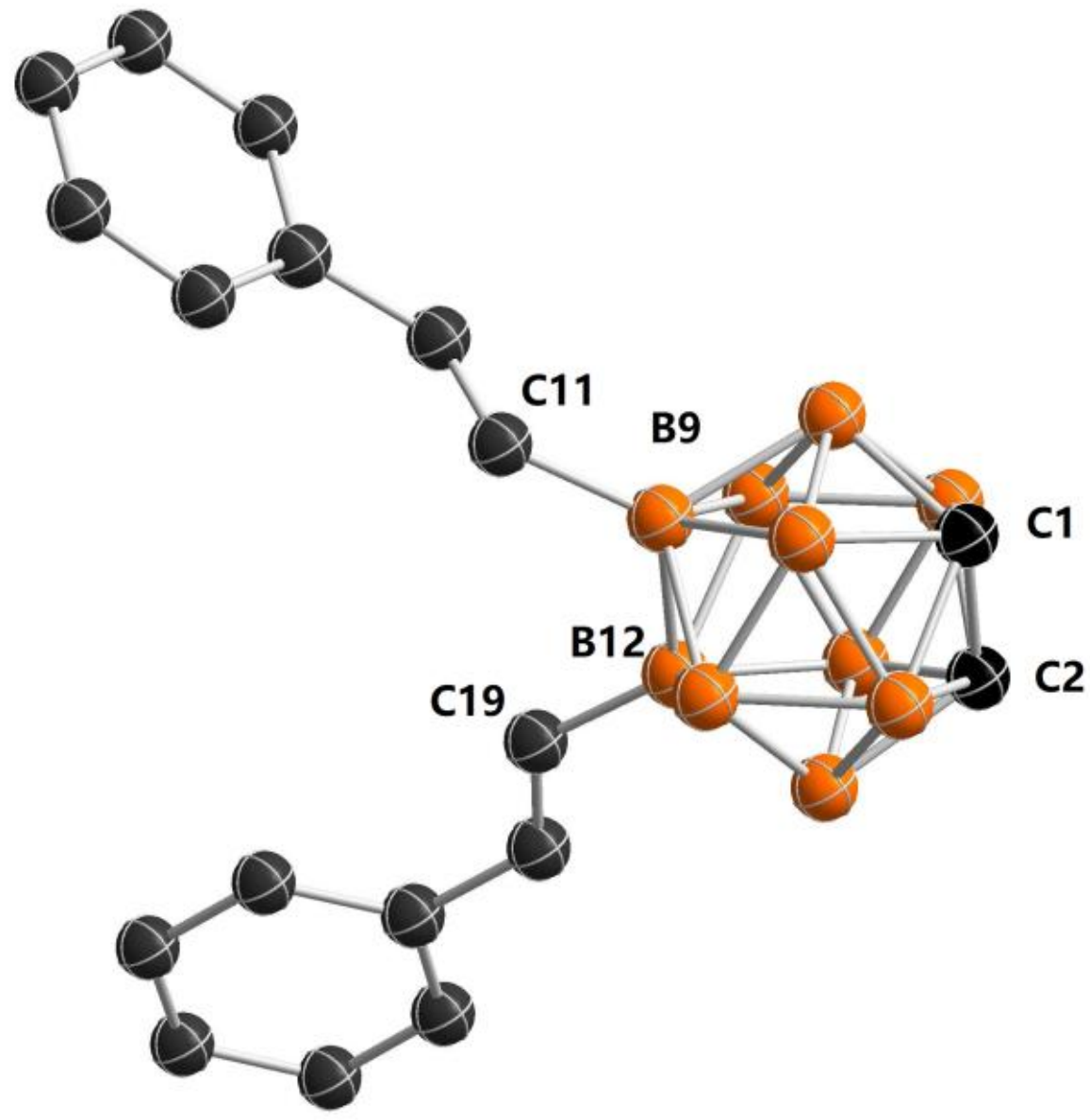

Fig. S7. Molecular Structure of **3ea**.

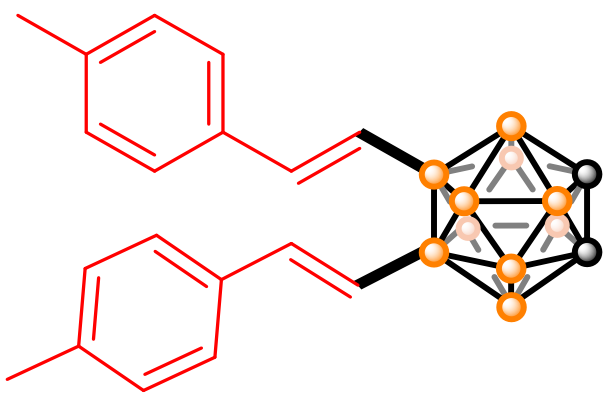

3eb: R_f (hexane/EA = 5:1): 0.59. 28.9 mg, 77% yield. White solid. Mp: 218−220 °C. ^{1}H NMR (500 MHz, CD_2Cl_2): δ 7.24 (d, J = 7.7 Hz, 4H), 7.07 (d, J = 7.7 Hz, 4H). (aryl C*H*), 6.57 (s, 2H), 6.29 (s, 2H) (alkenyl C*H*), 3.60 (s, 2H) (Cage C*H*), 2.29 (s, 6H) (CH_3). ^{13}C{^{1}H} NMR (125 MHz, CD_2Cl_2): δ 139.7, 129.5, 128.2, 126.9 (aryl and alkenyl *C*), 55.7, 51.0 (cage *C*H). Bcage-*C* was not observed. ^{11}B{^{1}H} NMR (160 MHz, CD_2Cl_2): δ 5.8 (2B) (*B*-C), -8.2 (2B), -12.9 − -15.5 (6B). HRMS (APCI): *m/z* calcd for $C_{20}H_{28}{}^{10}B_2{}^{11}B_8$ [M-H]$^-$: 375.3131. Found: 375.3119.

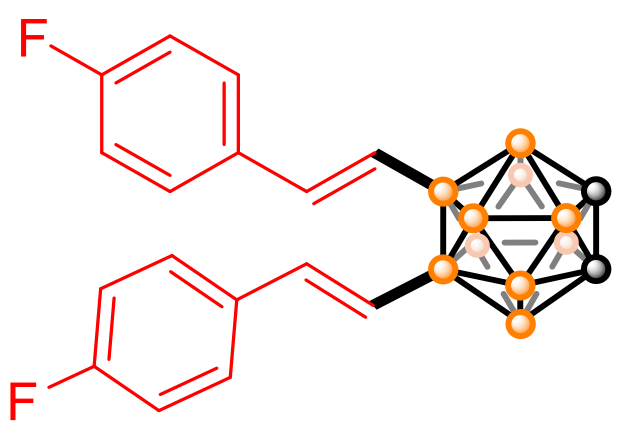

3ei: R_f (hexane/EA = 5:1): 0.51. 24.3 mg, 68% yield. White solid. Mp: 188−189 °C. ^{1}H NMR (500 MHz, CD_3CN): δ 7.59−7.27 (m, 4H), 7.01 (d, J = 8.8 Hz, 4H) (aryl C*H*), 6.60 (d, J = 18.1 Hz, 2H), 6.27 (d, J = 18.1 Hz, 2H) (alkenyl C*H*), 4.02 (s, 2H) (Cage C*H*).

$^{13}C\{^{1}H\}$ NMR (125 MHz, CD_3CN): δ 162.0 (d, $^{1}J_{CF}$ = 244.2 Hz), 137.8, 135.2 (d, $^{4}J_{CF}$ = 3.6 Hz), 127. (d, $^{3}J_{CF}$ = 7.9 Hz), 115.17 (d, $^{2}J_{CF}$ = 21.7 Hz) (aryl and alkenyl *C*), 49.8 (cage *C*H). Bcage-*C* was not observed. $^{11}B\{^{1}H\}$ NMR (160 MHz, CD_3CN): δ 7.1 (1B) (*B*-C), 5.5 (2B) (*B*-C), -2.1 (2B), -14.0 – -15.8 (6B). ^{19}F NMR (376 MHz, CD_3CN): δ -118.7 (s, 2F). HRMS (APCI): *m/z* calcd for $C_{18}H_{22}{}^{10}B_2{}^{11}B_8F_2$ $[M+H]^+$: 358.2774. Found: 358.2771.

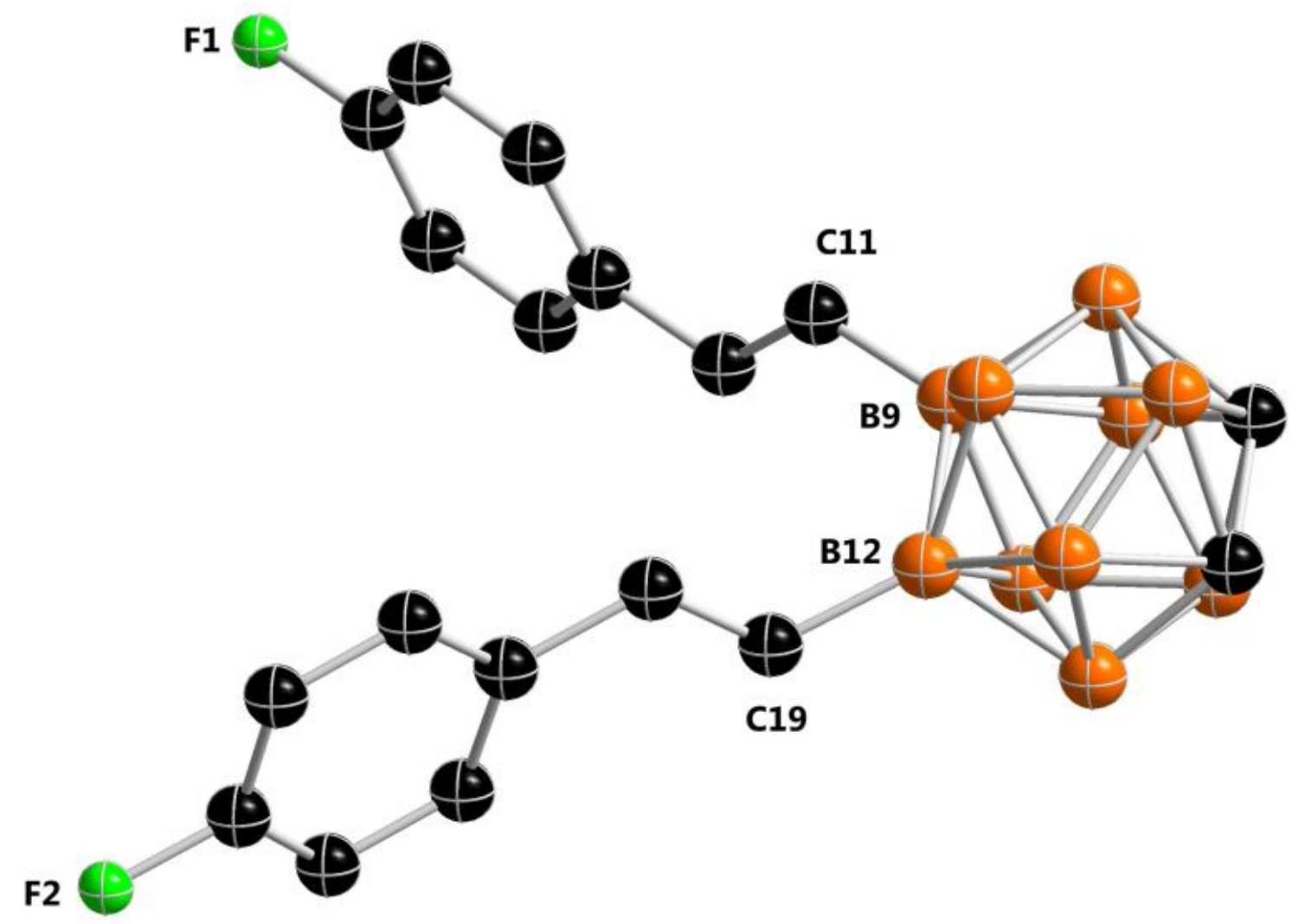

Fig. S8. Molecular Structure of **3ei**.

Preparation of B(9)-Alkenyl-*m*-Carboranes (3f). A Representative Procedure. 1a (0.10 mmol), alkene **2** (3 equiv, 0.3 mmol), $Ni(PPh_3)_2I_2$ (0.1 equiv, 8.4 mg, 0.01 mmol), $P(4\text{-}F\text{-}C_6H_4)_3$ (0.4 equiv, 12.8 mg, 0.04 mmol), Zn (1 equiv, 6.4 mg, 0.1 mmol) and Cs_2CO_3 (2 equiv, 65.2 mg, 0.2 mmol) were mixed in MeCN (1 mL). The resulting mixture was stirred with 30W blue LED for 48 h under argon. After reaction, the reaction mixture was filtered through a suction funnel and washed with diethyl ether. The filtrate was concentrated to dryness in vacuo, which was subjected to flash column

chromatography on silica gel (230-400 mesh) using *n*-hexane as eluent to give the products **3f**.

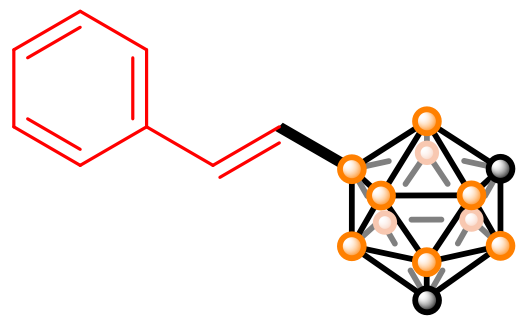

3fa: R_f (hexane/EA = 5:1): 0.51. 19.1 mg, 78% yield. White solid. Mp: 188−189 °C. ^{1}H NMR (400 MHz, CD_2Cl_2): δ 7.44−7.39 (m, 2H), 7.31 (t, *J* = 7.6 Hz, 2H), 7.24−7.19 (m, 1H) (aryl C*H*), 6.77 (d, *J* = 17.9 Hz, 1H), 6.44 (d, *J* = 18.0 Hz, 1H) (alkenyl C*H*), 3.04 (s, 2H) (Cage C*H*). ^{13}C{^{1}H} NMR (101 MHz, CD_2Cl_2): δ 140.6, 139.1, 128.8, 127.7, 126.5 (aryl and alkenyl *C*), 54.7 (cage *C*H). Bcage-*C* was not observed. ^{11}B{^{1}H} NMR (128 MHz, CD_2Cl_2): δ -1.3 (1B) (*B*-C), -6.5 (2B), -9.9 (1B) (*B*-C), -13.1 – -13.9 (4B), -17.4 (1B), -19.9 (1B). The data were the same as the reported ones.[4]

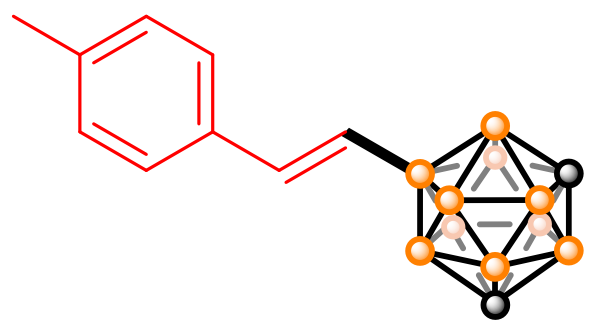

3fb: R_f (hexane/EA = 5:1): 0.58. 19.5 mg, 75% yield. White solid. ^{1}H NMR (400 MHz, CD_2Cl_2): δ 7.30 (d, *J* = 7.9 Hz, 2H), 7.12 (d, *J* = 7.9 Hz, 2H) (aryl C*H*), 6.73 (d, *J* = 18.0 Hz, 1H), 6.37 (d, *J* = 18.0 Hz, 1H) (alkenyl C*H*), 3.03 (s, 2H) (Cage C*H*), 2.33 (s, 3H) (CH_3). ^{13}C{^{1}H} NMR (101 MHz, CD_2Cl_2): δ 140.6, 137.6, 136.4, 129.5, 126.4 (aryl and alkenyl *C*), 54.7 (cage *C*H), 21.3 (*C*H_3). Bcage-*C* was not observed. ^{11}B{^{1}H} NMR (128 MHz, CD_2Cl_2): δ -1.2 (1B) (*B*-C), -6.6 (2B), -10.0 (1B) (*B*-C), -13.1 – -14.0 (4B), -17.4 (1B), -19.9 (1B). HRMS (APCI): *m/z* calcd for $C_{11}H_{20}{}^{10}B_2{}^{11}B_8$ $[M+H]^+$: 261.2646. The data were the same as the reported ones.[4]

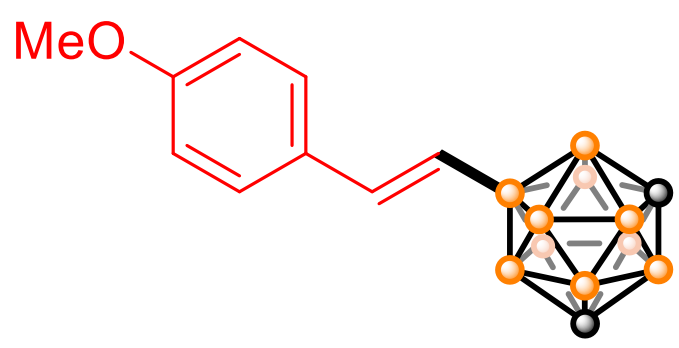

3ff: R_f (hexane/EA = 5:1): 0.51. 21.5 mg, 78% yield. ^{1}H NMR (400 MHz, CD_2Cl_2): δ 7.37−7.32 (m, 2H), 6.87-6.82 (m, 2H) (aryl C*H*), 6.70 (d, *J* = 18.0 Hz, 1H), 6.27 (d, *J* = 18.0 Hz, 1H) (alkenyl C*H*), 3.79 (s, 3H) (CH_3), 3.03 (s, 2H) (Cage C*H*). ^{13}C{^{1}H} NMR (101 MHz, CD_2Cl_2): δ 159.6, 140.1, 132.0, 127.6, 114.2 (aryl and alkenyl *C*), 55.6

(CH_3), 54.7 (cage *C*H). Bcage-*C* was not observed. $^{11}B\{^{1}H\}$ NMR (128 MHz, CD_2Cl_2): δ -1.0 (1B) (*B*-C), -6.6 (2B), -9.9 (1B) (*B*-C), -13.1 – -14.0 (4B), -17.4 (1B), -20.0 (1B). The data were the same as the reported ones.[4]

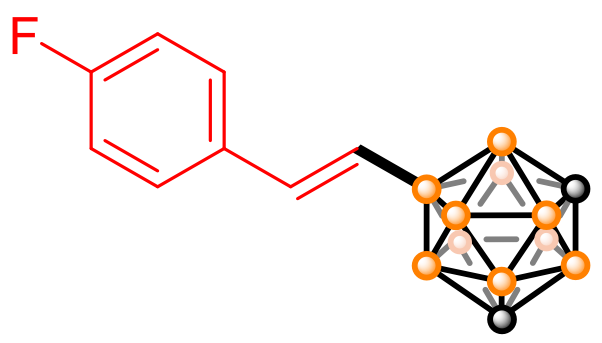

3fi: R_f (hexane/EA = 5:1): 0.57. 25.5 mg, 81% yield.Colorless oil. ^{1}H NMR (400 MHz, CD_2Cl_2): δ 7.43-7.36 (m, 2H), 7.06–6.96 (m, 2H) (aryl C*H*), 6.73 (d, J = 18.0 Hz, 1H), 6.35 (d, J = 18.0 Hz, 1H) (alkenyl C*H*), 3.04 (s, 2H) (Cage C*H*). $^{13}C\{^{1}H\}$ NMR (101 MHz, CD_2Cl_2): δ 162.6 (d, $^{1}J_{CF}$ = 245.2 Hz), 139.3, 135.4, 128.0 (d, $^{3}J_{CF}$ = 8.0 Hz), 115.6 (d, $^{2}J_{CF}$ = 21.8 Hz) (aryl and alkenyl *C*), 54.8 (cage *C*H). Bcage-*C* was not observed. $^{11}B\{^{1}H\}$ NMR (128 MHz, CD_2Cl_2): δ -1.4 (1B) (*B*-C), -6.6 (2B), -9.9 (1B) (*B*-C), -13.1 – -13.9 (4B), -17.4 (1B), -19.9 (1B). ^{19}F NMR (377 MHz, CD_2Cl_2): δ -116.1 (s, 1F). The data were the same as the reported ones.[4]

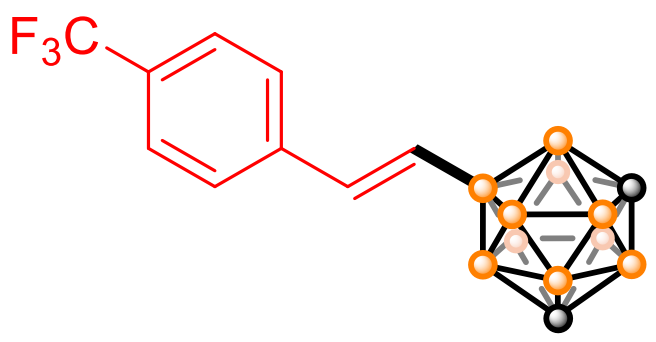

3fl: R_f (hexane/EA = 5:1): 0.50. 25.1 mg, 80% yield. ^{1}H NMR (400 MHz, CD_2Cl_2): δ 7.55 (d, J = 8.0 Hz, 2H), 7.43 (d, J = 8.0 Hz, 2H), 7.04 (s, 1H), 5.94 (d, J = 14.6 Hz, 1H), 2.92 (s, 2H). $^{13}C\{^{1}H\}$ NMR (101 MHz, CD_2Cl_2): δ 144.0, 139.8, 129.7, 128.9, 125.0–124.83 (m) (aryl and alkenyl *C*), 54.9 (cage *C*H). Bcage-*C* was not observed. $^{11}B\{^{1}H\}$ NMR (128 MHz, CD_2Cl_2): δ -3.5 (1B) (*B*-C), -6.4 (2B), -9.6 (1B) (*B*-C), -13.1 – -13.9 (4B), -17.5 (1B), -19.1 (1B). ^{19}F NMR (377 MHz, CD_2Cl_2): δ -61.4 (s, 3F). The data were the same as the reported ones.[4]

Preparation of (2,2-diphenylvinyl)-*m*-Carborane (5aa). 1a (0.10 mmol), 1,1-diphenylethlene **4a** (3 equiv, 0.3 mmol), $Ni(PPh_3)_2I_2$ (0.1 equiv, 8.4 mg, 0.01 mmol), $P(4\text{-}F\text{-}C_6H_4)_3$ (0.4 equiv, 12.8 mg, 0.04 mmol), Zn (1 equiv, 6.4 mg, 0.1 mmol) and Cs_2CO_3 (2 equiv, 65.2 mg, 0.2 mmol) were mixed in MeCN (1 mL). The resulting

mixture was stirred with 30W blue LED for 48 h under argon. After reaction, the reaction mixture was filtered through a suction funnel and washed with diethyl ether. The filtrate was concentrated to dryness in vacuo, which was subjected to flash column chromatography on silica gel (230-400 mesh) using *n*-hexane as eluent to give the products **5aa**.

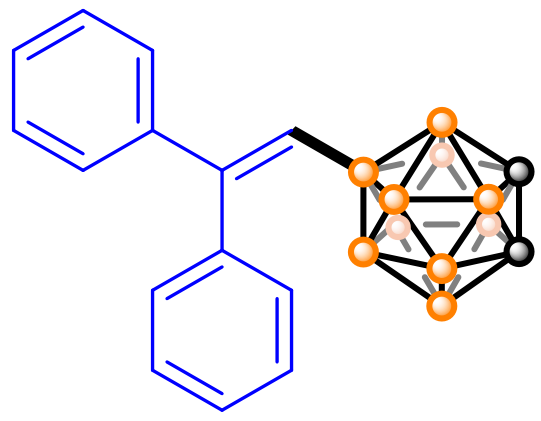

5aa: R_f (hexane/EA = 5:1): 0.62. 18.5 mg, 57% yield. ^{1}H NMR (400 MHz, CD_3CN): δ 7.35-7.10 (m, 10H) (aryl C*H*), 6.19 (s, 1H) (alkenyl C*H*), 3.90 (s, 1H), 3.84 (s, 1H) (Cage C*H*). ^{11}B{^{1}H} NMR (128 MHz, CD_2Cl_2): δ 3.5 (1B) (*B*-C), -3.7 (1B), -10.0 (2B), -14.9 – -16.5 (6B). These data are the same as the reported ones.[5]

Preparation of 9-[(3',4'-dihydronaphthalen-1-yl)methyl]-*o*-Carborane (5ab). **1a** (0.10 mmol), (1-cyclopropylvinyl)benzene **4b** (3 equiv, 0.3 mmol), $Ni(PPh_3)_2I_2$ (0.1 equiv, 8.4 mg, 0.01 mmol), $P(4\text{-}F\text{-}C_6H_4)_3$ (0.4 equiv, 12.8 mg, 0.04 mmol), Zn (1 equiv, 6.4 mg, 0.1 mmol) and Cs_2CO_3 (2 equiv, 65.2 mg, 0.2 mmol) were mixed in MeCN (1 mL). The resulting mixture was stirred with 30W blue LED for 48 h under argon. After reaction, the reaction mixture was filtered through a suction funnel and washed with diethyl ether. The filtrate was concentrated to dryness in vacuo, which was subjected to flash column chromatography on silica gel (230-400 mesh) using *n*-hexane as eluent to give the products **5ab**.

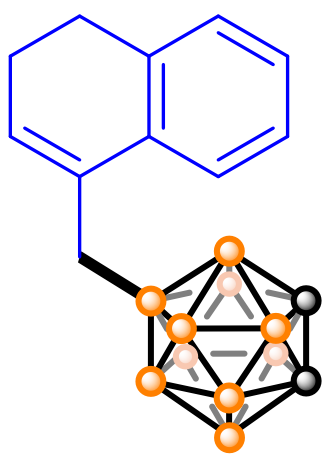

5ab: Rf (hexane/EA = 5:1): 0.81. 6.0 mg, 21% yield. Colorless oil. ^{1}H NMR (400 MHz, $CDCl_3$): δ 7.27-26 (m, 1H), 7.21 – 7.16 (m, 3H), 5.54 (t, *J* = 7.0 Hz, 1H), 3.34 (s, 2H), 2.61 – 2.55 (m, 2H), 2.19 (d, *J* = 7.1 Hz, 2H), 2.06 (s, 2H). ^{13}C NMR (101 MHz, $CDCl_3$): δ 144.3, 140.6, 136.9, 128.3, 127.9, 126.9, 126.2, 125.8 (aryl and alkyl *C*), 53.2, 48.4 (cage *C*H), 29.1,

22.4 (alkyl *C*). $^{11}B\{^1H\}$ NMR (128 MHz, $CDCl_3$): δ 6.9 (1B) (*B*-C), -2.8 (1B), -9.7 (2B), -14.8, -15.5, -16.6 (6B). HRMS (APCI): *m/z* calcd for $C_{13}H_{22}{}^{10}B_2{}^{11}B_8$ $[M+H]^+$: 287.2804. Found: 287.2800.

UV-Vis test. According to the UV-Vis spectrum, Ni(II) with Zn mixture had absorption peak of 335 nm, and Ni(0) species did not have absorption peak at the position, which indicated that Ni(I) reduced from Ni(II) by Zn had the reaction activity and Ni(0) did not have the photo activity. And Ni(I) species mixed with 9-iodo-*o*-carborane exhibited similar absorption with Ni(I) species, which indicated that no oxidative addition intermediate was generated.

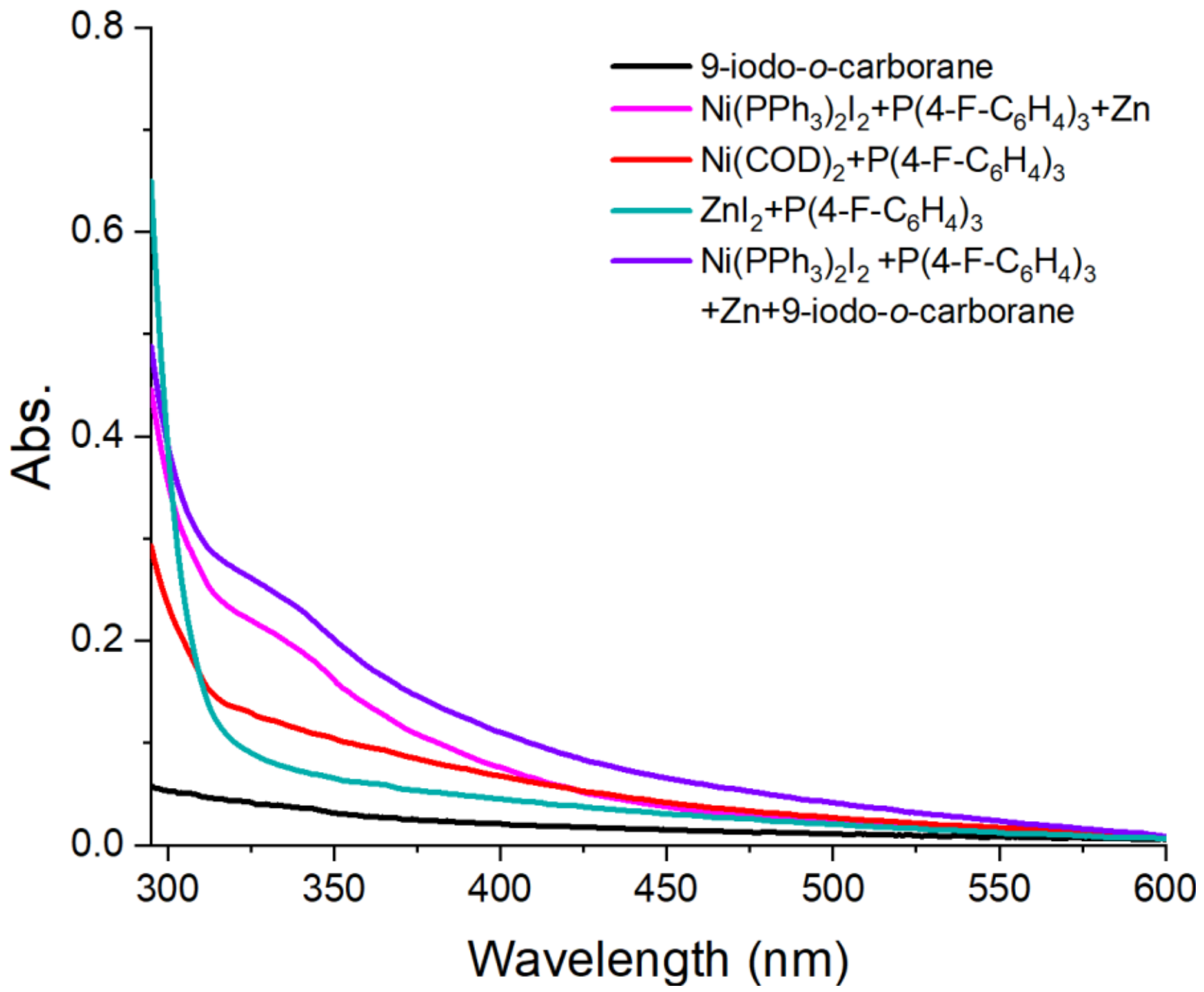

Fig. S9. UV-Vis absorption spectrum.

X-ray Structure Determination

Single-crystal X-ray data of compounds were collected at 293 K on a Bruker SMART 1000 CCD diffractometer using Mo-Kα radiation. An empirical absorption correction was applied using the SADABS program.[6] All structures were solved by direct methods and subsequent Fourier difference techniques and refined anisotropically for all non-hydrogen atoms by full-matrix least squares calculations on F^2 using the SHELXTL program package.[7] All hydrogen atoms were geometrically fixed using the riding model.

CCDC 2533189−2533195 for compounds (**3ah**, **3bb**, **3bi**, **3ce, 3ci, 3ea** and **3ei**) contain the supplementary crystallographic data for this paper. These data can be obtained free of charge from The Cambridge Crystallographic Data Centre via www.ccdc.cam.ac.uk/data_request/cif.

Table S1. Crystal data and structure refinement parameters of **3ah, 3bb, 3bi**, and **3ce**.

	**3ah**	**3bb**	**3bi**	**3ce**
CCDC number	2533189	2533190	2533191	2533192
Empirical formula	$C_{12}H_{23}B_{10}N$	$C_{11}H_{20}B_{10}$	$C_{10}H_{17}B_{10}F$	$C_{14}H_{26}B_{10}$
Formula weight	289.41	260.37	264.33	302.45
Temperature (K)	296(2)	296(2)	273.15	173.15
Crystal system	orthorhombic	orthorhombic	orthorhombic	orthorhombic
Space group	Pbca	$Pna2_1$	$Pna2_1$	Pccn
a (Å)	14.324(4)	33.476(3)	33.476(3)	25.8400(18)
b (Å)	15.084(4)	6.5423(6)	6.5423(6)	11.1749(8)
c (Å)	16.718(5)	6.9319(6)	6.9319(6)	12.8560(9)
α (°)	90	90	90	90
β (°)	90	90	90	90
γ (°)	90	90	90	90
Volume (Å)	3612.0(17)	1518.2(2)	1518.2(2)	3712.3(5)
Z	8	4	4	8
ρ_c (g/cm^3)	1.064	1.139	1.157	1.082
μ (mm^{-1})	0.053	0.054	0.064	0.052
F (000)	1216	544	544	1280
Crystal size (mm^3)	0.5 × 0.4 × 0.3	0.4 × 0.3 × 0.2	0.13 × 0.12 × 0.11	0.5 × 0.4 × 0.3
Radiation	MoKα (λ = 0.71073)	MoKα (λ = 0.71073)	MoKα (λ = 0.71073)	MoKα (λ = 0.71073)
2Θ range for data collection (°)	4.616 to 50.5	6.346 to 50.486	4.868 to 56.168	5.08 to 55.944
Index ranges	-17 ≤ h ≤ 17, -18 ≤ k ≤ 18, -20 ≤ l ≤ 20	-40 ≤ h ≤ 40, -7 ≤ k ≤ 7, -8 ≤ l ≤ 8	-44 ≤ h ≤ 44, -8 ≤ k ≤ 8, -9 ≤ l ≤ 9	-34 ≤ h ≤ 34, -14 ≤ k ≤ 14, -16 ≤ l ≤ 16
Reflections collected	96408	36886	46443	113522
Independent reflections	3250 [R_{int} = 0.0447, R_{sigma} = 0.0117]	2692 [R_{int} = 0.0539, R_{sigma} = 0.0233]	3674 [R_{int} = 0.0599, R_{sigma} = 0.0290]	4449 [R_{int} = 0.0730, R_{sigma} = 0.0245]
Data/restraints/parameters	3250/23/227	2692/8/202	3674/7/191	4449/6/224
Goodness-of-fit on F^2	1.052	1.163	1.073	1.049
Final R indexes [I> = 2σ (I)]	R_1 = 0.0975, wR_2 = 0.2588	R_1 = 0.0720, wR_2 = 0.1953	R_1 = 0.0521, wR_2 = 0.1361	R_1 = 0.0881, wR_2 = 0.2335
Final R indexes [all data]	R_1 = 0.1125, wR_2 = 0.2774	R_1 = 0.0757, wR_2 = 0.2013	R_1 = 0.0635, wR_2 = 0.1442	R_1 = 0.1148, wR_2 = 0.2553
Largest diff. peak/hole (e Å^{-3})	0.53/-0.28	0.59/-0.29	0.16/-0.24	0.60/-0.42

Table S2. Crystal data and structure refinement parameters of **3ci**, **3ea**, and **3ei**.

	**3ci**	**3ea**	**3ei**
CCDC number	2533193	2533194	2533195
Empirical formula	$C_{10}H_{17}B_{10}F$	$C_{18}H_{24}B_{10}$	$C_9H_{11}B_5F$
Formula weight	264.33	348.47	192.23
Temperature (K)	296	300	172
Crystal system	monoclinic	monoclinic	monoclinic
Space group	$P2_1/c$	$P2_1/n$	C2/c
a (Å)	9.8091(8)	10.2002(8)	18.904(4)
b (Å)	11.1430(8)	13.5863(12)	9.616(2)
c (Å)	14.6638(11)	15.7091(13)	12.114(2)
α (°)	90	90	90
β (°)	103.719(4)	95.653(2)	104.209(7)
γ (°)	90	90	90
Volume (Å)	1557.1(2)	2166.4(3)	2134.8(8)
Z	4	4	8
ρ_c (g/cm^3)	1.128	1.068	1.196
μ (mm^{-1})	0.468	0.053	0.073
F (000)	544	728	792
Crystal size (mm^3)	0.13 × 0.12 × 0.11	0.13 × 0.12 × 0.11	0.13 × 0.12 × 0.11
Radiation	CuKα (λ = 1.54178)	MoKα (λ = 0.71073)	MoKα (λ = 0.71073)
2Θ range for data collection (°)	9.28 to 136.508	4.564 to 49.996	4.784 to 55.712
Index ranges	-11 ≤ h ≤ 11, -13 ≤ k ≤ 13, -17 ≤ l ≤ 17	-12 ≤ h ≤ 12, -16 ≤ k ≤ 16, -18 ≤ l ≤ 18	-24 ≤ h ≤ 24, -12 ≤ k ≤ 12, -15 ≤ l ≤ 14
Reflections collected	17876	23378	21586
Independent reflections	2837 [R_{int} = 0.0412, R_{sigma} = 0.0327]	3816 [R_{int} = 0.0995, R_{sigma} = 0.0648]	2519 [R_{int} = 0.0303, R_{sigma} = 0.0166]
Data/restraints/parameters	2837/713/310	3816/9/253	2519/0/136
Goodness-of-fit on F^2	1.074	1.024	1.052
Final R indexes [I> = 2σ (I)]	R_1 = 0.0808, wR_2 = 0.2105	R_1 = 0.0823, wR_2 = 0.2197	R_1 = 0.0603, wR_2 = 0.1626
Final R indexes [all data]	R_1 = 0.0959, wR_2 = 0.2267	R_1 = 0.1716, wR_2 = 0.2995	R_1 = 0.0744, wR_2 = 0.1795
Largest diff. peak/hole (e Å^{-3})	0.23/-0.22	0.54/-0.20	0.35/-0.20

3 DFT Calculations

3.1 Calculational details for the energy profiles

All of the DFT calculations were performed with the Gaussian 09 program package.[8] The geometry optimization of all the minima involved were performed at the (U)B3LYP level of theory[9,10] with Grimme's dispersion correction with Becke-Johnson Damping (D3-BJ)[11] and def2-SVP basis set for all atoms. The structures of the reactants, intermediates, transition states, and products were fully optimized without any restriction. The vibrational frequencies were computed at the same level to check whether each optimized structure is an energy minimum or a transition state and to evaluate its zero-point vibrational energy (ZPVE) and thermal corrections at 298 K. IRC calculations[12-15] were used to confirm that the transition states found from the optimization calculations connect the related reactants and products. Single-point solvent calculations were performed at the (U)M06 level of theory[16] with Grimme's D3 empirical dispersion correction[17] and def2-TZVP basis set at the optimized geometries for all the intermediates and transition states. Solvation effects were taken into account by applying the SMD[18] solvation model with acetonitrile solvent both in geometry optimization and single-point energy calculations. The vertical excitation energies were calculated by time-dependent density functional theory (TD-DFT)[19-21] calculations at the (U)M06-D3/def2-TZVP/SMD(MeCN). The reported energies are Gibbs free energies in acetonitrile solution (ΔG_{sol}). The 3D structures were presented using the CYLview visualization software.[22]

3.2 DFT-Computed Energies of All Stationary Points

Table S3. Sum of electronic and thermal free energies (*G*, in Hartree), thermal correction to Gibbs free energy (*CGFE*, in Hartree), electronic energy in acetonitrile (E_{sol}, in Hartree), and free energies in acetonitrile (G_{sol}, in Hartree).

Structure	*G*	*CGFE*	E_{sol}	G_{sol}	Ifreq
**1a**	-629.031996	0.132564	-629.207198	-629.074634	
**L_2NiI**	-4472.310853	0.416196	-4473.751617	-4473.335421	
**INT1**	-5101.344805	0.574164	-5102.983727	-5102.409563	
**INT2**	-331.121488	0.134116	-331.332922	-331.198806	
**L_2NiI_2**	-4770.141532	0.42417	-4771.548481	-4771.124311	
**2a**	-309.362791	0.102388	-309.512172	-309.409784	
**TS1**	-640.473813	0.253995	-640.854502	-640.600507	-44.62
**INT3**	-640.542096	0.258443	-640.921024	-640.662581	
**INT4**	-640.385310	0.261751	-640.765218	-640.503467	
**$L_2NiI_2^-$**	-4770.304622	0.414970	-4771.711121	-4771.296151	
**$(Cs_2CO_3)_2$**	-608.276388	-0.021703	-608.476961	-608.498664	
**$CsICsHCO_3$**	-602.624734	-0.018382	-602.678292	-602.696674	
**3a**	-639.970283	0.249254	-640.339734	-640.090480	

For transition state structures, one imaginary frequency (Ifreq) was observed and given below. For all minimum structures, no imaginary frequency was observed.

The coordinates of all stationary points (Å)

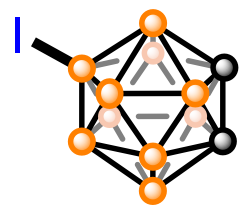

1a

C	-3.12150200	0.00000200	0.00535300
H	-4.20421300	0.00000700	-0.11076000
B	-0.84604600	-1.45260400	0.48507300
B	-2.35934800	-0.88986400	1.22844300
H	-3.03394500	-1.48675700	2.01177400
B	-2.34108800	-1.44927600	-0.45788300
H	-3.00212000	-2.34566900	-0.87703900
B	-0.85232500	-0.00012700	1.53406600
H	-0.33438700	-0.00022700	2.61436800
B	-0.82951500	-0.89175300	-1.20645700
H	-0.40352100	-1.48355900	-2.15031900
B	-2.35934200	0.88967500	1.22858900
H	-3.03395300	1.48642700	2.01200900
B	-2.34108900	1.44935000	-0.45764500
H	-3.00210700	2.34581200	-0.87667500
H	-2.82855800	0.00019500	-2.29219100
B	-0.84604200	1.45252400	0.48530400
H	-0.32482500	2.48233200	0.80209500
B	-0.82951700	0.89194400	-1.20631500
H	-0.40350700	1.48391100	-2.15006600
H	-0.32482400	-2.48246400	0.80170400
B	0.08077800	-0.00000300	0.02646900
C	-2.25886500	0.00010900	-1.36413500
I	2.27916700	0.00000000	0.00131300

L_2NiI

Ni	-0.00494300	-1.24014800	-0.43519300
P	1.76593200	0.10025700	-0.08655000
C	2.38700300	-0.01230500	1.63273400
C	3.04474000	1.03115300	2.30147600
C	2.16843100	-1.22792000	2.30308200
C	3.47091200	0.87071400	3.62164800
H	3.22333700	1.98331700	1.79959100
C	2.59788100	-1.40577000	3.61777900
H	1.64682600	-2.03913900	1.78902200
C	3.23825200	-0.34635600	4.25673500
C	3.19583700	-0.39192900	-1.12318700
C	4.50949700	0.01837300	-0.84360200
C	2.95071600	-1.20439900	-2.24041300
C	5.56363400	-0.36863300	-1.67053000
H	4.71700500	0.64556000	0.02590000
C	3.99574500	-1.59525400	-3.08068600
H	1.93224900	-1.53903700	-2.45085400
C	5.28520700	-1.16930700	-2.77758200
C	1.59960500	1.89380100	-0.41861800
C	2.03214200	2.44587100	-1.63519700
C	0.88234200	2.70326200	0.47888300
C	1.74736100	3.77433300	-1.95812200
H	2.58520000	1.83501400	-2.35061600
C	0.59148900	4.03129500	0.16904000
H	0.54372000	2.29829400	1.43131100
C	1.02414900	4.54314300	-1.05144600
H	-0.31655000	1.09112900	-2.21385700
C	-2.76900100	3.20679800	-3.20141100
C	-1.70518200	0.86302200	1.54522600
C	-2.19145900	2.15720500	1.77874000
C	-0.98010200	0.20990600	2.55675000
C	-1.95207700	2.79563100	2.99778100
H	-2.74167600	2.69053400	1.00304300
C	-0.73533000	0.83262100	3.77974800
H	-0.58357600	-0.79093600	2.37605400
C	-1.22202800	2.12329600	3.97450000
C	-3.37353200	-1.00201200	0.09129400
C	-4.32793500	-0.80147000	1.09919900
C	-3.58558400	-2.00907500	-0.86624500
C	-5.48408400	-1.58500000	1.14833600
H	-4.17484900	-0.02956100	1.85559500
C	-4.73869100	-2.79238900	-0.83401900
H	-2.83539100	-2.18821300	-1.64047100
C	-5.66964200	-2.56481800	0.17765800
P	-1.83055800	-0.02140600	-0.05289400
C	-2.26108600	1.25181400	-1.29483100
C	-3.51704300	1.87988700	-1.34738500
C	-3.77623000	2.86563200	-2.30020700
C	-1.27931500	1.60107000	-2.23434700
H	-4.30290000	1.60084000	-0.64242500
C	-1.52293300	2.58704700	-3.19145100
H	-6.23726000	-1.44385800	1.92607600
H	-4.91871900	-3.57806400	-1.57036400
H	-0.16087600	0.34107700	4.56705300

H	-2.31332600	3.80725700	3.19248000
H	2.43302400	-2.34302400	4.15271300
H	3.97788900	1.67400200	4.15973500
H	6.59210200	-0.06194600	-1.46998300
H	3.82314300	-2.22646500	-3.95451100
H	-0.76529100	2.87390300	-3.92260300
H	-4.74359900	3.36870200	-2.35501800
H	0.03109500	4.66552400	0.85835900
H	2.07325600	4.21364600	-2.90284300
I	0.13805500	-3.78717600	-0.73009000
F	-3.01545300	4.15896300	-4.11809500
F	-6.78118400	-3.32164400	0.22036600
F	-0.97802100	2.74061200	5.14388800
F	3.64237000	-0.50344300	5.52943600
F	0.73783200	5.82089000	-1.36114700
F	6.29933400	-1.54451900	-3.57661800

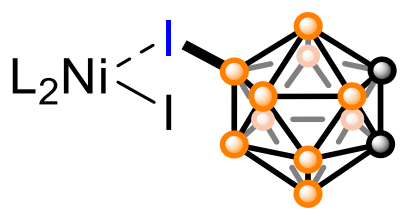

INT1

Ni	-0.09805300	0.02743200	-0.53655000
P	1.03545900	1.83437900	0.17494300
C	2.34683700	2.56853700	-0.88511700
C	3.47964500	3.22901500	-0.38401500
C	2.17510200	2.47046400	-2.27575500
C	4.43551100	3.76313000	-1.25107100
H	3.63242600	3.32892000	0.69139800
C	3.11500000	3.00983300	-3.15410400
H	1.30025100	1.95432000	-2.67757300
C	4.23679200	3.64105500	-2.62346700
C	-0.07711200	3.26809200	0.46369900
C	0.37557200	4.47331400	1.02660200
C	-1.42062900	3.15428400	0.07994600
C	-0.49964300	5.54341400	1.21073400
H	1.41940800	4.58132600	1.32911200
C	-2.30919700	4.21804500	0.25325100
H	-1.78189500	2.22220400	-0.35432200
C	-1.83000300	5.39492800	0.81858300
C	1.84165700	1.57573000	1.79743900
C	1.12864800	1.80613400	2.98641700
C	3.09741000	0.94733500	1.87068400
C	1.65239300	1.41867700	4.22174800
H	0.14457000	2.27646400	2.95440900
C	3.63278000	0.55730600	3.09813300
H	3.66421700	0.74392300	0.96246900
C	2.89474600	0.79280500	4.25462900
H	0.89071800	-0.72948800	2.53972700
C	1.41129100	-3.72498200	4.02098000
C	2.98369300	-1.46892200	-0.57093900
C	4.05675100	-1.93957700	0.19951400
C	3.24402300	-0.63963700	-1.67544400
C	5.36942800	-1.58093400	-0.11722100
H	3.87535800	-2.57576900	1.06668500
C	4.54866100	-0.27983600	-2.00848100
H	2.41142800	-0.25634100	-2.26742200
C	5.59077500	-0.75209200	-1.21356100
C	0.68585800	-3.10889000	-1.25124700
C	1.55892600	-3.83907600	-2.06844900
C	-0.69015900	-3.40116000	-1.27955000
C	1.07351800	-4.85258500	-2.90053000
H	2.62857000	-3.62177700	-2.06116600
C	-1.18562700	-4.41698300	-2.09527100
H	-1.38182000	-2.82355300	-0.66126200
C	-0.29069100	-5.12490900	-2.89583600
P	1.22548000	-1.74887900	-0.14600100
C	1.29016300	-2.57488500	1.49161600
C	1.53734000	-3.95019700	1.63521300
C	1.59885200	-4.53365900	2.90193900
C	1.09491900	-1.79401600	2.64084900
H	1.68737800	-4.57648600	0.75389000
C	1.15979600	-2.36034600	3.91442700
H	1.73903700	-5.42996600	-3.54537600
H	-2.25028900	-4.65654900	-2.12805300
H	4.76242700	0.37493000	-2.85507800
H	6.21567600	-1.92937400	0.47810900
H	2.99420800	2.93488600	-4.23655700
H	5.32601300	4.27105400	-0.87553100
H	-0.16751700	6.48655900	1.64917700
H	-3.35739400	4.13916000	-0.04053300
H	1.02005000	-1.75827400	4.81396500
H	1.78981100	-5.60083000	3.03163500
H	4.60165200	0.05871400	3.16278700
H	1.10399200	1.58595800	5.15073800
I	-1.05108000	0.13041700	-2.99322700
F	1.47072500	-4.28355800	5.24323500
F	-0.76350700	-6.10118700	-3.69280900
F	6.85227500	-0.39474500	-1.51548700

F	5.15784900	4.14864100	-3.46278000
F	3.39151700	0.39205400	5.43996900
F	-2.67569600	6.42642400	0.99419400
C	-7.01549400	0.13193100	-0.48473800
H	-8.06176200	0.15409300	-0.78558100
B	-4.98324000	1.33384300	0.90878800
B	-6.06302600	1.53109500	-0.48862200
H	-6.53892500	2.55207800	-0.88345300
B	-6.67949000	0.84912700	1.03155500
H	-7.58061000	1.30823400	1.65879900
B	-4.41214000	0.92913700	-0.73872800
H	-3.59679700	1.59017000	-1.30490400
B	-5.41701700	-0.19450300	1.71650600
H	-5.41908000	-0.40535700	2.89055800
B	-5.76272600	0.19328000	-1.62385800
H	-6.04022900	0.31932000	-2.77781300
B	-6.19381300	-1.33009000	-0.81860500
H	-6.79575400	-2.21762300	-1.33450600
H	-7.48035000	-1.39418900	1.19349100
B	-4.49662600	-0.85245600	-0.94387500
H	-3.73837700	-1.46655200	-1.63323100
B	-5.12082400	-1.53488200	0.57905600
H	-4.92302500	-2.63281400	1.00114200
H	-4.56541800	2.26596500	1.53101500
B	-4.02850300	-0.14076300	0.61800500
C	-6.65195400	-0.83897200	0.75584100
I	-1.99951400	-0.38373400	1.42435600

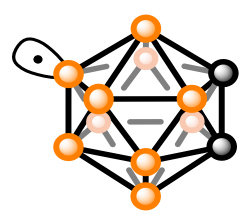

INT2

C	-0.00000200	0.04783200	1.46104100
H	-0.00000200	-0.03527700	2.54698600
B	1.45269700	0.46268900	-0.82895700
B	0.89108800	1.25045400	0.66980300
H	1.48593200	2.05096400	1.32723600
B	1.45061500	-0.43863900	0.69735500
H	2.34441200	-0.83872500	1.37475600
B	-0.00002000	1.51264300	-0.84978500
H	-0.00003300	2.58853000	-1.38306100
B	0.89050400	-1.22798900	-0.79746200
H	1.48627300	-2.19036400	-1.18175300
B	-0.89112400	1.25043100	0.66980100
H	-1.48599000	2.05092400	1.32723500
B	-1.45060500	-0.43867700	0.69735300
H	-2.34439200	-0.83879000	1.37475100
H	0.00003000	-2.25627200	1.24649800
B	-1.45270800	0.46265000	-0.82896000
H	-2.49308900	0.76966800	-1.34147700
B	-0.89046900	-1.22801400	-0.79746200
H	-1.48621200	-2.19040500	-1.18175300
H	2.49307000	0.76973800	-1.34147100
B	0.00000200	-0.02106400	-1.71198700
C	0.00001800	-1.34823300	0.64455100

L_2NiI_2

Ni	-0.12459000	-1.08545000	0.36623000
P	1.65519100	0.13372500	-0.19443000
C	2.75458500	0.86103100	1.07286600
C	3.76369700	1.76890900	0.70160700
C	2.59342100	0.53880000	2.42851500
C	4.59406800	2.34335600	1.66290000
H	3.90621800	2.03904200	-0.34556800
C	3.41488800	1.10868200	3.40113100
H	1.82156900	-0.16700600	2.73157900
C	4.40172400	2.00423100	3.00017900
C	2.67558200	-1.03744300	-1.17581400
C	4.07426900	-1.06735600	-1.09646200
C	2.01570800	-1.94221600	-2.02663400
C	4.80880300	-1.97595800	-1.86219700
H	4.60891000	-0.39256500	-0.42801000
C	2.73782200	-2.84570500	-2.80370400
H	0.92486600	-1.95920700	-2.07108000
C	4.12750900	-2.84658600	-2.70659700
C	1.40541000	1.52864900	-1.36759000
C	1.33611500	1.29615900	-2.75125700
C	1.24895600	2.84233000	-0.89410900
C	1.09905600	2.34140500	-3.64300200
H	1.46382300	0.29129200	-3.15176000
C	1.01126300	3.89700600	-1.77432500
H	1.32378300	3.05722700	0.17014200
C	0.93247300	3.62687900	-3.13705000
H	-0.78147900	-0.48122400	-2.34928000
C	-3.03957000	1.32540900	-4.10856200
C	-1.15218100	2.06052700	0.98999300
C	-1.52006900	3.33506300	0.53439900
C	-0.35294600	1.94902900	2.13900100

C	-1.09958500	4.47792400	1.21479600
H	-2.11419500	3.45697600	-0.36965400
C	0.07834800	3.08225700	2.82741500
H	-0.05162400	0.96301400	2.49284700
C	-0.30401800	4.33221500	2.34868800
C	-3.16452000	0.05161300	1.15232400
C	-3.10700200	0.05340300	2.55545600
C	-4.34393800	-0.36940600	0.51746900
C	-4.20851300	-0.34116500	3.31517200
H	-2.19835200	0.35852800	3.07535700
C	-5.45565600	-0.75671600	1.26612500
H	-4.40369500	-0.40824200	-0.56967100
C	-5.36839200	-0.73707800	2.65495900
P	-1.66192300	0.49314400	0.19042300
C	-2.23770200	0.85761100	-1.49352700
C	-3.30933900	1.72982700	-1.75972200
C	-3.71114200	1.97243200	-3.07226400
C	-1.59543900	0.21062800	-2.55922200
H	-3.85001200	2.21093300	-0.94350000
C	-1.99124200	0.44093100	-3.87602400
H	-4.17560500	-0.34380100	4.40622500
H	-6.38034200	-1.07979100	0.78425700
H	0.71232500	3.00530900	3.71219300
H	-1.36806700	5.47675100	0.86601000
H	3.29417700	0.86982400	4.45929400
H	5.38105300	3.04814100	1.38869000
H	5.89828200	-2.01568300	-1.80582900
H	2.23782800	-3.55306800	-3.46779500
H	-1.49697700	-0.05162300	-4.71507300
H	-4.53894100	2.64622300	-3.30030900
H	0.88367600	4.91857200	-1.41264300
H	1.03800900	2.16720700	-4.71857000
I	-1.86638700	-2.90102100	-0.32687700
F	-3.42592500	1.55723000	-5.37301600
F	-6.43495200	-1.11378700	3.38076600
F	0.10955000	5.43229700	2.99836000
F	5.19279700	2.56109900	3.93071300
F	0.69813500	4.63855200	-3.98985600
F	4.83062500	-3.72159600	-3.44550500
I	1.37671800	-2.83391300	1.60621800

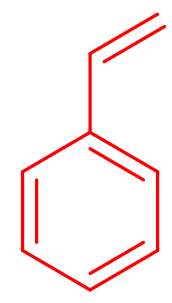

2a

C	-0.51604700	-0.22590300	0.00006900
C	0.40943300	-1.28707800	0.00004300
C	1.78556000	-1.04521400	0.00003500
C	2.26584400	0.26721900	0.00002100
C	1.35752800	1.33428300	-0.00001800
C	-0.01542300	1.09226700	-0.00002700
C	-1.95640600	-0.53582800	-0.00009700
C	-2.97378500	0.33917400	0.00006100
H	0.03778400	-2.31548800	0.00000100
H	2.48457300	-1.88555700	0.00000200
H	3.34143100	0.46085800	-0.00002500
H	1.72509100	2.36372600	-0.00019100
H	-0.70783900	1.93688100	-0.00019400
H	-2.82000800	1.42259500	0.00031100
H	-2.19108100	-1.60671000	-0.00056600
H	-4.01017400	-0.00982500	0.00013800

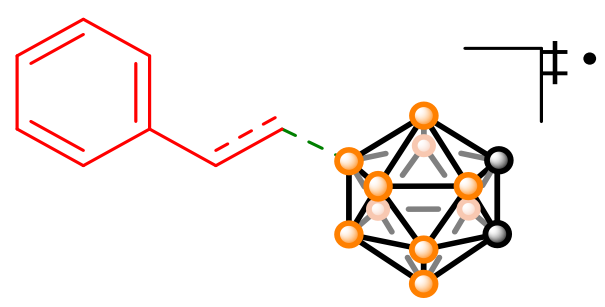

TS1

C	-3.23472700	-1.31548100	0.47063400
H	-3.97647800	-2.07856400	0.70158100
B	-0.98915200	-0.61876300	-0.94830900
B	-1.58974500	-1.69545500	0.34203000
H	-1.24054700	-2.81651400	0.56279400
B	-2.57632900	-1.39547900	-1.10600800
H	-2.96468700	-2.24774500	-1.84174700
B	-0.76471900	-0.17937600	0.77172500
H	0.31286700	-0.19608300	1.29565800
B	-2.37819900	0.31379300	-1.56321100
H	-2.59371900	0.61837500	-2.69917900
B	-2.21691700	-0.68482100	1.66683200
H	-2.28715400	-1.12995600	2.77337100
B	-3.59820100	0.25220100	1.05196100
H	-4.61646700	0.41610400	1.64747800
H	-4.69245700	-0.28995700	-1.00191800
B	-2.01213100	1.02728900	1.20998700

H	-1.84233400	1.87644700	2.04073400
B	-3.00471700	1.32334800	-0.24041500
H	-3.64019800	2.30511100	-0.48921000
H	-0.08984600	-0.95265800	-1.66656800
B	-1.27969900	1.03355700	-0.39643800
C	-3.68467200	-0.19286800	-0.59935700
C	2.81741800	0.78633600	0.08006400
C	3.57748500	0.31515000	1.16914200
C	4.31658000	-0.86627200	1.07741900
C	4.31283300	-1.60574100	-0.10927200
C	3.56423900	-1.14971700	-1.20244200
C	2.82663200	0.02981300	-1.11087100
C	2.03479300	2.01954800	0.23483000
C	1.23333600	2.58570400	-0.68797200
H	3.57912600	0.88690300	2.10112700
H	4.89636500	-1.21216700	1.93713500
H	4.88806800	-2.53193000	-0.18403700
H	3.55465700	-1.72186100	-2.13384500
H	2.24621400	0.36546400	-1.97216000
H	2.10214100	2.48583400	1.22425100
H	1.13659600	2.19389900	-1.70286600
H	0.69339300	3.50900100	-0.46642000

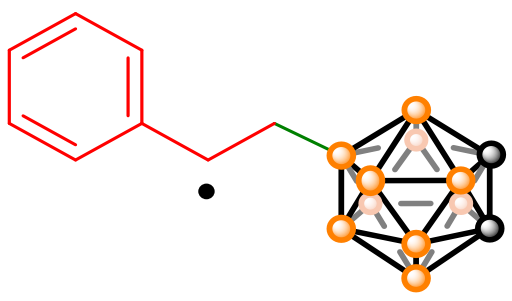

INT3

C	-3.45879300	-0.84063400	0.76369600
H	-4.36462900	-1.33496200	1.11120300
B	-0.82476400	-1.07691800	0.03668400
B	-1.94029900	-1.20952300	1.41525300
H	-1.87820400	-1.99184400	2.31586800
B	-2.41674700	-1.81213800	-0.18400900
H	-2.73922900	-2.93599200	-0.41014500
B	-1.00024900	0.27820300	1.18336000
H	-0.12552900	0.57707000	1.94664000
B	-1.78827000	-0.68523000	-1.40314800
H	-1.61318400	-1.09196800	-2.51293300
B	-2.70514400	0.37641100	1.66903900
H	-3.15557700	0.65563200	2.73959500
B	-3.66448400	0.77124200	0.22867600
H	-4.75732700	1.24359500	0.25594300
H	-4.22912300	-0.86510100	-1.41681800

B	-2.07027500	1.50099700	0.44766300
H	-1.96855100	2.67628600	0.66379800
B	-2.55413100	0.89470200	-1.15159100
H	-2.89198100	1.55120800	-2.09100700
H	0.16163000	-1.75207400	-0.03444700
B	-0.88871600	0.61314600	-0.57187700
C	-3.37292200	-0.54641300	-0.82403400
C	2.60114500	0.67693100	-0.16290300
C	3.58544400	0.95377600	0.83695900
C	4.66467100	0.10537300	1.04465600
C	4.81841200	-1.05685400	0.26911400
C	3.86717300	-1.35125400	-0.72082800
C	2.78141700	-0.51025200	-0.93785400
C	1.50649400	1.55735600	-0.34541900
C	0.36300800	1.35136600	-1.27395500
H	3.47207500	1.85452900	1.44673500
H	5.39913100	0.34349800	1.81902300
H	5.66793800	-1.72358900	0.43495200
H	3.97842700	-2.25340500	-1.32878500
H	2.05352200	-0.76264900	-1.71015400
H	1.45279100	2.41947700	0.32742000
H	0.65964900	0.77081000	-2.16188700
H	0.00727500	2.32865000	-1.64182900

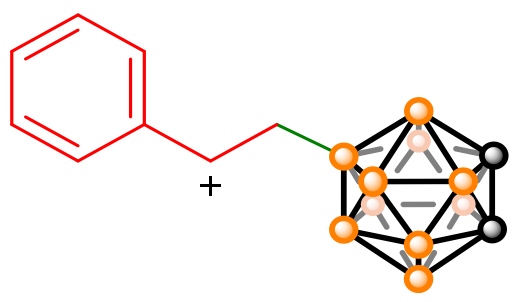

INT4

C	3.34398900	-0.98499000	-0.65092400
H	4.23260300	-1.54718000	-0.93443000
B	0.72453700	-1.04779600	0.14848600
B	1.80578600	-1.41631900	-1.21262800
H	1.70629400	-2.32542800	-1.97950700
B	2.30408700	-1.77730200	0.45339900
H	2.60681700	-2.85952800	0.84455200
B	0.90160000	0.11077100	-1.20234900
H	0.01167900	0.31191600	-1.97798500
B	1.72692100	-0.46263600	1.49567700
H	1.55687500	-0.67744300	2.65693500
B	2.59895500	0.09361300	-1.72347400
H	3.03099000	0.19330500	-2.83153000
B	3.59679400	0.68361900	-0.37837300
H	4.69717500	1.12032900	-0.49781500

H	4.15937600	-0.68795500	1.49518500
B	2.01530100	1.41133400	-0.68366400
H	1.92332300	2.54098100	-1.07079400
B	2.52284400	1.05020000	0.98485300
H	2.87966200	1.83745500	1.80728700
H	-0.28241200	-1.66499900	0.34175300
B	0.85742100	0.70302300	0.47559900
C	3.29807900	-0.44691300	0.87366100
C	-2.50550700	0.69329600	0.10553000
C	-3.36009300	0.78338700	-1.02983400
C	-4.42725900	-0.08993200	-1.17097100
C	-4.65683200	-1.06246200	-0.18657000
C	-3.82652500	-1.16670300	0.94309700
C	-2.75949000	-0.29827500	1.09622900
C	-1.41352400	1.58174300	0.19017100
C	-0.36396000	1.58288200	1.16519200
H	-3.15607700	1.54530700	-1.78498300
H	-5.08406400	-0.02489700	-2.04017200
H	-5.49830600	-1.75085700	-0.29761300
H	-4.02720900	-1.92985200	1.69710500
H	-2.11619000	-0.36844700	1.97341900
H	-1.32229800	2.30418300	-0.62908300
H	-0.61573700	1.09969600	2.11605900
H	0.05299900	2.58868600	1.31050600

$L_2NiI_2^-$

Ni	-0.02983800	-1.04156100	0.33527700
P	-1.83586600	0.23911500	0.05616100
C	-2.54845700	0.57462500	-1.61030200
C	-3.19050500	1.76937400	-1.97155600
C	-2.45398900	-0.45874900	-2.55731800
C	-3.70736900	1.94193700	-3.25786600
H	-3.29006800	2.58421700	-1.25320600
C	-2.97692800	-0.30674900	-3.84154600
H	-1.95220700	-1.39177300	-2.28945400
C	-3.58806900	0.89969300	-4.17261800
C	-3.31255000	-0.42805700	0.92907900
C	-4.49310400	0.31789000	1.08497900
C	-3.26367100	-1.74160500	1.41747600
C	-5.60400700	-0.23228600	1.72336700
H	-4.54867100	1.34091000	0.70669000
C	-4.37092300	-2.30981300	2.05374500
H	-2.35121300	-2.32919300	1.29896600
C	-5.52095200	-1.54122400	2.19731100
C	-1.60623100	1.89609800	0.80089600
C	-1.83776800	2.08581700	2.17463000
C	-0.98027400	2.92295200	0.07269100
C	-1.45919000	3.27086700	2.80836700
H	-2.30068800	1.29450600	2.76669300
C	-0.59677100	4.11205800	0.69348900
H	-0.77008700	2.79467400	-0.98858400
C	-0.83679900	4.26226900	2.05596800
H	0.70927000	1.59686700	1.98666700
C	3.67164700	3.01108100	2.80852700
C	1.44844800	1.29007300	-1.71638300
C	1.89630200	2.61703500	-1.78686800
C	0.62817800	0.78859300	-2.74089000
C	1.52176200	3.43776400	-2.85415900
H	2.52749400	3.02780600	-0.99773200
C	0.25218400	1.59228100	-3.81601200
H	0.26527400	-0.23869400	-2.68369400
C	0.70031900	2.91091100	-3.84686400
C	3.13919600	-0.88620900	-0.85665500
C	3.90046100	-0.61937600	-2.00327300
C	3.42908000	-2.02881900	-0.09055200
C	4.94235800	-1.47139000	-2.38082500
H	3.68565900	0.26055600	-2.61221600
C	4.47268600	-2.88124100	-0.44893000
H	2.82397500	-2.25465100	0.79042700
C	5.21131300	-2.58628900	-1.59298800
P	1.75658500	0.17973800	-0.28942700
C	2.56781800	1.27610500	0.93808800
C	3.93765500	1.58237600	0.89974100
C	4.49883000	2.45197200	1.83707200
C	1.76785800	1.84508400	1.94081900
H	4.57711900	1.14534000	0.13061600
C	2.31141500	2.72439000	2.87793700
H	5.54326300	-1.27966000	-3.27204300
H	4.71025200	-3.77213000	0.13577500
H	-0.39680000	1.21818800	-4.60974200
H	1.85168100	4.47648400	-2.91883900
H	-2.90400300	-1.10162100	-4.58647600
H	-4.20038500	2.86974700	-3.55496100
H	-6.52831500	0.33354000	1.85642600
H	-4.34804900	-3.33139700	2.43802900
H	1.69574800	3.18363100	3.65340400
H	5.56185100	2.70062900	1.82260100
H	-0.09948200	4.90870200	0.13705500

H	-1.62717700	3.42508700	3.87600100
I	0.42603500	-1.64973500	2.95339200
F	4.20752200	3.85302500	3.71160300
F	6.21428300	-3.41036000	-1.95036500
F	0.32723800	3.70086500	-4.87102300
F	-4.07886500	1.06355100	-5.41546200
F	-0.44229500	5.39523000	2.66905900
F	-6.59178900	-2.07627900	2.81238500
I	-0.06819300	-3.40407200	-1.00883300

$(Cs_2CO_3)_2$

C	0.58977400	-0.07230700	1.87074100
O	1.17143500	1.08375000	1.91249000
O	1.31230900	-1.14947700	1.92458600
O	-0.69385900	-0.15906300	1.72342100
Cs	-3.21337300	0.00307900	0.26824800
Cs	3.09274300	-0.02667700	-0.20690400
Cs	-0.16224900	-2.87484400	0.13120800
O	-1.29359300	-1.10991000	-1.85553000
C	-0.71145300	0.04583500	-1.81166400
O	-1.43342600	1.12347800	-1.86451300
O	0.57206800	0.13165100	-1.66301900
Cs	0.04183800	2.84929400	-0.07214800

$CsICsHCO_3$

C	-0.12695000	-1.87414000	1.16210200
O	0.01843400	-1.96339000	-0.07793900
O	1.07619600	-1.84867000	1.87477800
O	-1.17365800	-1.78120200	1.83082500
Cs	-2.71949400	-0.58260400	-0.49533300
H	0.84020600	-1.75363500	2.81209100
Cs	2.70214200	-0.61317200	-0.50441800
I	0.02845500	2.33042000	0.30528900

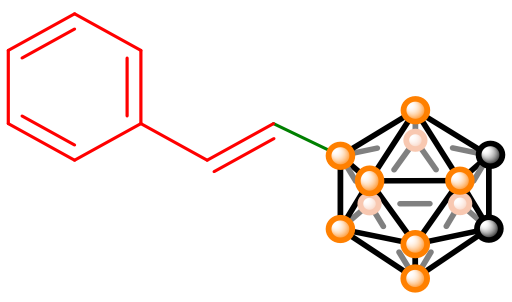

3a

C	4.15751000	0.29245300	-0.00669600
H	5.23881100	0.39738800	-0.07989800
B	1.68937200	1.08448800	-0.89980200
B	3.13107100	1.64174300	-0.02334700
H	3.61868500	2.73149600	-0.06033200
B	3.29596700	0.60065100	-1.45220800
H	3.91159100	0.89234200	-2.42888300
B	1.70196400	1.10411400	0.88361200
H	1.03985200	1.87961000	1.51556300
B	1.98034000	-0.59219000	-1.41817500
H	1.64431600	-1.09204600	-2.44960100
B	3.32582300	0.63215400	1.42831900
H	3.94098500	1.04735900	2.36431000
B	3.61337400	-1.04230700	0.91409900
H	4.42585000	-1.76564700	1.39829900
H	4.20474800	-1.60435700	-1.33017000
B	2.00595900	-0.55911400	1.46460700
H	1.56752000	-0.98267500	2.49664900
B	2.17449600	-1.59739200	0.03283200
H	1.96449600	-2.77196900	-0.02625800
H	1.02324300	1.83078500	-1.55973000
B	0.96787600	-0.28000400	0.02078100
C	3.50776800	-0.96292200	-0.79295600
C	-2.99443100	0.15137000	0.01044400
C	-3.83937400	1.27894300	0.01201900
C	-5.22945400	1.14213600	0.00097700
C	-5.80820300	-0.13030200	-0.01184100
C	-4.98238900	-1.26242000	-0.01314500
C	-3.59503600	-1.12487600	-0.00199100
C	-1.53658500	0.35539700	0.01926200
C	-0.57261700	-0.58614800	0.01659900
H	-3.39106600	2.27634100	0.02166300
H	-5.86301800	2.03295700	0.00220900
H	-6.89530100	-0.24213400	-0.02086800
H	-5.42621500	-2.26140600	-0.02321100
H	-2.96866500	-2.01952900	-0.00364000
H	-1.23443600	1.40986700	0.02602500
H	-0.87573700	-1.64086700	0.00823700

4 References

1 J. S. Andrews, J. Zayas and M. Jones, *Inorg. Chem.*, 2002, **41**, 3715–3716.

2 H. Lyu, Y. Quan and Z. Xie, *Chem. Eur. J.*, 2017,**23**, 14866–14871.

3 L. Ji, F. L. Cameron and J. J. Maitland, *Inorg. Chem.*, 1991, **30**, 4866–4868.

4 J.-R. Chang, H.-J. Cao, Y.-N. Ma and X. Chen, *Org. Lett.*, 2025, **27**, 1858–1863.

5 S. Li and Z. Xie, *J. Am. Chem. Soc.*, 2022, **144**, 7960–7965.

6 G. M. Sheldrick, *SADABS: Program for Empirical Absorption Correction of Area Detector Data*; University of Göttingen: Göttingen, Germany, 1996.

7 G. M. Sheldrick, *SHELXTL 5.10 for Windows NT: Structure Determination Software Programs*; Bruker Analytical X-ray Systems, Inc.: Madison, Wisconsin, USA, 1997.

8 M. J. Frisch, G. W. Trucks, H. B. Schlegel, G. E. Scuseria, M. A. Robb, J. R. Cheeseman, G. Scalmani, V. Barone, B. Mennucci, G. A. Petersson, H. Nakatsuji, M. Caricato, X. Li, H. P. Hratchian, A. F. Izmaylov, J. Bloino, G. Zheng, J. L. Sonnenberg, M. Hada, M. Ehara, K. Toyota, R. Fukuda, J. Hasegawa, M. Ishida, T. Nakajima, T. Honda, O. Kitao, H. Nakai, T. Vreven, J. A. Montgomery, Jr., J. E. Peralta, F. Ogliaro, M. Bearpark, J. J. Heyd, E. Brothers, K. N. Kudin, V. N. Staroverov, R. Kobayashi, J. Normand, K. Raghavachari, A. Rendell, J. C. Burant, S. S. Iyengar, J. Tomasi, M. Cossi, N. Rega, J. M. Millam, J. E. Klene, J. E. Knox, J. B. Cross, V. Bakken, C. Adamo, J. Jaramillo, R. Gomperts, R. E. Stratmann, O. Yazyev, A. J. Austin, R. Cammi, C. Pomelli, J. W. Ochterski, R. L. Martin, K. Morokuma, V. G. Zakrzewski, V. A. Voth, P. Salvador, J. J. Dannenberg, S. Dapprich, A. D. Daniels, Ö. Farkas, J. B. Foresman, J. V. Ortiz, J. Cioslowski and D. J. Fox, *Gaussian 09, Revision A.01*; Gaussian, Inc.: Wallingford, CT, 2009.

9 A. D. Becke, *J. Chem. Phys.*, 1993, **98**, 5648–5652.

10 C. Lee, W. Yang and R. G. Parr, *Phys. Rev. B*, 1988, **37**, 785–789.

11 S. Grimme, S. Ehrlich and L. Goerigk, *J. Comput. Chem.*, 2011, **32**, 1456–1465.

12 K. Fukui, *J. Phys. Chem.*, 1970, **74**, 4161–4163.

13 K. Fukui, *Acc. Chem. Res.*, 1981, **14**, 363–368.

14 C. Gonzalez and H. B. Schlegel, *J. Chem. Phys.*, 1989, **90**, 2154–2161.

15 C. Gonzalez and H. B. Schlegel, *J. Phys. Chem.*, 1990, **94**, 5523–5527.

16 Y. Zhao and D. G. Truhlar, *Theor. Chem. Acc.*, 2008, **120**, 215–241.

17 S. Grimme, J. Antony, S. Ehrlich and H. Krieg, *J. Chem. Phys.*, 2010, **132**, 154104.

18 A. V. Marenich, C. J. Cramer and D. G. Truhlar, *J. Phys. Chem. B,* 2009, **113**, 6378–6396.

19 E. Runge and E. K. U. Gross, *Phys. Rev. Lett.*, 1984, **52**, 997–1000.

20 R. Bauernschmitt and R. Ahlrichs, *Chem. Phys. Lett.*, 1996, **256**, 454–464.

21 R. E. Stratmann, G. E. Scuseria and M. J. Frisch, *J. Chem. Phys.*, 1998, **109**, 8218–8224.

22 C. Y. Legault, *CYLview, Version 1.0b*; Université de Sherbrooke: Sherbrooke, Quebec, Canada, 2009.

5 NMR Spectra of New Compound

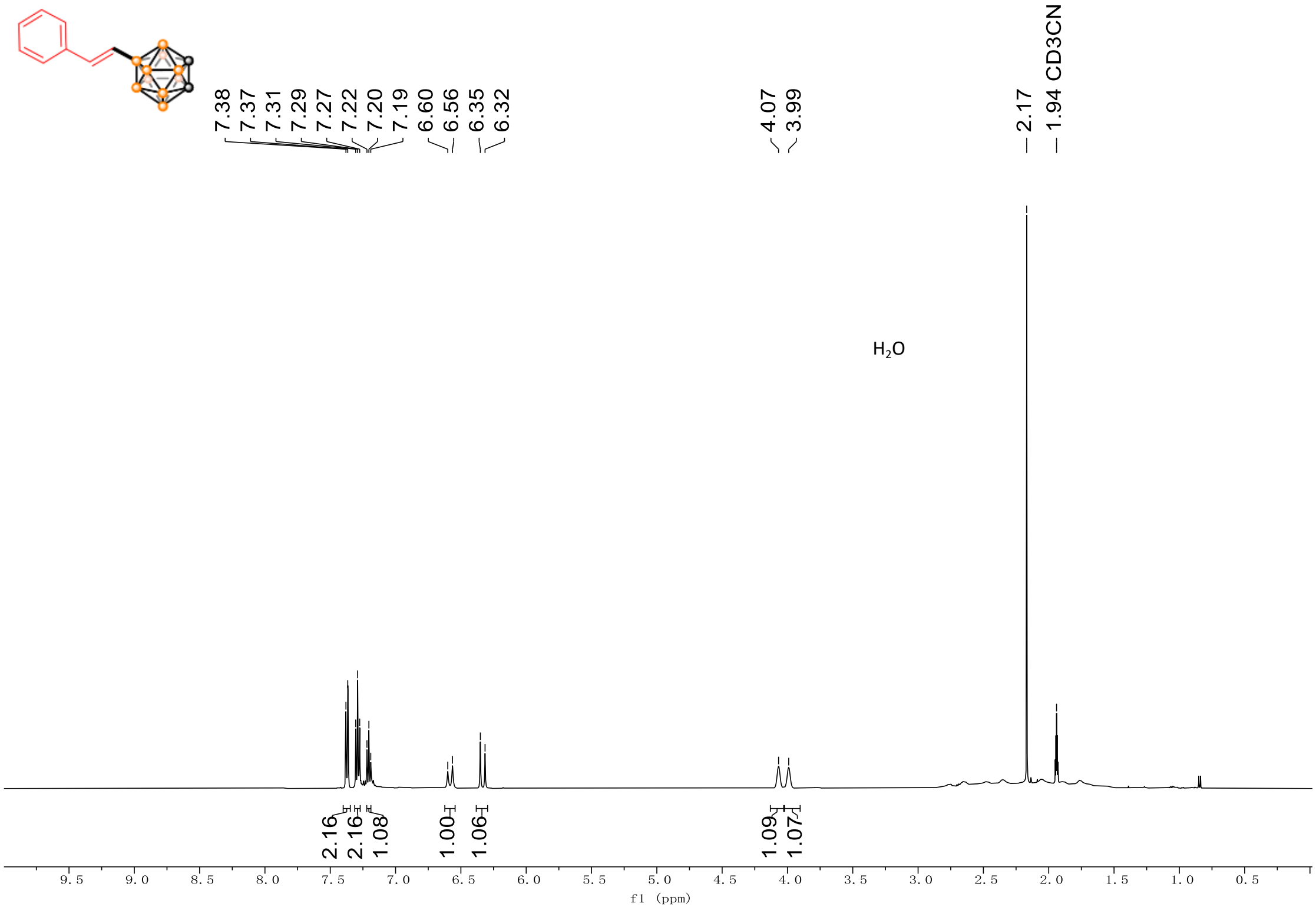

^{1}H NMR (500 MHz, CD_3CN) of **3aa**

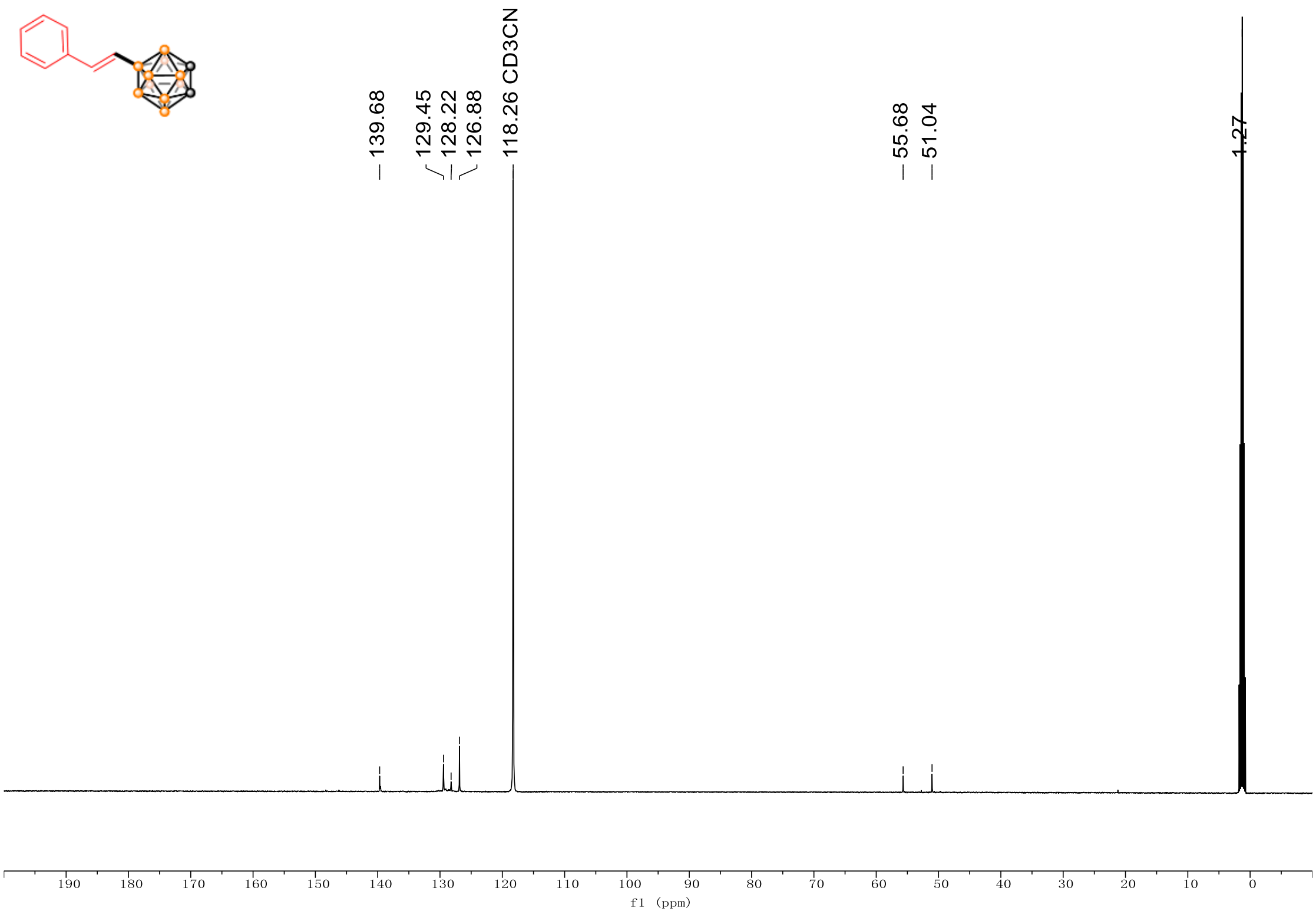

$^{13}C\{^{1}H\}$ NMR (125 MHz, CD_3CN) of **3aa**

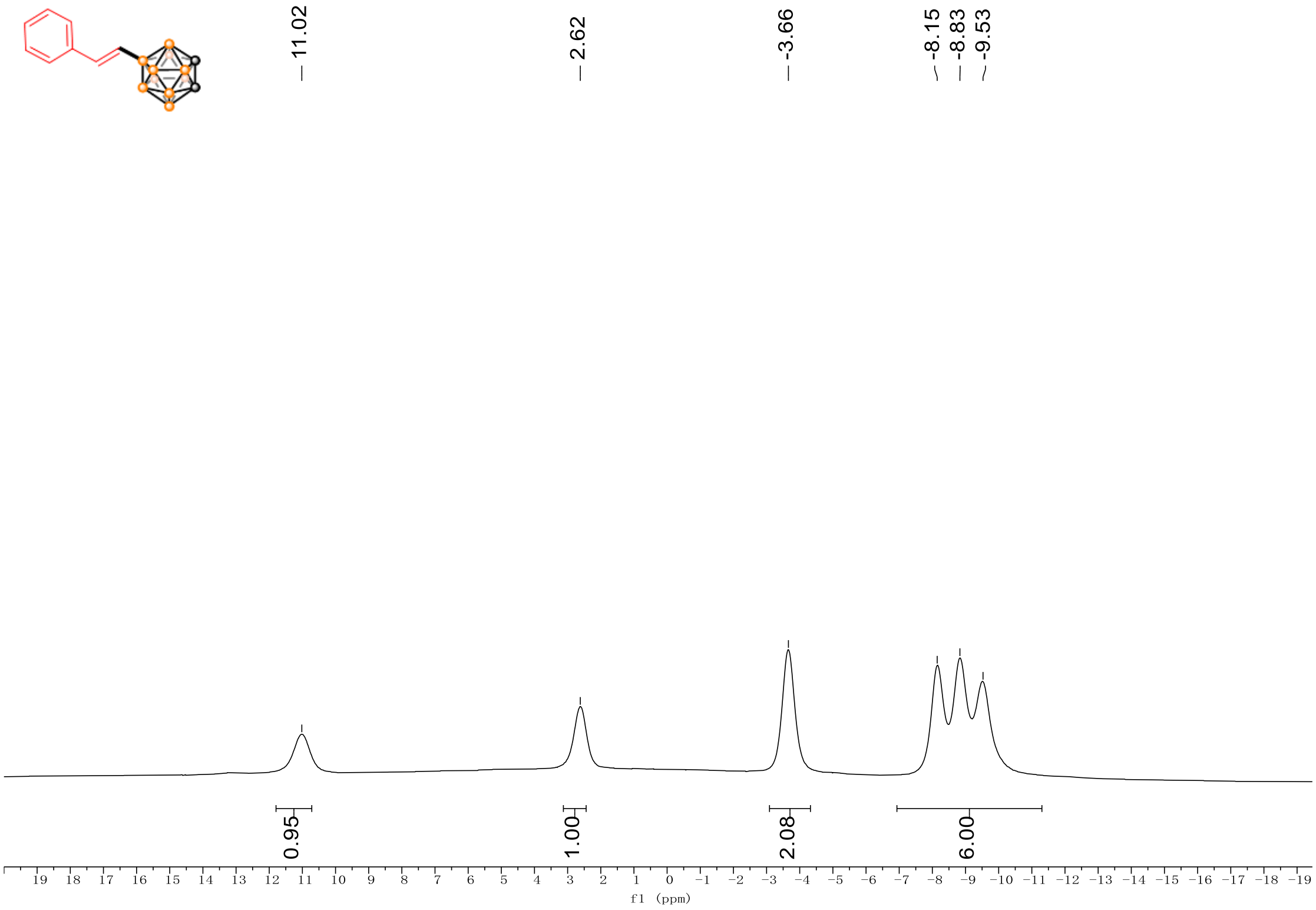

$^{11}B\{^{1}H\}$ NMR (160 MHz, CD_3CN) of **3aa**

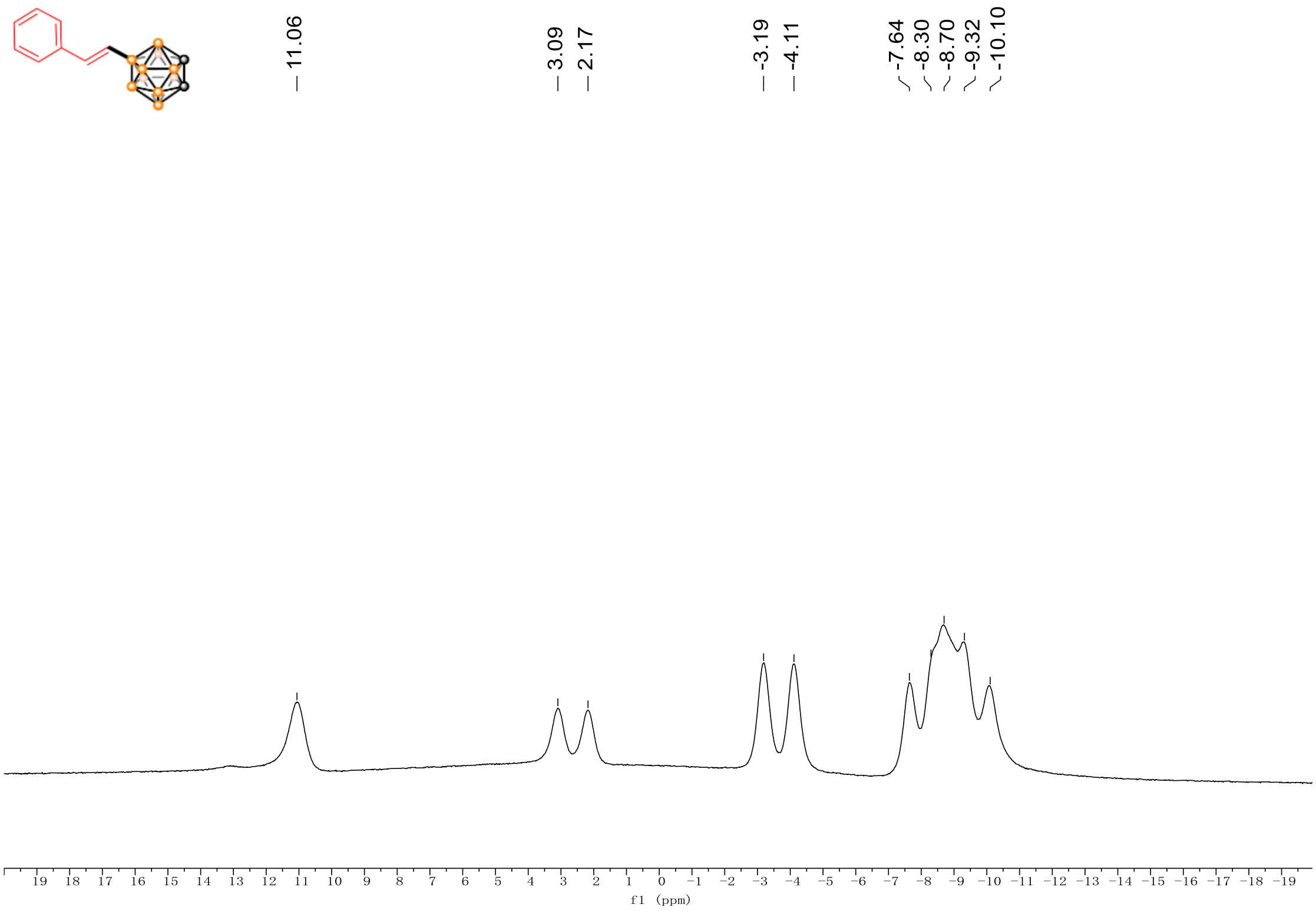

^{11}B NMR (160 MHz, CD_3CN) of **3aa**

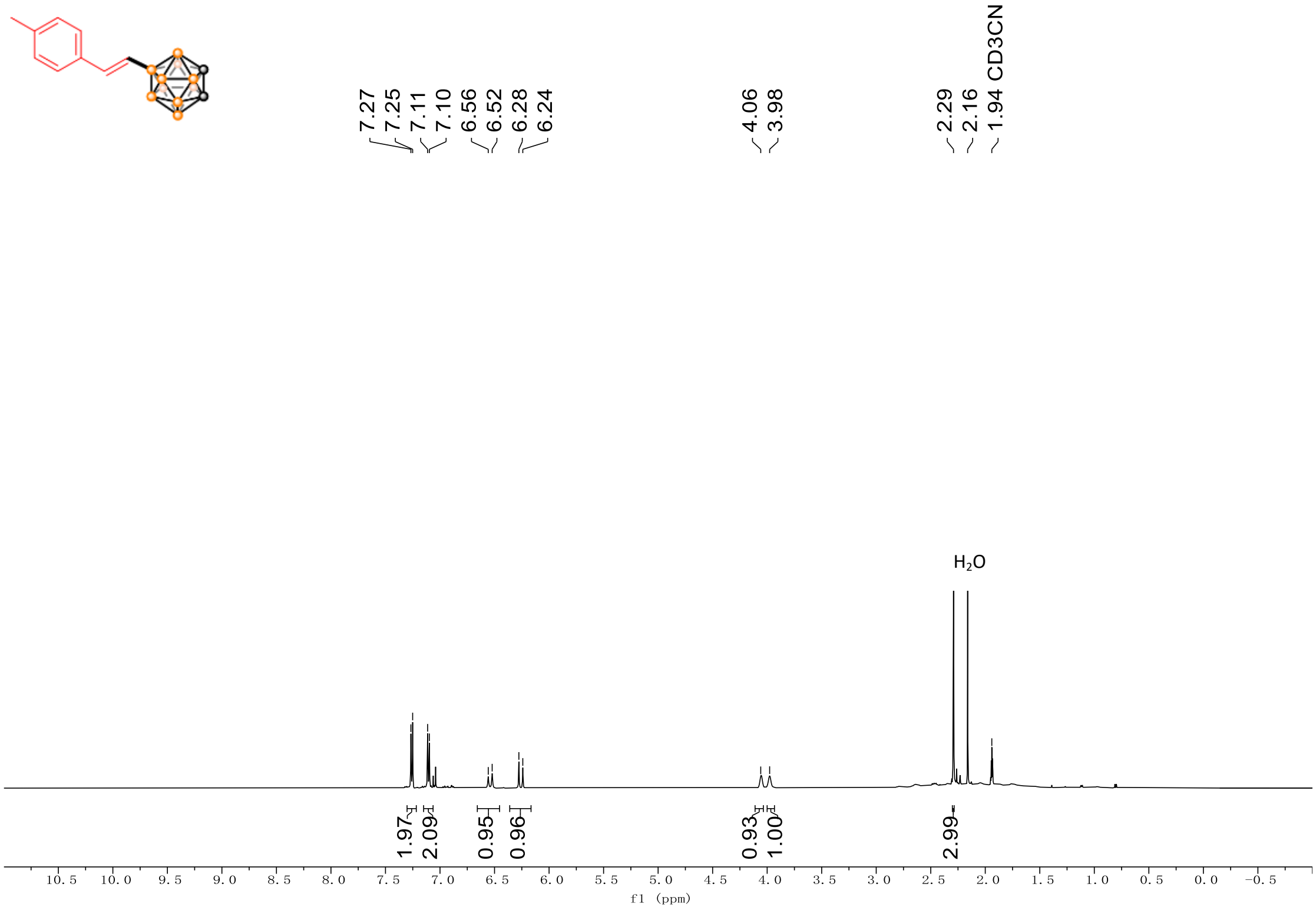

^{1}H NMR (500 MHz, CD_3CN) of **3ab**

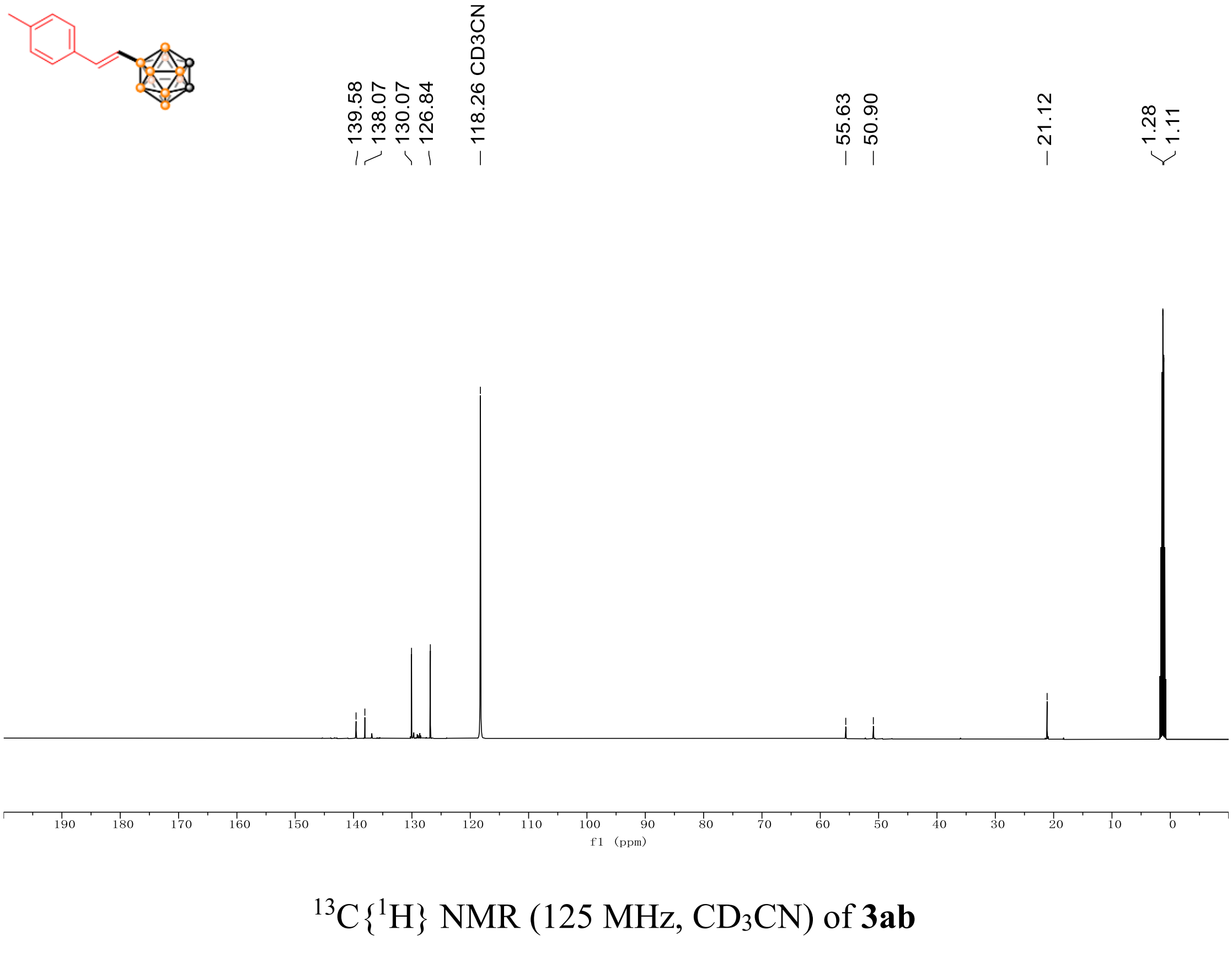

$^{13}C\{^1H\}$ NMR (125 MHz, CD_3CN) of **3ab**

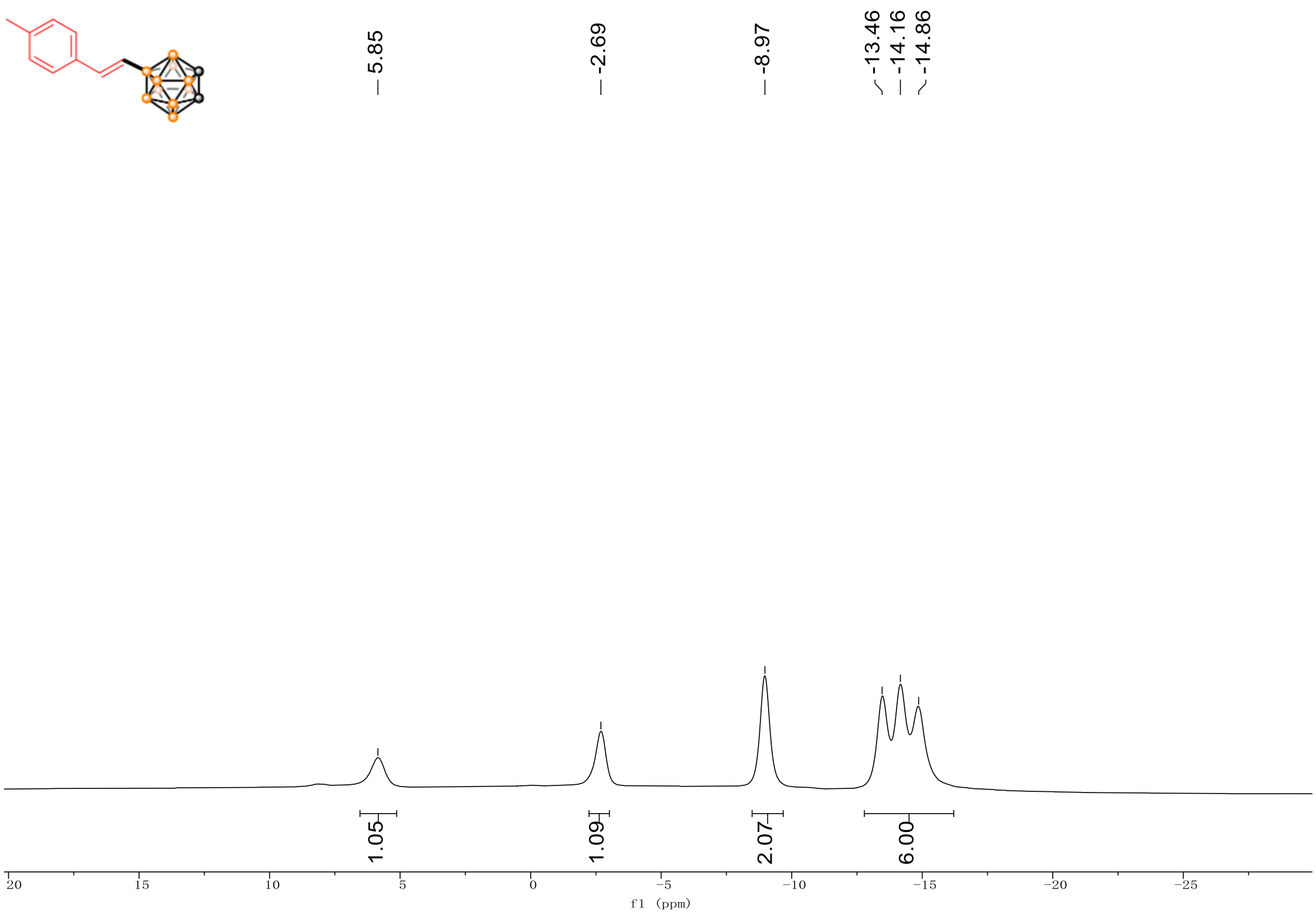

$^{11}B\{^1H\}$ NMR (160 MHz, CD_3CN) of **3ab**

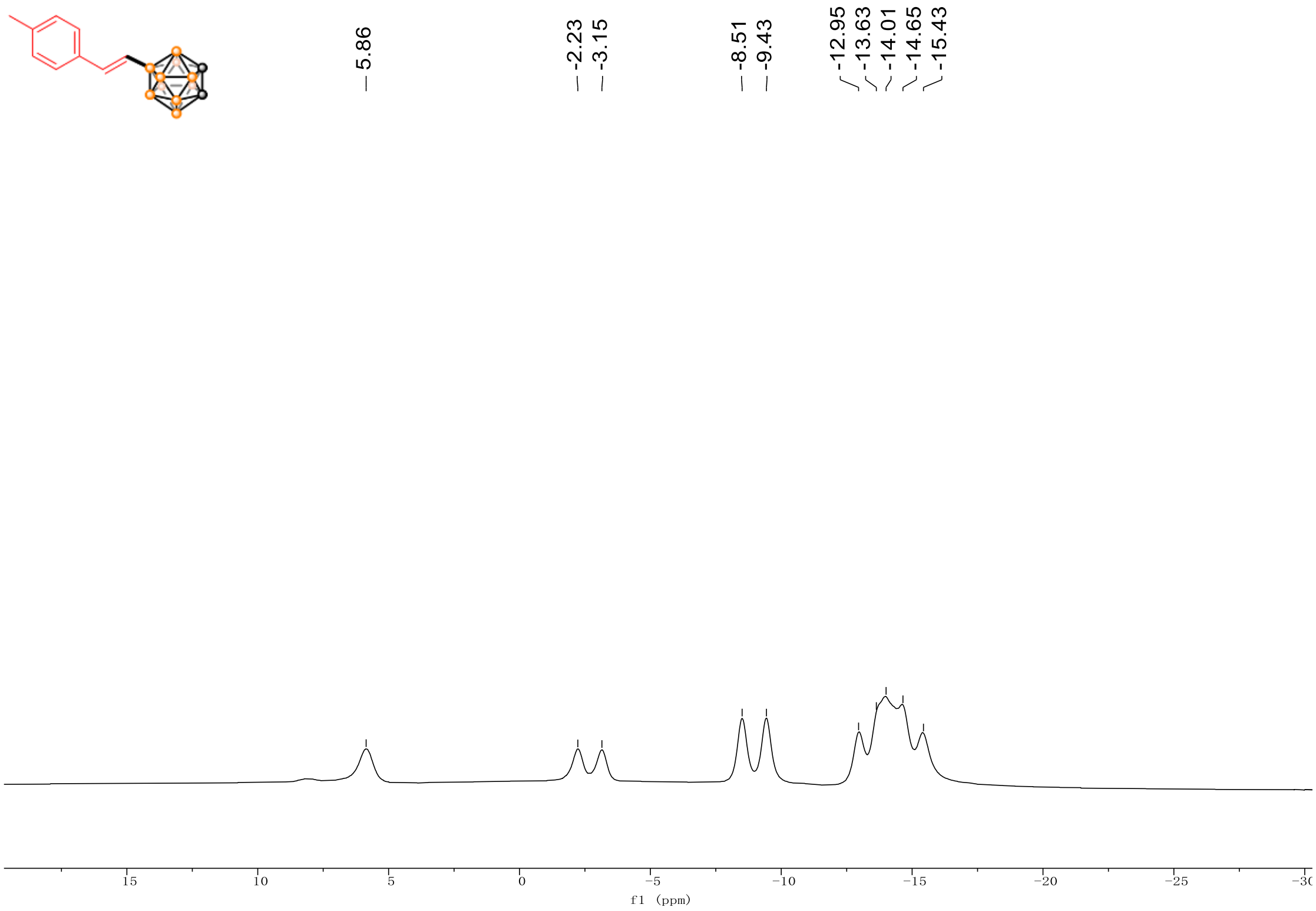

^{11}B NMR (160 MHz, CD_3CN) of **3ab**

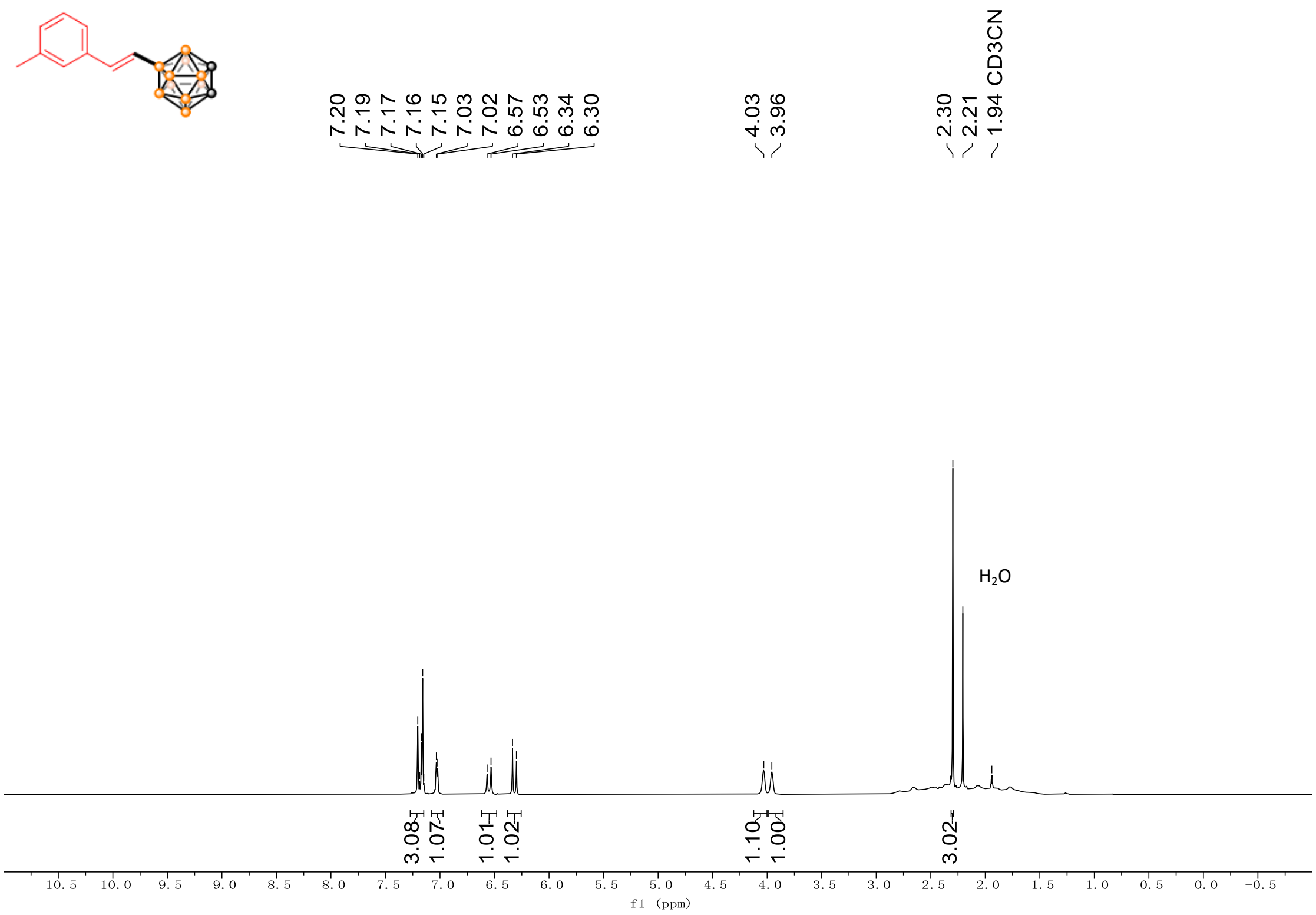

^{1}H NMR (500 MHz, CD_3CN) of **3ac**

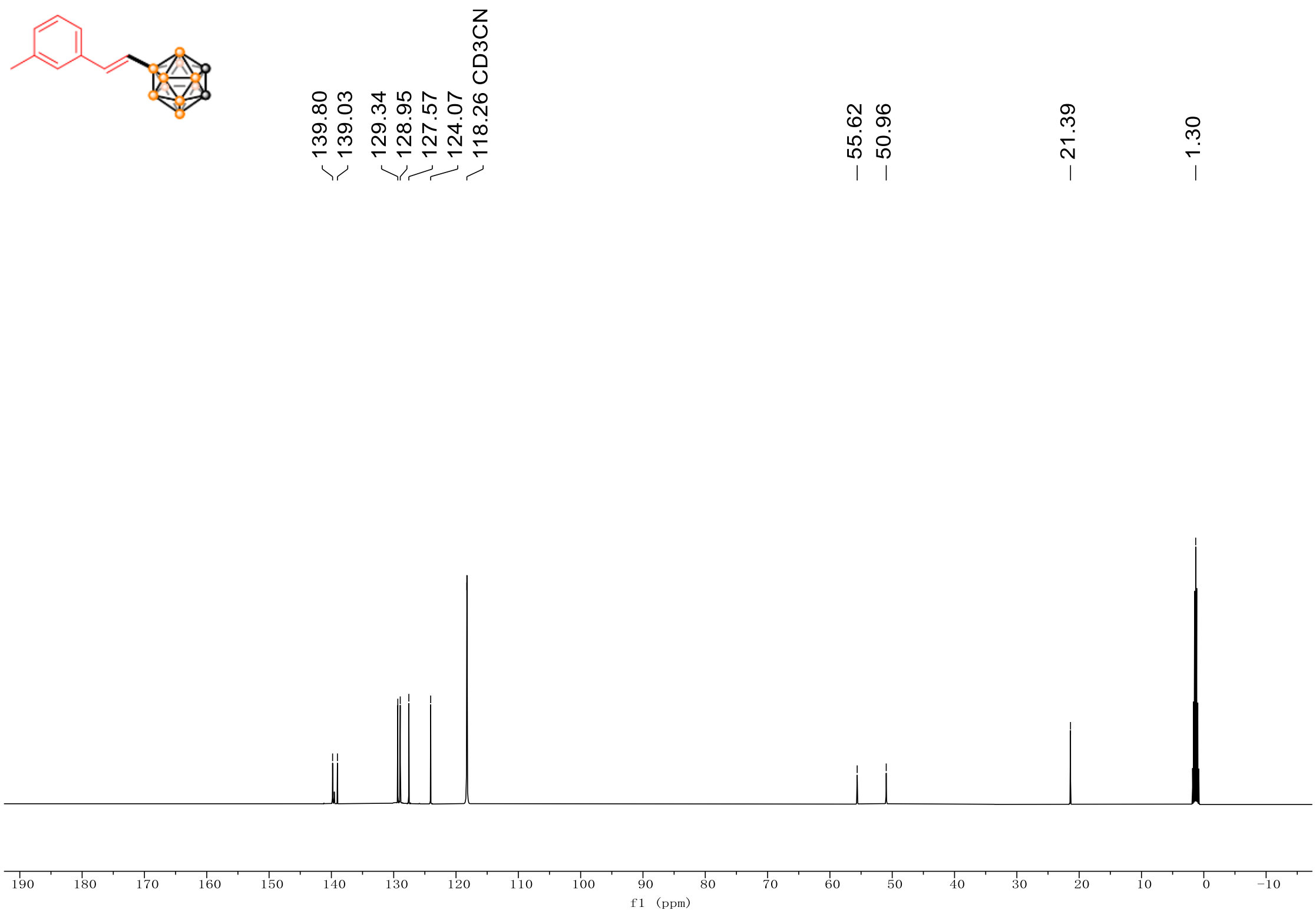

$^{13}C\{^{1}H\}$ NMR (125 MHz, CD_3CN) of **3ac**

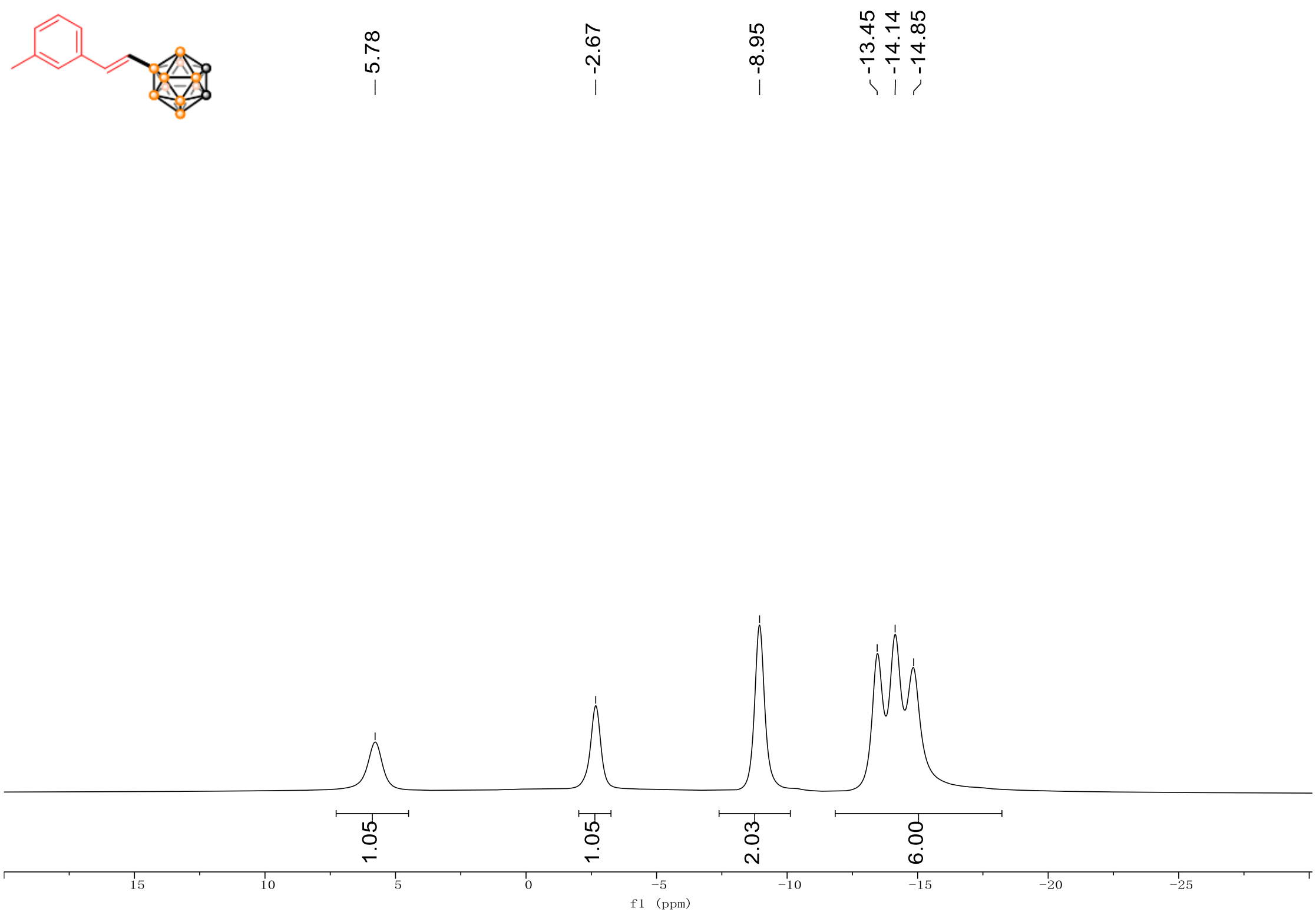

$^{11}B\{^{1}H\}$ NMR (160 MHz, CD_3CN) of **3ac**

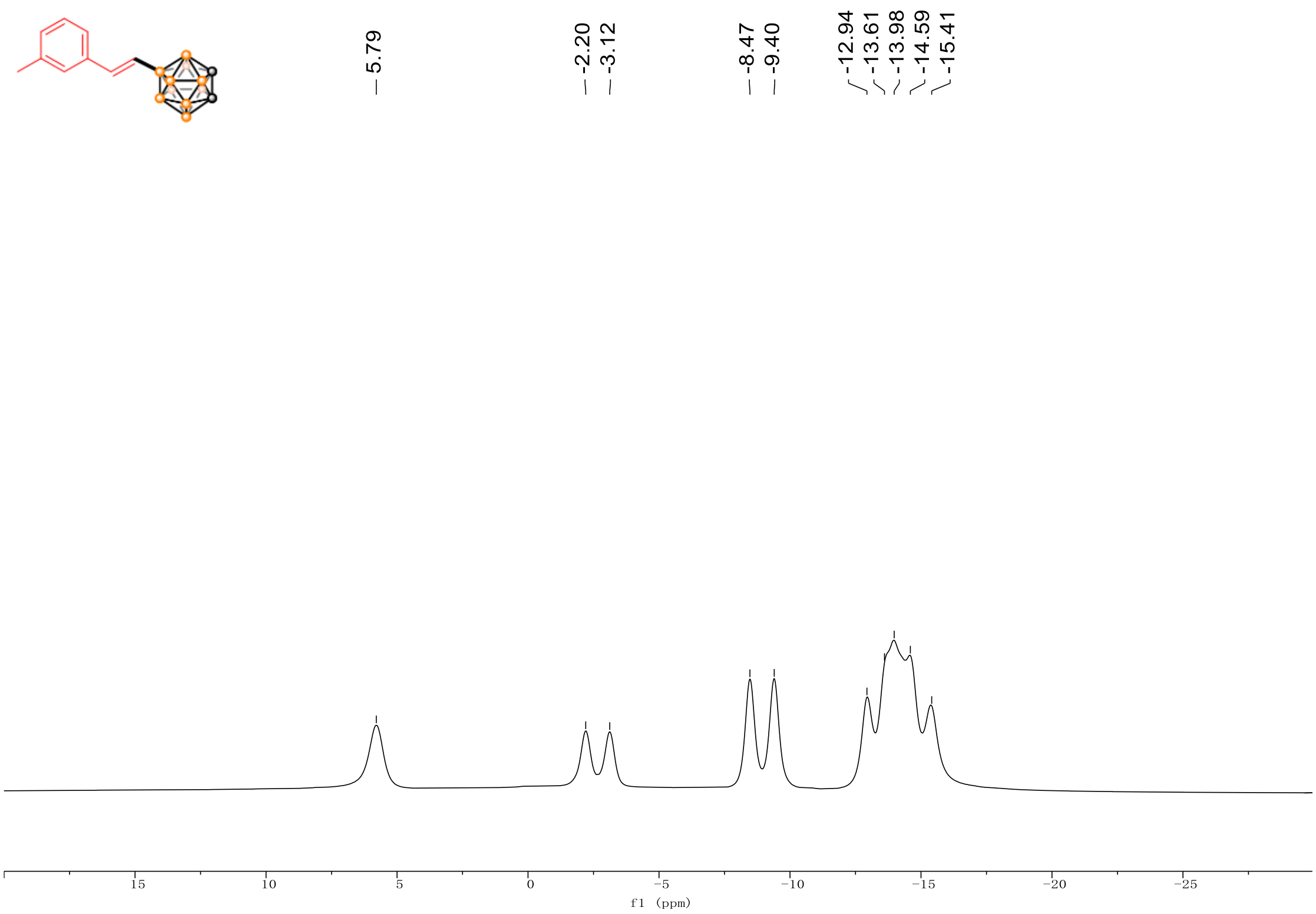

^{11}B NMR (160 MHz, CD_3CN) of **3ac**

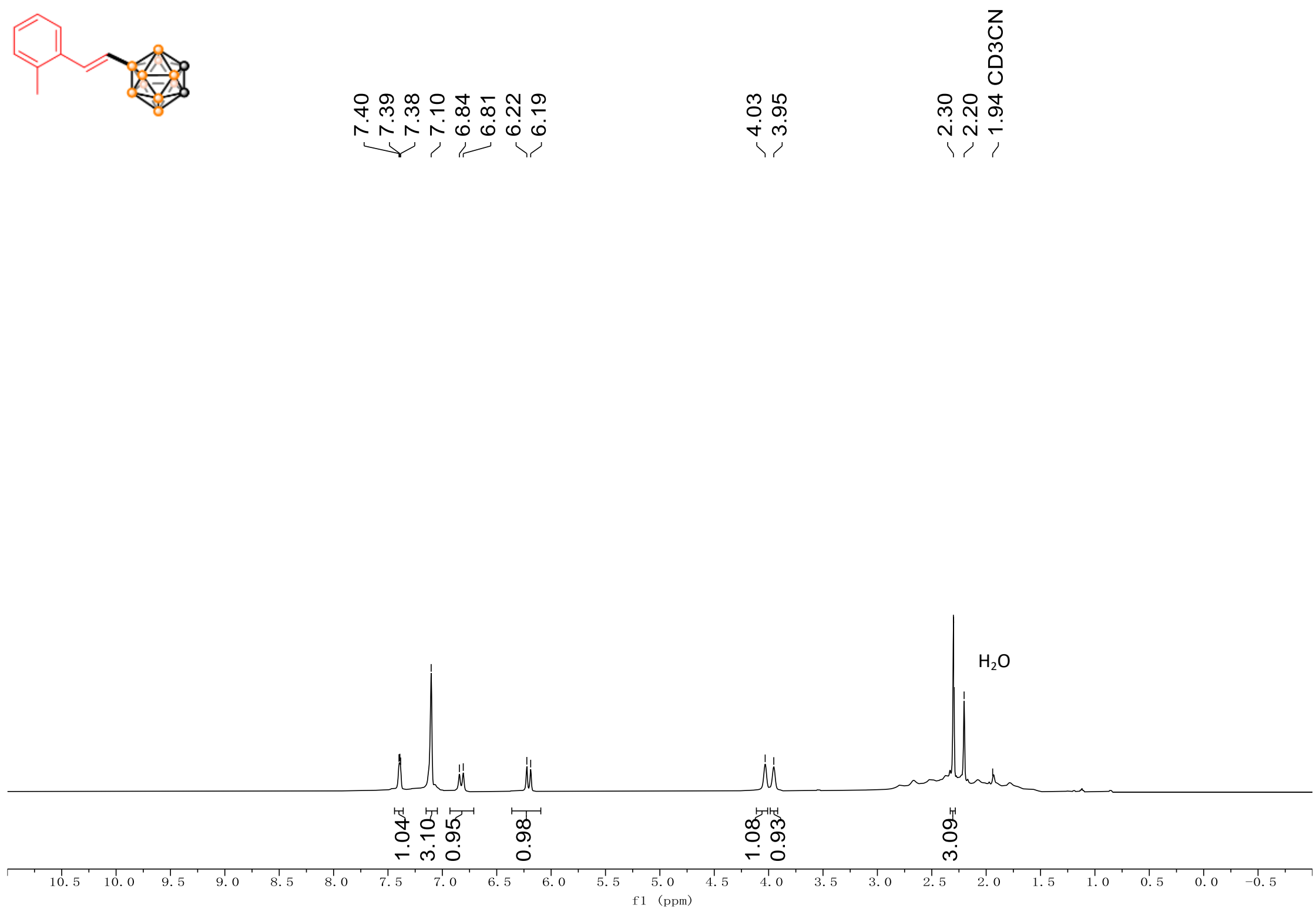

^{1}H NMR (500 MHz, CD_3CN) of **3ad**

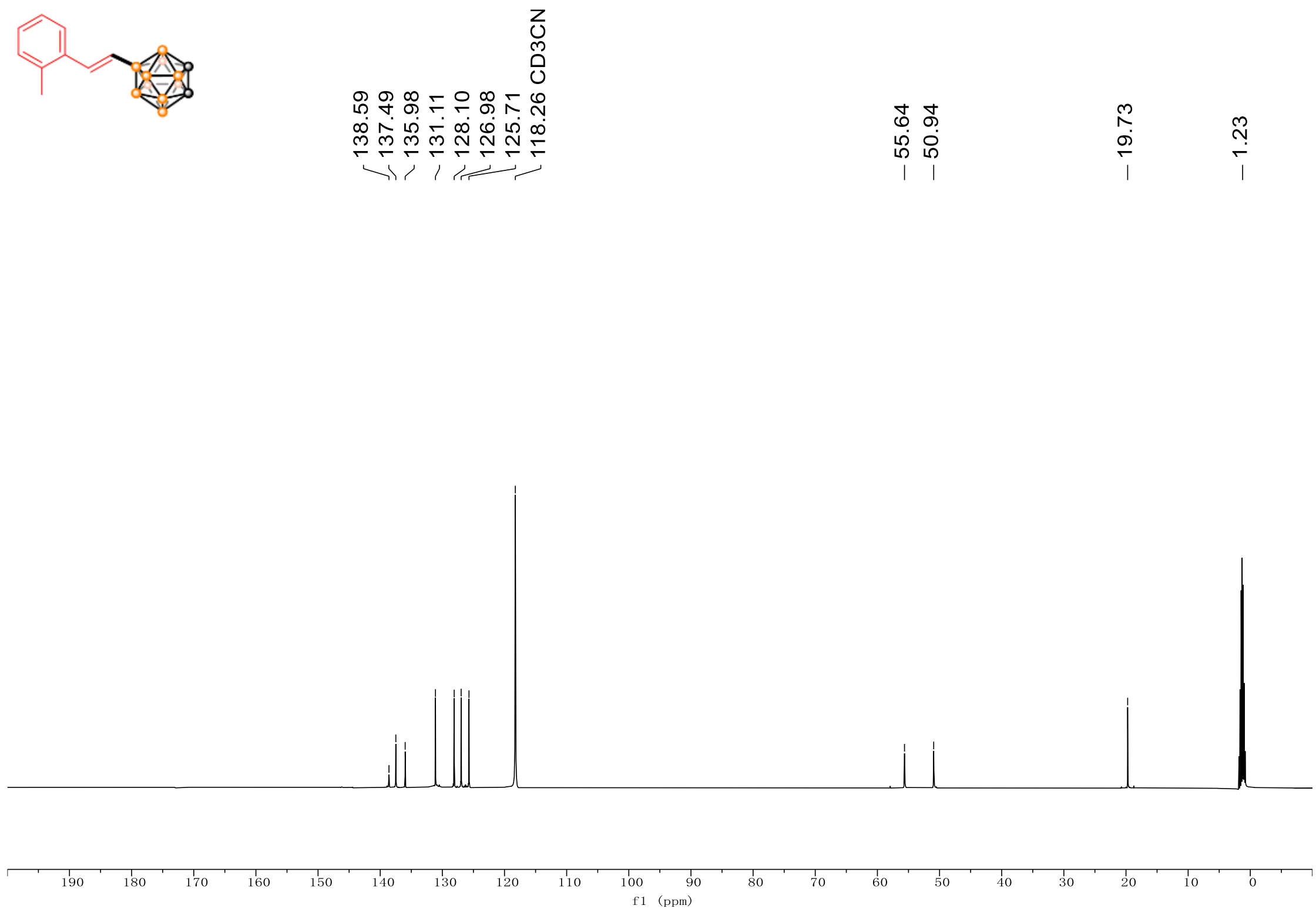

$^{13}C\{^{1}H\}$ NMR (125 MHz, CD_3CN) of **3ad**

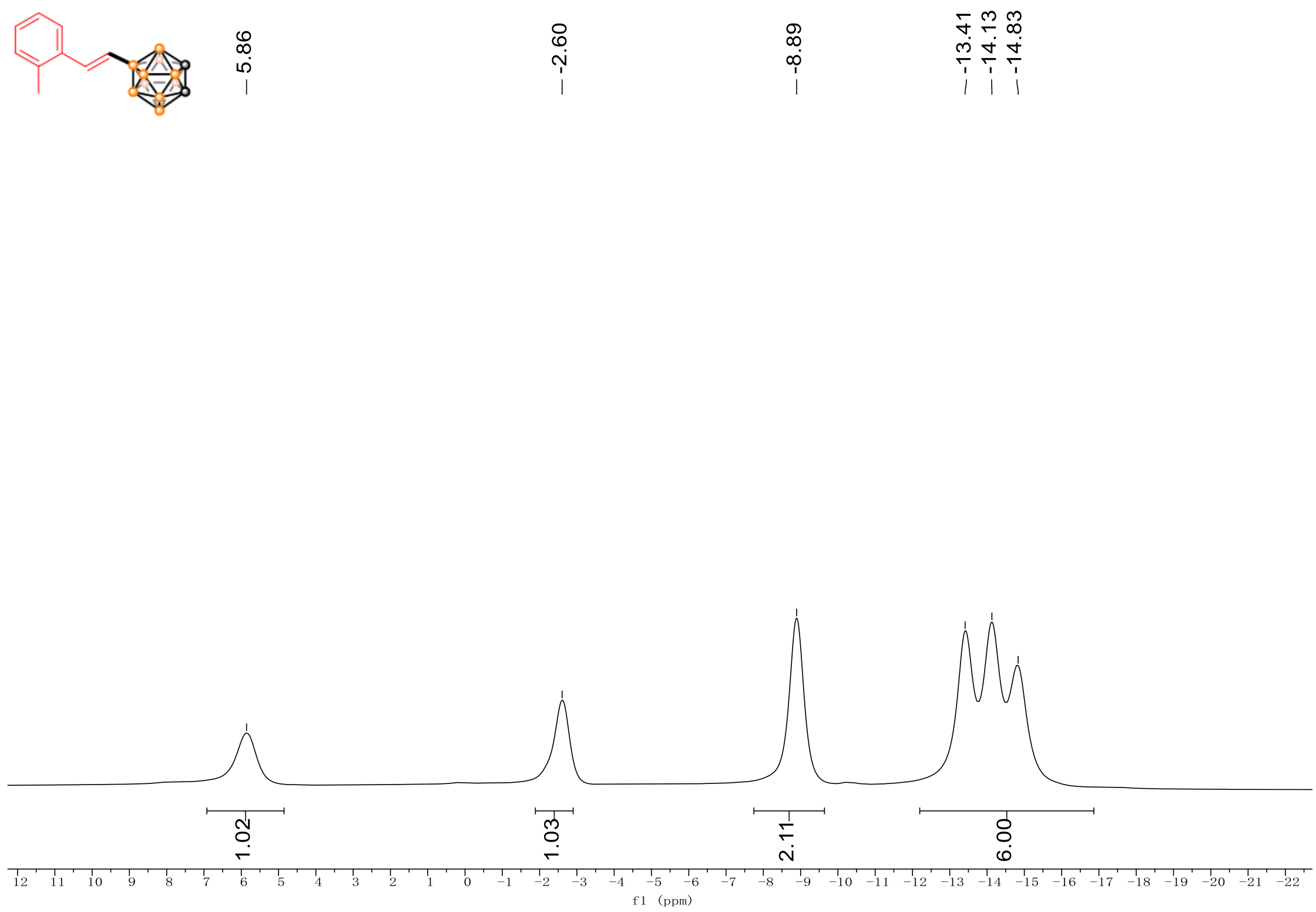

$^{11}B\{^{1}H\}$ NMR (160 MHz, CD_3CN) of **3ad**

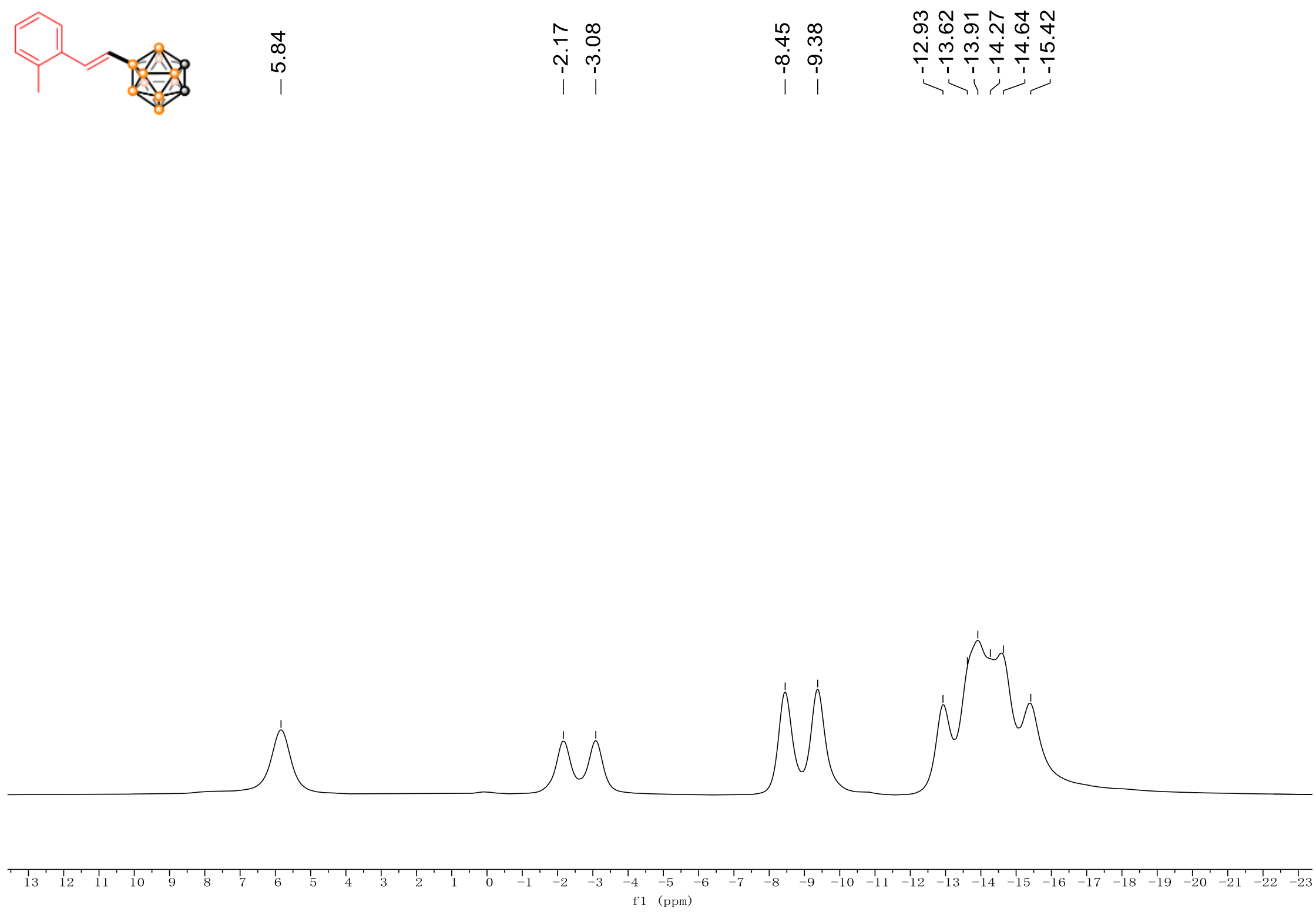

^{11}B NMR (160 MHz, CD_3CN) of **3ad**

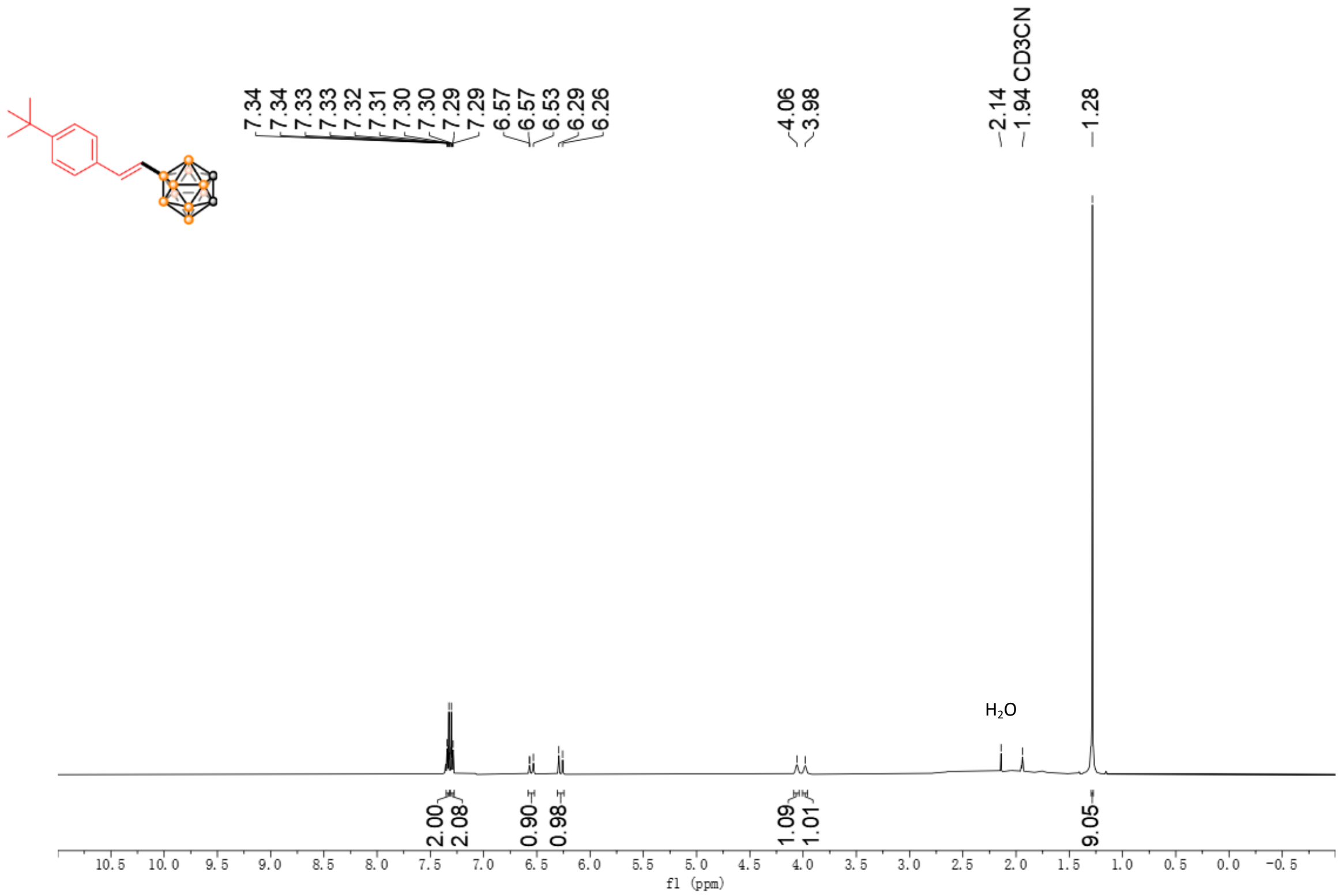

^{1}H NMR (500 MHz, CD_3CN) of **3ae**

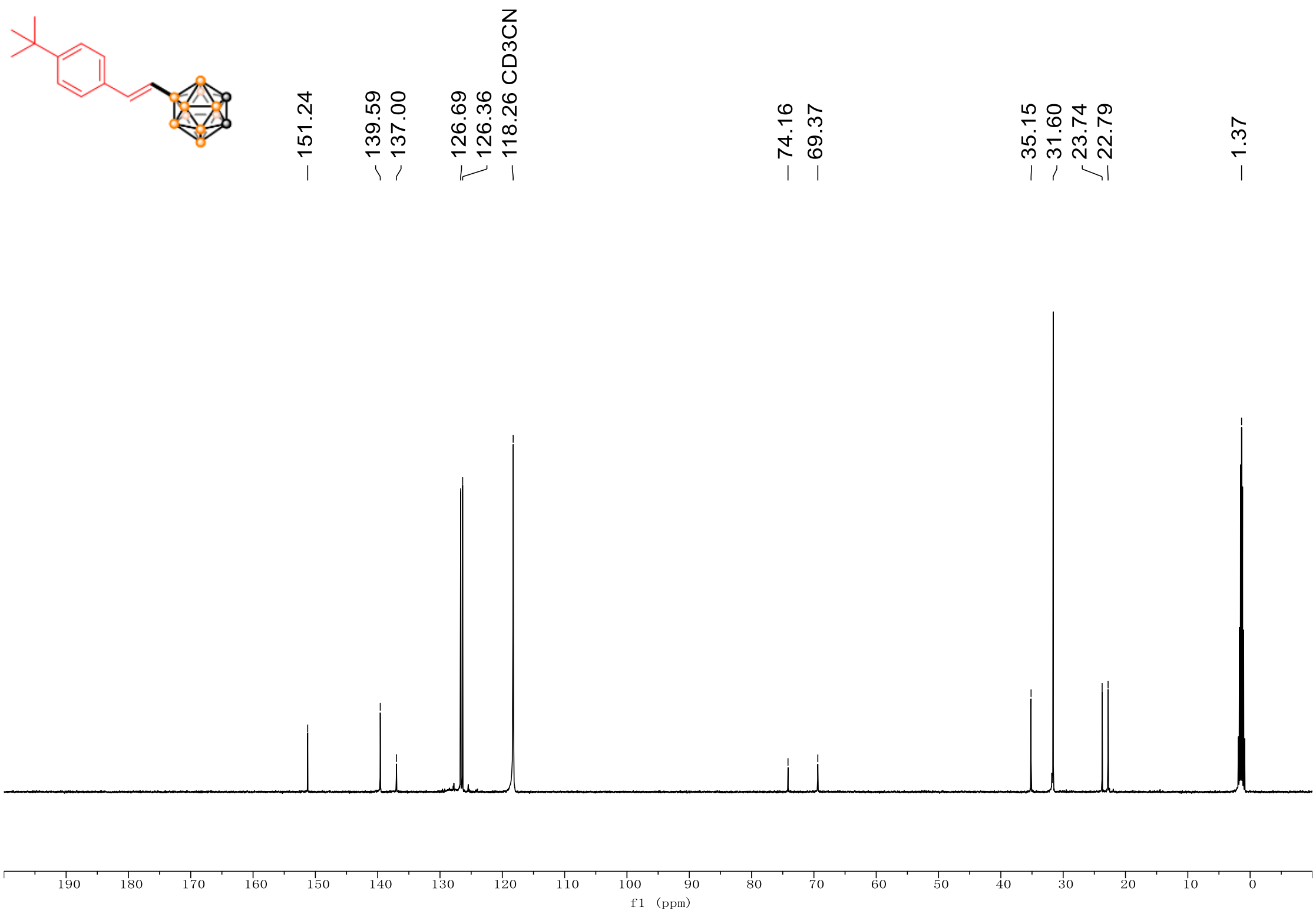

$^{13}C\{^{1}H\}$ NMR (125 MHz, CD_3CN) of **3ae**

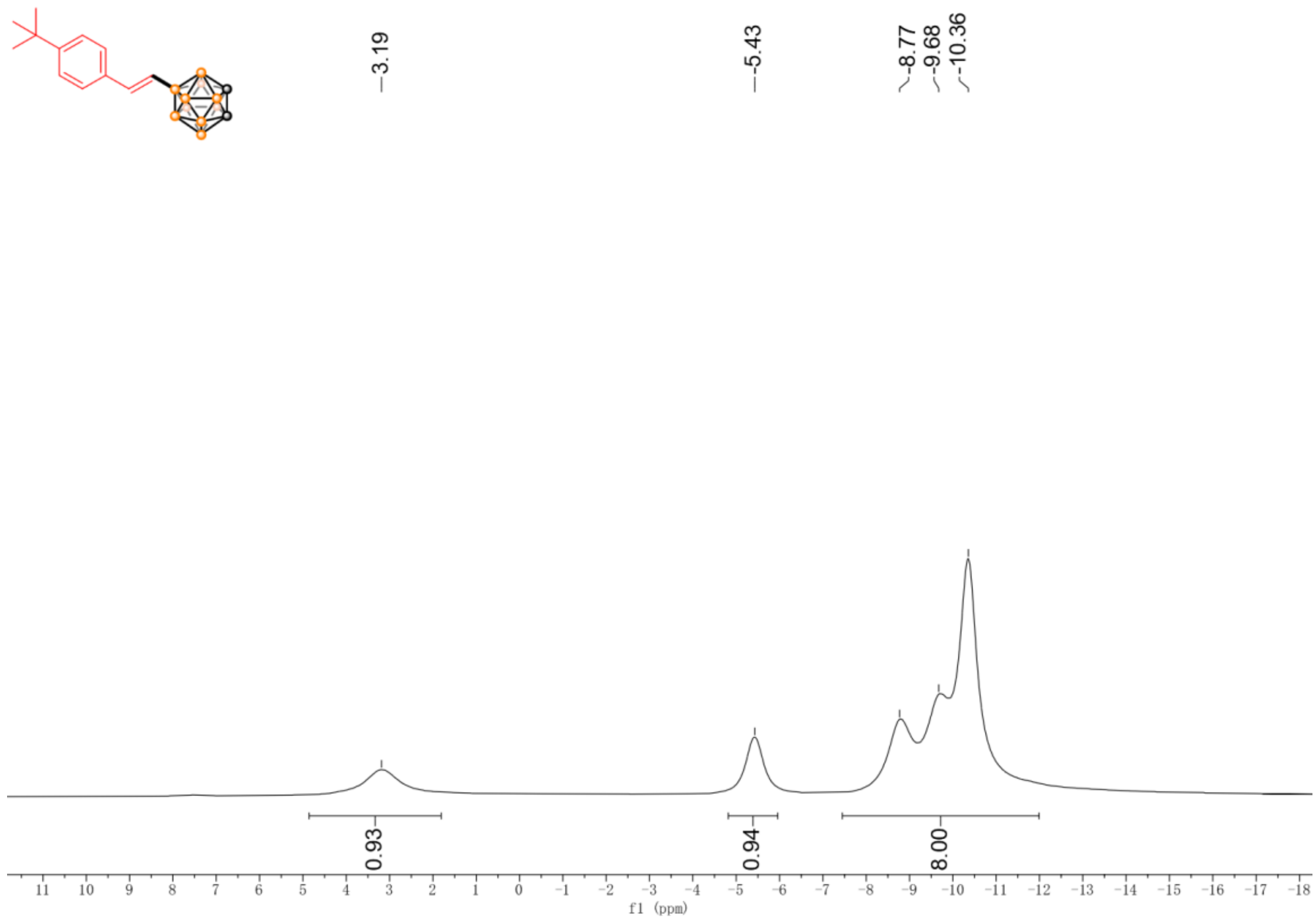

$^{11}B\{^{1}H\}$ NMR (160 MHz, CD_3CN) of **3ae**

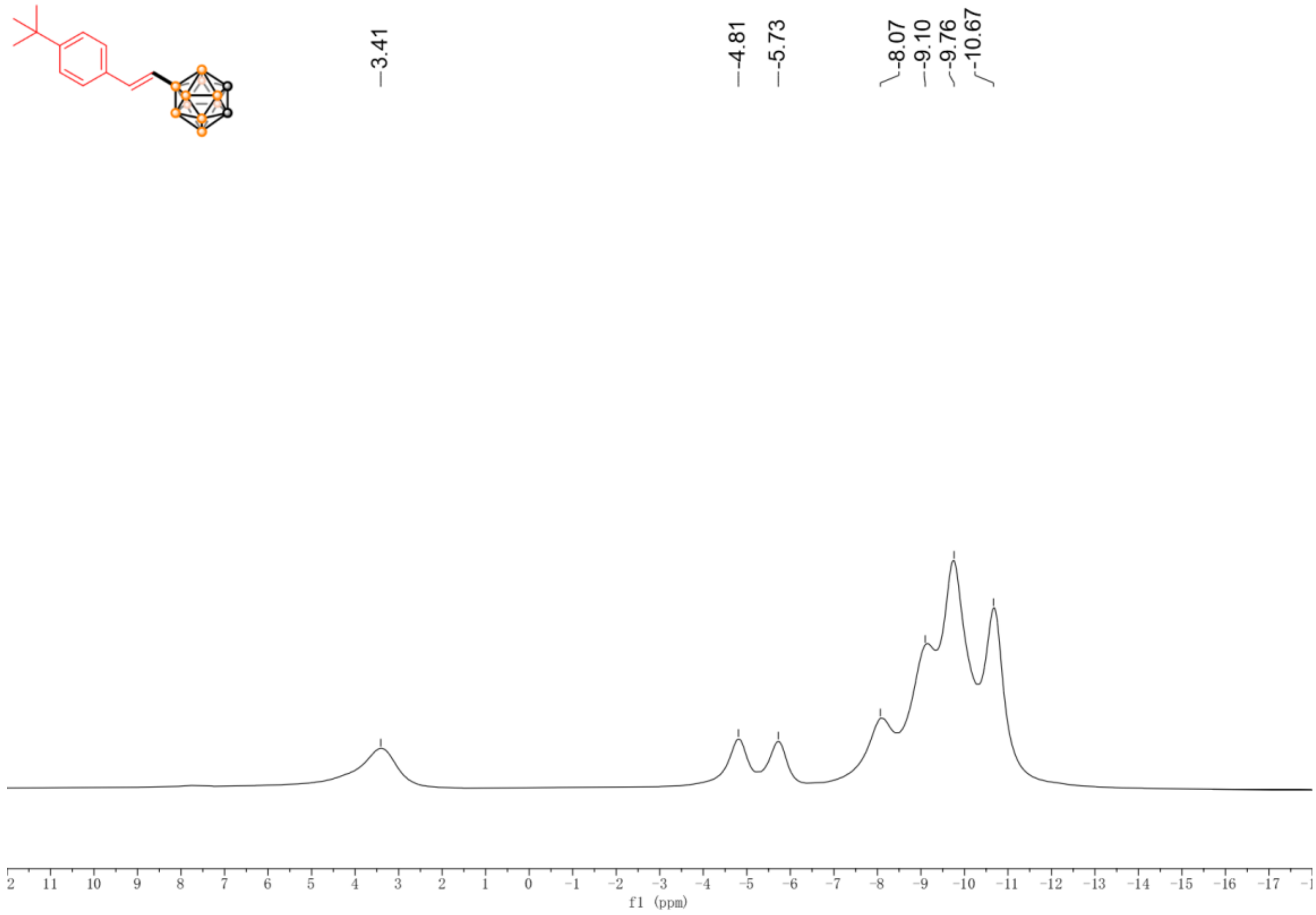

^{11}B NMR (160 MHz, CD_3CN) of **3ae**

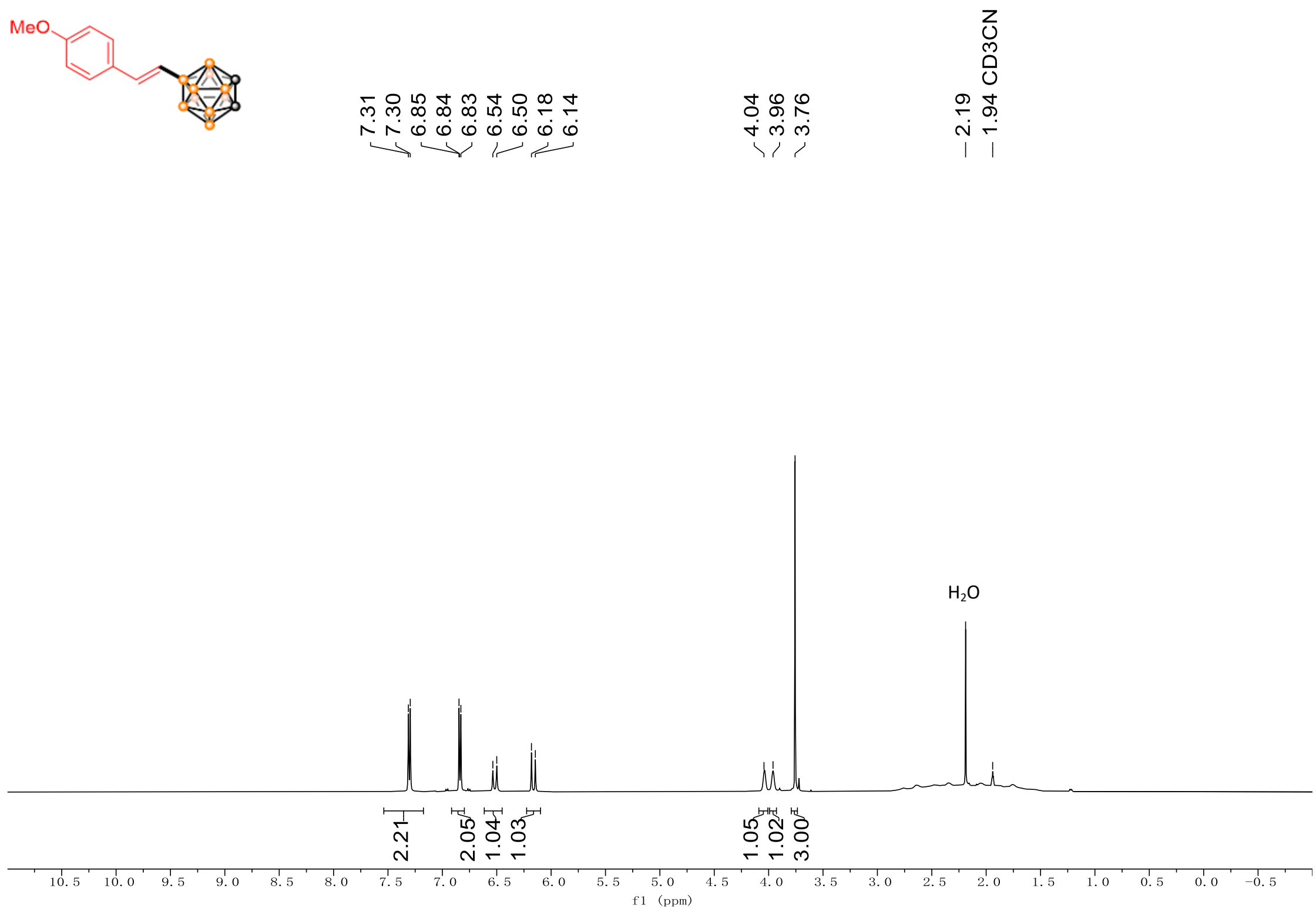

^{1}H NMR (500 MHz, CD_3CN) of **3af**

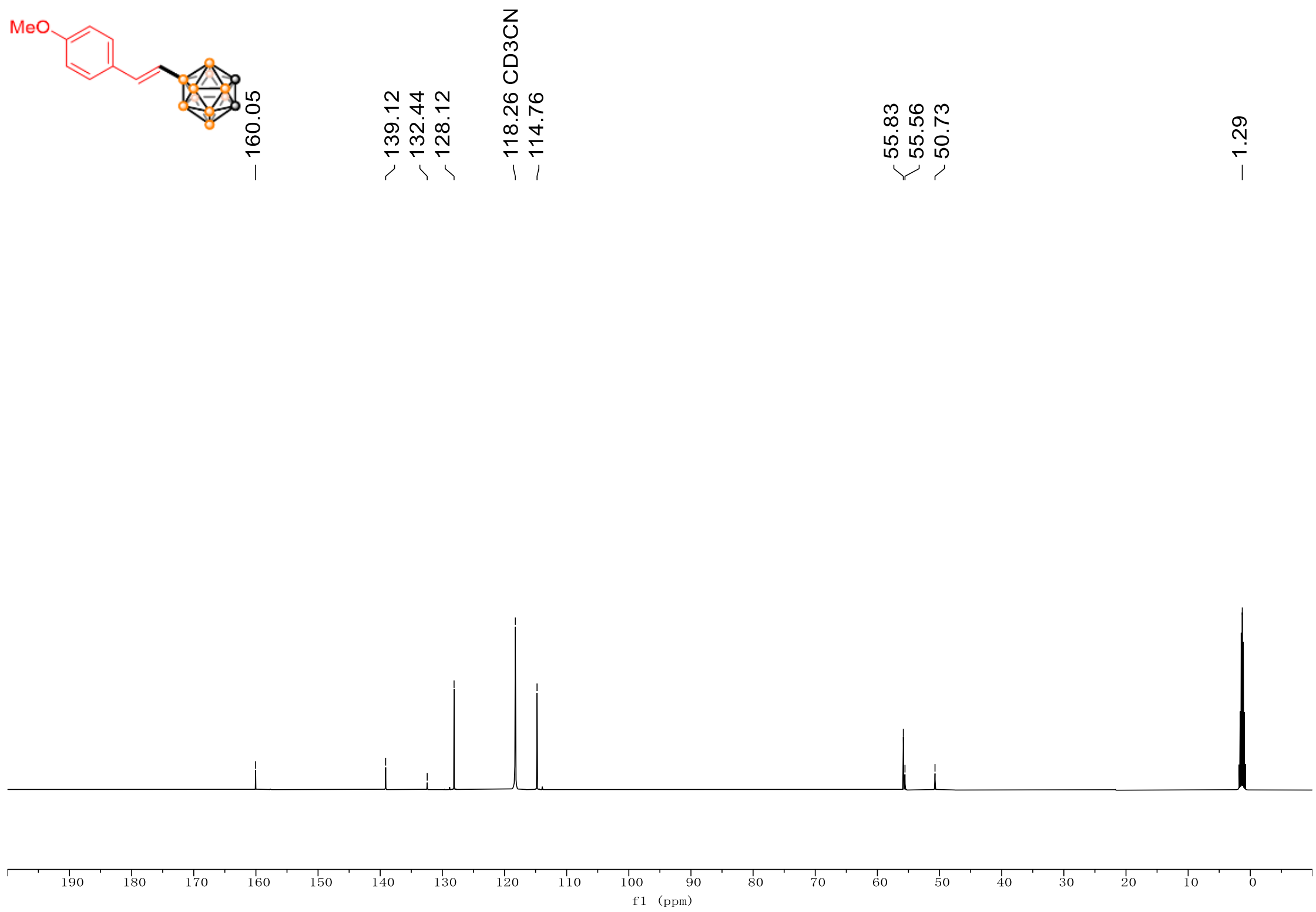

$^{13}C\{^{1}H\}$ NMR (125 MHz, CD_3CN) of **3af**

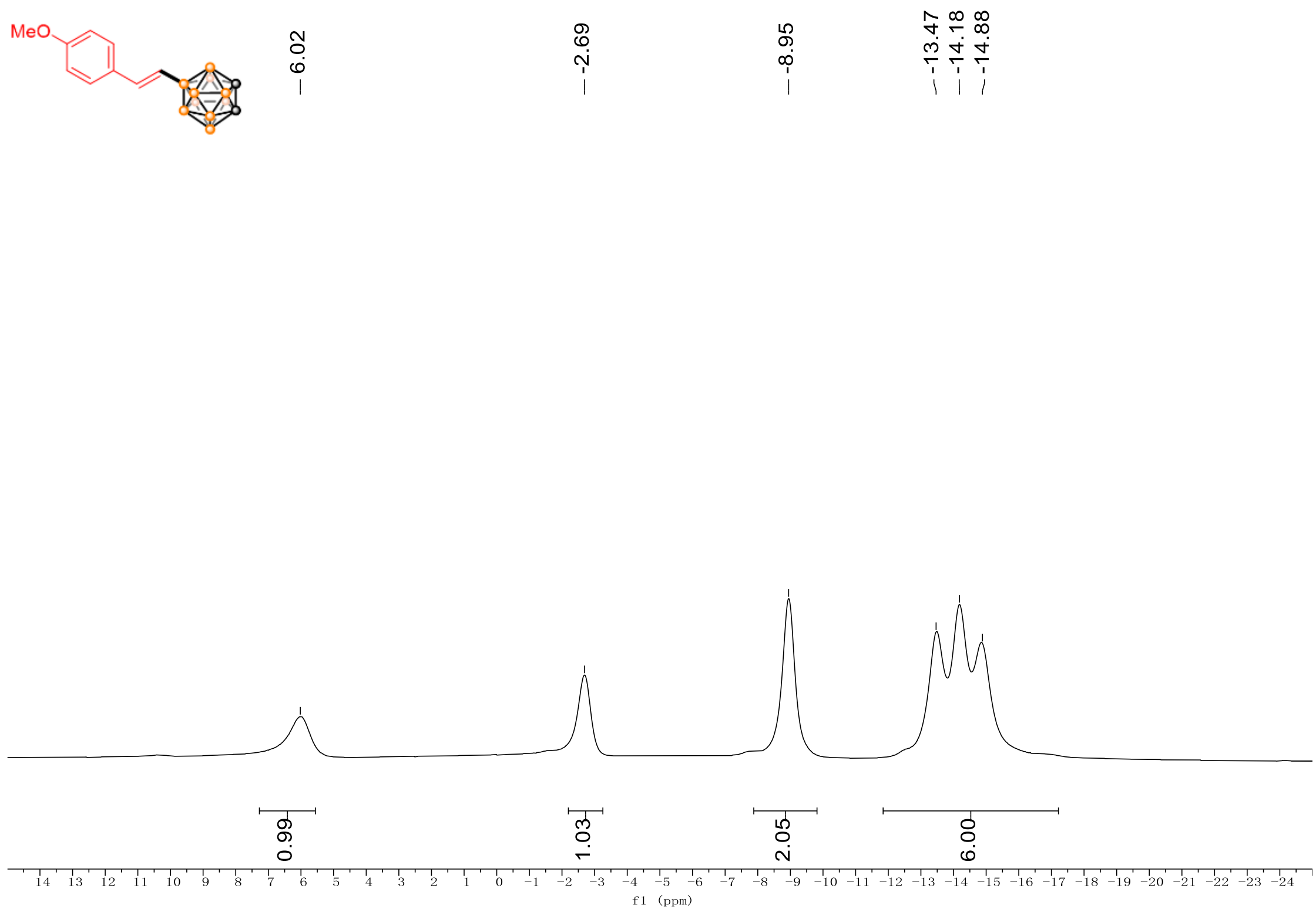

$^{11}B\{^{1}H\}$ NMR (160 MHz, CD_3CN) of **3af**

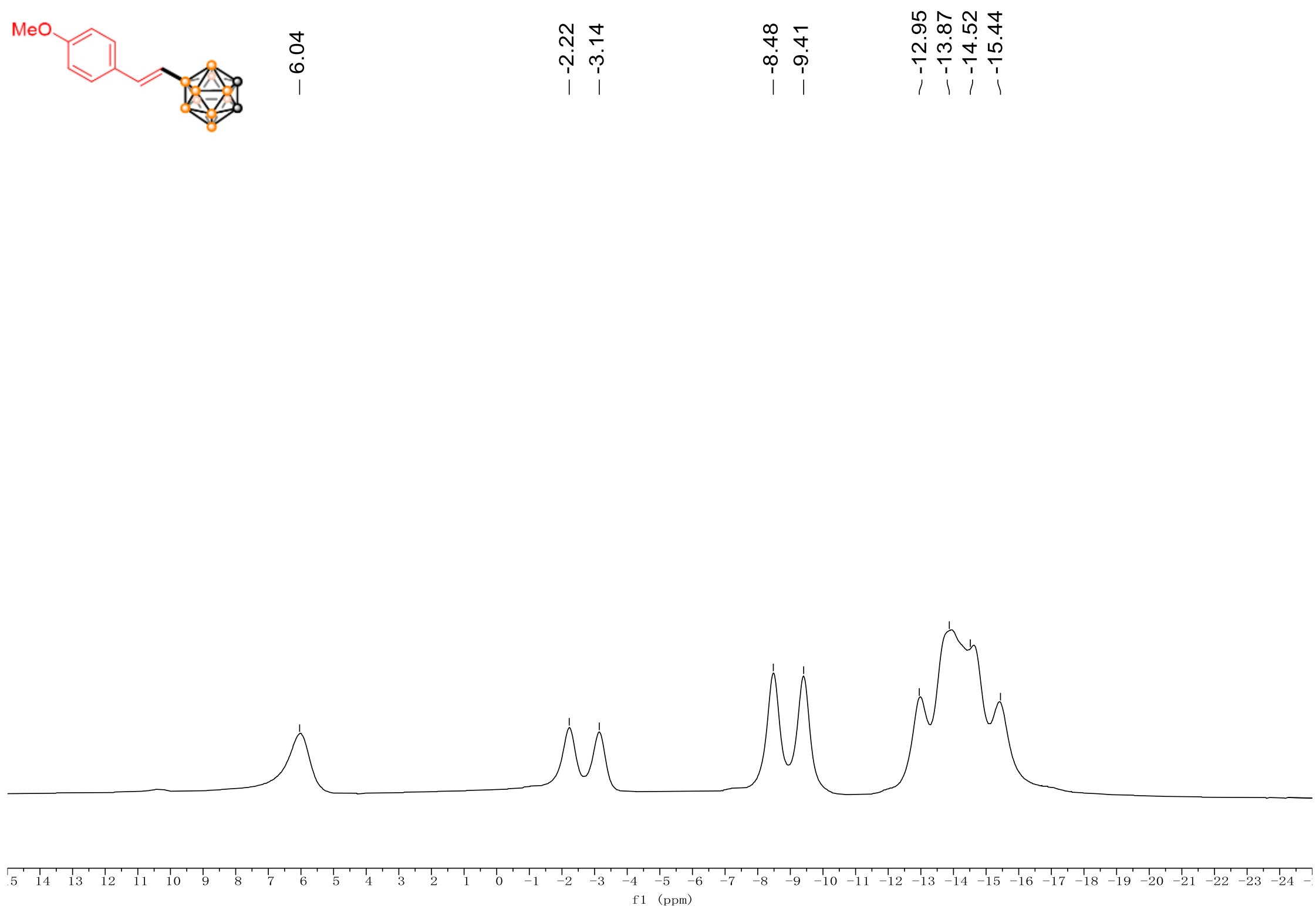

^{11}B NMR (160 MHz, CD_3CN) of **3af**

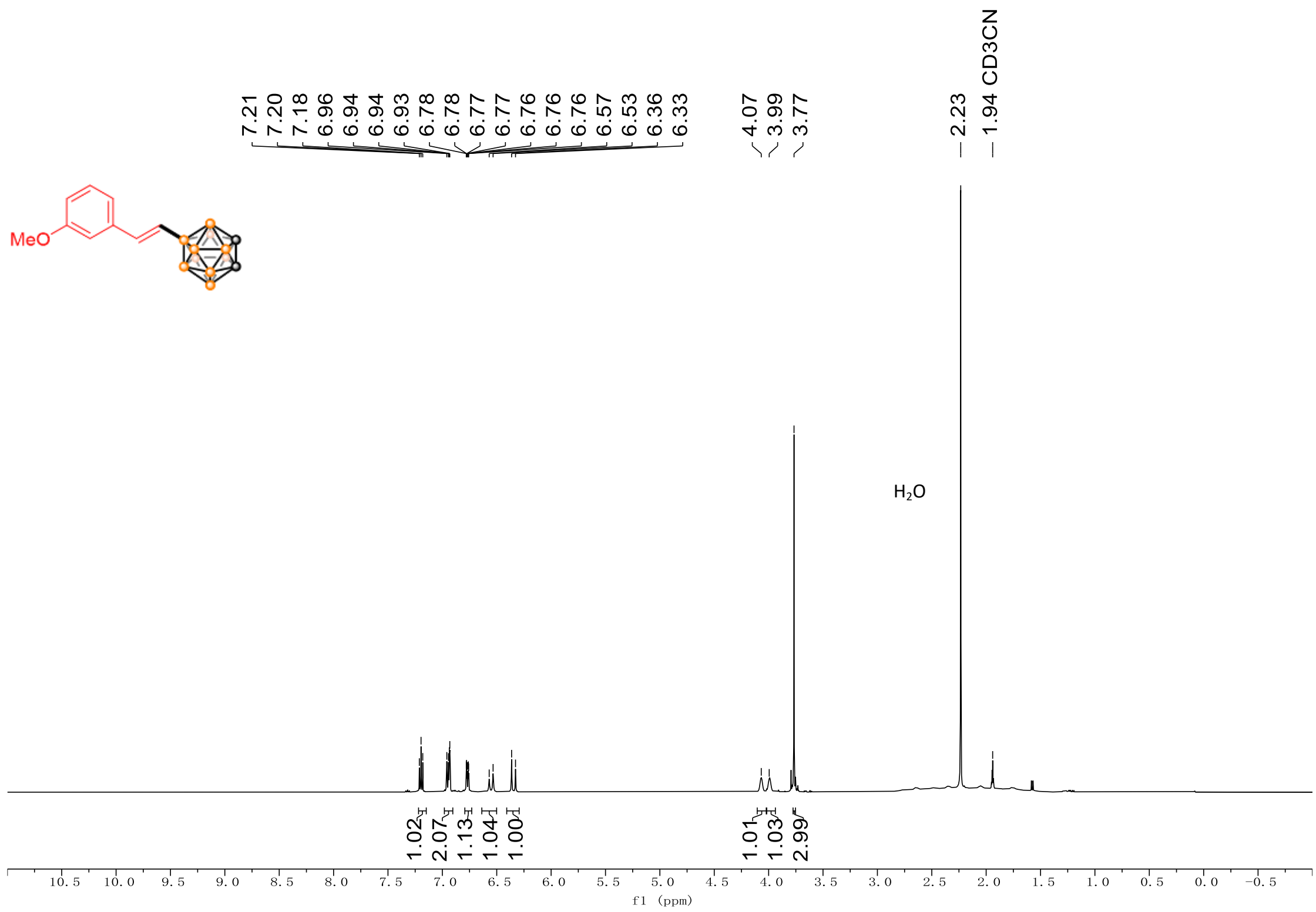

^{1}H NMR (500 MHz, CD_3CN) of **3ag**

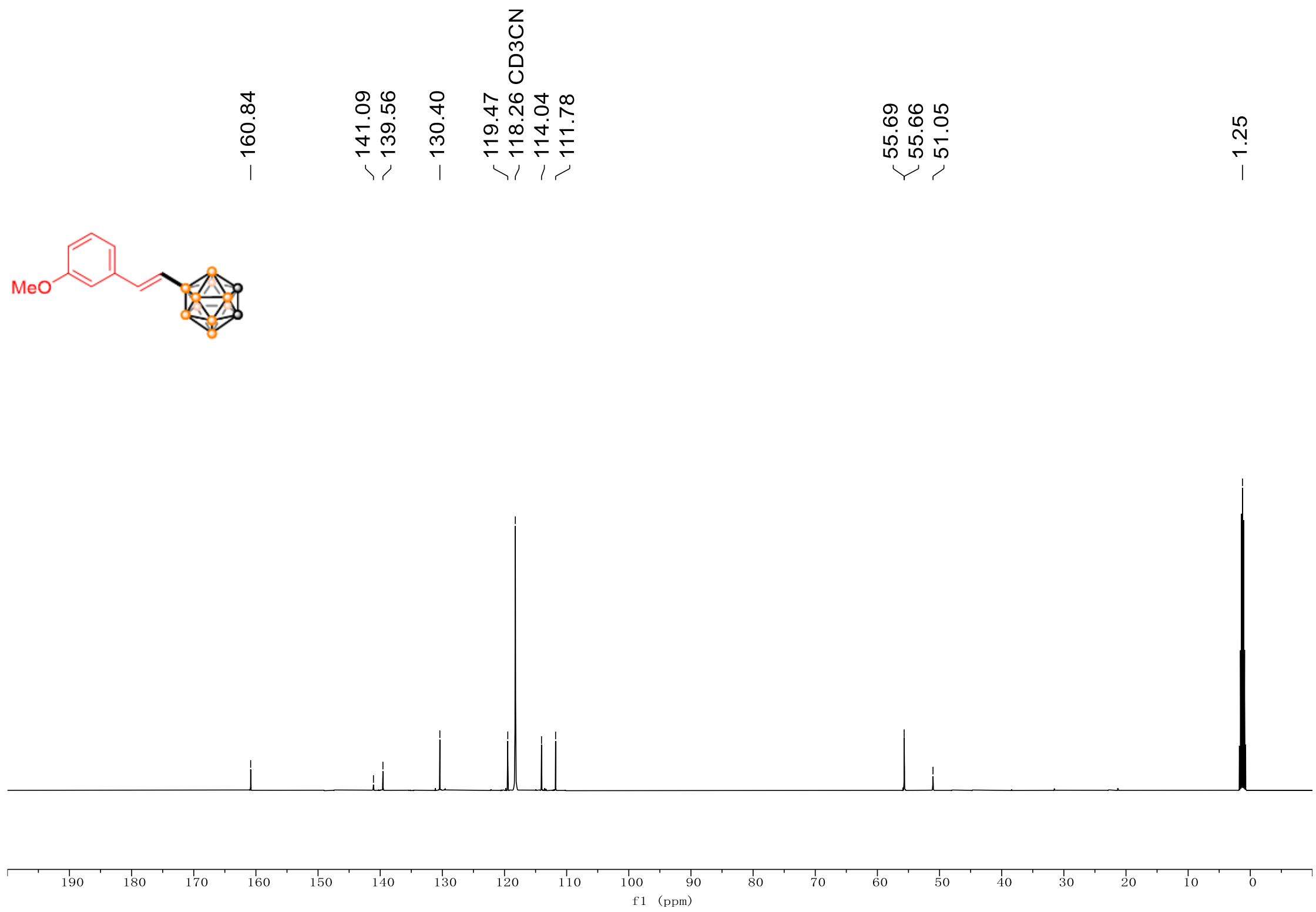

$^{13}C\{^{1}H\}$ NMR (125 MHz, CD_3CN) of **3ag**

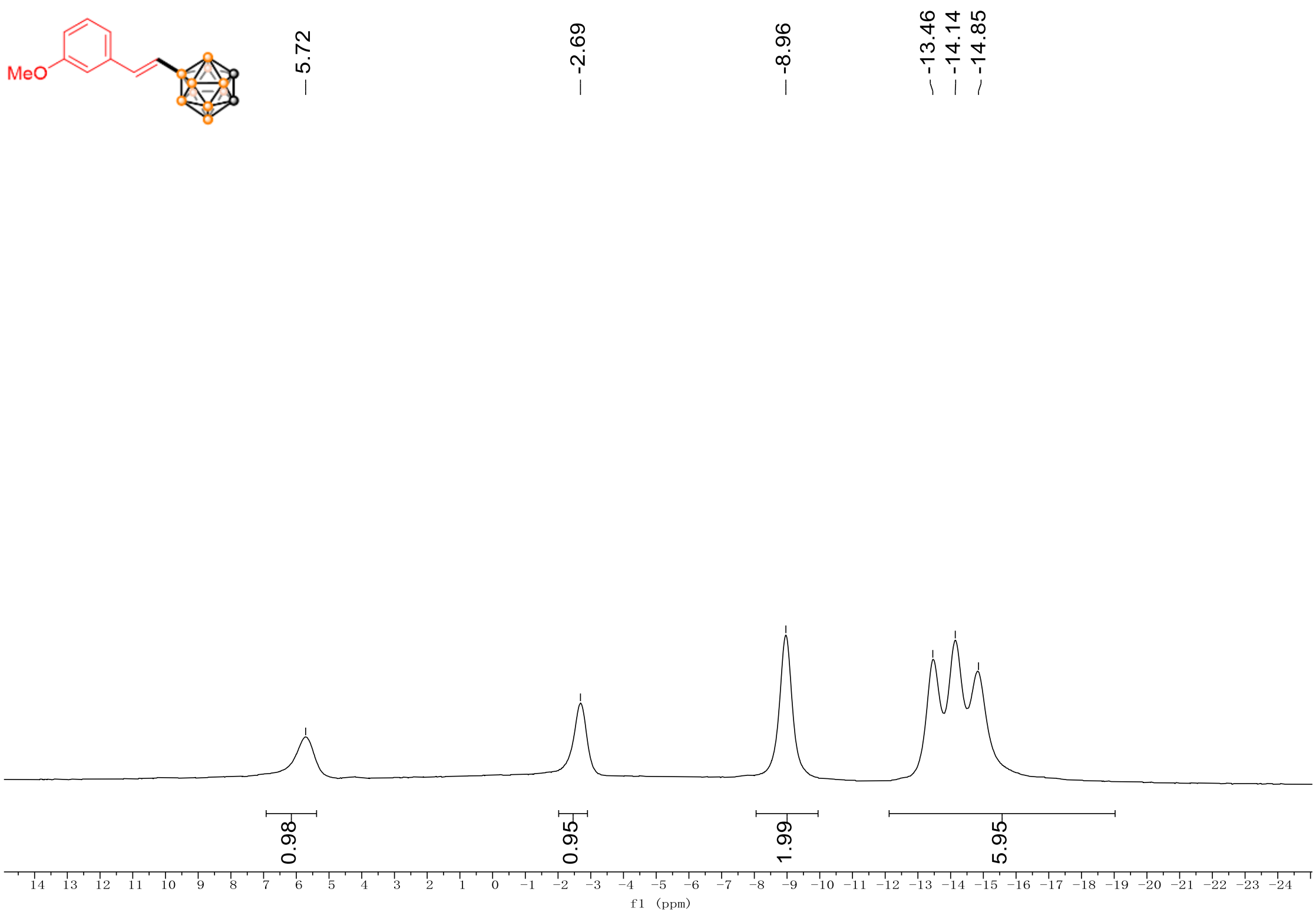

$^{11}B\{^{1}H\}$ NMR (160 MHz, CD_3CN) of **3ag**

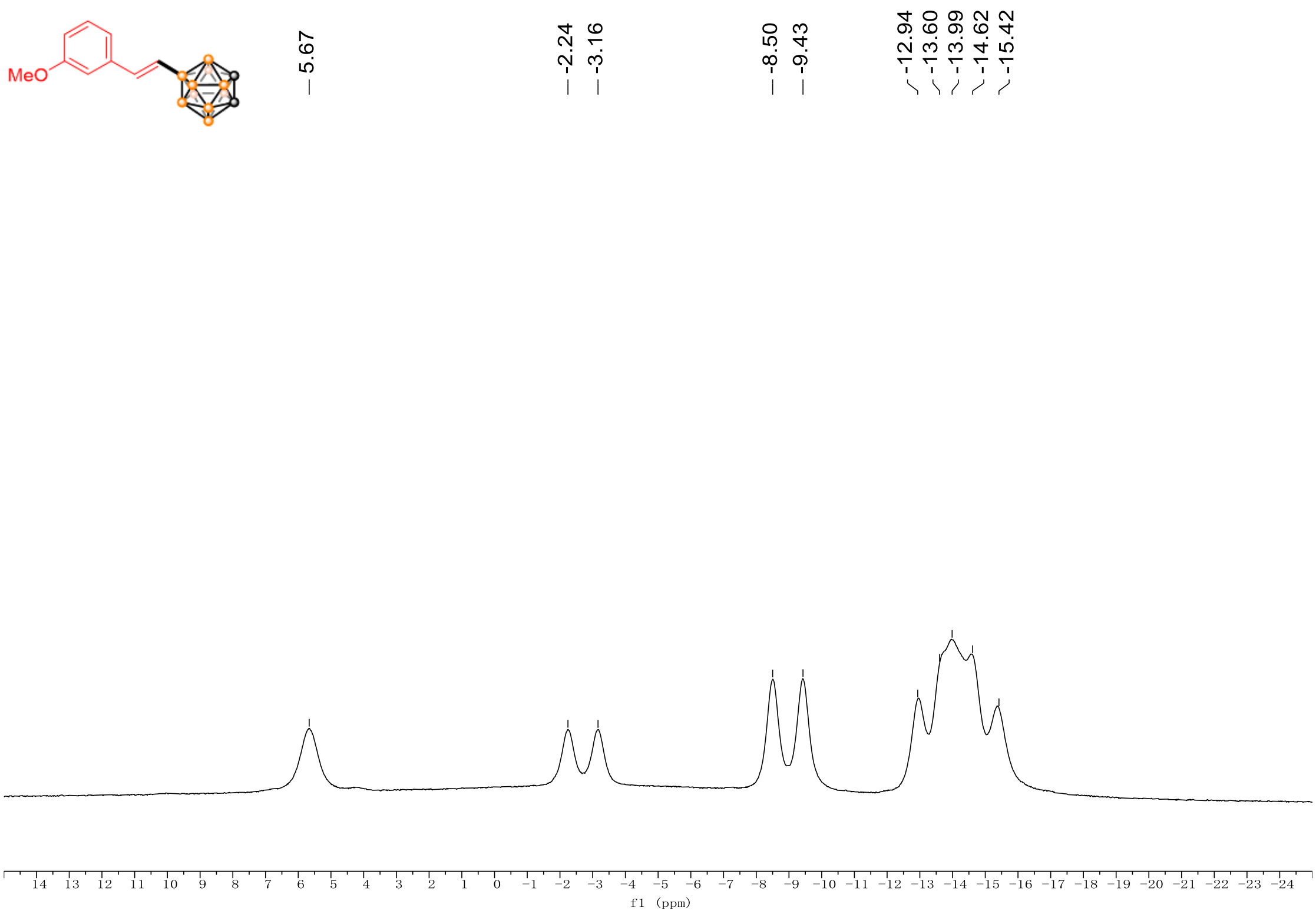

^{11}B NMR (160 MHz, CD_3CN) of **3ag**

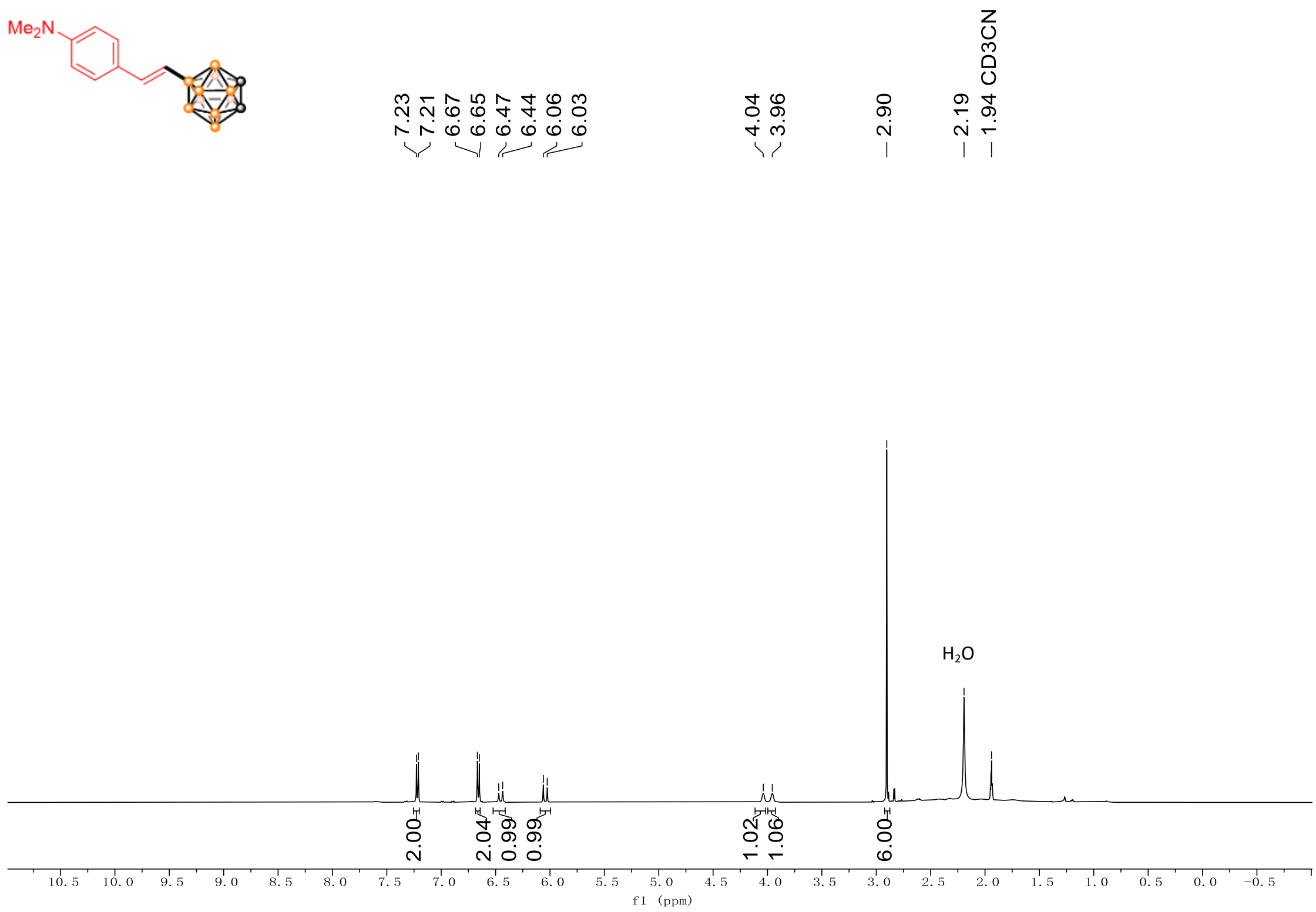

^{1}H NMR (500 MHz, CD_3CN) of **3ah**

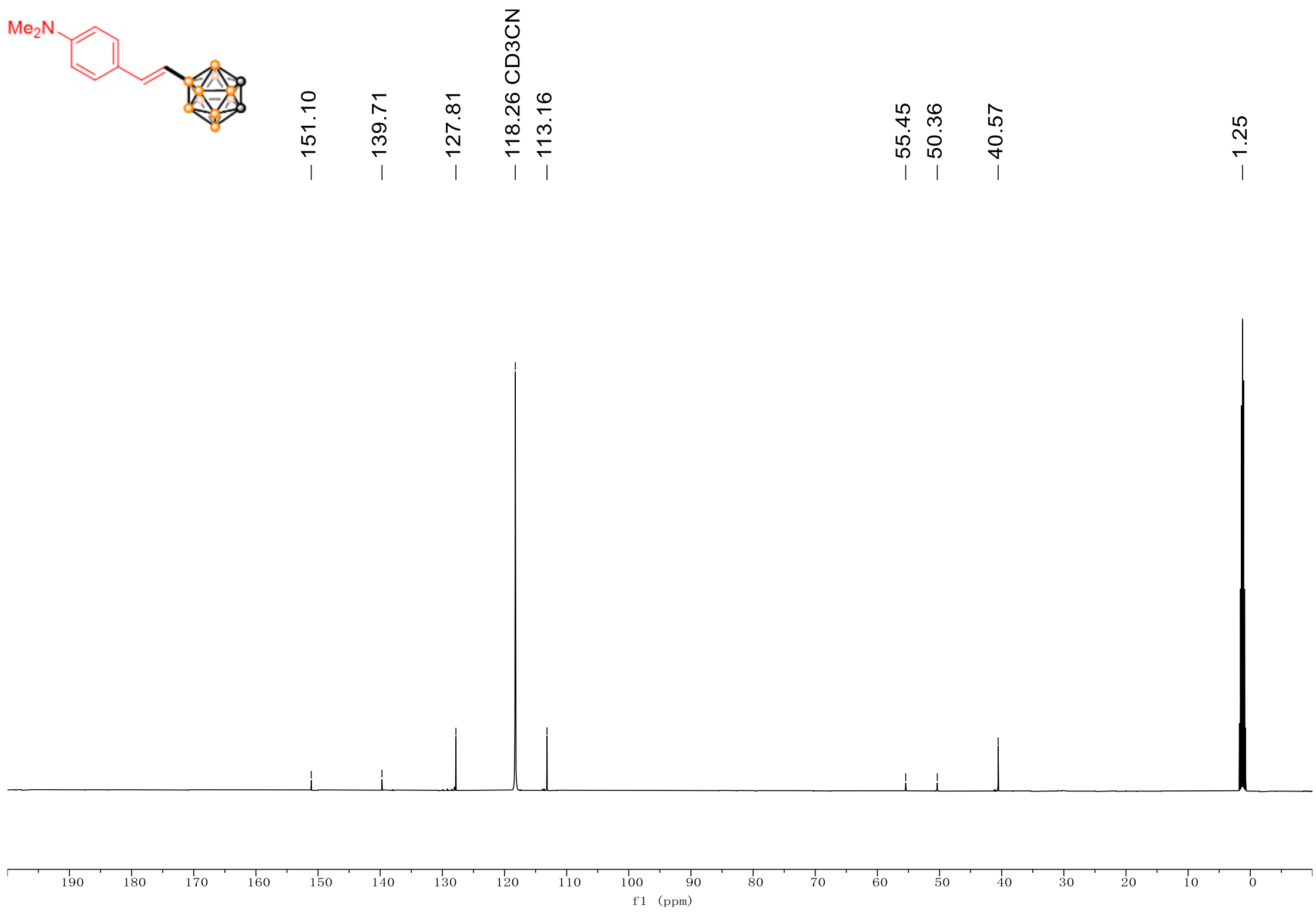

$^{13}C\{^{1}H\}$ NMR (125 MHz, CD_3CN) of **3ah**

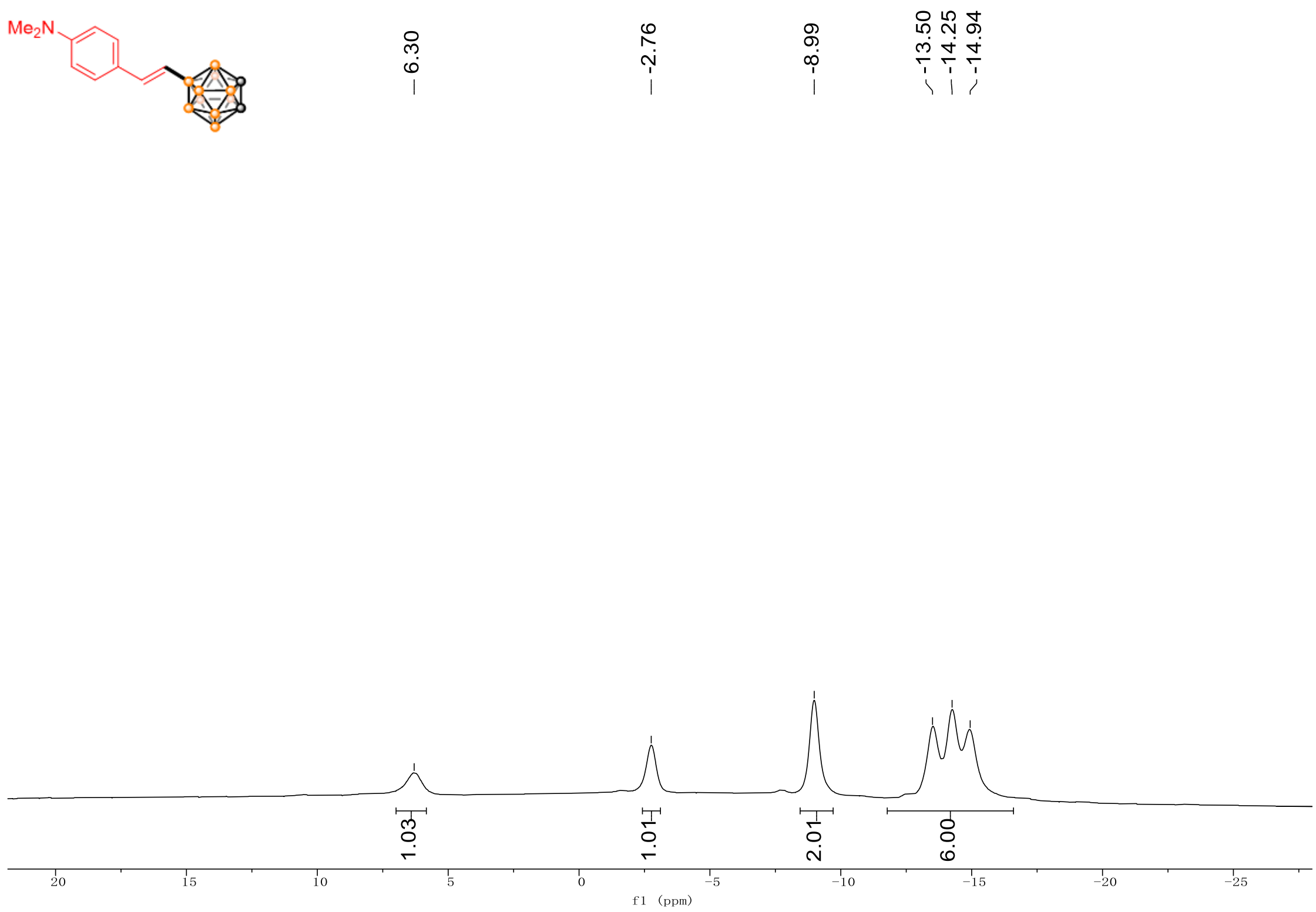

$^{11}B\{^{1}H\}$ NMR (160 MHz, CD_3CN) of **3ah**

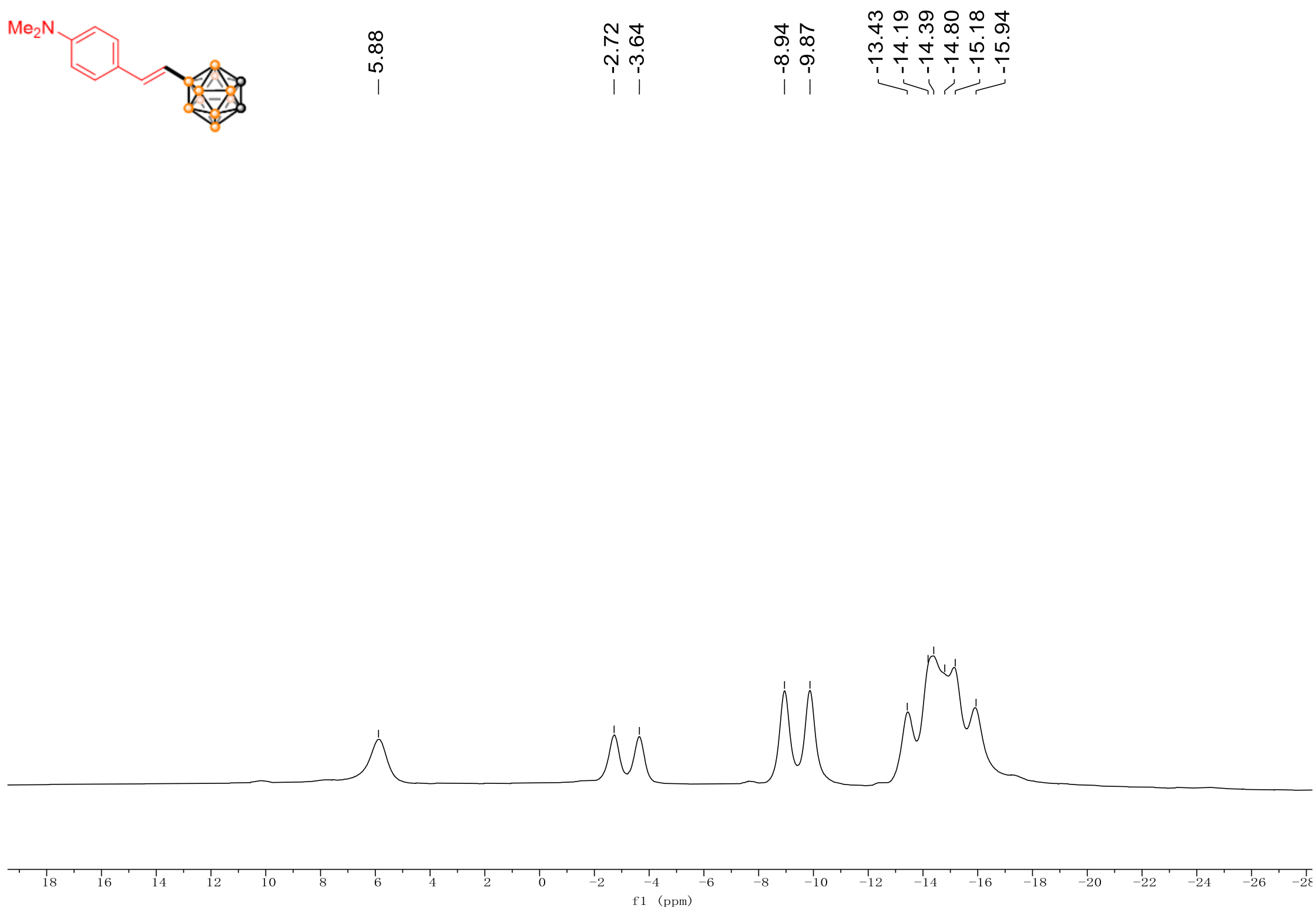

^{11}B NMR (160 MHz, CD_3CN) of **3bb**

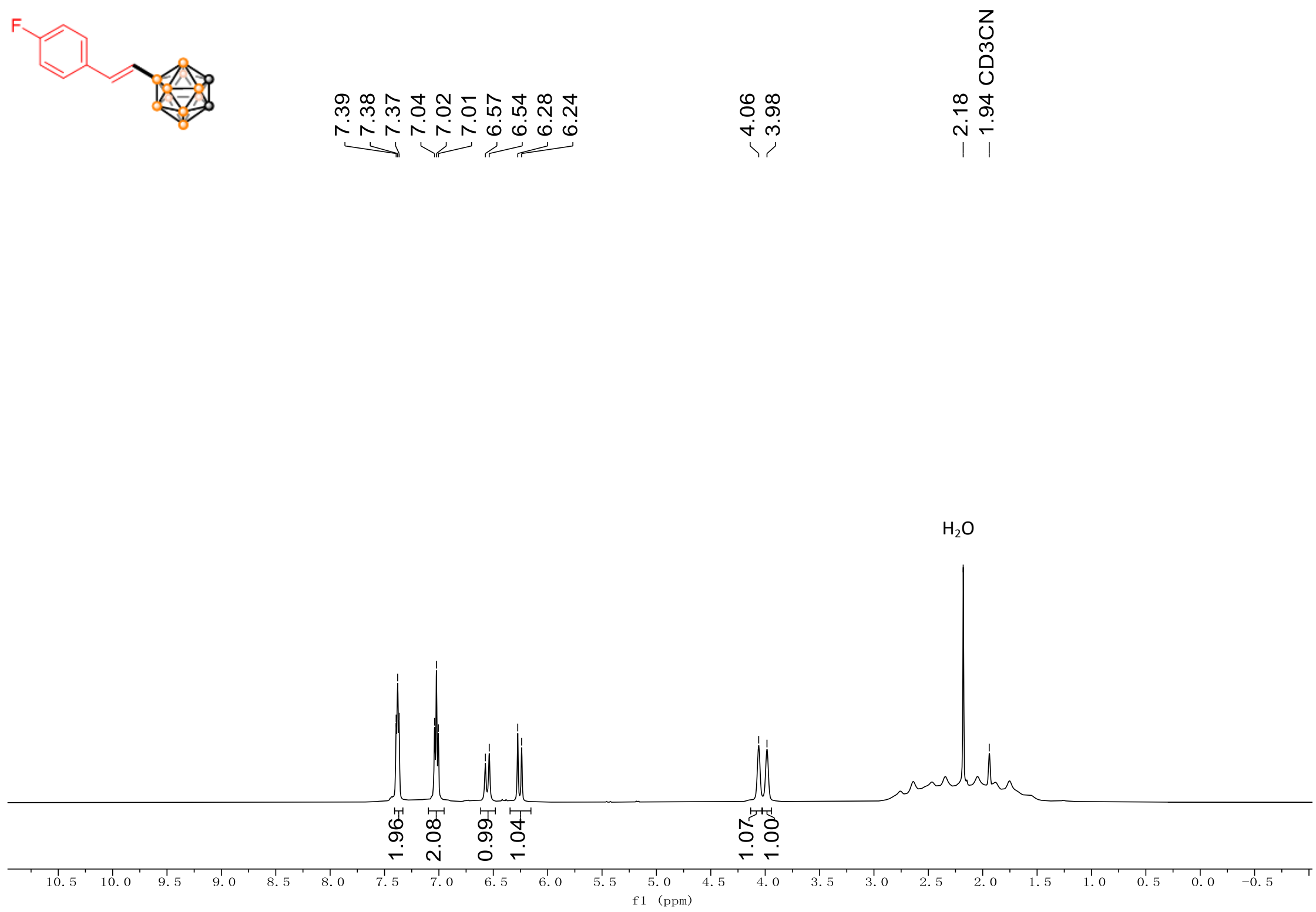

^{1}H NMR (500 MHz, CD_3CN) of **3ai**

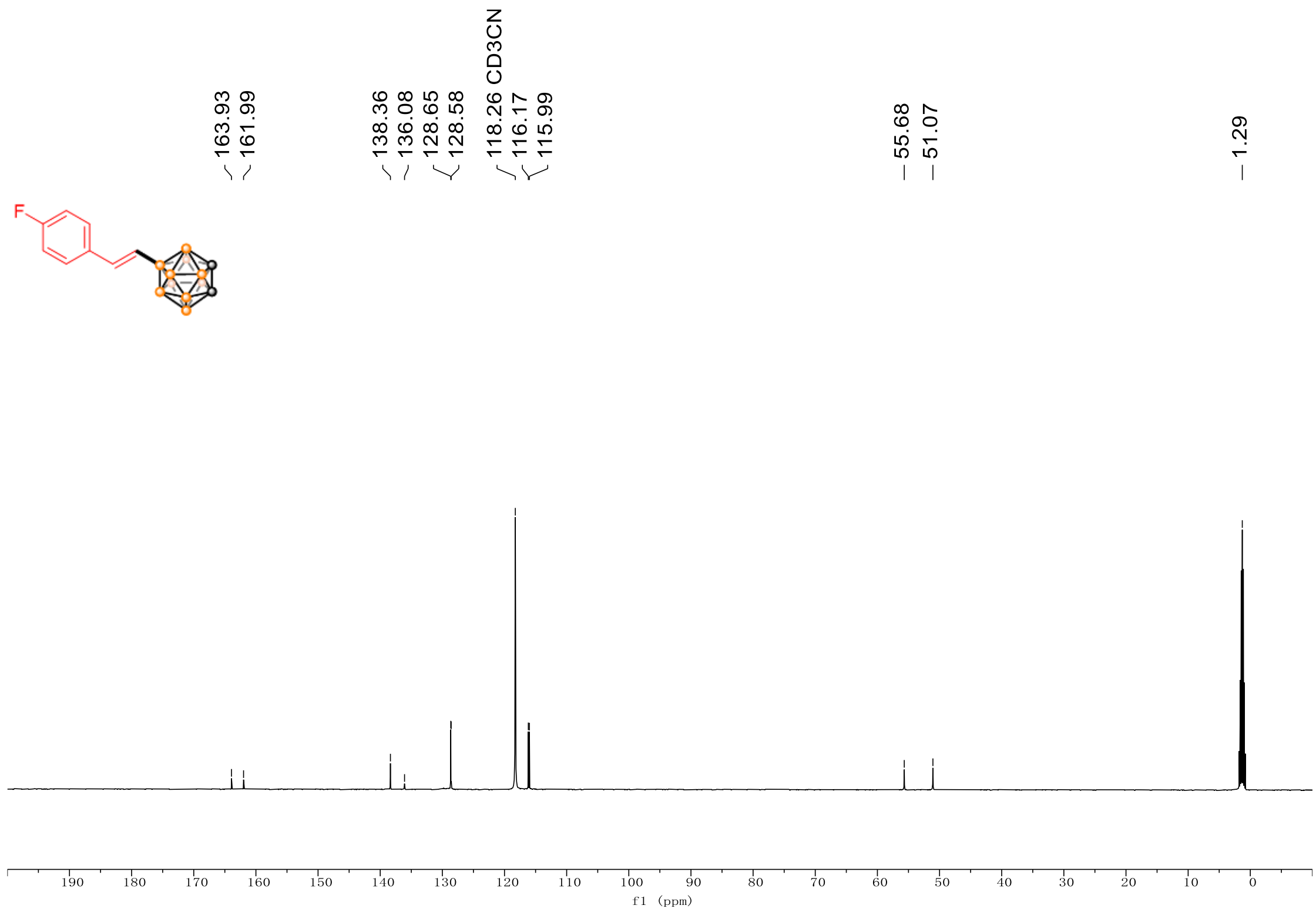

$^{13}C\{^1H\}$ NMR (125 MHz, CD_3CN) of **3ai**

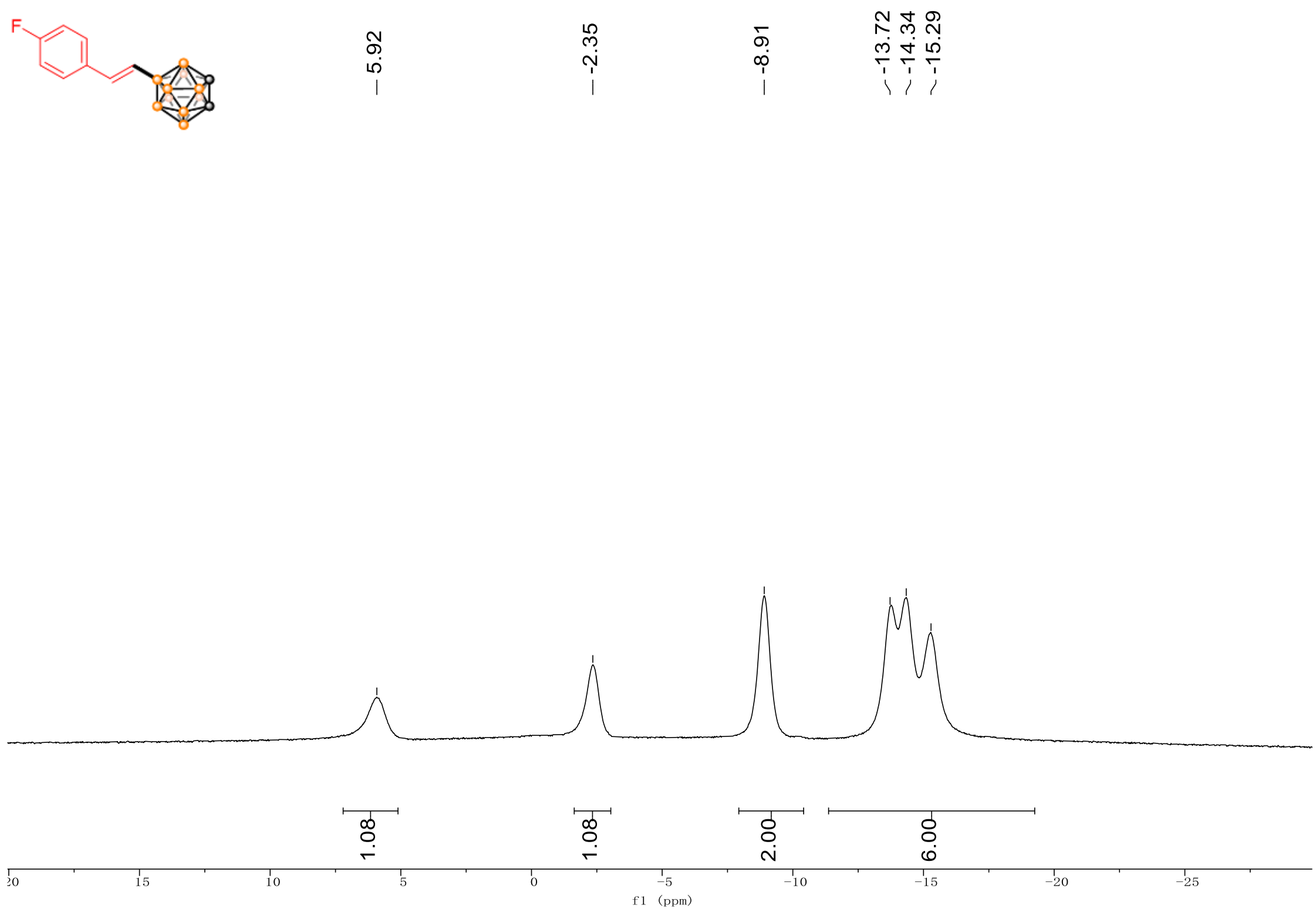

$^{11}B\{^{1}H\}$ NMR (160 MHz, CD_3CN) of **3ai**

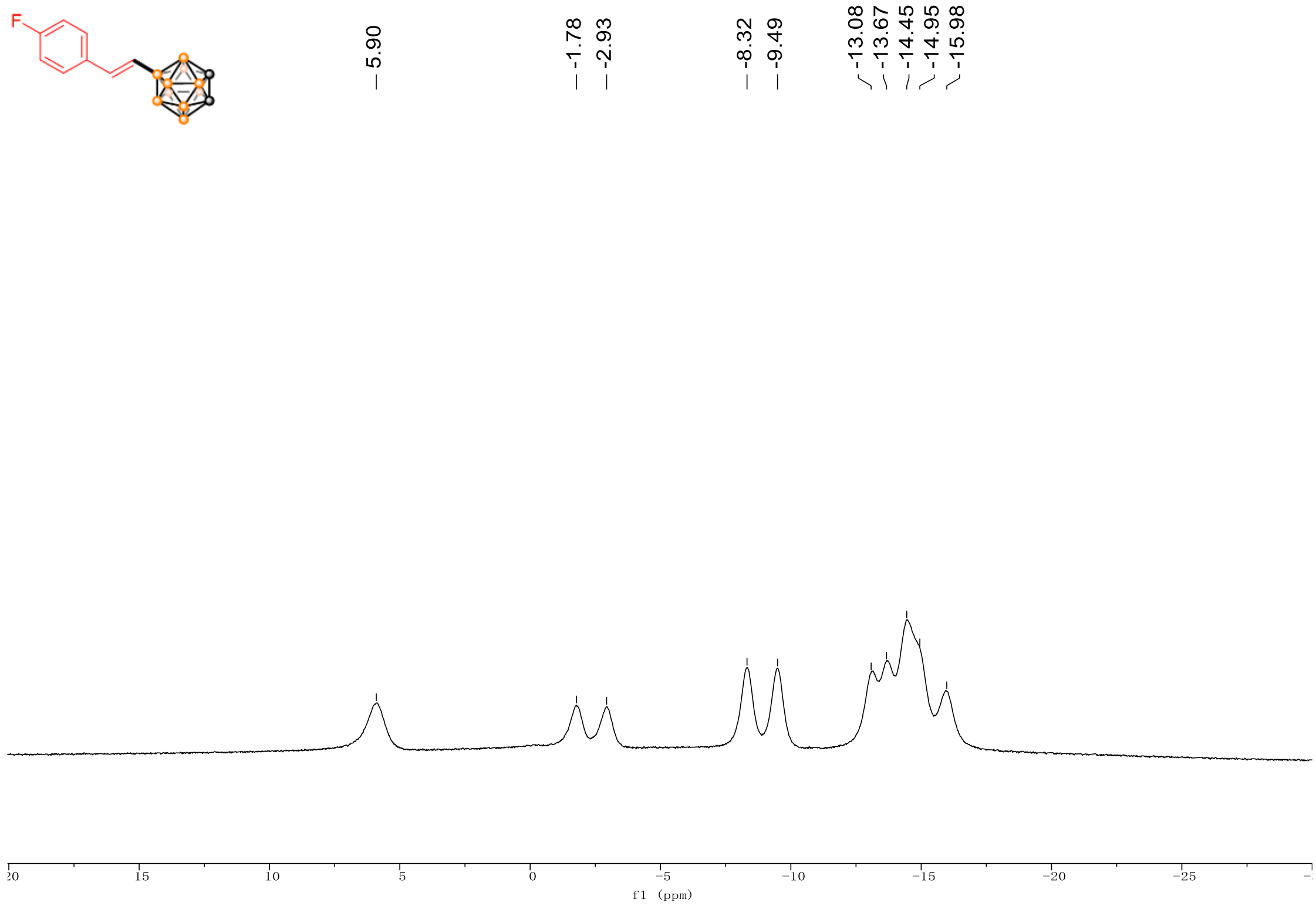

^{11}B NMR (160 MHz, CD_3CN) of **3ai**

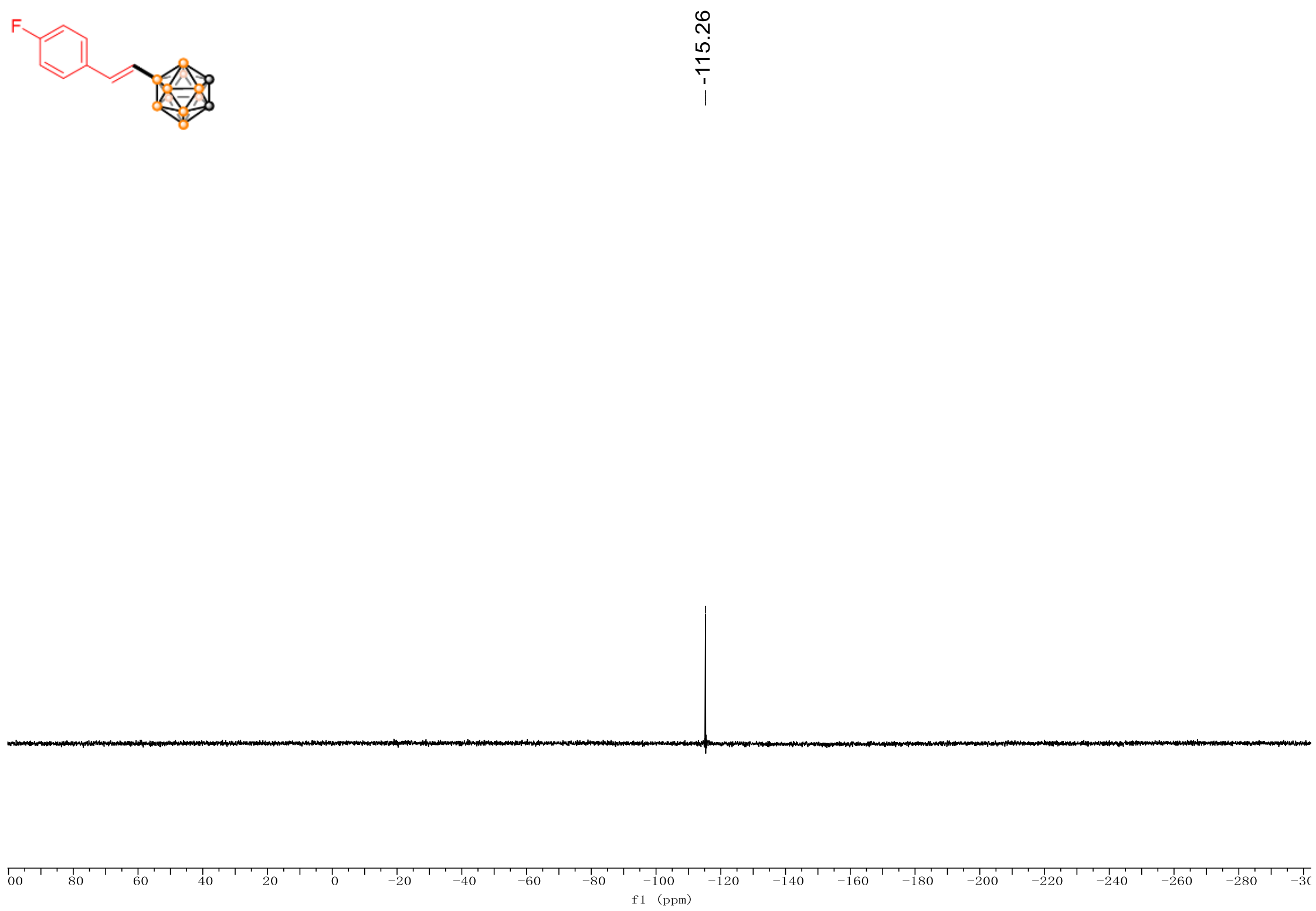

^{19}F NMR (376 MHz, CD_3CN) of **3ai**

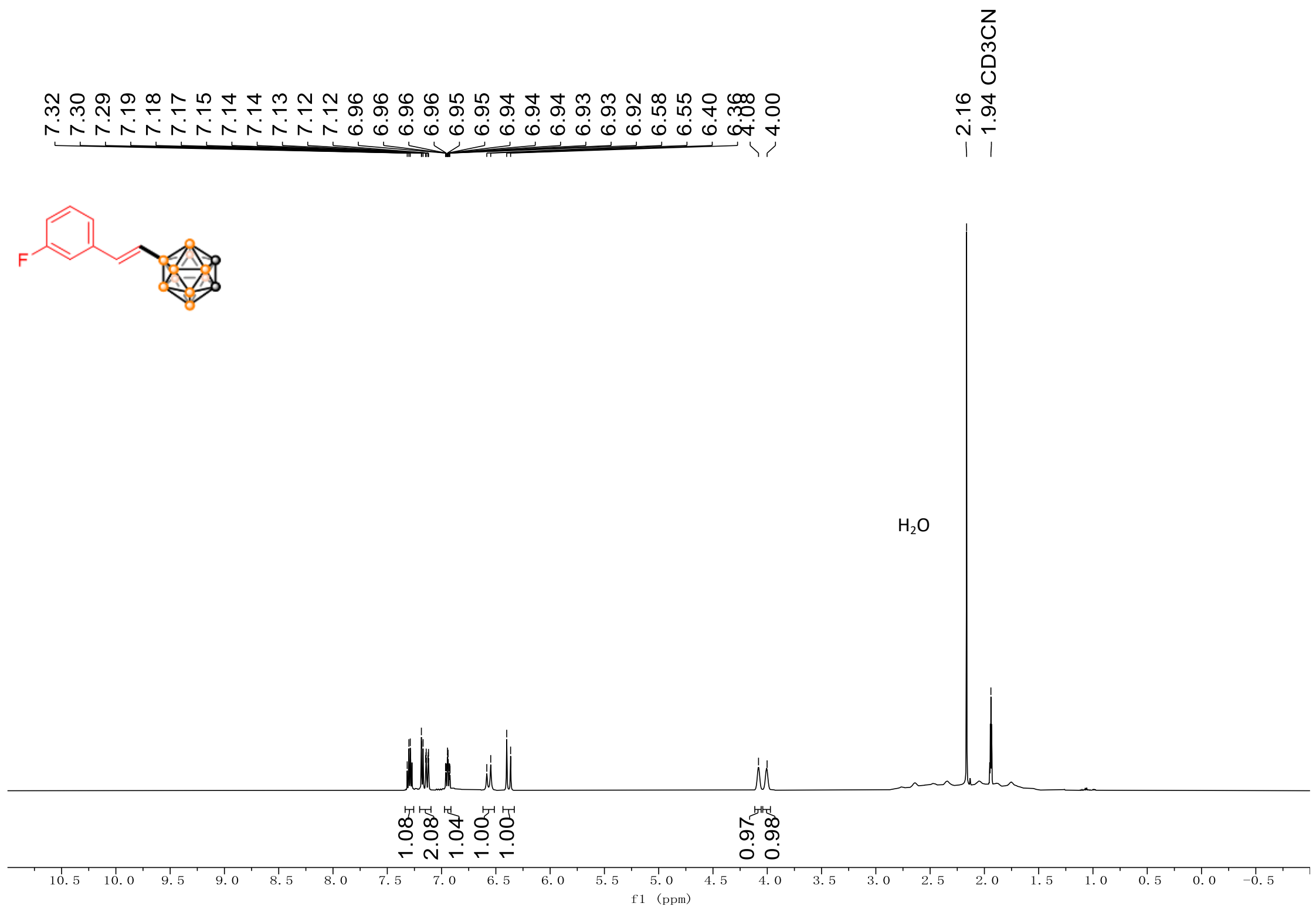

^{1}H NMR (500 MHz, CD_3CN) of **3aj**

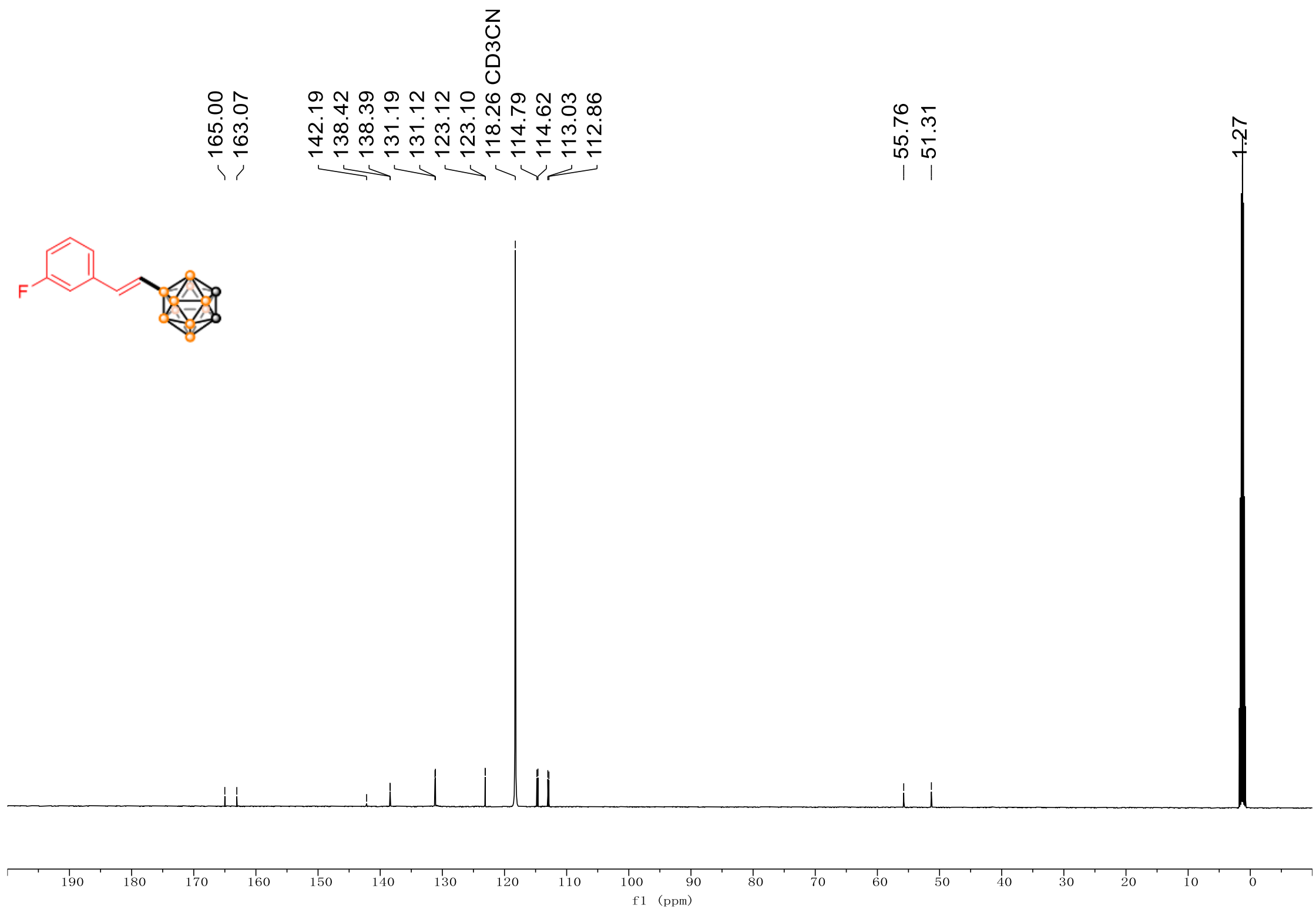

$^{13}C\{^{1}H\}$ NMR (125 MHz, CD_3CN) of **3aj**

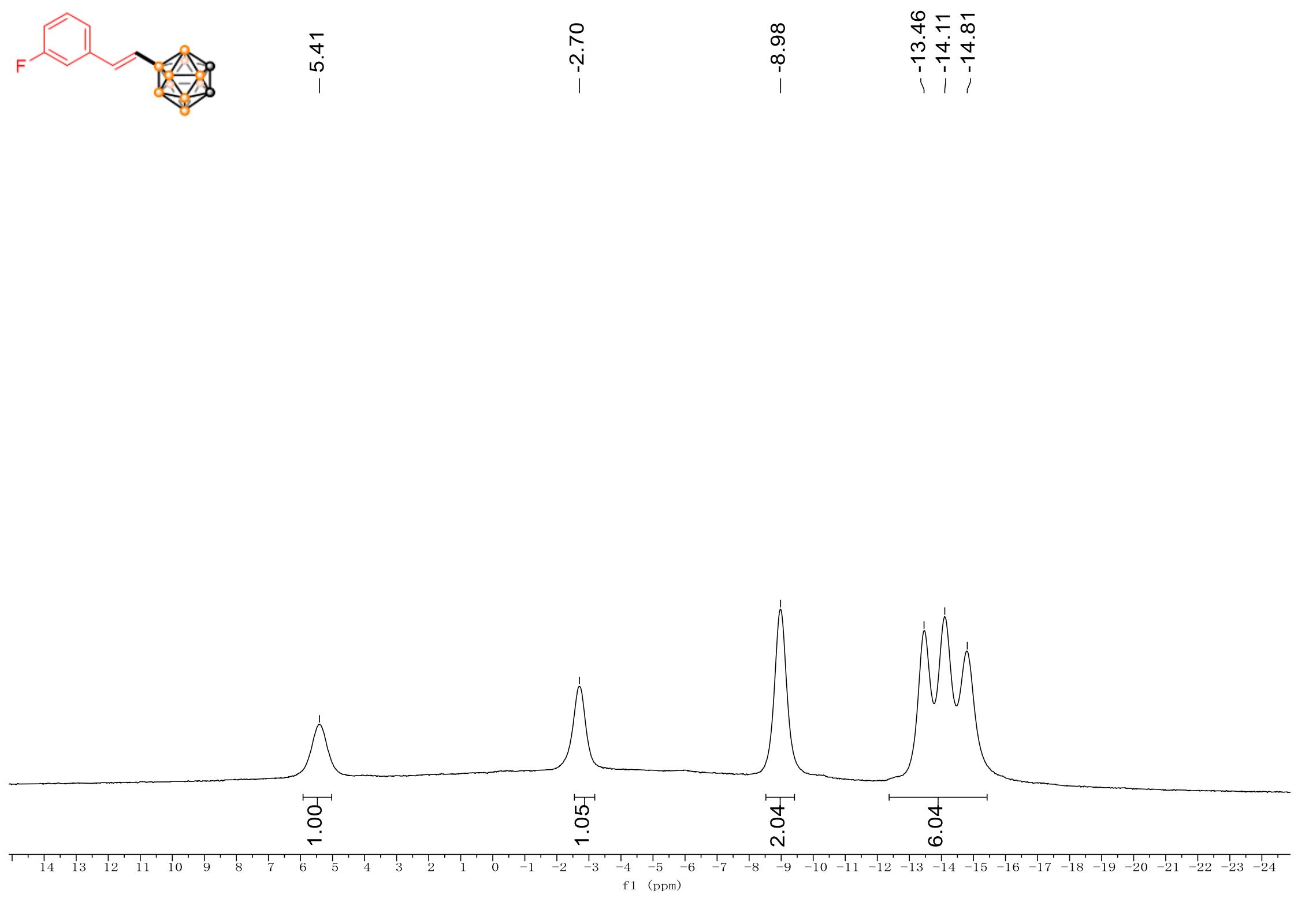

$^{11}B\{^{1}H\}$ NMR (160 MHz, CD_3CN) of **3aj**

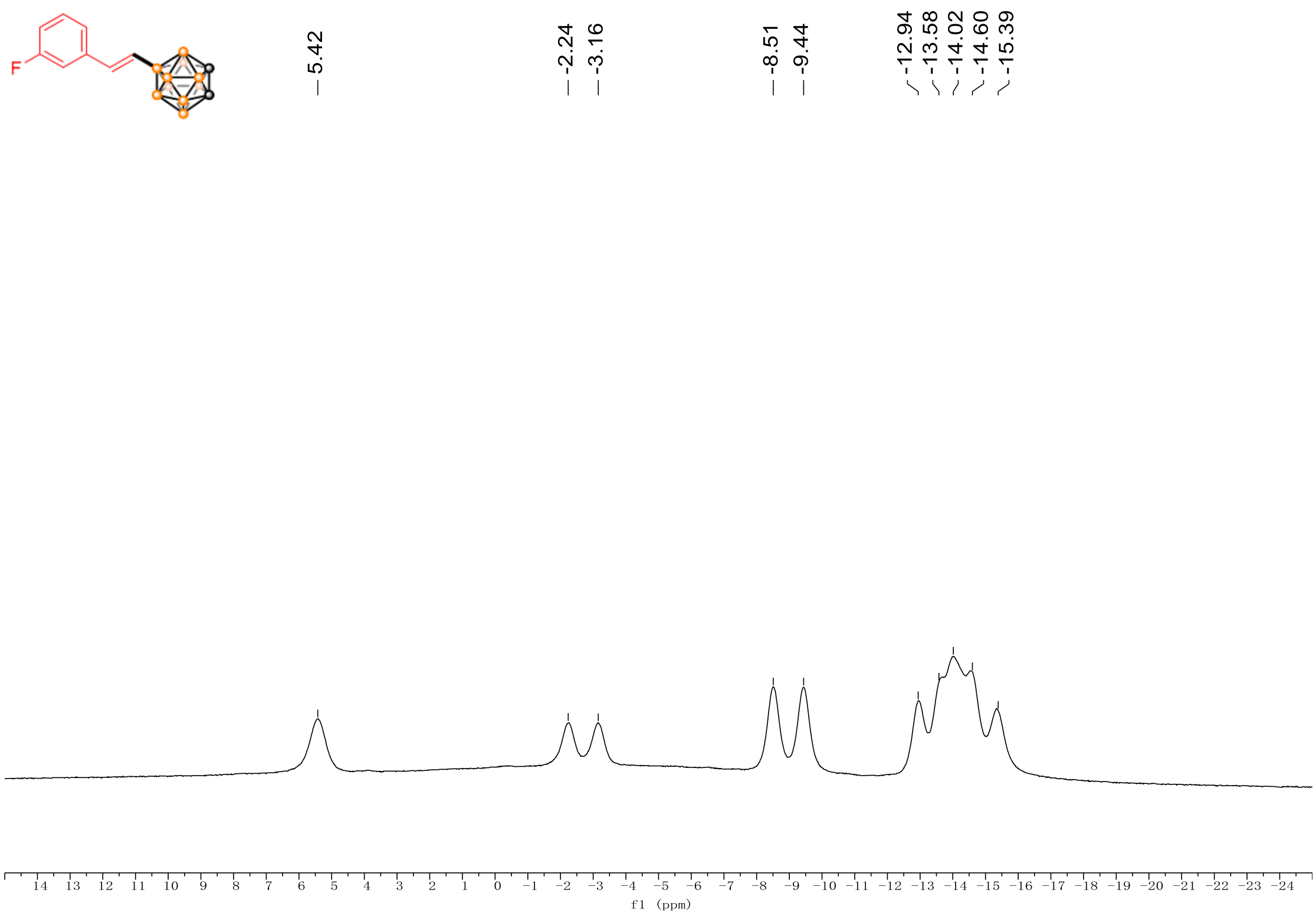

^{11}B NMR (160 MHz, CD_3CN) of **3aj**

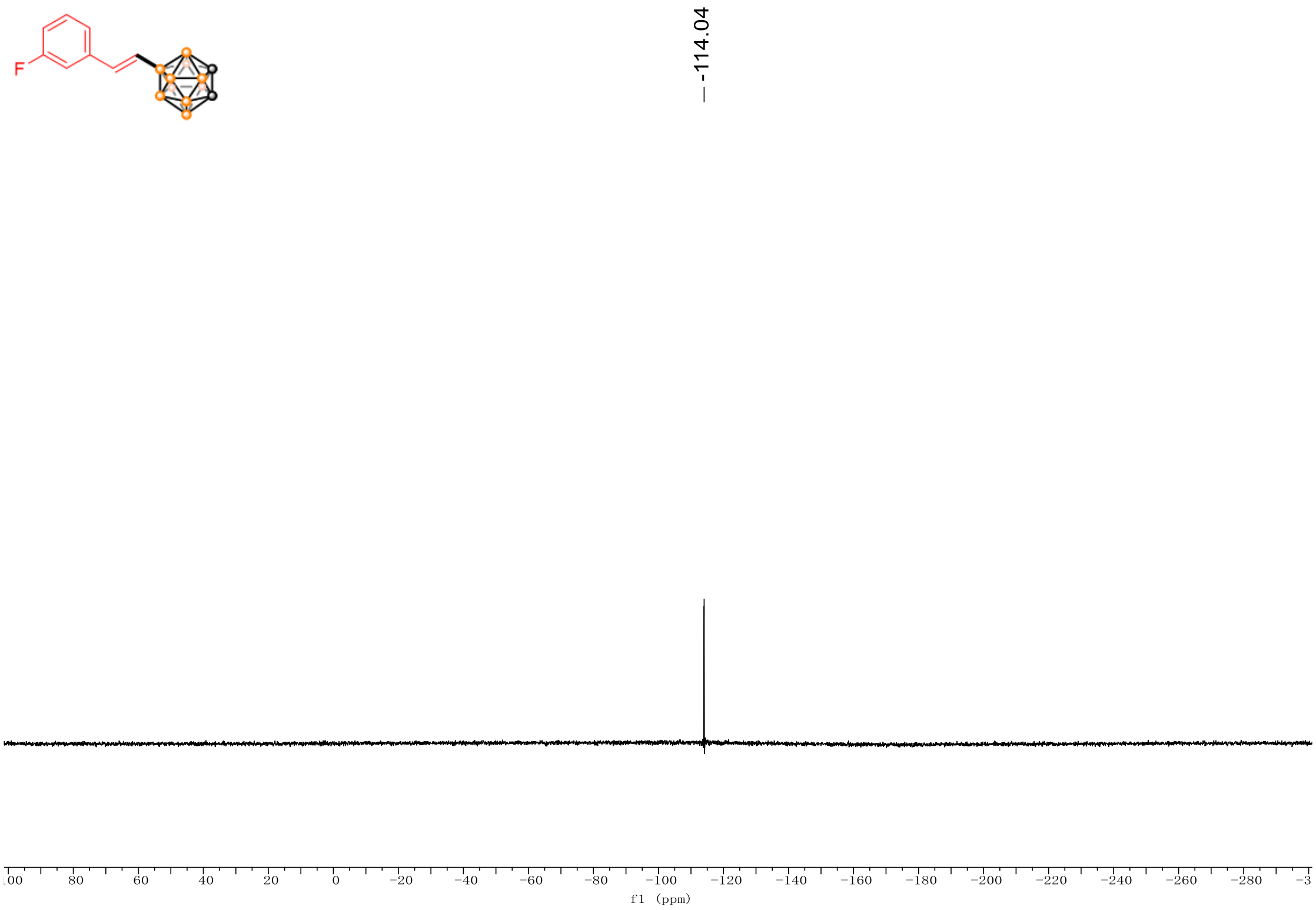

^{19}F NMR (376 MHz, CD_3CN) of **3aj**

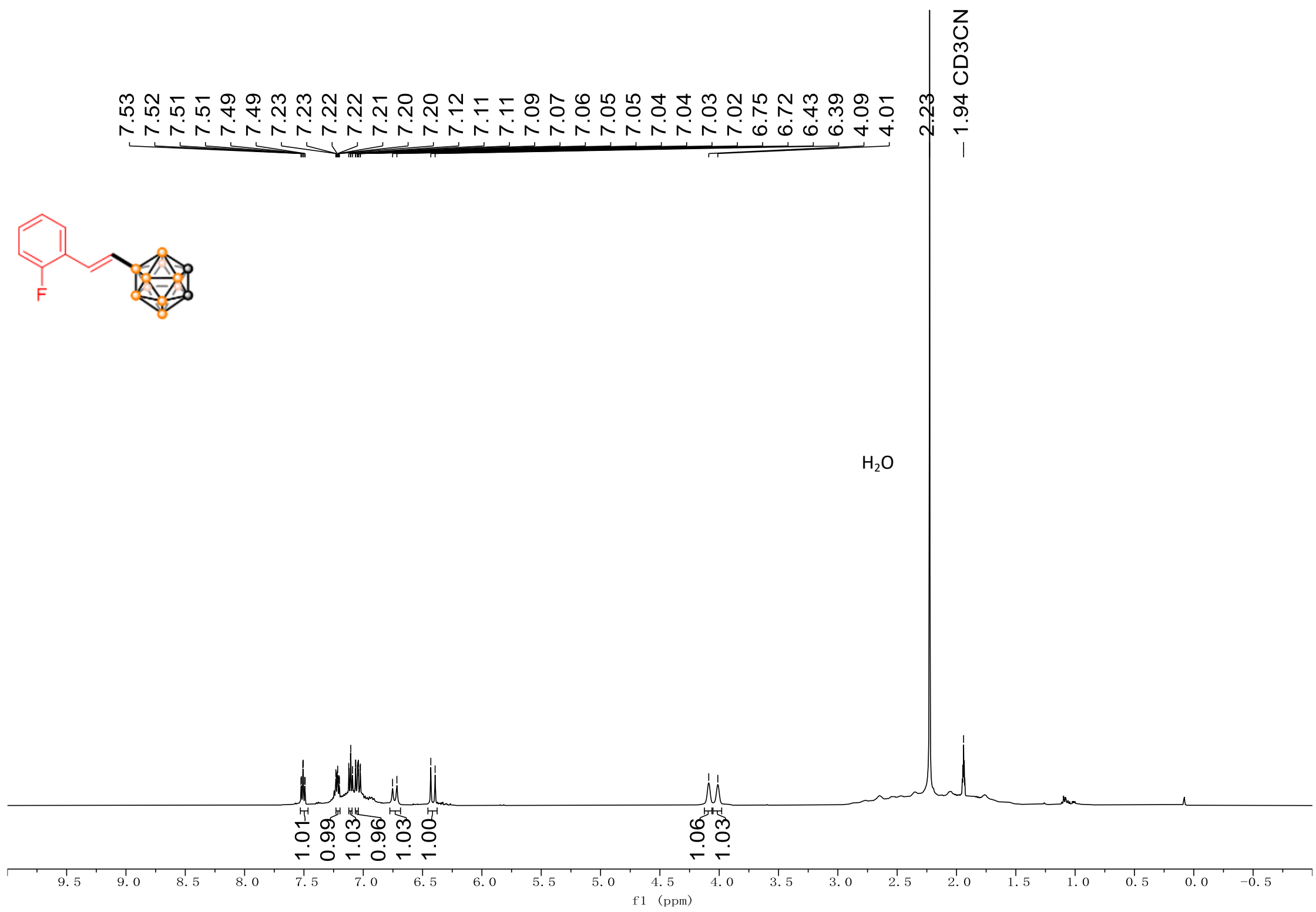

^{1}H NMR (500 MHz, CD_3CN) of **3ak**

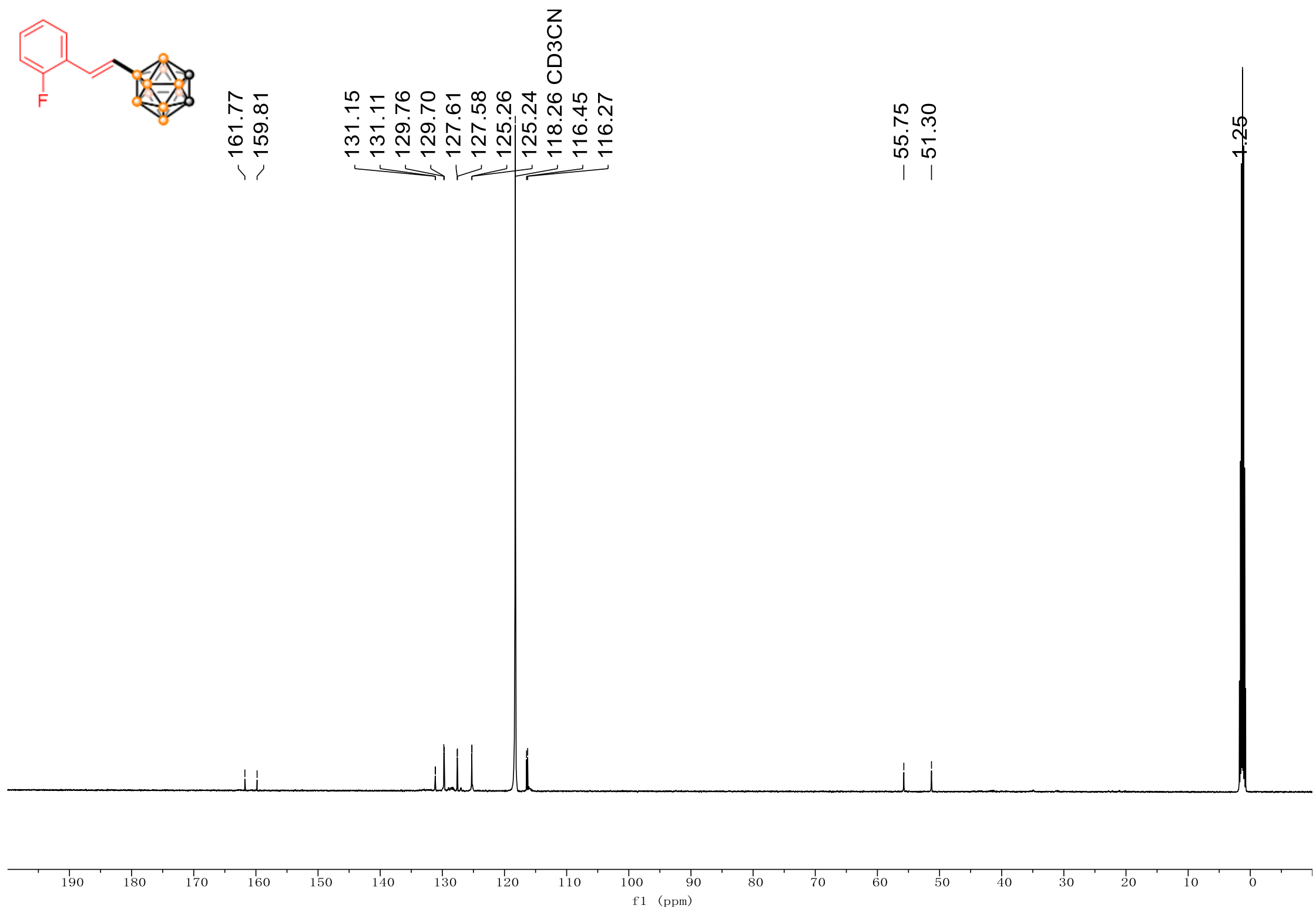

^{13}C{^{1}H} NMR (125 MHz, CD_3CN) of **3ak**

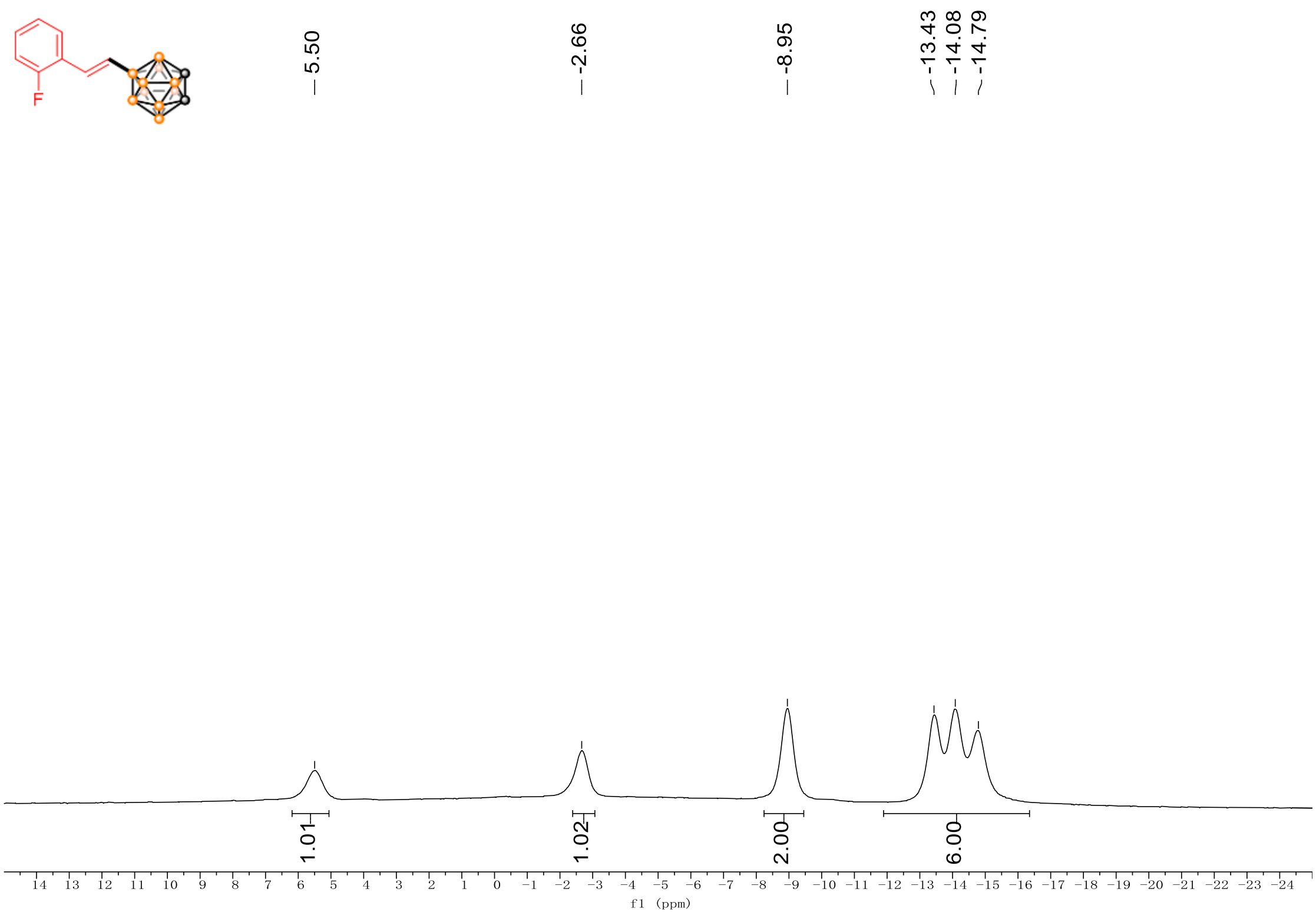

$^{11}B\{^{1}H\}$ NMR (160 MHz, CD_3CN) of **3ak**

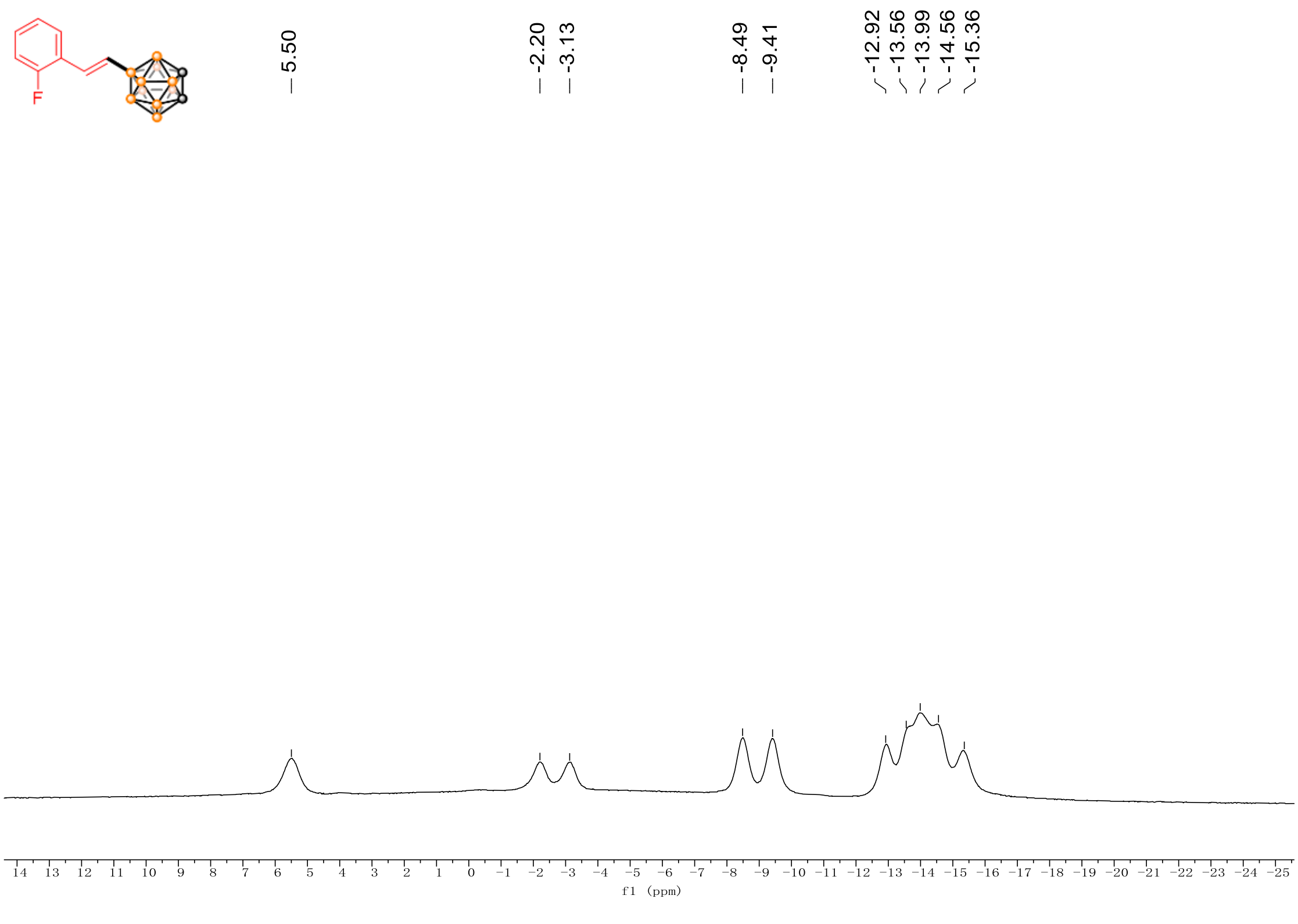

^{11}B NMR (160 MHz, CD_3CN) of **3ak**

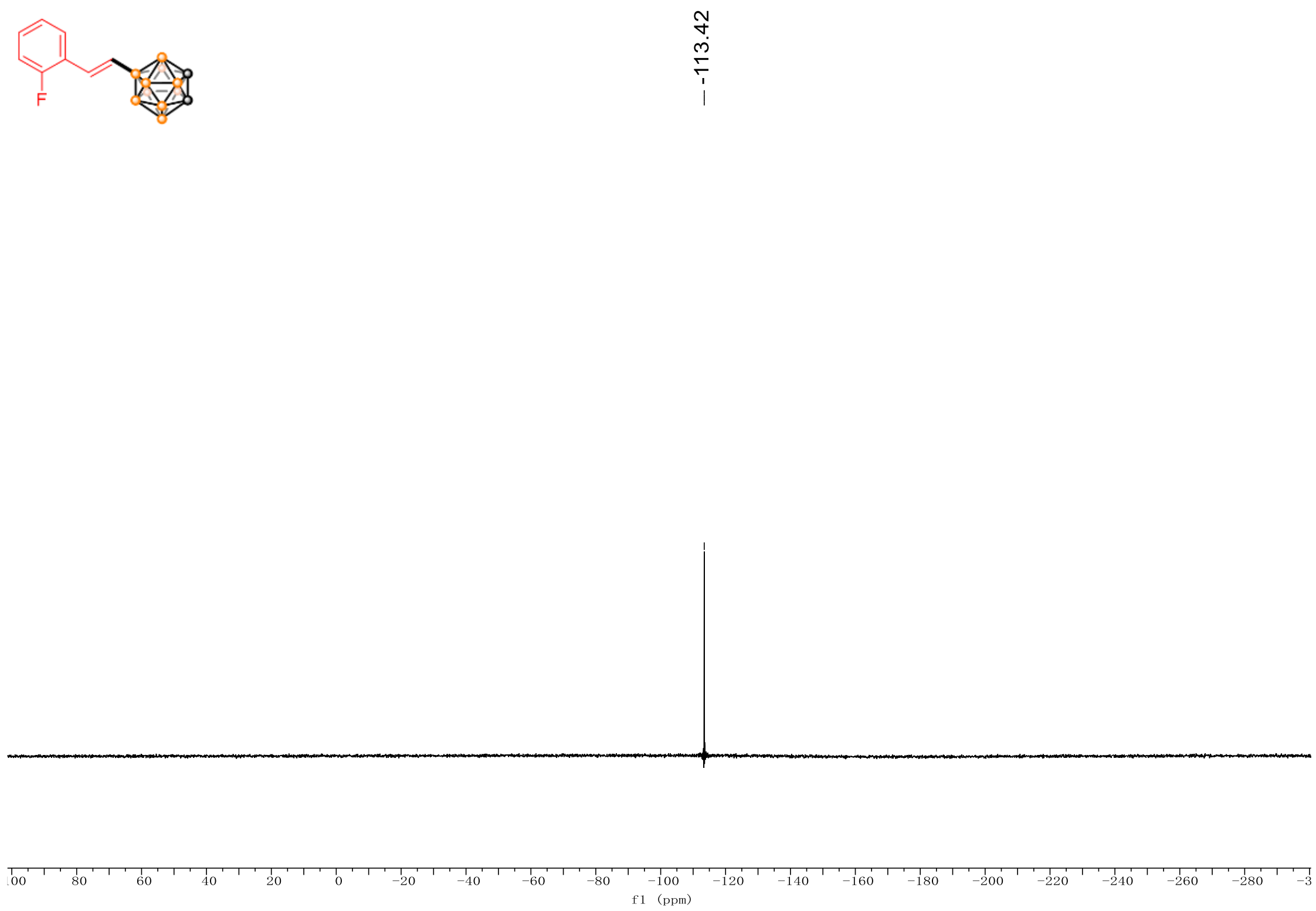

^{19}F NMR (376 MHz, CD_3CN) of **3ak**

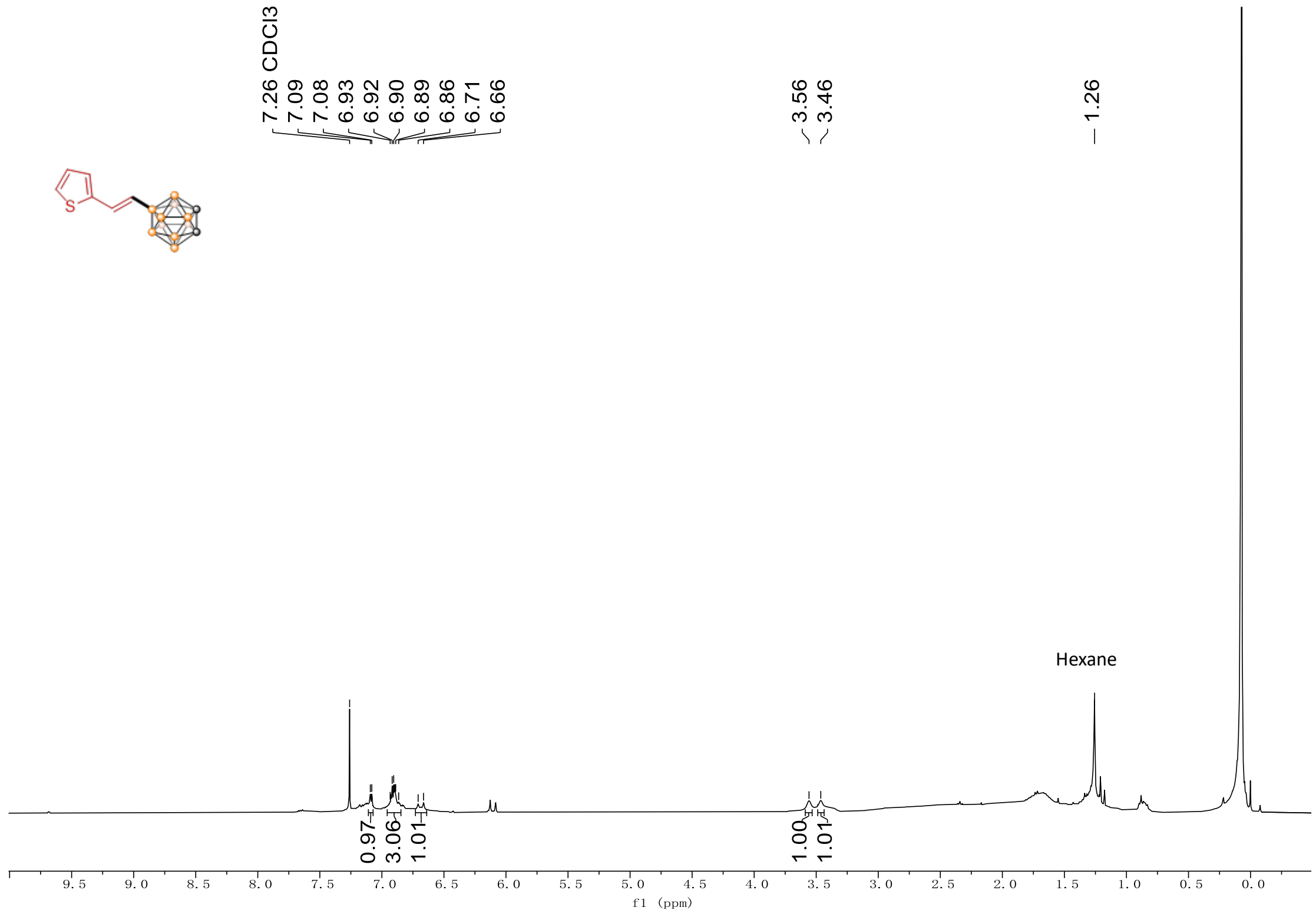

^{1}H NMR (400 MHz, $CDCl_3$) of **3al**

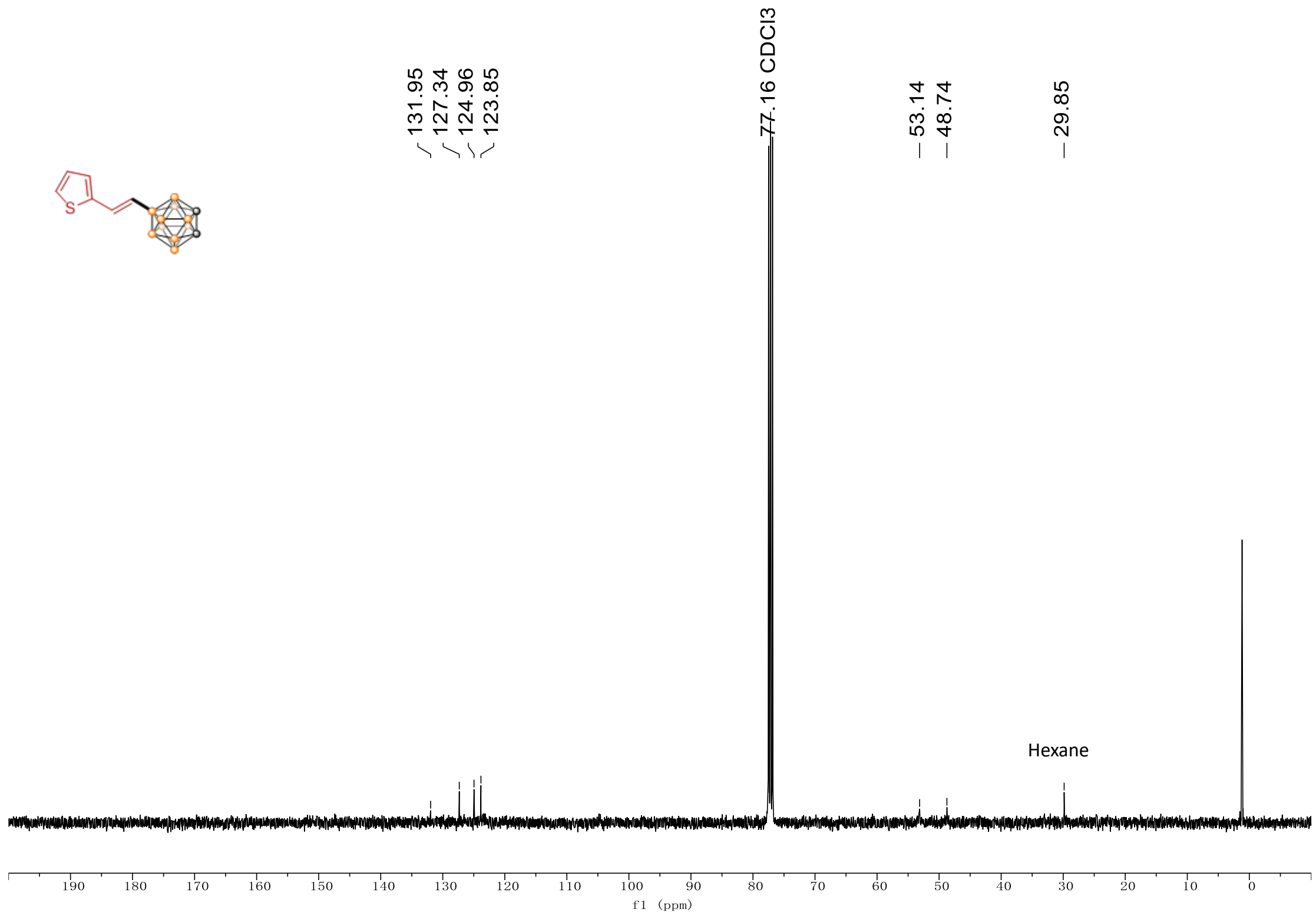

$^{13}C\{^{1}H\}$ NMR (101 MHz, $CDCl_3$) of **3al**

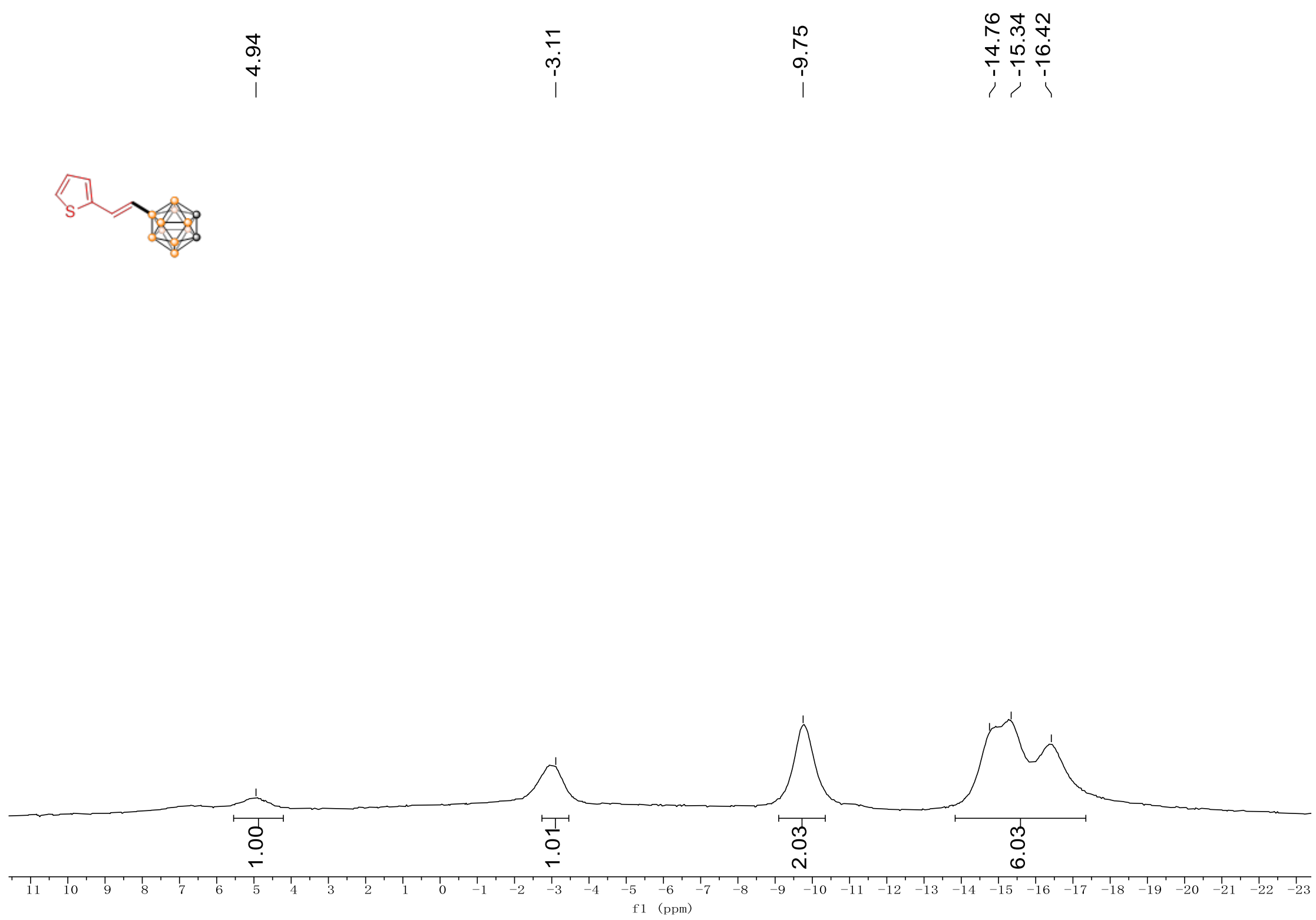

$^{11}B\{^{1}H\}$ NMR (128 MHz, $CDCl_3$) of **3al**

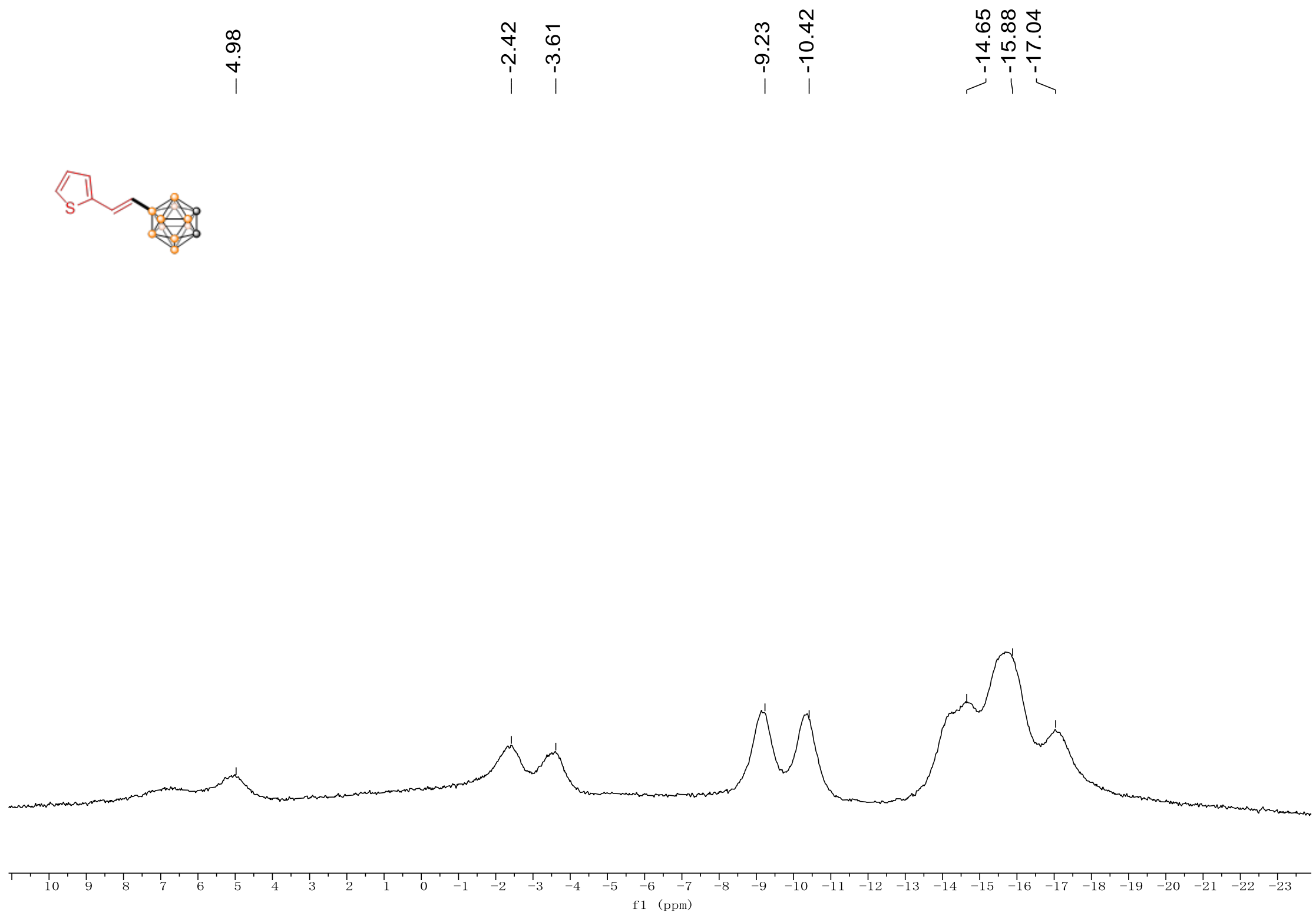

^{11}B NMR (128 MHz, $CDCl_3$) of **3al**

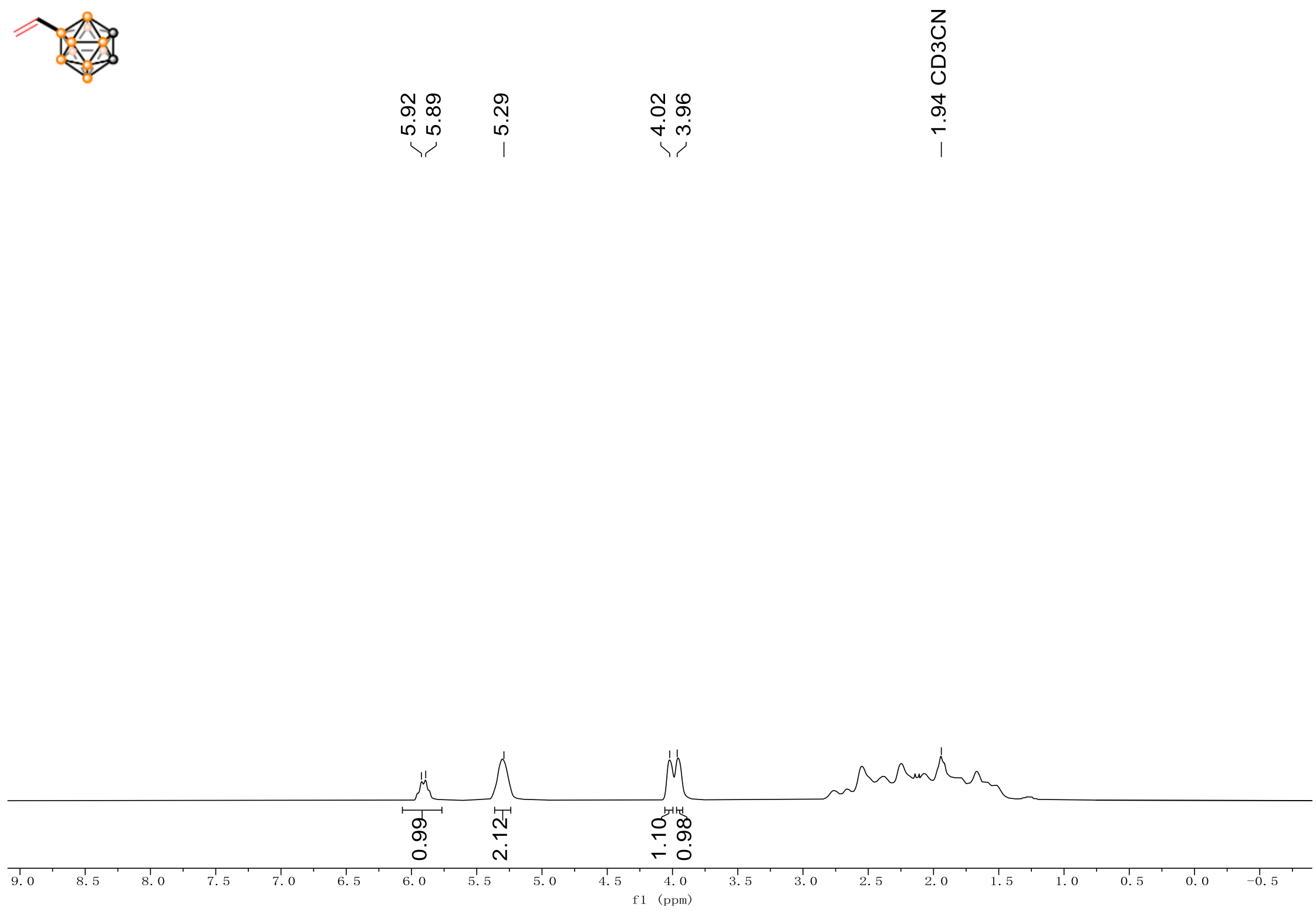

^{1}H NMR (500 MHz, CD_3CN) of **3an**

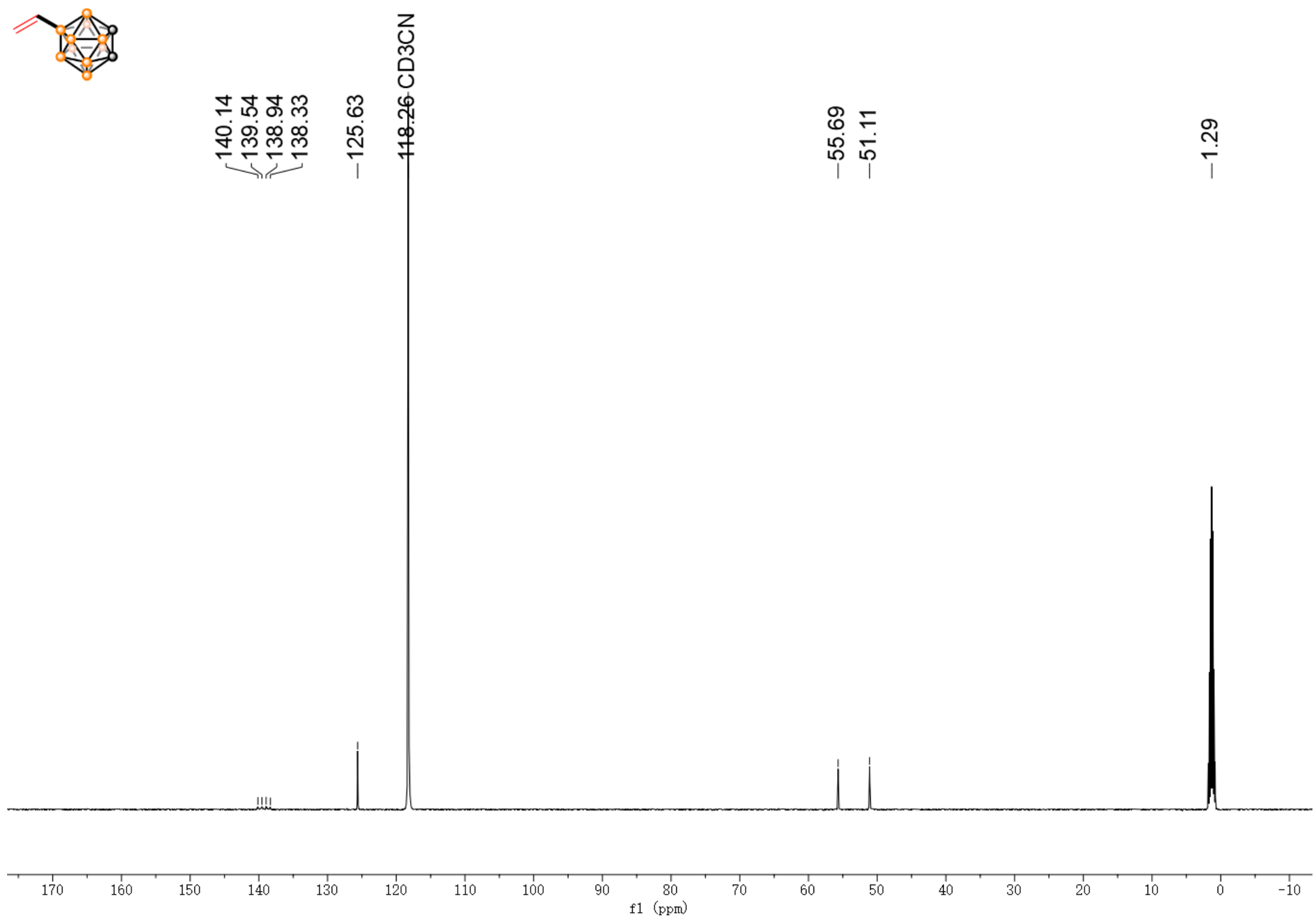

$^{13}C\{^{1}H\}$ NMR (125 MHz, CD_3CN) of **3an**

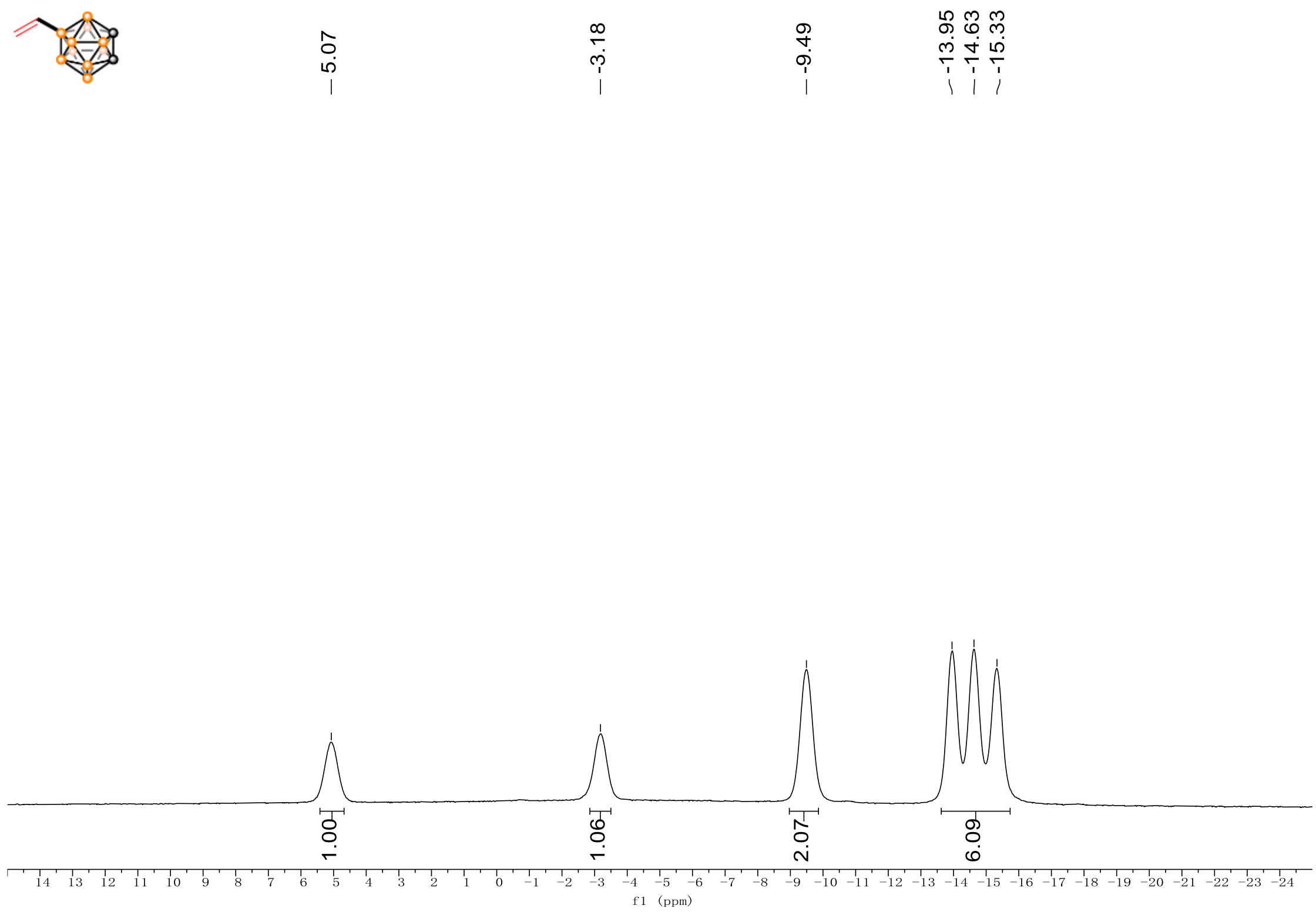

$^{11}B\{^{1}H\}$ NMR (160 MHz, CD_3CN) of **3an**

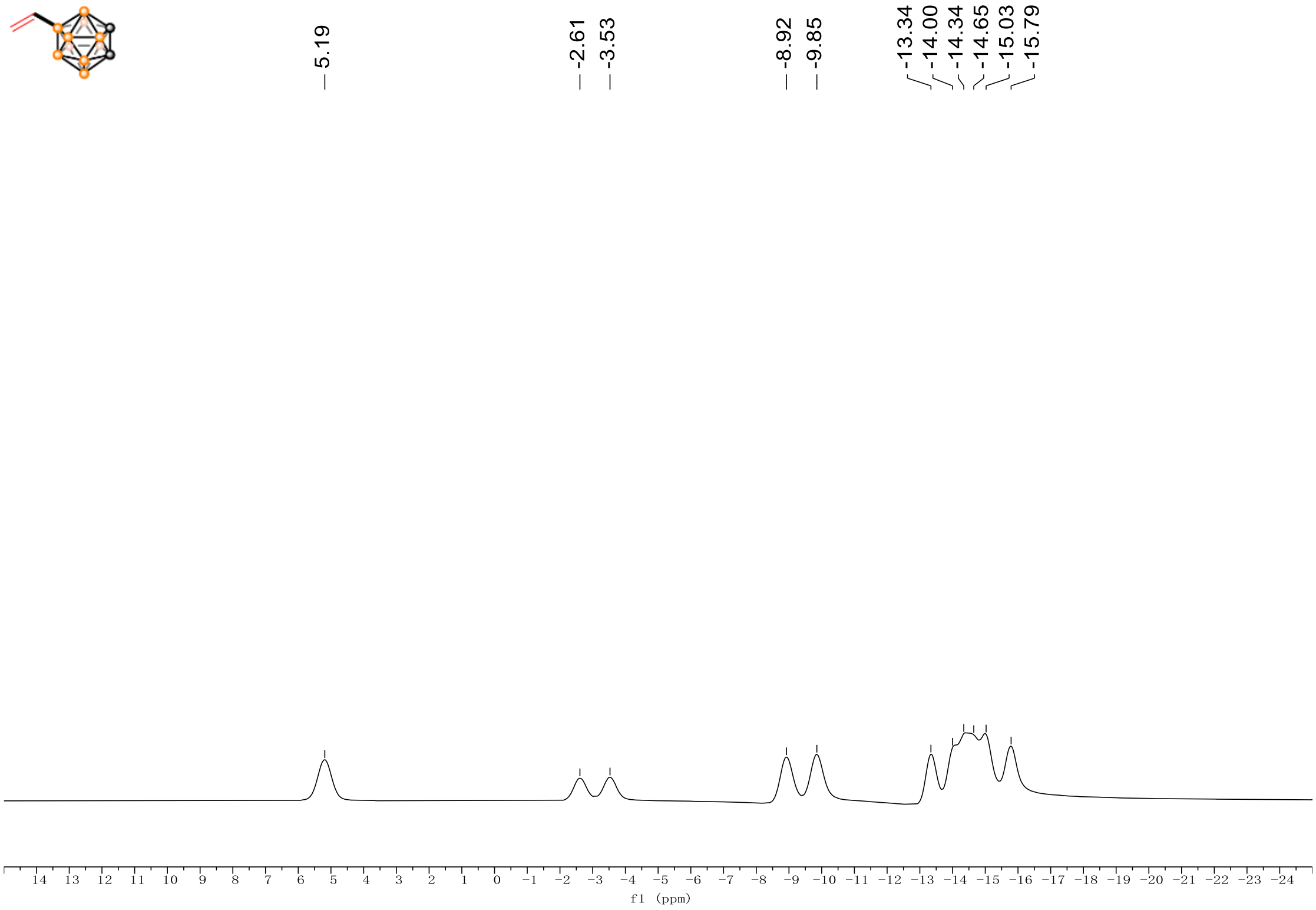

^{11}B NMR (160 MHz, CD_3CN) of **3an**

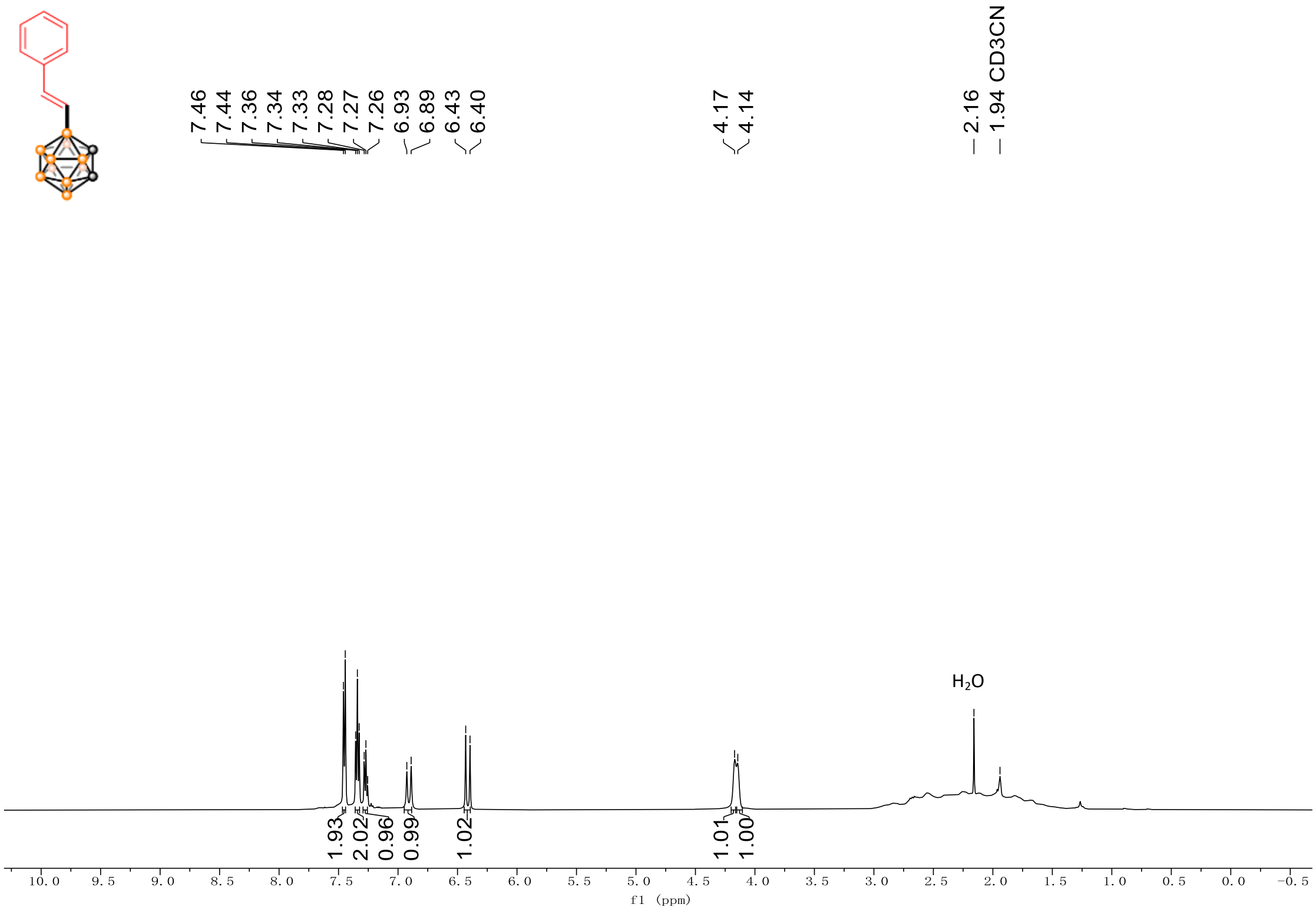

^{1}H NMR (500 MHz, CD_3CN) of **3ba**

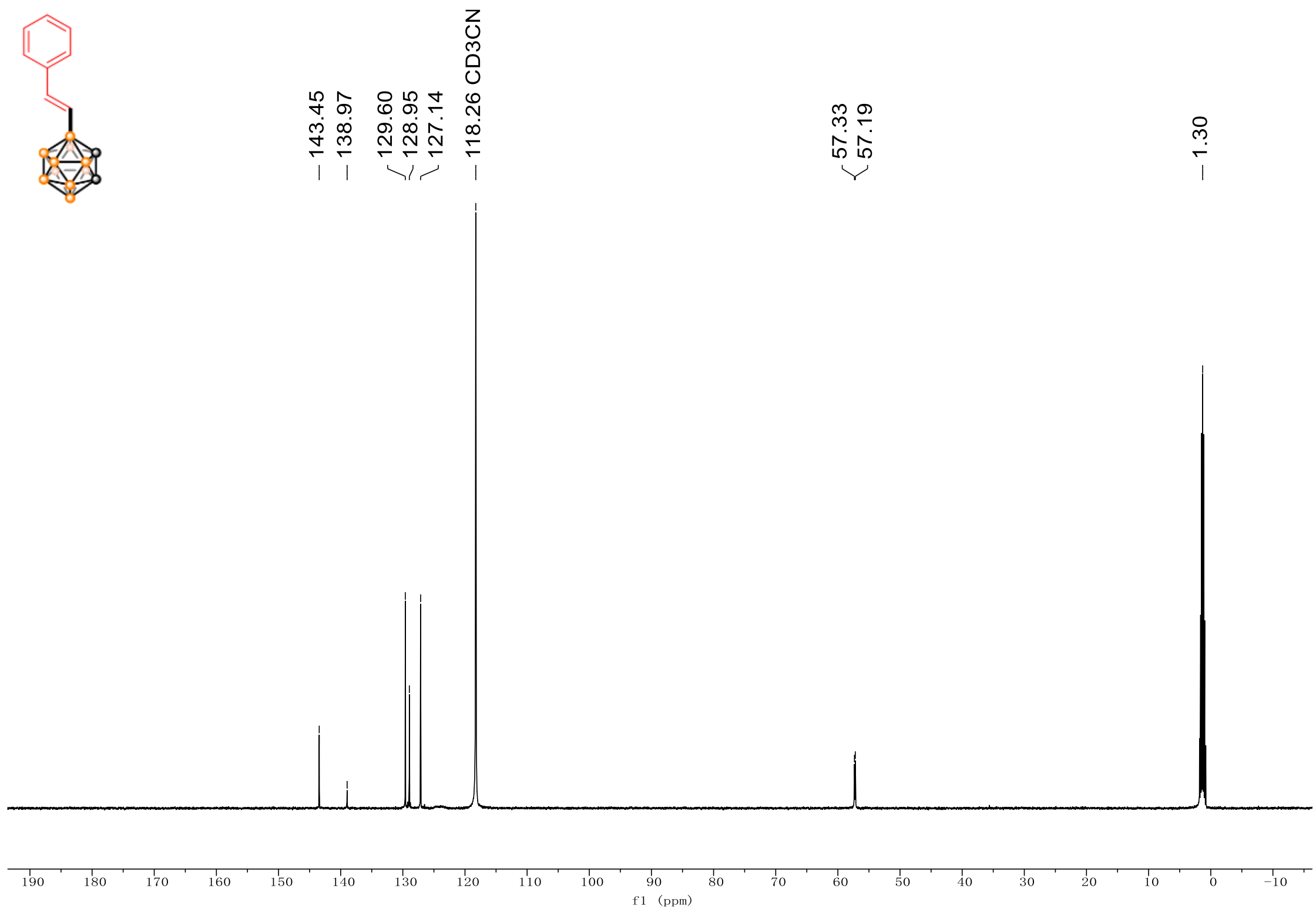

$^{13}C\{^{1}H\}$ NMR (125 MHz, CD_3CN) of **3ba**

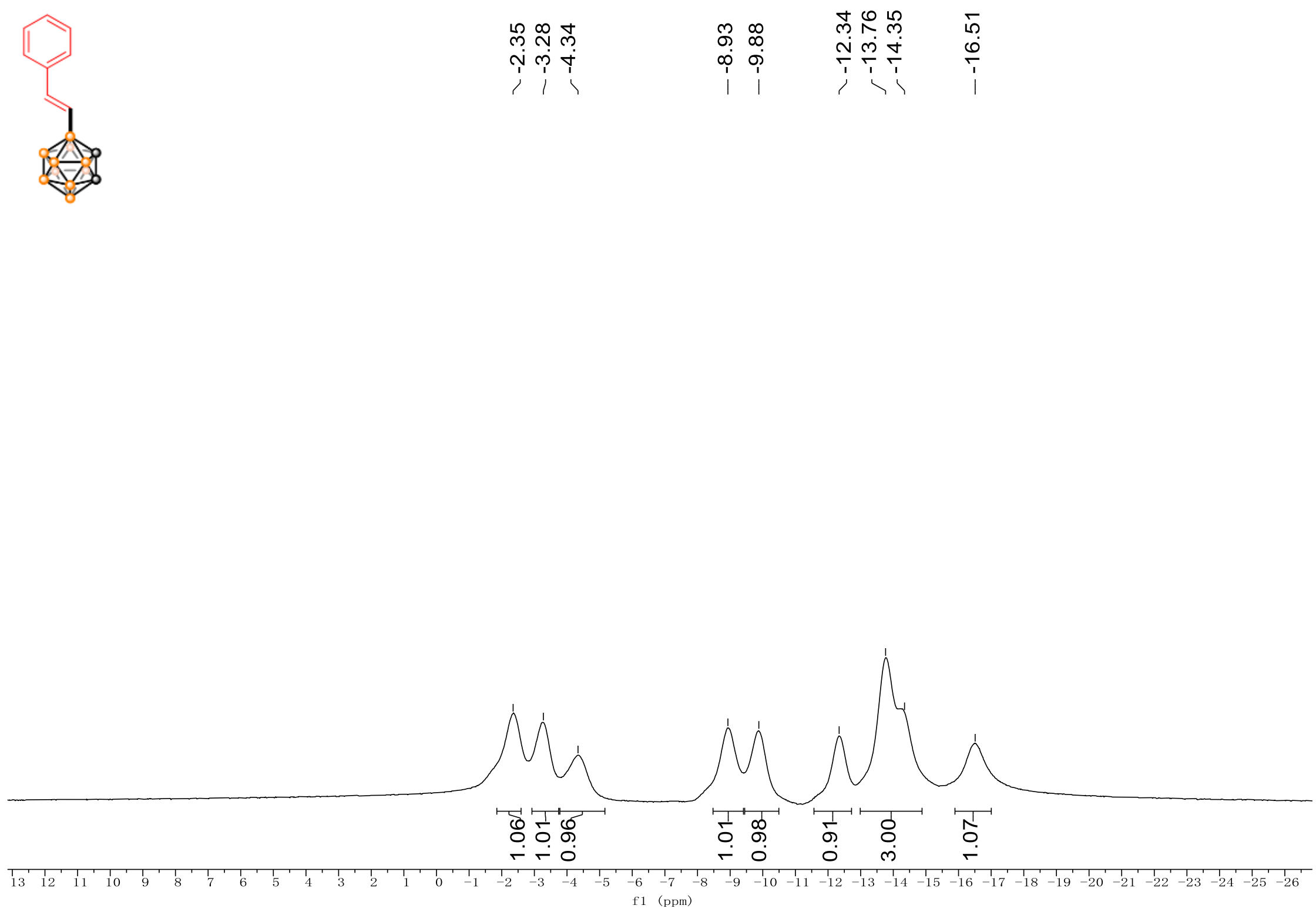

$^{11}B\{^{1}H\}$ NMR (160 MHz, CD_3CN) of **3ba**

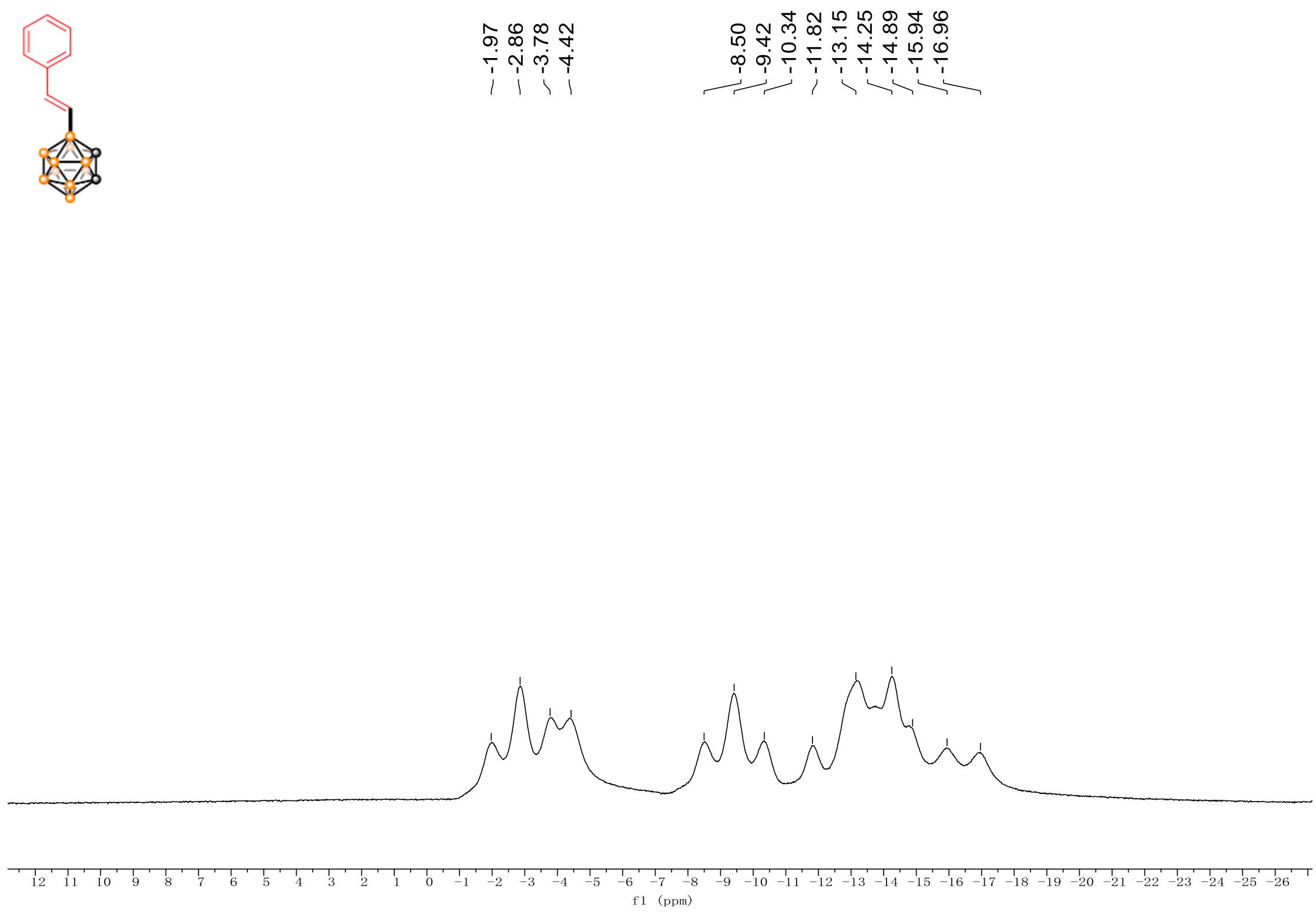

^{11}B NMR (160 MHz, CD_3CN) of **3ba**

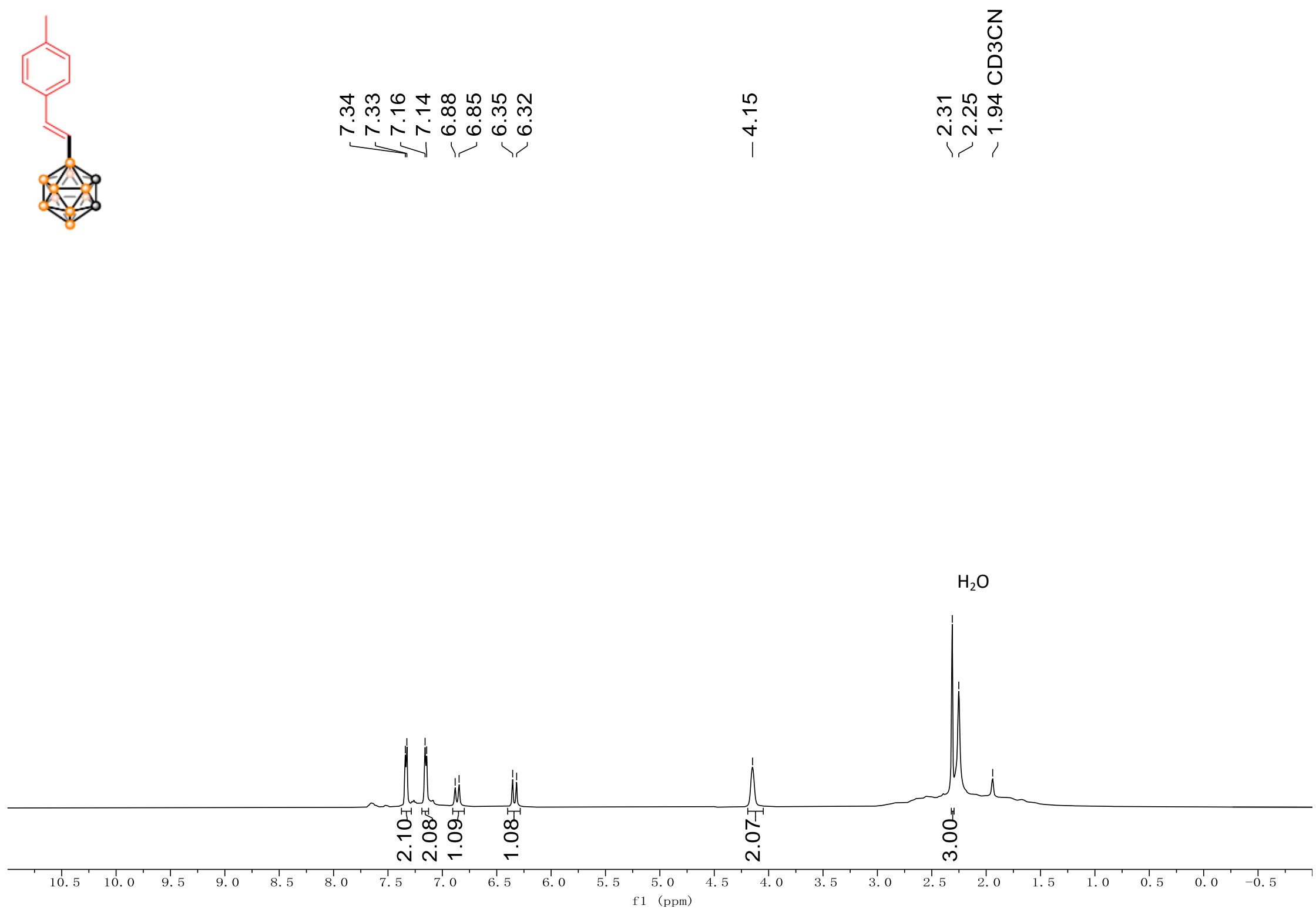

^{1}H NMR (500 MHz, CD_3CN) of **3bb**

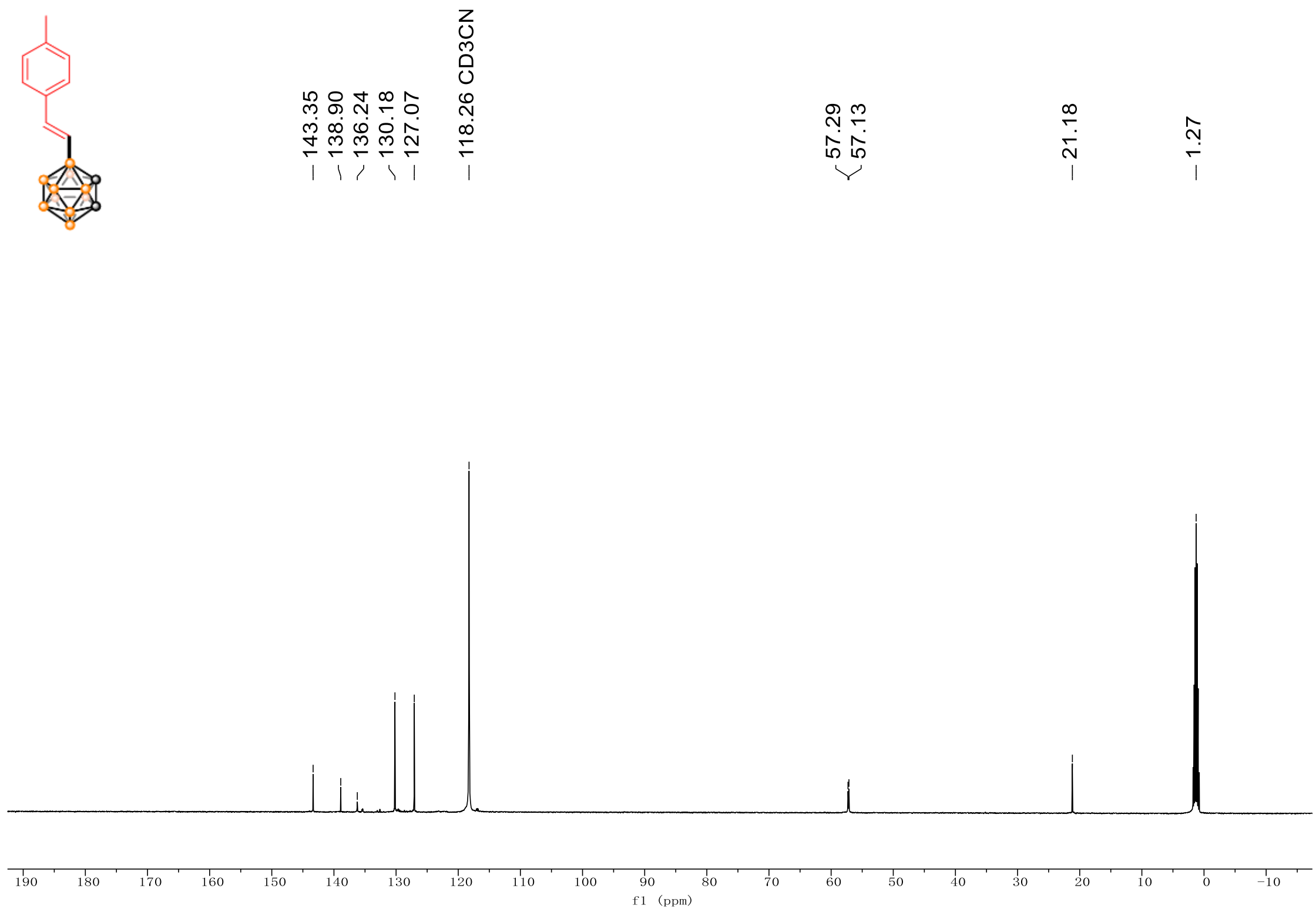

$^{13}C\{^{1}H\}$ NMR (125 MHz, CD_3CN) of **3bb**

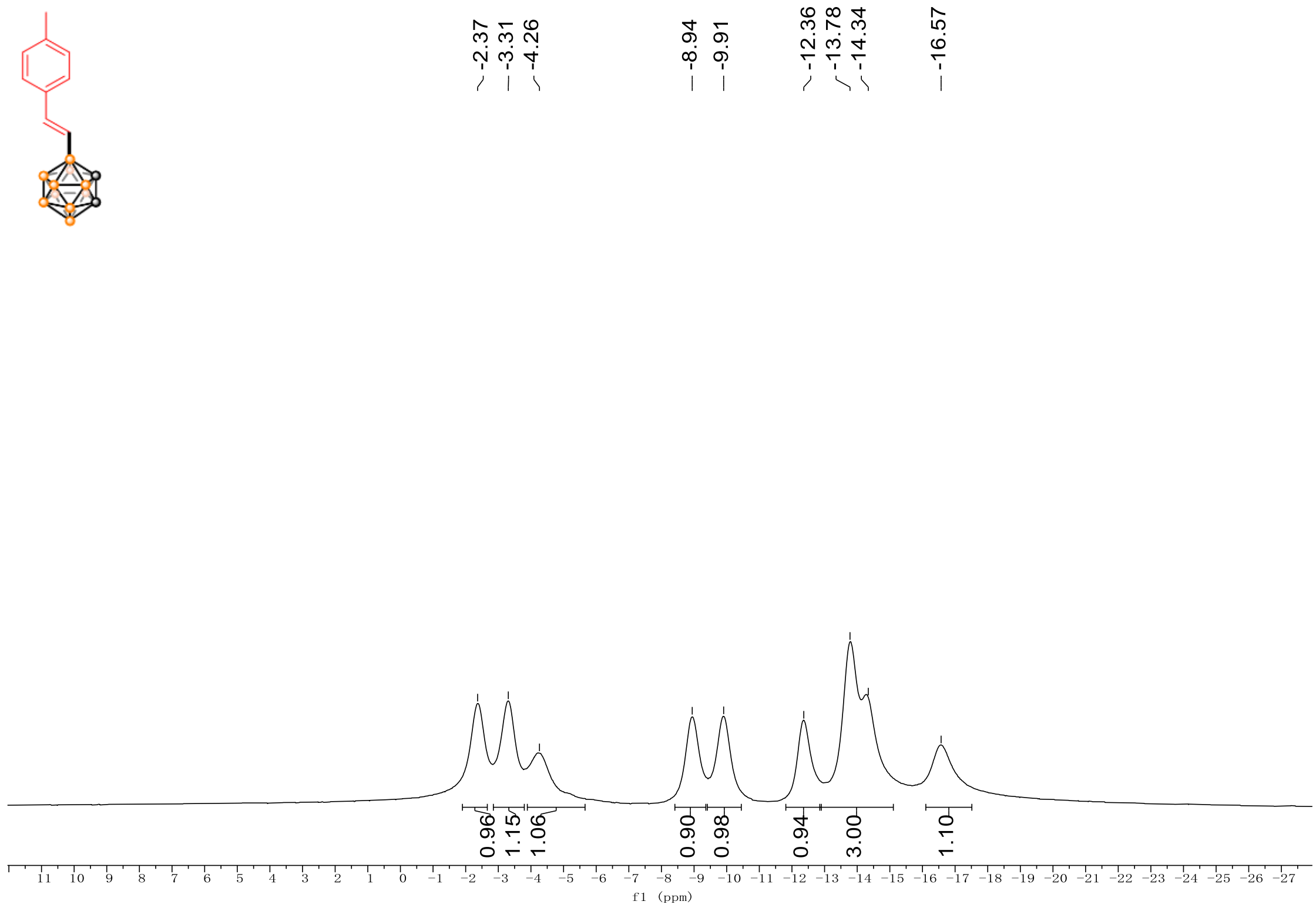

$^{11}B\{^{1}H\}$ NMR (160 MHz, CD_3CN) of **3bb**

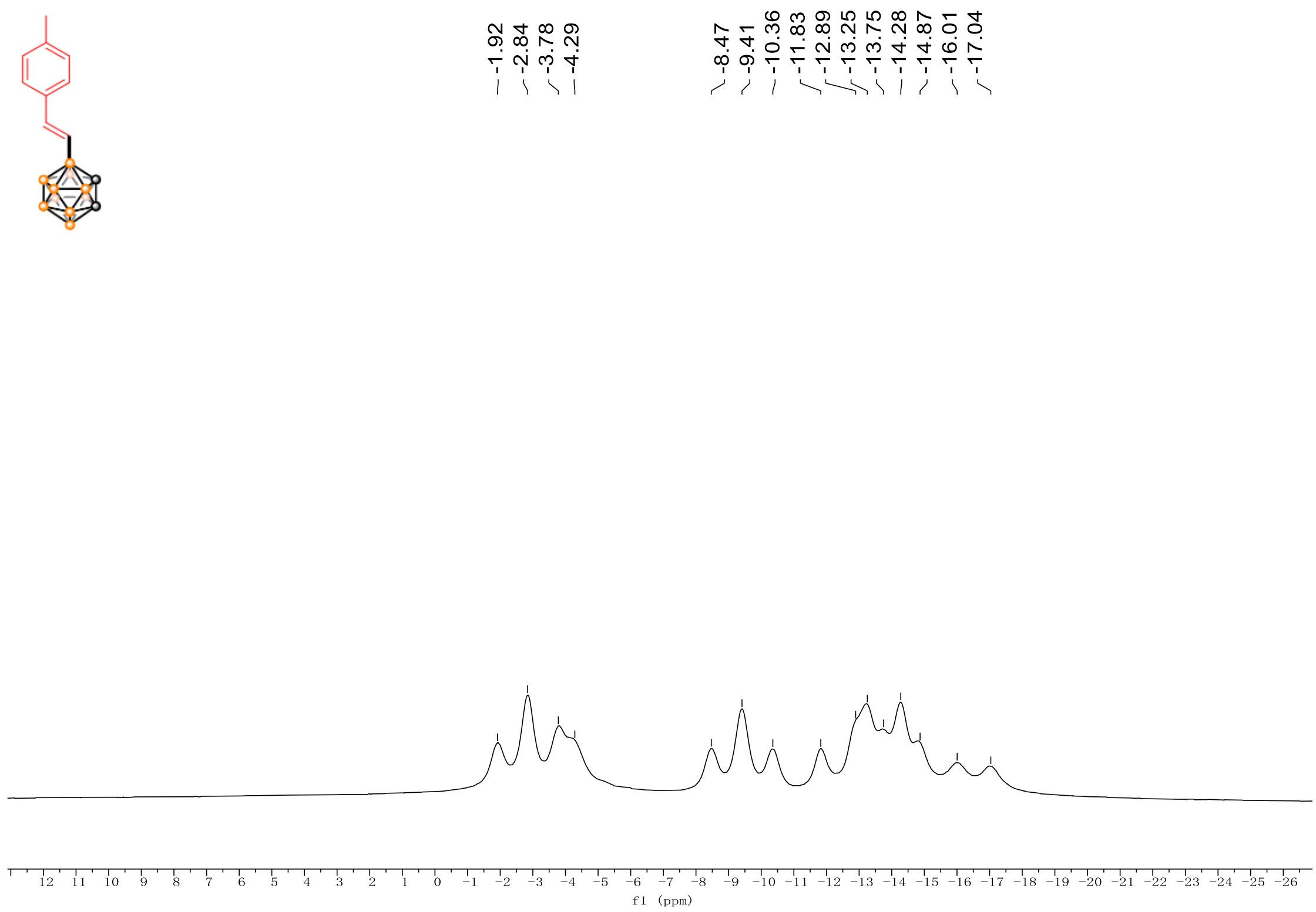

^{11}B NMR (160 MHz, CD_3CN) of **3bb**

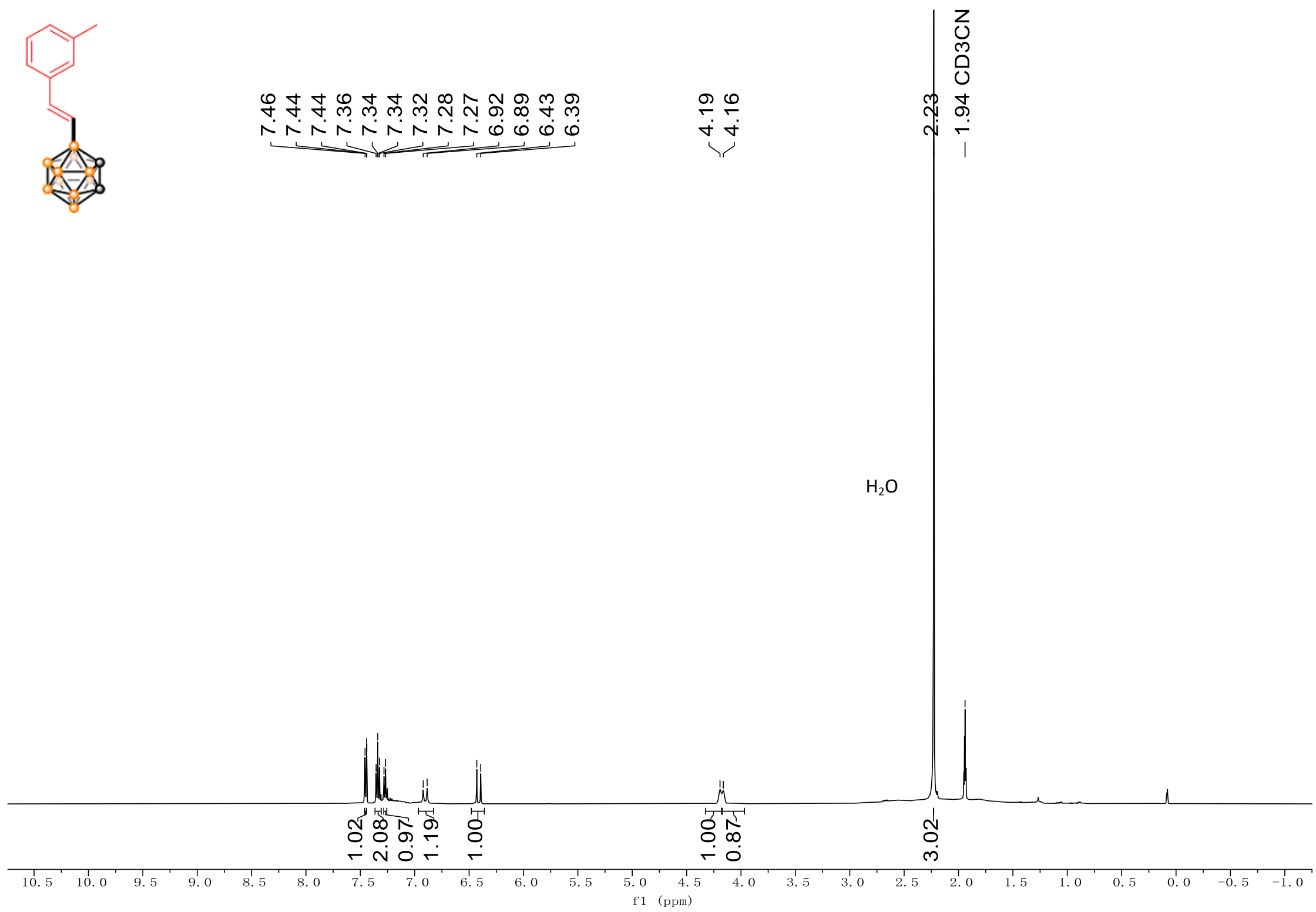

^{1}H NMR (500 MHz, CD_3CN) of **3bc**

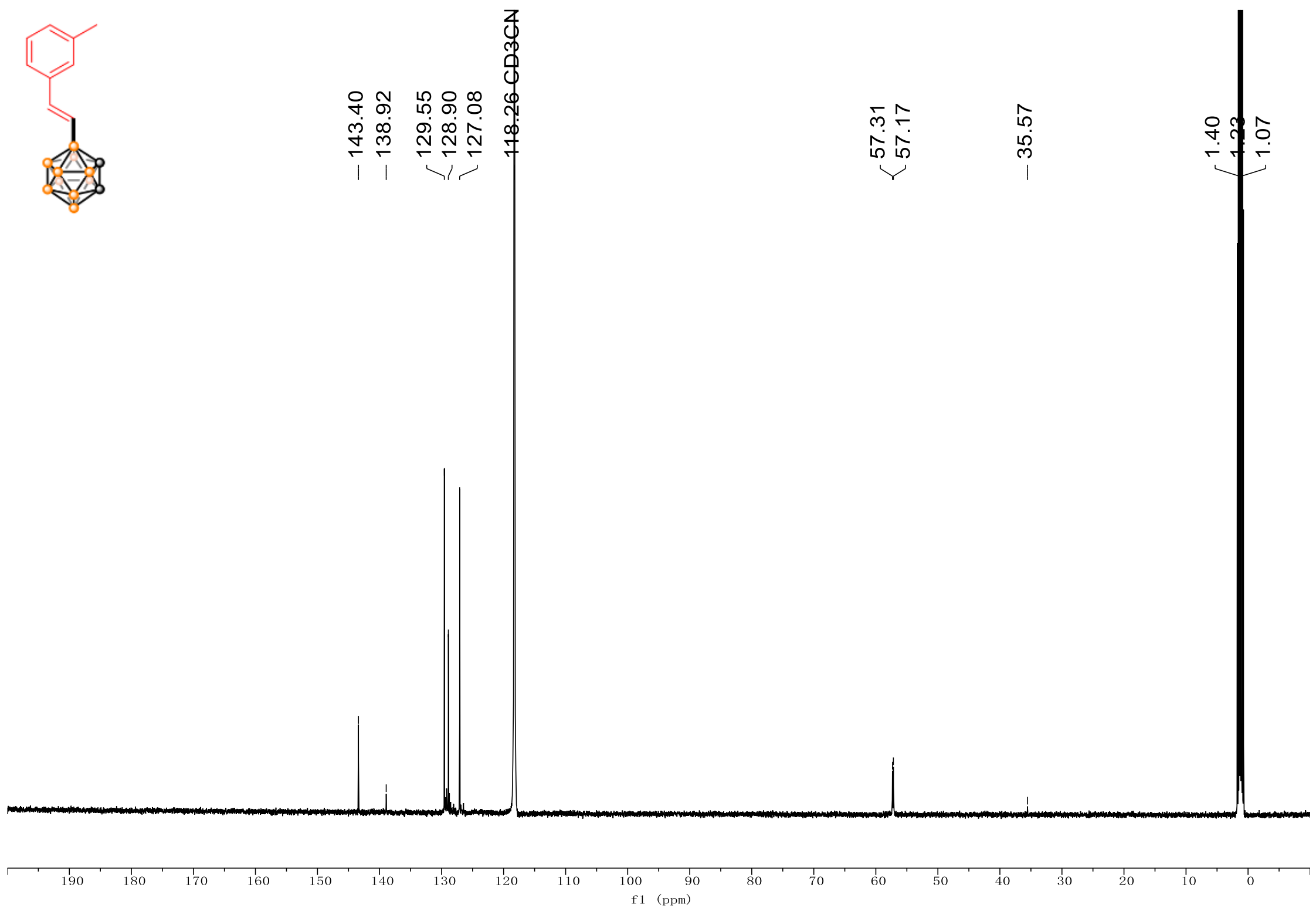

$^{13}C\{^{1}H\}$ NMR (125 MHz, CD_3CN) of **3bc**

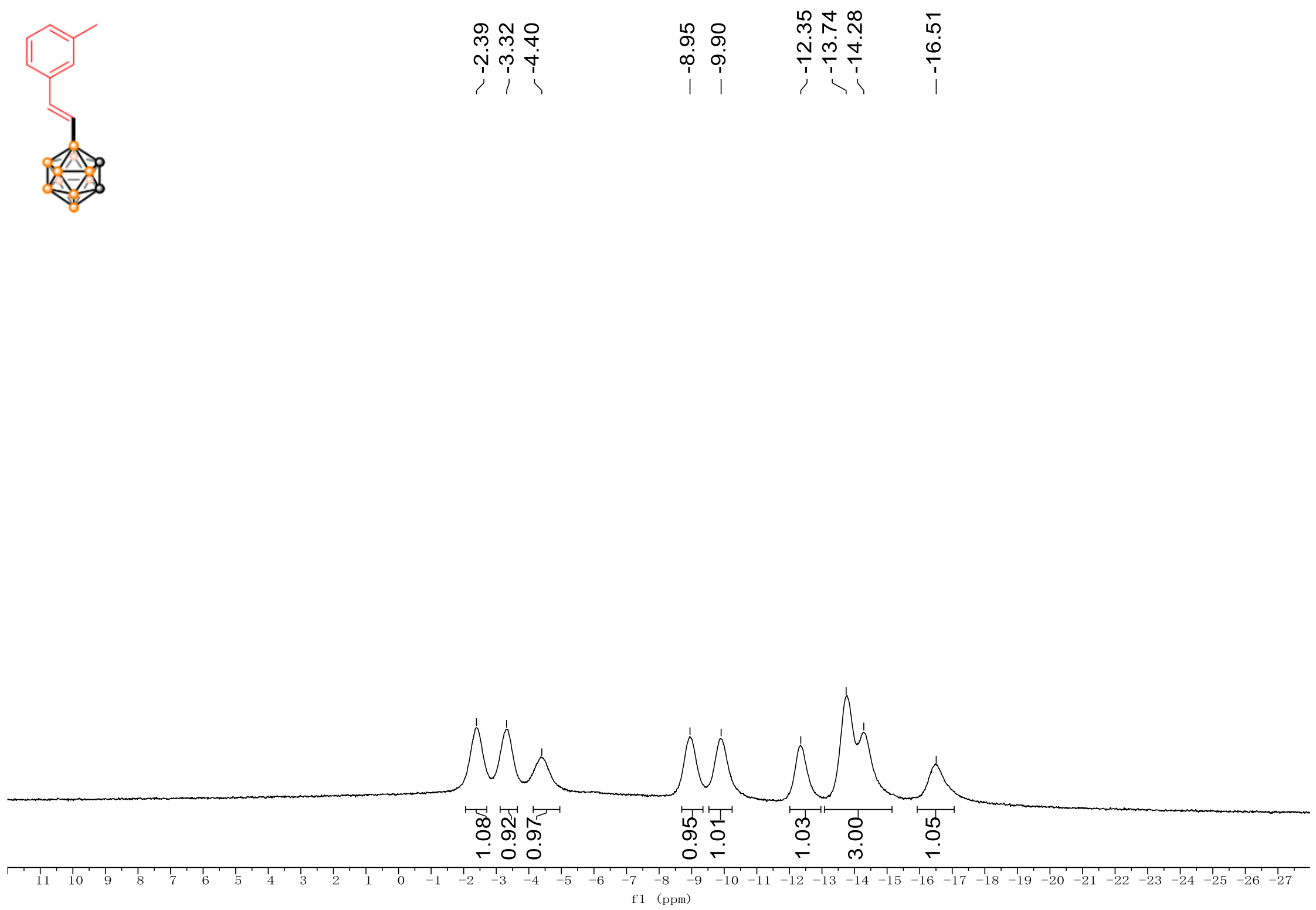

$^{11}B\{^{1}H\}$ NMR (160 MHz, CD_3CN) of **3bc**

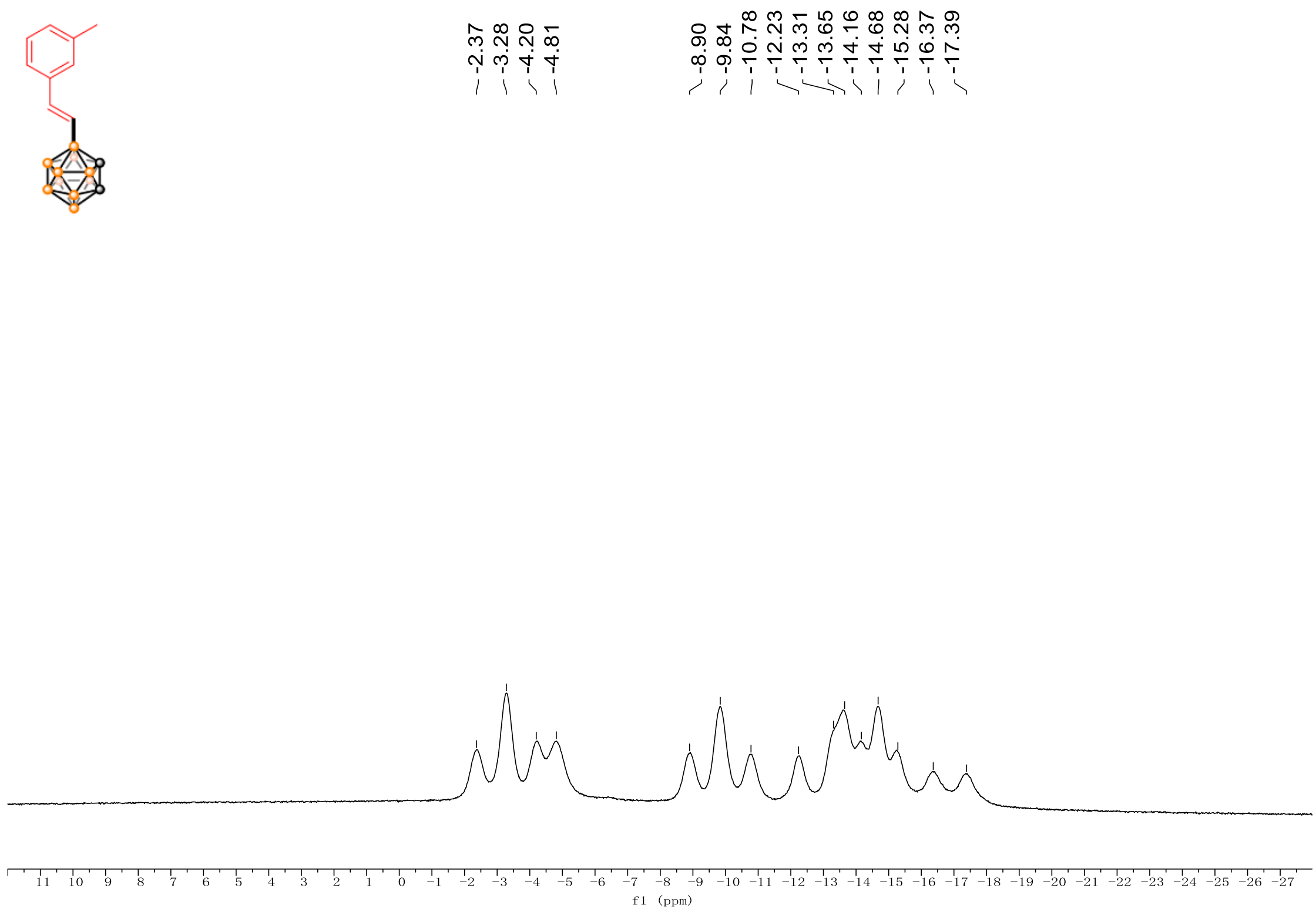

^{11}B NMR (160 MHz, CD_3CN) of **3bc**

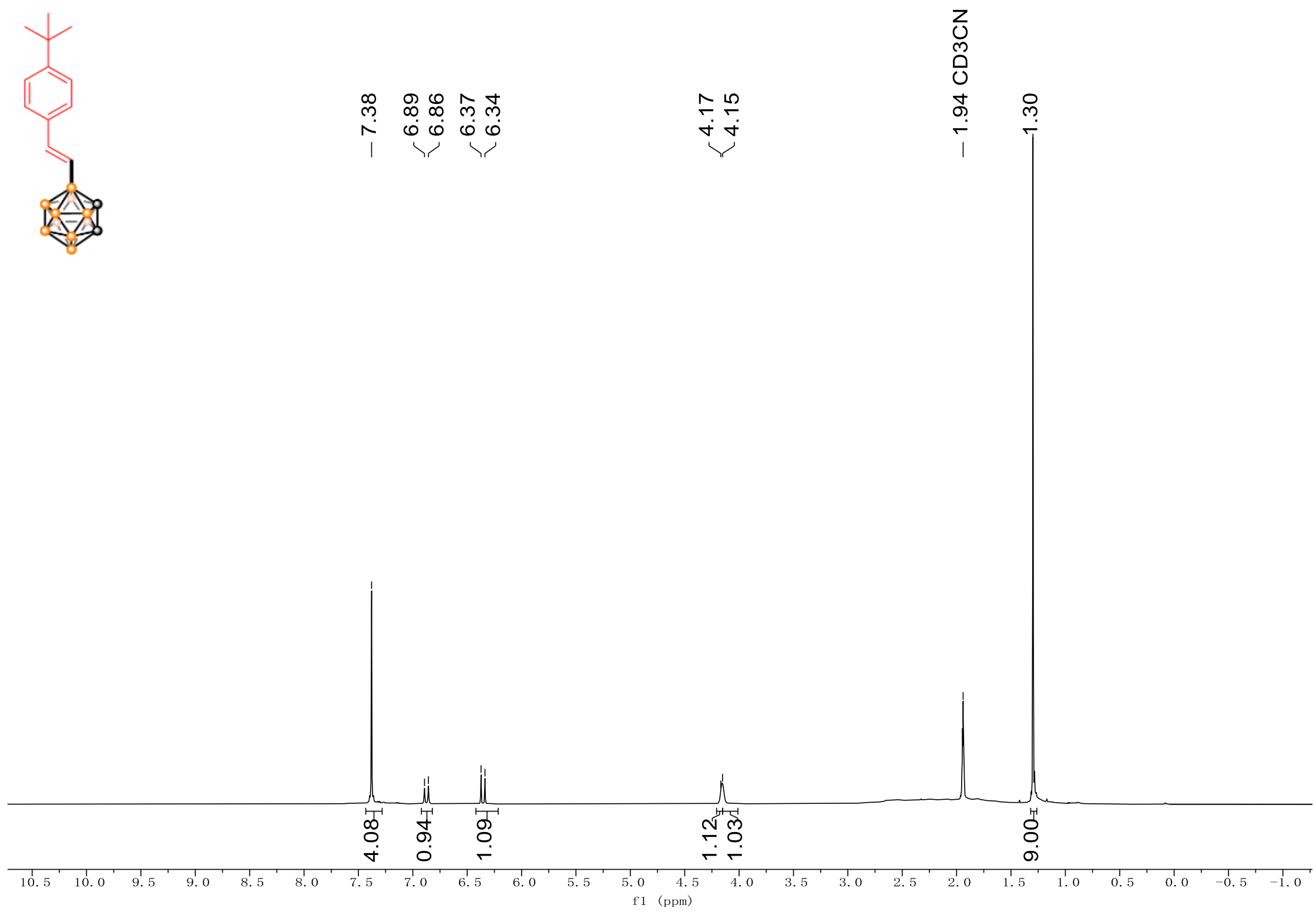

^{1}H NMR (500 MHz, CD_3CN) of **3be**

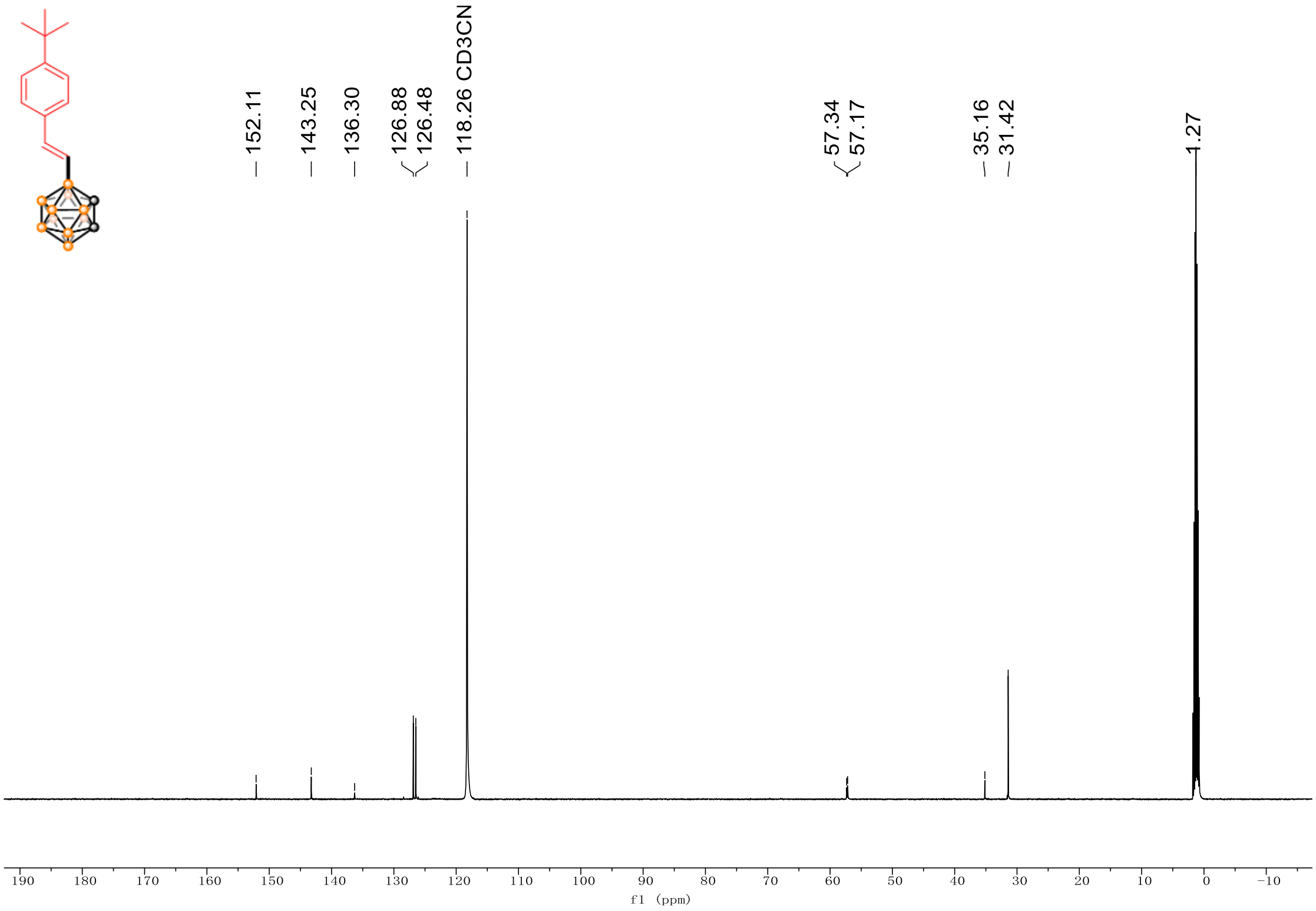

$^{13}C\{^{1}H\}$ NMR (125 MHz, CD_3CN) of **3be**

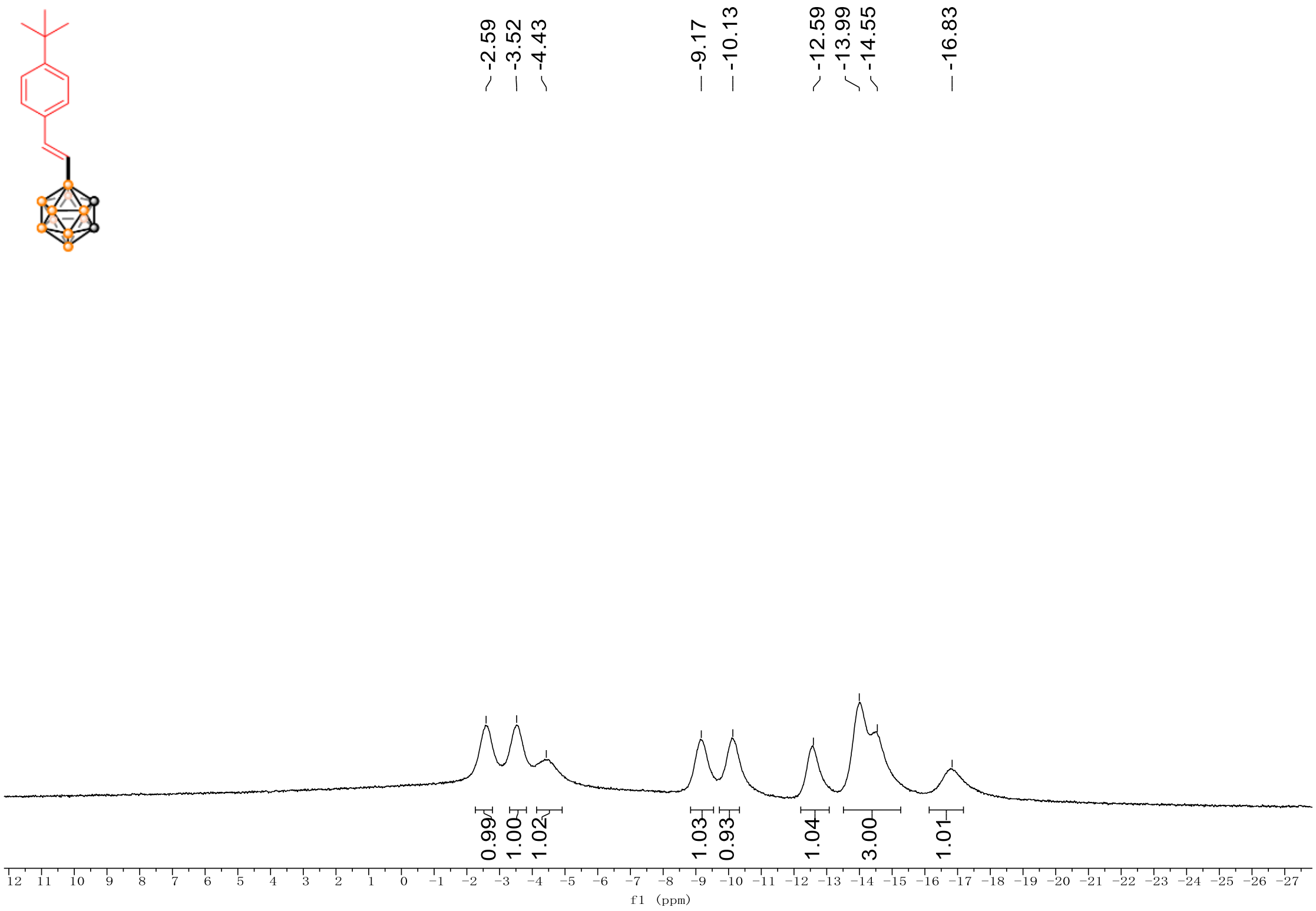

$^{11}B\{^{1}H\}$ NMR (160 MHz, CD_3CN) of **3be**

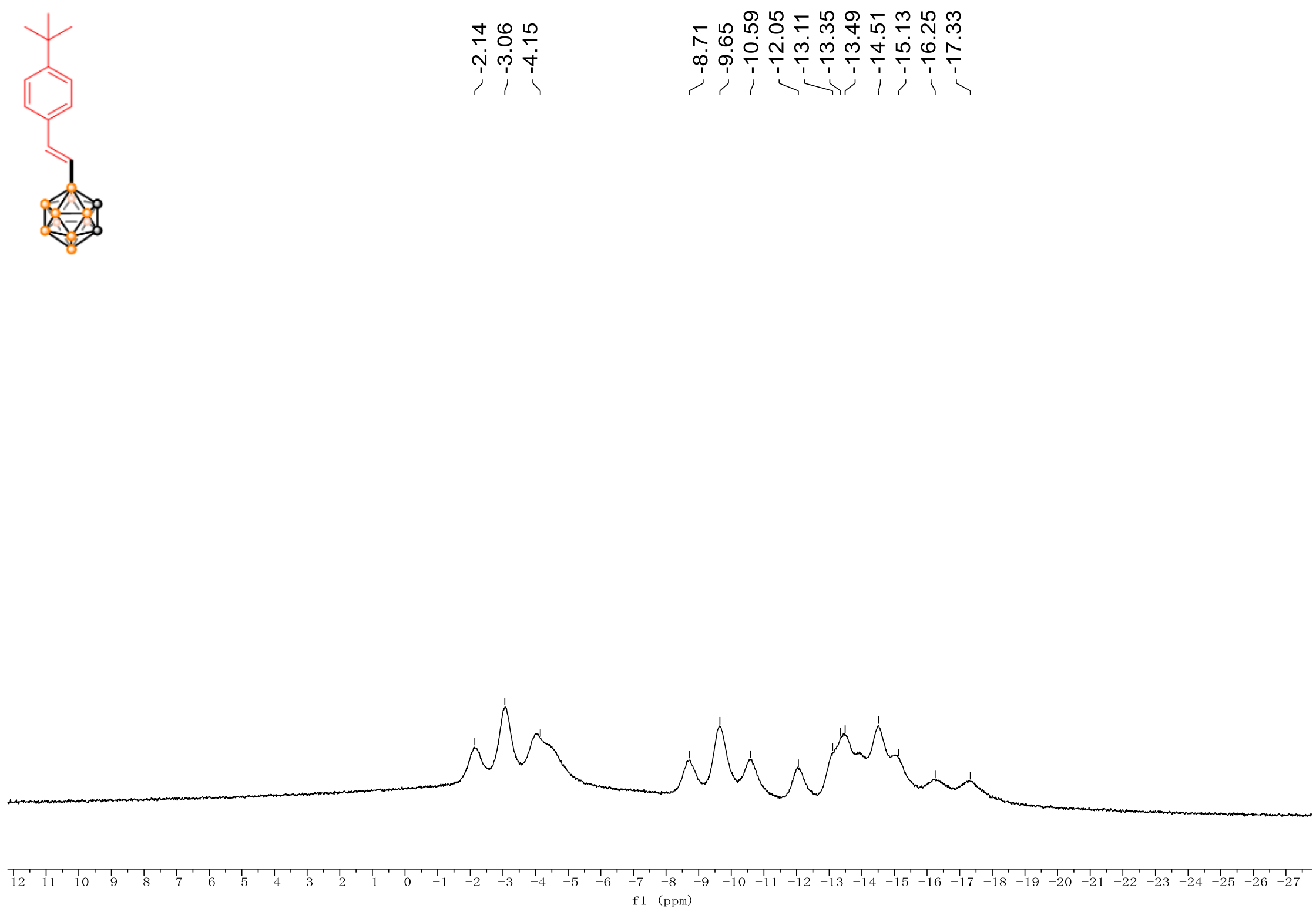

^{11}B NMR (160 MHz, CD_3CN) of **3be**

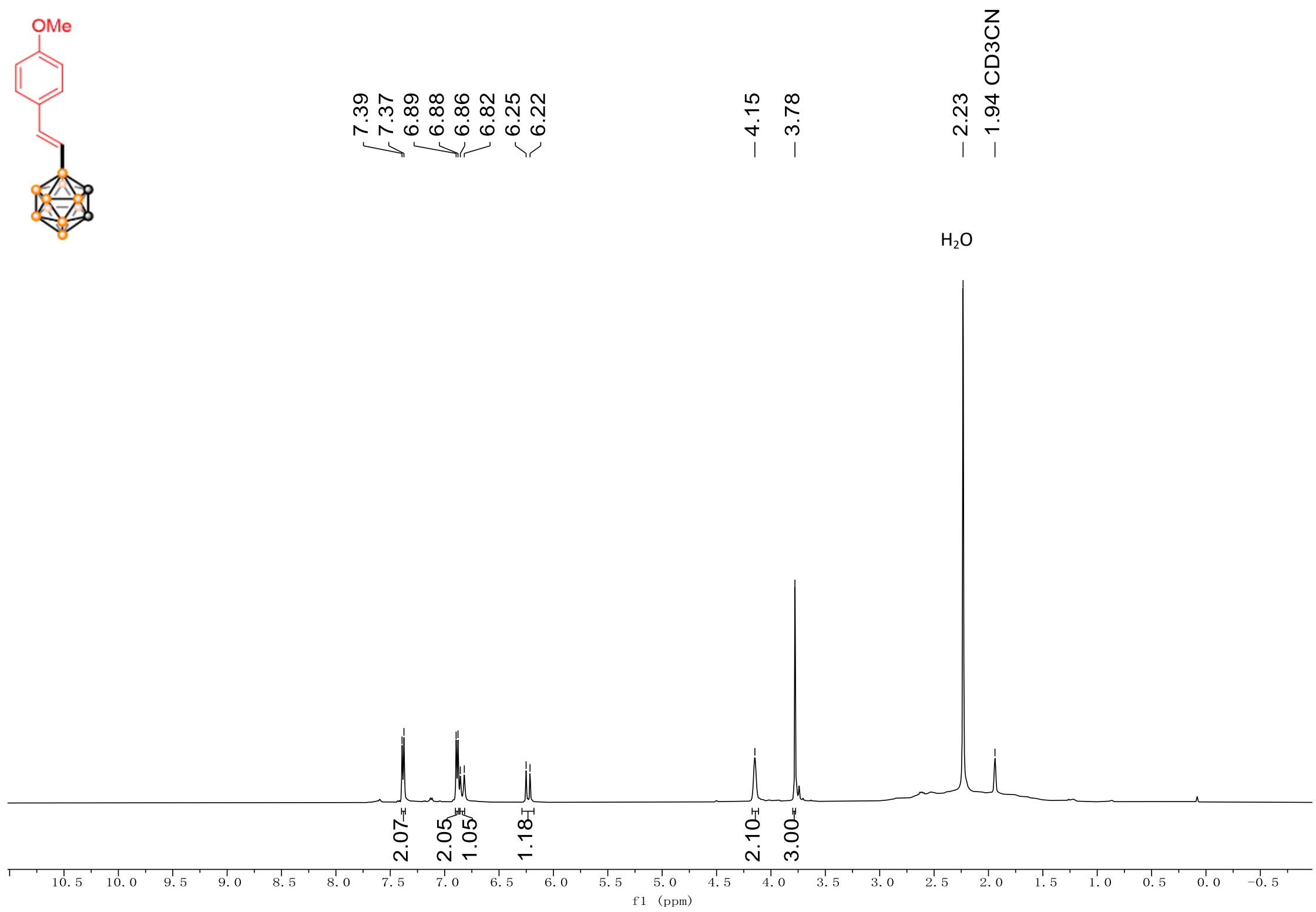

^{1}H NMR (500 MHz, CD_3CN) of **3bf**

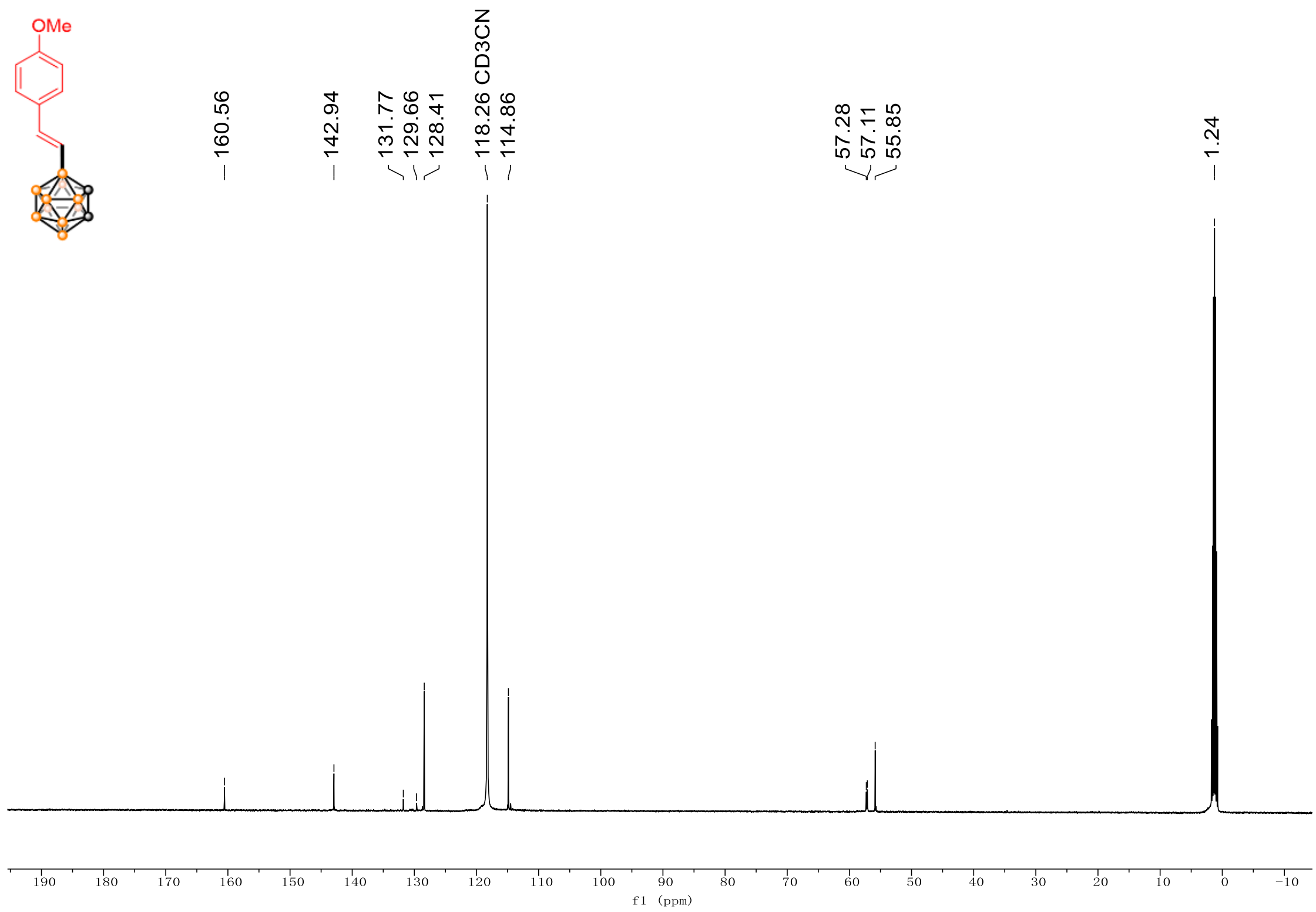

$^{13}C\{^1H\}$ NMR (125 MHz, CD_3CN) of **3bf**

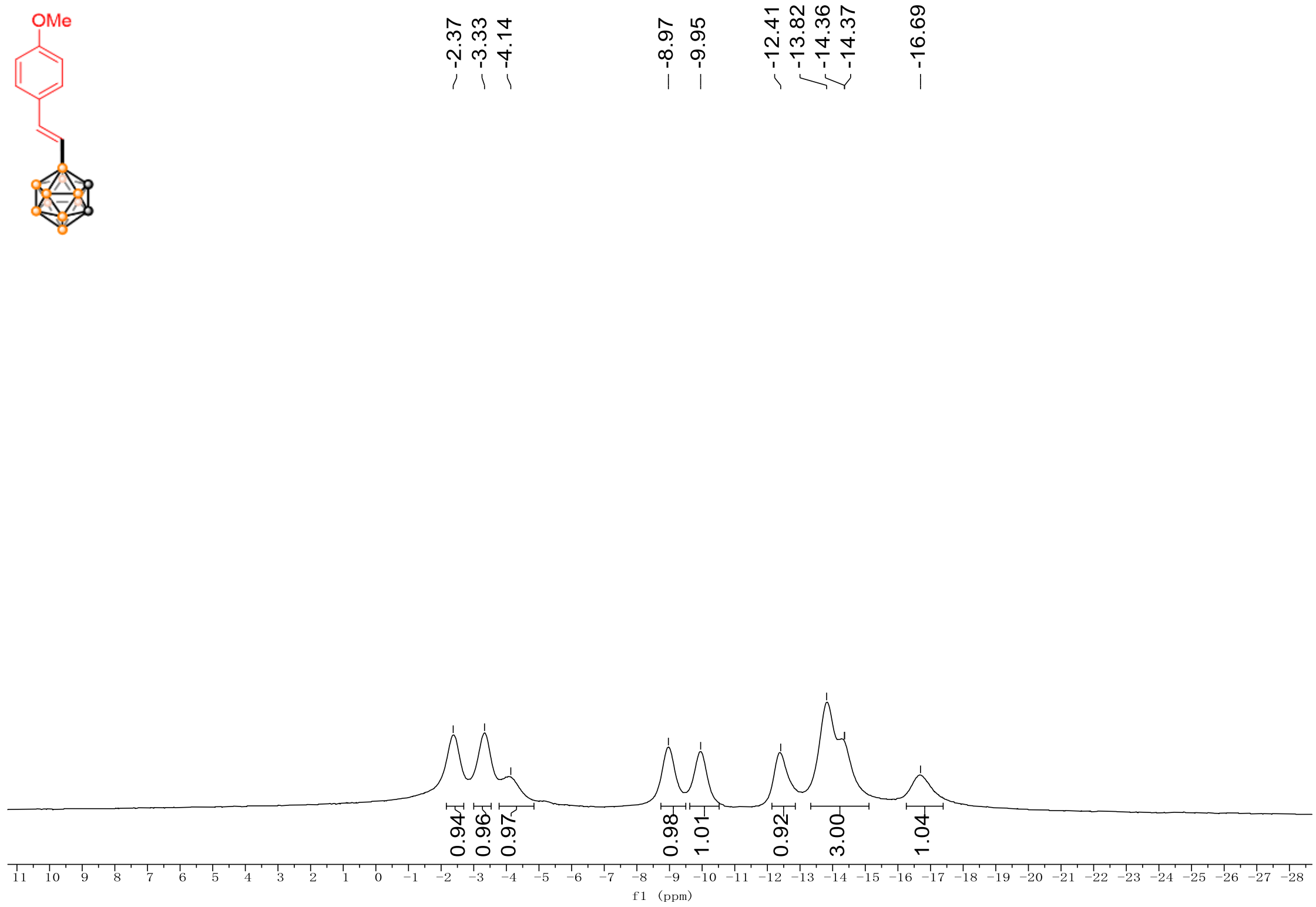

$^{11}B\{^1H\}$ NMR (160 MHz, CD_3CN) of **3bf**

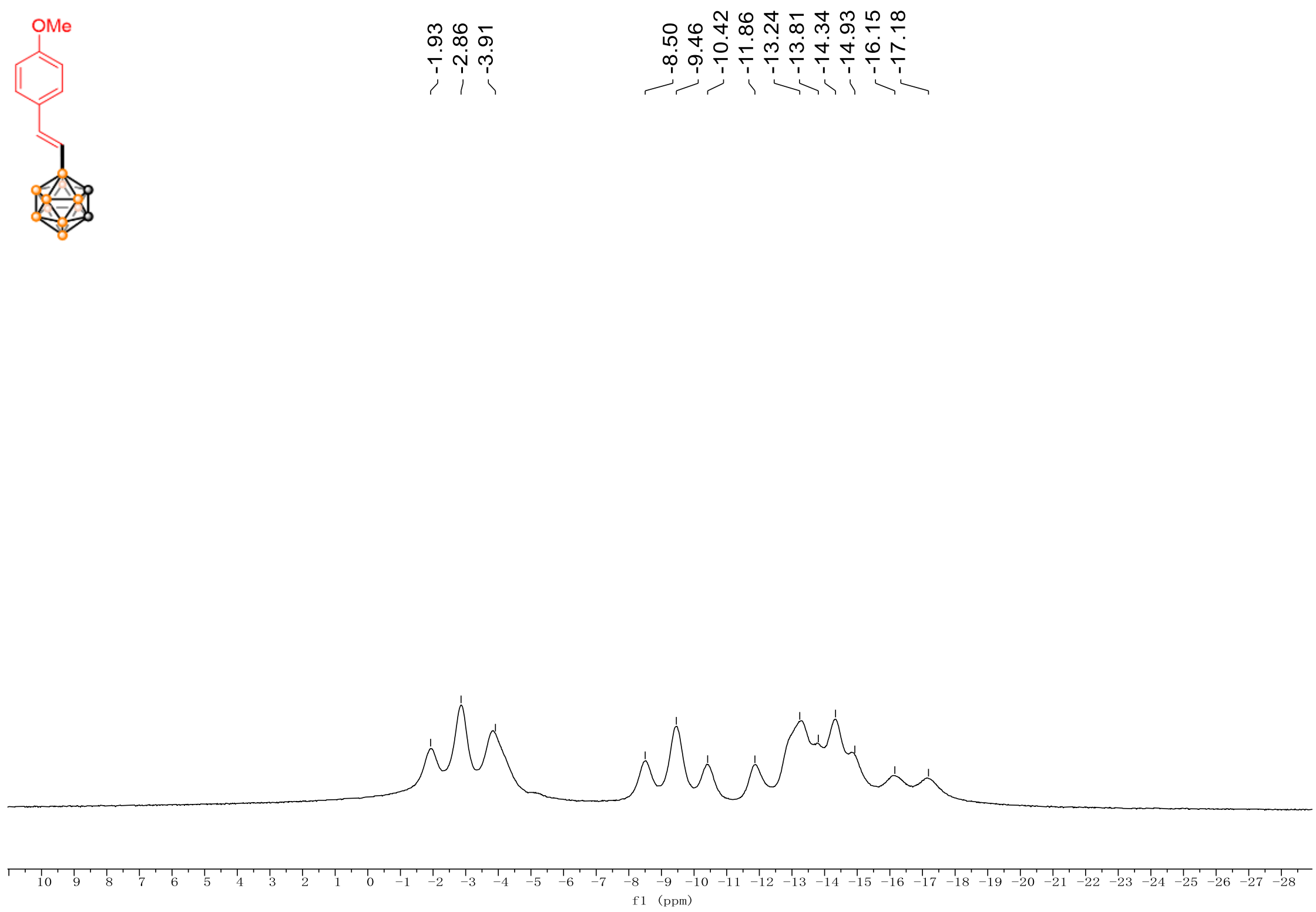

^{11}B NMR (160 MHz, CD_3CN) of **3bf**

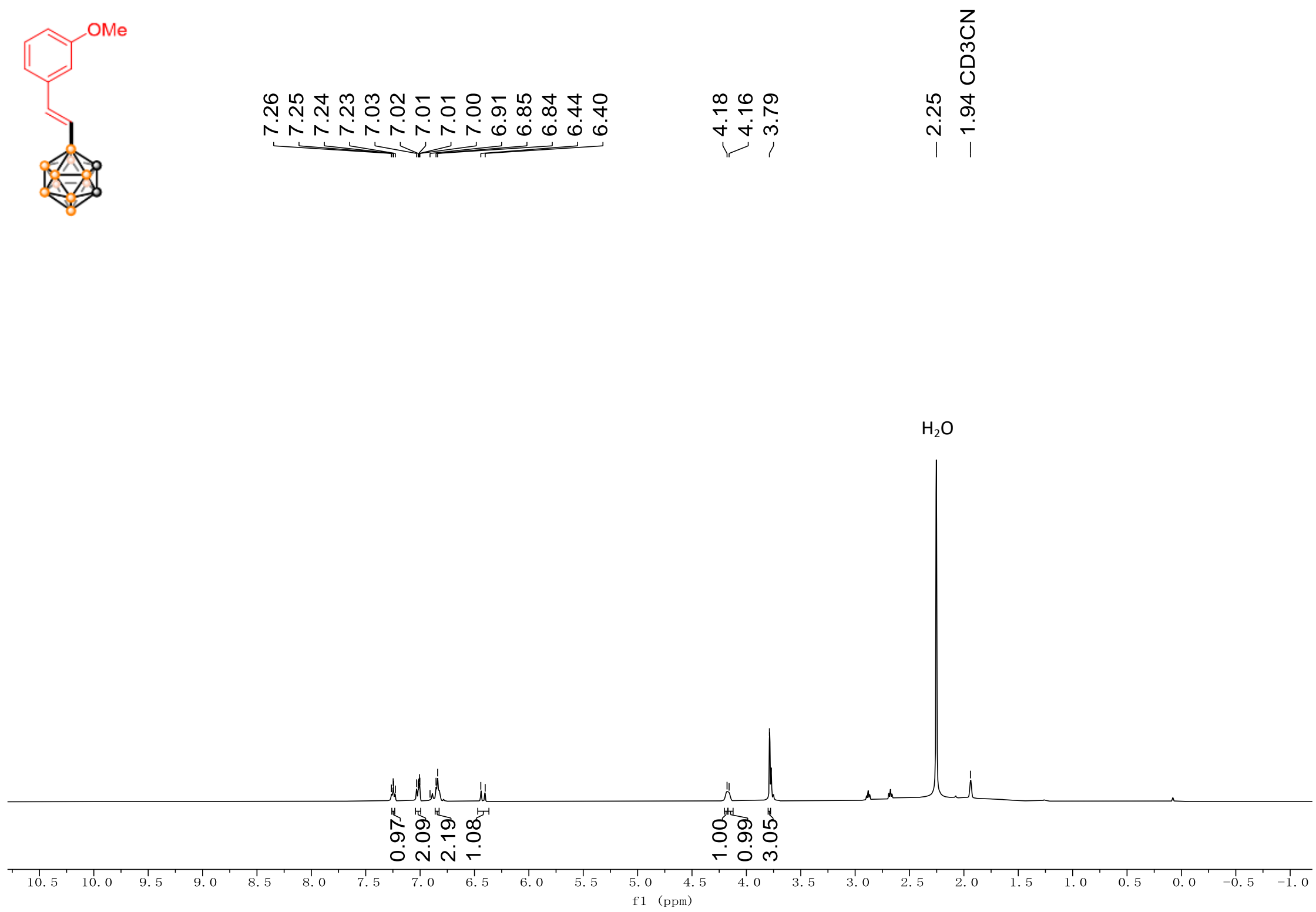

^{1}H NMR (500 MHz, CD_3CN) of **3bg**

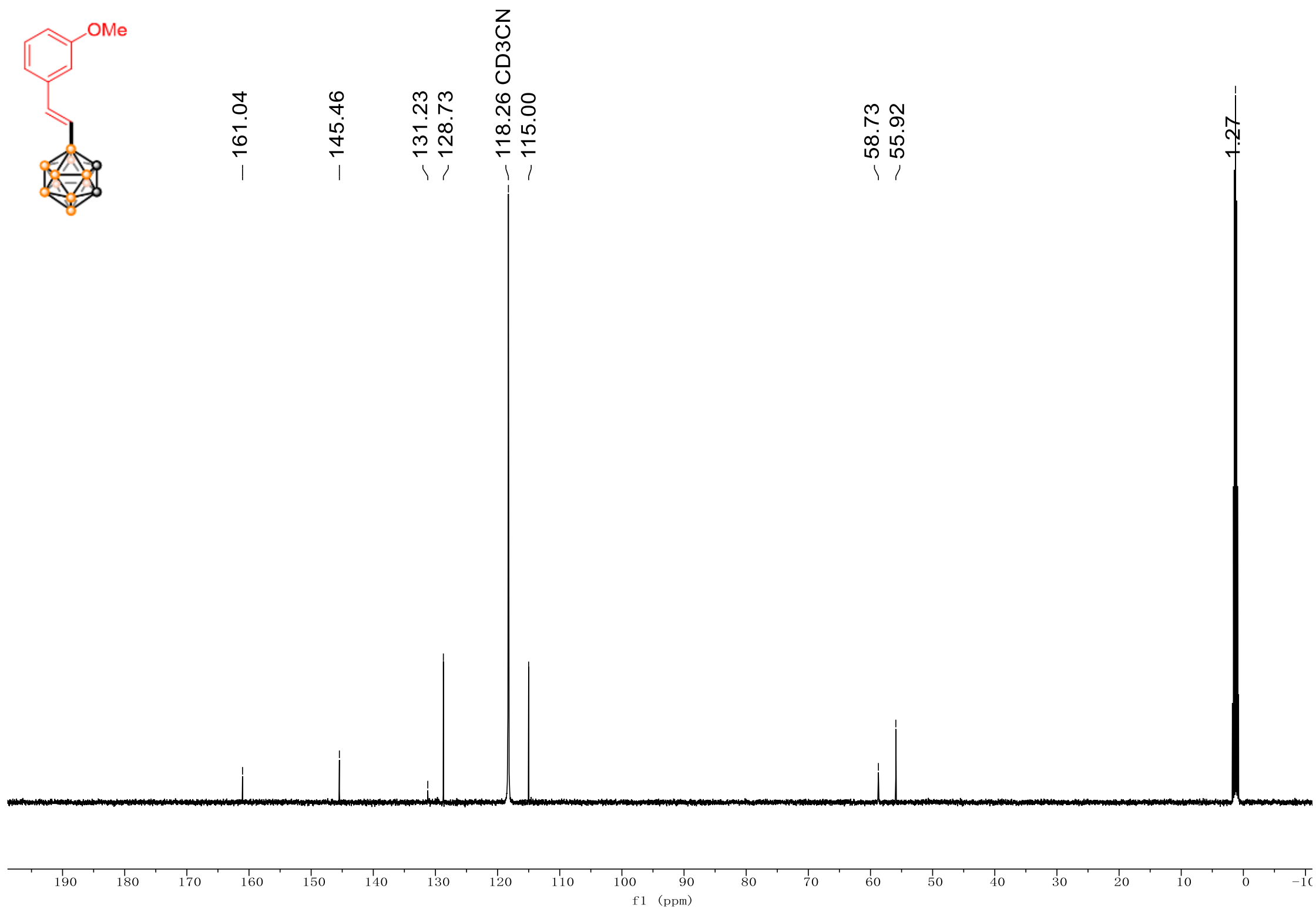

$^{13}C\{^{1}H\}$ NMR (125 MHz, CD_3CN) of **3bg**

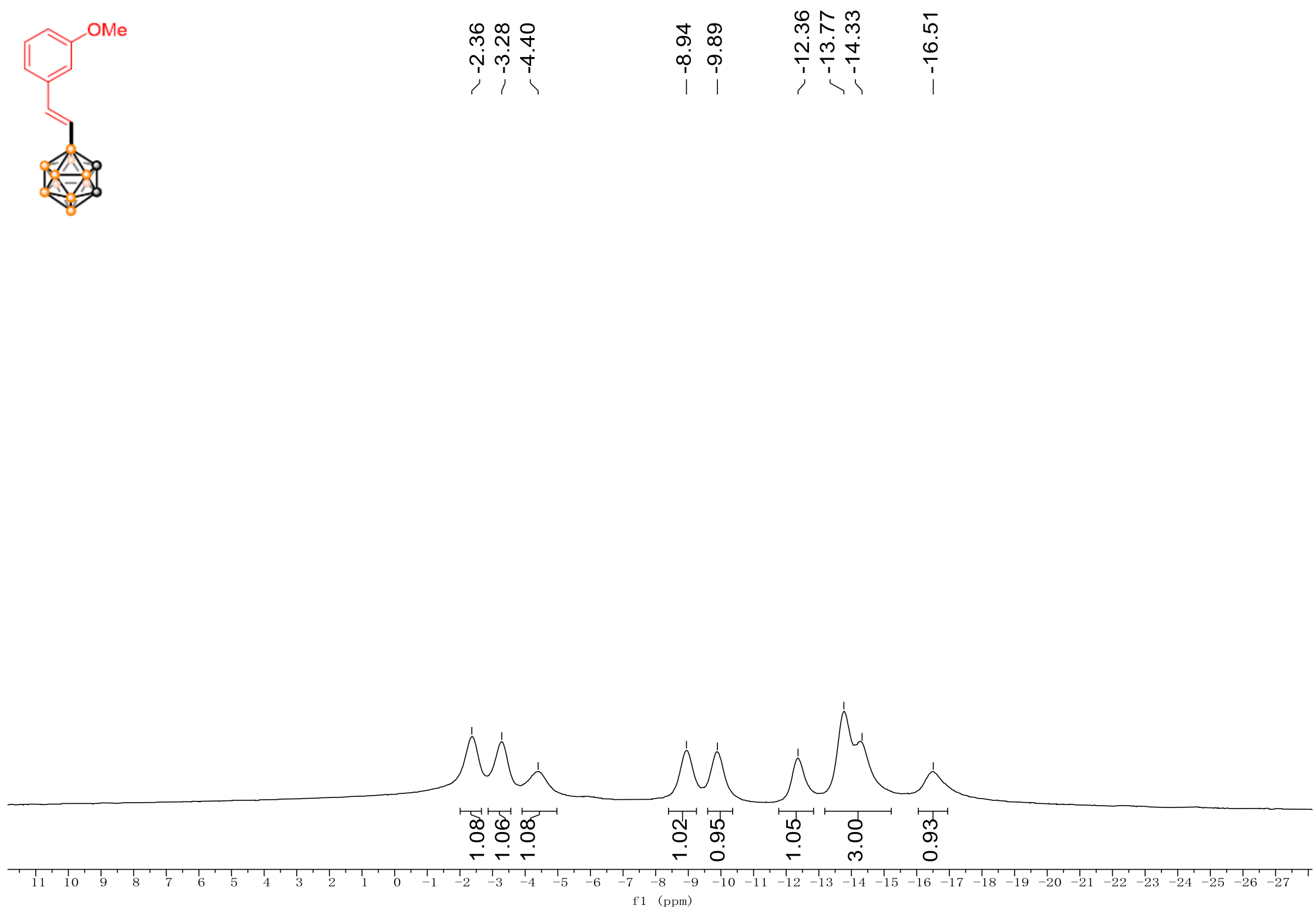

$^{11}B\{^{1}H\}$ NMR (160 MHz, CD_3CN) of **3bg**

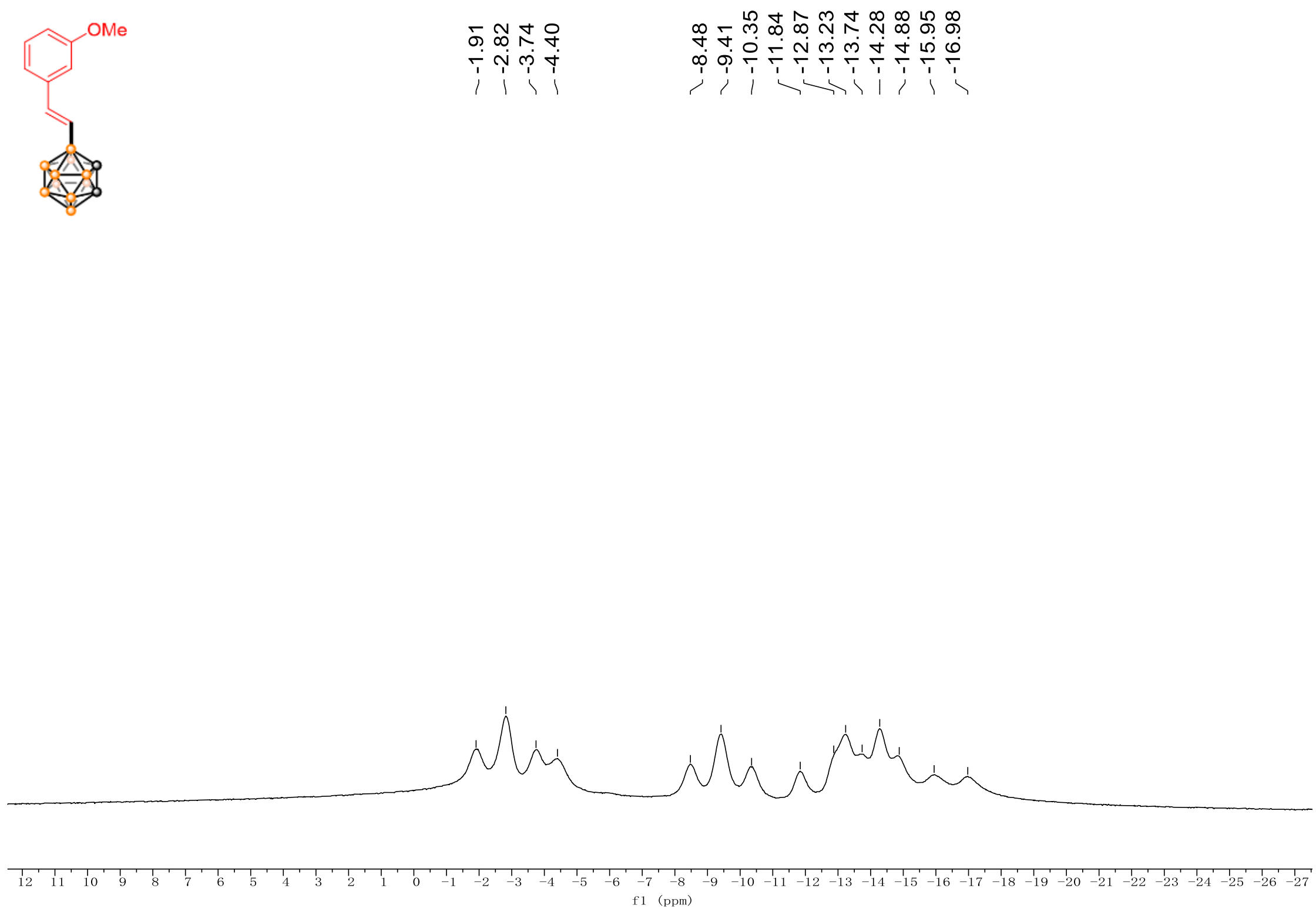

^{11}B NMR (160 MHz, CD_3CN) of **3bg**

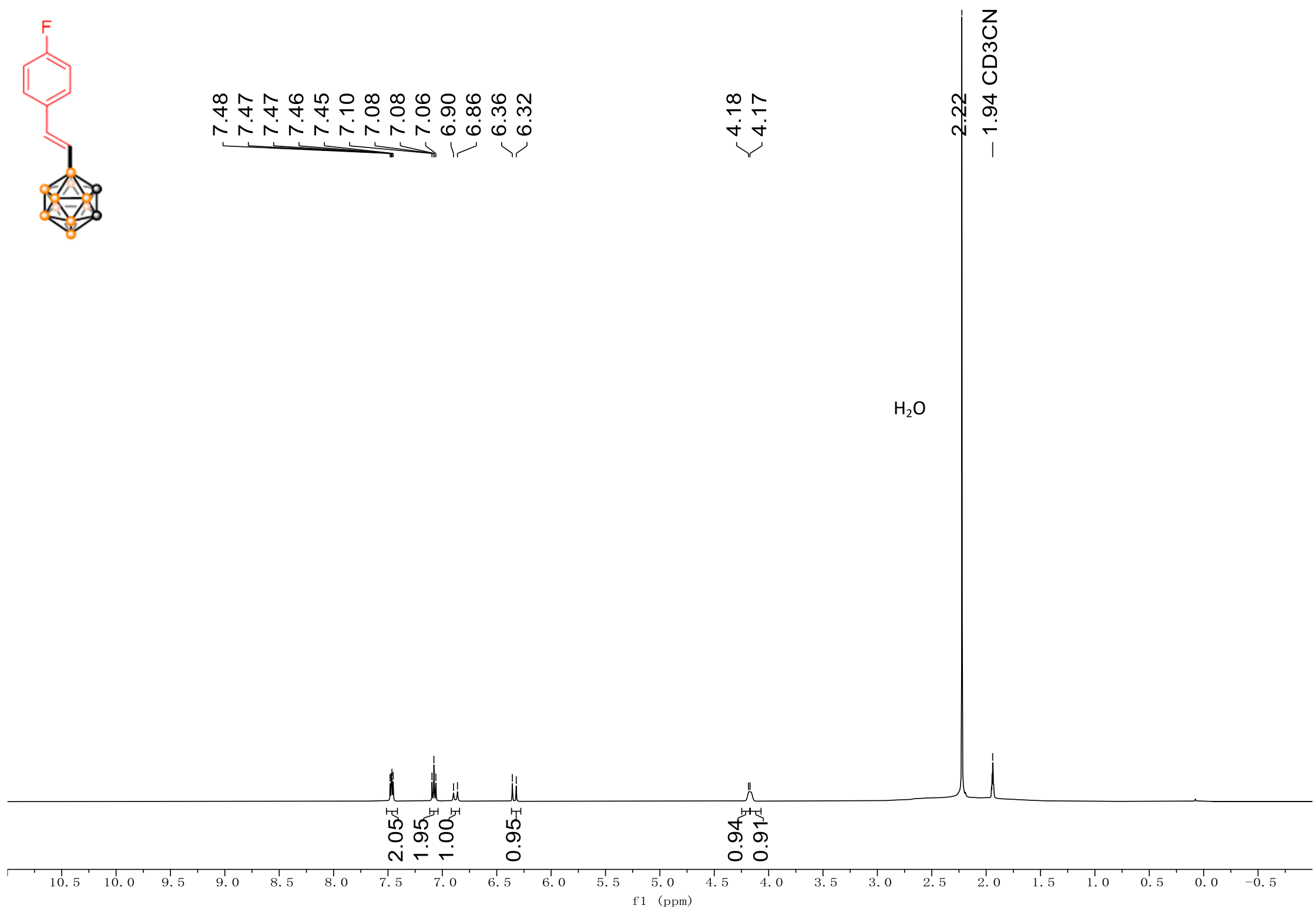

^{1}H NMR (500 MHz, CD_3CN) of **3bi**

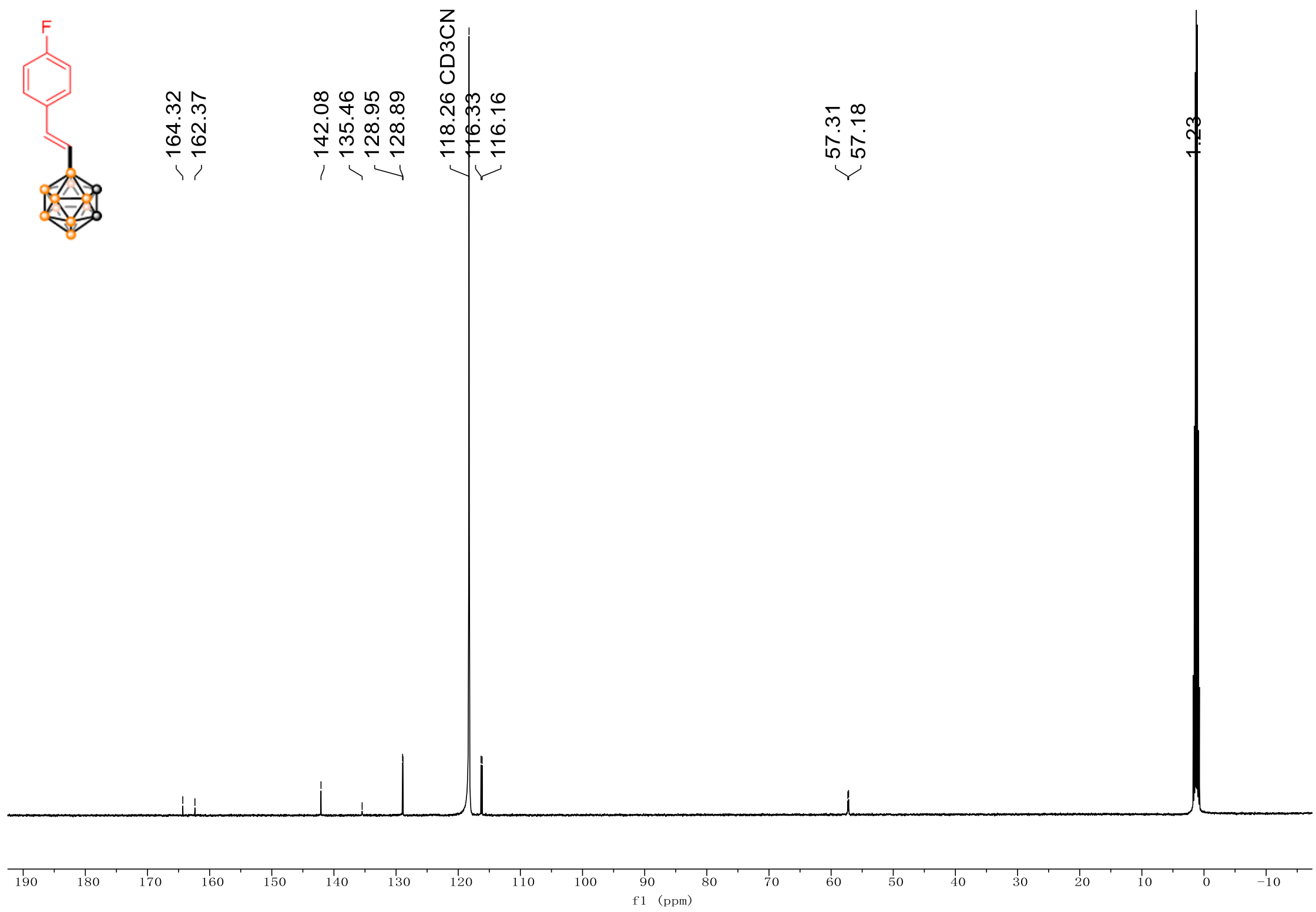

$^{13}C\{^{1}H\}$ NMR (125 MHz, CD_3CN) of **3bi**

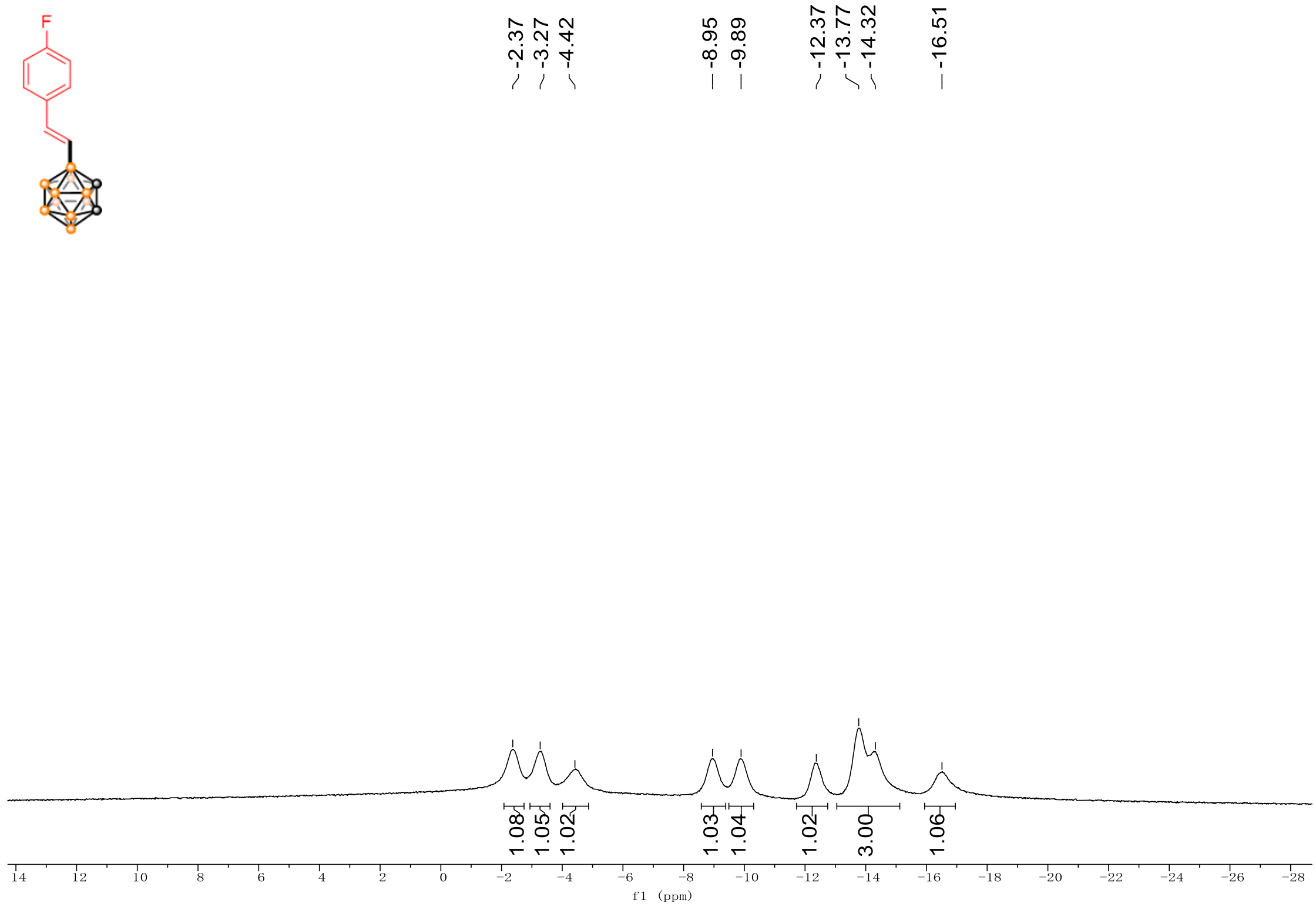

$^{11}B\{^{1}H\}$ NMR (160 MHz, CD_3CN) of **3bi**

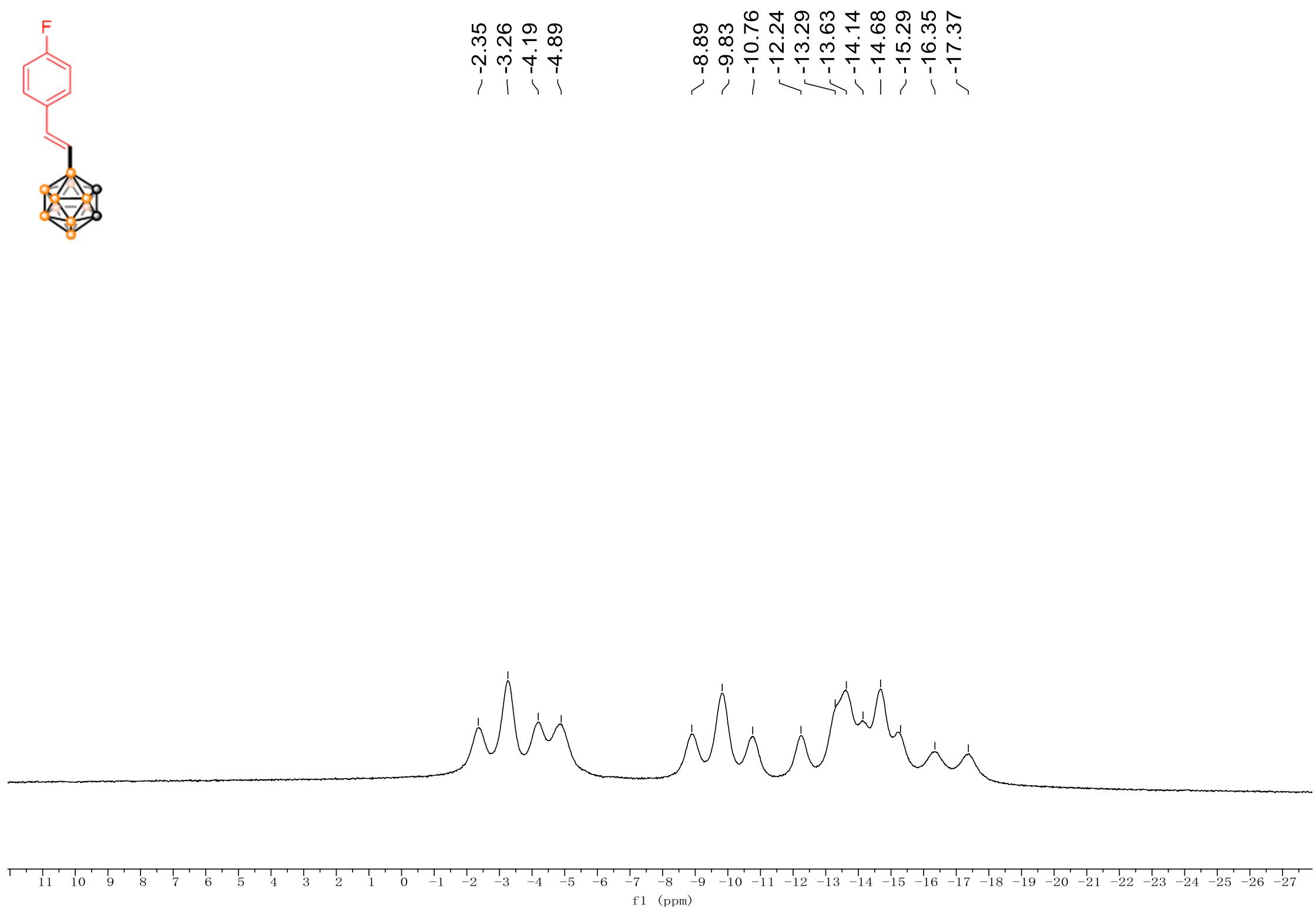

^{11}B NMR (160 MHz, CD_3CN) of **3bi**

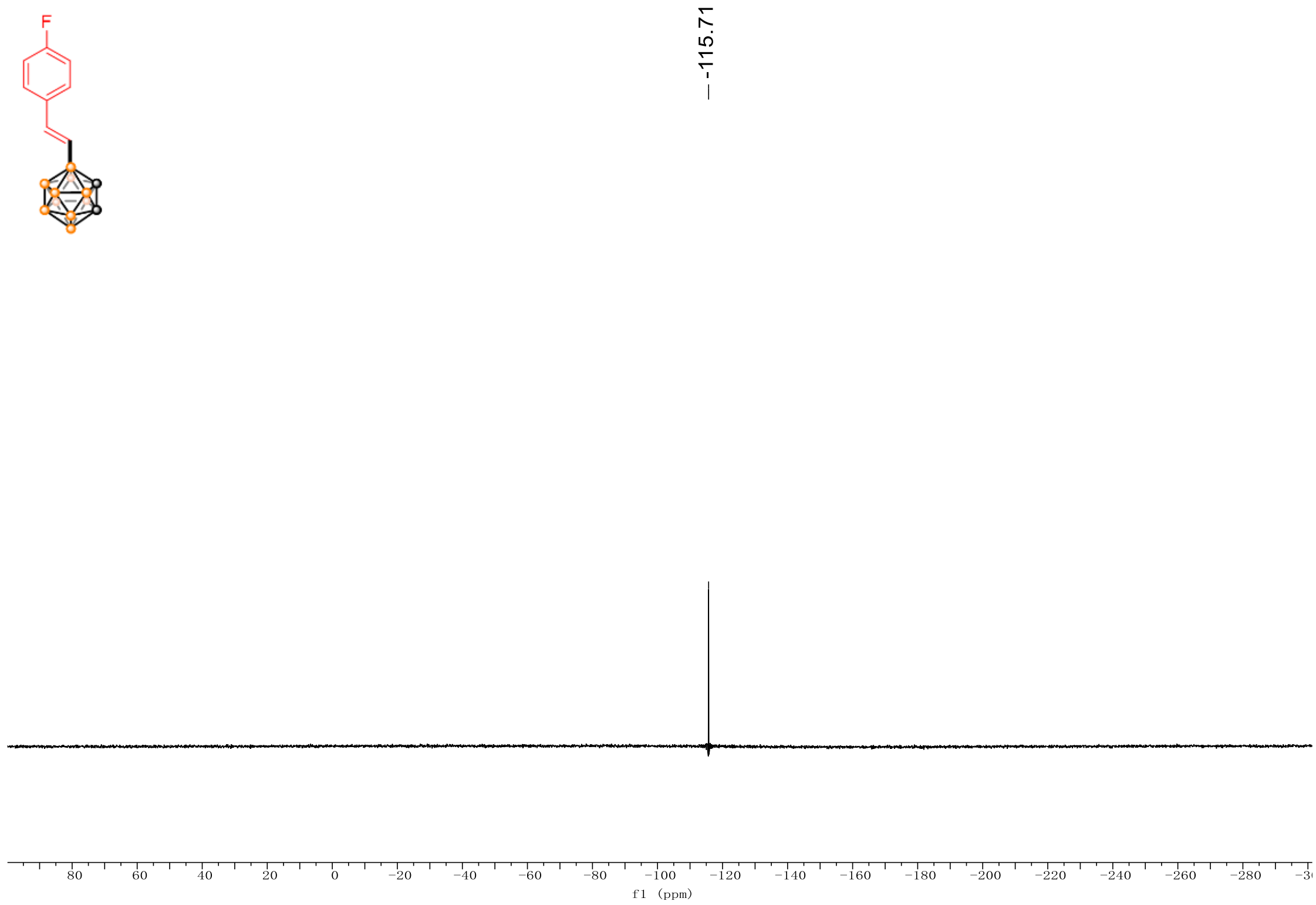

^{19}F NMR (376 MHz, CD_3CN) of **3bi**

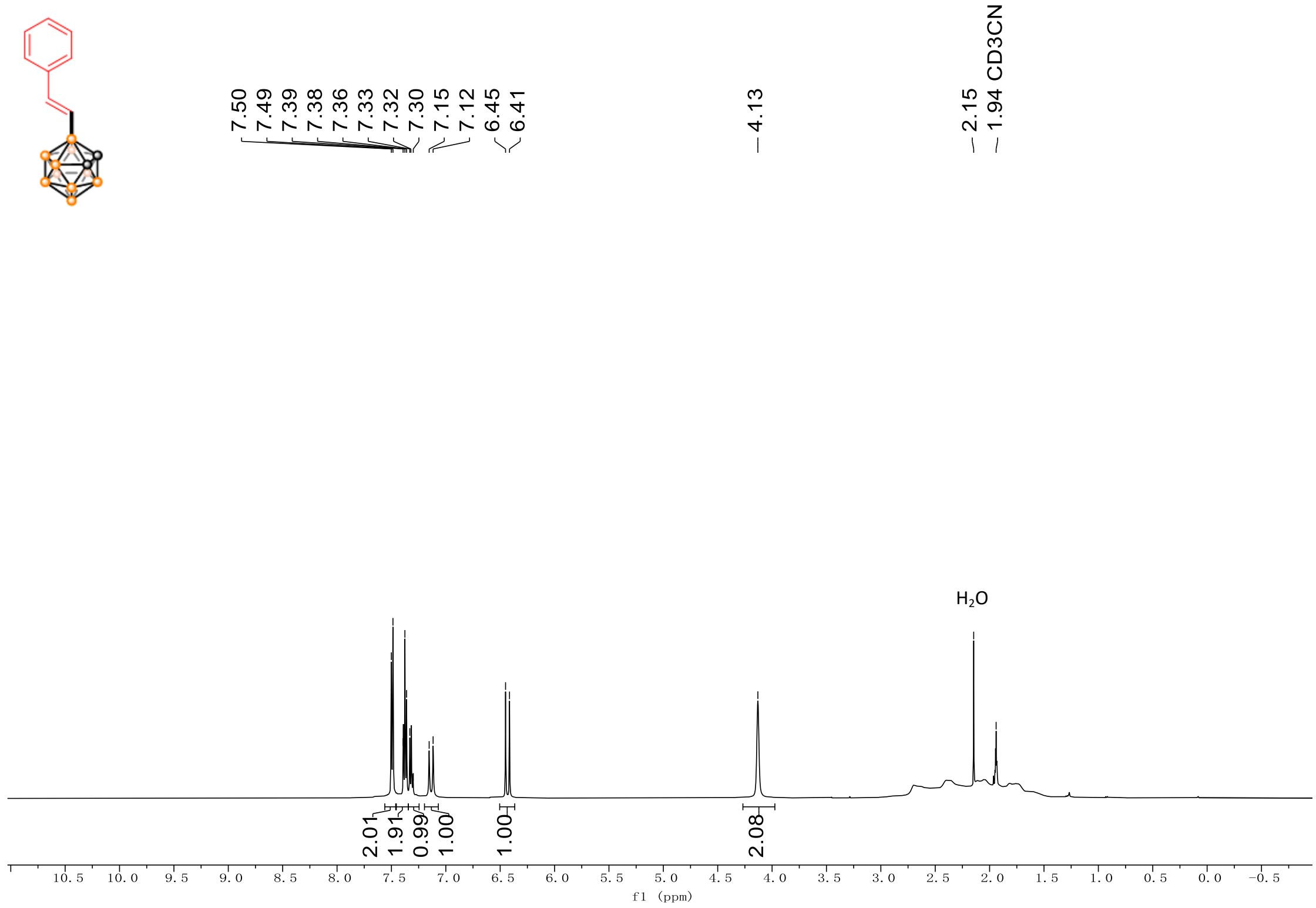

^{1}H NMR (500 MHz, CD_3CN) of **3ca**

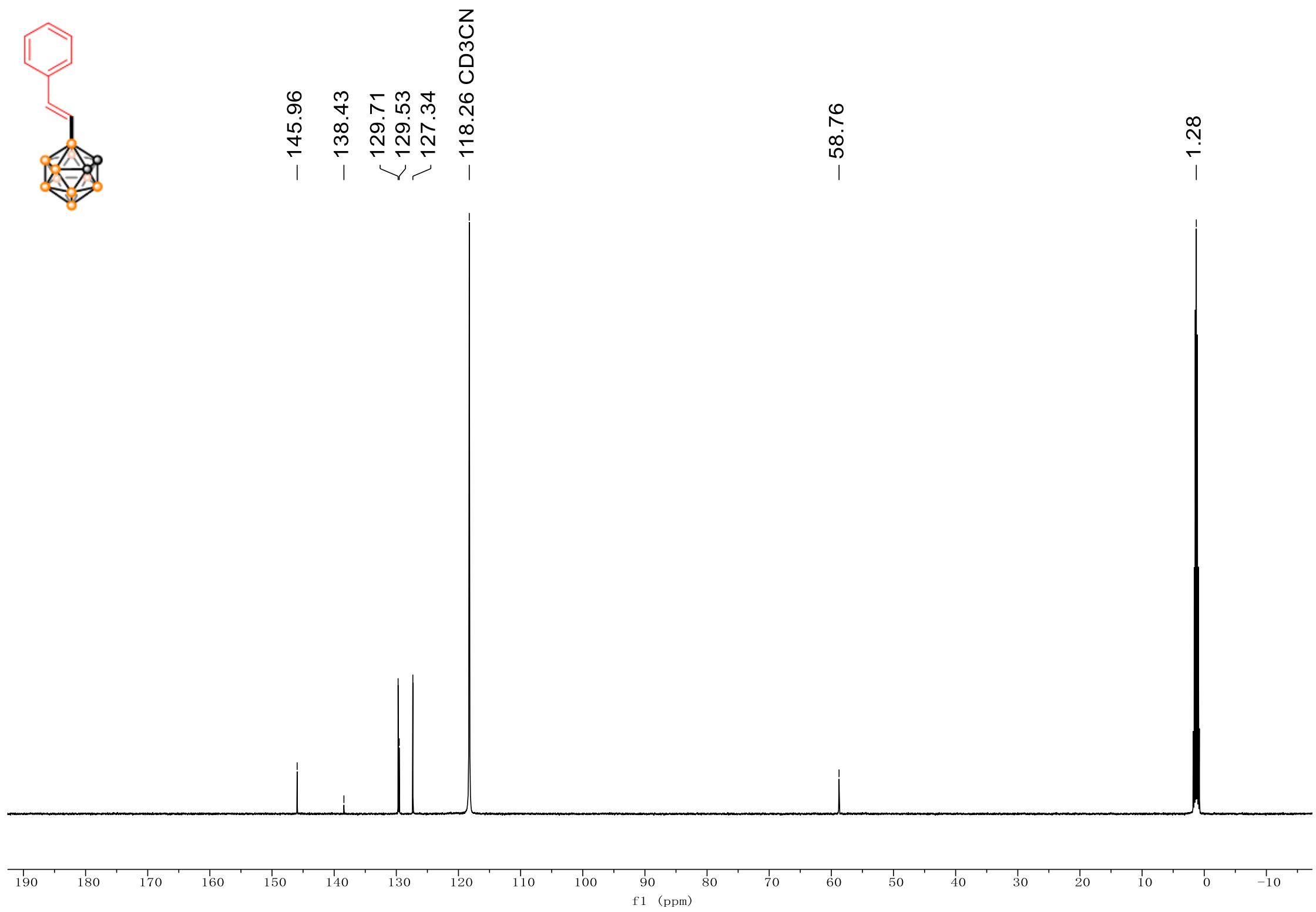

$^{13}C\{^1H\}$ NMR (125 MHz, CD_3CN) of **3ca**

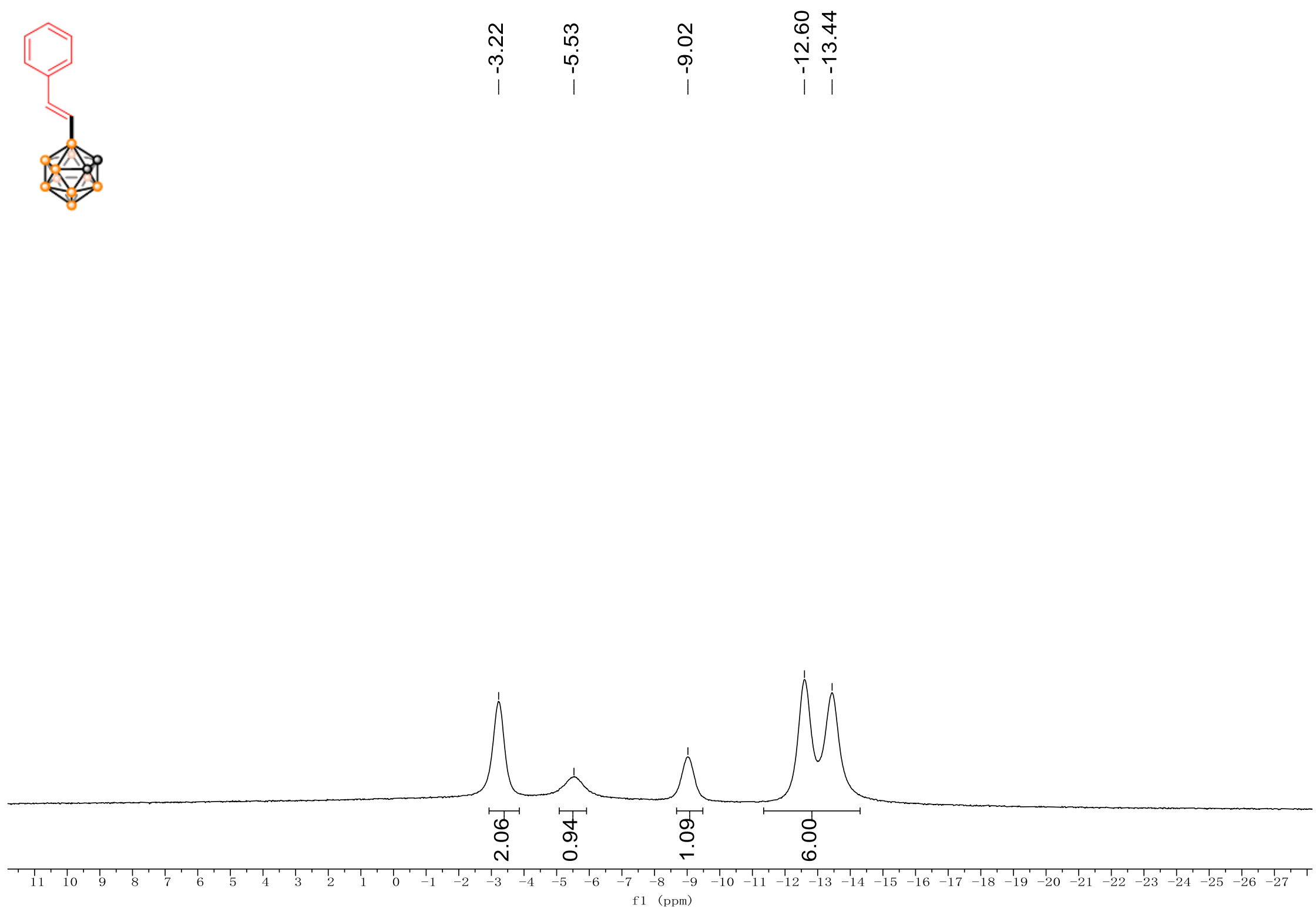

$^{11}B\{^{1}H\}$ NMR (160 MHz, CD_3CN) of **3ca**

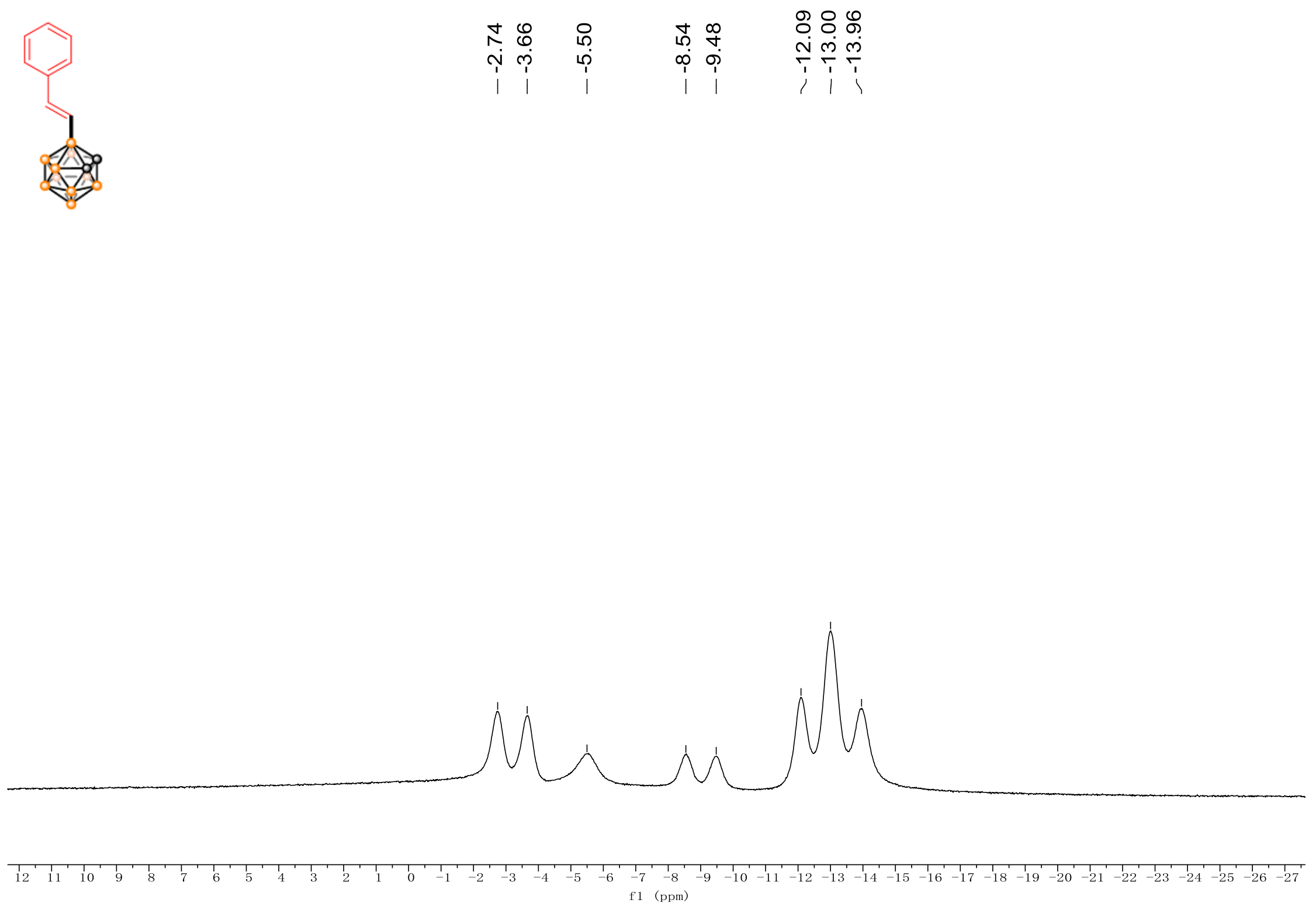

^{11}B NMR (160 MHz, CD_3CN) of **3ca**

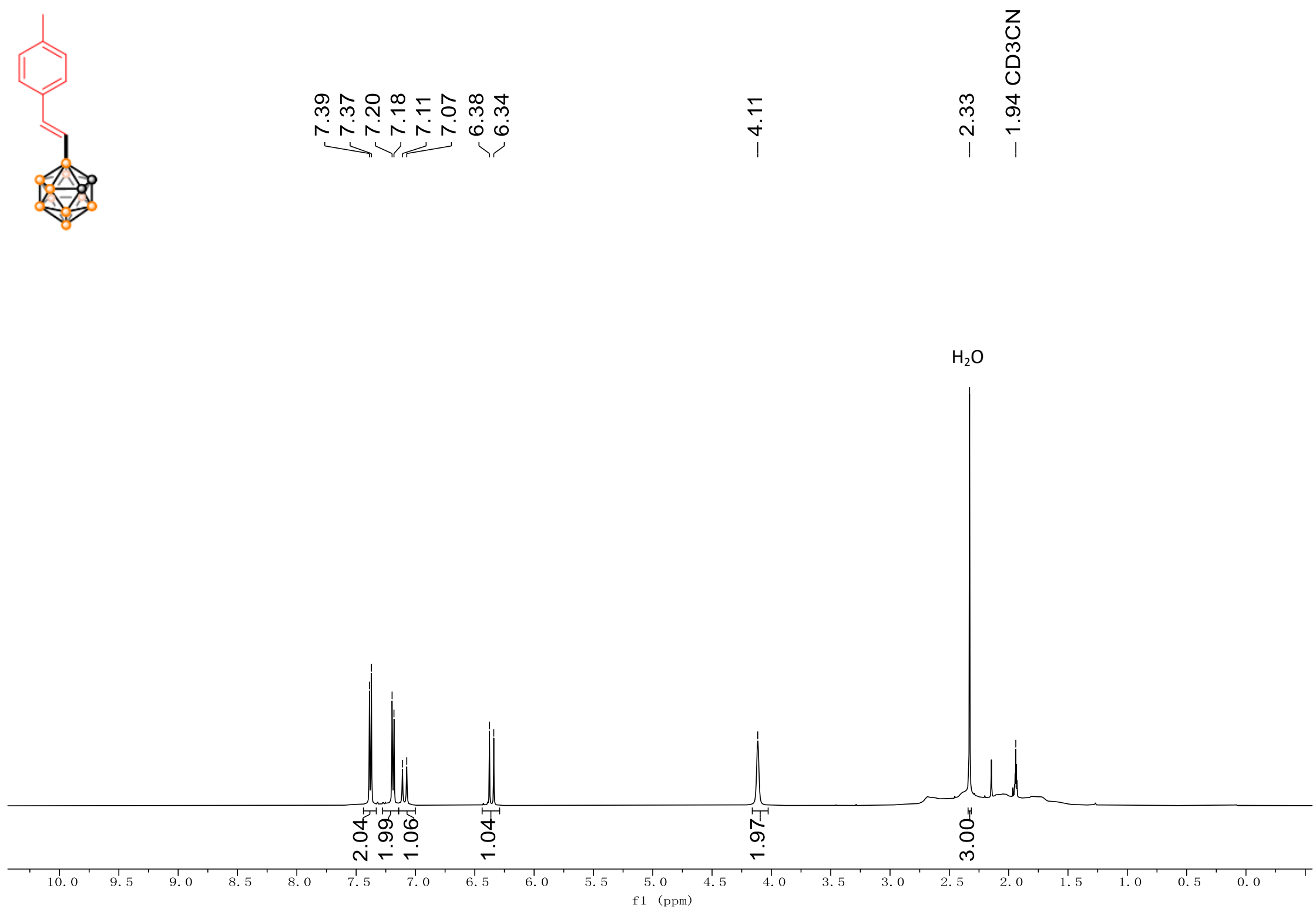

^1H NMR (500 MHz, CD_3CN) of **3cb**

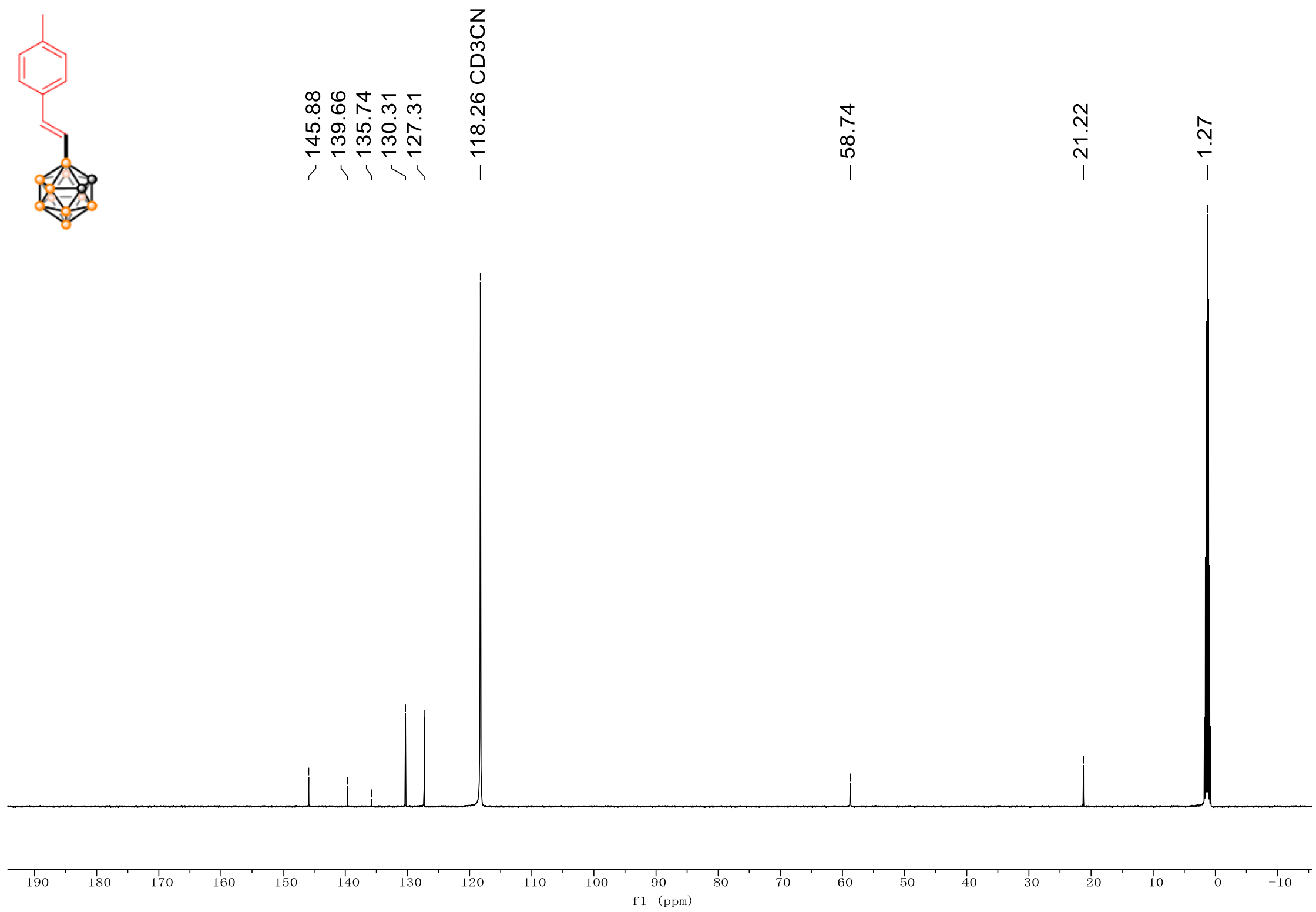

$^{13}C\{^1H\}$ NMR (125 MHz, CD_3CN) of **3cb**

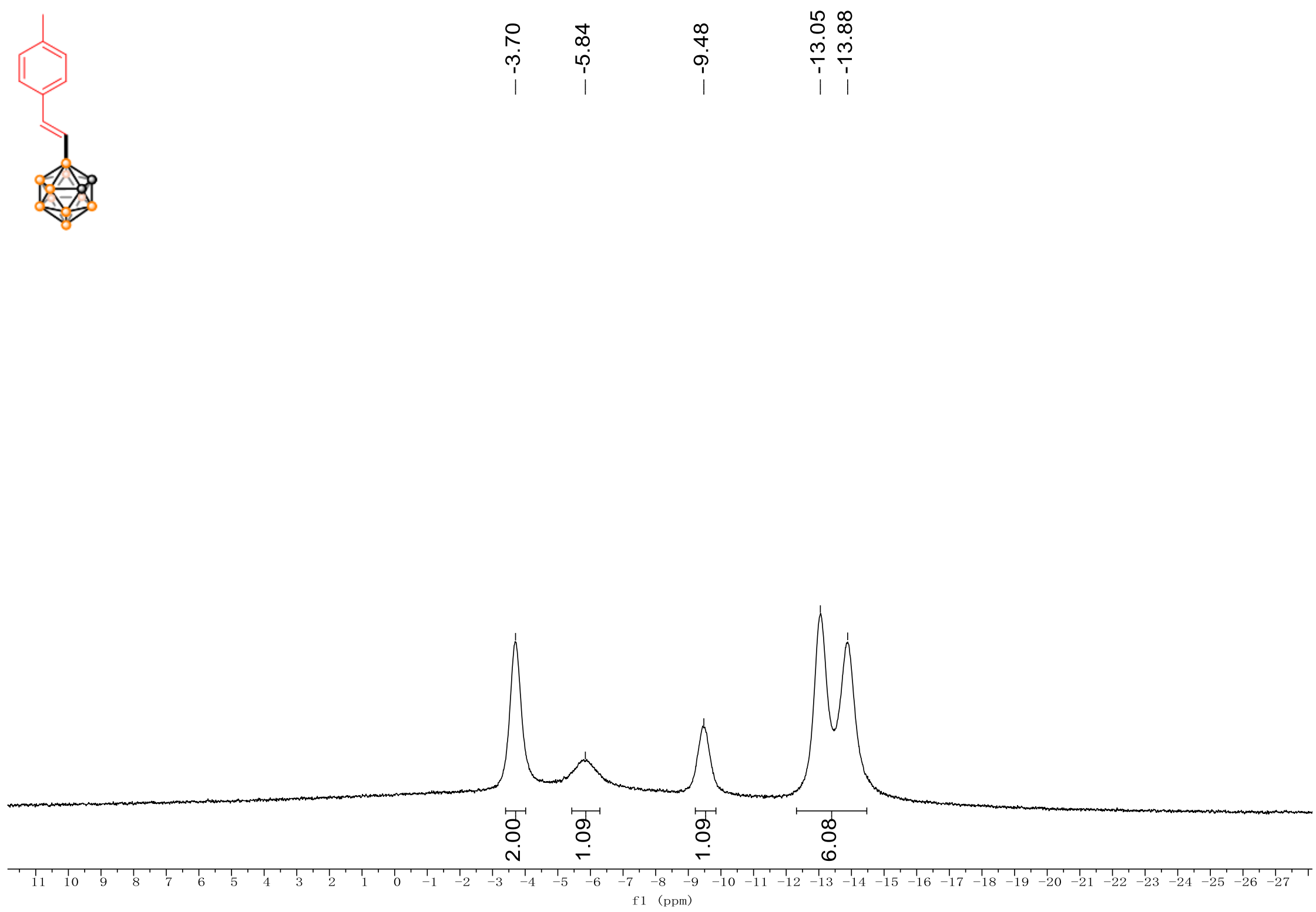

$^{11}B\{^1H\}$ NMR (160 MHz, CD_3CN) of **3cb**

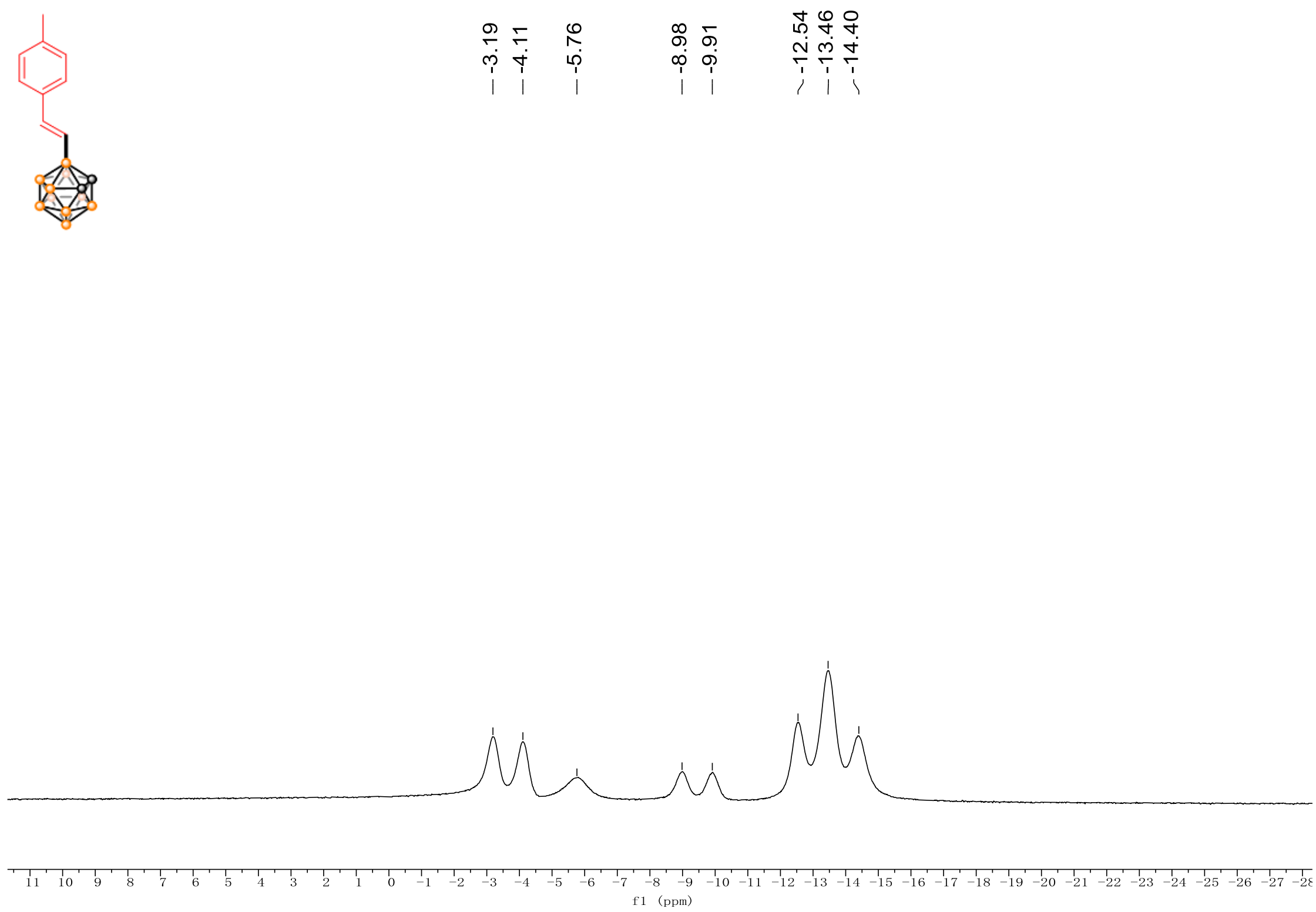

^{11}B NMR (160 MHz, CD_3CN) of **3cb**

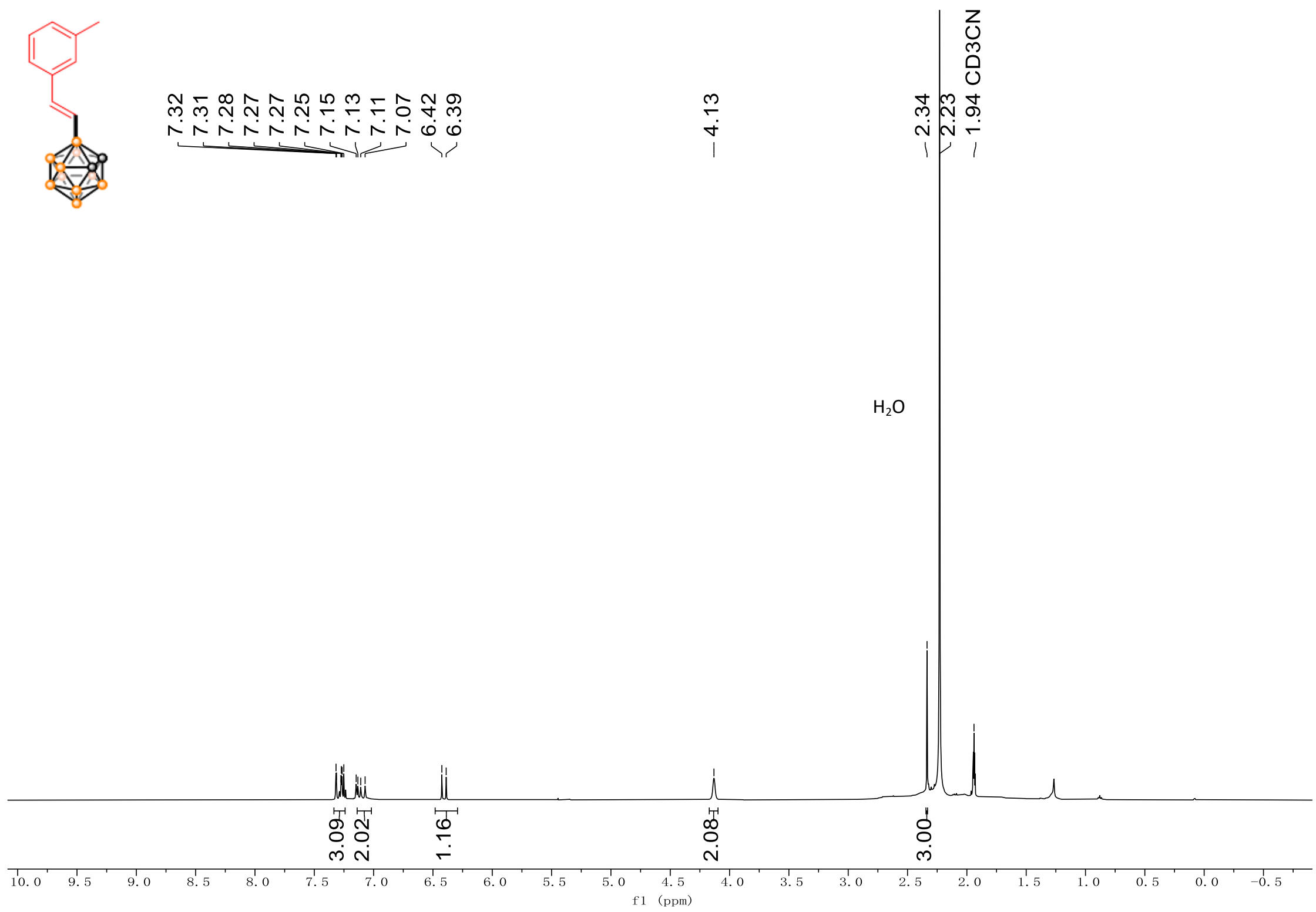

^1H NMR (500 MHz, CD_3CN) of **3cc**

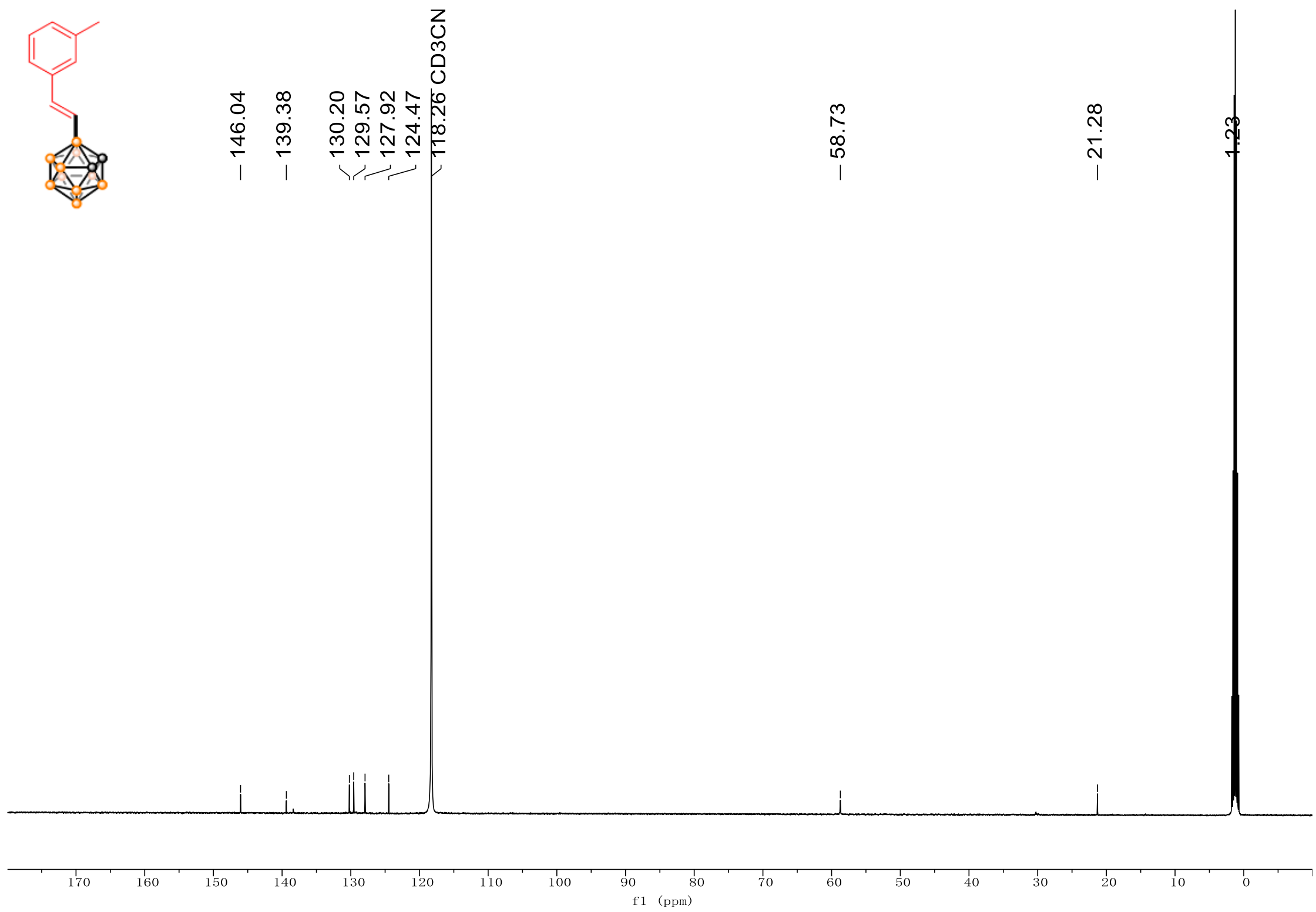

$^{13}C\{^1H\}$ NMR (125 MHz, CD_3CN) of **3cc**

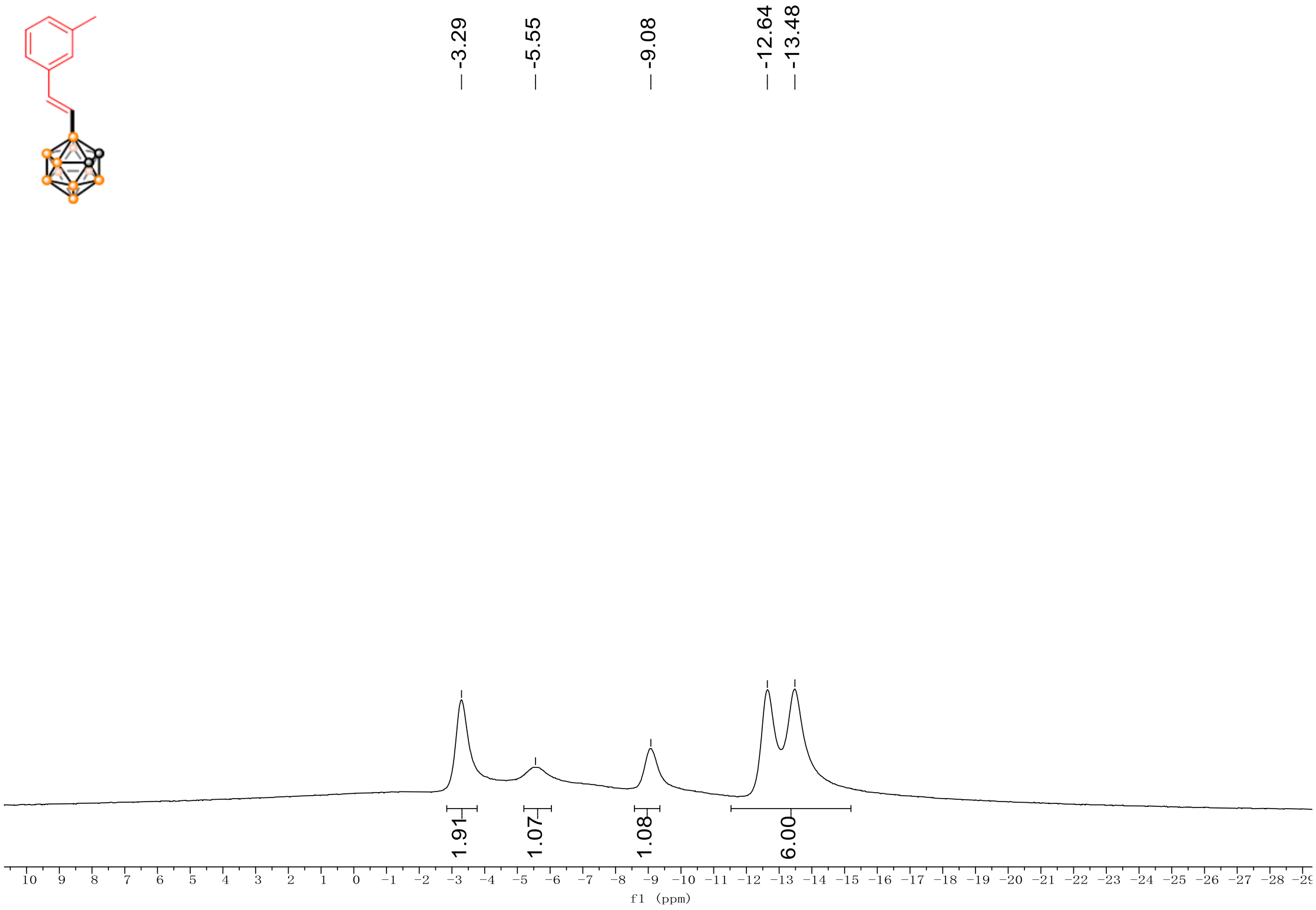

$^{11}B\{^{1}H\}$ NMR (160 MHz, CD_3CN) of **3cc**

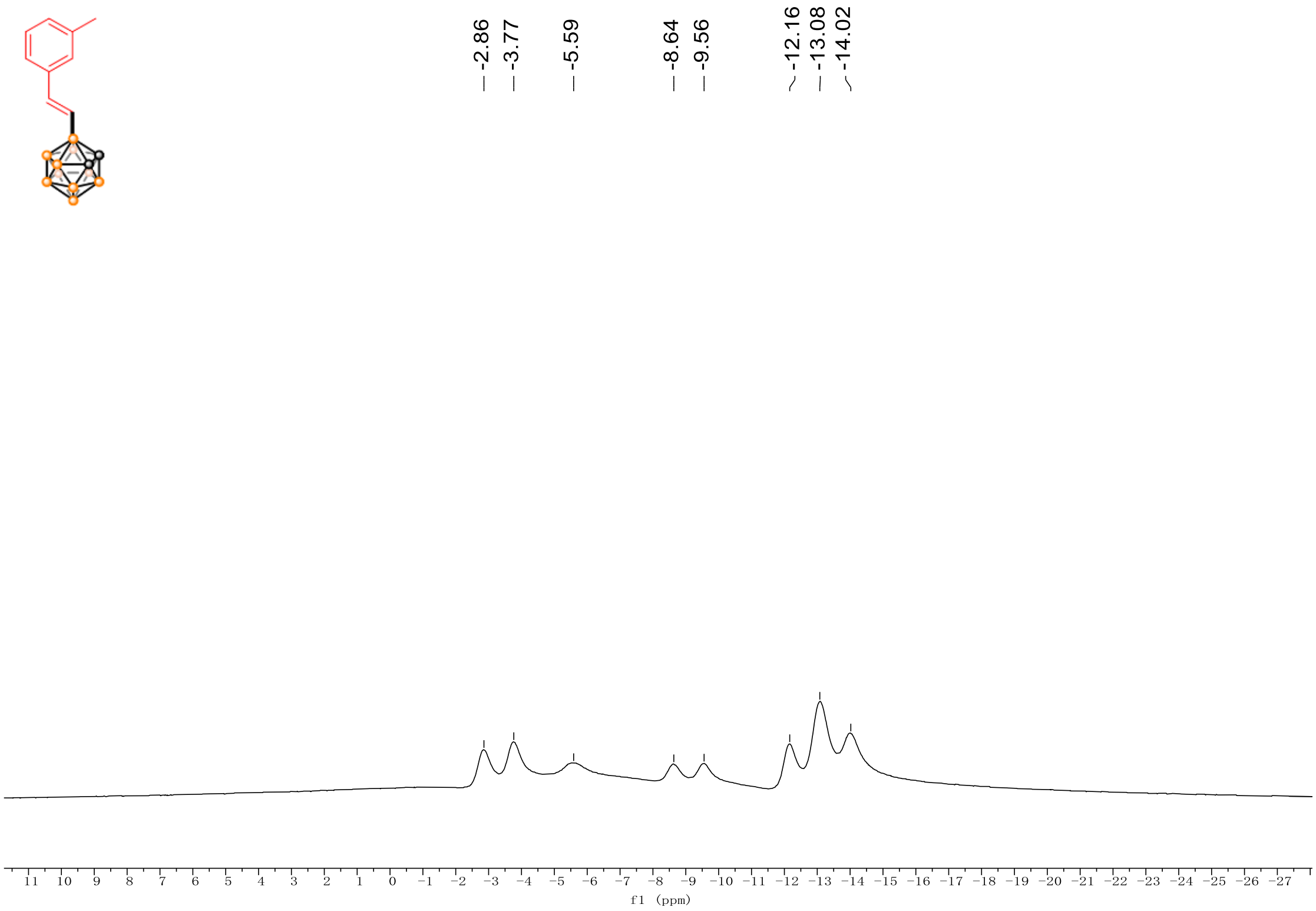

^{11}B NMR (160 MHz, CD_3CN) of **3cc**

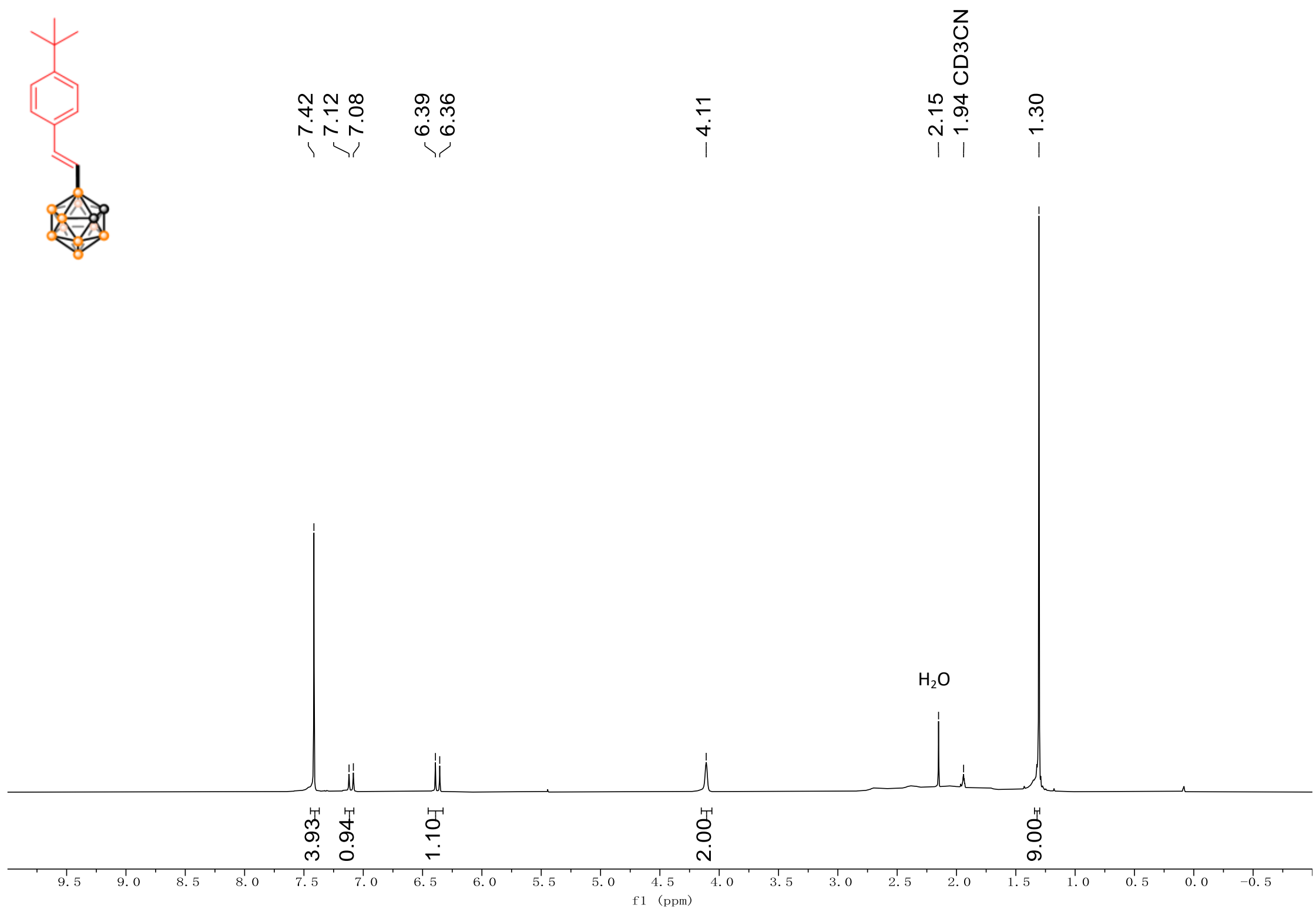

^{1}H NMR (500 MHz, CD_3CN) of **3ce**

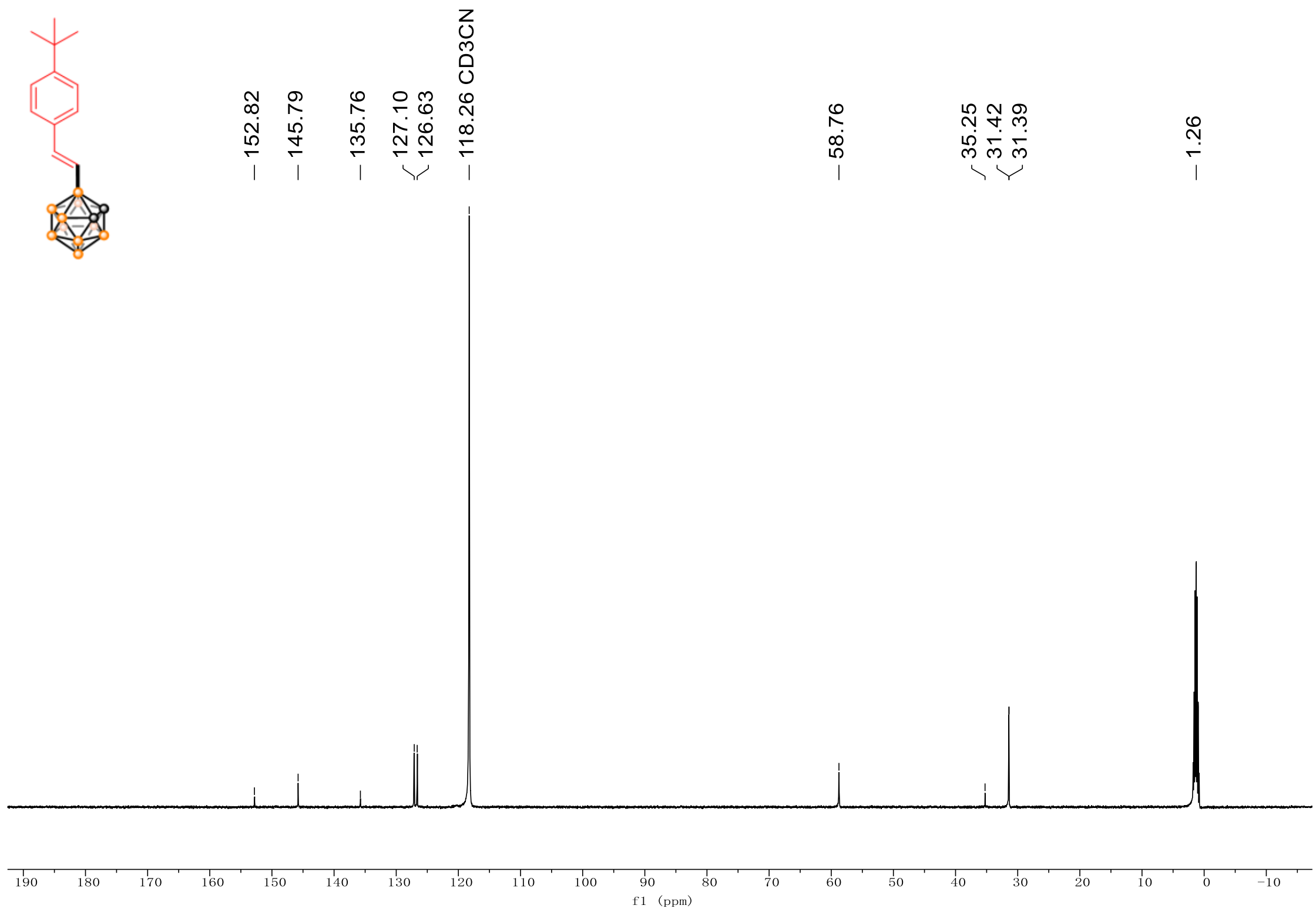

$^{13}C\{^{1}H\}$ NMR (125 MHz, CD_3CN) of **3ce**

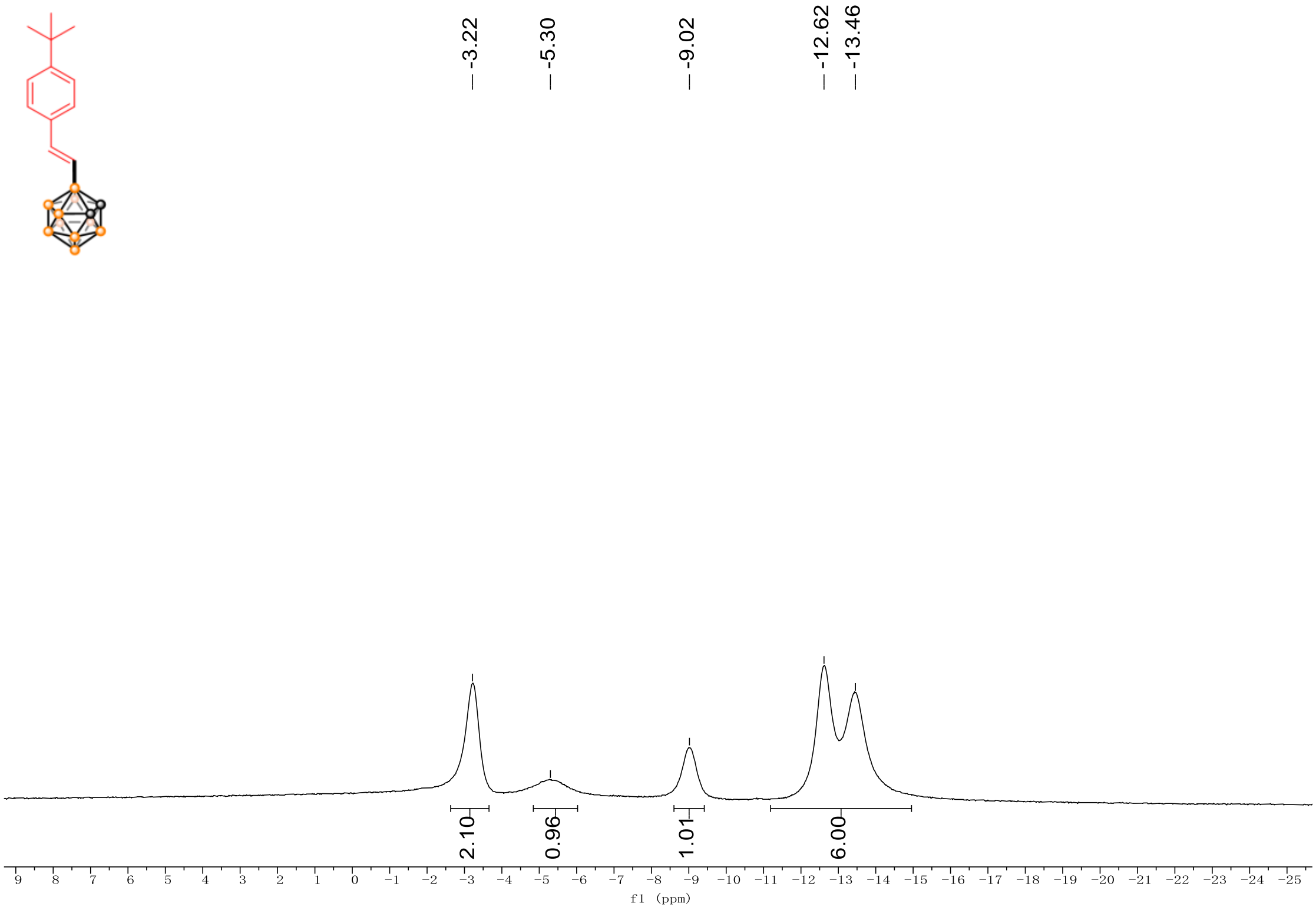

$^{11}B\{^{1}H\}$ NMR (160 MHz, CD_3CN) of **3ce**

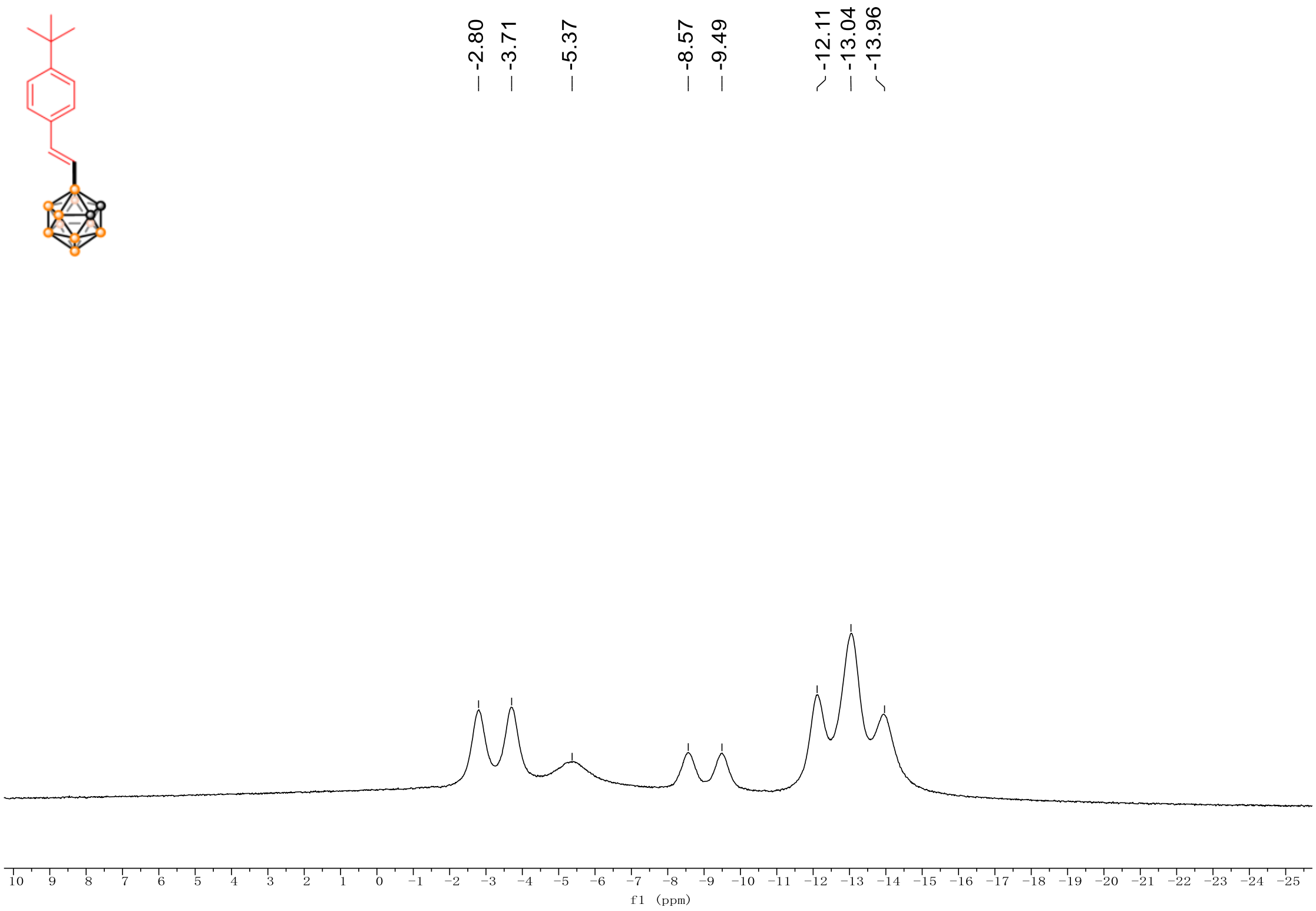

^{11}B NMR (160 MHz, CD_3CN) of **3ce**

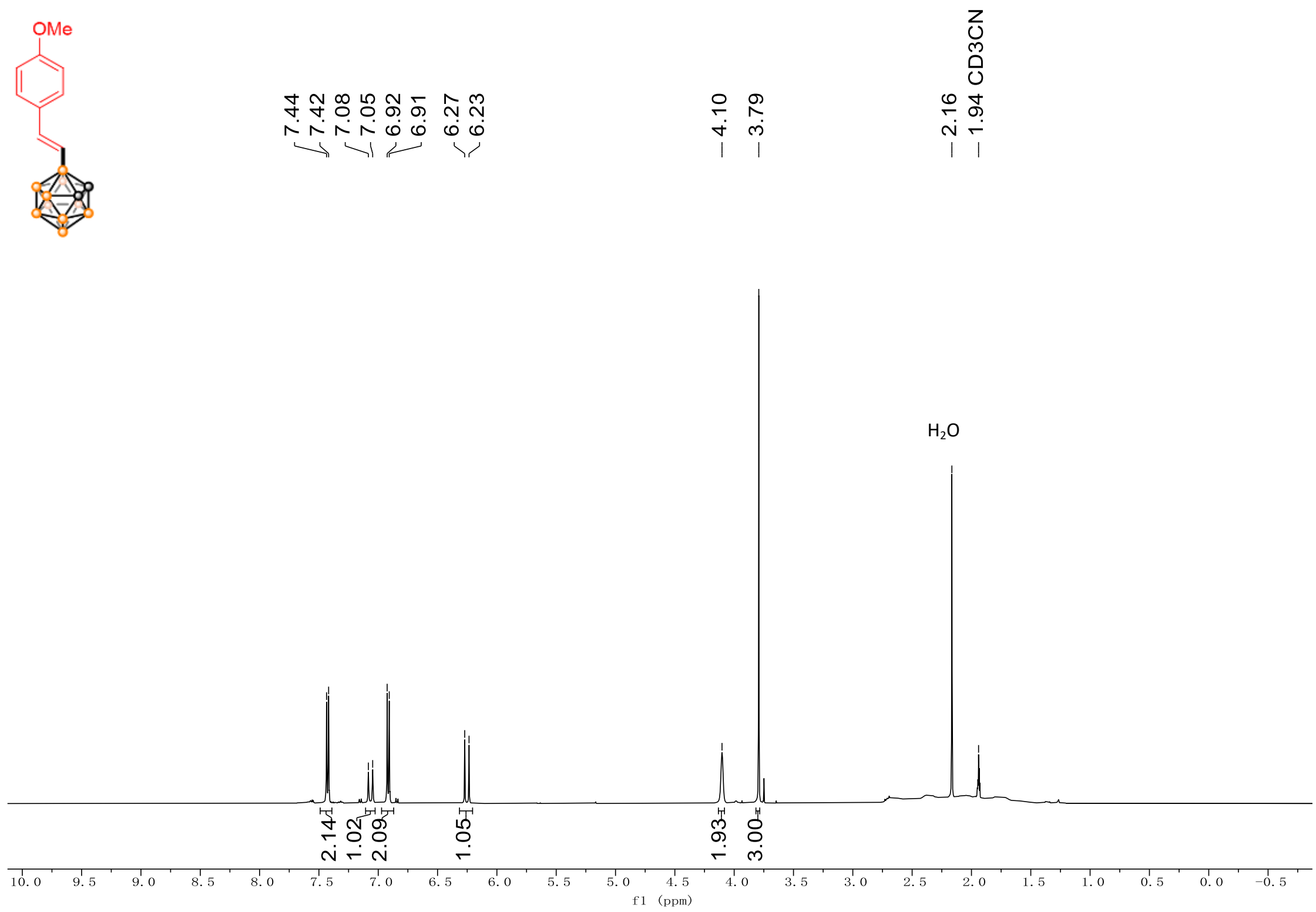

^{1}H NMR (500 MHz, CD_3CN) of **3cf**

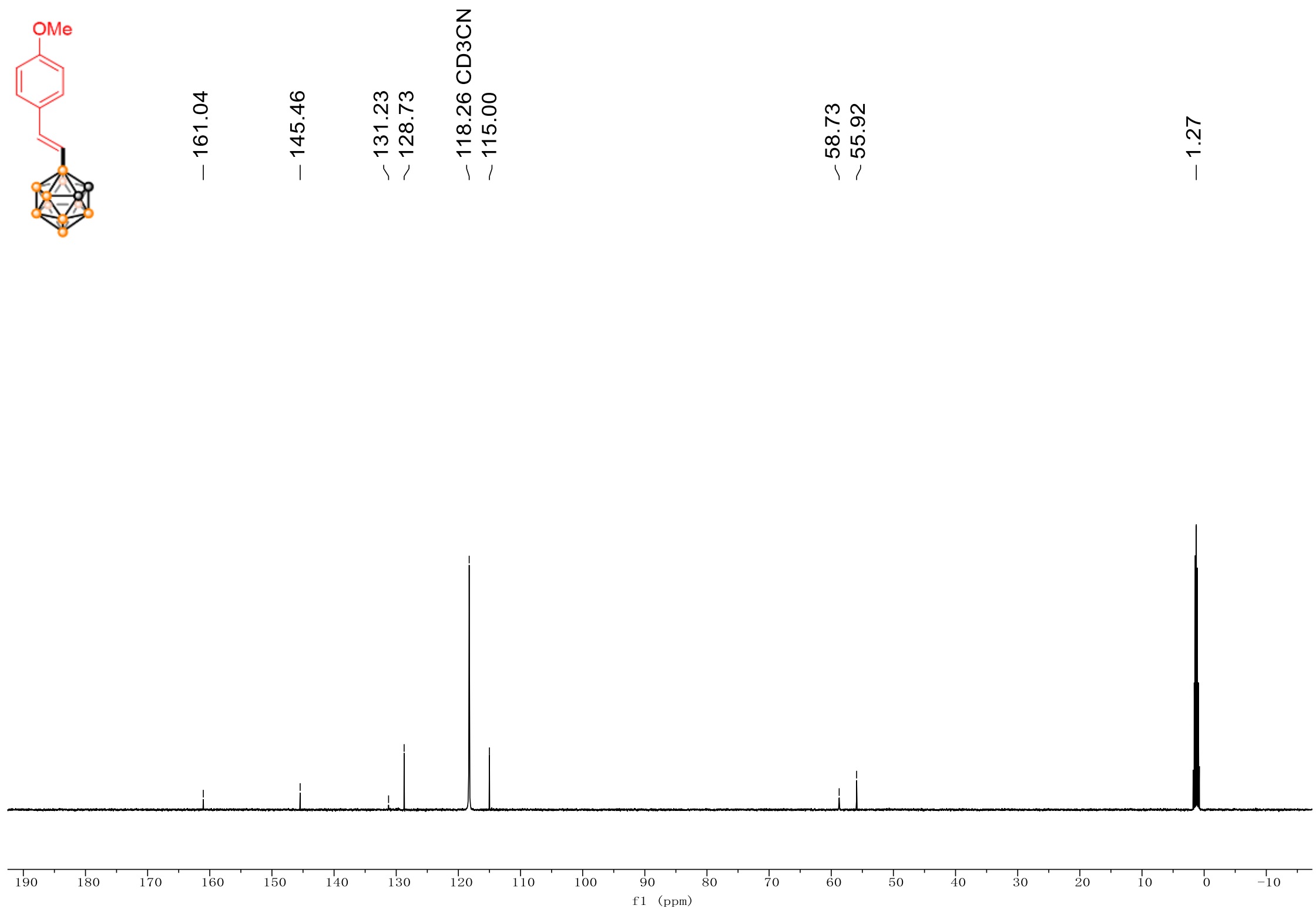

$^{13}C\{^{1}H\}$ NMR (125 MHz, CD_3CN) of **3cf**

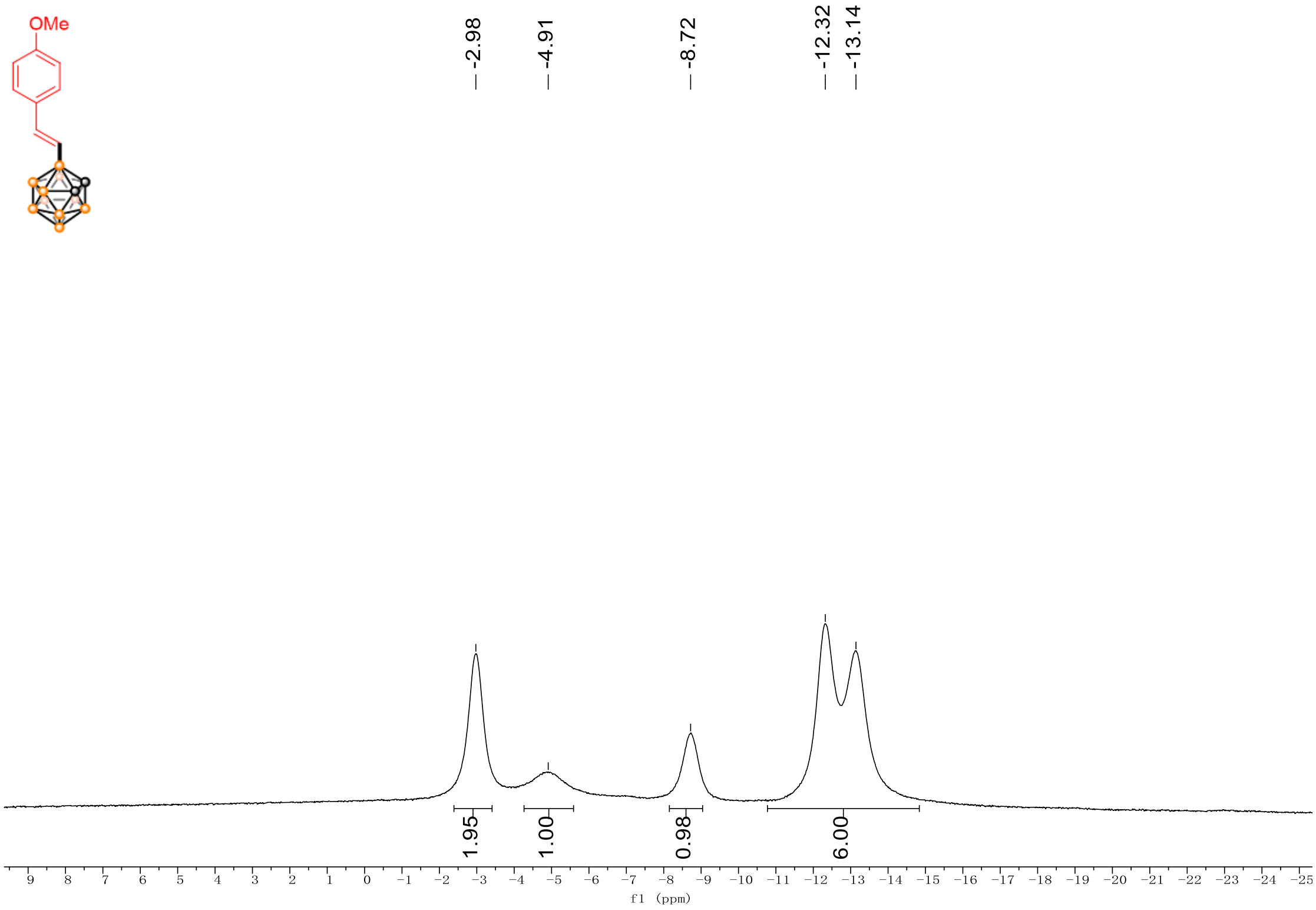

$^{11}B\{^{1}H\}$ NMR (160 MHz, CD_3CN) of **3cf**

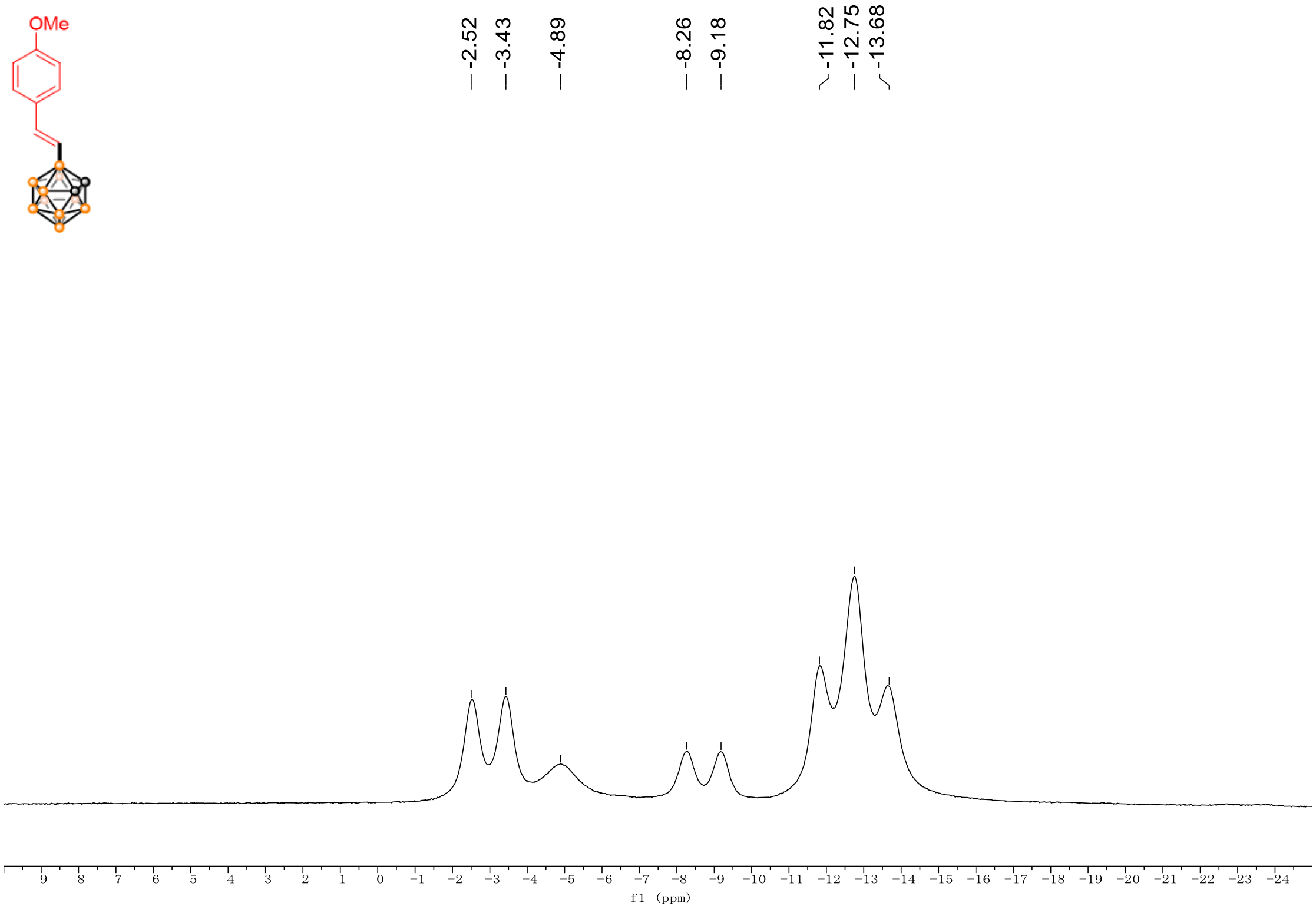

^{11}B NMR (160 MHz, CD_3CN) of **3cf**

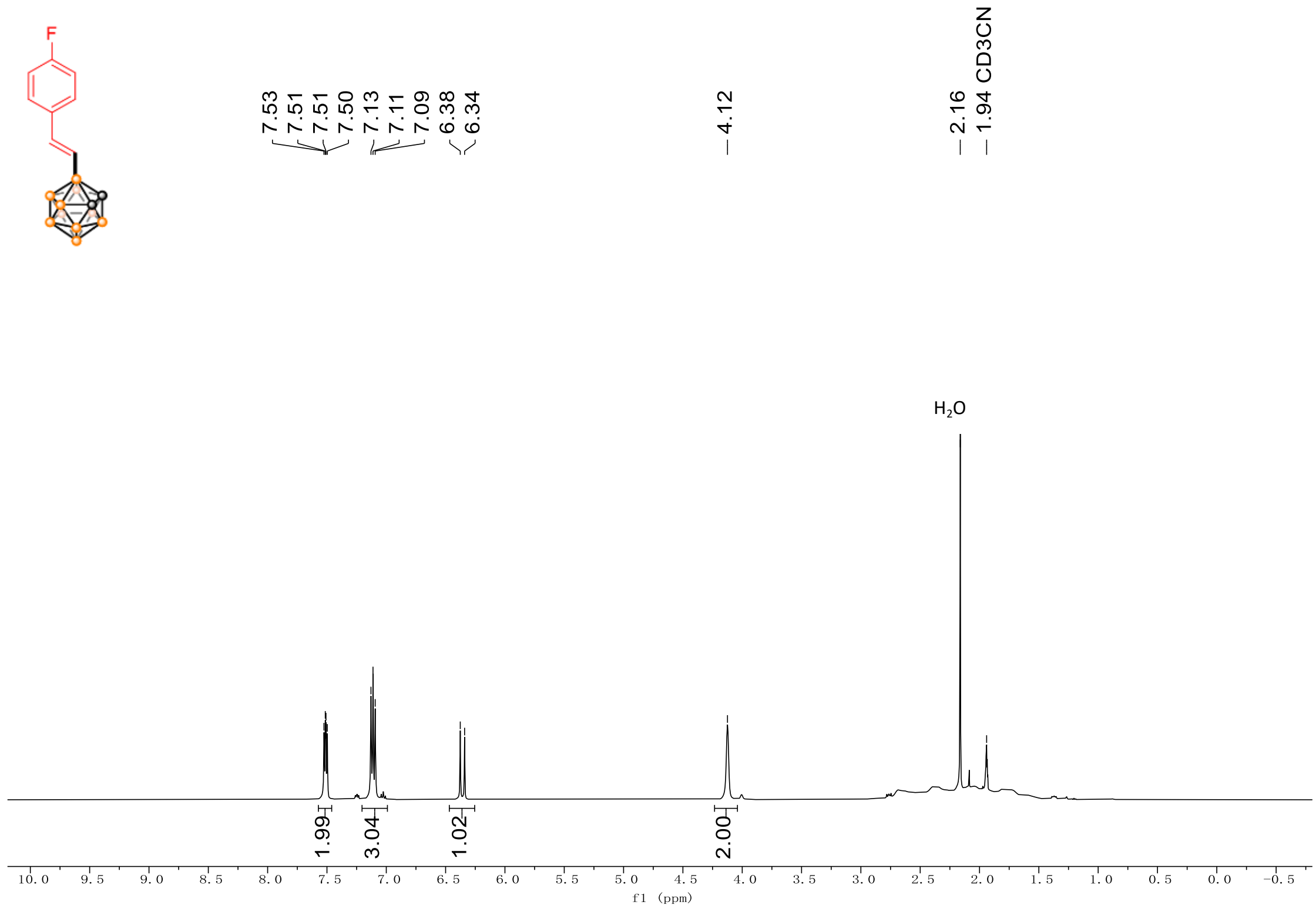

^{1}H NMR (500 MHz, CD_3CN) of **3ci**

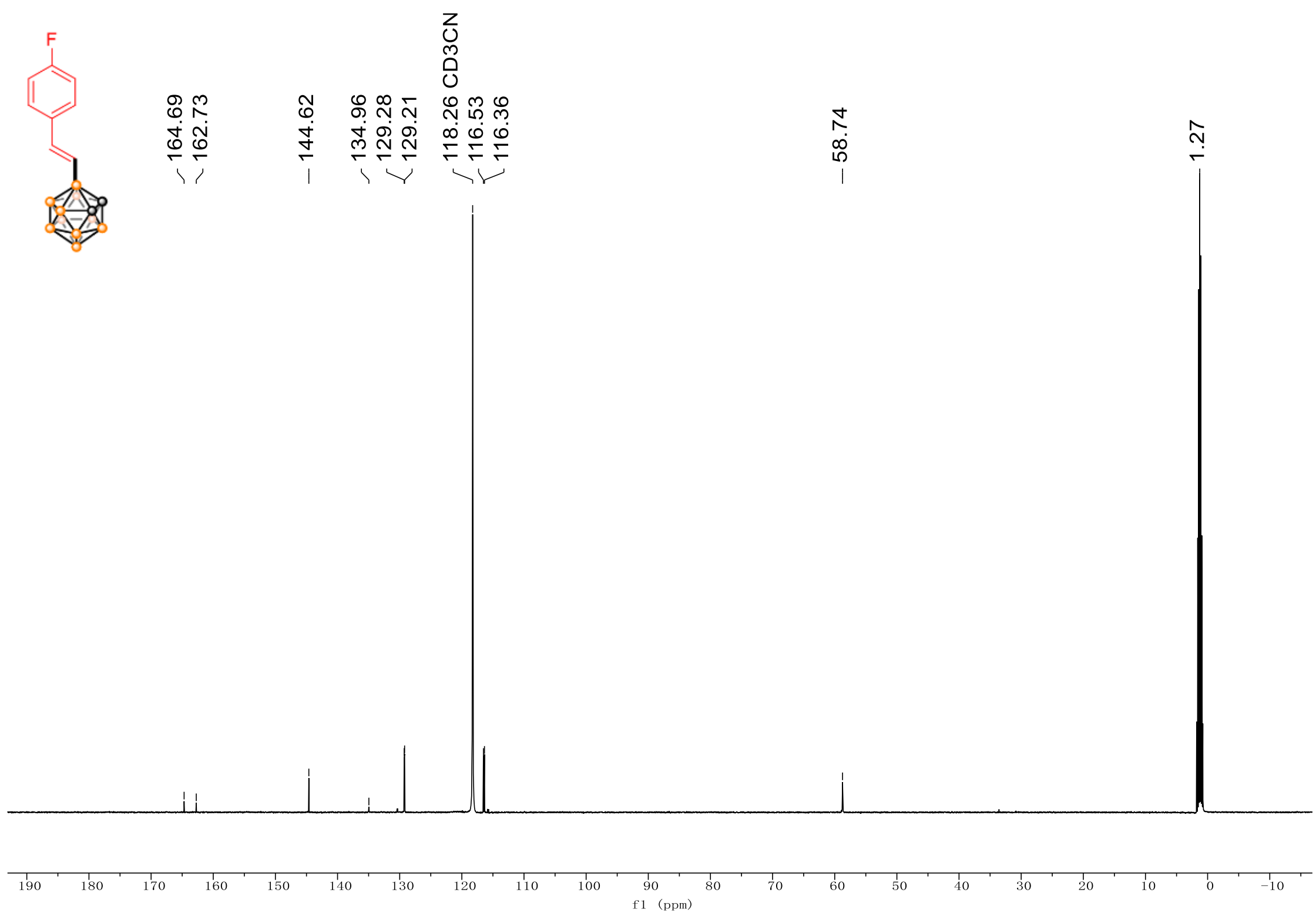

$^{13}C\{^{1}H\}$ NMR (125 MHz, CD_3CN) of **3ci**

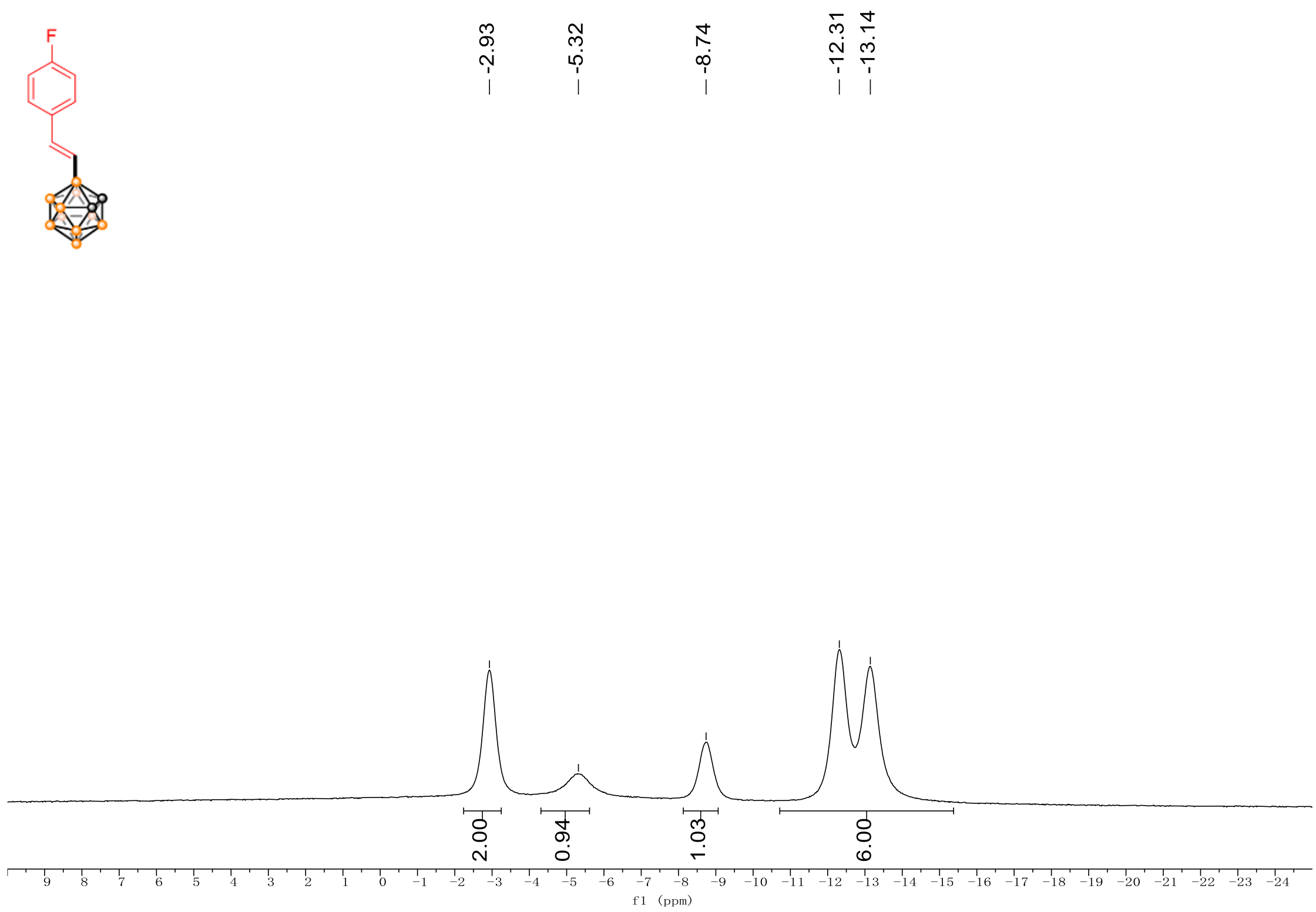

$^{11}B\{^{1}H\}$ NMR (160 MHz, CD_3CN) of **3ci**

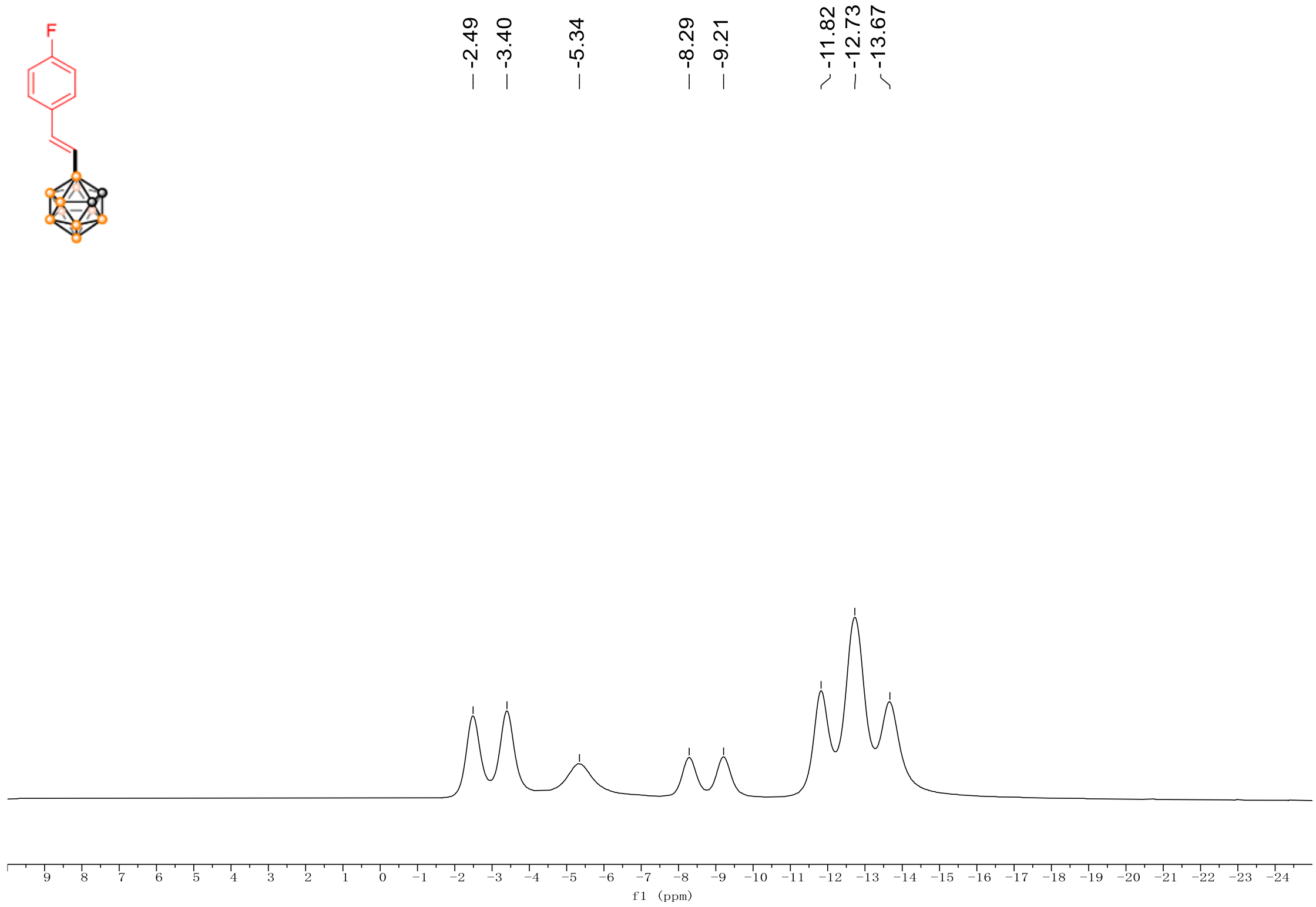

^{11}B NMR (160 MHz, CD_3CN) of **3ci**

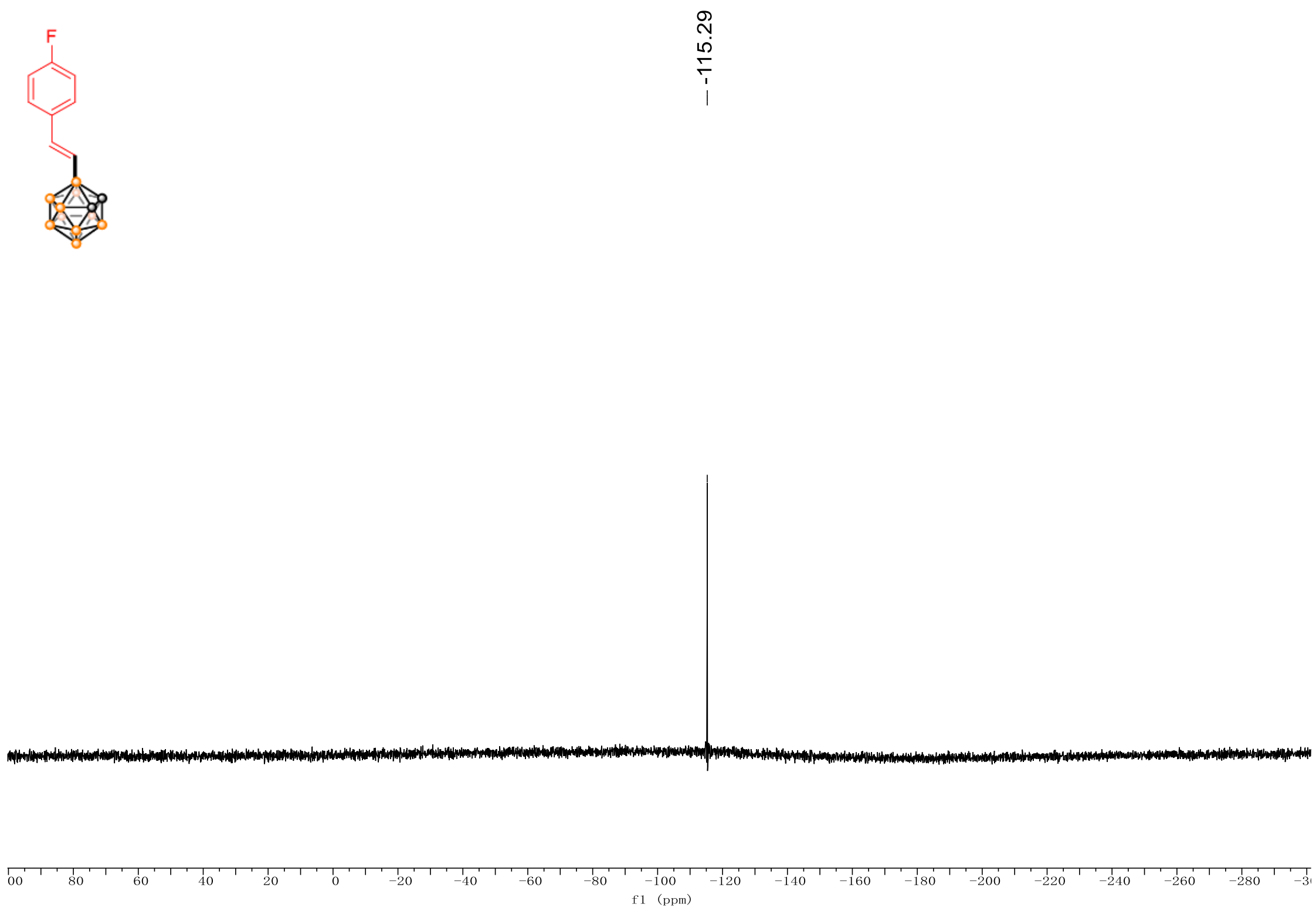

^{19}F NMR (376 MHz, CD_3CN) of **3ci**

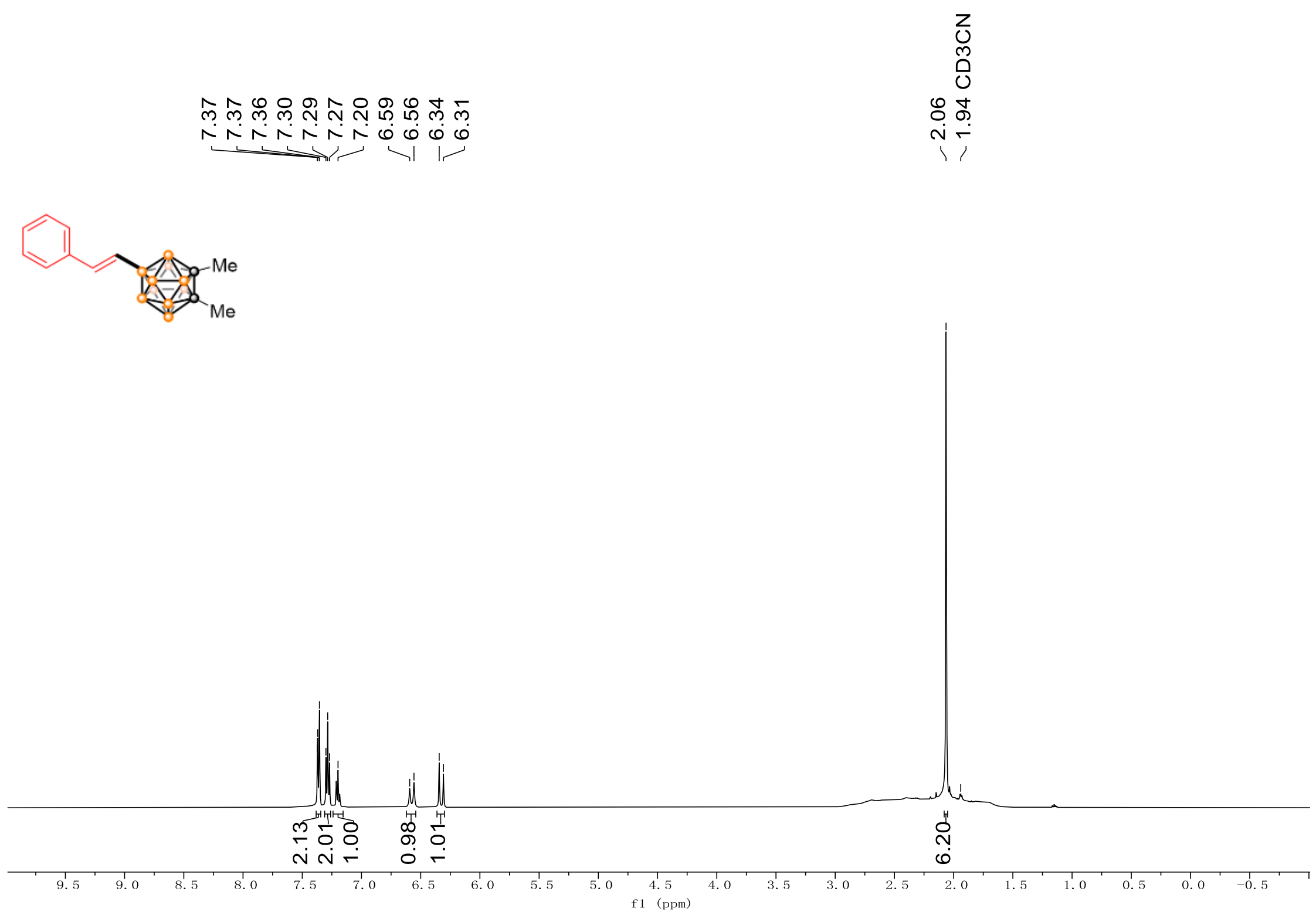

^{1}H NMR (500 MHz, CD_3CN) of **3da**

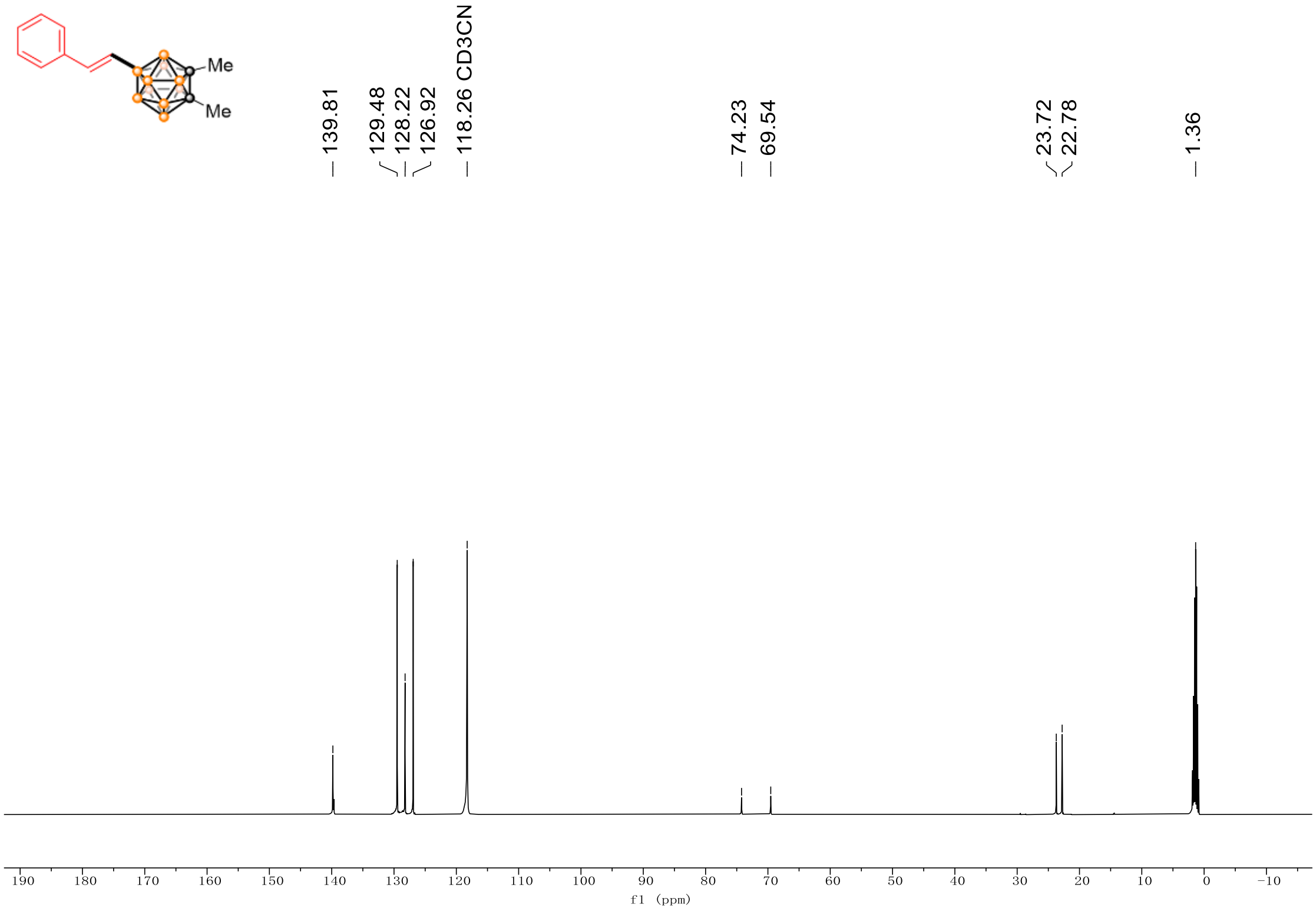

$^{13}C\{^{1}H\}$ NMR (125 MHz, CD_3CN) of **3da**

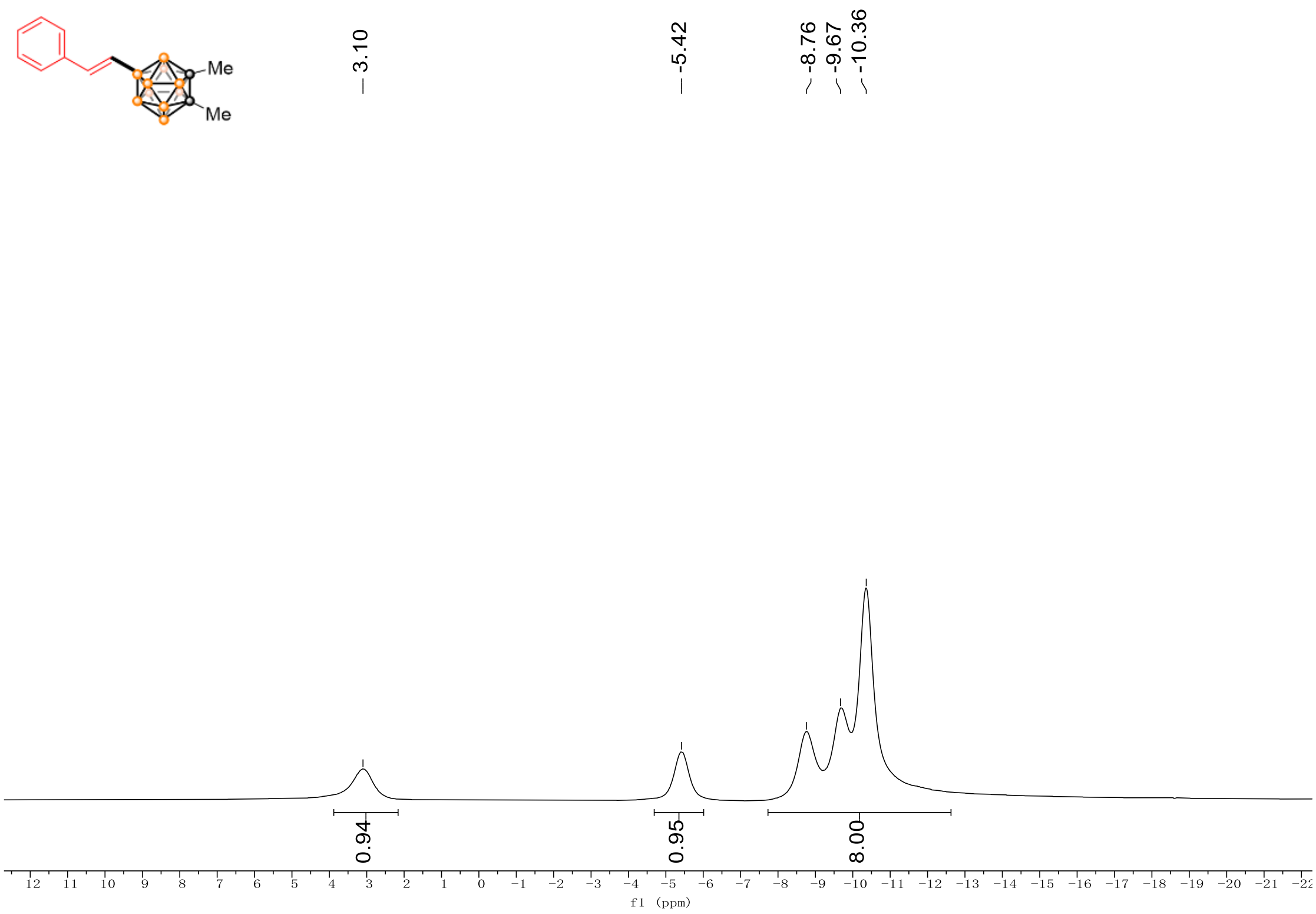

$^{11}B\{^{1}H\}$ NMR (160 MHz, CD_3CN) of **3da**

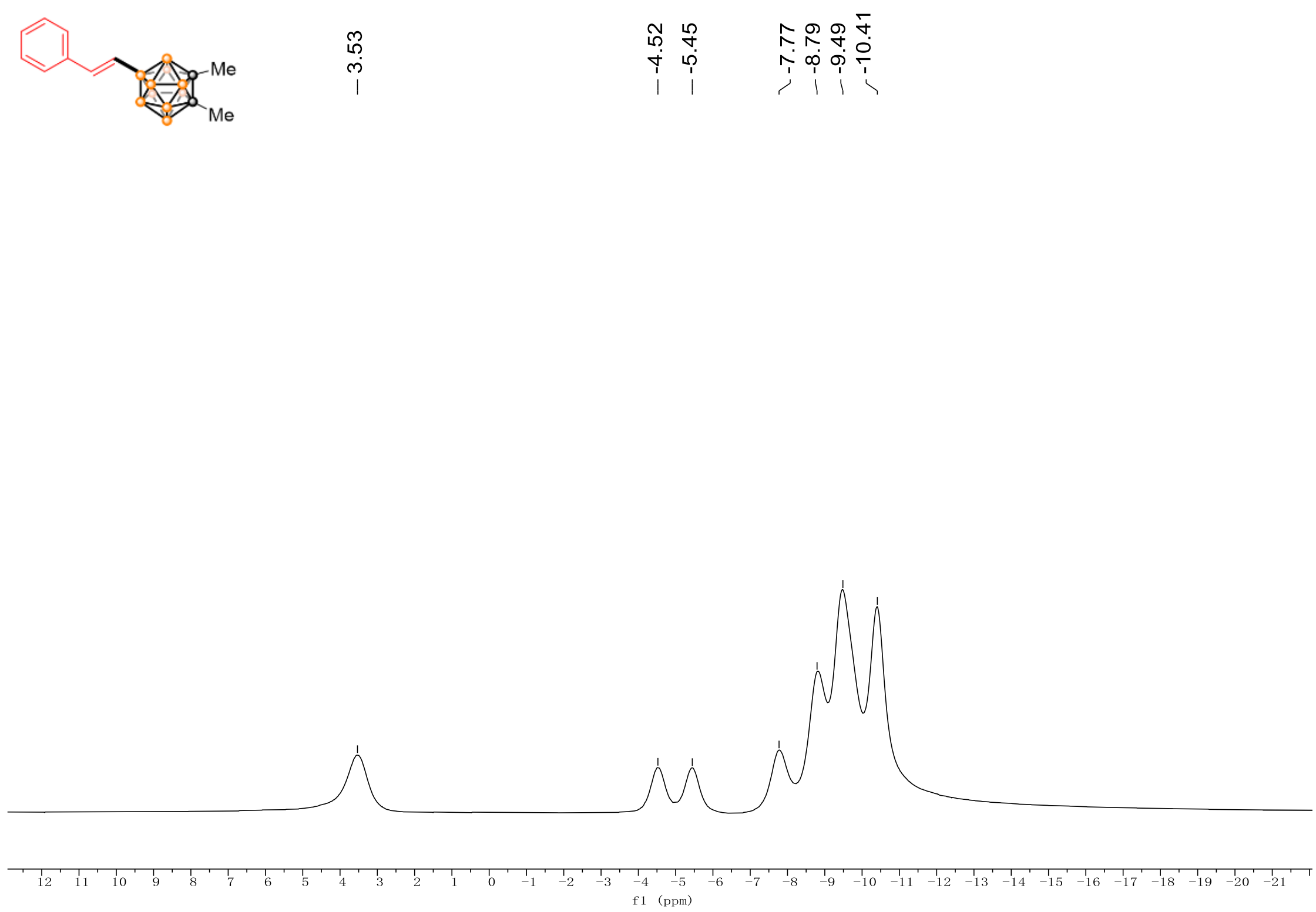

^{11}B NMR (160 MHz, CD_3CN) of **3da**

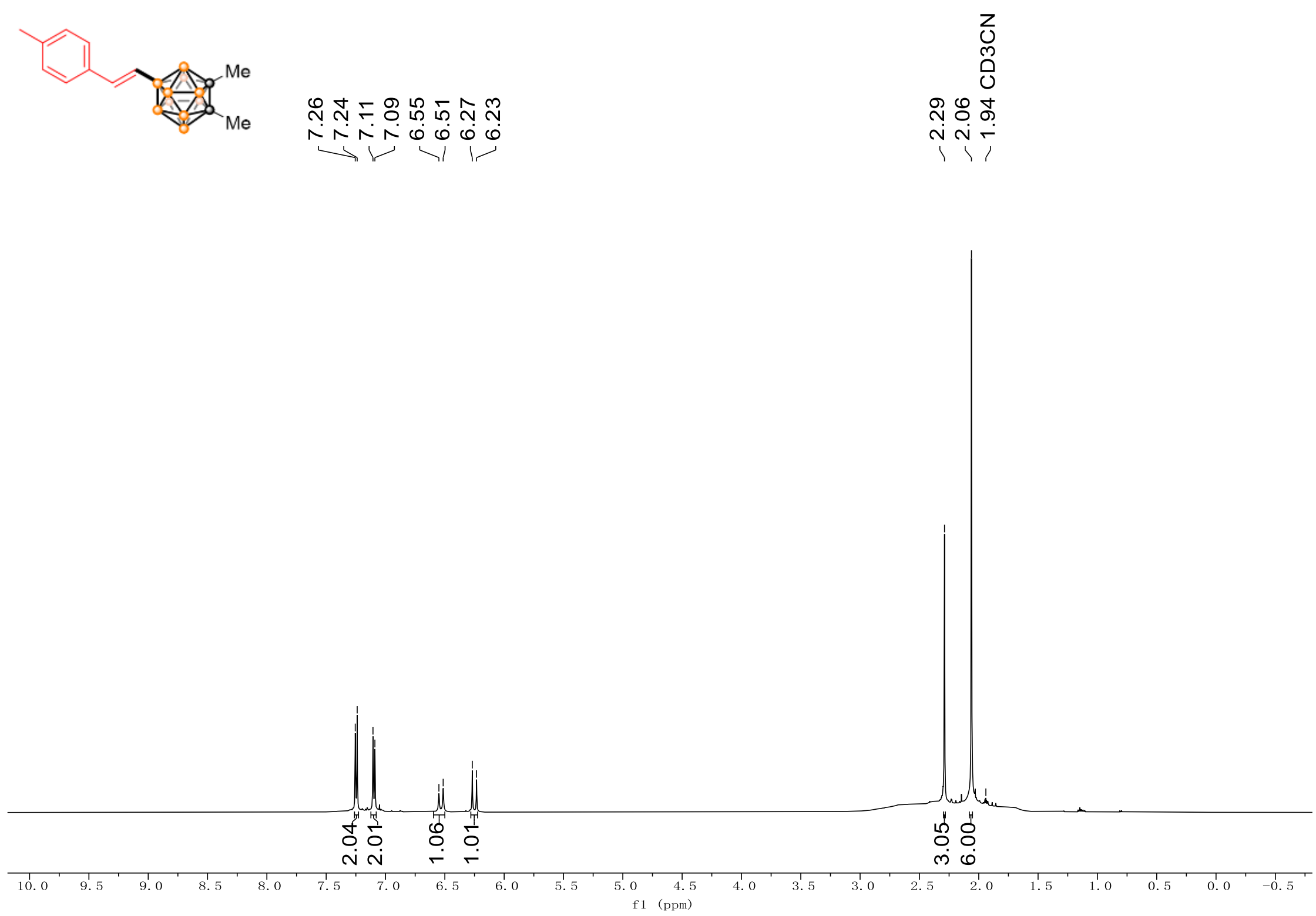

^{1}H NMR (500 MHz, CD_3CN) of **3db**

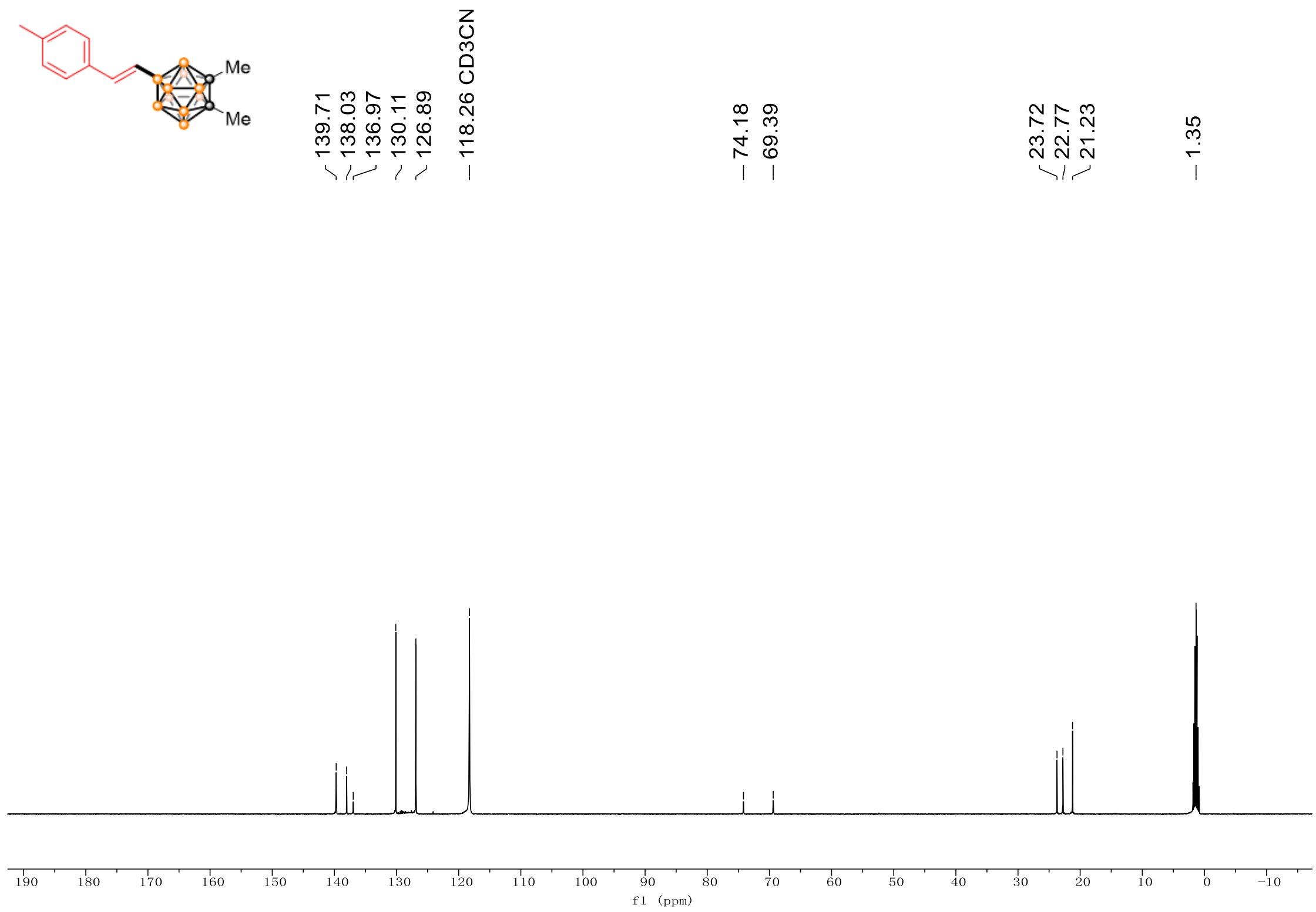

$^{13}C\{^{1}H\}$ NMR (125 MHz, CD_3CN) of **3db**

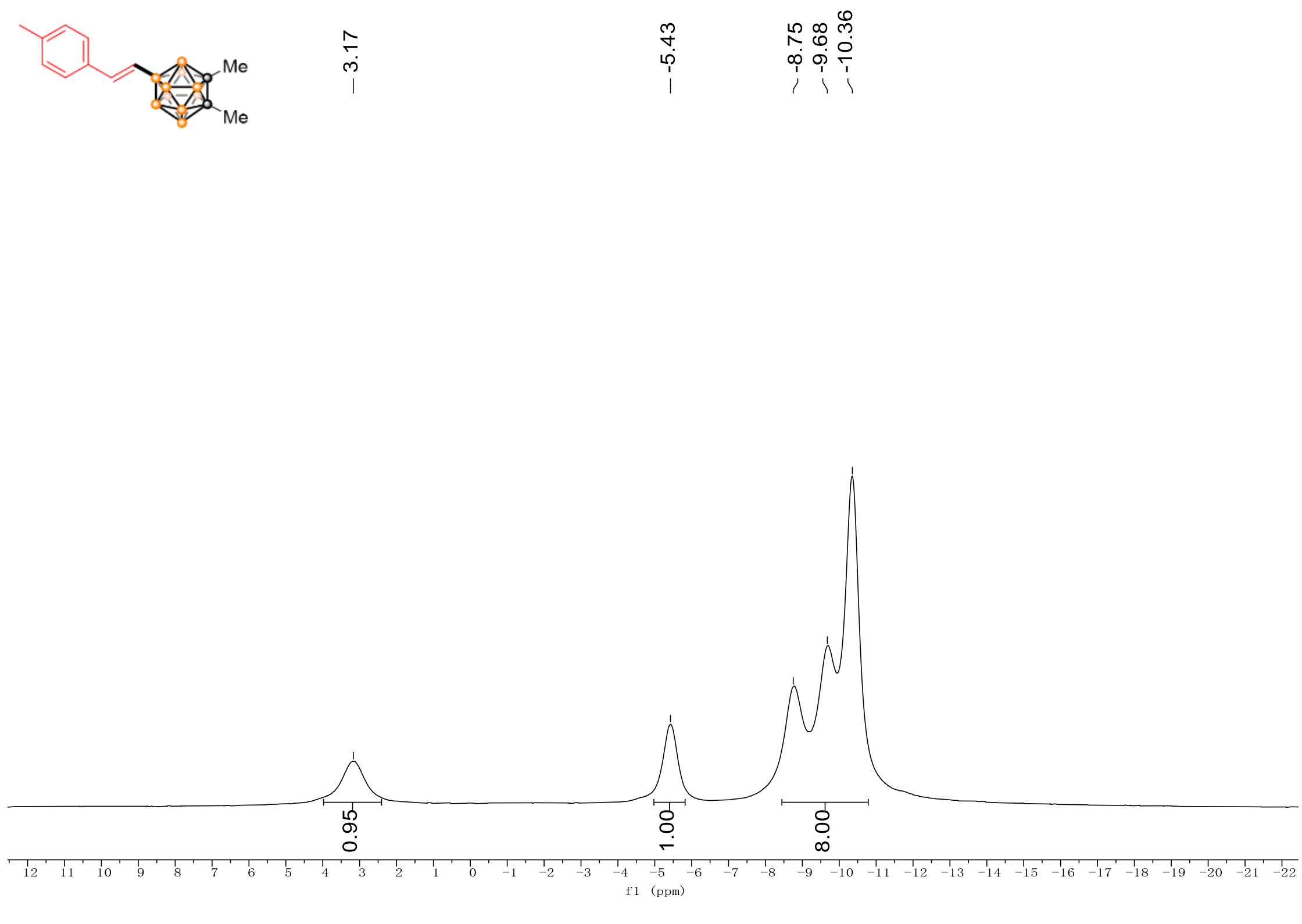

$^{11}B\{^{1}H\}$ NMR (160 MHz, CD_3CN) of **3db**

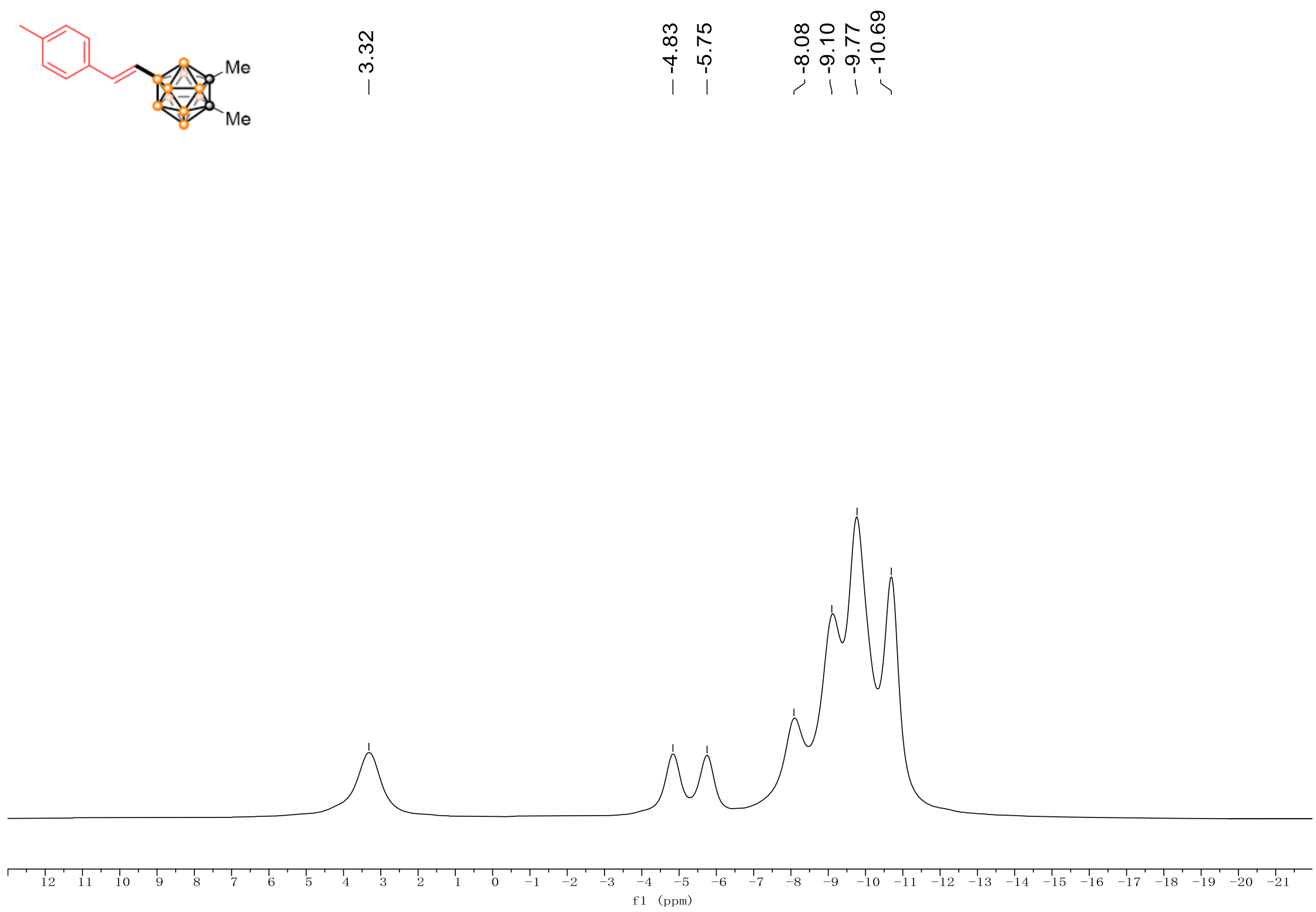

^{11}B NMR (160 MHz, CD_3CN) of **3db**

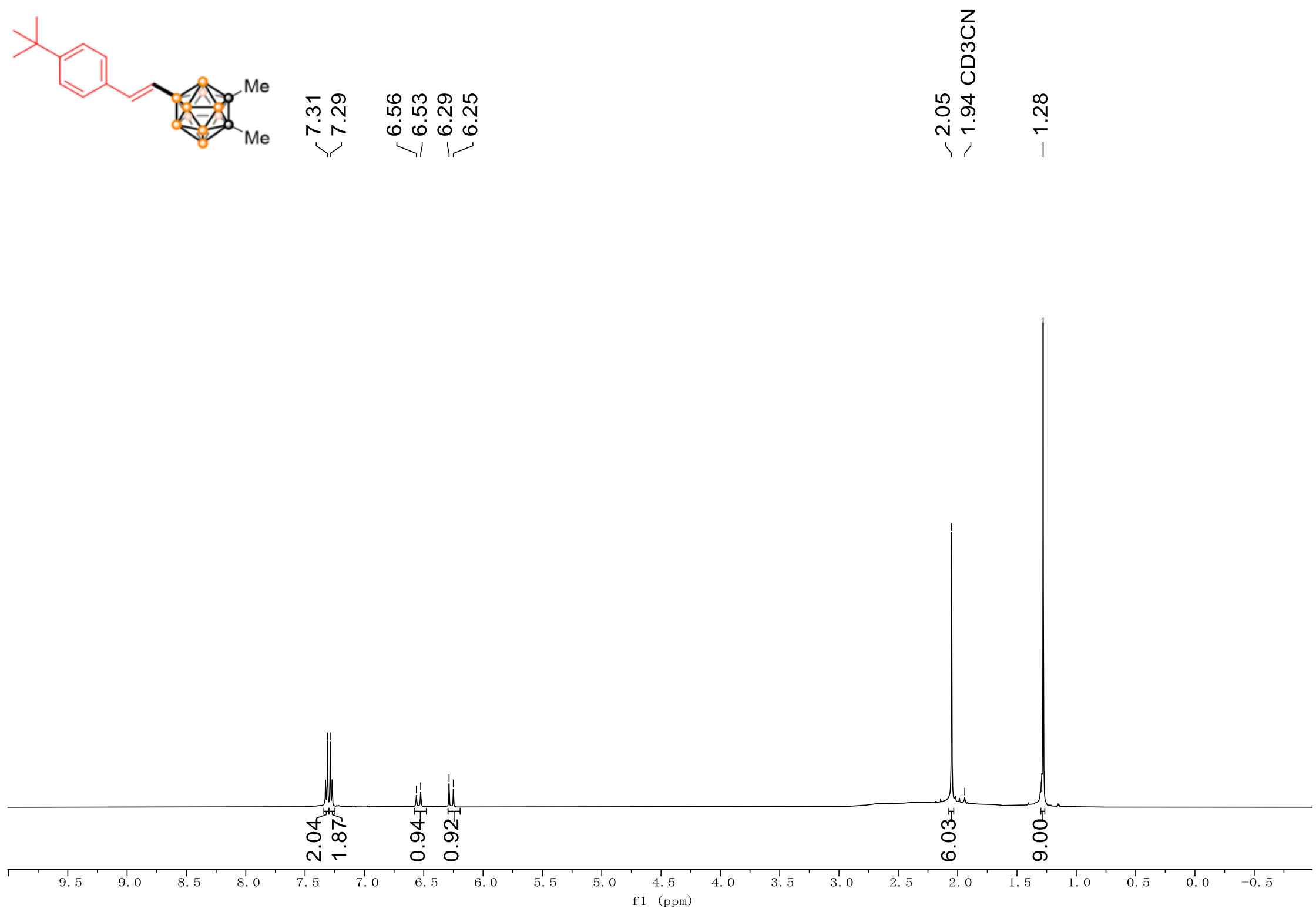

^{1}H NMR (500 MHz, CD_3CN) of **3de**

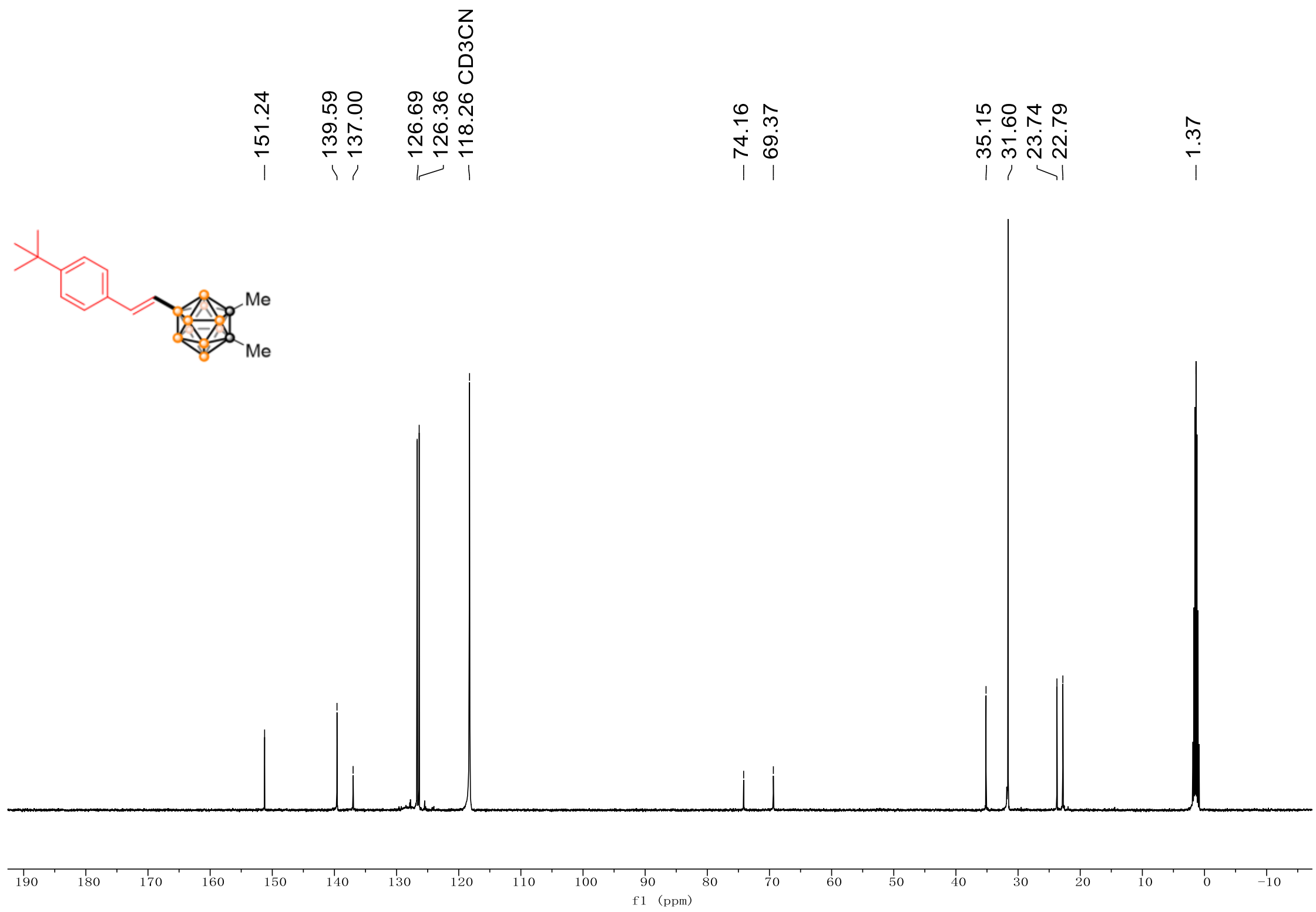

$^{13}C\{^{1}H\}$ NMR (125 MHz, CD_3CN) of **3de**

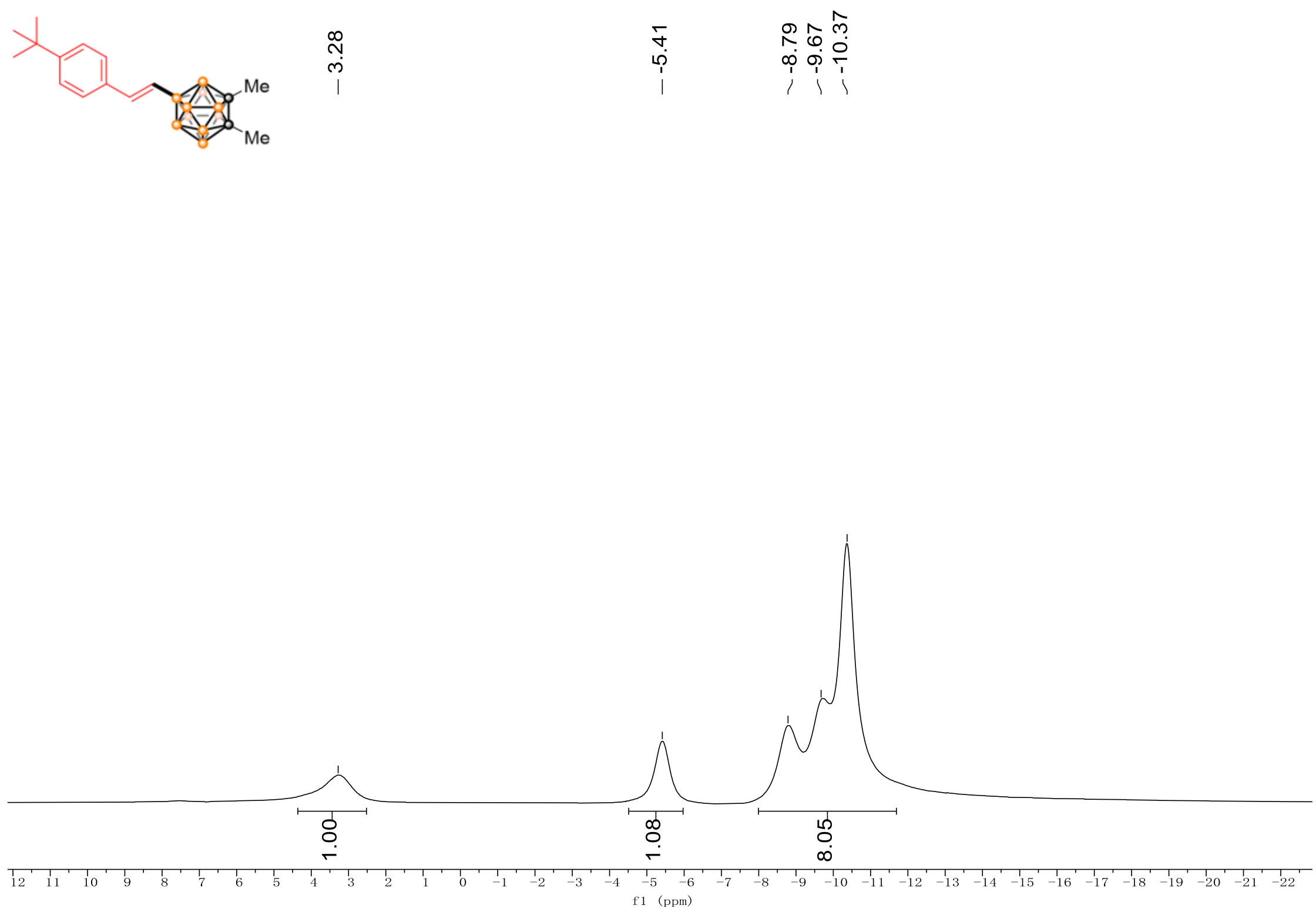

$^{11}B\{^{1}H\}$ NMR (160 MHz, CD_3CN) of **3de**

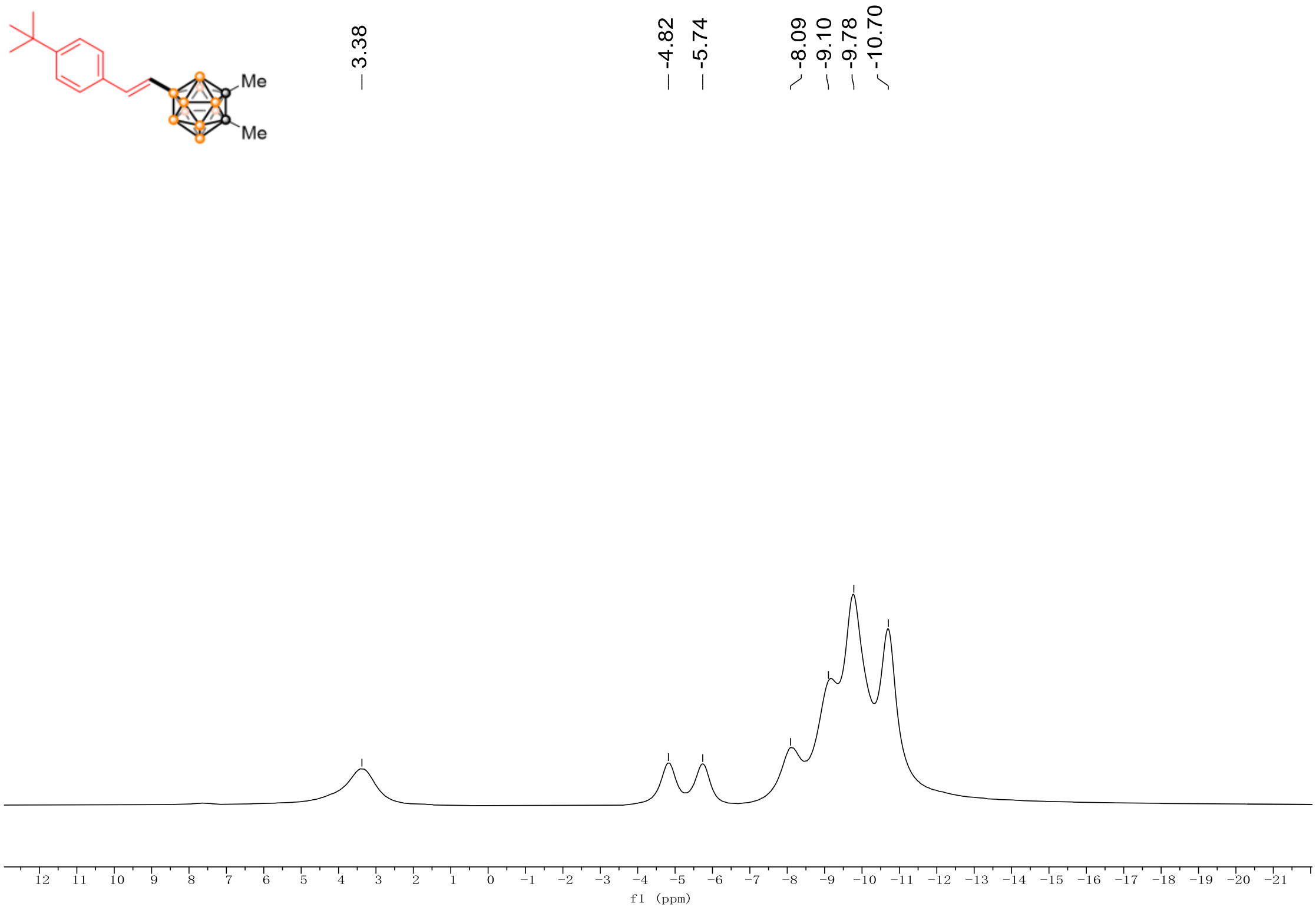

^{11}B NMR (160 MHz, CD_3CN) of **3de**

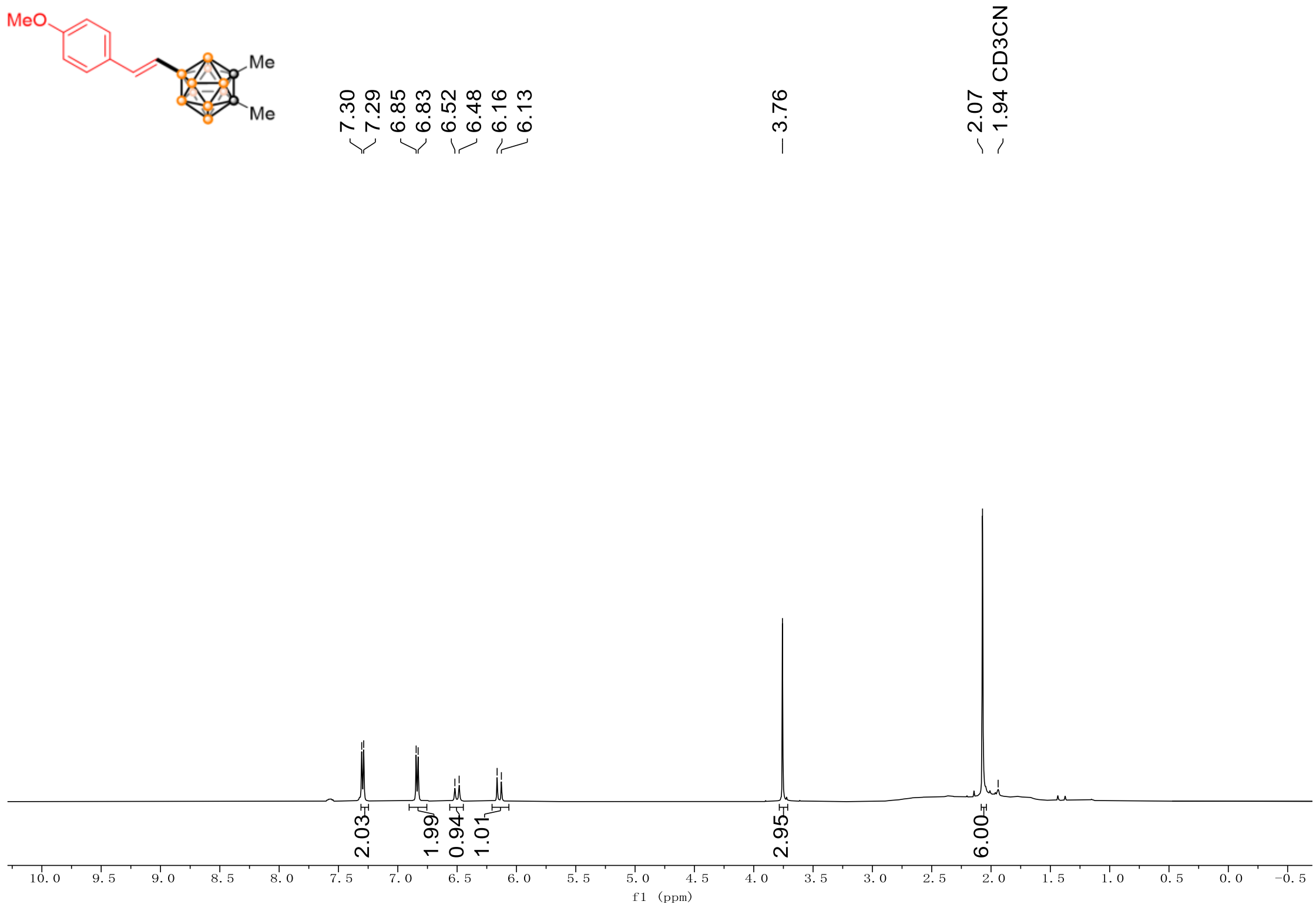

^{1}H NMR (500 MHz, CD_3CN) of **3df**

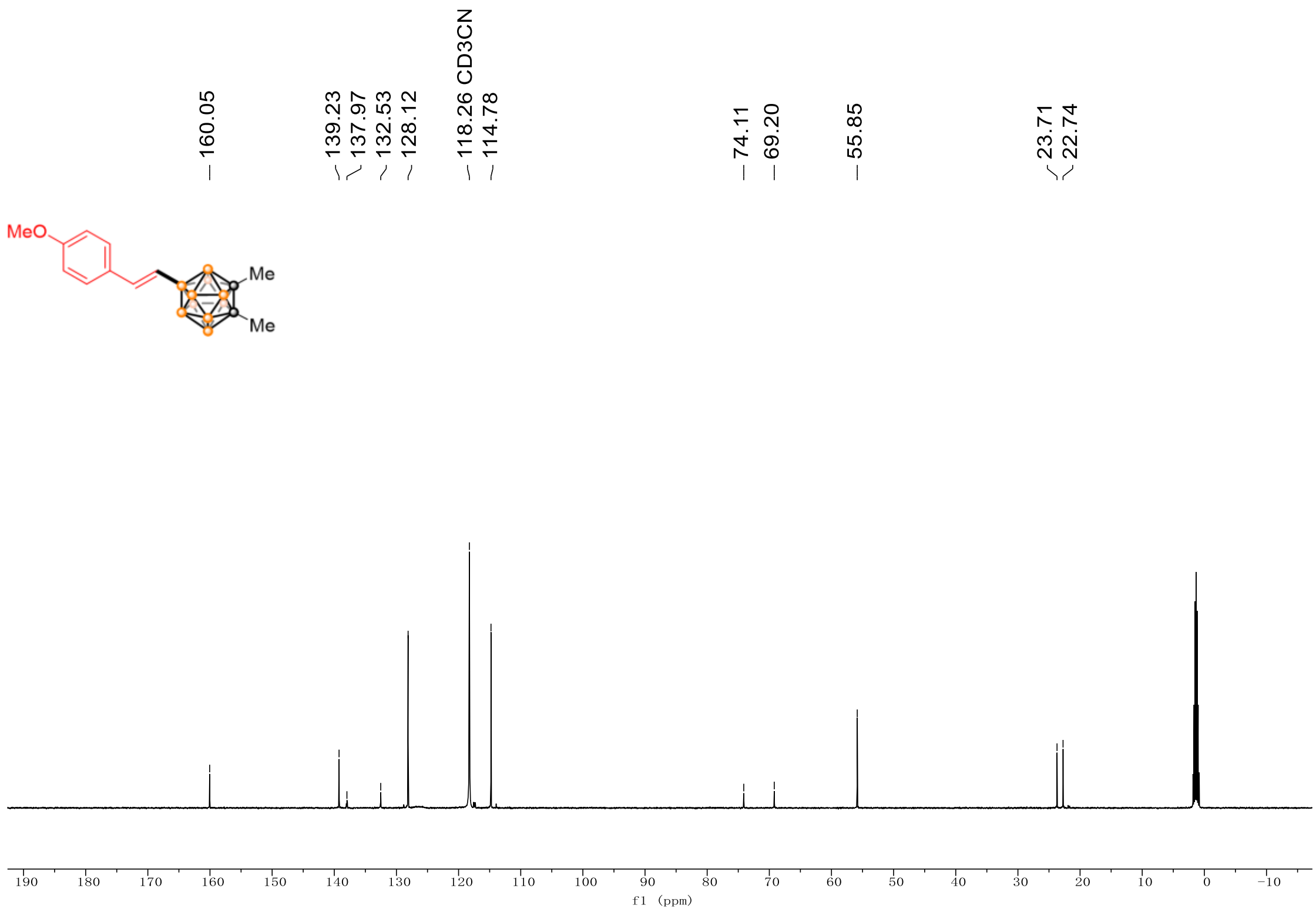

$^{13}C\{^1H\}$ NMR (125 MHz, CD_3CN) of **3df**

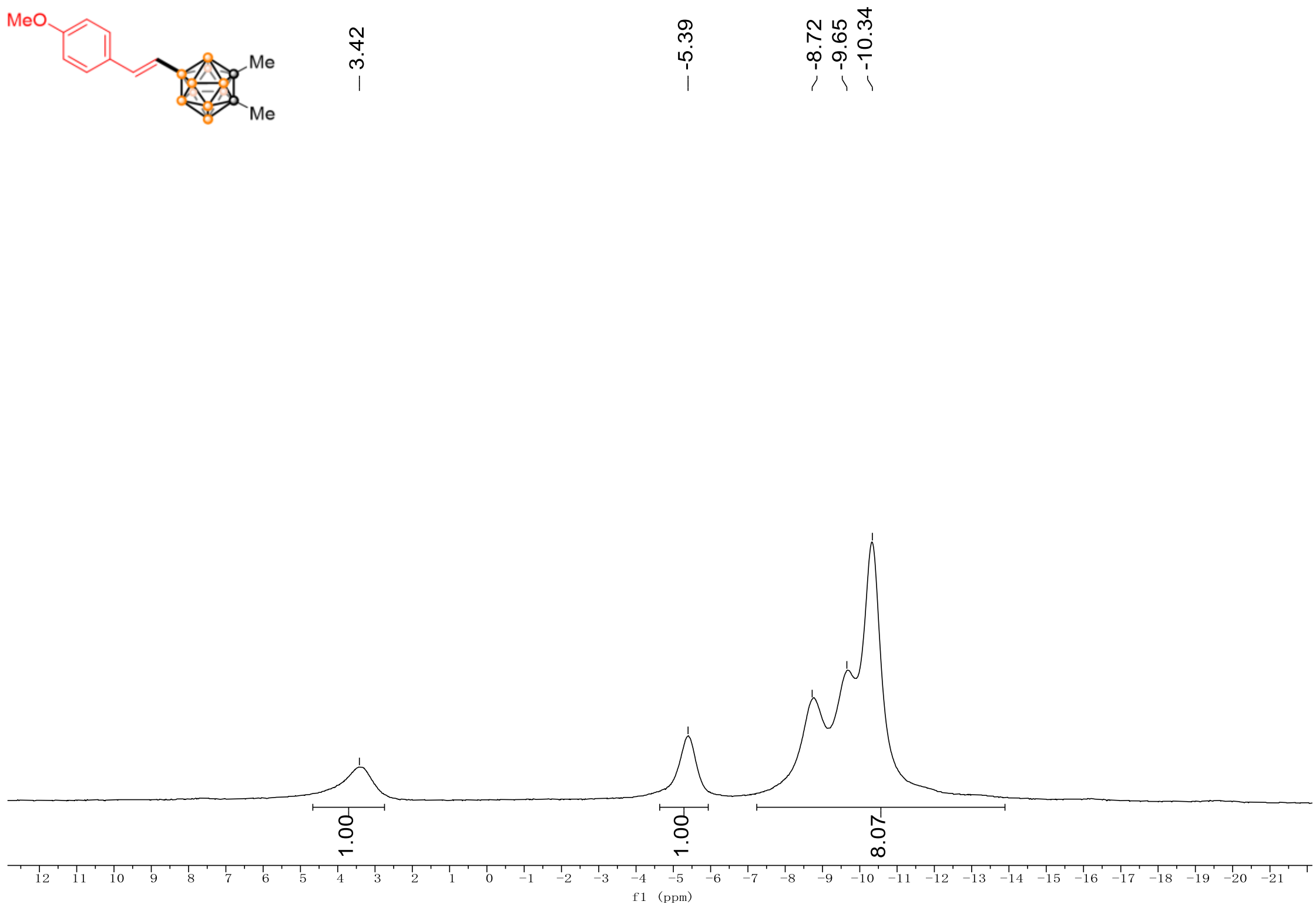

$^{11}B\{^1H\}$ NMR (160 MHz, CD_3CN) of **3df**

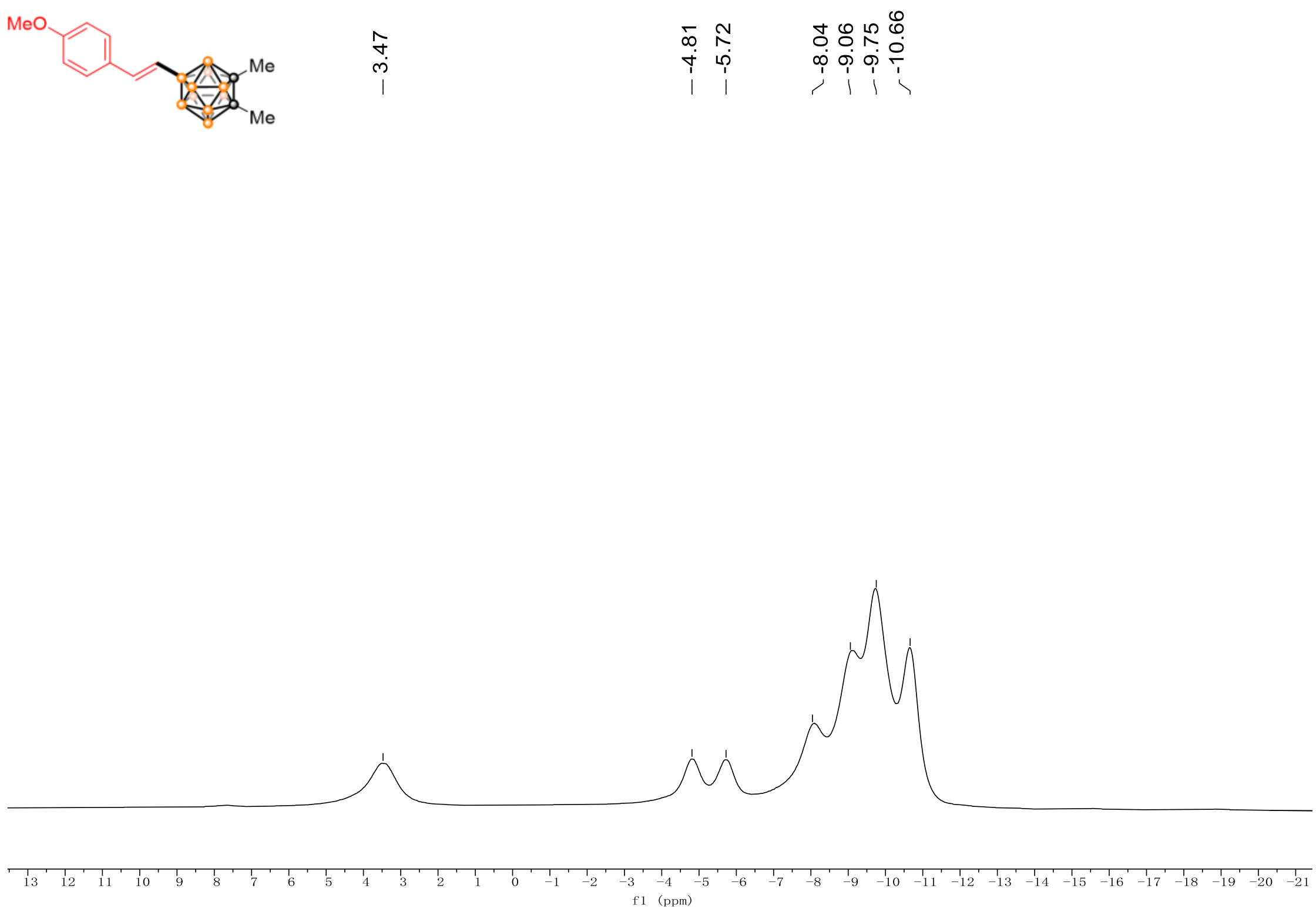

^{11}B NMR (160 MHz, CD_3CN) of **3df**

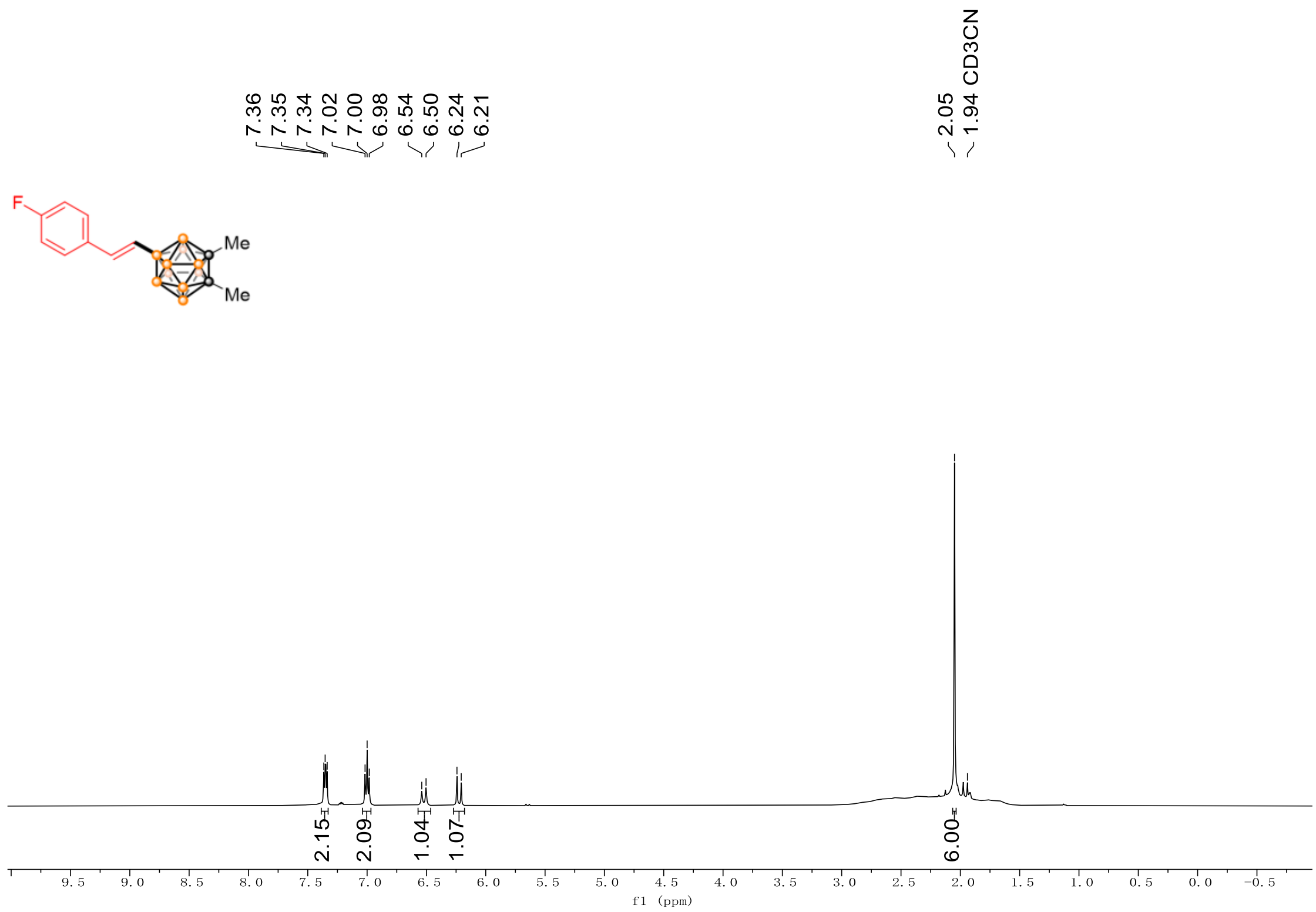

^{1}H NMR (500 MHz, CD_3CN) of **3di**

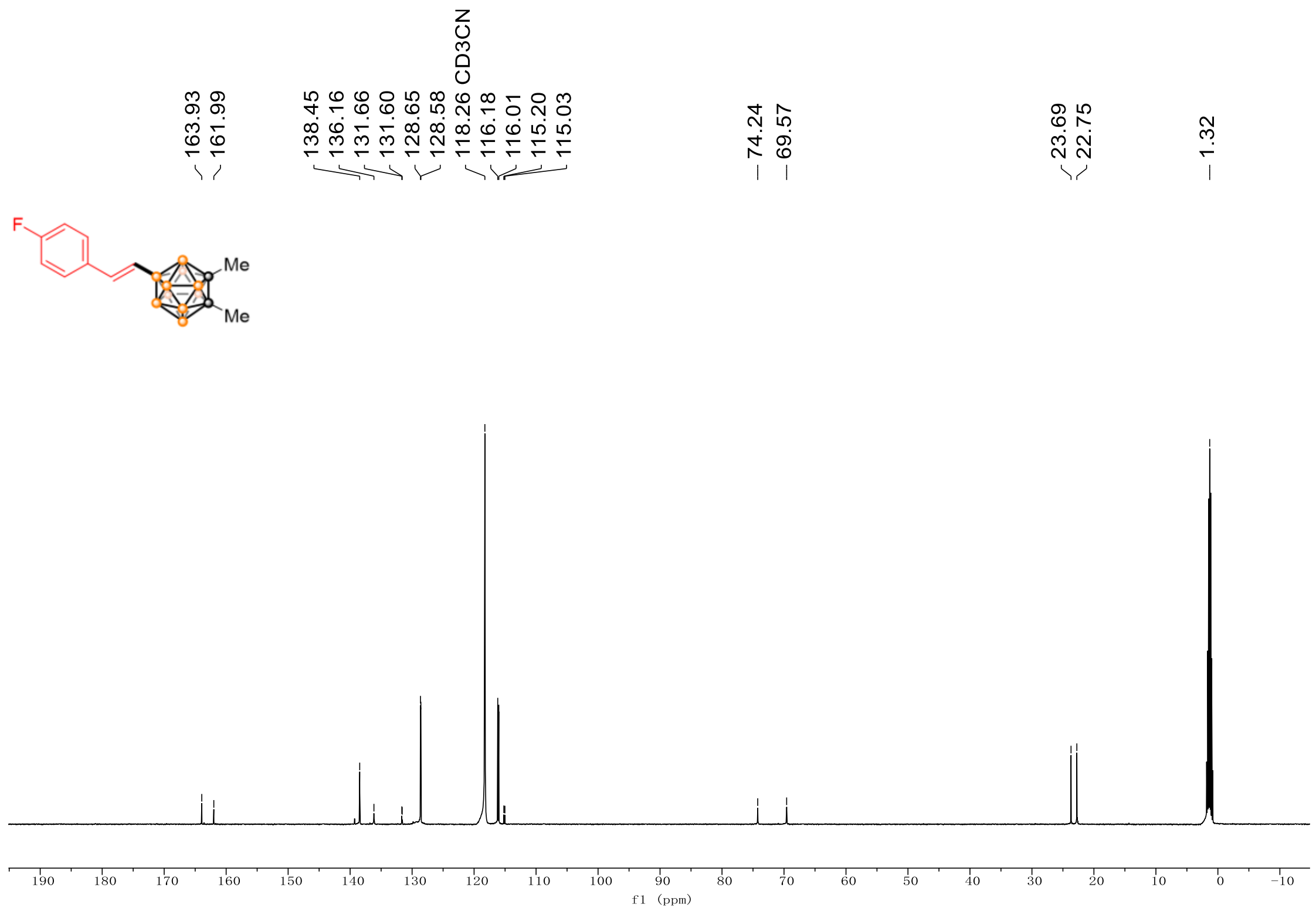

$^{13}C\{^{1}H\}$ NMR (125 MHz, CD_3CN) of **3di**

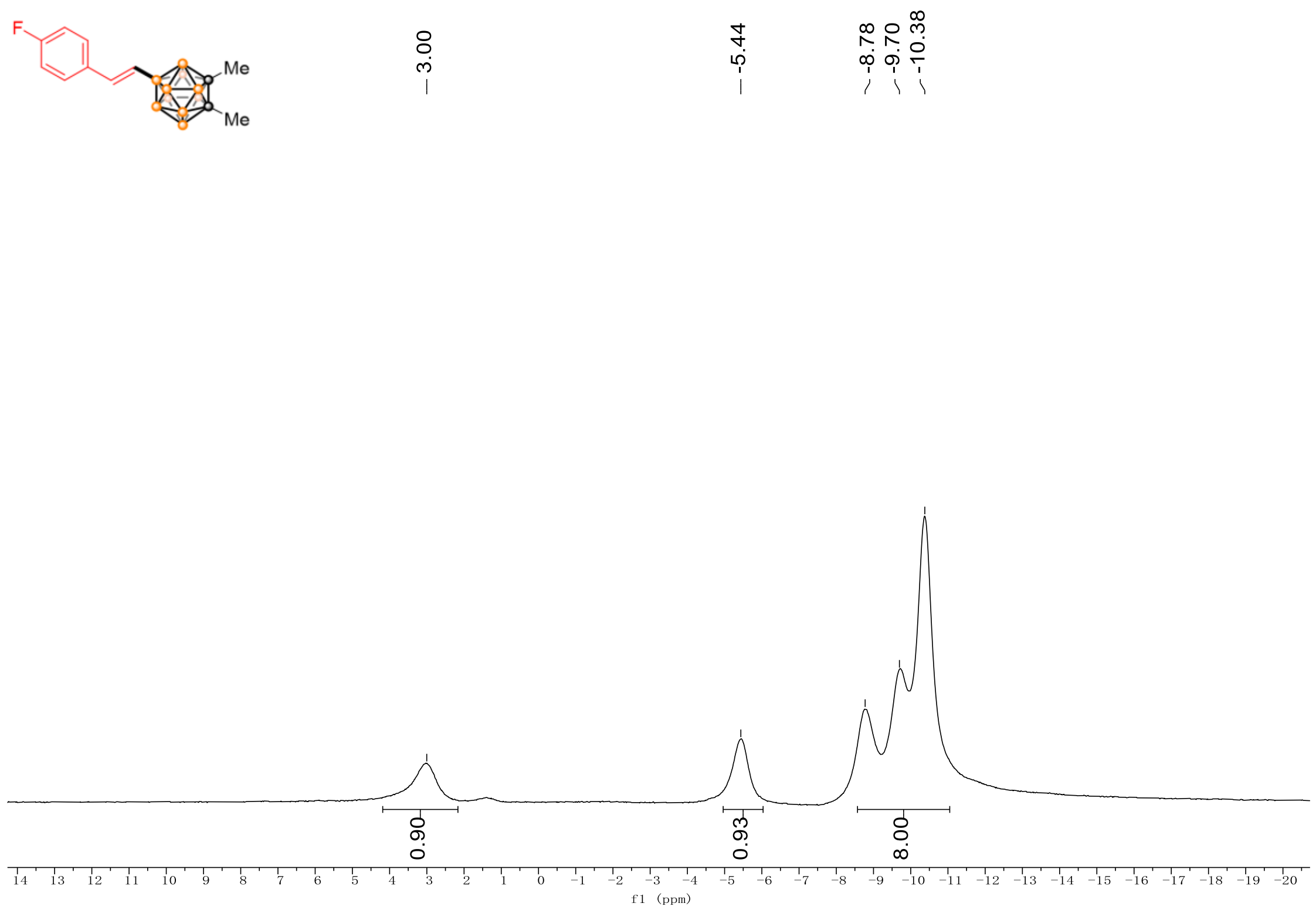

$^{11}B\{^{1}H\}$ NMR (160 MHz, CD_3CN) of **3di**

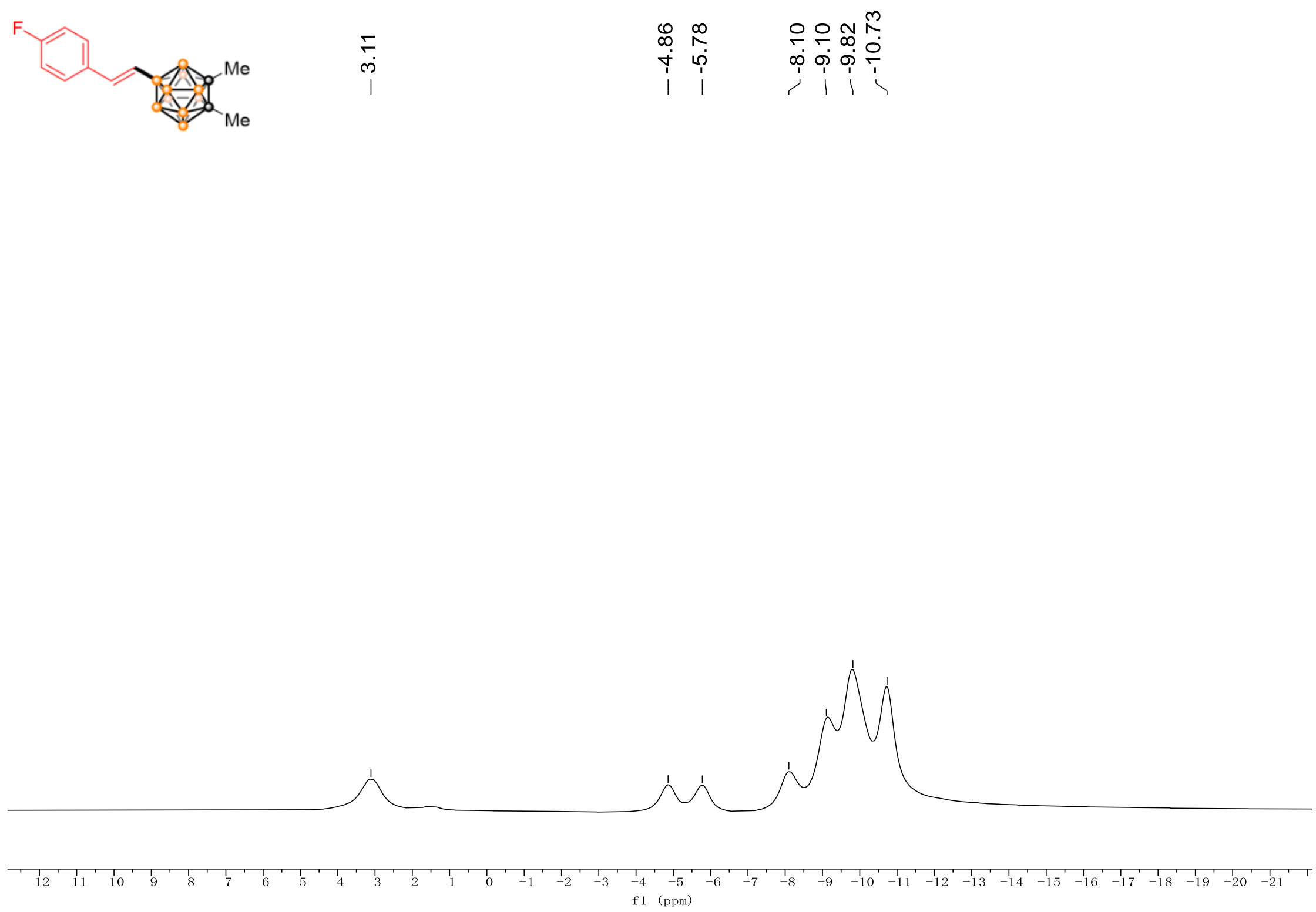

^{11}B NMR (160 MHz, CD_3CN) of **3di**

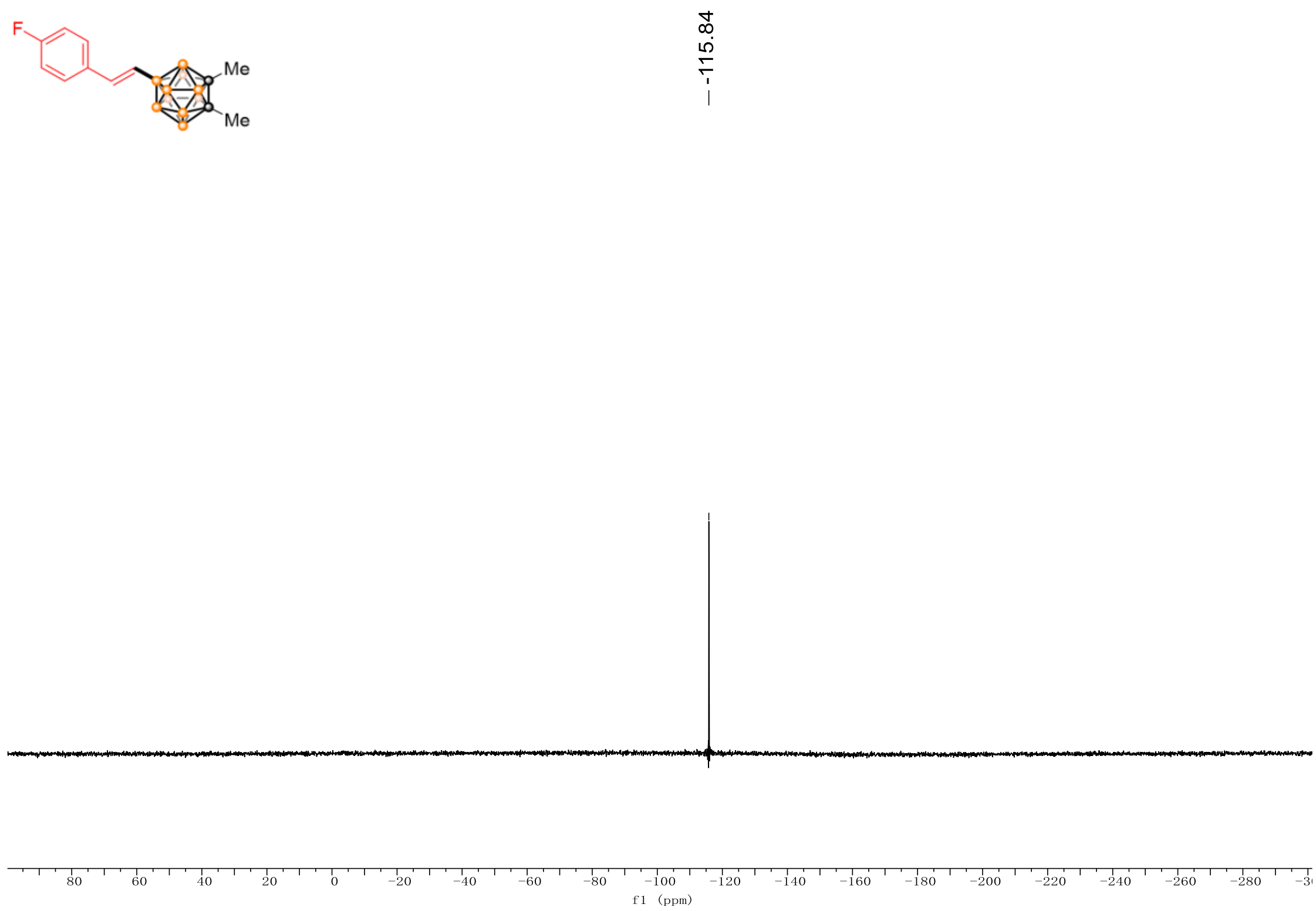

^{19}F NMR (376 MHz, CD_3CN) of **3di**

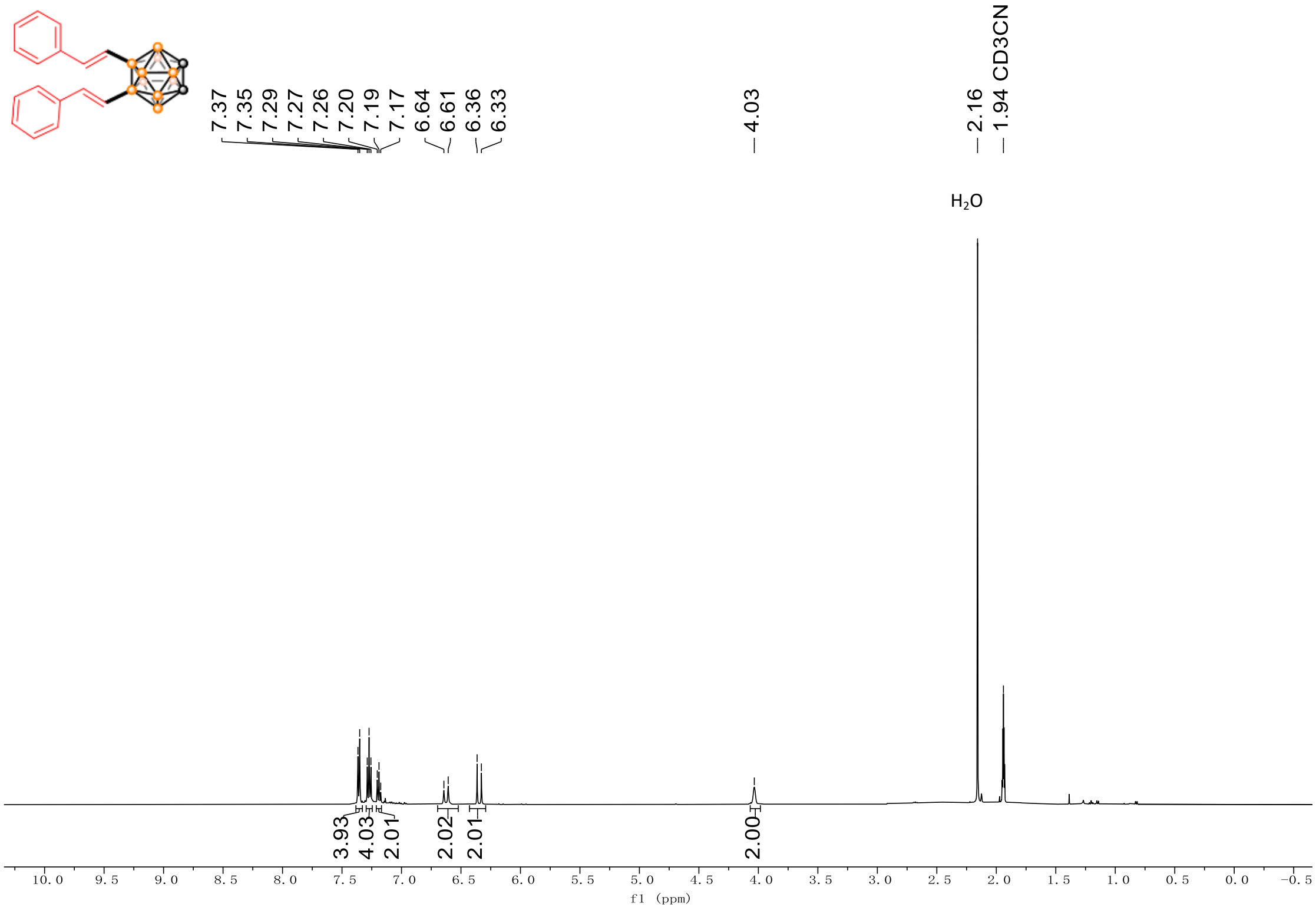

^{1}H NMR (500 MHz, CD_3CN) of **3ea**

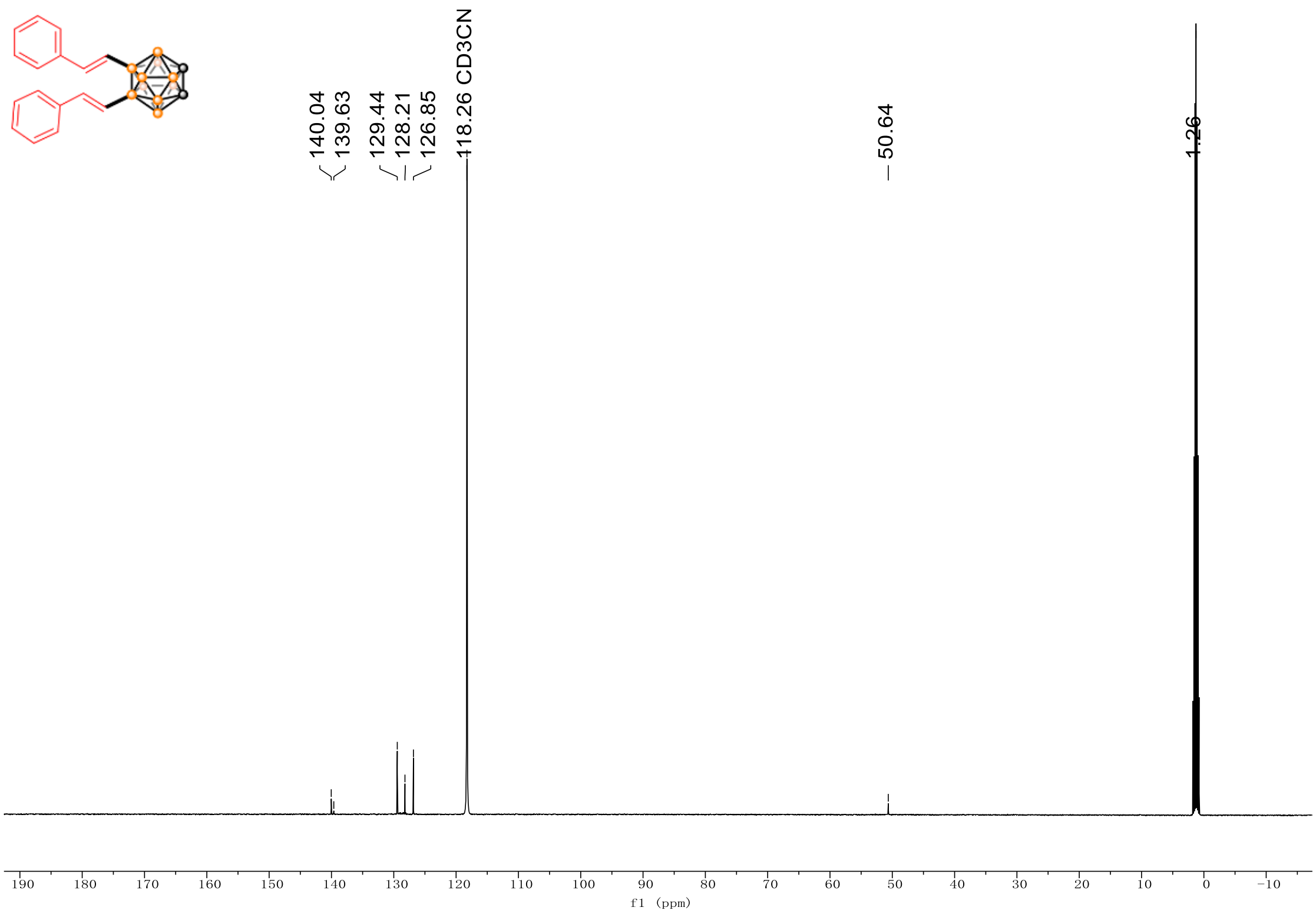

^{13}C{^{1}H} NMR (125 MHz, CD_3CN) of **3ea**

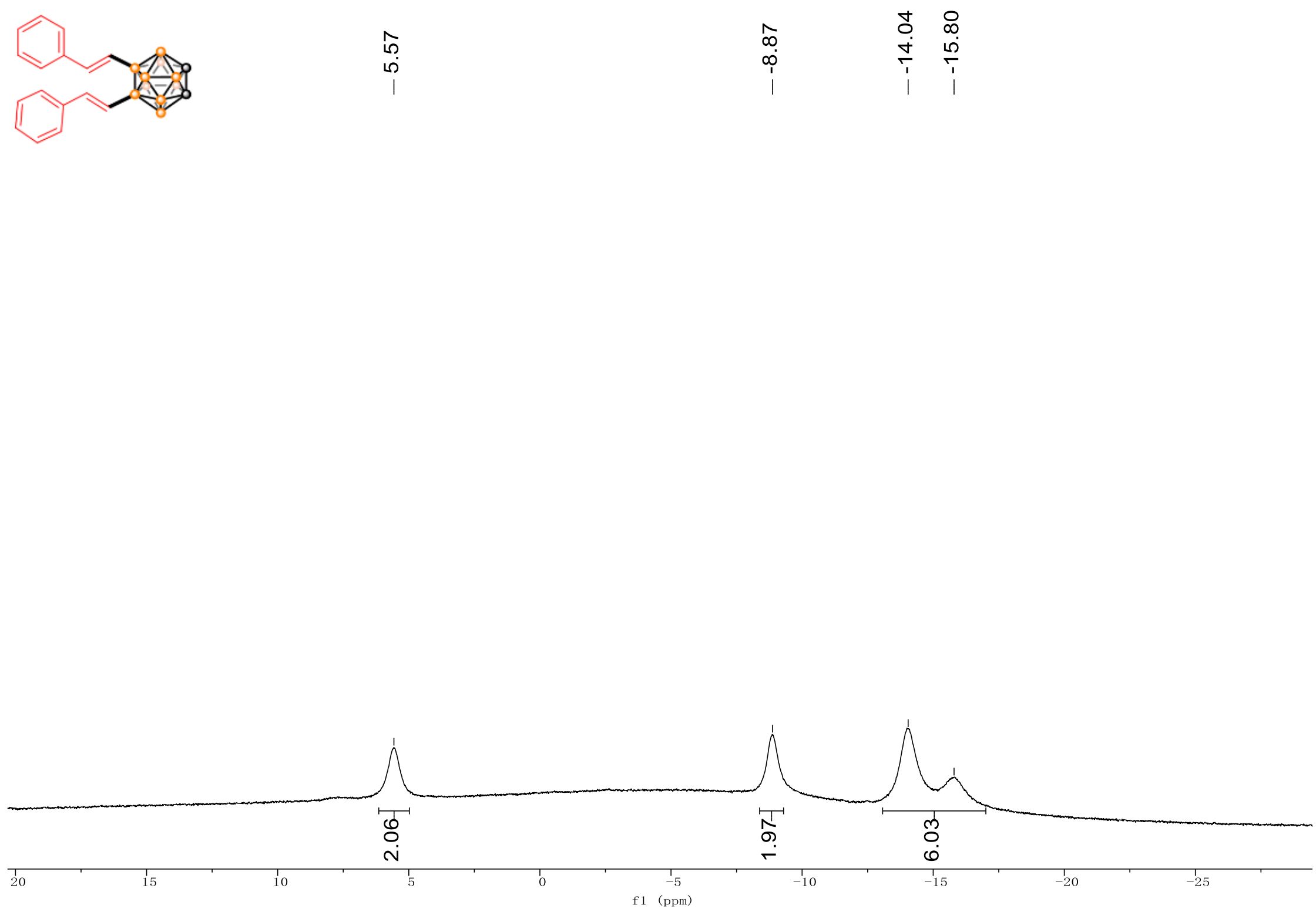

$^{11}B\{^{1}H\}$ NMR (160 MHz, CD_3CN) of **3ea**

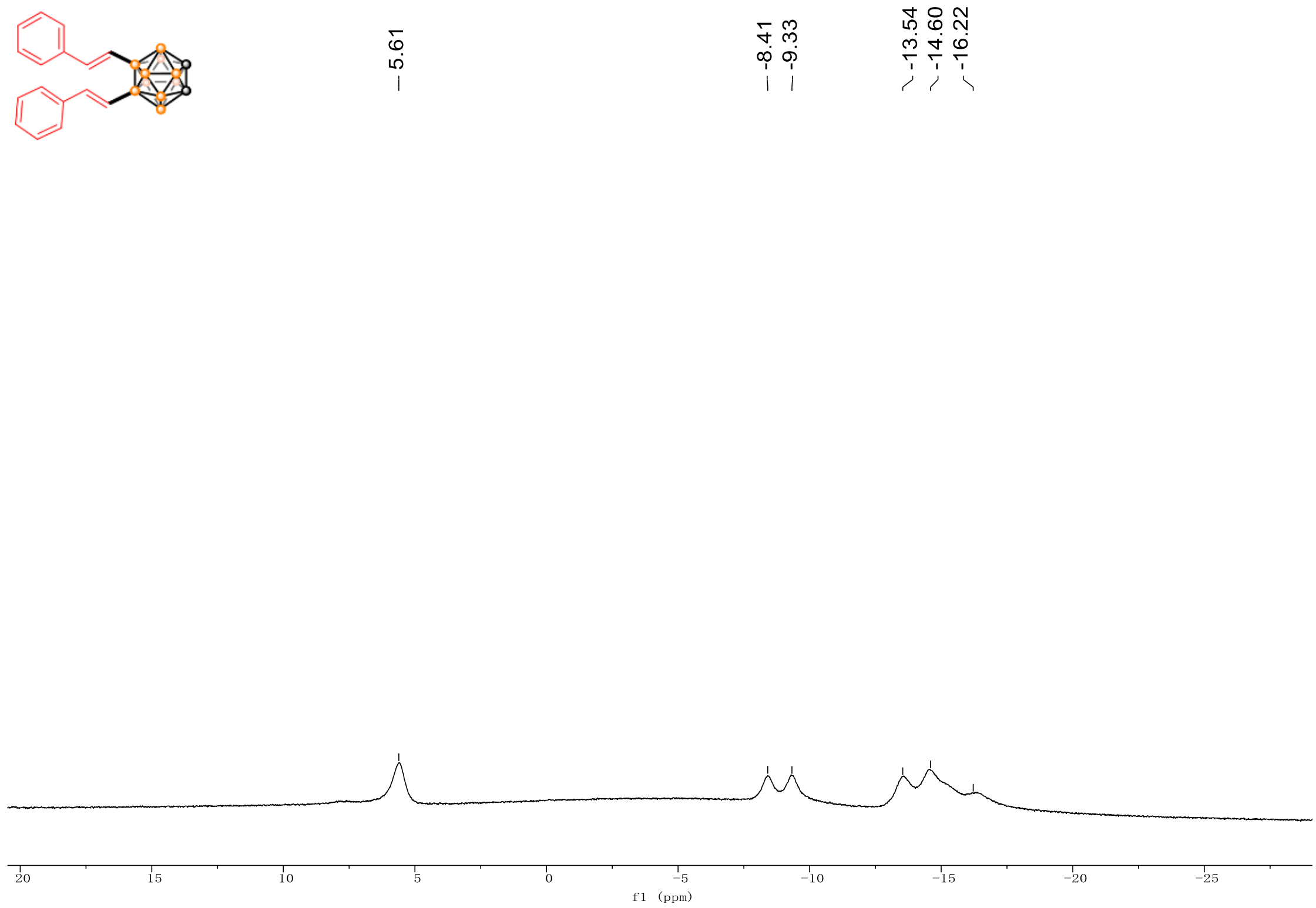

^{11}B NMR (160 MHz, CD_3CN) of **3ea**

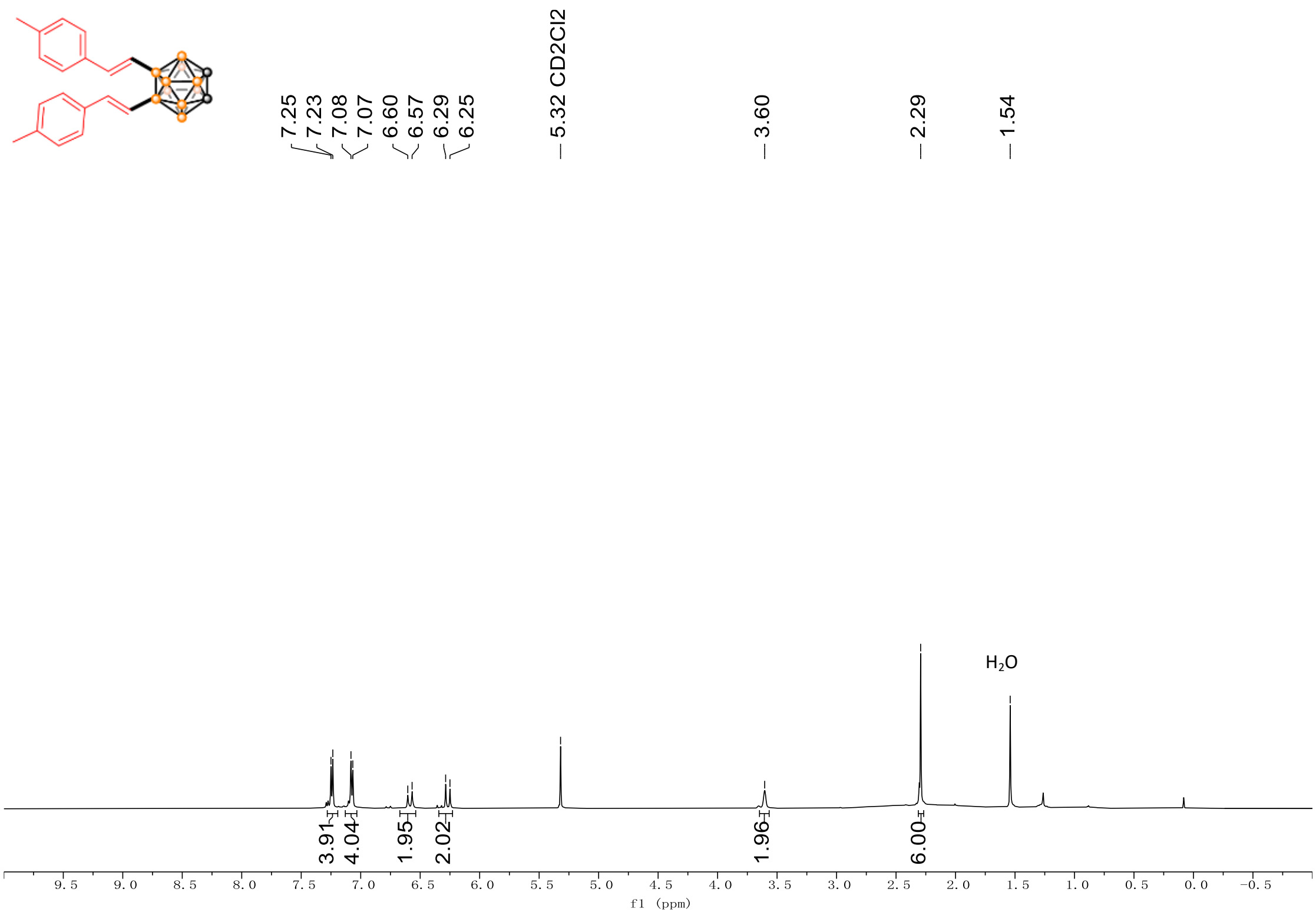

^{1}H NMR (500 MHz, CD_2Cl_2) of **3eb**

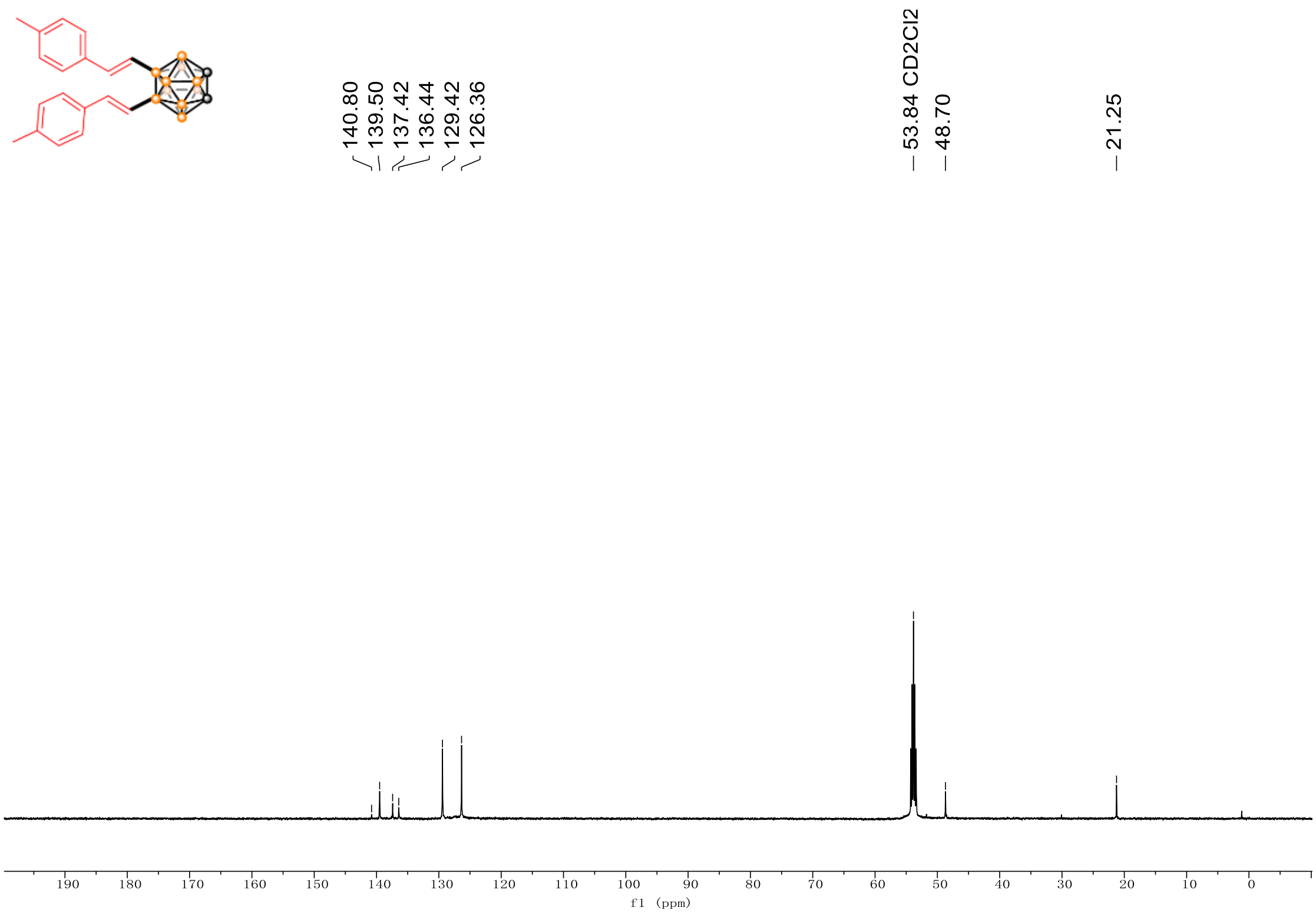

^{13}C{^{1}H} NMR (125 MHz, CD_2Cl_2) of **3eb**

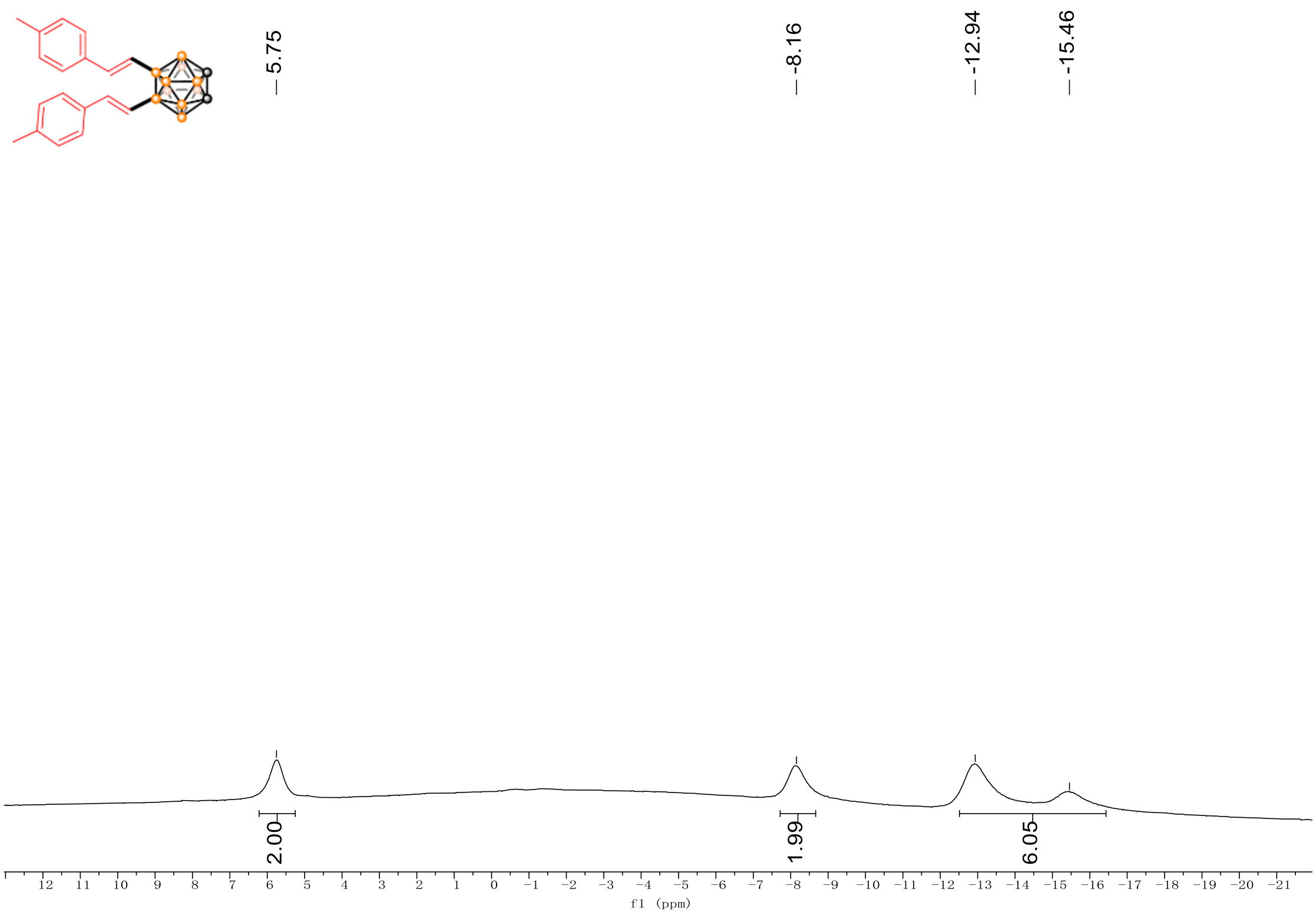

$^{11}B\{^{1}H\}$ NMR (160 MHz, CD_2Cl_2) of **3eb**

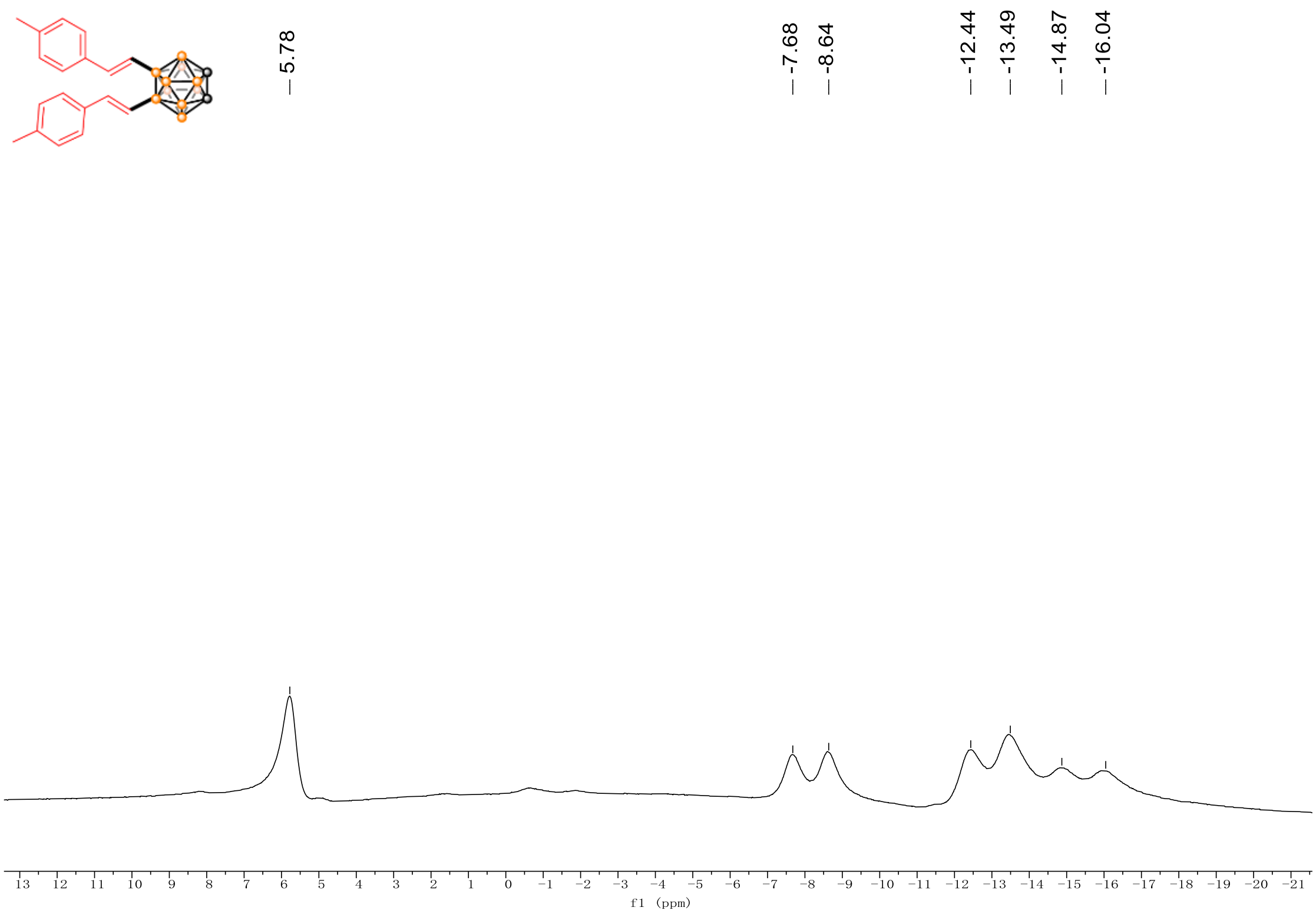

^{11}B NMR (160 MHz, CD_2Cl_2) of **3eb**

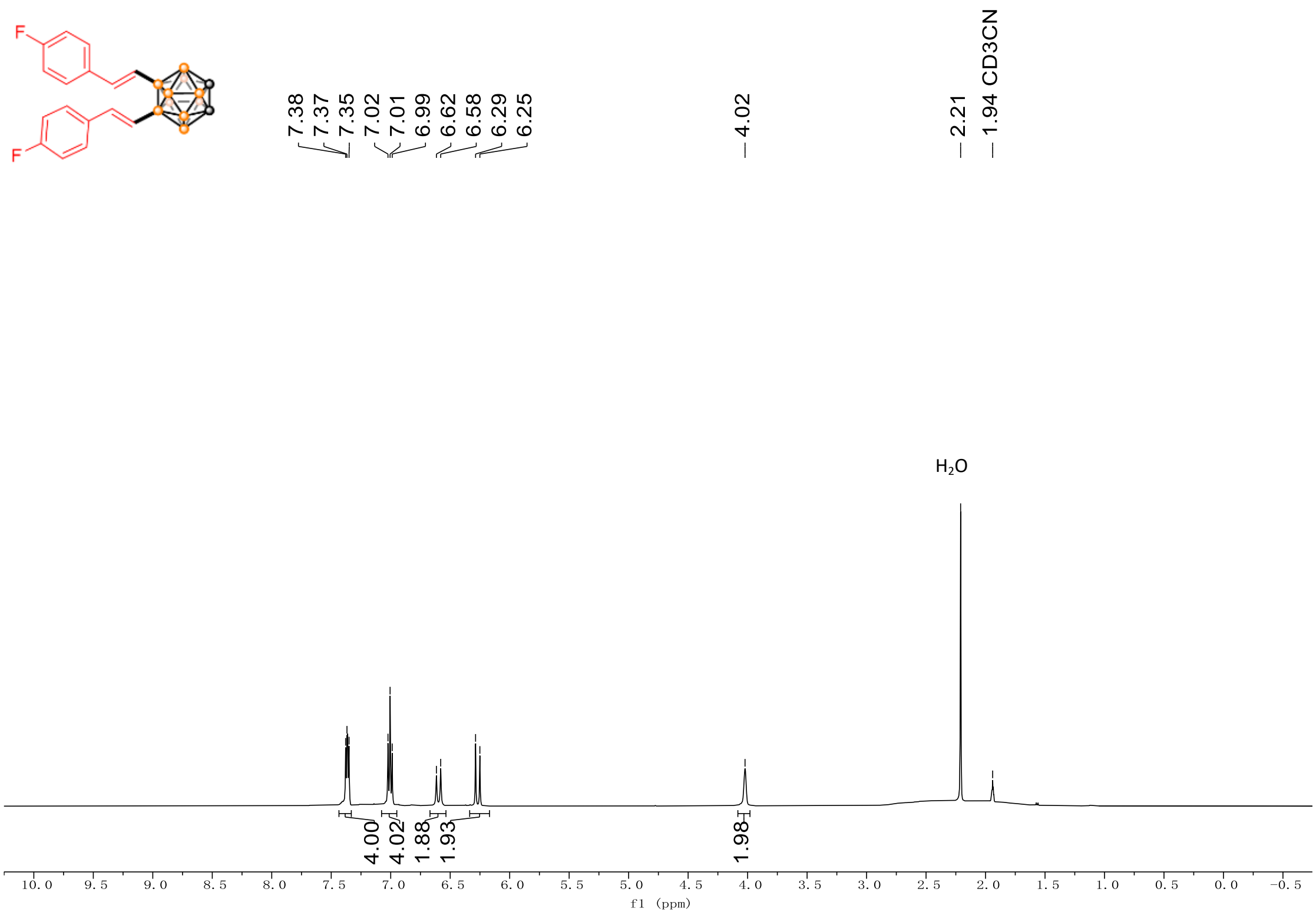

^{1}H NMR (500 MHz, CD_3CN) of **3ei**

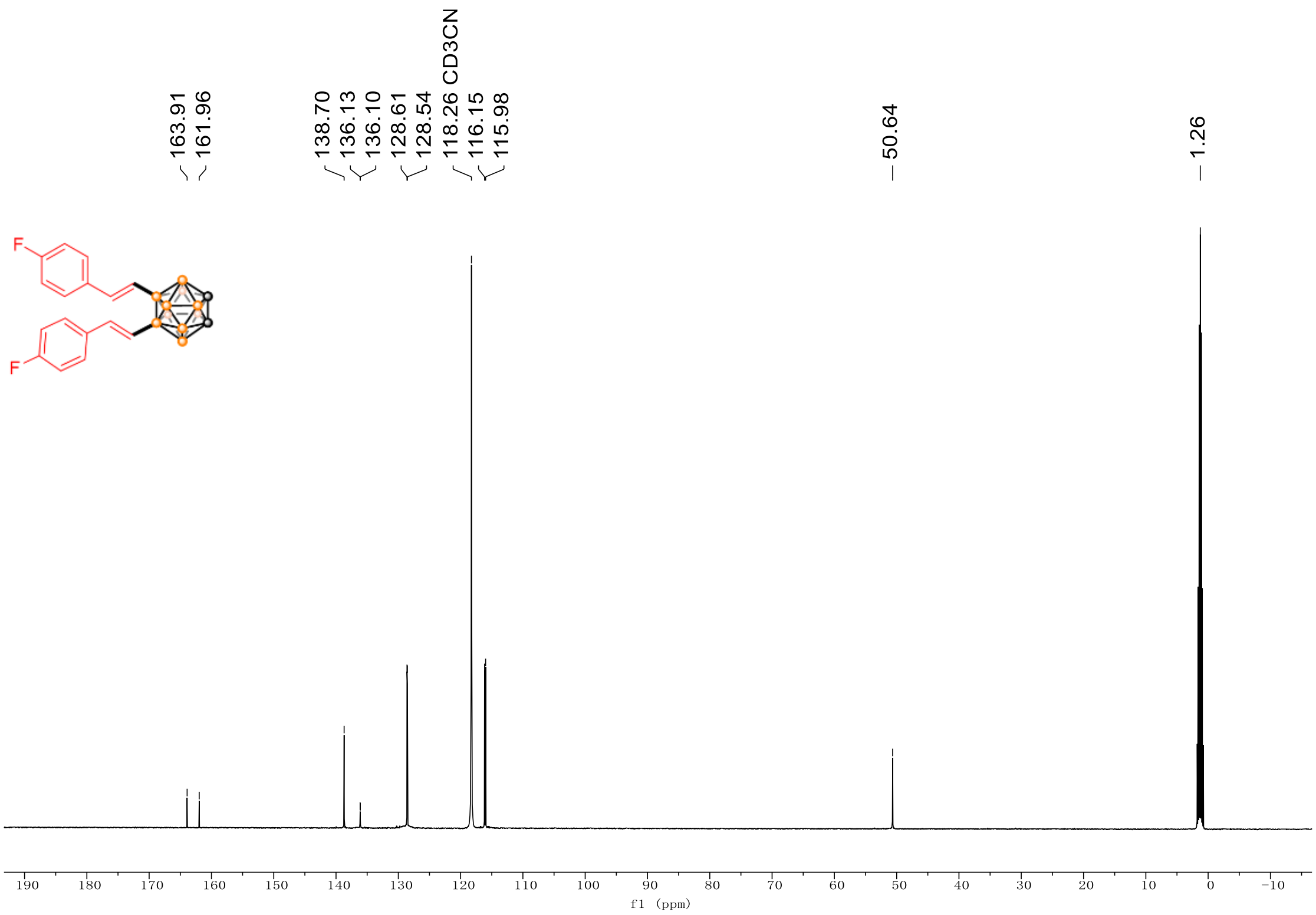

$^{13}C\{^{1}H\}$ NMR (125 MHz, CD_3CN) of **3ei**

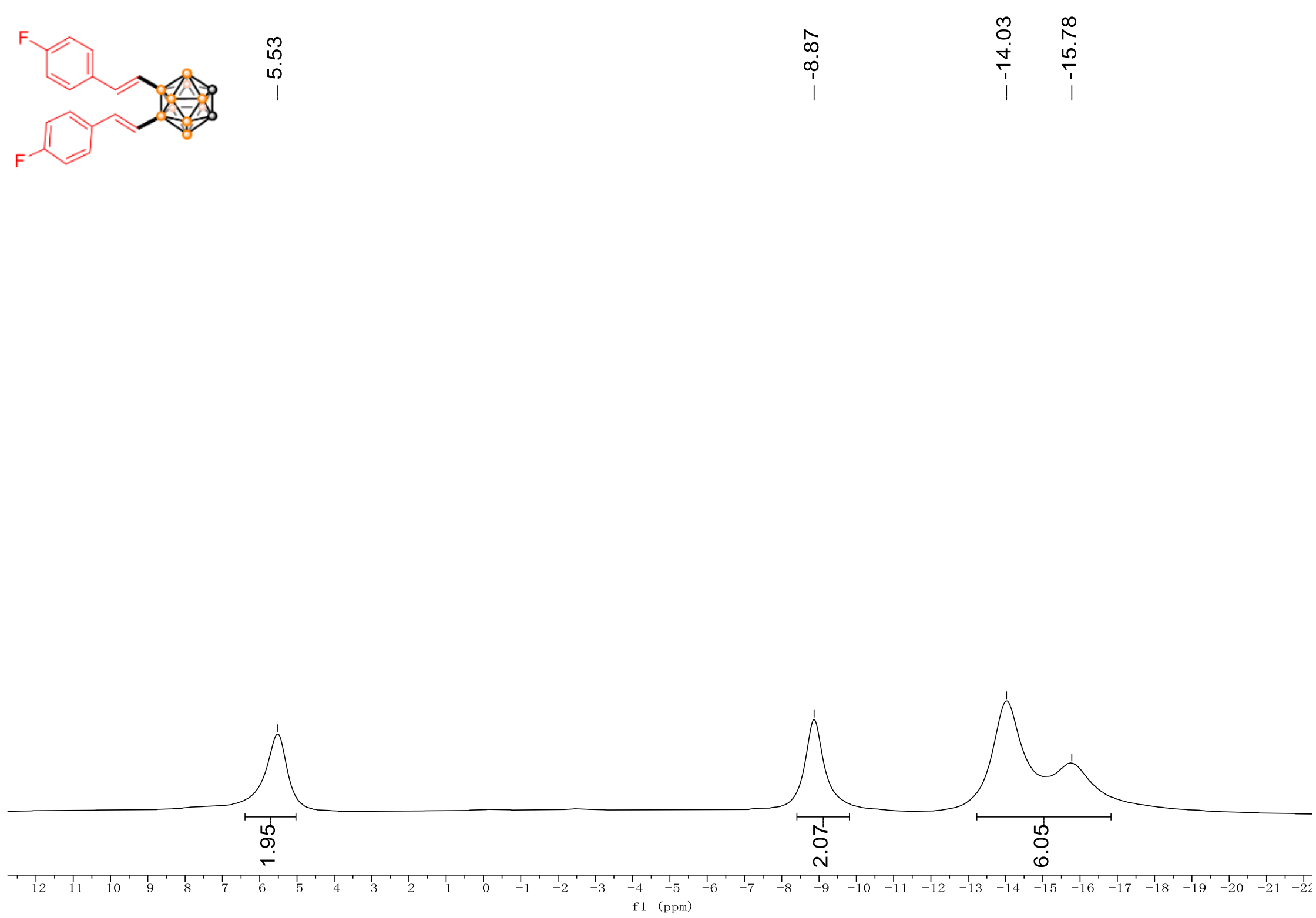

$^{11}B\{^{1}H\}$ NMR (160 MHz, CD_3CN) of **3ei**

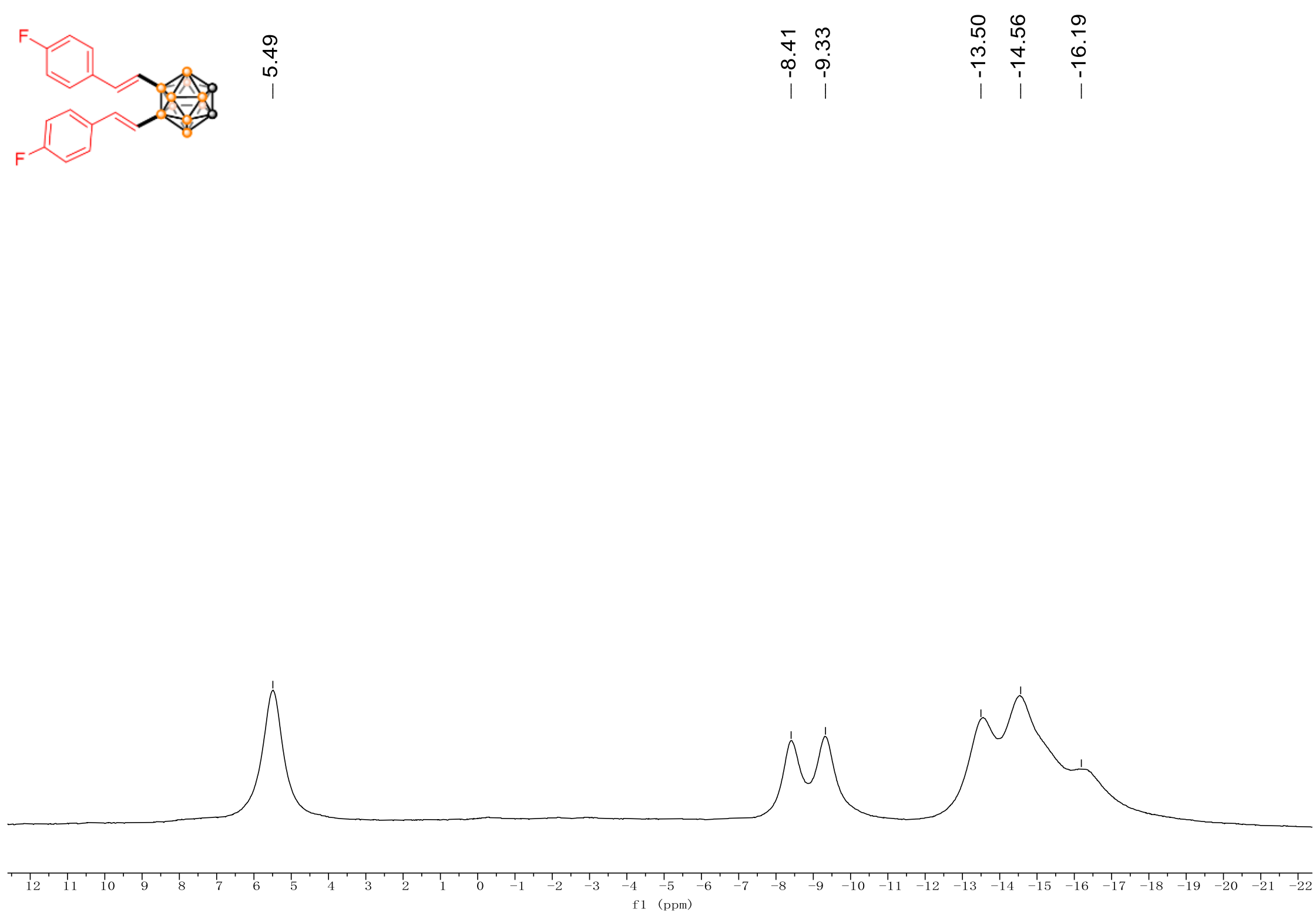

^{11}B NMR (160 MHz, CD_3CN) of **3ei**

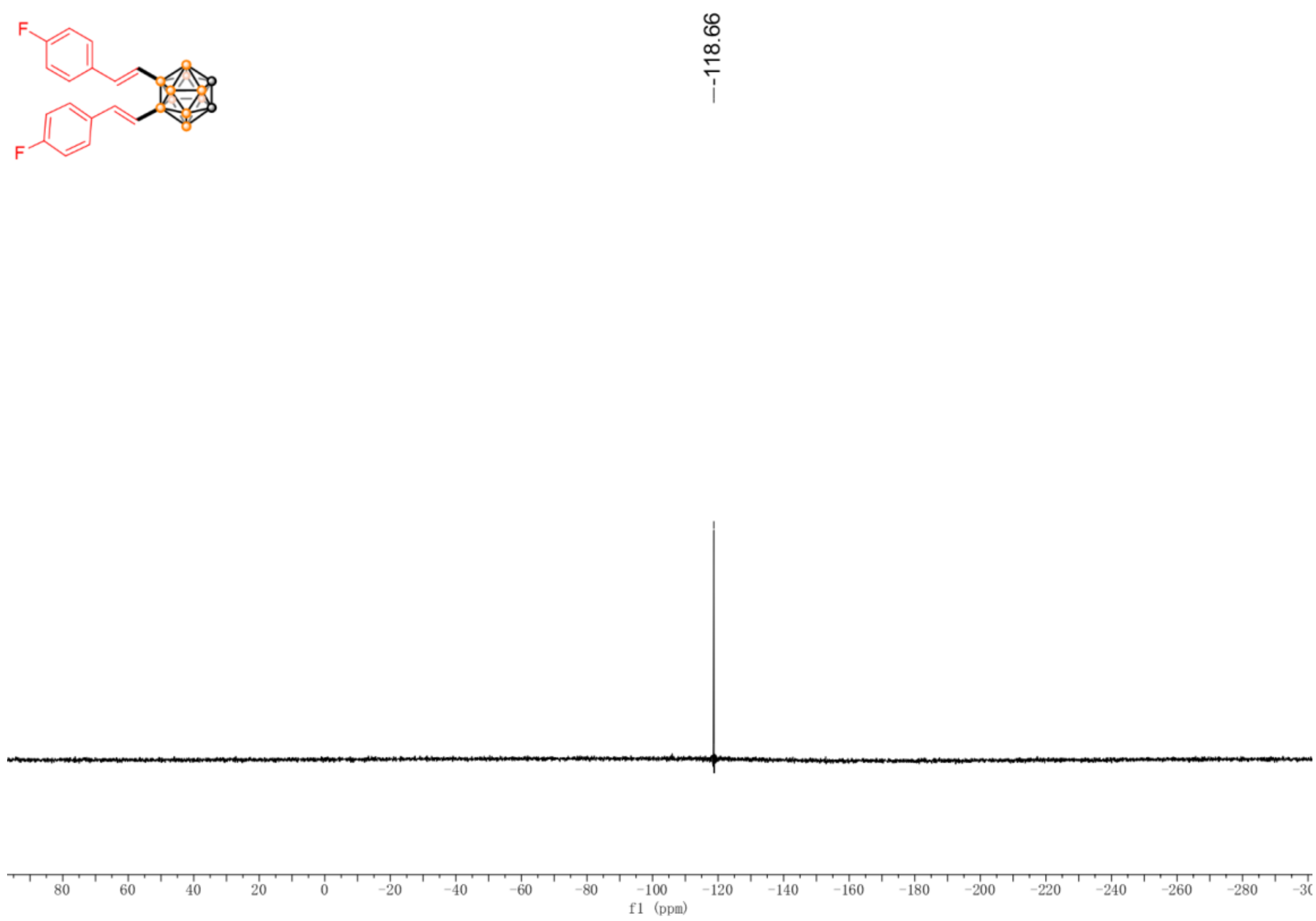

^{19}F NMR (376 MHz, CD_3CN) of **3ei**

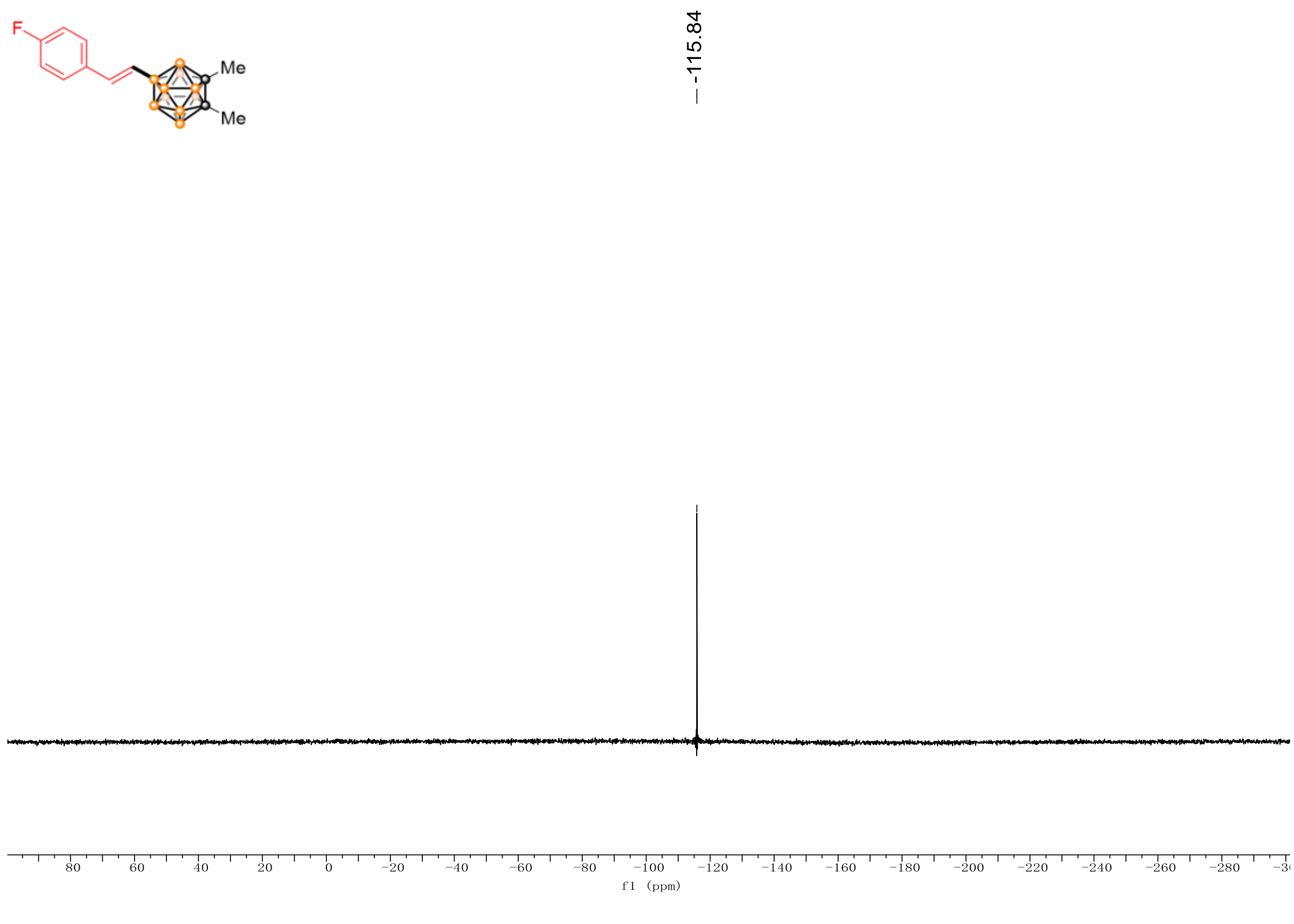

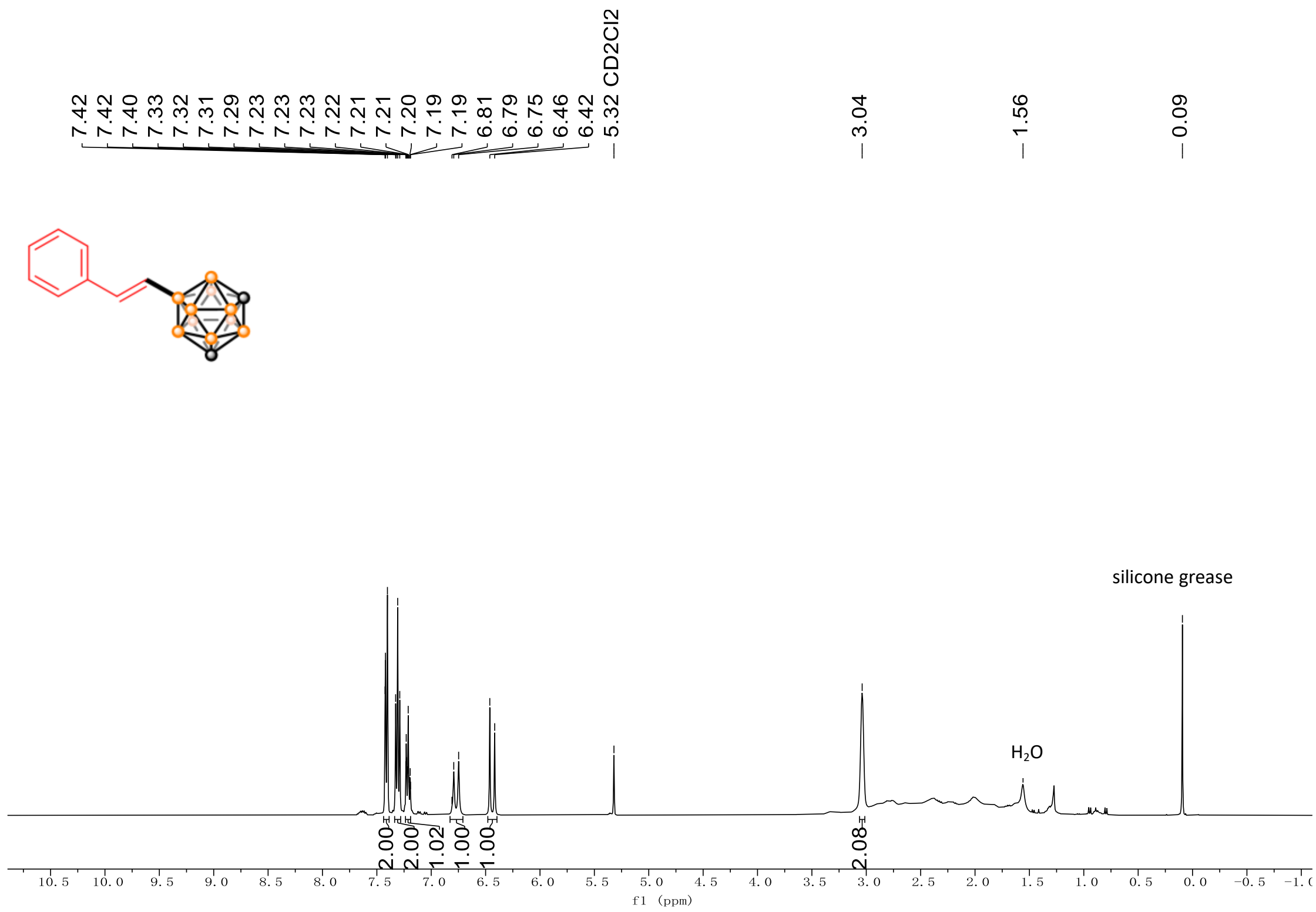

^{1}H NMR (400 MHz, CD_2Cl_2) of **3fa**

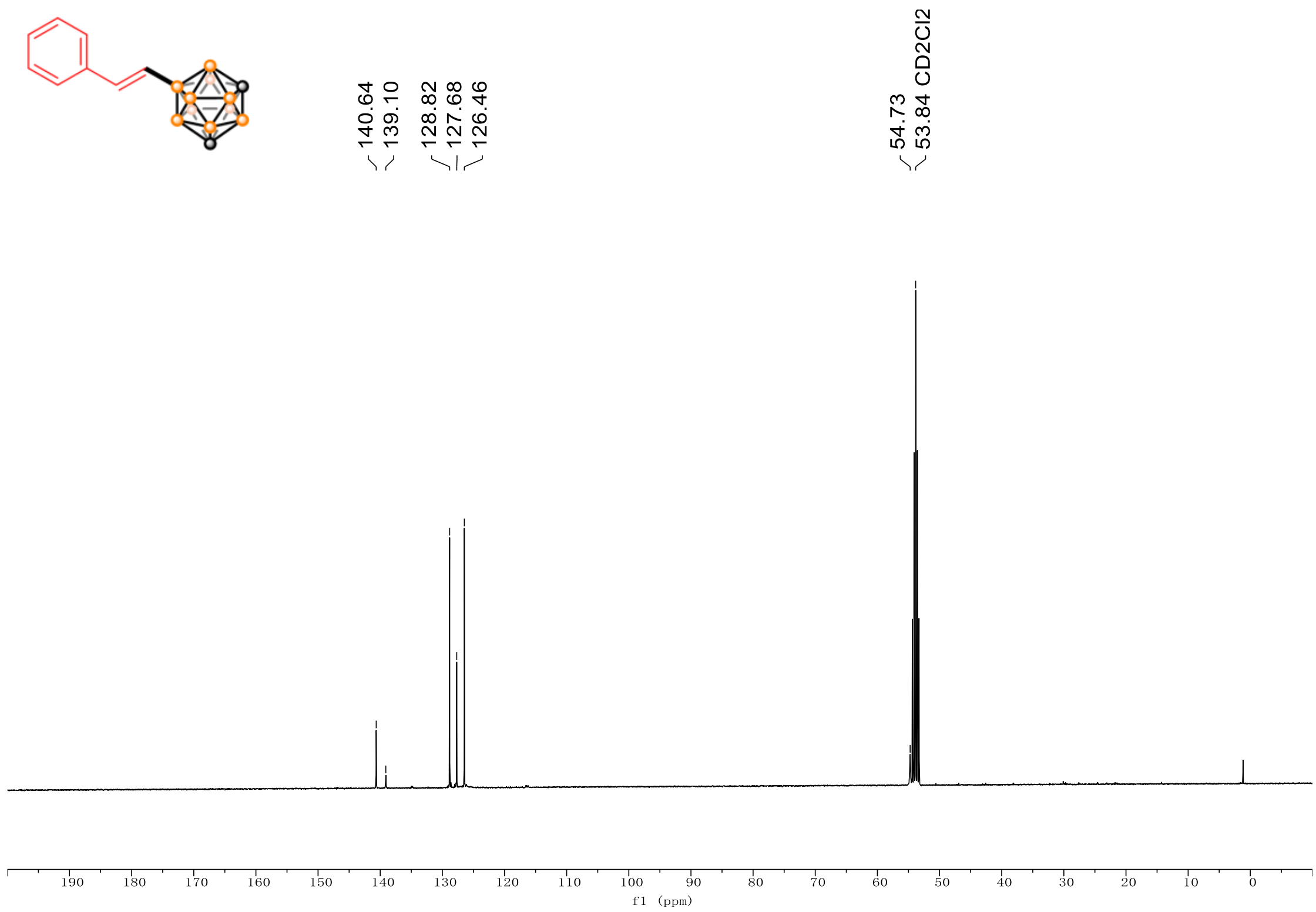

$^{13}C\{^{1}H\}$ NMR (101 MHz, CD_2Cl_2) of **3fa**

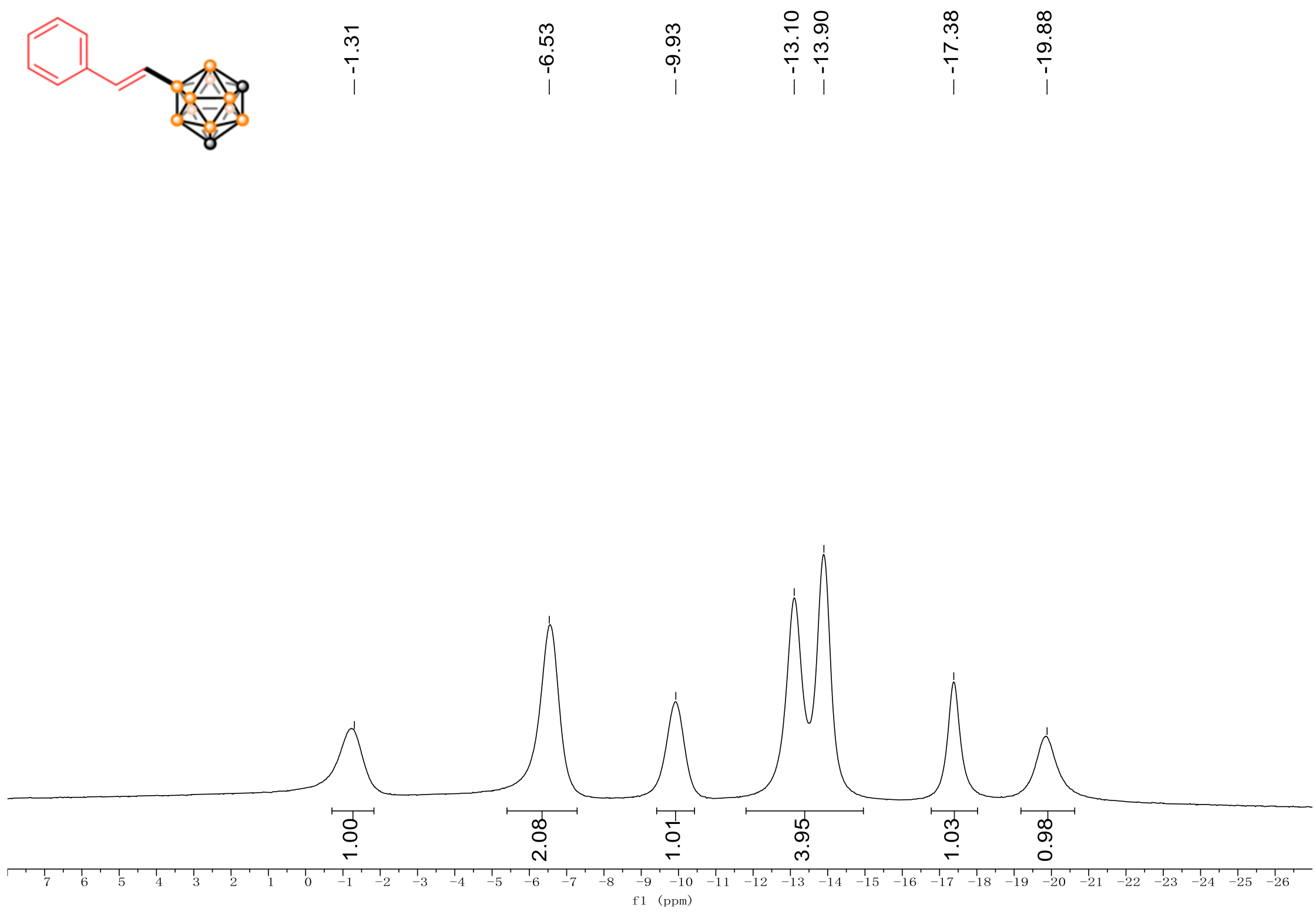

^{11}B{^{1}H} NMR (128 MHz, CD_2Cl_2) of **3fa**

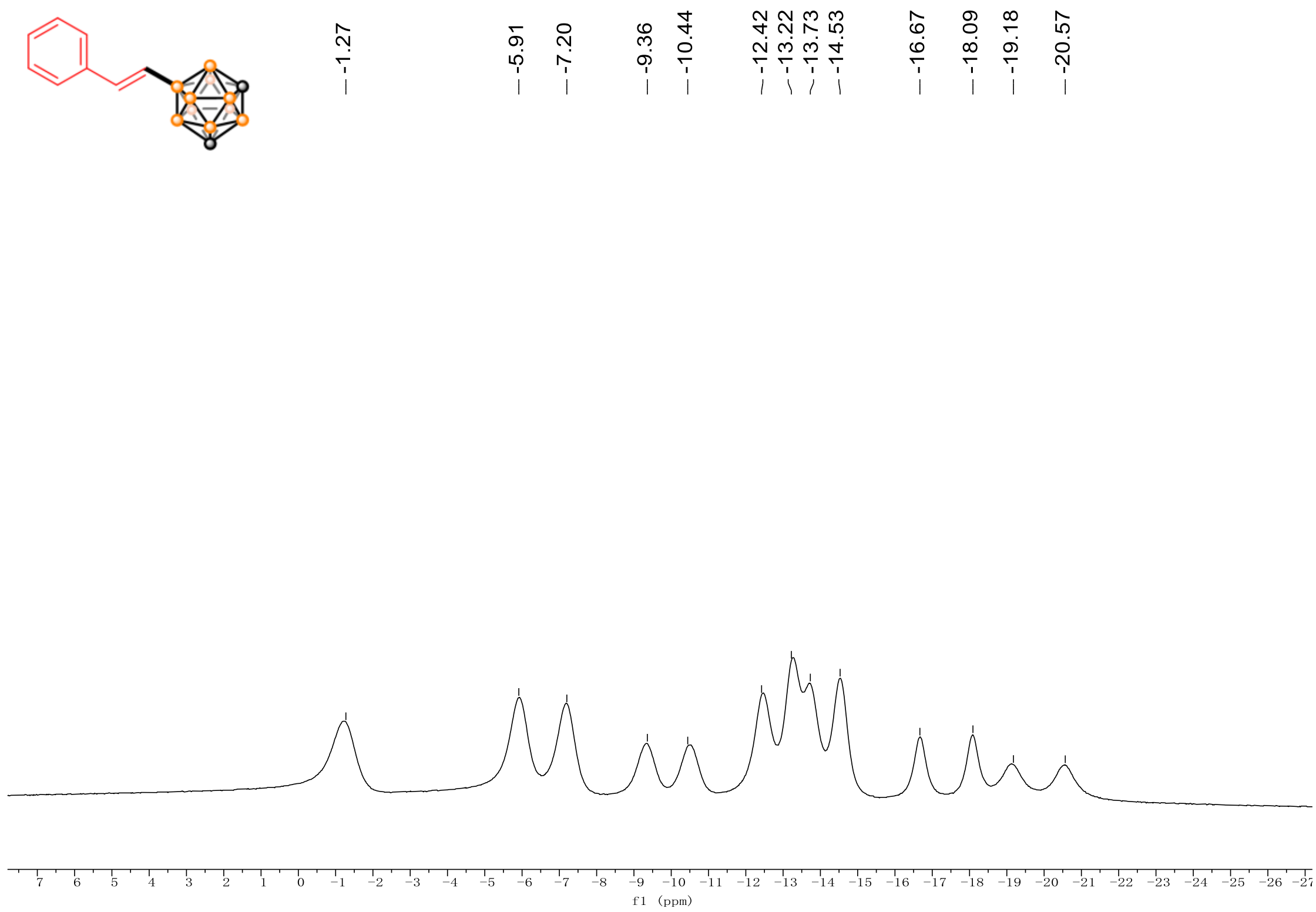

^{11}B NMR (128 MHz, CD_2Cl_2) of **3fa**

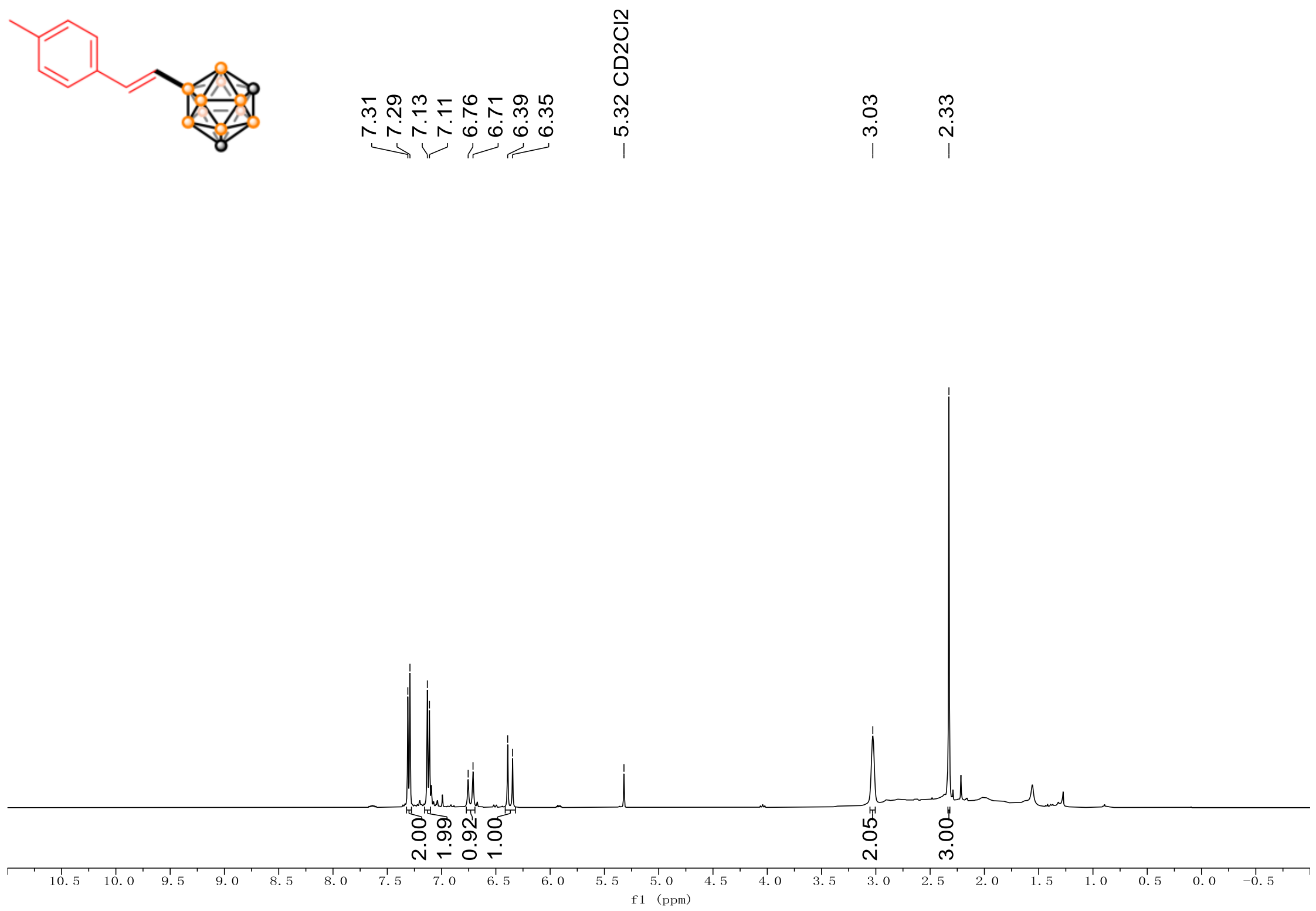

^{1}H NMR (400 MHz, CD_2Cl_2) of **3fb**

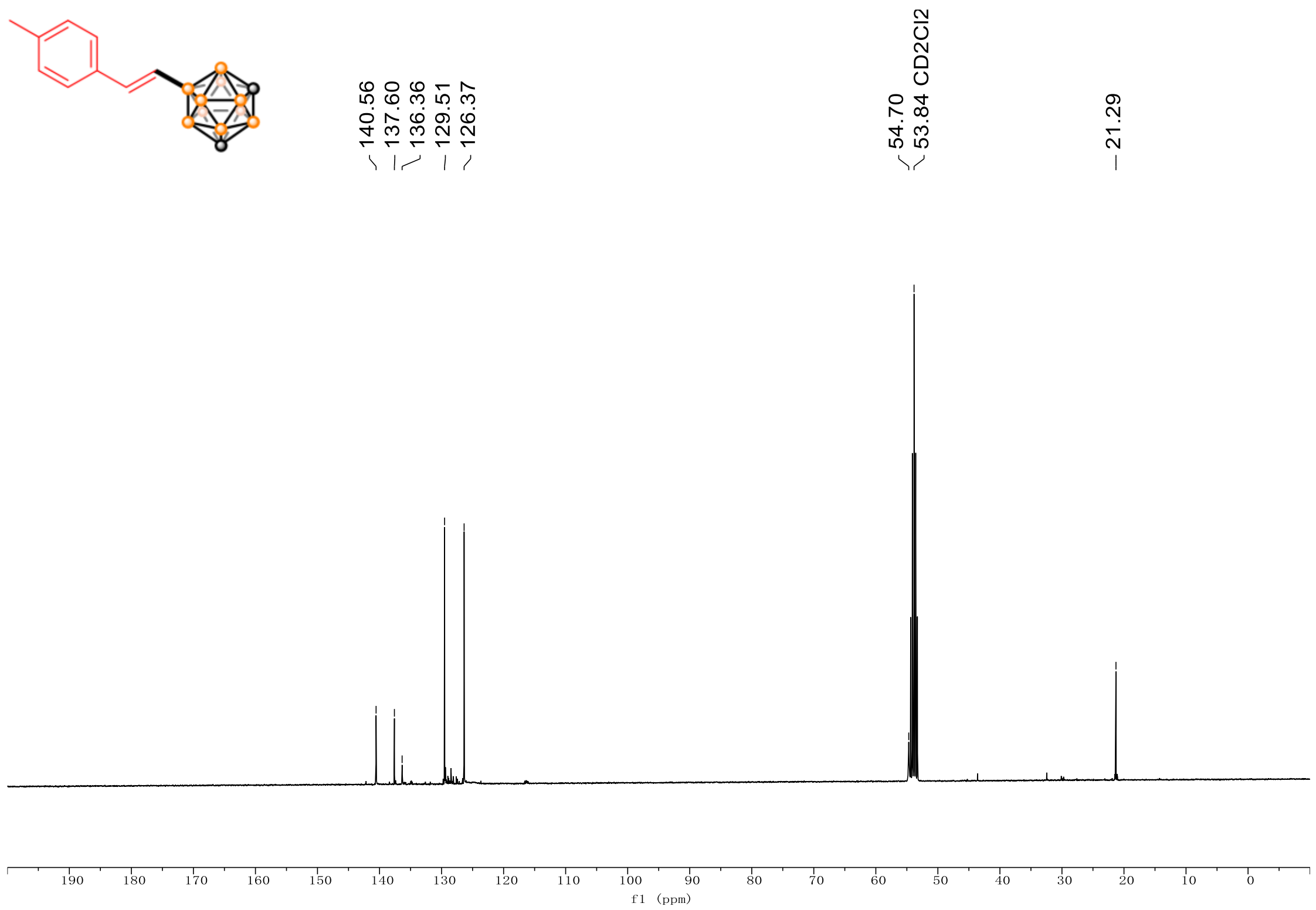

$^{13}C\{^{1}H\}$ NMR (101 MHz, CD_2Cl_2) of **3fb**

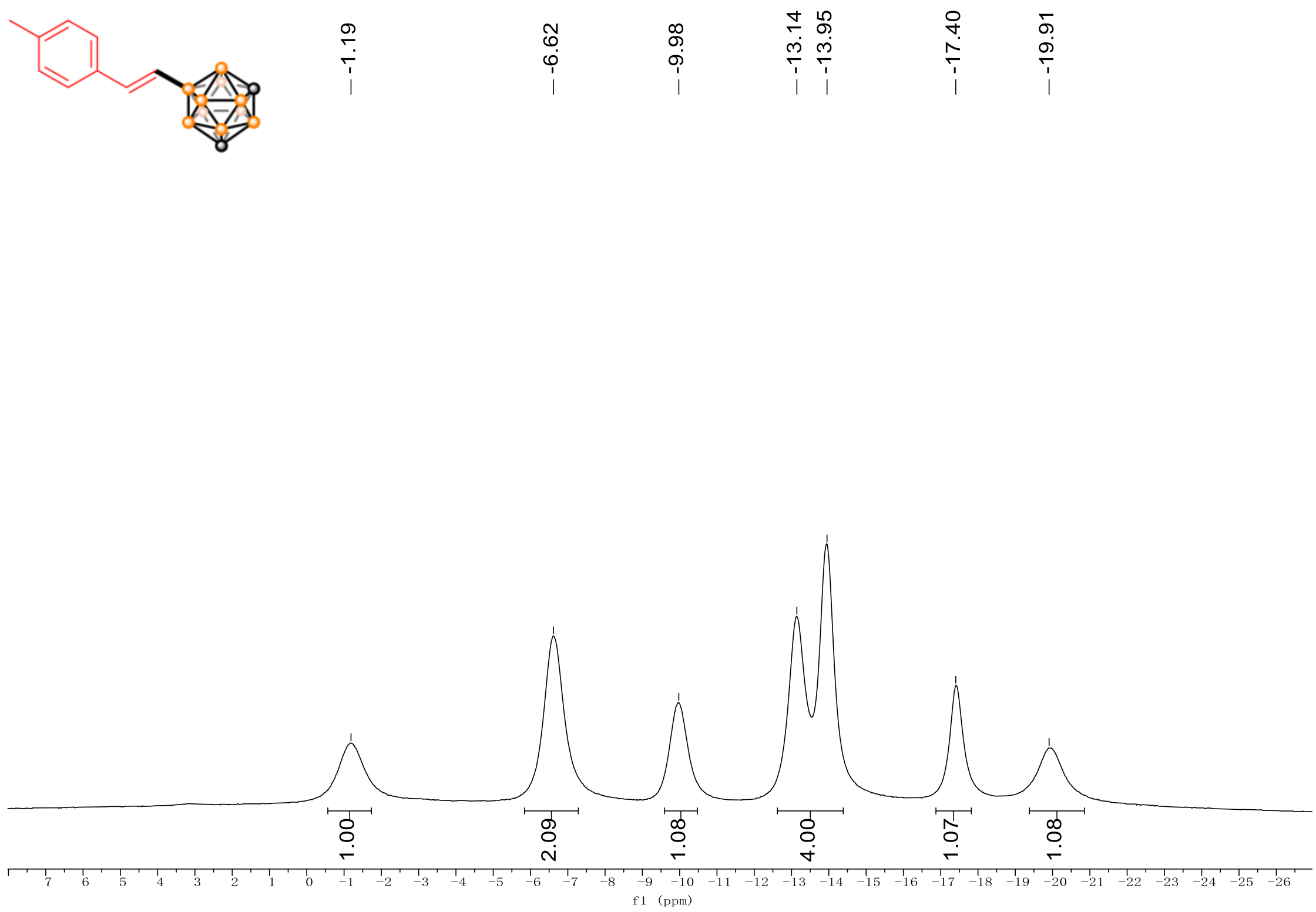

$^{11}B\{^1H\}$ NMR (128 MHz, CD_2Cl_2) of **3fb**

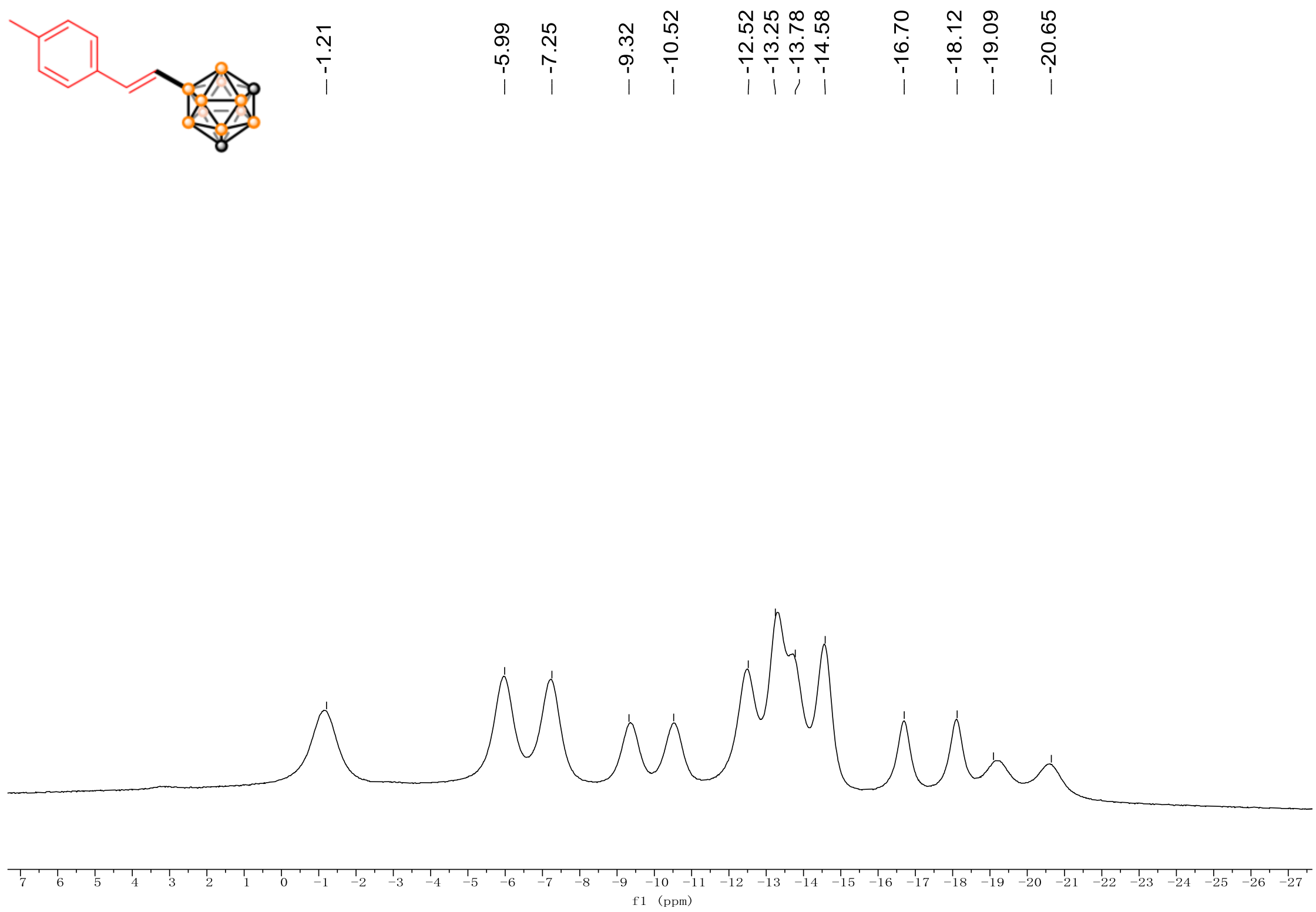

^{11}B NMR (128 MHz, CD_2Cl_2) of **3fb**

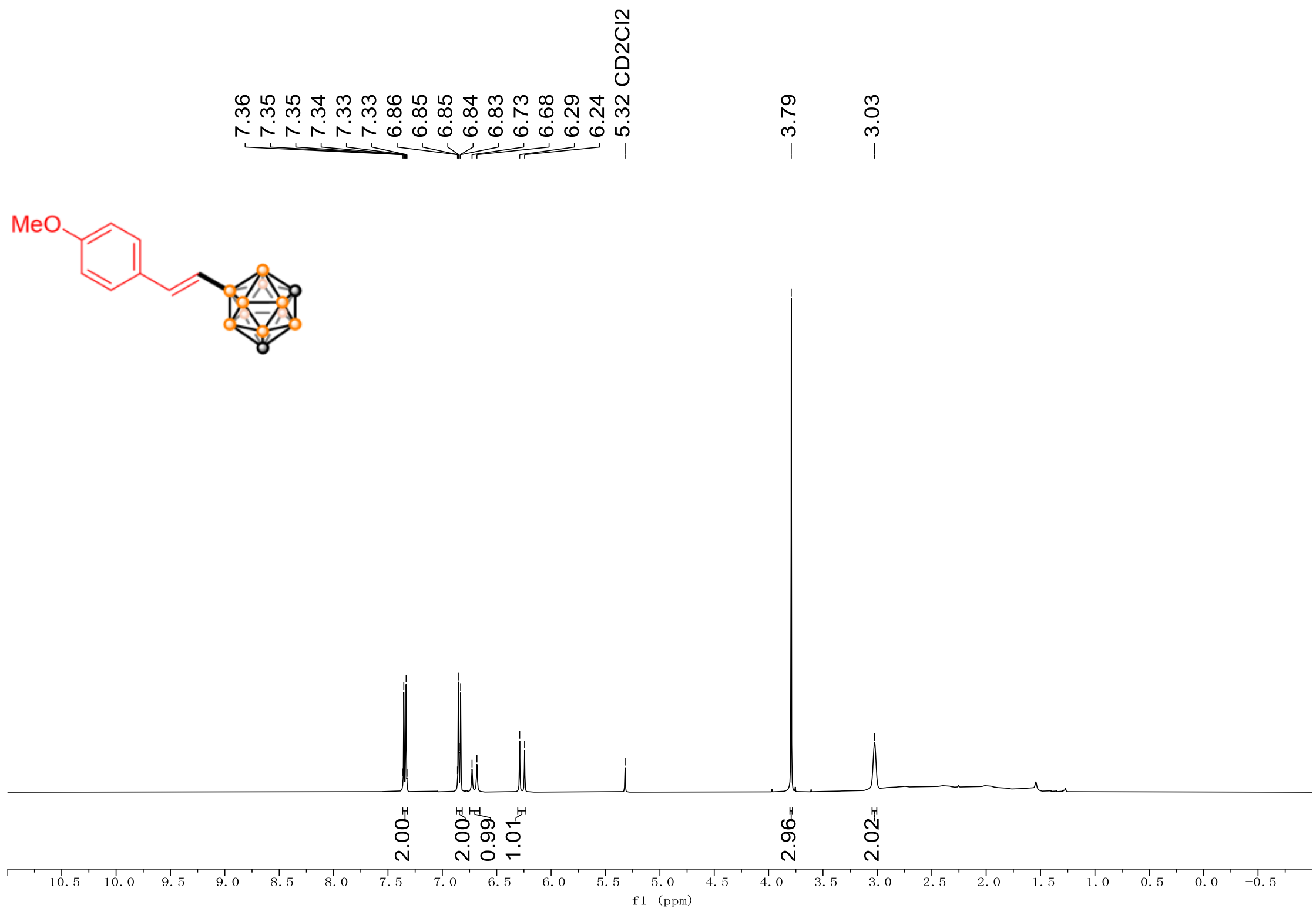

^{1}H NMR (400 MHz, CD_2Cl_2) of **3ff**

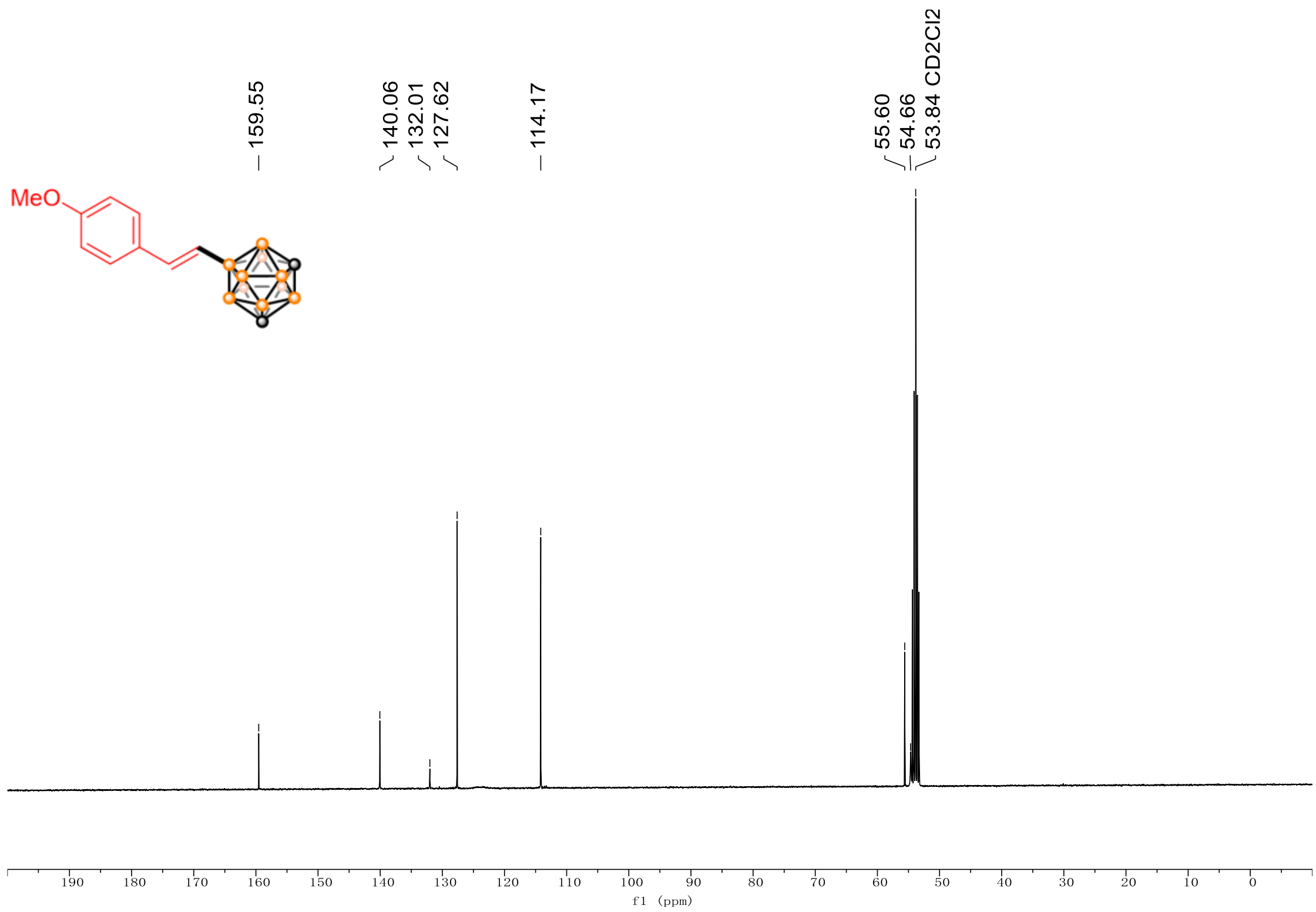

$^{13}C\{^{1}H\}$ NMR (101 MHz, CD_2Cl_2) of **3ff**

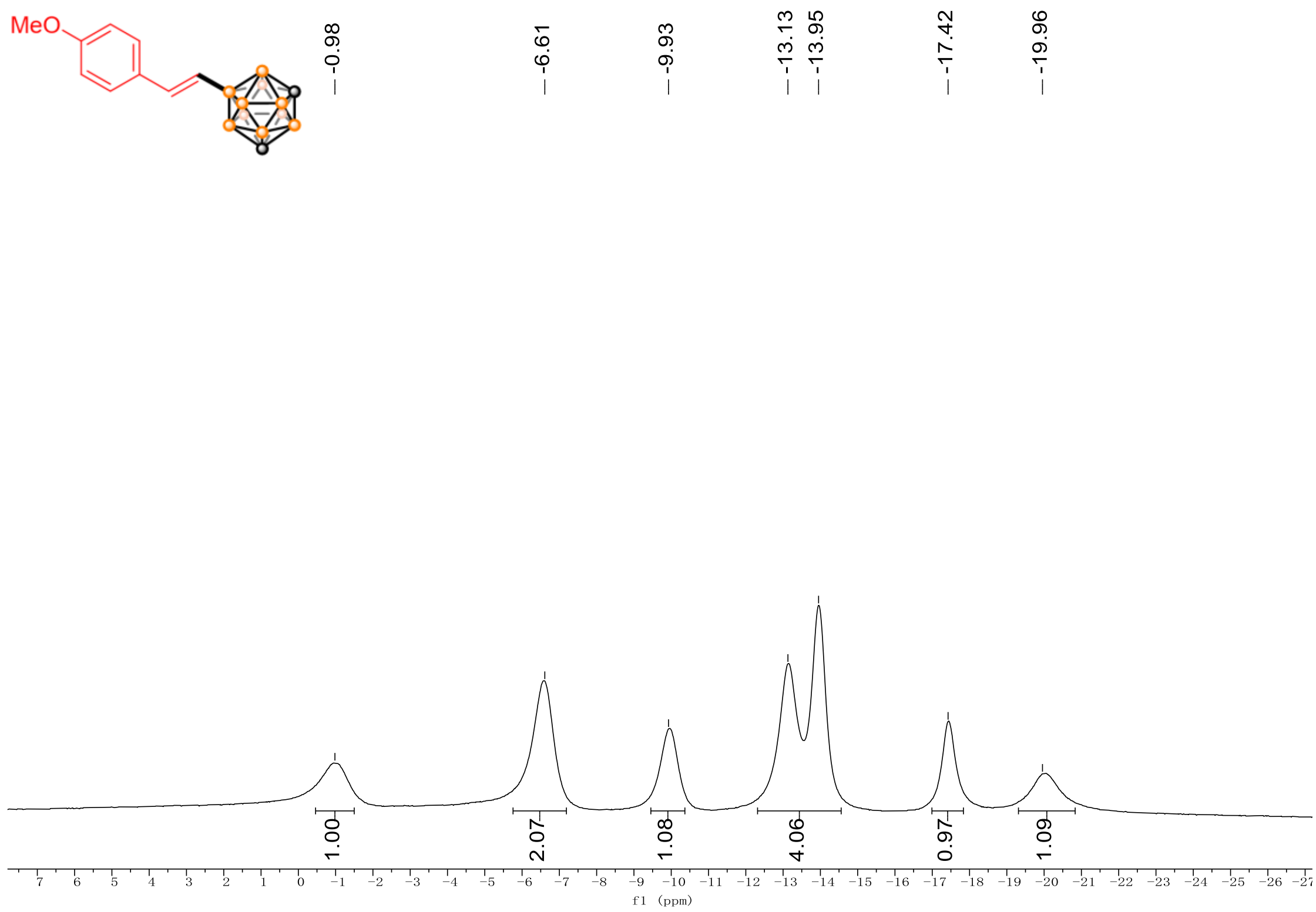

$^{11}B\{^{1}H\}$ NMR (128 MHz, CD_2Cl_2) of **3ff**

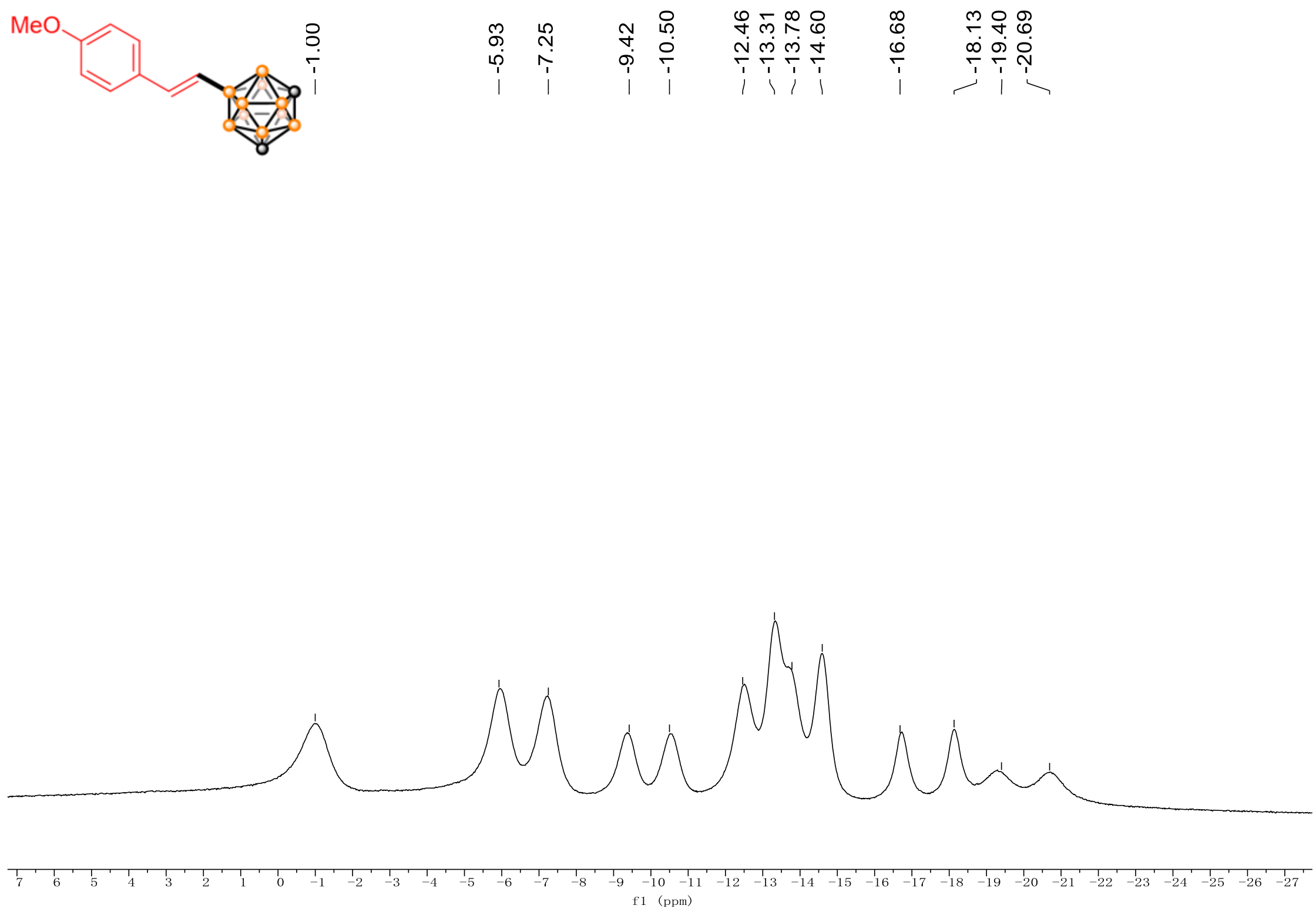

^{11}B NMR (128 MHz, CD_2Cl_2) of **3ff**

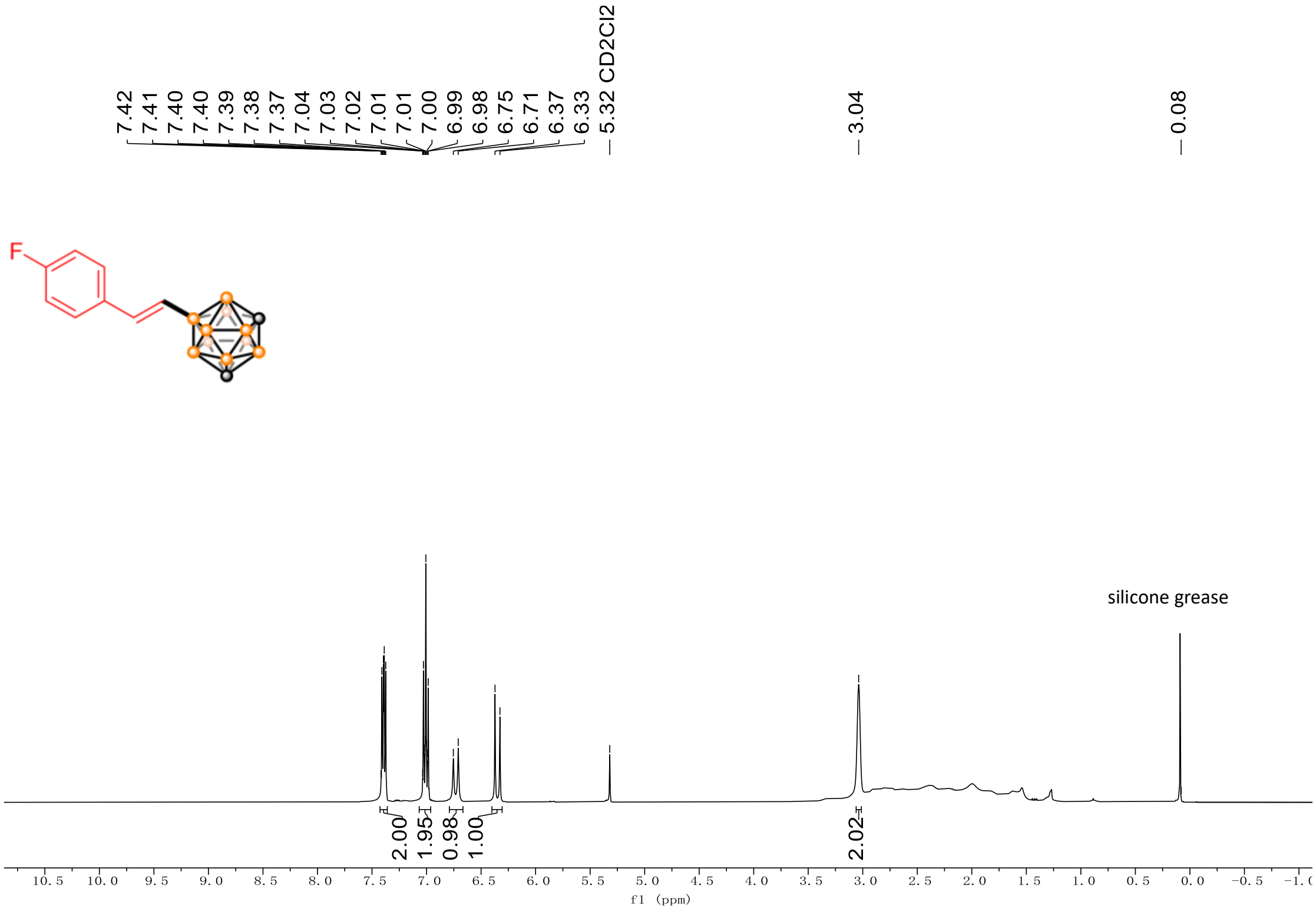

^{1}H NMR (400 MHz, CD_2Cl_2) of **3fi**

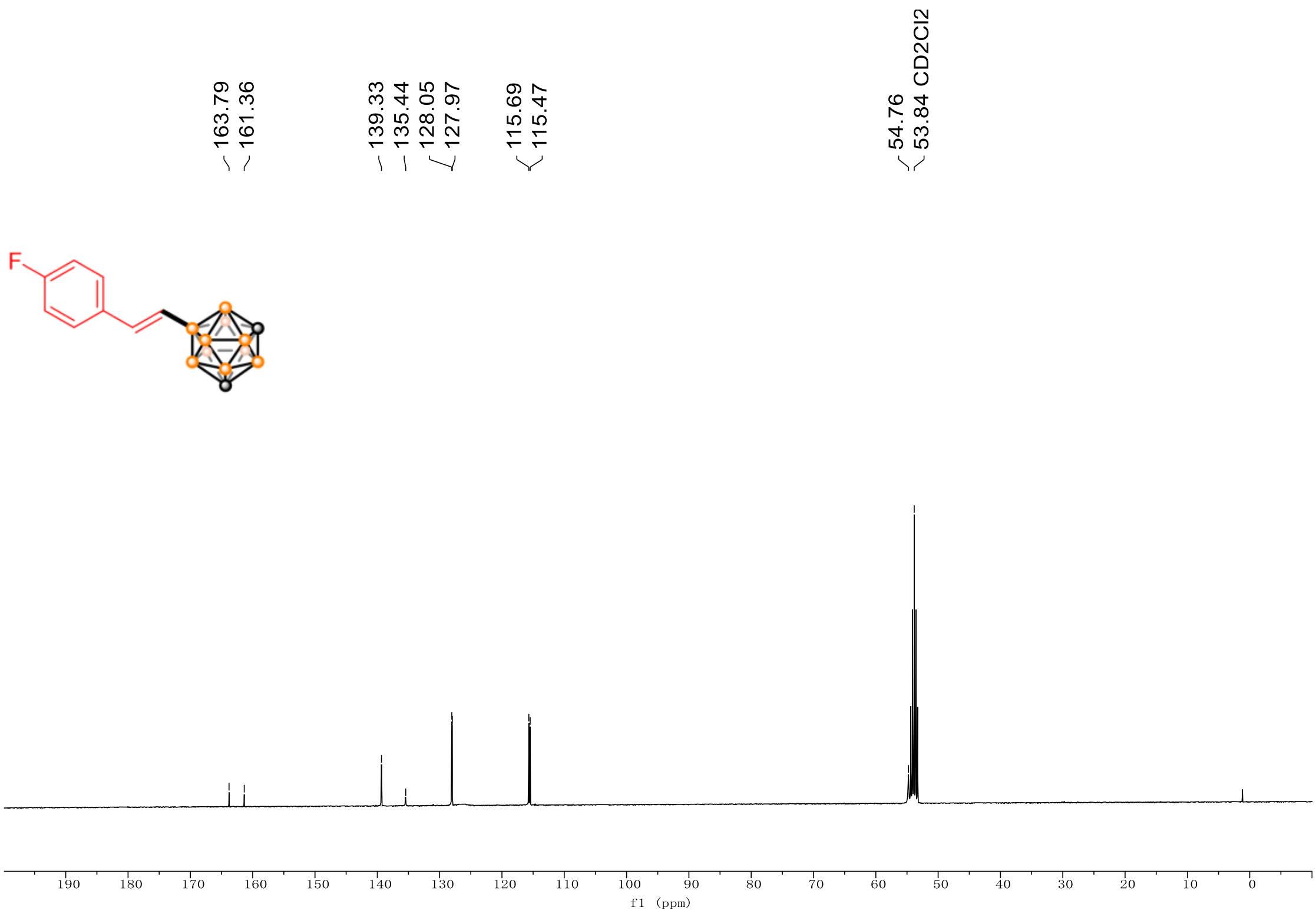

^{13}C{^{1}H} NMR (101 MHz, CD_2Cl_2) of **3fi**

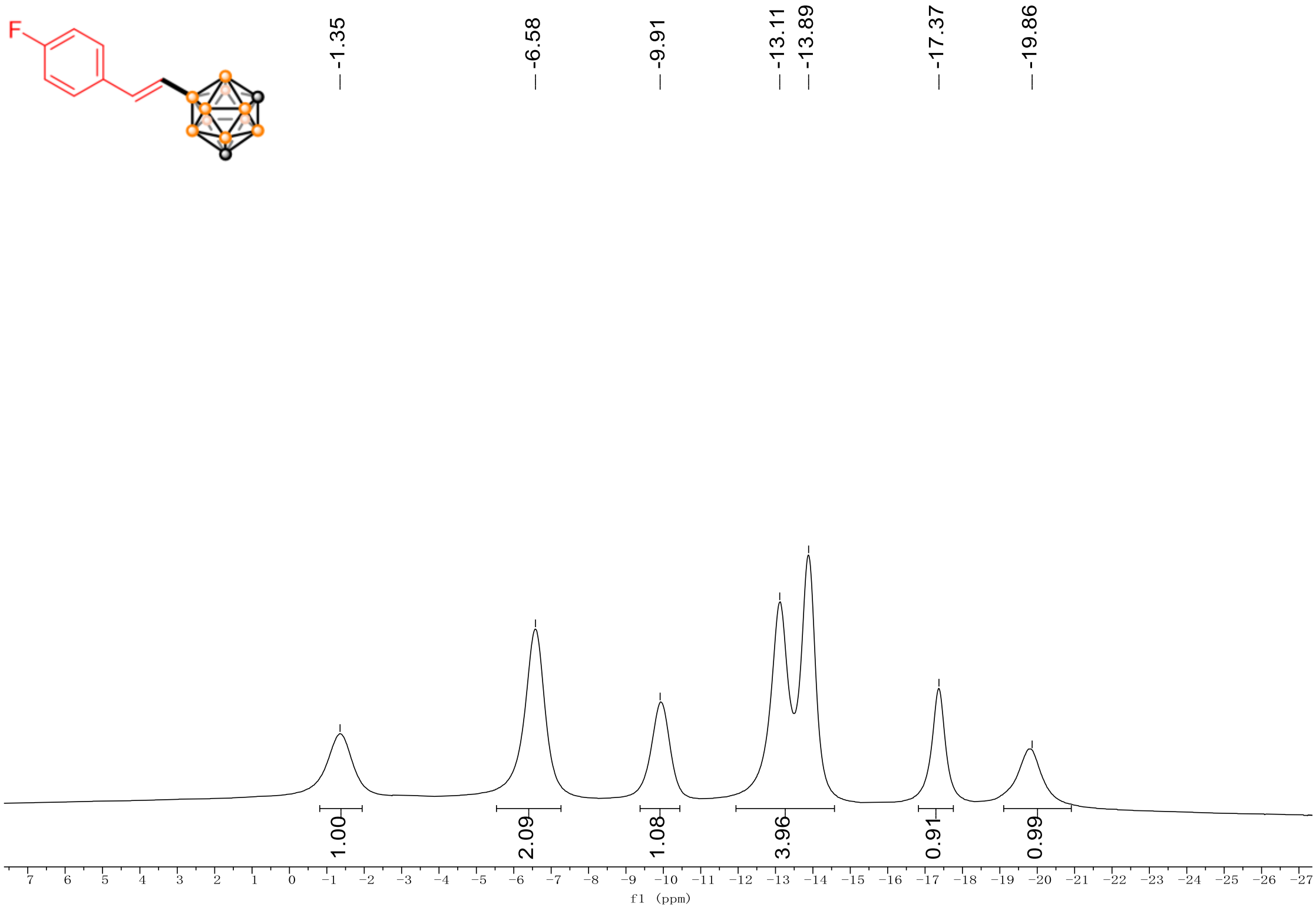

$^{11}B\{^1H\}$ NMR (128 MHz, CD_2Cl_2) of **3fi**

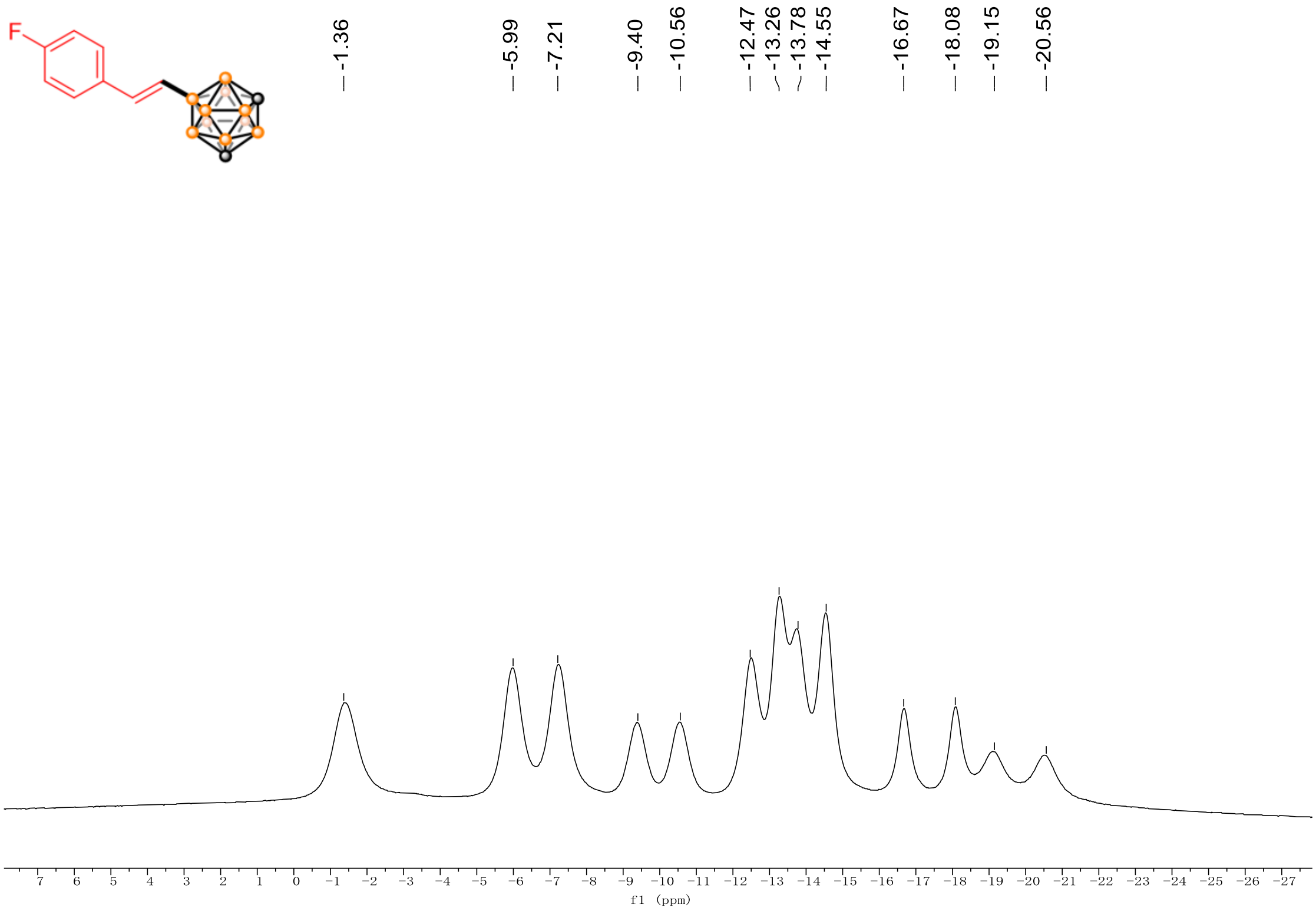

^{11}B NMR (128 MHz, CD_2Cl_2) of **3fi**

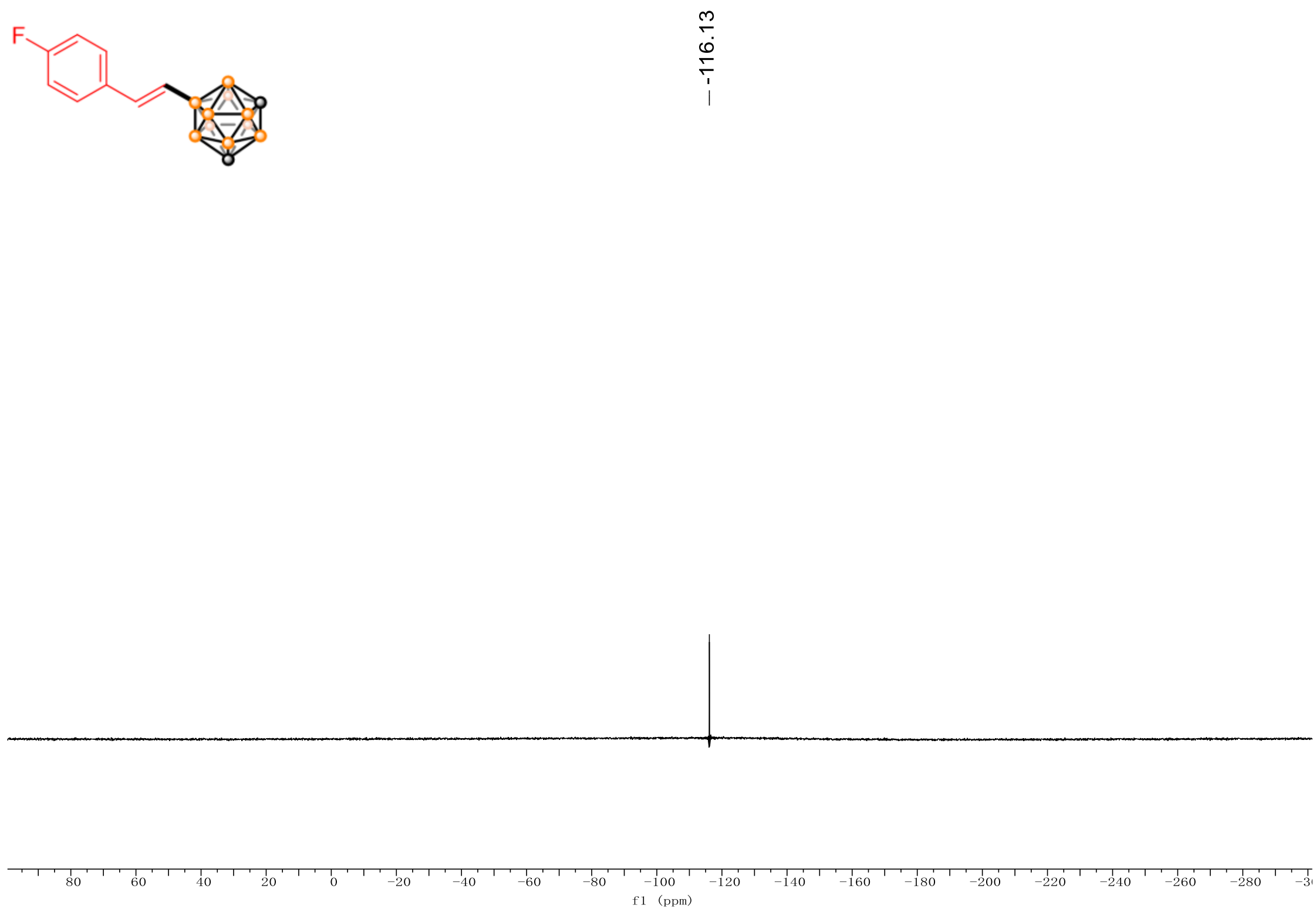

^{19}F NMR (377 MHz, CD_2Cl_2) of **3fi**

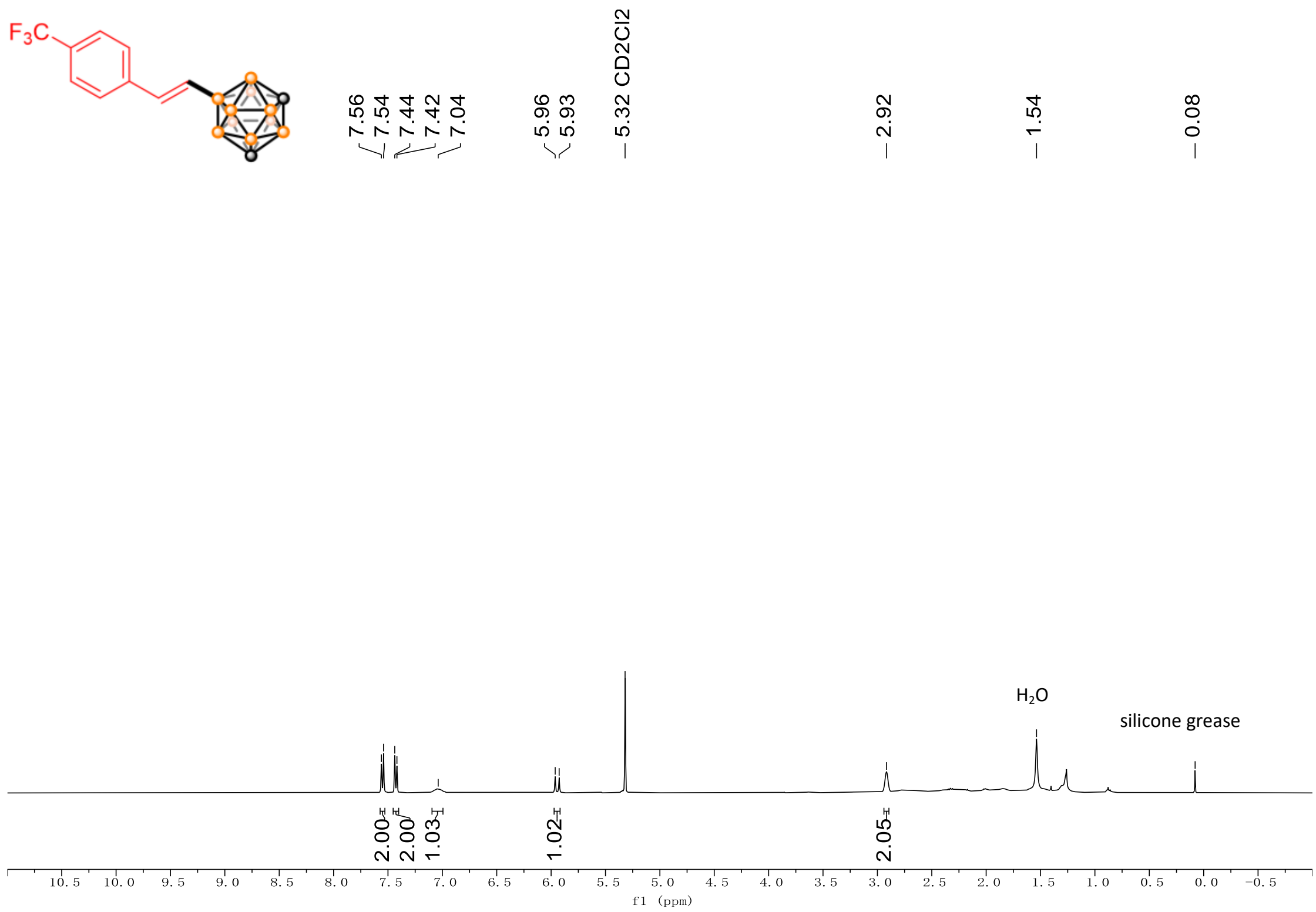

^{1}H NMR (400 MHz, CD_2Cl_2) of **3fl**

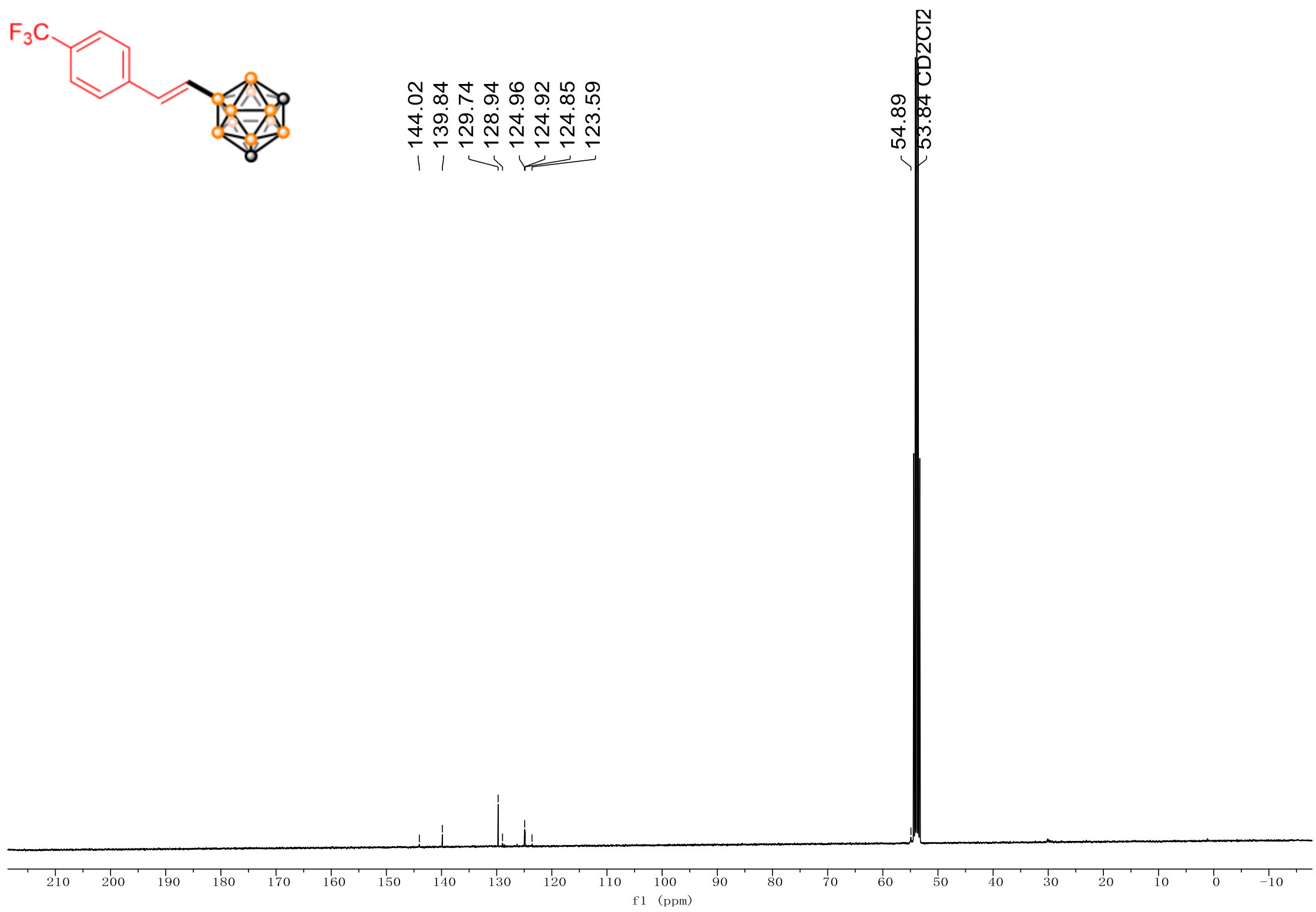

$^{13}C\{^{1}H\}$ NMR (101 MHz, CD_2Cl_2) of **3fl**

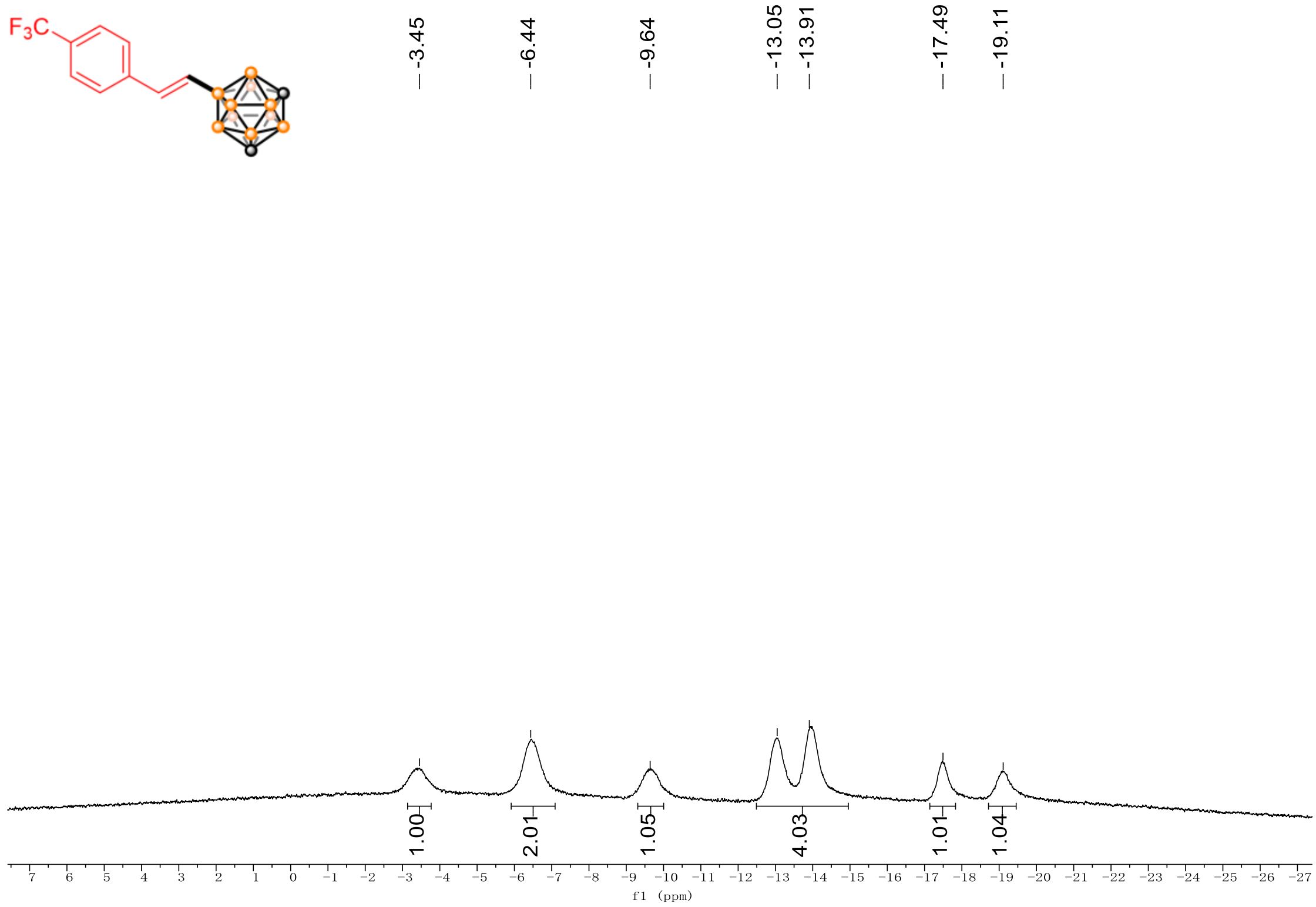

$^{11}B\{^{1}H\}$ NMR (128 MHz, CD_2Cl_2) of **3fl**

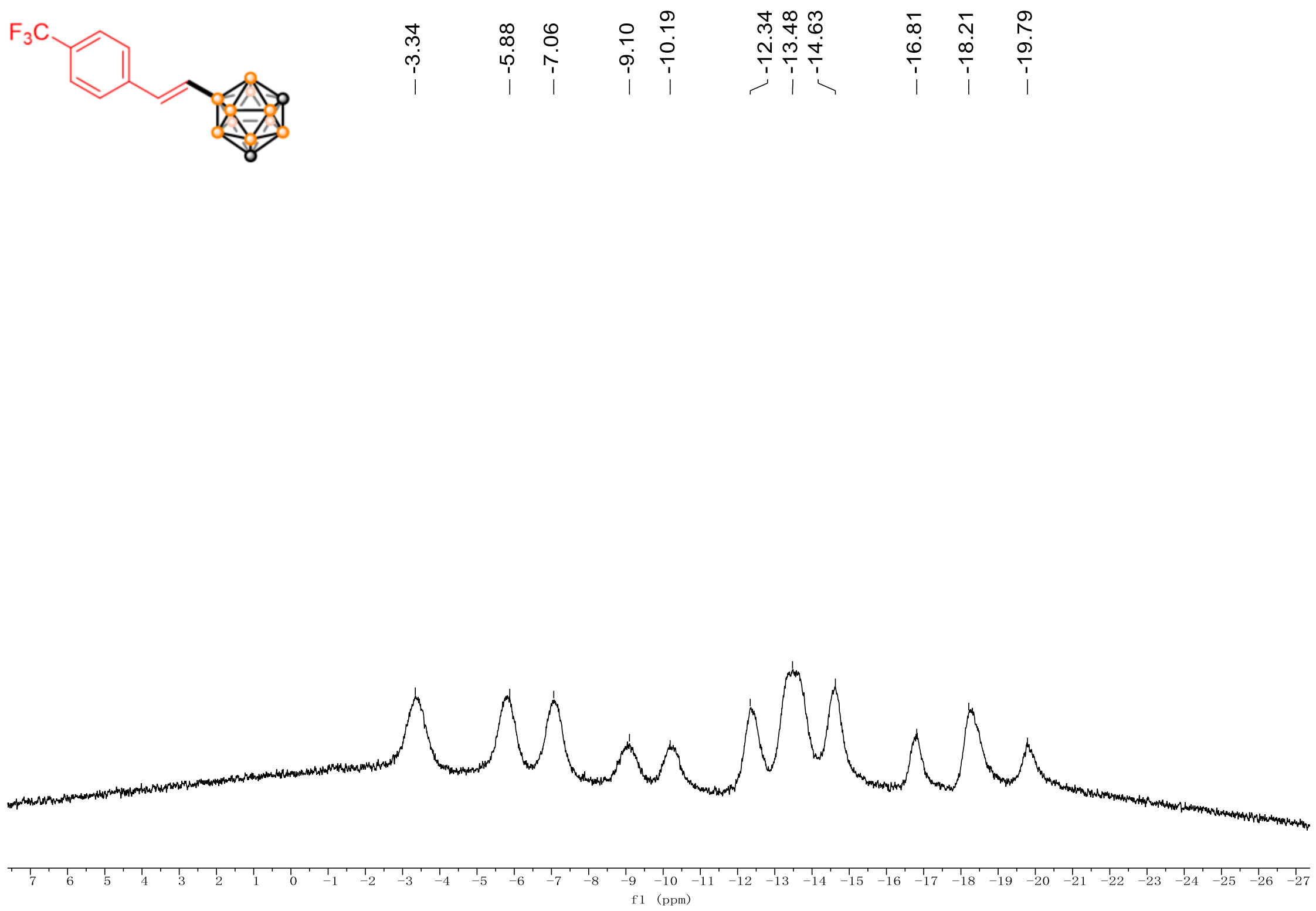

^{11}B NMR (128 MHz, CD_2Cl_2) of **3fl**

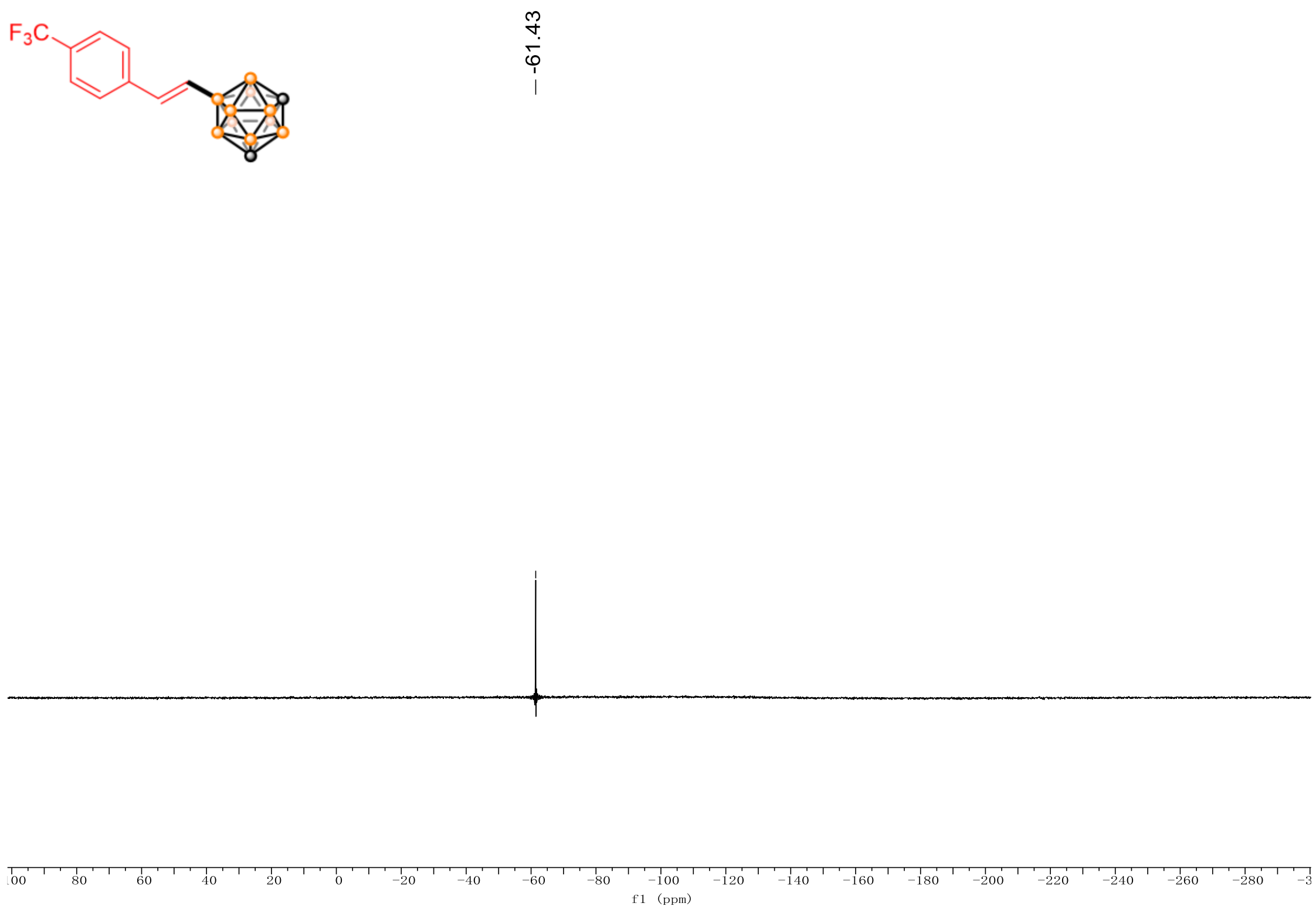

^{19}F NMR (377 MHz, CD_2Cl_2) of **3fl**

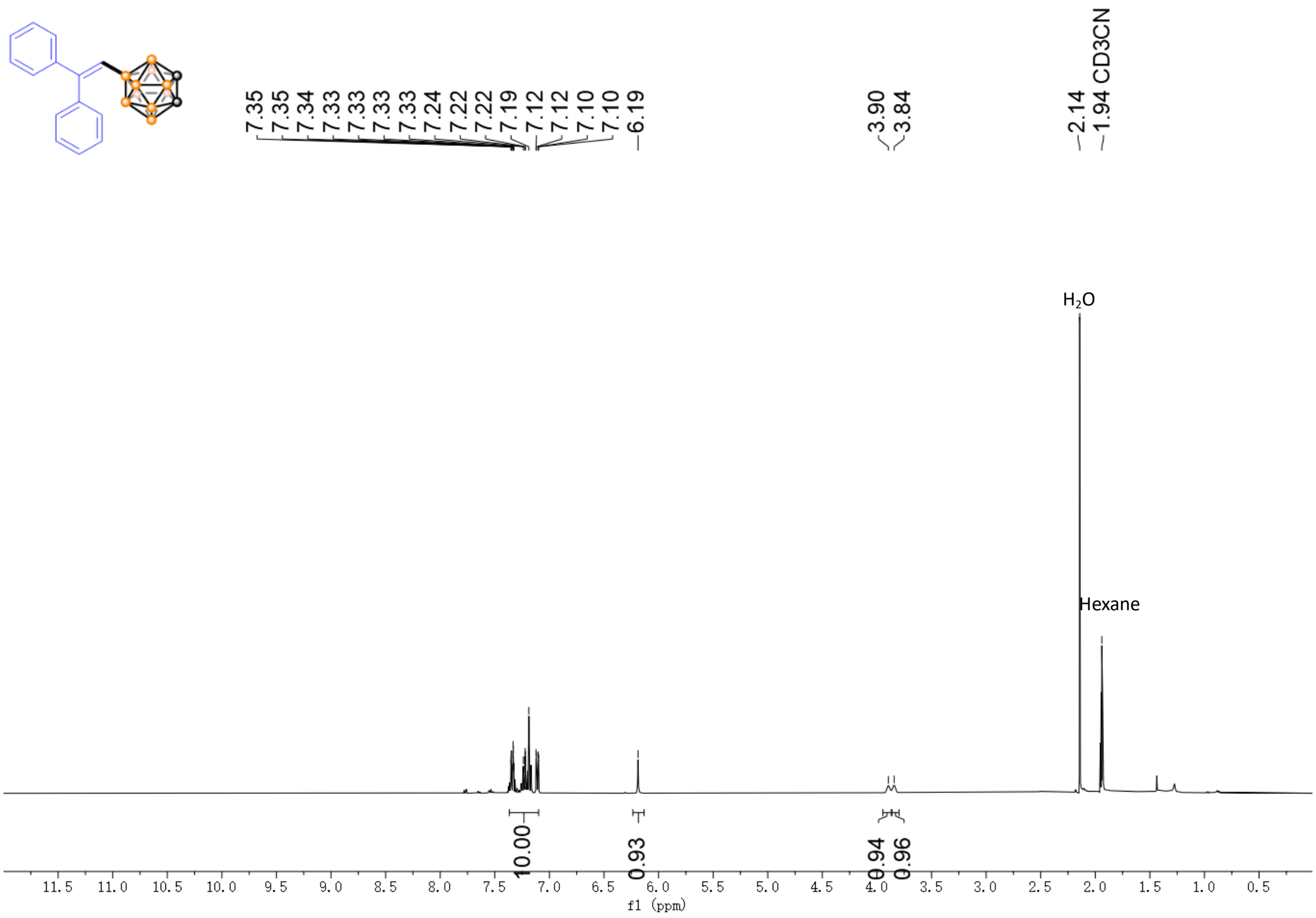

^{1}H NMR (400 MHz, CD_3CN) of **3an**

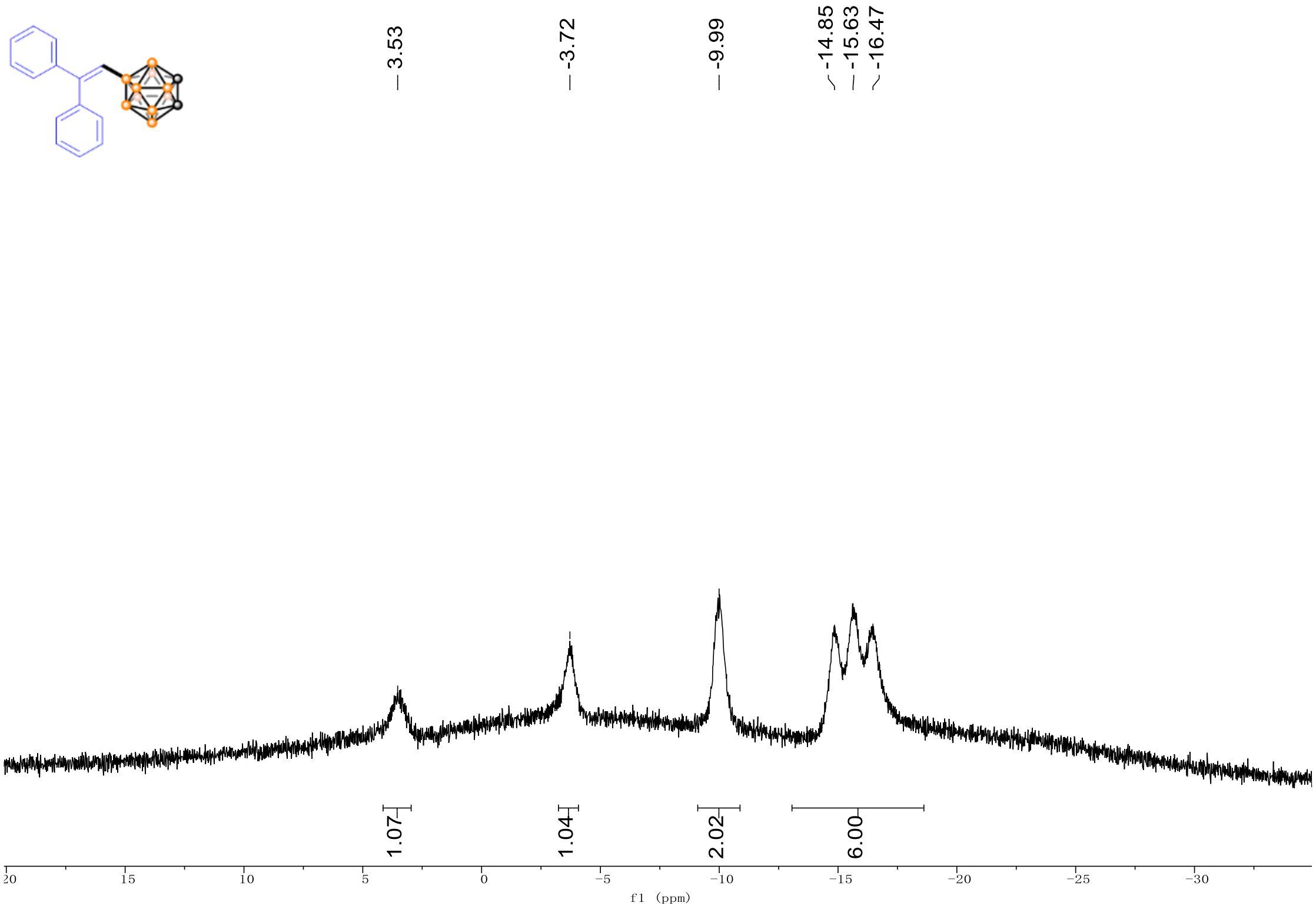

$^{11}B\{^{1}H\}$ NMR (128 MHz, CD_3CN) of **3an**

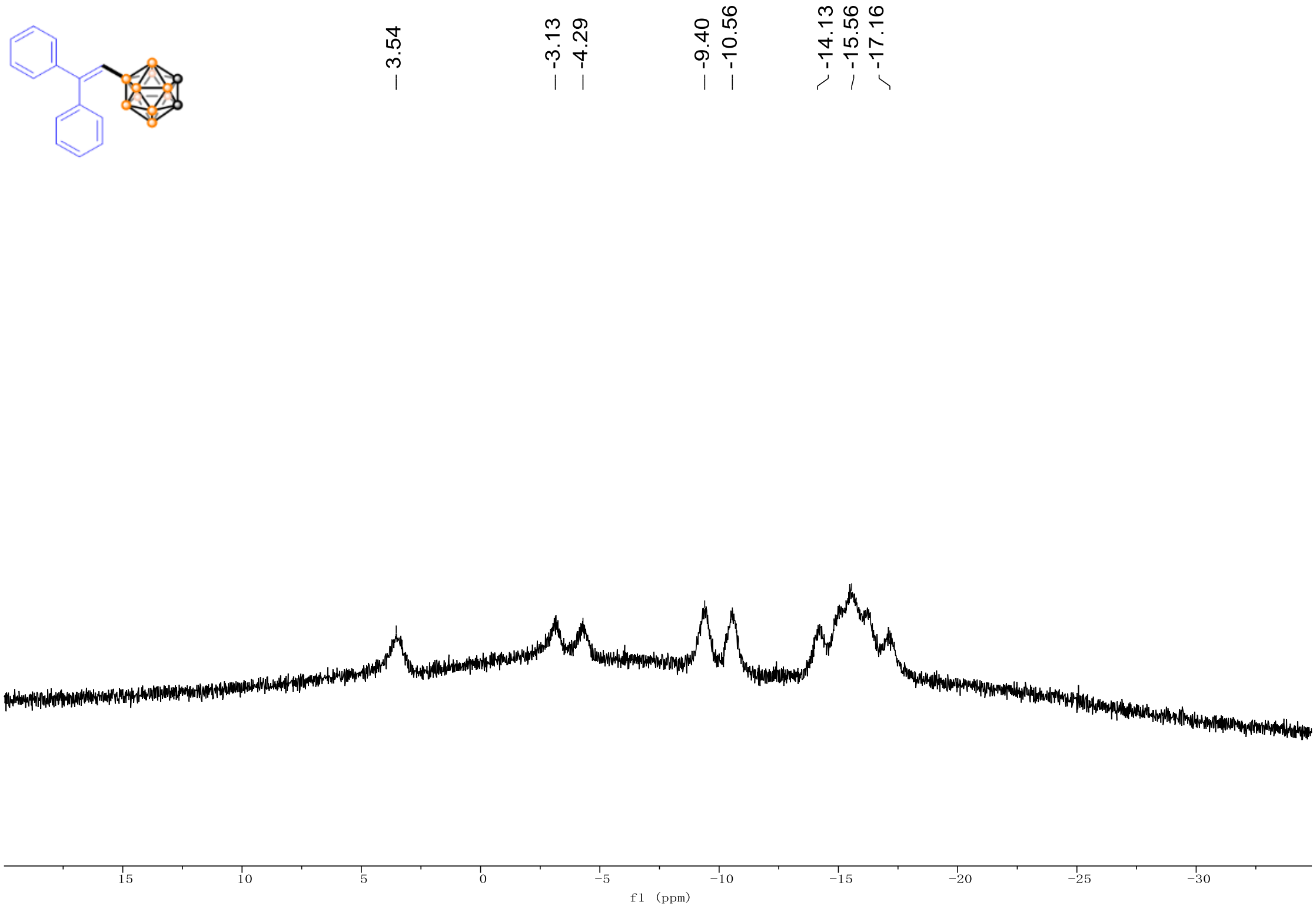

^{11}B NMR (128 MHz, CD_3CN) of **3an**

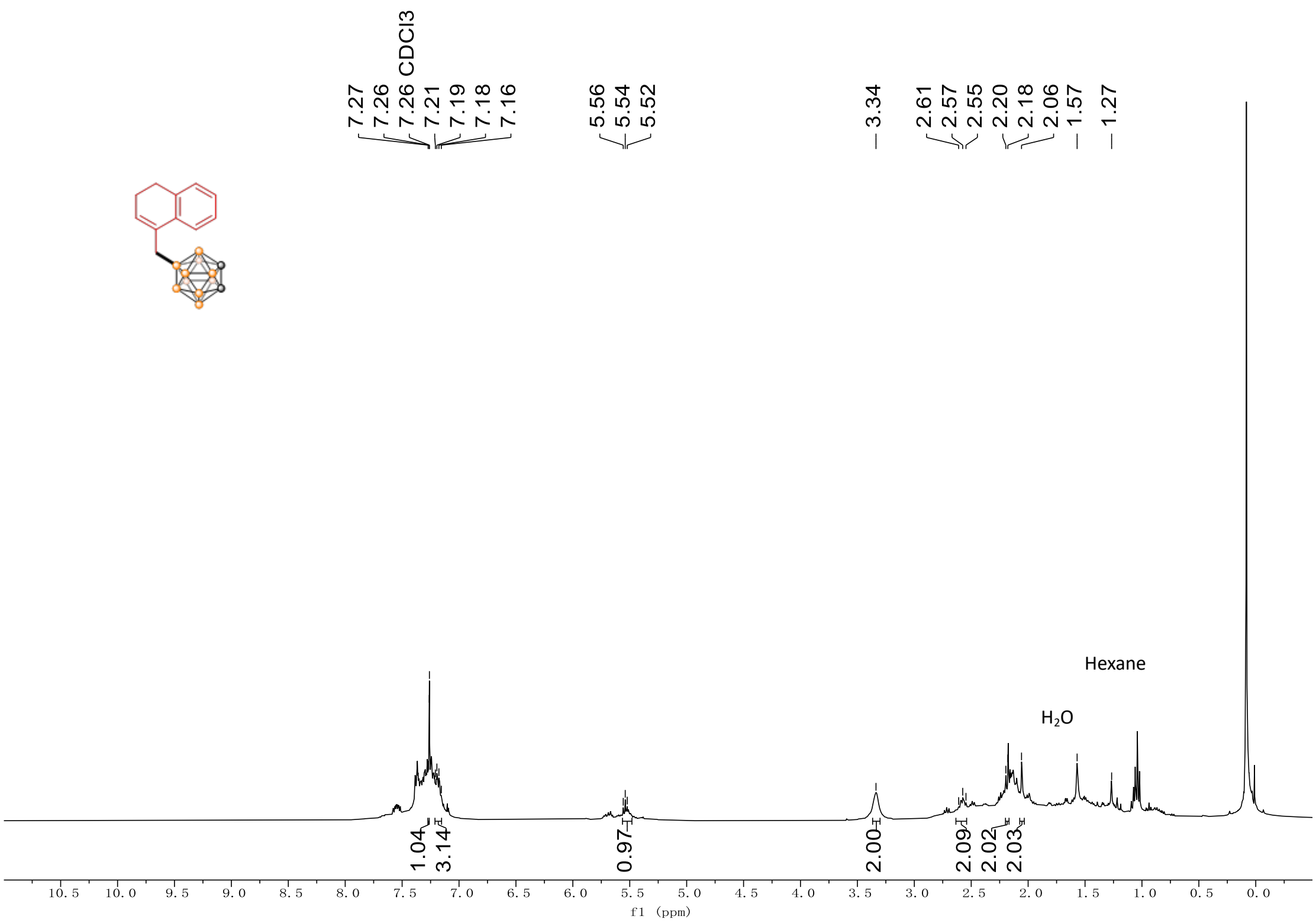

^{1}H NMR (400 MHz, CD_3CN) of

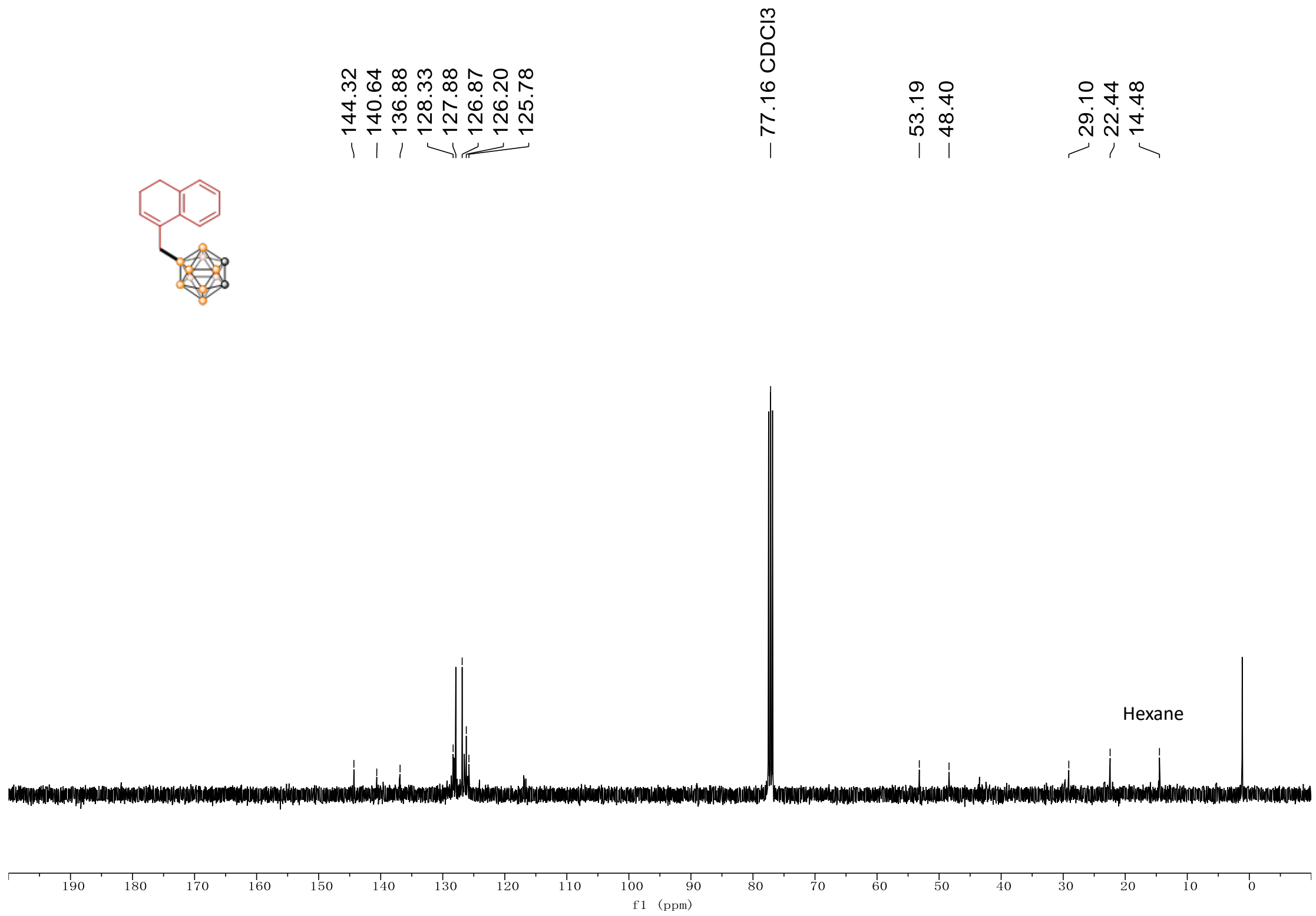

$^{13}C\{^{1}H\}$ NMR (126 MHz, CD_3CN) of

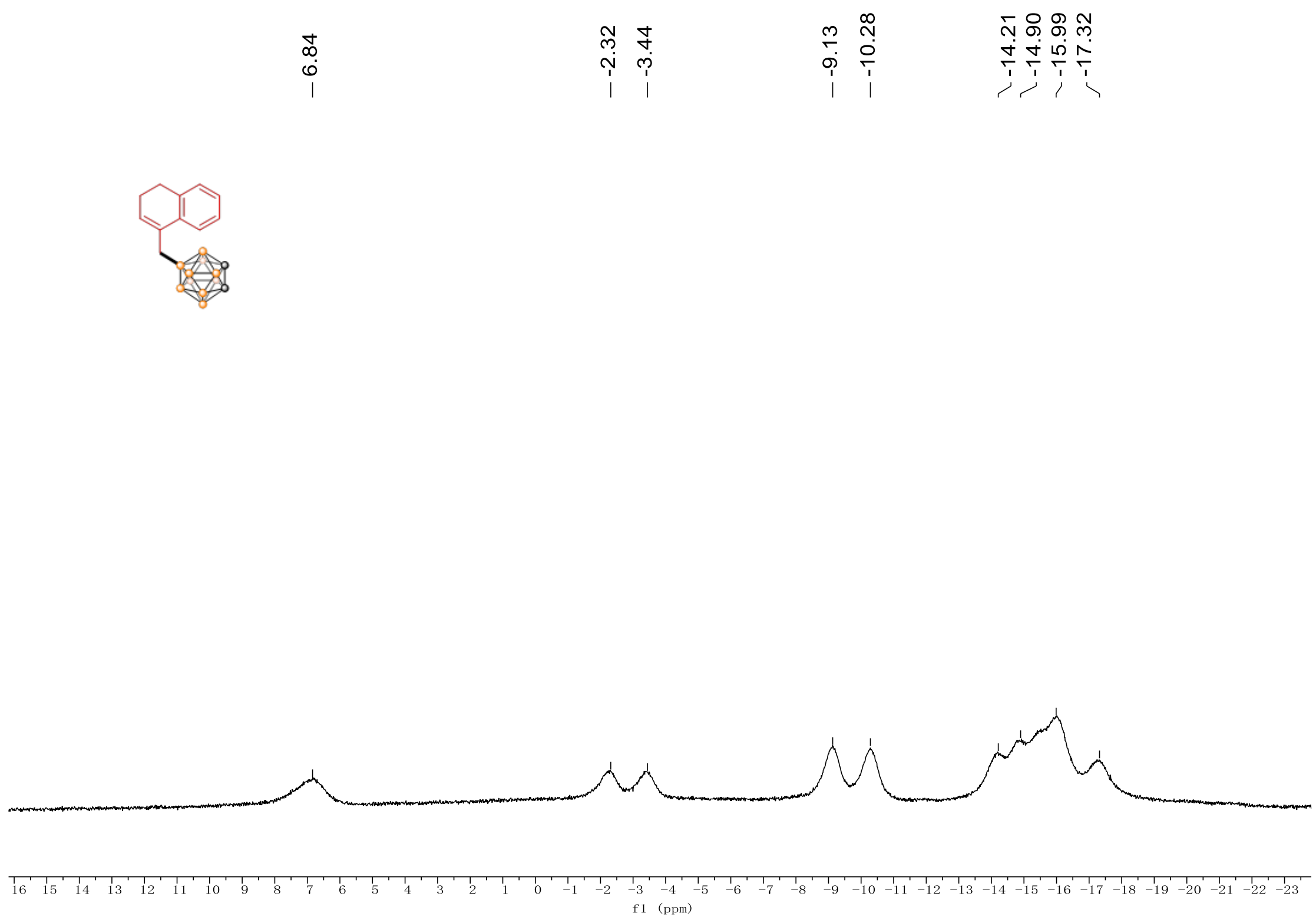

$^{11}B\{^{1}H\}$ NMR (128 MHz, CD_3CN) of

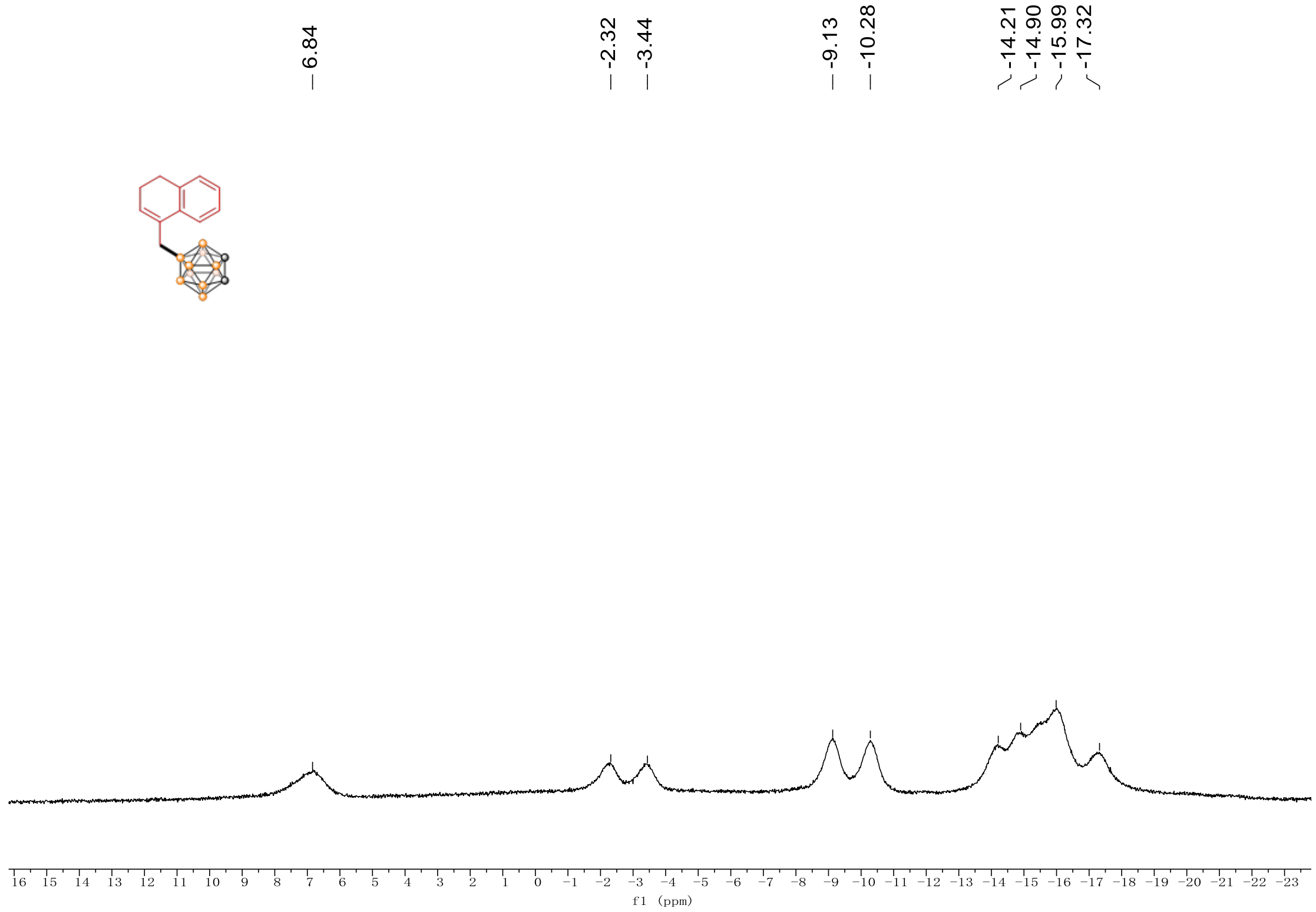

^{11}B NMR (128 MHz, CD_3CN) of
